# Cancer pain knowledge and attitudes of healthcare professionals: A systematic review of surveys and their measurement properties

**DOI:** 10.1177/20494637261442745

**Published:** 2026-04-13

**Authors:** Martin Galligan, Rebecca Verity, Theresa Wiseman, Emma Briggs

**Affiliations:** 1 Royal Marsden NHS Foundation Trust, Royal Marsden School, London, UK; 2 Florence Nightingale Faculty of Nursing, Midwifery & Palliative Care, King’s College London, London, UK

**Keywords:** pain, cancer, Cancer-related pain, pain education, knowledge

## Abstract

**Background:**

Effective management of cancer-related pain (CRP) requires support from knowledgeable healthcare professionals. Existing literature states that healthcare professionals’ knowledge of CRP is poor. However, there is no consistency in how knowledge and attitudes are assessed or the standards against which they are measured. This systematic review evaluates the cancer-related content and psychometric properties of surveys used to assess knowledge of CRP.

**Method:**

Using JBI methodology for Systematic Reviews of Measurement Properties, the search was conducted in MEDLINE, EMBASE, CINAHL and PsycINFO (limited to 2011–2024). Screening and extraction were completed by two researchers using the COVIDENCE system. The COSMIN Risk of Bias checklist was used to assess psychometric properties, and additional data were extracted on content feasibility.

**Results:**

A total of 1024 papers were identified, and 37 were included in the final analysis. Sixteen different surveys were found, with four being used in multiple studies. No survey was deemed superior, and all were rated poorly using the COSMIN Risk of Bias checklist. The CRP content within the surveys varied significantly, with none capturing the complexity of CRP. The feasibility of administering these surveys in practice was not reported in the studies examined.

**Conclusion:**

The wide variation in the design and content of surveys identified makes it difficult to assess the current state of healthcare professionals’ knowledge of CRP. This raises questions about the validity and reliability of their conclusions, as they lack a robust evaluation of psychometric properties. Further research is needed to accurately assess healthcare professionals’ knowledge of CRP.

## Introduction

Cancer-related pain (CRP) is a complex, multidimensional phenomenon that affects patients’ physical, spiritual, social, and psychological functioning.^1,2^ While the exact prevalence of CRP is difficult to determine, it is estimated to affect up to 54% of individuals with advanced disease and 35% of those who have completed curative treatment for cancer.^3^ CRP is commonly reported following a cancer diagnosis and occurs alongside other cancer-related symptoms.^4^ This results in individuals experiencing pain that varies in location, intensity, duration, and impact.^5^ This makes the management of CRP challenging for healthcare professionals (HCPs). Given the prevalence and complexity of CRP, HCPs must be equipped to support those affected.

Two published reviews have explored the extent of CRP knowledge among HCPs.^6,7^ A 2017 narrative review^7^ and a 2019 systematic review^6^ reported that HCPs’ knowledge of CRP was poor. However, several limitations in these reviews diminish confidence in their findings. Both reviews utilised mean scores to assess knowledge levels across the included studies. This approach does not allow for a transparent comparison across studies due to variations in the survey designs used to capture knowledge levels. Furthermore, neither review addressed the content, validity, and reliability of the surveys used to measure knowledge, aside from simple internal consistency for some measures. This further limits the ability to explore the strengths and limitations of HCPs knowledge of CRP. Both reviews^6,7^ document commonly used surveys such as the Knowledge and Attitudes Survey Regarding Pain (KASRP),^8^ which was not designed to assess knowledge of CRP. The reviews also reported frequent use of self-designed surveys with limited or no information on their content or reliability. Nonetheless, neither review addressed whether these notable methodological limitations affected the overall confidence in their findings and conclusions.

To improve the quality of care for patients with CRP, it is essential that they have access to HCPs with adequate knowledge to address their individual needs. While the current literature suggests that knowledge levels are poor, there are many limitations that prevent a comprehensive exploration of HCP knowledge of CRP. Further exploration of the reliability, validity and content of these surveys is needed to accurately measure HCP knowledge of CRP. This is key to enabling the development of HCPs and measuring the impact of future educational interventions.

This systematic review addresses the question, ‘What are the cancer-related content, measurement properties, and feasibility of surveys used to assess healthcare professionals’ knowledge and attitudes of cancer-related pain in contemporary cancer practice?’.

This paper reports on the first known systematic review to evaluate the properties of surveys used to assess HCP’s knowledge and attitudes towards CRP and to evaluate the surveys’ CRP content and feasibility of delivery. This review is the first phase of a mixed-method study that aims to understand the experience of CRP among HCPs, patients, and their families/caregivers, and identify components of an intervention that can improve the knowledge, skills, and confidence of HCPs.

## Method

The review was conducted using the JBI guidelines for Systematic Reviews of Measurement Properties framework,^9^ which provides one of the few approaches to evaluating the measurement properties of surveys. Although its origins stem from patient-reported outcome measures (PROMS), the principles and techniques can be applied to other types of surveys and have been used to evaluate surveys that capture HCP knowledge and competence.^10^ Therefore, it was considered an optimal design to facilitate a robust exploration of the properties of surveys designed to assess CRP knowledge and attitudes. The review’s reporting adhered to the Preferred Reporting Items for Systematic Reviews and Meta-Analyses (PRISMA).^11^ A protocol was developed and registered with PROSPERO (CRD42022286646).

### Search strategy

An initial scope of the literature identified appropriate MeSH terms that were further refined against the PICO framework (Supplemental 1). The final terms were entered into the individual databases and combined using Boolean operators ‘AND’ and ‘OR’. An academic librarian supported completing the search and identifying appropriate MeSH terms (Supplemental 1). The final search was conducted in accordance with the JBI Manual for Evidence Synthesis^9^ and completed in MEDLINE, EMBASE, CINAHL and PsycINFO to ensure that studies captured a wide range of professional groups. The search was limited by year (initially 2011–2021 and updated to include up to June 2024). The decision was made to narrow the search based on JBI guidelines, suggesting older publications are less likely to represent contemporary clinical practice.^9^ Given the advancements in cancer treatments, such as immunotherapy and advanced surgical techniques, we now see people living longer with the consequences of cancer and its treatment, with cancer-related pain being an ever-present consequence.^3^

Papers were restricted to those published in English Language. Identified studies were uploaded into Covidence, enabling two researchers (MG and EB) to independently screen titles and abstracts against the inclusion/exclusion criteria (supplemental 2). Following initial screening, full-text review was also undertaken independently by both researchers. Any disagreements were highlighted within the Covidence systems and consensus was agreed. A third researcher (RV) was available to resolve any disputes; however, this was not required.

## Assessment of methodological quality

The COSMIN Risk of Bias Checklist^12^ was used to assess the methodological quality of included studies.^9^ This is a PROMS based checklist where the principles can be applied to other surveys to assess methodological quality.^13^ The COSMIN Risk of Bias checklist includes ten domains to assess quality:

### Content validity

1) Measurement development, 2) Content validity

### Internal structure

3) Structural validity, 4) Internal consistency, 5) Cross-cultural validity or measurement invariance

### Remaining psychometric properties

6) Reliability 7) Measurement error, 8) Criterion validity, 9) Hypotheses testing for construct validity and 10) Responsiveness.

As per COSMIN guidance,^12^ each identified survey was assessed against the relevant sections of the risk of bias checklist and given a rating of ‘very good’, ’adequate’, ‘doubtful’ or ‘inadequate’. The assessment of the surveys in this manner allows the generation of a summary table outlining the individual merits of each survey.^9^ The COSMIN risk of bias checklist was completed independently by two researchers (MG & EB) and consensus reached for each of the studies included.

## Data extraction/synthesis

Data was extracted from the screened studies using a table of evidence (Table 1), which was constructed according to the JBI Manual for Evidence Synthesis.^9^ The data extracted from the studies included study information, survey used, construct assessed, mode of administration, setting, participants, and results.

**Table 1.** Data extraction.

Study 1. Author 2. Date 3. Location	Instrument	Construct assessed	Mode of administration 1. Collection method 2. Distribution	Setting/Context	Participants	Results 1. Response rate 2. Mean knowledge & attitudes scores 3. Knowledge gaps
1. Kaki^14^	Weissman & Dah (1990)	Knowledge	1. Paper	Medical students working in a single site hospital over a period of 2 years	600 final year medical students	1. 55% (*n* = 325)
2. 2011		Beliefs	2. Postal			2. Not reported
3. Saudi Arabia		Attitudes				3. Cancer pain is untreatable, pain assessment practice, management techniques, addiction.
1. Kim et al.^15^	Self-designed	Knowledge, attitudes, educational experience	1. Not reported	Attending a gathering day, first day as public health doctors	1204 public health doctors	1. 100% (*n* = 1204)
2. 2011			2. Not reported			2. 7.35/16 knowledge scale
3. South Korea						3. Equianalgesic doses, pain assessment scales, did not agree with using an adjuvant for analgesic effect
1. Cheng & Yu^16^	Self-designed	Attitudes	1. Paper	Medical students from single medical school in China.	300 medical students across a range of specialities. In year 6 & 7	1. 97.3% (*n* = 292)
2. 2012		Educational demands	2. In-person			2. Not reported
3. China						3. Pain assessment practice, pharmacology knowledge and addiction.
1. Shahnazi et al.^17^	Self-designed	Knowledge	1. Paper	Educational hospital in Iran	Random selection of 98 nurse	1. 100% (*n* = 98)
2. 2012		Attitudes	2. No reported			2. Knowledge 61.2/100; attitudes 63/100; health beliefs various.
3. Iran		Health beliefs constructs.				3. Opioids and addiction
1. Gustafsson & Borglin^18^	Knowledge and attitudes survey regarding pain (KASRP)	Knowledge	1. Not reported	Two surgical wards (one control and one intervention) at a single hospital site in southern Sweden.	60 registered nursing working on two surgical wards.	1. 60% (*n* = 40)
2. 2013		Attitudes	2. No reported			2. Knowledge & attitudes baseline 67.6%, 4 weeks 72.6%, 12 weeks 69.5%
3. Sweden						3. Pain assessment, pharmacology, adverse effects
1. Piano et al.^19^	Vignette with 5 stages of disease	Knowledge	1. Electronic	Members of national pain society	912 physicians with advanced training in pain.	1. 24% (*n* = 214)
2. 2013			2. Email			2. Not reported
3. France						3. Symptoms related to pain, for example, insomnia, depression, half followed guidelines on neuropathic pain management
1. Jho et al.^20^	Self-designed	Knowledge, practices, and perceived barriers	1. Paper	11 hospitals (6 public and 5 private) across Korea	Doctors and nurses involved in the care of cancer patients across 11 hospital sites. Sample size not stated.	1. 333 (*n* = 149 physicians, 284 nurses)
2. 2014			2. In person			2. Knowledge scores nurse 9.0/14; physician 10.8/14
3. Korea						3. Pharmacology, pain assessment, interventional treatments
1. Kim et al.^21^	Self-designed	Knowledge and practice	1. Not reported	Nationwide across Korea	180 physicians	1. Not reported
2. 2014			2. Not reported			2. Knowledge score 14.6/20
3. Korea						3. Equianalgesic dosing for opioid rotation, adverse effects of opioids, management of opioid naive patient.
1. Breuer et al.^22^	Self-designed vignettes	Management responses to cancer pain scenarios	1. Paper	Oncologist, pain medicine and palliative medicine specialists registered with the American medical associations from across all 50 states were included in the study.	1116 oncologist, pain medicine and palliative medicine specialists	1. 49% (*n* = 550)
2. 2015			2. Postal			2. Not reported
3. USA						3. Treatment options and opioid conversations.
1. Hashemi et al.^23^	Self-designed	Knowledge, attitudes, and practice	1. Not reported	Residents from the school of medicine.	69 third year residents from a school of medicine	1. 100% (*n* = 69)
2. 2015			2. Not reported			2. Attitudes 35.8/56; knowledge 25.1/38; practice 11.2/19
3. Iran						3. Interventional techniques, pain assessment, pharmacology, neuropathic pain, addiction, physiology of cancer pain.
1. Shahriary et al.^24^	KASRP	Knowledge and attitudes	1. Not reported	Oncology, Shahid Sadoughi hospital	62 oncology nurses	1. 93.5% (*n* = 58)
2. 2015			2. Not reported			2. Knowledge & attitudes score 28.5/39
3. Iran						3. General low levels. Patient’s pain scores and perceived contrasts in behaviour, fear of addiction
1. Beck et al.^25^	KASRP	Knowledge	1. Paper	6 oncology units from 3 hospital sites (two academic medical centres and one community-based hospital)	91 registered nurses who worked at least 60% of their time in oncology units	1. Not reported
2. 2016		Attitudes	2. Postal			2. Knowledge & attitudes score 82% (OCN certified); 76% (non-certified); 74% (ineligible for certification)
3. USA						3. Addiction
1. Ferreira et al.^26^	Ramous (1994)	Knowledge	1. Paper	Resident nurses enrolled in a multi-professional oncology residency program within a high complexity oncology centre in Brazil.	29 nurses in first and second year of the residency program.	1. 22/29
2. 2016			2. In person			2. Not reported
3. Brazil						3. Not reported
1. Kasasbeh et al.^27^	KASRP	Knowledge	1. Not reported	Two medical, two surgical wards at a single site.	170 doctors, nurse managers and registered nurses. Pharmacists also invited but none in final group.	1. Pre 52.9% (*n* = 90), post 43.5% (*n* = 74)
2. 2016		Attitudes	2. Not reported			2. Knowledge & attitudes score pre-intervention −22.37, post intervention 26.01 (*p* < 0.001)
3. Ireland		Practices				3. Not reported
1. Tufail et al.^28^	KASRP	Nursing knowledge and attitudes regarding pain management of cancer patients.	1. Not reported	Oncology units across 4 hospitals of Lahore	100 nursing working in selected oncology units. Those with post graduate qualifications and in leadership roles were excluded.	1. 100%
2. 2017			2. Not reported			2. Knowledge 59%; attitudes 60%
3. Pakistan						3. Not reported
1. García-Mata et al.^29^	Self-designed	Oncologist perspectives on cancer pain management	1. Paper	Medical oncologist from across Spain.	Medical oncologist registered by the Spanish association of medical Oncology. Total sample not stated.	1. Wave one: 73; wave two: 82 (no sample population given)
2. 2018			2. Not reported			2. Not reported
3. Spain						3. Pharmacology management of cancer pain; pain assessment
1. Utne et al.^30^	KASRP	Knowledge	1. Electronic	Cancer forum members	312 nurses	1. 18.3% (*n* = 57)
2. 2018		Attitudes	2. Online survey			2. Knowledge & attitudes score 31/41
3. Norway						3. Medication for breakthrough pain, what they say they do and decisions made, fear of opioid dependence
1. Al-Atiyyat et al.^31^	KASRP	Knowledge and attitudes of nurses towards cancer pain management (English version)	1. Paper	Oncology units in two hospital sites in UAE (Dubai and Al ain)	124 nurses with 1–2 years experience, who can speak English and completed a nursing degree programme.	1. 93% (*n* = 115)
2. 2019			2. Postal			2. Knowledge & attitudes score 45%
3. UAE						3. Opioid use, fear of addiction and pain assessment
1. Alnajar et al.^32^	KASRP	Nurses knowledge towards cancer pain management	1. Paper	4 oncology units in Jordan	152 registered nurses working in oncology units who can read/write English, with at least 3 months experience in oncology.	1. 91.4% (*n* = 135)
2. 2019			2. In person			2. Knowledge & attitudes score 51.1%
3. Jordan						3. Pain assessment, addiction, pharmacology
1. Cowperthwaite et al.^33^	KASRP	Nursing knowledge and attitudes regarding pain scores pre and post intervention	1. Electronic	Single site medical oncology unit	11 (preintervention) & 9 (postintervention) nurses working on medical oncology unit, no detail regarding inclusion/exclusion criteria.	1. Pre intervention 31% (*n* = 11); post intervention 25% (*n* = 9)
2. 2019			2. Email			2. Knowledge & attitudes score pre intervention 30.6; post intervention 30.9
3. USA						3. Not reported
1. Darawad et al.^34^	KASRP	Knowledge and attitudes towards cancer pain management	1. Paper	Participants working in oncology units across military, educational, oncology centre and public sectors across Jordan.	88 physicians and 150 nurses working across the oncology units with at least 3 months experience were invited to take part.	1. Physicians 86.2% (n = 75); nurses 91.4% (n = 139)
2. 2019			2. In person			2. Knowledge & attitudes score physicians 62.3%; nurses 51.5%
3. Jordan						3. Pharmacology of opioids, addiction, adverse effects
1. Ferreira et al.^35^	Ramous (1994)	Nurses’ knowledge about cancer pain management	1. Electronic	High complexity oncology reference centre in the city of Rio de Janeiro, Brazil	207 registered nurses working within the high complexity oncology reference centre	1. 126/207
2. 2019			2. Email			2. Not reported
3. Brazil						3. Pain management strategies
1. Toba et al.^36^	Jho (2014)	Knowledge, barriers, practice	1. Not reported	Eight hospitals in West bank/Palestine	220 nurses working in government and private hospitals.	1. 88% (*n* = 194)
2. 2019			2. Not reported			2. Knowledge score 5.1/14
3. Palestine						3. Opioid rescue dose calculation, risk of addiction, respiratory suppression, ceiling effect of opioids, other modalities
1. Admass et al.^37^	KASRP	Nursing knowledge and attitudes of cancer pain management	1. Paper	Oncology centres across Ethiopia	138 nurses working in oncology centres	1. 99.3% (*n* = 138)
2. 2020			2. In person			2. Knowledge & attitudes score 49.84%
3. Ethiopia						3. Pharmacology of pain medications, routes of administration, fear of addiction and disbelieving patients report of pain.
1. El-Aqoul et al.^38^	KASRP	Effects of an education intervention on knowledge of cancer-related pain management	1. Paper	Specialist cancer centre in Jordan	150 registered nurses with >3 months experience working within a cancer centre	1. 85% (*n* = 128)
2. 2020			2. Not reported			2. Knowledge & attitudes score baseline mean score: 23.6; post intervention group: 32.7; post control group: 23
3. Jordan						3. Not reported
1. Yassin et al.^39^	KASRP	Knowledge, attitudes	1. Not reported	Six oncology and haematology units	159 nurses with 3 years oncology experience	1. 81.1% (*n* = 128)
2. 2020			2. Not reported			2. Knowledge & attitudes score 58%
3. Qatar						3. Patients sleeping despite pain, opioid use when pain is unknown, aspirin and NSAIDs for bone metastases, gabapentenoids use, opioids and substance misuse.
1. Yu et al.^40^	KASRP	Knowledge, attitudes	1. Paper	140 hospitals in 20 provinces	505 oncology nurses	1. 97.4% (*n* = 491)
2. 2020			1. Paper			2. Knowledge & attitudes score 56.1%
3. China						3. Opioid dependency, analgesic side effects, acute pain management, pain assessment
1. Hadjisavva et al.^41^	KASRP	Knowledge and attitudes of home care nurses regarding management of cancer pain.	1. Paper	Home care nurses caring for patients with cancer across two not for profit organisations in the capital of Cyprus	31 home care nurses.	1. 100% (*n* = 31)
2. 2021			2. Not reported			2. Knowledge & attitudes score 51.14%
3. Cyprus						3. Use of opioids, pharmacology of action, addiction, pain assessment and non-pharmacology techniques.
1. Kwok et al.^42^	Self-designed	Knowledge, attitudes and perceived practice of breakthrough cancer pain	1. Paper	Six wards in medical geriatric unit from a single regional hospital.	108 nurses (50 control, 58 intervention)	1. 97% (*n* = 104)
2. 2021			2. Postal			2. Knowledge score intervention 53%(pre) 82% (post): Control 51% & 55%
3. Hong Kong						3. Not reported
1. Li et al.^43^	KARSP	Knowledge and attitudes	1. Paper	26 hospitals from across China	982 oncology nurses	1. 97.61% (*n* = 959)
2. 2021			2. Postal			2. Knowledge & attitudes score 21.56
3. China						3. Dose titration, side effects, analgesic use and administration route
1. Su et al.^44^	Self-designed	Knowledge, practice, barriers	1. Electronic	Seven provincial cancer centres in China	411; 186 physicians, 225 nurses	1. 82.2%(*n* = 338)
2. 2021			2. Online survey d			2. Not reported
3. China						3. Assessment, opioid prescription
1. Omer & Nematala^45^	KARSP	Knowledge, attitudes, practice and effectiveness of educational intervention	1. Not reported	Two teaching hospitals	64 nurses (32 intervention, 32 control)	1. 100% (*n* = 64)
2. 2022			2. Not reported			2. Knowledge & attitudes score intervention 9.78 (pre) 17.00 (post): Control pre (not reported) & 10.90 (post)
3. Iraq						3. Not reported
1. Othman & Al-Atiyyat^46^	Jho (2014)	Knowledge, attitudes, barriers, perceived practice	1. Electronic	Three primary health sectors	502 nurses	1. 99.8% (*n* = 501)
2. 2022			2. Online survey			2. Knowledge 6.48
3. Jordan						3. Opioid rescue dose and ceiling effect, risk of addiction, opioid induced respiratory depression, non-steroidal anti-inflammatories
1. Xie et al.^47^	Self-designed	Knowledge, attitudes and perceived practice	1. Electronic	Hospitals in major cities	515; 221 nurses, 178 pharmacists, 116 physicians	1. 100% (*n* = 515)
2. 2022			2. Online survey			2. Knowledge 63.6 (nurses 61.22, pharmacists 63.4. Physicians 67.3).
3. China						3. Not reported
1. Yu et al.^48^	Self-designed	Knowledge, practice and barriers	1. Not reported	Oncology unit staff from 22 provinces	1262; 486 nurses, 317 pharmacists, 459 physicians	1. 94.2% (*n* = 1189)
2. 2022			2. Not reported			2. Knowledge scores 45.1% nurses, 72.2% pharmacists, 75.6% physicians had good knowledge.
3. China						3. Dose titration, side effects, opioid dose calculations
1. Zaabi et al.^49^	KARSP	Knowledge, attitudes	1. Not reported	Hospital, national cancer centre	150 nurses	1. 78% (*n*-118)
2. 2023			2. Not reported			2. Knowledge scores mean 49.6%
3. Oman						3. Dose titration, reliance on vital signs, opioid dependency, withholding opioids for diagnosis
1. Shimizu et al.^50^	End of life nursing education Consortium-JapanCore Quiz – Pain domain only	Knowledge, practice	1. Paper	Hospital and home care environments	309 nurses	1. 44.7% (*n* = 128)
2. 2024			2. Postal via nurse administrators			2. Knowledge accuracy 49.1%
3. Japan’s remote islands (*n* = 32)						3. Psychological dependency, selection of opioids, neuropathic pain, ceiling effect of doses

Data on each survey’s feasibility was extracted and mapped against the core feasibility characteristics recommended by the JBI Manual.^9^ These included the number and type of questions, completion instructions, time to complete and score the measure, information on literacy level and language options, ease of administration, cost of the measure, and availability of the survey for use.

The content of the surveys was also examined to understand the breadth of questions regarding CRP. Given the absence of a standard curriculum regarding CRP knowledge, surveys were mapped against the current European Society for Oncology Medicine Cancer Pain Management Guidelines.^51^ Thus, data across the following areas were extracted:

(1) Physiology of CRP
(2) Pain Assessment
(3) Pharmacological management
(4) Non-pharmacological management
(5) Breakthrough CRP
(6) Cancer-related bone pain
(7) Cancer-related neuropathic pain
(8) Interventional management techniques.

When a full-text version of the survey was not provided in the text, the primary authors of all the studies were contacted to request one. In the event of non-response, the survey content was extrapolated from the questions presented in the studies. Following the JBI Manual for Evidence Synthesis,^9^ a descriptive narrative review approach was employed to synthesise the data.

## Results

**Figure 1.**
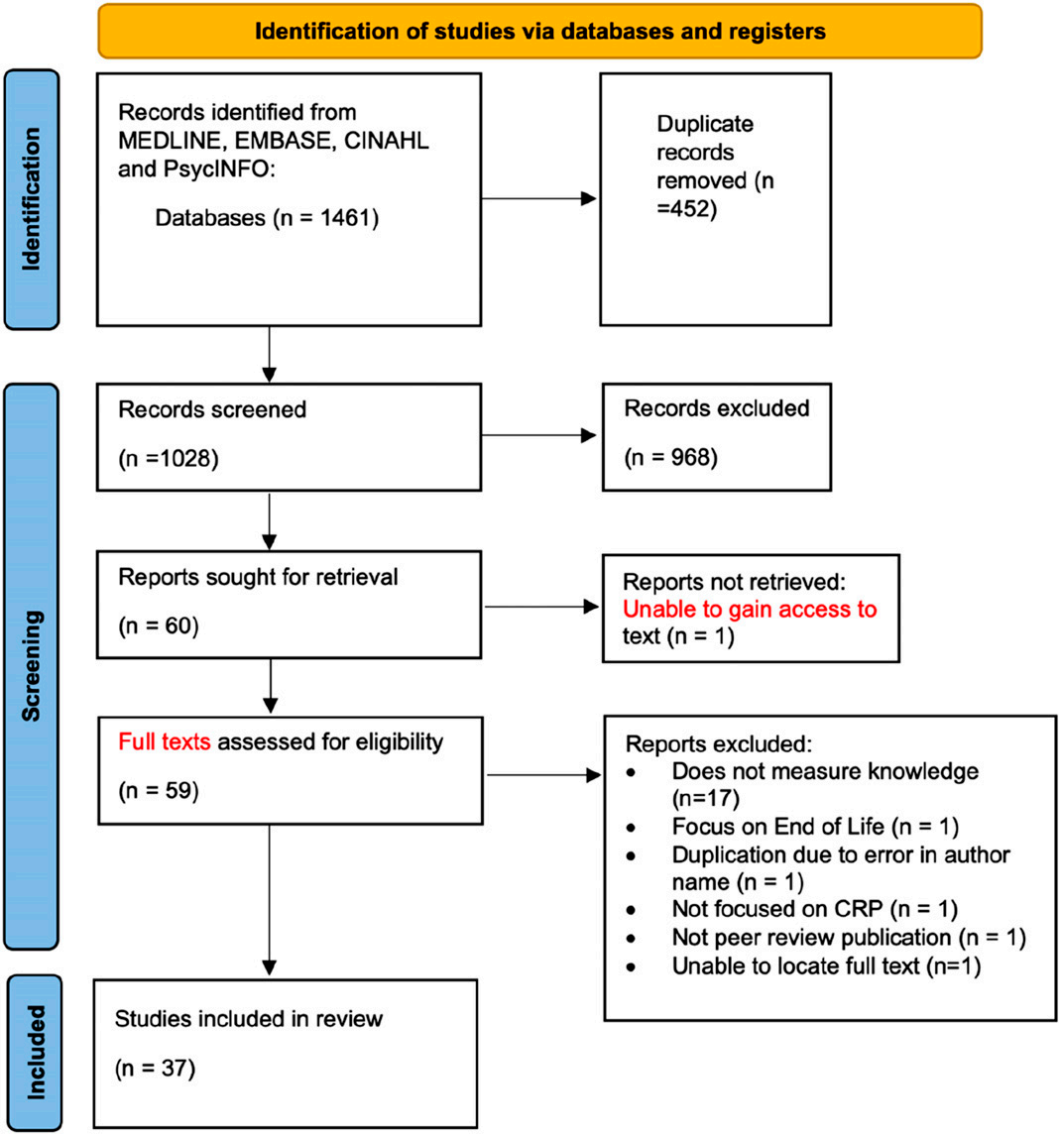
Prisma.

A total of 1024 papers were identified following the initial search. Following the screening and full-text review, 37 studies were included in the final review (see Figure 1). Of the 37 studies included (Table 1), 14 were conducted in the Middle East,^14,17,23,24,28,31,32,34,36,38,39,45,46,49^ 11 in Asia,^15,16,20,21,40,42,43,44,47,48,50^ 6 across Europe,^18,19,27,29,30,41^ 3 across the United States of America,^22,25,33^ 2 in Brazil^26,35^ and 1 in Ethiopia.^37^

### Setting and participants

Across the studies, surveys were administered in various formats, including postal,^14,22,25,31,42,43,50^ face-to-face,^16,26,32,34,37,40^ and electronic.^19,30,35,44,46,47^ The remaining studies provided no details on administration methods (Table 1). Twenty-nine studies assessed nurses’ knowledge,^17,18,20,24–28,30–50^ fourteen assessed physicians’ knowledge,^14–16,19–23,27,29,34,44,47,48^ and three assessed pharmacists’ knowledge.^27,47,48^ No studies have assessed the knowledge of CRP among allied health professionals.

### Surveys used

Within the studies, sixteen distinct surveys were employed. Eighteen studies utilised the ‘Knowledge and Attitudes Survey Regarding Pain’ (KASRP)^8^ (Supplemental 3). The KASRP comprises 39 items. However, thirteen studies exhibited a variation in the number of questions included, with only two studies^33,40^ detailing the modifications made to the original survey. No studies outlined any additional psychometric testing or piloting of their versions of the KASRP. Two studies^26,35^ employed the 24-item ‘Nurses’ Knowledge About the Management of Cancer Pain – WHO’ survey.^52^ Three studies^20,36,46^ utilised the 14-item survey developed by Jho,^20^ while two studies^14,42^ adopted the 18-item survey developed by Weissman & Dahl (1990).^53^ One study employed the pain domain of the End-of-Life Nursing Education Consortium Japan Core Quiz.^50^ The remaining eleven studies (Table 1) developed their own surveys specifically for their study. All surveys incorporated a variety of question types, including multiple-choice, Likert scale, true/false responses, and vignettes.

Across the surveys, scoring systems were heterogeneous, and descriptions of what constitutes a good level of knowledge varied widely; this ranged from 60% to 80% of items answered correctly.

### Cancer content

Only 11 studies provided a full-text version of the survey used or cited the original publication of the survey. The authors of the remaining surveys, specifically designed for those studies, were contacted for copies, but none responded. Consequently, the content was extrapolated where possible for these surveys using questions presented in the articles.

Table 2 shows the content for each of the surveys mapped across domains of the European Society for Oncology Medicine Cancer Pain Management Guidelines.^51^ In the absence of a published curriculum, this was used as a benchmark. No survey covered all aspects when mapped across these domains. However, one study contained 6/8 domains,^23^ one contained 5/8 domains,^22^ three contained 4/8,^15,19,20^ two contained 3/8 domains,^42,44^ with the remaining surveys containing 2/8 domains.

**Table 2.** Cancer content within measurement tools.

Tool/Author	Physiology of cancer-related pain	Pain assessment	Pharmacological management	Non-pharmacological management	Breakthrough cancer-related pain	Cancer-related bone pain	Cancer-related neuropathic pain	Interventional management techniques
Knowledge and attitudes survey regarding pain^8^	No	Yes	Yes	No	No	No	No	No
Nurses’ knowledge about the management of cancer pain – WHO^52^	No	Yes	Yes	No	No	No	No	No
Breuer et al. (2015)^22^	No	No	Yes	No	Yes	Yes	Yes	Yes
Cheng & Yu (2011)^16^	No	Yes	Yes	No	No	No	No	No
Garcia-Mata et al. (2018)^29^	No	Yes	Yes	No	No	No	No	No
Hashemi et al. (2015)^23^	Yes	Yes	Yes	Yes	No	No	Yes	Yes
Jho (2014)^20^ (also used in Toba 2019, Othman 2022)	No	Yes	Yes	No	No	Yes	No	Yes
Weissman & Dahl (1990)^53^ used in Kaki (2011)^14^	No	Yes	Yes	No	No	No	No	No
Kim (2014)^21^	No	No	Yes	No	No	No	Yes	No
Kim (2011)^15^	Yes	Yes	Yes	No	No	No	Yes	No
Kwok (2021)^42^	No	Yes	Yes	No	Yes	No	No	No
Piano (2013)^19^	Yes	No	Yes	Yes	No	No	No	Yes
Su (2021)^44^	Yes	Yes	Yes	No	No	No	No	No
Xie (2022)^47^	Unknown	Unknown	Unknown	Unknown	Unknown	Unknown	Unknown	Unknown
Yu (2022)^48^	No	Yes	Yes	No	No	No	No	No
Arahata et al. (2018)^54^ used in Shimizu et al. (2024)^0^	No	No	Yes	No	No	No	Yes	No

Fifteen surveys contained questions regarding the pharmacological management of CRP,^8,15,16,19,20,21,22,23,29,42,44,48,50,52,53^ and eleven of the surveys also contained questions regarding pain assessment,^8,15,16,20,23,29,42,44,48,52,53^ while four did not.^19,21,22,50^

The remaining aspects of CRP were largely absent from the surveys, with only two including items regarding non-pharmacological management,^19,23^ cancer-related bone pain,^20,22^ and breakthrough cancer pain.^22,42^

### Quality of surveys used

The COSMIN Risk of Bias Checklist^12^ was used to assess the psychometric properties and quality of the surveys. Table 3 outlines the results of this process and rates each stage of their development and implementation. The COSMIN checklist identified weaknesses across all the surveys (Table 3).

**Table 3.** COSMIN quality assessment.

Instrument	Outcomes									
	Content validity		Internal structure			Remaining measurement properties				
	Instrument development	Content validity	Structural validity^a^	Internal consistency	Cross-cultural validity/measurement invariance^b^	Reliability	Measurement error	Criterion validity	Hypotheses testing for construct validity	Responsiveness
Knowledge and attitudes survey regarding pain^8^	**I**	**I**	**N**	**I**	**N**	**D**	**I**	**N**	**N**	**D**
Nurses’ knowledge about the management of cancer pain – WHO^52^	**I**	**I**	**N**	**V**	**N**	**D**	**I**	I	I	**D**
Jho (2014)^a^ ^20^	**D**	**I**	**N**	**I**	**N**	**I**	**I**	**I**	**D**	**D**
Breuer et al. (2015)^22^	**D**	**I**	**N**	**I**	**N**	**I**	**I**	**I**	**I**	**I**
Cheng & Yu (2011)^a^ ^16^	**I**	**I**	**N**	**I**	**N**	**I**	**I**	**I**	**I**	**I**
Garcia-Mata et al. (2018)^a^ ^29^	**I**	**I**	**N**	**I**	**N**	**I**	**I**	**I**	**I**	**I**
Hashemi (2015)^a^ ^23^	**I**	**I**	**N**	**I**	**N**	**I**	**I**	**I**	**I**	**I**
Kim et al. (2011)^15^	**D**	**I**	**N**	**D**	**N**	**I**	**I**	**I**	**I**	**D**
Kim et al. (2014)^21^	**D**	**I**	**N**	**I**	**N**	**I**	**I**	**I**	**I**	**D**
Kwok (2021)^42^	**D**	**I**	**N**	**I**	**N**	**I**	**I**	**I**	**D**	**D**
Piano (2013)^19^	**D**	**I**	**N**	**I**	**N**	**I**	**I**	**I**	**D**	**D**
Su (2021)^44^	**D**	**I**	**N**	**I**	**N**	**I**	**I**	**I**	**D**	**D**
Weissman & Dahl (1990)^53^	**D**	**I**	**N**	**I**	**N**	**I**	**I**	**I**	**D**	**I**
Xie (2022)^47^	**I**	**I**	**N**	**I**	**N**	**I**	**I**	**I**	**I**	**I**
Yu (2022)^48^	**I**	**I**	**N**	**I**	**N**	**I**	**I**	**I**	**I**	**I**
Arahata et al. (2018)^54^ used in Shimizu et al. (2024)^50^	**I**	**I**	**N**	**V**	**N**	**D**	**I**	**I**	**I**	**I**

Rating: V = very good; A = adequate; D = doubtful; I = inadequate; N = not applicable.

^a^Not applicable as items relate to a number of constructs and not measuring a one underlying construct.

^b^No studies examined the cross-cultural validity of measures in cancer pain.

### Content validity (instrument development and content development)

All the surveys were rated as doubtful or inadequate (Table 3) under instrument development since they provided limited, or no description of the process involved in identifying and testing constructs during the development phase. When research teams developed their surveys, they typically authored these with limited or no pilot testing within target samples. All 16 surveys were rated inadequate for content validity because there was little evidence of subject experts assessing relevance and comprehensiveness as part of the formal testing process. This was usually limited to an expert panel to determine content validity (including face validity). However, the nature of the panel or review process is often unclear in the published reports.

### Internal structure (structural validity, internal consistency, cross-cultural validation)

From the three sub-sections of the COSMIN Risk of Bias checklist, internal consistency is the most relevant property to evaluate as structural validity applies to unidimensional scales and cross-cultural validation.

Three studies reported an assessment using Cronbach’s alpha and internal consistency.^15,26,35^ Kim et al.^15^ survey (unnamed) reported Cronbach’s alpha in the sub-scales of attitudes (0.798) and knowledge (0.788). Cronbach’s alpha was used in the remaining two studies,^26,35^ which used the survey developed by Ramos.^52^ Testing was completed for the overall survey and individual sections; ≥0.70 was considered a good level of reliability by the authors. Scores for the whole survey were 0.71^26^ and 0.76,^35^ with acceptable scores in the sub-sections, pain assessment 0.64^26^ & 0.57^35^ and continuous care 0.66^26^ & 0.74.^35^ The control strategies domain was considered insufficient (0.31^26^ & 0.51^35^). Shumizu^50^ used the pain management domain from the End-of-Life Nursing Education Consortium Japan Core Quiz^54^ but did not cite the psychometric testing. Further exploration of the End-of-Life Nursing Education Consortium Japan Core Quiz^54^ identified a Cronbach’s alpha score of 0.76 for the pain management domain.

The original Knowledge and Attitudes Survey Regarding Pain^8^ reported an internal consistency coefficient of >0.70 in 2014 in the knowledge and attitude domains. However, none of the 17 studies in this review reported assessing KASRP for internal consistency in the context of their CRP research despite altering the nature and number of questions.

### Remaining psychometric properties (reliability, measurement error, criterion validity, hypothesis testing for construct validity and responsiveness)

In addition to internal consistency, test-retest is a common technique for exploring a survey’s reliability and risk of measurement error. However, only three studies reported repeated measures testing,^8,26,54^ although the conditions under which this occurred are not clearly described. Therefore, these surveys receive a doubtful rating. The risk of measurement error was not addressed in the studies included in this review, so they were rated inadequate as per COSMIN risk of bias checklist.

Criterion validity refers to the extent to which the score reflects a gold-standard measurement.^12^ There is no agreed-upon gold-standard measurement scale for CRP knowledge against which to compare newly developed surveys.

The studies included in this review did not describe hypothesis testing for construct validity, where internal relationships between scores are explored or compared with similar surveys. A survey’s responsiveness is its ability to detect changes over time, and it can take a criterion – or construct-based approach. As a measurement property, the responsiveness of surveys was not adequately described in the studies included in this review.

### Feasibility of surveys

In terms of length, this varied significantly, with total question numbers ranging from the shortest at four items^22^ to the longest at 77 items.^29^ However, only two surveys^22,42^ stated the time required to complete, and none commented on the feasibility of collating and reviewing responses. None of the surveys mentioned literacy levels or readability of the surveys used, and only eight surveys^8,14,19,20,21,44,48,50^ were freely available within the publication. Ease of administration and cost implications were not explored in any of the presented studies (supplemental 5).

## Discussion

The narrative in previous literature reviews indicates that HCPs have poor knowledge regarding CRP.^6,7^ After examining the content, quality, validity, reliability, and feasibility of the surveys in this systematic review, it is worthwhile to consider how confident we can be in this observation. There are several limitations to existing surveys regarding their scope, content, and methodological robustness, which have impacted the validity and reliability of findings to date. The design and development of the surveys identified in this systematic review were inconsistent and did not address all aspects of the COSMIN risk of bias checklist. However, it is important to note that the COSMIN risk of bias checklist was developed in 2006, predating some of the surveys identified in this review. The guidelines for survey design continue to be debated across the broader evidence base, with many existing reporting guidelines available on the EQUATOR network related to survey development having limitations, with none being superior to the others.^55^ Considering this, it is impossible to recommend a single survey that could adequately be described as a valid and reliable measure of HCPs’ knowledge and attitudes toward CRP.

Our attention should focus on the variability of surveys in the literature, which hinders our understanding of the true extent of HCPs’ knowledge of CRP. This review also clarifies that existing research has primarily focused on nursing and medicine, highlighting the need to explore allied health professionals’ knowledge. All HCPs must be included in future studies to reflect the interdisciplinary approach needed to improve the quality of life of those impacted by CRP.^51^

### Appropriateness of content

The surveys identified do not recognise the complexity of CRP; no survey covered all aspects of CRP. The KASRP^8^ survey was the most widely used across the studies. This is also consistent with studies exploring HCP’s knowledge of non-cancer pain.^56^ While the extensive use of the KASRP^8^ throughout the literature provides reassurance in its ability to measure HCP knowledge of pain, it should be used with caution in cancer settings, as it does not fully capture the complexity of CRP. It does not include key areas of CRP such as bone pain, breakthrough cancer pain, and persistent pain resulting from cancer treatments. A dedicated survey that reflects the complexity of CRP is necessary to assess knowledge of HCPs.

Some surveys are outdated and unlikely to represent contemporary practices given the advancements in cancer treatment over the last decade. For example, Kaki^14^ used the survey by Weissman & Dahl,^53^ which was developed almost 20 years prior and thus unlikely to reflect practices at the time of their study. Similarly, Ferreira et al.^26^ and Ferreira et al.^35^ used a survey developed in 1994.^52^ Future development of these surveys must take into consideration not only the wider impact of CRP but also ensure that they reflect contemporary CRP management practices.

### Psychometric robustness

There was evidence of a lack of rigour in testing all surveys prior to their use. Evaluating measurement properties is an essential step in developing any research instrument, as it reassures the researcher that the survey is reliable and valid, ensuring that the items measure the desired factor and that the results are consistent.^57^ Using the COSMIN Risk of Bias checklist^12^ facilitated a thorough evaluation of the surveys’ psychometric properties, highlighting errors across all aspects of psychometric testing. However, a caveat to the low ratings from the COSMIN Risk of Bias checklist is that the overall score for each section must reflect the lowest rating in each domain. While helpful in measuring overall quality and rigour, it may not always capture the positive aspects of the surveys.

The KASRP^8^ is one of the few surveys with a freely available full-text version that includes guidance notes. While information on validity and reliability is provided, there are no citations or published studies detailing the KASRP’s psychometric properties. None of the reviewed studies that used the KASRP^8^ explored its validity and reliability for CRP knowledge or tested it within a specific cancer context. Similarly, while the original version contains 39 questions, several studies employed modified versions. There was limited information on how the surveys were modified, whether any psychometric testing was conducted following modification, and what impact this may have had on the results. Much more transparency is needed in future research.

### Knowledge & attitude scores

The studies reviewed commonly employed total knowledge and attitude scores, with a strong emphasis on quantifying these scores into numerical values to define what qualifies as a ‘good’ level of knowledge. However, research teams established their own standards for determining what constitutes good knowledge, leading to a lack of consensus in the field. This absence of agreement presents challenges when attempting to make comparisons across studies.

This is more evident in those who used the KASRP. Despite using the same survey, there was variation in what researchers considered ‘good’ knowledge, with scores ranging between 70–80% (Supplemental 4). Within the KASRP guidance notes,^8^ there is no reference to a specific, overall score that would be considered a ‘good’ level of knowledge. However, in earlier work ‘good’ was considered with a score of 80%.^58^ The basis of this rating is unclear and appears to be the consensus of the authors rather than rigorous testing during the development of the survey.^58^

Another example of this is seen from Al-Khawaldeh et al.,^59^ who identified a score of 75% as ‘good’ knowledge based on personal views; this was then replicated in subsequent studies reported in other systematic reviews.^32,34,38^ This lack of an evidence base for scores and consensus further obscures our understanding of the current state of HCP’s knowledge regarding CRP. It could be argued that there should be less emphasis on quantitative scoring and a greater focus on knowledge gaps to support HCPs in developing their CRP practice.

### Feasibility and accessibility

Exploring feasibility characteristics provides insight into the practicalities of administering surveys in practice. While length and completion time are undoubtedly important, only two studies^22,42^ reported the time to complete surveys. Equally, surveys comprising only four items^22^ are likely insufficiently sensitive to examine professionals’ knowledge, while surveys with over 70 items^29^ may be considered onerous to complete.

Researchers utilising existing surveys encounter a lack of clarity concerning the resources needed for administration, the time required for completion, and the time taken to score these surveys. Studies that provide open access to surveys with guidance enhance opportunities to replicate research in various settings, facilitating testing and translation. There is a distinct need to develop a comprehensive survey that is concise, accessible, and reflective of the complexities of CRP, which should be rigorously tested and refined during its development. A crucial precursor to this is achieving an international consensus on the core knowledge domains for HCPs supporting those affected by CRP.

### Language of surveys

This review identified that study locations are spread across the globe, with most studies being performed in Asia and the Middle East (*N* = 24). Despite this, all the studies identified were published in English, and the most used survey was KASRP,^8^ which was developed in English based on clinical practice in the United States of America. Although all the studies in this review were published in English, few indicated whether the original surveys were translated into participants’ first language or if items in the surveys were changed to reflect local practice or cultural differences. Translating versions without appropriate psychometric testing or requiring participants to answer in a second language may impact the validity and reliability. It will also prevent the researchers from identifying the knowledge and attitudes that reflect their local practices.

## Limitations

The review concentrated on publications in English from key databases; consequently, some omissions may have occurred. The included papers often limited in their description of the surveys, and attempts to obtain further information from the authors were unsuccessful. This limitation is evident in some missing or unclear data presented in the tables. It is possible that some crucial data regarding the content or the psychometric evaluation of these surveys is excluded from this review. Two researchers conducted the analysis of the studies; although every effort was made to minimise bias, there remains potential for some to have emerged due to the small size of the review team.

## Conclusion

There has been a consistent narrative suggesting HCPs have poor knowledge of CRP. This systematic review has allowed us to pause and reflect on how HCP knowledge of CRP is being captured and consider existing surveys’ role in contemporary cancer practice. While the surveys used in the past have weaknesses, this is reflective of the broader limitations of survey design research. Therefore, the focus of research moving forward should be on designing a survey that not only reflects contemporary cancer practice but is designed in an inclusive way that brings together all those impacted by the complexity of CRP. Only then can we begin to understand the state of knowledge of HCPs regarding CRP and identify interventions to support the development of HCPs and the wider health and social care workforce.

## Supplemental material

Supplemental material - Cancer pain knowledge and attitudes of healthcare professionals: A systematic review of surveys and their measurement properties Supplemental material for Cancer pain knowledge and attitudes of healthcare professionals: A systematic review of surveys and their measurement properties by Martin Galligan, Rebecca Verity, Theresa Wiseman, Emma Briggs in British Journal of Pain

Supplemental material - Cancer pain knowledge and attitudes of healthcare professionals: A systematic review of surveys and their measurement properties Supplemental material for Cancer pain knowledge and attitudes of healthcare professionals: A systematic review of surveys and their measurement properties by Martin Galligan, Rebecca Verity, Theresa Wiseman, Emma Briggs in British Journal of Pain

Supplemental material - Cancer pain knowledge and attitudes of healthcare professionals: A systematic review of surveys and their measurement properties Supplemental material for Cancer pain knowledge and attitudes of healthcare professionals: A systematic review of surveys and their measurement properties by Martin Galligan, Rebecca Verity, Theresa Wiseman, Emma Briggs in British Journal of Pain

Supplemental material - Cancer pain knowledge and attitudes of healthcare professionals: A systematic review of surveys and their measurement properties Supplemental material for Cancer pain knowledge and attitudes of healthcare professionals: A systematic review of surveys and their measurement properties by Martin Galligan, Rebecca Verity, Theresa Wiseman, Emma Briggs in British Journal of Pain

## Data Availability

All data is included in this manuscript.*
