# Supplementary material for: Cancer pain knowledge and attitudes of healthcare professionals: A systematic review of surveys and their measurement properties
Source: Br J Pain. 2026 Apr 13:20494637261442745. Online ahead of print. doi: 10.1177/20494637261442745 (PMC13076462; doi:10.1177/20494637261442745)
Supplement: Supplemental material - Cancer pain knowledge and attitudes of healthcare professionals: A systematic review of surveys and their measurement properties [file sj-pdf-1-bjp-10.1177_20494637261442745.pdf]

Supplementary Information 1PICO & Example Search Strategy

Table 1 PICO

PICO	Inclusion Criteria	Exclusion Criteria
P – Population / Problem	Healthcare Professionals (Nurse, Doctor, Physician, Allied Health Professional) caring for those with cancer-related pain	Non-healthcare professionals. Those not caring for those with cancer-related pain.
I –Issue	Knowledge and attitudes of cancer-related pain	Exploring topics not related to knowledge and attitudes of cancer-related pain.
C- Construct	Healthcare professionals' knowledge and attitudes of the complexity of assessment and management of cancer-related pain	Not focused on exploring knowledge and attitudes of the complexity of assessment and management of cancer-related pain
O – Outcome	Knowledge, attitudes of Healthcare Professionals	Other domains not focused on knowledge and attitudes.

HDAS Export
Strategy Systematic Review CINAHL

See full search strategy

Strategy 1081122/74

#	Database	Search term	Results
74	PubMed	(("cancer pain").ti,ab OR "CANCER PAIN"/) AND (((("health professional*" OR "health care professional*" OR "healthcare professional*" OR nurs* OR doctor* OR "allied health" OR physician).ti,ab OR exp "HEALTH PERSONNEL"/) AND (knowledge OR attitud* OR barrier*).ti,ab) OR exp "ATTITUDE OF HEALTH PERSONNEL"/)	352

Contents 300 of 352 results on PubMed - (("cancer pain").ti,ab OR "CANCER PAIN"/) AND (((("health professional*" OR "health care professional*" OR "healthcare professional*" OR nurs* OR doctor* OR "allied health" OR physician).ti,ab OR exp "HEALTH PERSONNEL"/) AND (knowledge OR attitud* OR barrier*).ti,ab) OR exp "ATTITUDE OF HEALTH PERSONNEL"/)

1. Pain assessment and registration in medical oncology clinics: operationalised through the lens of health care professionals and patients.

2. Differences in Attitudes and Practices of Cancer Pain Management between Medical Oncologists and Palliative Care Physicians.

3. A Comparative Study of Seminars Combined with Case-Based Learning versus Lecture-Based Learning for Cancer Pain Teaching in Medical Oncology Internship.

4. Practice, Knowledge, and Attitude of Health Care Providers regarding Cancer Pain Management: A National Survey.

5. Effect of Physician-Pharmacist Participation in the Management of Ambulatory Cancer Pain Through a Digital Health Platform: Randomized Controlled Trial.

6. [History and Present of Stereotactic and Functional Neurosurgery].

7. Barriers and facilitators encountered by family physicians prescribing opioids for chronic non-cancer pain: a qualitative study.

8. Effect of a theory-driven educational intervention on the level of knowledge, attitudes, and assessment practices regarding breakthrough cancer pain (BTCP) management among medical nurses in Hong Kong.

9. European clinical practice recommendations on opioids for chronic noncancer pain - Part 2: Special situations.

10. Breaking the Barrier: Challenges of Methadone Use - An Introductory Observation.

11. The opioid-prescribing practices of Australian general practice registrars: an interview study.

12. Current Status of Cancer-Related Pain and Opioid use in South Lebanon: A Pilot Study.

13. Knowledge and attitudes regarding cancer pain management among oncology nurses in China.

14. Modular transitional nursing intervention improves pain-related self-management for cancer patients: Study protocol for a randomized controlled trial.

15. Beyond the black stump: rapid reviews of health research issues affecting regional, rural and remote Australia.

16. Differences in nurses’ perceptions of self-reported pain and the administered morphine dose according to the patient’s facial expression in Korea

17. Exploration of Patient-Related Barriers to Effective Cancer Pain Management in a Diverse Multicultural Developing Country.

18. Effectiveness of advanced nursing care (ANC) on bone cancer pain, psychological disorders and quality of life in patients with primary bone cancers: A protocol for a PRISMA-compliant meta-analysis.

19. Effectiveness of Education Program on Nursing Knowledge and Attitude toward Pain Management.

20. Knowledge and Attitude of Ethiopian Oncology Nurses About Cancer Pain Management: National Survey.

21. Time trends and prescribing patterns of opioid drugs in UK primary care patients with non-cancer pain: A retrospective cohort study.

22. Cancer Pain Treatment and Management: An Interprofessional Learning Module for Prelicensure Health Professional Students.

23. Chronic non-cancer pain management by nurses in specialist pain clinics.

24. Manifestation and parental assessment of children's cancer pain at home: An exploratory mixed-methods study.

25. Breakthrough cancer pain treatment in Spain: physicians' perception of current opioids utilization and prescription.

26. Opioid Induced Hyperalgesia, a Research Phenomenon or a Clinical Reality? Results of a Canadian Survey.

27. Knowledge and Attitudes of Chinese Oncology Nurses Regarding Cancer Pain Management-a Cross-Sectional Study.

28. Morphine use for cancer pain: A strong analgesic used only at the end of life? A qualitative study on attitudes and perceptions of morphine in patients with advanced cancer and their caregivers.

29. Law, Culture, and Fear: A Qualitative Study of Health Professionals' Perceptions of Narcotic Use Related to Cancer Pain.

30. Risk of opioid misuse in people with cancer and pain and related clinical considerations: a qualitative study of the perspectives of Australian general practitioners.

31. Mindfulness-Based stress reduction in early palliative care for people with metastatic cancer: A mixed-method study.

32. Barriers to Cancer Pain Management Among Nurses in Kenya: A Focused Ethnography.

33. Maternal Pain Management at Home in Children with Cancer: A Turkish Sample.

34. Evaluating recruitment methods of patients with advanced cancer: a pragmatic opportunistic comparison.

35. Associated factors with the knowledge of nurses of a high complexity oncology centre in Brazil, on the management of cancer pain.

36. Attitudes of Hospice Providers Regarding Intrathecal Targeted Drug Delivery for Patients With Cancer.

37. Nurses' knowledge, perceived barriers, and practices regarding cancer pain management: a cross-sectional study from Palestine.

38. Managing Pain in People with Cancer-a Systematic Review of the Attitudes and Knowledge of Professionals, Patients, Caregivers and Public.

39. An Overview of Cancer Pain: Epidemiology and Pathophysiology.

40. Genetic Variants Associated with Cancer Pain and Response to Opioid Analgesics: Implications for Precision Pain Management.

41. Understanding the behavioural determinants of opioid prescribing among family physicians: a qualitative study.

42. The Global Burden of Cancer Pain.

43. Physician-pharmacist collaboration on chronic non-cancer pain management during the opioid crisis: A qualitative interview study.

44. Opioids and Cancer Pain Management in the United States: Public Policy and Legal Challenges.

45. Cancer Pain Assessment and Measurement.

46. Telephone Follow-up Design and Practice for Advanced Cancer Pain Patients.

47. Care management for the hospitalized child with chronic cancer pain: intervening conditions.

48. Cancer Pain and Quality of Life.

49. Addressing Educational Needs in Managing Complex Pain in Cancer Populations: Evaluation of APAM: An Online Educational Intervention for Nurses.

50. Therapeutic alternatives for supporting GPs to deprescribe opioids: a cross-sectional survey.

51. Family caregiver beliefs and barriers to effective pain management of cancer patients in home care settings.

52. [An assessment of physicians attitudes toward opioid usage and opiophobia: Results of a survey from a training and research hospital].

53. Attitudinal Barriers to Pain Management and Associated Factors Among Cancer Patients in Mainland China: Implications for Cancer Education.

54. Factors That Hinder and Facilitate Cancer Patients' Knowledge About Pain Management-A Qualitative Study.

55. Testing a pain self-management intervention by exploring reduction of analgesics' side effects in cancer outpatients and the involvement of family caregivers: a study protocol (PEINCA-FAM).

56. A Survey of the Knowledge and Attitudes of Oncology Nurses toward Pain in United Arab Emirates Oncology Settings.

57. Healthcare provider knowledge, attitudes, beliefs, and practices surrounding the prescription of opioids for chronic non-cancer pain in North America: protocol for a mixed-method systematic review.

58. [Elaboration and evaluation of a therapeutic education program in cancer pain management].

59. Cancer Pain Management Among Oncology Nurses: Knowledge, Attitude, Related Factors, and Clinical Recommendations: a Systematic Review.

60. Enhancing Cancer Pain Assessment and Management in Hospice.

61. PROMs for Pain in Adult Cancer Patients: A Systematic Review of Measurement Properties.

62. Parents' Voice in Managing the Pain of Children with Cancer during Palliative Care.

63. Oncology nurse reflection on the necessity of a pain assessment tool for Indonesian cancer patients.

64. Improving the management of pain from advanced cancer in the community: study protocol for a pragmatic multicentre randomised controlled trial.

65. [Challenges for home care services in the pain management of cancer patients : A qualitative study].

66. The Use of Comfort Kits to Optimize Adult Cancer Pain Management.

67. How do patients with cancer pain view community pharmacy services? An interview study.

68. New frontier: cancer pain management clinical fellowship.

69. Experiences of Urban African Americans with Cancer Pain.

70. Communicating Caregivers' Challenges With Cancer Pain Management: An Analysis of Home Hospice Visits.

71. Knowledge and beliefs about chronic non cancer pain management for family medicine group nurses.

72. Cancer pain management needs and perspectives of patients from Chinese backgrounds: a systematic review of the Chinese and English literature.

73. Cancer Pain Management at Oncology Units: Comparing Knowledge, Attitudes and Perceived Barriers Between Physicians and Nurses.

74. Update in Hospital Palliative Care: Symptom Management, Communication, Caregiver Outcomes, and Moral Distress.

75. Cancer-related pain: a nationwide survey of patients' treatment modification and satisfaction in Taiwan.

76. Knowledge and Attitudes Toward Cancer Pain Management Among Nurses at Oncology Units.

77. Developing a Short Form of the German Barriers Questionnaire II: A Validation Study in Four Steps.

78. Cancer pain management in China: current status and practice implications based on the ACHEON survey.

79. Multimodal intrathecal analgesia in refractory cancer pain.

80. Pain and pain management in hospitalized patients before and after an intervention.

81. The social and behavioral influences (SBI) study: study design and rationale for studying the effects of race and activation on cancer pain management.

82. Development and Testing of an Intelligent Pain Management System (IPMS) on Mobile Phones Through a Randomized Trial Among Chinese Cancer Patients: A New Approach in Cancer Pain Management.

83. Co-creative development of an eHealth nursing intervention: Self-management support for outpatients with cancer pain.

84. Testing the Implementation of a Pain Self-management Support Intervention for Oncology Patients in Clinical Practice: A Randomized Controlled Pilot Study (ANtiPain).

85. Cancer-Related Pain Assessment: Monitoring the Effectiveness of Interventions.

86. [Assessment and Strategy for Nociceptive Pain in Cancer].

87. Mapping French people and health professionals' positions regarding the circumstances of morphine use to relieve cancer pain.

88. Can A Complex Online Intervention Improve Cancer Nurses' Pain Screening and Assessment Practices? Results from a Multicenter, Pre-post Test Pilot Study.

89. Prescription Opioid Abuse in Chronic Pain: An Updated Review of Opioid Abuse Predictors and Strategies to Curb Opioid Abuse: Part 1.

90. Barriers to venipuncture-induced pain prevention in cancer patients: a qualitative study.

91. Healthcare Providers' Knowledge and Current Practice of Pain Assessment and Management: How Much Progress Have We Made?

92. Pain and social processes for hospice cancer patients: An integrative review.

93. Cancer Pain Management in Developing Countries.

94. [Patterns of prescription of opioid analgesics in Hôtel-Dieu de France of Beyrouth].

95. Managing Pain in Patients With Cancer: The Chinese Good Pain Management Experience.

96. Practice Patterns in Distinguishing Between Background Pain and Breakthrough Pain During Patient Education: a Korean Physician Survey.

97. Collaborative practice model for management of pain in patients with cancer.

98. Integrated pain and palliative medicine model.

99. Opioid-induced Hallucinations: A Review of the Literature, Pathophysiology, Diagnosis, and Treatment.

100. Nurse Attitude-Related Barriers to Effective Control of Cancer Pain among Iranian Nurses.

101. Cancer Pain Management Insights and Reality in Southeast Asia: Expert Perspectives From Six Countries.

102. Performance and quality indicators for the management of non-cancer chronic pain: a scoping review protocol.

103. Medical use of cannabis products: Lessons to be learned from Israel and Canada.

104. What to Do, and What Not to Do, When Diagnosing and Treating Breakthrough Cancer Pain (BTcP): Expert Opinion.

105. Knowledge and Beliefs about Chronic Non Cancer Pain Management for Family Medicine Group Nurses.

106. Diagnosis and management of breakthrough cancer pain: Have all the questions been resolved? A Delphi-based consensus assessment (DOIRON).

107. Oncology Nursing Certification: Relation to Nurses' Knowledge and Attitudes About Pain, Patient-Reported Pain Care Quality, and Pain Outcomes.

108. Comparison of Oncology Patients' and Their Family Caregivers' Attitudes and Concerns Toward Pain and Pain Management.

109. Oncology Nurses Knowledge and Attitudes Regarding Cancer Pain Management.

110. Interventions for Nurse-Related Barriers in Cancer Pain Management.

111. Australian survey of current practice and guideline use in adult cancer pain assessment and management: The community nurse perspective.

112. A holistic approach to chronic pain management that involves all stakeholders: change is needed.

113. Priority interventions to improve the management of chronic non-cancer pain in primary care: a participatory research of the ACCORD program.

114. Self-management support intervention to control cancer pain in the outpatient setting: a randomized controlled trial study protocol.

115. Current practices in cancer pain management in Asia: a survey of patients and physicians across 10 countries.

116. Evaluating resident physicians' knowledge, attitude, and practice regarding the pain control in cancer patients.

117. Nurse-led educational interventions on cancer pain outcomes for oncology outpatients: a systematic review.

118. A satisfaction survey on cancer pain management using a self-reporting pain assessment tool.

119. Intravenous lidocaine for cancer pain without electrocardiographic monitoring: a retrospective review.

120. Educational gaps among healthcare providers: an institution needs assessment to improve pain management for postsurgical patients.

121. Improving cancer pain control with NCCN guideline-based analgesic administration: a patient-centered outcome.

122. Management of persistent pain in the older patient: a clinical review.

123. Knowledge, practices, and perceived barriers regarding cancer pain management among physicians and nurses in Korea: a nationwide multicenter survey.

124. Use of Opioids and Sedatives at End-of-Life.

125. Breakthrough cancer pain (BTcP): a synthesis of taxonomy, pathogenesis, therapy, and good clinical practice in adult patients in Italy.

126. Healthcare providers' perspectives of the supportive care needs of men with advanced prostate cancer.

127. A nationwide survey of knowledge of and compliance with cancer pain management guidelines by korean physicians.

128. Pain in the cancer patient: different pain characteristics CHANGE pharmacological treatment requirements.

129. Patient education, coaching, and self-management for cancer pain.

130. A survey on doctors' knowledge and attitude of treating chronic pain in three tertiary hospitals in Nigeria.

131. An ethnographic study of barriers to cancer pain management and opioid availability in India.

132. A systematic review: non-pharmacological interventions in treating pain in patients with advanced cancer.

133. Pain medication management processes used by oncology outpatients and family caregivers part I: health systems contexts.

134. "We all talk about it as though we're thinking about the same thing." Healthcare professionals' goals in the management of pain due to advanced cancer: a qualitative study.

135. Improvement of pain-related self-management for cancer patients through a modular transitional nursing intervention: a cluster-randomized multicenter trial.

136. Regional medical professionals' confidence in providing palliative care, associated difficulties and availability of specialized palliative care services in Japan.

137. European Oncology Nursing Society breakthrough cancer pain guidelines.

138. Challenges of conducting experimental studies within a clinical nursing context.

139. GPs prescribing of strong opioid drugs for patients with chronic non-cancer pain: a qualitative study.

140. [Cancer pain management: good clinical practices, use of strong opioids].

141. Can a theory-based educational intervention change nurses' knowledge and attitudes concerning cancer pain management? A quasi-experimental design.

142. [Attitudes of Hungarian adults toward use of opioids in pain management].

143. Barriers to cancer pain management: Jordanian nurses' perspectives.

144. The appropriate treatment of chronic pain.

145. How nurses assess breakthrough cancer pain, and the impact of this pain on patients' daily lives--results of a European survey.

146. The PRO-SELF pain control program improves family caregivers' knowledge of cancer pain management.

147. Interventional pain management in the palliative care patient.

148. Results of a randomized controlled pilot study of a self-management intervention for cancer pain.

149. Cancer pain part 2: assessment and management.

150. Opioid epidemic in the United States.

151. The PRO-SELF(©) Pain Control Program improves patients' knowledge of cancer pain management.

152. The appropriate treatment of chronic pain.

153. Pain in clinical oncology: patient satisfaction with management of cancer pain.

154. [Cancer patients adherence and symptom management: the influence of the patient-physician relationship].

155. The survey of nurse's knowledge and attitude toward cancer pain management: Application of Health Belief Model.

156. A randomized, clinical trial of education or motivational-interviewing-based coaching compared to usual care to improve cancer pain management.

157. [Physicians' knowledge on cancer pain therapy : Comparison of palliative care and prehospital emergency physicians in training].

158. Medical oncologists' attitudes and practice in cancer pain management: a national survey.

159. Attitudes, beliefs, and practices of Sri Lankan nurses toward cancer pain management: an ethnographic study.

160. Primary care providers' perspective on prescribing opioids to older adults with chronic non-cancer pain: a qualitative study.

161. Medical students' knowledge and attitude toward cancer pain management in Saudi Arabia.

162. A pain education programme to improve patient satisfaction with cancer pain management: a randomised control trial.

163. Nursing's role in cancer pain management.

164. Attitude and knowledge of physicians about cancer pain management: young doctors of South Korea in their early career.

165. Meta-analysis of cultural differences in Western and Asian patient-perceived barriers to managing cancer pain.

166. Opioids for cancer pain in the Middle Eastern countries: a physician point of view.

167. Urine drug testing in chronic pain.

168. Using latent transition analysis in nursing research to explore change over time.

169. Cancer pain management in ambulatory care: can we link assessment and action to outcomes?

170. The DSCP-CA: a decision support computer program--cancer pain management.

171. Effect of certification in oncology nursing on nursing-sensitive outcomes.

172. Rational use and effectiveness of morphine in the palliative care of cancer patients at the Ocean Road Cancer Institute in Dar es Salaam, Tanzania.

173. Types and epidemiology of cancer-related neuropathic pain: the intersection of cancer pain and neuropathic pain.

174. Psychological and behavioural predictors of pain management outcomes in patients with cancer.

175. Review of the effect of opioid-related side effects on the undertreatment of moderate to severe chronic non-cancer pain: tapentadol, a step toward a solution?

176. Using leadership and advocacy to improve cancer pain management--based on a presentation at the cancer pain, suffering and spirituality course.

177. Improvement of pain related self management for oncologic patients through a trans institutional modular nursing intervention: protocol of a cluster randomized multicenter trial.

178. A survey on physician knowledge and attitudes towards clinical use of morphine for cancer pain treatment in China.

179. Guilty until proven innocent: a qualitative study of the management of chronic non-cancer pain among patients with a history of substance abuse.

180. [Attitude survey of medical staff on the participation of community pharmacists in palliative home care].

181. Barriers to cancer pain management: a review of empirical research.

182. The Danish Barriers Questionnaire-II: preliminary validation in cancer pain patients.

183. Effect of certification in oncology nursing on nursing-sensitive outcomes.

184. Pediatric palliative care: use of opioids for the management of pain.

185. Nursing pain management--a qualitative interview study of patients with pain, hospitalized for cancer treatment.

186. Cancer-related pain: a pan-European survey of prevalence, treatment, and patient attitudes.

187. Health care providers' assessments of the quality of advanced-cancer care in Latin American medical institutions: a comparison of predictors in five countries: Argentina, Brazil, Cuba, Mexico, and Peru.

188. Doctors' opinions, knowledge and attitudes towards cancer pain management in a university hospital.

189. The Danish version of the Medication Adherence Report Scale: preliminary validation in cancer pain patients.

190. Opioids and cancer survivors: issues in side-effect management.

191. Cancer pain: perspectives of a medical oncologist.

192. Pain centers professionals' beliefs on non-cancer chronic pain.

193. [Variable prescription of opioids to cancer patients in Norway].

194. Concepts within the Chinese culture that influence the cancer pain experience.

195. Prevalence and treatment of cancer pain in Italian oncological wards centres: a cross-sectional survey.

196. [Nursing certification system in cancer nursing].

197. Nurse coaching to explore and modify patient attitudinal barriers interfering with effective cancer pain management.

198. Knowledge and attitudes of Turkish oncology nurses about cancer pain management.

199. Action research: developing a pediatric cancer pain program in jordan.

200. Controlling cancer pain with pharmacotherapy.

201. Patient training in cancer pain management using integrated print and video materials: a multisite randomized controlled trial.

202. [An attitude survey on the medical use of narcotics for cancer pain relief].

203. Audit and feedback as a clinical practice guideline implementation strategy: a model for acute care nurse practitioners.

204. Implementing the Fatigue Guidelines at one NCCN member institution: process and outcomes.

205. Physician-related barriers to cancer pain management with opioid analgesics: a systematic review.

206. Evaluation of "The Many Faces of Pain": a chronic cancer pain management education program.

207. Management of cancer pain with complementary therapies.

208. Educating for tomorrow: enhancing nurses' pain management knowledge.

209. Pain management in hospitalized cancer patients: a systematic review.

210. Knowledge and attitudes about cancer pain management: a national survey of Italian hospice nurses.

211. The pain of residents with terminal cancer in USA nursing homes: family members' perspectives.

212. [Reading nursing Literature in English: new inputs for practicing nurses].

213. Can patient coaching reduce racial/ethnic disparities in cancer pain control? Secondary analysis of a randomized controlled trial.

214. Pain issues from the palliative perspective: a survey among doctors in Hospital Melaka.

215. Perioperative and intraoperative pain and anesthetic care of the chronic pain and cancer pain patient receiving chronic opioid therapy.

216. Clinicians' practice and attitudes toward cancer pain management in Korea.

217. Knowledge and attitudes about cancer pain management: a national survey of Italian oncology nurses.

218. Structural visualization of expert nursing: Cancer pain management.

219. The measurement of pain from metastatic bone disease: capturing the patient's experience.

220. What doctors know about cancer pain management: an exploratory study in Sarawak, Malaysia.

221. [Essentials for transition of palliative care patients to palliative home care and for management of their cancer pain].

222. The prescription of opioid analgesics to terminal cancer patients: impact of physicians' general attitudes and contextual factors.

223. A randomized controlled trial of an educational intervention on Hellenic nursing staff's knowledge and attitudes on cancer pain management.

224. Home care nurses' perceptions of control over cancer pain.

225. Cancer pain in palliative care: why is management so difficult?

226. Alternatives in cancer pain treatment: the application of chiropractic care.

227. Palliative care. Some organisational considerations.

228. Evaluation of education in palliative care: determining the effects on nurses' knowledge and attitudes.

229. Nurses' willingness to maximize opioid analgesia for severe cancer pain, and its predictor.

230. Physicians' knowledge of transdermal fentanyl.

231. A survey of cancer pain management knowledge and attitudes of British Columbian physicians.

232. The PRO-SELF pain control program improves patients' knowledge of cancer pain management.

233. National Institutes of Health State-of-the-Science Conference Statement: Symptom management in cancer: pain, depression, and fatigue, July 15-17, 2002.

234. Improving cancer pain management by homecare nurses.

235. A randomized controlled trial of a nurse-administered educational intervention for improving cancer pain management in ambulatory settings.

236. Managing children's cancer pain in Morocco.

237. Epidemiology of cancer pain and factors influencing poor pain control.

238. Use of strong opioids for non-cancer pain in the community: a case study.

239. Attitudes toward opioid use for chronic pain: a Canadian physician survey.

240. Clinical decision making in pain management: Contributions of physician and patient characteristics to variations in practice.

241. Evaluation of a cancer pain education module.

242. Are nurses prepared to manage cancer pain? A national survey of nurses' knowledge about pain control in Taiwan.

243. My love is hurting: the meaning spouses attribute to their loved ones' pain during palliative care.

244. Knowledge and attitudes about cancer pain management: a comparison of oncology and nononcology nurses.

245. National Institutes of Health State-of-the-Science Conference Statement: Symptom Management in Cancer: Pain, Depression, and Fatigue, July 15-17, 2002.

246. Clinicians communicating with patients experiencing cancer pain.

247. Barriers to cancer pain management: home-health and hospice nurses and patients.

248. Physician variability in the management of acute postoperative and cancer pain: a quantitative analysis of the Michigan experience.

249. Developing a computerized data collection and decision support system for cancer pain management.

250. [A survey of physicians' knowledge about pain therapy with strong opioid analgesics].

251. Multicenter study of pain and its management in patients with advanced cancer in Korea.

252. The Zero Acceptance of Pain (ZAP) Quality Improvement Project: evaluation of pain severity, pain interference, global quality of life, and pain-related costs.

253. Decision support computer program for cancer pain management.

254. The PRO-SELF(c): Pain Control Program--an effective approach for cancer pain management.

255. Patient-related barriers to pain management: the Barriers Questionnaire II (BQ-II).

256. [Indicators of structural quality in palliative care for cancer pain patients in Lower-Saxony].

257. Chronic non-cancer pain in older people: current evidence for prescribing.

258. Ethical issues in pain management.

259. Pediatric nurses' knowledge and attitudes survey regarding pain.

260. Cancer pain management among underserved minority outpatients: perceived needs and barriers to optimal control.

261. Why study pain? A qualitative analysis of medical and nursing faculty and students' knowledge of and attitudes to cancer pain management.

262. The knowledge and attitudes of surgical staff towards the use of opioids in cancer pain management: can the Hospital Palliative Care Team make a difference?

263. Opioid use in chronic pain management in the Philippines.

264. [Factors that enable the patients to live their life till death at home by controlling cancer-pains with continuous subcutaneous injection of opioids-case reports of 3 patients with a terminal cancer].

265. Understanding opioid tolerance in cancer pain.

266. An educational implementation of a cancer pain algorithm for ambulatory care.

267. [Improvement of palliative outpatient treatment of terminally ill cancer patients - SUPPORT as example - The ethically preferable alternative to euthanasia].

268. Establishing a cancer pain clinic in a developing country: effect of a collaborative link project with a UK cancer pain center.

269. Educational interventions to improve cancer pain control: a systematic review.

270. Attitudes and knowledge about cancer pain in Flanders. The educational effect of workshops regarding pain and symptom control.

271. Is hands-on experience more effective than didactic workshops in postgraduate cancer pain education?

272. Feasibility of quantitative pain assessment in outpatient oncology practice.

273. Barriers in cancer pain management.

274. Hospice and hospital oncology unit nurses: a comparative survey of knowledge and attitudes about cancer pain.

275. Physicians' knowledge and attitudes toward the use of analgesics for cancer pain management: a survey of two medical centers in Taiwan.

276. [Palliative care of the terminal head and neck cancer patient].

277. Patients' and nurses' assessment of cancer pain.

278. Minority cancer patients and their providers: pain management attitudes and practice.

279. Persisting misconceptions of Belgian physicians and nurses about cancer pain treatment.

280. Influencing nurses' knowledge, attitudes, and practice in cancer pain management.

281. Concerns and misconceptions about pain among Hong Kong Chinese patients with cancer.

282. Testing a multimedia module in cancer pain management.

283. [Attitudes towards terminal care among the general population and medical practitioners in Japan].

284. Evolution of the French public's knowledge and attitudes regarding postoperative pain, cancer pain, and their treatments: two national surveys over a six-year period.

285. Patient-related barriers to cancer pain management in a palliative care setting in Hong Kong.

286. The treatment of chronic cancer pain in a cancer hospital in The Netherlands.

287. [The pharmaceutical care and pain caused by cancer].

288. Improving cancer pain management using a performance improvement framework.

289. Learning effects of a workshop in palliative cancer care for general practitioners.

290. Cancer pain: knowledge and attitudes of physicians in Israel.

291. Cancer and chronic pain.

292. Barriers to cancer pain relief: fear of tolerance and addiction.

293. Knowledge and attitudes of health-care providers toward cancer pain management: a comparison of physicians, nurses, and pharmacists in the state of New Hampshire.

294. Cancer pain survey: patient-centered issues in control.

295. Regulatory barriers to pain management.

296. An examination of nursing attitudes and pain management practices.

297. [Indications and limits of nerve block techniques].

298. A pain education program for chronic cancer pain patients: follow-up results from a randomized controlled trial.

299. Patients' knowledge of and attitudes toward the management of cancer pain.

300. Attitudes of Italian general practitioners in the treatment of cancer pain. The Committee of the Associazione Italiana di Oncologia Medica (AIOM).

Full strategy

Results 300 of 352 results on PubMed - (("cancer pain").ti,ab OR "CANCER PAIN"/) AND (((("health professional*" OR "health care professional*" OR "healthcare professional*" OR nurs* OR doctor* OR "allied health" OR physician).ti,ab OR exp "HEALTH PERSONNEL"/) AND (knowledge OR attitud* OR barrier*).ti,ab) OR exp "ATTITUDE OF HEALTH PERSONNEL"/)

1. Pain assessment and registration in medical oncology clinics: operationalised through the lens of health care professionals and patients.

Author(s): O'Connor L; Hassett A; Sheridan N

Source: HRB open research; 2021; vol. 4 ; p. 86

Publication Date: 2021

Publication Type(s): Journal Article

DOI: http://dx.doi.org/10.12688/hrbopenres.13367.1

ISSN: 2515-4826

Place of Publication: Ireland

PubMedID: 34514326

Accession Number: 34514326

Keywords: Subject Terms: Pain assessment; cancer pain; documentation of pain; interprofessional expertise; mixed methods; pain-related barriers; self-report of pain

Abstract: Background: Pain is a common symptom in patients who survive cancer and in those who live with progressive advanced disease. Systematic screening and documentation of pain are necessary to improve the quality of cancer pain treatment, because a key pain-related barrier is that patients are reluctant to discuss pain, due to fear that reporting pain will distract the healthcare professional from their cancer treatment. Methods: This study adopted an explanatory sequential mixed-methods design. Data collection incorporated three strands. The first strand involved a quantitative enquiry in which medical chart reviews of patients (n=100) attending the medical oncology outpatient clinic were examined. The second qualitative strand comprised of semi-structured interviews with patients (n=10) attending that service. The third strand was qualitative and consisted of focus group discussions with healthcare professionals (n=12). Results: All 100 patients had cancer. The quantitative findings confirmed the suboptimum assessment and subsequent recording of patient's pain, that seemed to afford a reality check for all healthcare professionals. For patients, the outcomes of the anti-cancer treatment were their priority, and pain was perceived as inevitable, being associated with a cancer diagnosis. There were multifaceted complexities voiced amongst healthcare professionals associated with balancing the benefits and harms aligned with treating cancer pain. Conclusions: Pain assessment in medical records was not systematically recorded by healthcare professionals. Patients were reluctant to self-report pain during their medical oncology outpatient review. The expectation that patients will self-report pain can be accommodated by healthcare professionals if a personalized pain goal is part of the cancer pain management plan during each clinical encounter. Healthcare professionals reported a need to take distinct responsibility for supplementing their dearth of knowledge, skills and beliefs regarding assessing and managing patients' cancer pain. Optimal pain management stems from an interprofessional approach that was applied in this study design.

Institutions:
(O'Connor L) UCD School of Nursing Midwifery & Health Systems, University College Dublin, Dublin 4, Ireland, D004ViW8, Ireland.; (Hassett A) Pain Medicine, Mater Misericordiae University Hospital, Dublin, Dublin 7, Ireland, D07 AX57, Ireland.; (Sheridan N) Cancer Directorate, Mater Misericordiae University Hospital, Dublin, Dublin 7, Ireland, D07 AX57, Ireland.
(O'Connor L) UCD School of Nursing Midwifery & Health Systems, University College Dublin, Dublin 4, Ireland, D004ViW8, Ireland.; (Hassett A) Pain Medicine, Mater Misericordiae University Hospital, Dublin, Dublin 7, Ireland, D07 AX57, Ireland.; (Sheridan N) Cancer Directorate, Mater Misericordiae University Hospital, Dublin, Dublin 7, Ireland, D07 AX57, Ireland.
(O'Connor L) UCD School of Nursing Midwifery & Health Systems, University College Dublin, Dublin 4, Ireland, D004ViW8, Ireland.; (Hassett A) Pain Medicine, Mater Misericordiae University Hospital, Dublin, Dublin 7, Ireland, D07 AX57, Ireland.; (Sheridan N) Cancer Directorate, Mater Misericordiae University Hospital, Dublin, Dublin 7, Ireland, D07 AX57, Ireland.
Database: PubMed

2. Differences in Attitudes and Practices of Cancer Pain Management between Medical Oncologists and Palliative Care Physicians.

Author(s): Kunitomi T; Nasu J; Minami D; Iwamoto T; Nishie H; Saito S; Fujiwara T; Matsuoka J

Source: Acta medica Okayama; Aug 2021; vol. 75 (no. 4); p. 431-437

Publication Date: Aug 2021

Publication Type(s): Journal Article

DOI: http://dx.doi.org/10.18926/AMO/62394

ISSN: 0386-300X

Place of Publication: Japan

PubMedID: 34511609

Accession Number: 34511609

Keywords: Subject Terms: barriers; cancer pain management; medical oncologist; opioid; palliative care physician

Abstract: This study aimed to evaluate whether there are differences in the attitudes and practices of cancer pain manage-ment between medical oncologists and palliative care physicians. An online nationwide survey was used to collect responses from board-certified medical oncologists and palliative care physicians in Japan. The survey questionnaire comprised 30 questions. The differences in responses between medical oncologists and palliative care physicians were examined. Out of the 1,227 questionnaires sent, 522 (42.5%) were returned. After apply-ing the exclusion criteria, 445 questionnaires (medical oncologists: n = 283; palliative care physicians: n = 162) were retained for analysis. Among the questions about potential barriers to optimal cancer pain man-agement, both medical oncologists and palliative care physicians considered the reluctance of patients to take opioids due to fear of adverse effects as the greatest barrier. Significantly different ratings between medical oncologists and palliative care physicians were observed on 5 of the 8 questions in this area. Significantly differ-ent ratings were

observed for all questions concerning pain specialists and their knowledge. For effective cancer pain management, it is important to account for differences in attitudes and practice between medical oncolo-gists and palliative care physicians.

Institutions:

(Kunitomi T) Department of Gastroenterological Surgery, Okayama University Graduate School of Medicine, Dentistry and Pharmaceutical Sciences.; (Nasu J) Department of Internal Medicine, Okayama Saiseikai General Hospital.; (Minami D) Department of Palliative Care Team, Okayama University Hospital.; (Iwamoto T) Department of Gastroenterological Surgery, Okayama University Graduate School of Medicine, Dentistry and Pharmaceutical Sciences.; (Nishie H) Department of Palliative Care Team, Okayama University Hospital.; (Saito S) Department of Palliative Care Team, Okayama University Hospital.; (Fujiwara T) Department of Gastroenterological Surgery, Okayama University Graduate School of Medicine, Dentistry and Pharmaceutical Sciences.; (Matsuoka J) Department of Gastroenterological Surgery, Okayama University Graduate School of Medicine, Dentistry and Pharmaceutical Sciences.
(Kunitomi T) Department of Gastroenterological Surgery, Okayama University Graduate School of Medicine, Dentistry and Pharmaceutical Sciences.; (Nasu J) Department of Internal Medicine, Okayama Saiseikai General Hospital.; (Minami D) Department of Palliative Care Team, Okayama University Hospital.; (Iwamoto T) Department of Gastroenterological Surgery, Okayama University Graduate School of Medicine, Dentistry and Pharmaceutical Sciences.; (Nishie H) Department of Palliative Care Team, Okayama University Hospital.; (Saito S) Department of Palliative Care Team, Okayama University Hospital.; (Fujiwara T) Department of Gastroenterological Surgery, Okayama University Graduate School of Medicine, Dentistry and Pharmaceutical Sciences.; (Matsuoka J) Department of Gastroenterological Surgery, Okayama University Graduate School of Medicine, Dentistry and Pharmaceutical Sciences.
(Kunitomi T) Department of Gastroenterological Surgery, Okayama University Graduate School of Medicine, Dentistry and Pharmaceutical Sciences.; (Nasu J) Department of Internal Medicine, Okayama Saiseikai General Hospital.; (Minami D) Department of Palliative Care Team, Okayama University Hospital.; (Iwamoto T) Department of Gastroenterological Surgery, Okayama University Graduate School of Medicine, Dentistry and Pharmaceutical Sciences.; (Nishie H) Department of Palliative Care Team, Okayama University Hospital.; (Saito S) Department of Palliative Care Team, Okayama University Hospital.; (Fujiwara T) Department of Gastroenterological Surgery, Okayama University Graduate School of Medicine, Dentistry and Pharmaceutical Sciences.; (Matsuoka J) Department of Gastroenterological Surgery, Okayama University Graduate School of Medicine, Dentistry and Pharmaceutical Sciences.
(Kunitomi T) Department of Gastroenterological Surgery, Okayama University Graduate School of Medicine, Dentistry and Pharmaceutical Sciences.; (Nasu J) Department of Internal Medicine, Okayama Saiseikai General Hospital.; (Minami D) Department of Palliative Care Team, Okayama University Hospital.; (Iwamoto T) Department of Gastroenterological Surgery, Okayama University Graduate School of Medicine, Dentistry and Pharmaceutical Sciences.; (Nishie H) Department of Palliative Care Team, Okayama University Hospital.; (Saito S) Department of Palliative Care Team, Okayama University Hospital.; (Fujiwara T) Department of Gastroenterological Surgery, Okayama University Graduate School of Medicine, Dentistry and Pharmaceutical Sciences.; (Matsuoka J) Department of

Gastroenterological Surgery, Okayama University Graduate School of Medicine, Dentistry and Pharmaceutical Sciences.
(Kunitomi T) Department of Gastroenterological Surgery, Okayama University Graduate School of Medicine, Dentistry and Pharmaceutical Sciences.; (Nasu J) Department of Internal Medicine, Okayama Saiseikai General Hospital.; (Minami D) Department of Palliative Care Team, Okayama University Hospital.; (Iwamoto T) Department of Gastroenterological Surgery, Okayama University Graduate School of Medicine, Dentistry and Pharmaceutical Sciences.; (Nishie H) Department of Palliative Care Team, Okayama University Hospital.; (Saito S) Department of Palliative Care Team, Okayama University Hospital.; (Fujiwara T) Department of Gastroenterological Surgery, Okayama University Graduate School of Medicine, Dentistry and Pharmaceutical Sciences.; (Matsuoka J) Department of Gastroenterological Surgery, Okayama University Graduate School of Medicine, Dentistry and Pharmaceutical Sciences.
(Kunitomi T) Department of Gastroenterological Surgery, Okayama University Graduate School of Medicine, Dentistry and Pharmaceutical Sciences.; (Nasu J) Department of Internal Medicine, Okayama Saiseikai General Hospital.; (Minami D) Department of Palliative Care Team, Okayama University Hospital.; (Iwamoto T) Department of Gastroenterological Surgery, Okayama University Graduate School of Medicine, Dentistry and Pharmaceutical Sciences.; (Nishie H) Department of Palliative Care Team, Okayama University Hospital.; (Saito S) Department of Palliative Care Team, Okayama University Hospital.; (Fujiwara T) Department of Gastroenterological Surgery, Okayama University Graduate School of Medicine, Dentistry and Pharmaceutical Sciences.; (Matsuoka J) Department of Gastroenterological Surgery, Okayama University Graduate School of Medicine, Dentistry and Pharmaceutical Sciences.
(Kunitomi T) Department of Gastroenterological Surgery, Okayama University Graduate School of Medicine, Dentistry and Pharmaceutical Sciences.; (Nasu J) Department of Internal Medicine, Okayama Saiseikai General Hospital.; (Minami D) Department of Palliative Care Team, Okayama University Hospital.; (Iwamoto T) Department of Gastroenterological Surgery, Okayama University Graduate School of Medicine, Dentistry and Pharmaceutical Sciences.; (Nishie H) Department of Palliative Care Team, Okayama University Hospital.; (Saito S) Department of Palliative Care Team, Okayama University Hospital.; (Fujiwara T) Department of Gastroenterological Surgery, Okayama University Graduate School of Medicine, Dentistry and Pharmaceutical Sciences.; (Matsuoka J) Department of Gastroenterological Surgery, Okayama University Graduate School of Medicine, Dentistry and Pharmaceutical Sciences.
(Kunitomi T) Department of Gastroenterological Surgery, Okayama University Graduate School of Medicine, Dentistry and Pharmaceutical Sciences.; (Nasu J) Department of Internal Medicine, Okayama Saiseikai General Hospital.; (Minami D) Department of Palliative Care Team, Okayama University Hospital.; (Iwamoto T) Department of Gastroenterological Surgery, Okayama University Graduate School of Medicine, Dentistry and Pharmaceutical Sciences.; (Nishie H) Department of Palliative Care Team, Okayama University Hospital.; (Saito S) Department of Palliative Care Team, Okayama University Hospital.; (Fujiwara T) Department of Gastroenterological Surgery, Okayama University Graduate School of Medicine, Dentistry and Pharmaceutical Sciences.; (Matsuoka J) Department of Gastroenterological Surgery, Okayama University Graduate School of Medicine, Dentistry and Pharmaceutical Sciences.

Database: PubMed

3. A Comparative Study of Seminars Combined with Case-Based Learning versus Lecture-Based Learning for Cancer Pain Teaching in Medical Oncology Internship.

Author(s): Yang Y; Yao JH; Xu LJ; Zhou ZG; Wang MX; Wang ZS; Zhao FY

Source: Journal of pain research; 2021; vol. 14 ; p. 2665-2675

Publication Date: 2021

Publication Type(s): Journal Article

DOI: http://dx.doi.org/10.2147/JPR.S320498

ISSN: 1178-7090

Place of Publication: New Zealand

PubMedID: 34483686

Accession Number: 34483686

Available at Journal of pain research - from Europe PubMed Central - Open Access

Keywords: Subject Terms: cancer pain teaching; case-based learning; comparative study; lecture-based learning; seminar method

Abstract: Purpose: To determine whether the teaching method of seminars combined with case-based learning (CBL) is superior to the traditional lecture-based learning (LBL) for teaching cancer pain in medical oncology internship.Methods: Sixty medical and nursing interns in the medical oncology department of our hospital were selected between January 2019 and December 2020. Thirty students received traditional LBL instruction as the control group, and 30 students received combined seminars and CBL instruction as the observation group. The teaching evaluation and assessment was performed by theoretical and practical examinations and questionnaires.Results: In the after-class examination, case analysis, clinical practice and overall scores of the observation group were higher than those of the control group (all $p < 0.001$). Theoretical knowledge scores did not differ significantly between the two groups ($p = 0.470$). In the questionnaire regarding attitudes towards opioid use, the observation group had better perceptions of using opioids than the control group (all $p < 0.01$). In the meantime, students in the observation group outperformed the control group in four aspects: self-learning ($p < 0.001$), analytical and problem-solving ($p < 0.001$), clinical thinking ($p = 0.001$), and clinical practice ($p = 0.002$) abilities all improved, while stimulating learning interest ($p = 0.184$) and enhancing theoretical knowledge mastery ($p = 0.221$) were not significantly different from those of the control group. Overall, students in the observation group were more satisfied with the teaching, teaching methods and teacher performances than the control group (all $p < 0.001$).Conclusion: Compared to the LBL, the combination of seminars and CBL is a more effective teaching method for cancer pain management, which is worth further study.

Institutions:
(Yang Y) Department of Medical Oncology, The First Affiliated Hospital of Bengbu Medical College, Bengbu, Anhui, People's Republic of China.; (Yao JH) Department of Medical Oncology, The First Affiliated Hospital of Bengbu Medical College, Bengbu, Anhui, People's Republic of China.; (Xu LJ) Department of Medical

Oncology, The First Affiliated Hospital of Bengbu Medical College, Bengbu, Anhui, People's Republic of China.; (Zhou ZG) Department of Medical Oncology, The First Affiliated Hospital of Bengbu Medical College, Bengbu, Anhui, People's Republic of China.; (Wang MX) Department of Medical Oncology, The First Affiliated Hospital of Bengbu Medical College, Bengbu, Anhui, People's Republic of China.; (Wang ZS) Department of Medical Oncology, The First Affiliated Hospital of Bengbu Medical College, Bengbu, Anhui, People's Republic of China.; (Zhao FY) Department of Medical Oncology, The First Affiliated Hospital of Bengbu Medical College, Bengbu, Anhui, People's Republic of China.

(Yang Y) Department of Medical Oncology, The First Affiliated Hospital of Bengbu Medical College, Bengbu, Anhui, People's Republic of China.; (Yao JH) Department of Medical Oncology, The First Affiliated Hospital of Bengbu Medical College, Bengbu, Anhui, People's Republic of China.; (Xu LJ) Department of Medical Oncology, The First Affiliated Hospital of Bengbu Medical College, Bengbu, Anhui, People's Republic of China.; (Zhou ZG) Department of Medical Oncology, The First Affiliated Hospital of Bengbu Medical College, Bengbu, Anhui, People's Republic of China.; (Wang MX) Department of Medical Oncology, The First Affiliated Hospital of Bengbu Medical College, Bengbu, Anhui, People's Republic of China.; (Wang ZS) Department of Medical Oncology, The First Affiliated Hospital of Bengbu Medical College, Bengbu, Anhui, People's Republic of China.; (Zhao FY) Department of Medical Oncology, The First Affiliated Hospital of Bengbu Medical College, Bengbu, Anhui, People's Republic of China.

(Yang Y) Department of Medical Oncology, The First Affiliated Hospital of Bengbu Medical College, Bengbu, Anhui, People's Republic of China.; (Yao JH) Department of Medical Oncology, The First Affiliated Hospital of Bengbu Medical College, Bengbu, Anhui, People's Republic of China.; (Xu LJ) Department of Medical Oncology, The First Affiliated Hospital of Bengbu Medical College, Bengbu, Anhui, People's Republic of China.; (Zhou ZG) Department of Medical Oncology, The First Affiliated Hospital of Bengbu Medical College, Bengbu, Anhui, People's Republic of China.; (Wang MX) Department of Medical Oncology, The First Affiliated Hospital of Bengbu Medical College, Bengbu, Anhui, People's Republic of China.; (Wang ZS) Department of Medical Oncology, The First Affiliated Hospital of Bengbu Medical College, Bengbu, Anhui, People's Republic of China.; (Zhao FY) Department of Medical Oncology, The First Affiliated Hospital of Bengbu Medical College, Bengbu, Anhui, People's Republic of China.

(Yang Y) Department of Medical Oncology, The First Affiliated Hospital of Bengbu Medical College, Bengbu, Anhui, People's Republic of China.; (Yao JH) Department of Medical Oncology, The First Affiliated Hospital of Bengbu Medical College, Bengbu, Anhui, People's Republic of China.; (Xu LJ) Department of Medical Oncology, The First Affiliated Hospital of Bengbu Medical College, Bengbu, Anhui, People's Republic of China.; (Zhou ZG) Department of Medical Oncology, The First Affiliated Hospital of Bengbu Medical College, Bengbu, Anhui, People's Republic of China.; (Wang MX) Department of Medical Oncology, The First Affiliated Hospital of Bengbu Medical College, Bengbu, Anhui, People's Republic of China.; (Wang ZS) Department of Medical Oncology, The First Affiliated Hospital of Bengbu Medical College, Bengbu, Anhui, People's Republic of China.; (Zhao FY) Department of Medical Oncology, The First Affiliated Hospital of Bengbu Medical College, Bengbu, Anhui, People's Republic of China.

(Yang Y) Department of Medical Oncology, The First Affiliated Hospital of Bengbu Medical College, Bengbu, Anhui, People's Republic of China.; (Yao JH) Department

of Medical Oncology, The First Affiliated Hospital of Bengbu Medical College, Bengbu, Anhui, People's Republic of China.; (Xu LJ) Department of Medical Oncology, The First Affiliated Hospital of Bengbu Medical College, Bengbu, Anhui, People's Republic of China.; (Zhou ZG) Department of Medical Oncology, The First Affiliated Hospital of Bengbu Medical College, Bengbu, Anhui, People's Republic of China.; (Wang MX) Department of Medical Oncology, The First Affiliated Hospital of Bengbu Medical College, Bengbu, Anhui, People's Republic of China.; (Wang ZS) Department of Medical Oncology, The First Affiliated Hospital of Bengbu Medical College, Bengbu, Anhui, People's Republic of China.; (Zhao FY) Department of Medical Oncology, The First Affiliated Hospital of Bengbu Medical College, Bengbu, Anhui, People's Republic of China.
(Yang Y) Department of Medical Oncology, The First Affiliated Hospital of Bengbu Medical College, Bengbu, Anhui, People's Republic of China.; (Yao JH) Department of Medical Oncology, The First Affiliated Hospital of Bengbu Medical College, Bengbu, Anhui, People's Republic of China.; (Xu LJ) Department of Medical Oncology, The First Affiliated Hospital of Bengbu Medical College, Bengbu, Anhui, People's Republic of China.; (Zhou ZG) Department of Medical Oncology, The First Affiliated Hospital of Bengbu Medical College, Bengbu, Anhui, People's Republic of China.; (Wang MX) Department of Medical Oncology, The First Affiliated Hospital of Bengbu Medical College, Bengbu, Anhui, People's Republic of China.; (Wang ZS) Department of Medical Oncology, The First Affiliated Hospital of Bengbu Medical College, Bengbu, Anhui, People's Republic of China.; (Zhao FY) Department of Medical Oncology, The First Affiliated Hospital of Bengbu Medical College, Bengbu, Anhui, People's Republic of China.
(Yang Y) Department of Medical Oncology, The First Affiliated Hospital of Bengbu Medical College, Bengbu, Anhui, People's Republic of China.; (Yao JH) Department of Medical Oncology, The First Affiliated Hospital of Bengbu Medical College, Bengbu, Anhui, People's Republic of China.; (Xu LJ) Department of Medical Oncology, The First Affiliated Hospital of Bengbu Medical College, Bengbu, Anhui, People's Republic of China.; (Zhou ZG) Department of Medical Oncology, The First Affiliated Hospital of Bengbu Medical College, Bengbu, Anhui, People's Republic of China.; (Wang MX) Department of Medical Oncology, The First Affiliated Hospital of Bengbu Medical College, Bengbu, Anhui, People's Republic of China.; (Wang ZS) Department of Medical Oncology, The First Affiliated Hospital of Bengbu Medical College, Bengbu, Anhui, People's Republic of China.; (Zhao FY) Department of Medical Oncology, The First Affiliated Hospital of Bengbu Medical College, Bengbu, Anhui, People's Republic of China.
Database: PubMed

4. Practice, Knowledge, and Attitude of Health Care Providers regarding Cancer Pain Management: A National Survey.

Author(s): Liu J; Zhang M; Luo J; Xie J; Chen X; Wang H; Li S; Yang S; Peng C; Yang L; Deng B; Zhang Y; Wang C; Hu J; Shi C

Source: Pain research & management; 2021; vol. 2021 ; p. 1247202

Publication Date: 2021

Publication Type(s): Journal Article; Research Support, Non-U.S. Gov't

DOI: http://dx.doi.org/10.1155/2021/1247202

ISSN: 1918-1523
Place of Publication: United States
PubMedID: 34471442
Accession Number: 34471442

Available at Pain research & management - from Europe PubMed Central - Open Access

Available at Pain research & management - from Hindawi Open Access Journals

Available at Pain research & management - from EBSCO (MEDLINE Complete)

Abstract: Background: A lack of knowledge and inadequate practices of health care providers (HCPs) are the main obstacles to effective cancer pain management (CPM). The main objective of the study was to evaluate the CPM knowledge, CPM practice, and attitudes towards pharmacists' participation and advanced methods in CPM of physicians, nurses, and pharmacists in China.Methods: An open online survey was adopted using social media software (WeChat) as the platform to conduct a nationwide survey of HCPs involved in CPM in public medical institutions at all levels in China from March to June 2019.Results: A total of 1279 physicians, 2267 nurses, and 1466 pharmacists participated in the survey. Among the three types of professionals, nurses had the highest level of practical ability (61.63 ± 28.99) and best attitudes towards pharmacists' participation and advanced methods in CPM (72.05 ± 33.71) and physicians had the best mastery of CPM-related knowledge (69.60 ± 28.45), while pharmacists performed the worst in these three aspects (50.04 ± 26.69, 61.49 ± 28.95, and 62.07 ± 36.46, respectively). Only 19.69% of the hospitals had a pharmacist to tumor patient ratio ≥1 : 50. Hierarchical analysis showed that passing a good pain management (GPM) ward program and participating in advanced training had positive impacts on the scores of all three parts in the three professions (ptrend <0.05).Conclusions: HCPs' levels of practice, knowledge, and attitudes towards pharmacists and advanced methods of CPM were average in China; however, pharmacists had the worst performance, which demonstrates a need for further improvement. Furthermore, GPM ward programs and advanced trainings are helpful for improving CPM levels.

Institutions:
(Liu J) Department of Pharmacy, Union Hospital, Tongji Medical College, Huazhong University of Science and Technology, Wuhan 430022, China.; (Zhang M) President's Office of Union Hospital, Tongji Medical College, Huazhong University of Science and Technology (HUST), Wuhan, China.; (Luo J) Department of Pharmacy, Union Hospital, Tongji Medical College, Huazhong University of Science and Technology, Wuhan 430022, China.; (Xie J) Department of Pharmacy, Union Hospital, Tongji Medical College, Huazhong University of Science and Technology, Wuhan 430022, China.; (Chen X) Department of Pharmacy, Union Hospital, Tongji Medical College, Huazhong University of Science and Technology, Wuhan 430022, China.; (Wang H) Department of Pharmacy, Union Hospital, Tongji Medical College, Huazhong University of Science and Technology, Wuhan 430022, China.; (Li S) Department of Pharmacy, Union Hospital, Tongji Medical College, Huazhong University of Science and Technology, Wuhan 430022, China.; (Yang S) Cancer Center, Union Hospital, Tongji Medical College, Huazhong University of Science and Technology, Wuhan, China.; (Peng C) Cancer Center, Union Hospital, Tongji Medical College, Huazhong University of Science and Technology, Wuhan, China.; (Yang L) Department of Occupational and Environmental Health, Ministry of Education and

Ministry of Environmental Protection, and Key Laboratory of Environmental Health, School of Public Health, Tongji Medical College, Huazhong University of Science and Technology, Wuhan, China.; (Deng B) Department of Pharmacy, Union Hospital, Tongji Medical College, Huazhong University of Science and Technology, Wuhan 430022, China.; (Zhang Y) Department of Pharmacy, Union Hospital, Tongji Medical College, Huazhong University of Science and Technology, Wuhan 430022, China.; (Wang C) Department of Pharmacy, Union Hospital, Tongji Medical College, Huazhong University of Science and Technology, Wuhan 430022, China.; (Hu J) Cancer Center, Union Hospital, Tongji Medical College, Huazhong University of Science and Technology, Wuhan, China.; (Shi C) Department of Pharmacy, Union Hospital, Tongji Medical College, Huazhong University of Science and Technology, Wuhan 430022, China.
(Liu J) Department of Pharmacy, Union Hospital, Tongji Medical College, Huazhong University of Science and Technology, Wuhan 430022, China.; (Zhang M) President's Office of Union Hospital, Tongji Medical College, Huazhong University of Science and Technology (HUST), Wuhan, China.; (Luo J) Department of Pharmacy, Union Hospital, Tongji Medical College, Huazhong University of Science and Technology, Wuhan 430022, China.; (Xie J) Department of Pharmacy, Union Hospital, Tongji Medical College, Huazhong University of Science and Technology, Wuhan 430022, China.; (Chen X) Department of Pharmacy, Union Hospital, Tongji Medical College, Huazhong University of Science and Technology, Wuhan 430022, China.; (Wang H) Department of Pharmacy, Union Hospital, Tongji Medical College, Huazhong University of Science and Technology, Wuhan 430022, China.; (Li S) Department of Pharmacy, Union Hospital, Tongji Medical College, Huazhong University of Science and Technology, Wuhan 430022, China.; (Yang S) Cancer Center, Union Hospital, Tongji Medical College, Huazhong University of Science and Technology, Wuhan, China.; (Peng C) Cancer Center, Union Hospital, Tongji Medical College, Huazhong University of Science and Technology, Wuhan, China.; (Yang L) Department of Occupational and Environmental Health, Ministry of Education and Ministry of Environmental Protection, and Key Laboratory of Environmental Health, School of Public Health, Tongji Medical College, Huazhong University of Science and Technology, Wuhan, China.; (Deng B) Department of Pharmacy, Union Hospital, Tongji Medical College, Huazhong University of Science and Technology, Wuhan 430022, China.; (Zhang Y) Department of Pharmacy, Union Hospital, Tongji Medical College, Huazhong University of Science and Technology, Wuhan 430022, China.; (Wang C) Department of Pharmacy, Union Hospital, Tongji Medical College, Huazhong University of Science and Technology, Wuhan 430022, China.; (Hu J) Cancer Center, Union Hospital, Tongji Medical College, Huazhong University of Science and Technology, Wuhan, China.; (Shi C) Department of Pharmacy, Union Hospital, Tongji Medical College, Huazhong University of Science and Technology, Wuhan 430022, China.
(Liu J) Department of Pharmacy, Union Hospital, Tongji Medical College, Huazhong University of Science and Technology, Wuhan 430022, China.; (Zhang M) President's Office of Union Hospital, Tongji Medical College, Huazhong University of Science and Technology (HUST), Wuhan, China.; (Luo J) Department of Pharmacy, Union Hospital, Tongji Medical College, Huazhong University of Science and Technology, Wuhan 430022, China.; (Xie J) Department of Pharmacy, Union Hospital, Tongji Medical College, Huazhong University of Science and Technology, Wuhan 430022, China.; (Chen X) Department of Pharmacy, Union Hospital, Tongji Medical College, Huazhong University of Science and Technology, Wuhan 430022,

China.; (Wang H) Department of Pharmacy, Union Hospital, Tongji Medical College, Huazhong University of Science and Technology, Wuhan 430022, China.; (Li S) Department of Pharmacy, Union Hospital, Tongji Medical College, Huazhong University of Science and Technology, Wuhan 430022, China.; (Yang S) Cancer Center, Union Hospital, Tongji Medical College, Huazhong University of Science and Technology, Wuhan, China.; (Peng C) Cancer Center, Union Hospital, Tongji Medical College, Huazhong University of Science and Technology, Wuhan, China.; (Yang L) Department of Occupational and Environmental Health, Ministry of Education and Ministry of Environmental Protection, and Key Laboratory of Environmental Health, School of Public Health, Tongji Medical College, Huazhong University of Science and Technology, Wuhan, China.; (Deng B) Department of Pharmacy, Union Hospital, Tongji Medical College, Huazhong University of Science and Technology, Wuhan 430022, China.; (Zhang Y) Department of Pharmacy, Union Hospital, Tongji Medical College, Huazhong University of Science and Technology, Wuhan 430022, China.; (Wang C) Department of Pharmacy, Union Hospital, Tongji Medical College, Huazhong University of Science and Technology, Wuhan 430022, China.; (Hu J) Cancer Center, Union Hospital, Tongji Medical College, Huazhong University of Science and Technology, Wuhan, China.; (Shi C) Department of Pharmacy, Union Hospital, Tongji Medical College, Huazhong University of Science and Technology, Wuhan 430022, China.

(Liu J) Department of Pharmacy, Union Hospital, Tongji Medical College, Huazhong University of Science and Technology, Wuhan 430022, China.; (Zhang M) President's Office of Union Hospital, Tongji Medical College, Huazhong University of Science and Technology (HUST), Wuhan, China.; (Luo J) Department of Pharmacy, Union Hospital, Tongji Medical College, Huazhong University of Science and Technology, Wuhan 430022, China.; (Xie J) Department of Pharmacy, Union Hospital, Tongji Medical College, Huazhong University of Science and Technology, Wuhan 430022, China.; (Chen X) Department of Pharmacy, Union Hospital, Tongji Medical College, Huazhong University of Science and Technology, Wuhan 430022, China.; (Wang H) Department of Pharmacy, Union Hospital, Tongji Medical College, Huazhong University of Science and Technology, Wuhan 430022, China.; (Li S) Department of Pharmacy, Union Hospital, Tongji Medical College, Huazhong University of Science and Technology, Wuhan 430022, China.; (Yang S) Cancer Center, Union Hospital, Tongji Medical College, Huazhong University of Science and Technology, Wuhan, China.; (Peng C) Cancer Center, Union Hospital, Tongji Medical College, Huazhong University of Science and Technology, Wuhan, China.; (Yang L) Department of Occupational and Environmental Health, Ministry of Education and Ministry of Environmental Protection, and Key Laboratory of Environmental Health, School of Public Health, Tongji Medical College, Huazhong University of Science and Technology, Wuhan, China.; (Deng B) Department of Pharmacy, Union Hospital, Tongji Medical College, Huazhong University of Science and Technology, Wuhan 430022, China.; (Zhang Y) Department of Pharmacy, Union Hospital, Tongji Medical College, Huazhong University of Science and Technology, Wuhan 430022, China.; (Wang C) Department of Pharmacy, Union Hospital, Tongji Medical College, Huazhong University of Science and Technology, Wuhan 430022, China.; (Hu J) Cancer Center, Union Hospital, Tongji Medical College, Huazhong University of Science and Technology, Wuhan, China.; (Shi C) Department of Pharmacy, Union Hospital, Tongji Medical College, Huazhong University of Science and Technology, Wuhan 430022, China.

(Liu J) Department of Pharmacy, Union Hospital, Tongji Medical College, Huazhong University of Science and Technology, Wuhan 430022, China.; (Zhang M) President's Office of Union Hospital, Tongji Medical College, Huazhong University of Science and Technology (HUST), Wuhan, China.; (Luo J) Department of Pharmacy, Union Hospital, Tongji Medical College, Huazhong University of Science and Technology, Wuhan 430022, China.; (Xie J) Department of Pharmacy, Union Hospital, Tongji Medical College, Huazhong University of Science and Technology, Wuhan 430022, China.; (Chen X) Department of Pharmacy, Union Hospital, Tongji Medical College, Huazhong University of Science and Technology, Wuhan 430022, China.; (Wang H) Department of Pharmacy, Union Hospital, Tongji Medical College, Huazhong University of Science and Technology, Wuhan 430022, China.; (Li S) Department of Pharmacy, Union Hospital, Tongji Medical College, Huazhong University of Science and Technology, Wuhan 430022, China.; (Yang S) Cancer Center, Union Hospital, Tongji Medical College, Huazhong University of Science and Technology, Wuhan, China.; (Peng C) Cancer Center, Union Hospital, Tongji Medical College, Huazhong University of Science and Technology, Wuhan, China.; (Yang L) Department of Occupational and Environmental Health, Ministry of Education and Ministry of Environmental Protection, and Key Laboratory of Environmental Health, School of Public Health, Tongji Medical College, Huazhong University of Science and Technology, Wuhan, China.; (Deng B) Department of Pharmacy, Union Hospital, Tongji Medical College, Huazhong University of Science and Technology, Wuhan 430022, China.; (Zhang Y) Department of Pharmacy, Union Hospital, Tongji Medical College, Huazhong University of Science and Technology, Wuhan 430022, China.; (Wang C) Department of Pharmacy, Union Hospital, Tongji Medical College, Huazhong University of Science and Technology, Wuhan 430022, China.; (Hu J) Cancer Center, Union Hospital, Tongji Medical College, Huazhong University of Science and Technology, Wuhan, China.; (Shi C) Department of Pharmacy, Union Hospital, Tongji Medical College, Huazhong University of Science and Technology, Wuhan 430022, China.
(Liu J) Department of Pharmacy, Union Hospital, Tongji Medical College, Huazhong University of Science and Technology, Wuhan 430022, China.; (Zhang M) President's Office of Union Hospital, Tongji Medical College, Huazhong University of Science and Technology (HUST), Wuhan, China.; (Luo J) Department of Pharmacy, Union Hospital, Tongji Medical College, Huazhong University of Science and Technology, Wuhan 430022, China.; (Xie J) Department of Pharmacy, Union Hospital, Tongji Medical College, Huazhong University of Science and Technology, Wuhan 430022, China.; (Chen X) Department of Pharmacy, Union Hospital, Tongji Medical College, Huazhong University of Science and Technology, Wuhan 430022, China.; (Wang H) Department of Pharmacy, Union Hospital, Tongji Medical College, Huazhong University of Science and Technology, Wuhan 430022, China.; (Li S) Department of Pharmacy, Union Hospital, Tongji Medical College, Huazhong University of Science and Technology, Wuhan 430022, China.; (Yang S) Cancer Center, Union Hospital, Tongji Medical College, Huazhong University of Science and Technology, Wuhan, China.; (Peng C) Cancer Center, Union Hospital, Tongji Medical College, Huazhong University of Science and Technology, Wuhan, China.; (Yang L) Department of Occupational and Environmental Health, Ministry of Education and Ministry of Environmental Protection, and Key Laboratory of Environmental Health, School of Public Health, Tongji Medical College, Huazhong University of Science and Technology, Wuhan, China.; (Deng B) Department of Pharmacy, Union Hospital, Tongji Medical College, Huazhong University of Science and Technology, Wuhan

430022, China.; (Zhang Y) Department of Pharmacy, Union Hospital, Tongji Medical College, Huazhong University of Science and Technology, Wuhan 430022, China.; (Wang C) Department of Pharmacy, Union Hospital, Tongji Medical College, Huazhong University of Science and Technology, Wuhan 430022, China.; (Hu J) Cancer Center, Union Hospital, Tongji Medical College, Huazhong University of Science and Technology, Wuhan, China.; (Shi C) Department of Pharmacy, Union Hospital, Tongji Medical College, Huazhong University of Science and Technology, Wuhan 430022, China.
(Liu J) Department of Pharmacy, Union Hospital, Tongji Medical College, Huazhong University of Science and Technology, Wuhan 430022, China.; (Zhang M) President's Office of Union Hospital, Tongji Medical College, Huazhong University of Science and Technology (HUST), Wuhan, China.; (Luo J) Department of Pharmacy, Union Hospital, Tongji Medical College, Huazhong University of Science and Technology, Wuhan 430022, China.; (Xie J) Department of Pharmacy, Union Hospital, Tongji Medical College, Huazhong University of Science and Technology, Wuhan 430022, China.; (Chen X) Department of Pharmacy, Union Hospital, Tongji Medical College, Huazhong University of Science and Technology, Wuhan 430022, China.; (Wang H) Department of Pharmacy, Union Hospital, Tongji Medical College, Huazhong University of Science and Technology, Wuhan 430022, China.; (Li S) Department of Pharmacy, Union Hospital, Tongji Medical College, Huazhong University of Science and Technology, Wuhan 430022, China.; (Yang S) Cancer Center, Union Hospital, Tongji Medical College, Huazhong University of Science and Technology, Wuhan, China.; (Peng C) Cancer Center, Union Hospital, Tongji Medical College, Huazhong University of Science and Technology, Wuhan, China.; (Yang L) Department of Occupational and Environmental Health, Ministry of Education and Ministry of Environmental Protection, and Key Laboratory of Environmental Health, School of Public Health, Tongji Medical College, Huazhong University of Science and Technology, Wuhan, China.; (Deng B) Department of Pharmacy, Union Hospital, Tongji Medical College, Huazhong University of Science and Technology, Wuhan 430022, China.; (Zhang Y) Department of Pharmacy, Union Hospital, Tongji Medical College, Huazhong University of Science and Technology, Wuhan 430022, China.; (Wang C) Department of Pharmacy, Union Hospital, Tongji Medical College, Huazhong University of Science and Technology, Wuhan 430022, China.; (Hu J) Cancer Center, Union Hospital, Tongji Medical College, Huazhong University of Science and Technology, Wuhan, China.; (Shi C) Department of Pharmacy, Union Hospital, Tongji Medical College, Huazhong University of Science and Technology, Wuhan 430022, China.
(Liu J) Department of Pharmacy, Union Hospital, Tongji Medical College, Huazhong University of Science and Technology, Wuhan 430022, China.; (Zhang M) President's Office of Union Hospital, Tongji Medical College, Huazhong University of Science and Technology (HUST), Wuhan, China.; (Luo J) Department of Pharmacy, Union Hospital, Tongji Medical College, Huazhong University of Science and Technology, Wuhan 430022, China.; (Xie J) Department of Pharmacy, Union Hospital, Tongji Medical College, Huazhong University of Science and Technology, Wuhan 430022, China.; (Chen X) Department of Pharmacy, Union Hospital, Tongji Medical College, Huazhong University of Science and Technology, Wuhan 430022, China.; (Wang H) Department of Pharmacy, Union Hospital, Tongji Medical College, Huazhong University of Science and Technology, Wuhan 430022, China.; (Li S) Department of Pharmacy, Union Hospital, Tongji Medical College, Huazhong University of Science and Technology, Wuhan 430022, China.; (Yang S) Cancer

Center, Union Hospital, Tongji Medical College, Huazhong University of Science and Technology, Wuhan, China.; (Peng C) Cancer Center, Union Hospital, Tongji Medical College, Huazhong University of Science and Technology, Wuhan, China.; (Yang L) Department of Occupational and Environmental Health, Ministry of Education and Ministry of Environmental Protection, and Key Laboratory of Environmental Health, School of Public Health, Tongji Medical College, Huazhong University of Science and Technology, Wuhan, China.; (Deng B) Department of Pharmacy, Union Hospital, Tongji Medical College, Huazhong University of Science and Technology, Wuhan 430022, China.; (Zhang Y) Department of Pharmacy, Union Hospital, Tongji Medical College, Huazhong University of Science and Technology, Wuhan 430022, China.; (Wang C) Department of Pharmacy, Union Hospital, Tongji Medical College, Huazhong University of Science and Technology, Wuhan 430022, China.; (Hu J) Cancer Center, Union Hospital, Tongji Medical College, Huazhong University of Science and Technology, Wuhan, China.; (Shi C) Department of Pharmacy, Union Hospital, Tongji Medical College, Huazhong University of Science and Technology, Wuhan 430022, China.
(Liu J) Department of Pharmacy, Union Hospital, Tongji Medical College, Huazhong University of Science and Technology, Wuhan 430022, China.; (Zhang M) President's Office of Union Hospital, Tongji Medical College, Huazhong University of Science and Technology (HUST), Wuhan, China.; (Luo J) Department of Pharmacy, Union Hospital, Tongji Medical College, Huazhong University of Science and Technology, Wuhan 430022, China.; (Xie J) Department of Pharmacy, Union Hospital, Tongji Medical College, Huazhong University of Science and Technology, Wuhan 430022, China.; (Chen X) Department of Pharmacy, Union Hospital, Tongji Medical College, Huazhong University of Science and Technology, Wuhan 430022, China.; (Wang H) Department of Pharmacy, Union Hospital, Tongji Medical College, Huazhong University of Science and Technology, Wuhan 430022, China.; (Li S) Department of Pharmacy, Union Hospital, Tongji Medical College, Huazhong University of Science and Technology, Wuhan 430022, China.; (Yang S) Cancer Center, Union Hospital, Tongji Medical College, Huazhong University of Science and Technology, Wuhan, China.; (Peng C) Cancer Center, Union Hospital, Tongji Medical College, Huazhong University of Science and Technology, Wuhan, China.; (Yang L) Department of Occupational and Environmental Health, Ministry of Education and Ministry of Environmental Protection, and Key Laboratory of Environmental Health, School of Public Health, Tongji Medical College, Huazhong University of Science and Technology, Wuhan, China.; (Deng B) Department of Pharmacy, Union Hospital, Tongji Medical College, Huazhong University of Science and Technology, Wuhan 430022, China.; (Zhang Y) Department of Pharmacy, Union Hospital, Tongji Medical College, Huazhong University of Science and Technology, Wuhan 430022, China.; (Wang C) Department of Pharmacy, Union Hospital, Tongji Medical College, Huazhong University of Science and Technology, Wuhan 430022, China.; (Hu J) Cancer Center, Union Hospital, Tongji Medical College, Huazhong University of Science and Technology, Wuhan, China.; (Shi C) Department of Pharmacy, Union Hospital, Tongji Medical College, Huazhong University of Science and Technology, Wuhan 430022, China.
(Liu J) Department of Pharmacy, Union Hospital, Tongji Medical College, Huazhong University of Science and Technology, Wuhan 430022, China.; (Zhang M) President's Office of Union Hospital, Tongji Medical College, Huazhong University of Science and Technology (HUST), Wuhan, China.; (Luo J) Department of Pharmacy, Union Hospital, Tongji Medical College, Huazhong University of Science and

Technology, Wuhan 430022, China.; (Xie J) Department of Pharmacy, Union Hospital, Tongji Medical College, Huazhong University of Science and Technology, Wuhan 430022, China.; (Chen X) Department of Pharmacy, Union Hospital, Tongji Medical College, Huazhong University of Science and Technology, Wuhan 430022, China.; (Wang H) Department of Pharmacy, Union Hospital, Tongji Medical College, Huazhong University of Science and Technology, Wuhan 430022, China.; (Li S) Department of Pharmacy, Union Hospital, Tongji Medical College, Huazhong University of Science and Technology, Wuhan 430022, China.; (Yang S) Cancer Center, Union Hospital, Tongji Medical College, Huazhong University of Science and Technology, Wuhan, China.; (Peng C) Cancer Center, Union Hospital, Tongji Medical College, Huazhong University of Science and Technology, Wuhan, China.; (Yang L) Department of Occupational and Environmental Health, Ministry of Education and Ministry of Environmental Protection, and Key Laboratory of Environmental Health, School of Public Health, Tongji Medical College, Huazhong University of Science and Technology, Wuhan, China.; (Deng B) Department of Pharmacy, Union Hospital, Tongji Medical College, Huazhong University of Science and Technology, Wuhan 430022, China.; (Zhang Y) Department of Pharmacy, Union Hospital, Tongji Medical College, Huazhong University of Science and Technology, Wuhan 430022, China.; (Wang C) Department of Pharmacy, Union Hospital, Tongji Medical College, Huazhong University of Science and Technology, Wuhan 430022, China.; (Hu J) Cancer Center, Union Hospital, Tongji Medical College, Huazhong University of Science and Technology, Wuhan, China.; (Shi C) Department of Pharmacy, Union Hospital, Tongji Medical College, Huazhong University of Science and Technology, Wuhan 430022, China.
(Liu J) Department of Pharmacy, Union Hospital, Tongji Medical College, Huazhong University of Science and Technology, Wuhan 430022, China.; (Zhang M) President's Office of Union Hospital, Tongji Medical College, Huazhong University of Science and Technology (HUST), Wuhan, China.; (Luo J) Department of Pharmacy, Union Hospital, Tongji Medical College, Huazhong University of Science and Technology, Wuhan 430022, China.; (Xie J) Department of Pharmacy, Union Hospital, Tongji Medical College, Huazhong University of Science and Technology, Wuhan 430022, China.; (Chen X) Department of Pharmacy, Union Hospital, Tongji Medical College, Huazhong University of Science and Technology, Wuhan 430022, China.; (Wang H) Department of Pharmacy, Union Hospital, Tongji Medical College, Huazhong University of Science and Technology, Wuhan 430022, China.; (Li S) Department of Pharmacy, Union Hospital, Tongji Medical College, Huazhong University of Science and Technology, Wuhan 430022, China.; (Yang S) Cancer Center, Union Hospital, Tongji Medical College, Huazhong University of Science and Technology, Wuhan, China.; (Peng C) Cancer Center, Union Hospital, Tongji Medical College, Huazhong University of Science and Technology, Wuhan, China.; (Yang L) Department of Occupational and Environmental Health, Ministry of Education and Ministry of Environmental Protection, and Key Laboratory of Environmental Health, School of Public Health, Tongji Medical College, Huazhong University of Science and Technology, Wuhan, China.; (Deng B) Department of Pharmacy, Union Hospital, Tongji Medical College, Huazhong University of Science and Technology, Wuhan 430022, China.; (Zhang Y) Department of Pharmacy, Union Hospital, Tongji Medical College, Huazhong University of Science and Technology, Wuhan 430022, China.; (Wang C) Department of Pharmacy, Union Hospital, Tongji Medical College, Huazhong University of Science and Technology, Wuhan 430022, China.; (Hu J) Cancer Center, Union Hospital, Tongji Medical College, Huazhong University of

Science and Technology, Wuhan, China.; (Shi C) Department of Pharmacy, Union Hospital, Tongji Medical College, Huazhong University of Science and Technology, Wuhan 430022, China.
(Liu J) Department of Pharmacy, Union Hospital, Tongji Medical College, Huazhong University of Science and Technology, Wuhan 430022, China.; (Zhang M) President's Office of Union Hospital, Tongji Medical College, Huazhong University of Science and Technology (HUST), Wuhan, China.; (Luo J) Department of Pharmacy, Union Hospital, Tongji Medical College, Huazhong University of Science and Technology, Wuhan 430022, China.; (Xie J) Department of Pharmacy, Union Hospital, Tongji Medical College, Huazhong University of Science and Technology, Wuhan 430022, China.; (Chen X) Department of Pharmacy, Union Hospital, Tongji Medical College, Huazhong University of Science and Technology, Wuhan 430022, China.; (Wang H) Department of Pharmacy, Union Hospital, Tongji Medical College, Huazhong University of Science and Technology, Wuhan 430022, China.; (Li S) Department of Pharmacy, Union Hospital, Tongji Medical College, Huazhong University of Science and Technology, Wuhan 430022, China.; (Yang S) Cancer Center, Union Hospital, Tongji Medical College, Huazhong University of Science and Technology, Wuhan, China.; (Peng C) Cancer Center, Union Hospital, Tongji Medical College, Huazhong University of Science and Technology, Wuhan, China.; (Yang L) Department of Occupational and Environmental Health, Ministry of Education and Ministry of Environmental Protection, and Key Laboratory of Environmental Health, School of Public Health, Tongji Medical College, Huazhong University of Science and Technology, Wuhan, China.; (Deng B) Department of Pharmacy, Union Hospital, Tongji Medical College, Huazhong University of Science and Technology, Wuhan 430022, China.; (Zhang Y) Department of Pharmacy, Union Hospital, Tongji Medical College, Huazhong University of Science and Technology, Wuhan 430022, China.; (Wang C) Department of Pharmacy, Union Hospital, Tongji Medical College, Huazhong University of Science and Technology, Wuhan 430022, China.; (Hu J) Cancer Center, Union Hospital, Tongji Medical College, Huazhong University of Science and Technology, Wuhan, China.; (Shi C) Department of Pharmacy, Union Hospital, Tongji Medical College, Huazhong University of Science and Technology, Wuhan 430022, China.
(Liu J) Department of Pharmacy, Union Hospital, Tongji Medical College, Huazhong University of Science and Technology, Wuhan 430022, China.; (Zhang M) President's Office of Union Hospital, Tongji Medical College, Huazhong University of Science and Technology (HUST), Wuhan, China.; (Luo J) Department of Pharmacy, Union Hospital, Tongji Medical College, Huazhong University of Science and Technology, Wuhan 430022, China.; (Xie J) Department of Pharmacy, Union Hospital, Tongji Medical College, Huazhong University of Science and Technology, Wuhan 430022, China.; (Chen X) Department of Pharmacy, Union Hospital, Tongji Medical College, Huazhong University of Science and Technology, Wuhan 430022, China.; (Wang H) Department of Pharmacy, Union Hospital, Tongji Medical College, Huazhong University of Science and Technology, Wuhan 430022, China.; (Li S) Department of Pharmacy, Union Hospital, Tongji Medical College, Huazhong University of Science and Technology, Wuhan 430022, China.; (Yang S) Cancer Center, Union Hospital, Tongji Medical College, Huazhong University of Science and Technology, Wuhan, China.; (Peng C) Cancer Center, Union Hospital, Tongji Medical College, Huazhong University of Science and Technology, Wuhan, China.; (Yang L) Department of Occupational and Environmental Health, Ministry of Education and Ministry of Environmental Protection, and Key Laboratory of Environmental Health,

School of Public Health, Tongji Medical College, Huazhong University of Science and Technology, Wuhan, China.; (Deng B) Department of Pharmacy, Union Hospital, Tongji Medical College, Huazhong University of Science and Technology, Wuhan 430022, China.; (Zhang Y) Department of Pharmacy, Union Hospital, Tongji Medical College, Huazhong University of Science and Technology, Wuhan 430022, China.; (Wang C) Department of Pharmacy, Union Hospital, Tongji Medical College, Huazhong University of Science and Technology, Wuhan 430022, China.; (Hu J) Cancer Center, Union Hospital, Tongji Medical College, Huazhong University of Science and Technology, Wuhan, China.; (Shi C) Department of Pharmacy, Union Hospital, Tongji Medical College, Huazhong University of Science and Technology, Wuhan 430022, China.
(Liu J) Department of Pharmacy, Union Hospital, Tongji Medical College, Huazhong University of Science and Technology, Wuhan 430022, China.; (Zhang M) President's Office of Union Hospital, Tongji Medical College, Huazhong University of Science and Technology (HUST), Wuhan, China.; (Luo J) Department of Pharmacy, Union Hospital, Tongji Medical College, Huazhong University of Science and Technology, Wuhan 430022, China.; (Xie J) Department of Pharmacy, Union Hospital, Tongji Medical College, Huazhong University of Science and Technology, Wuhan 430022, China.; (Chen X) Department of Pharmacy, Union Hospital, Tongji Medical College, Huazhong University of Science and Technology, Wuhan 430022, China.; (Wang H) Department of Pharmacy, Union Hospital, Tongji Medical College, Huazhong University of Science and Technology, Wuhan 430022, China.; (Li S) Department of Pharmacy, Union Hospital, Tongji Medical College, Huazhong University of Science and Technology, Wuhan 430022, China.; (Yang S) Cancer Center, Union Hospital, Tongji Medical College, Huazhong University of Science and Technology, Wuhan, China.; (Peng C) Cancer Center, Union Hospital, Tongji Medical College, Huazhong University of Science and Technology, Wuhan, China.; (Yang L) Department of Occupational and Environmental Health, Ministry of Education and Ministry of Environmental Protection, and Key Laboratory of Environmental Health, School of Public Health, Tongji Medical College, Huazhong University of Science and Technology, Wuhan, China.; (Deng B) Department of Pharmacy, Union Hospital, Tongji Medical College, Huazhong University of Science and Technology, Wuhan 430022, China.; (Zhang Y) Department of Pharmacy, Union Hospital, Tongji Medical College, Huazhong University of Science and Technology, Wuhan 430022, China.; (Wang C) Department of Pharmacy, Union Hospital, Tongji Medical College, Huazhong University of Science and Technology, Wuhan 430022, China.; (Hu J) Cancer Center, Union Hospital, Tongji Medical College, Huazhong University of Science and Technology, Wuhan, China.; (Shi C) Department of Pharmacy, Union Hospital, Tongji Medical College, Huazhong University of Science and Technology, Wuhan 430022, China.
(Liu J) Department of Pharmacy, Union Hospital, Tongji Medical College, Huazhong University of Science and Technology, Wuhan 430022, China.; (Zhang M) President's Office of Union Hospital, Tongji Medical College, Huazhong University of Science and Technology (HUST), Wuhan, China.; (Luo J) Department of Pharmacy, Union Hospital, Tongji Medical College, Huazhong University of Science and Technology, Wuhan 430022, China.; (Xie J) Department of Pharmacy, Union Hospital, Tongji Medical College, Huazhong University of Science and Technology, Wuhan 430022, China.; (Chen X) Department of Pharmacy, Union Hospital, Tongji Medical College, Huazhong University of Science and Technology, Wuhan 430022, China.; (Wang H) Department of Pharmacy, Union Hospital, Tongji Medical College,

Huazhong University of Science and Technology, Wuhan 430022, China.; (Li S) Department of Pharmacy, Union Hospital, Tongji Medical College, Huazhong University of Science and Technology, Wuhan 430022, China.; (Yang S) Cancer Center, Union Hospital, Tongji Medical College, Huazhong University of Science and Technology, Wuhan, China.; (Peng C) Cancer Center, Union Hospital, Tongji Medical College, Huazhong University of Science and Technology, Wuhan, China.; (Yang L) Department of Occupational and Environmental Health, Ministry of Education and Ministry of Environmental Protection, and Key Laboratory of Environmental Health, School of Public Health, Tongji Medical College, Huazhong University of Science and Technology, Wuhan, China.; (Deng B) Department of Pharmacy, Union Hospital, Tongji Medical College, Huazhong University of Science and Technology, Wuhan 430022, China.; (Zhang Y) Department of Pharmacy, Union Hospital, Tongji Medical College, Huazhong University of Science and Technology, Wuhan 430022, China.; (Wang C) Department of Pharmacy, Union Hospital, Tongji Medical College, Huazhong University of Science and Technology, Wuhan 430022, China.; (Hu J) Cancer Center, Union Hospital, Tongji Medical College, Huazhong University of Science and Technology, Wuhan, China.; (Shi C) Department of Pharmacy, Union Hospital, Tongji Medical College, Huazhong University of Science and Technology, Wuhan 430022, China.
Database: PubMed

5. Effect of Physician-Pharmacist Participation in the Management of Ambulatory Cancer Pain Through a Digital Health Platform: Randomized Controlled Trial.

Author(s): Zhang L; McLeod HL; Liu KK; Liu WH; Huang HX; Huang YM; Sun SS; Chen XP; Chen Y; Liu FZ; Xiao J

Source: JMIR mHealth and uHealth; ; vol. 9 (no. 8); p. e24555

Publication Type(s): Journal Article

DOI: http://dx.doi.org/10.2196/24555

ISSN: 2291-5222

Place of Publication: Canada

PubMedID: 34398796

Accession Number: 34398796

Available at JMIR mHealth and uHealth - from Europe PubMed Central - Open Access

Available at JMIR mHealth and uHealth - from ProQuest (Health Research Premium) - NHS Version

Keywords: Subject Terms: *ambulatory setting; *cancer pain; *digital health; *physician-pharmacist; *self-management

Abstract:BACKGROUND: Self-management of ambulatory cancer pain is full of challenges. Motivated by the need for better pain management, we developed a WeChat-supported platform, Medication Housekeeper (MediHK), to enhance communication, optimize outcomes, and promote self-management in the home setting.OBJECTIVE: We conducted a randomized controlled trial to assess whether the joint physician-pharmacist team through MediHK would provide better self-management of ambulatory patients with cancer pain.METHODS: Patients were

randomly assigned to either an intervention group or control group. During the 4-week study period, the pharmacist would send 24-hour pain diaries daily, adverse drug reaction (ADR) forms every 3 days, and the Brief Pain Inventory form every 15 days to patients in the intervention group via MediHK. If a patient needed a change in drug/dosage or treatment of an ADR after the comprehensive review, the pharmacist would propose pharmacological interventions to the attending physician, who was then responsible for prescribing or adjusting pain medications. If no adjustments were needed, the pharmacist provided appropriate targeted education based on knowledge deficits. Patients in the control group received conventional care and did not receive reminders to fill out the forms. However, if the control group patients filled out a form via MediHK, the pain management team would review and respond in the same way as for the intervention group. The primary outcomes included pain intensity and pain interference in daily life. Secondary outcomes included patient-reported outcome measures, medication adherence, ADRs, and rehospitalization rates.RESULTS: A total of 100 patients were included, with 51 (51%) in the intervention group and 49 (49%) in the control group. The worst pain scores, least pain scores, and average pain scores in the intervention group and the control group were statistically different, with median values of 4 (IQR 3-7) vs 7 (IQR 6-8; P=.001), 1 (IQR 0-2) vs 2 (IQR 1-3; P=.02), and 2 (IQR 2-4) vs 4 (IQR 3-5; P=.001), respectively, at the end of the study. The pain interference on patients' general activity, mood, relationships with others, and interests was reduced, but the difference was not statistically significant compared with the control group (Ps=.10-.76). The medication adherence rate increased from 43% to 63% in the intervention group, compared with an increase of 33% to 51% in the control group (P<.001). The overall number of ADRs increased at 4 weeks, and more ADRs were monitored in the intervention group (P=.003). Rehospitalization rates were similar between the 2 groups.CONCLUSIONS: The joint physician-pharmacist team operating through MediHK improved pain management. This study supports the feasibility of integrating the internet into the self-management of cancer pain.TRIAL REGISTRATION: Chinese Clinical Trial Registry ChiCTR1900023075; https://www.chictr.org.cn/showproj.aspx?proj=36901.

Institutions:

(Zhang L) Department of Pharmacy, Xiangya Hospital, Central South University, Changsha, China.; (McLeod HL) Geriatric Oncology Consortium, Tampa, FL, United States.; (Liu KK) Department of Pharmacy, Xiangya Hospital, Central South University, Changsha, China.; (Liu WH) Department of Pharmacy, The Second Xiangya Hospital, Central South University, Changsha, China.; (Huang HX) Department of Pharmacy, Xiangya Hospital, Central South University, Changsha, China.; (Huang YM) Department of Pharmacy, Xiangya Hospital, Central South University, Changsha, China.; (Sun SS) Department of Pharmacy, Xiangya Hospital, Central South University, Changsha, China.; (Chen XP) Institute for Rational and Safe Medication Practices, National Clinical Research Center for Geriatric Disorders, Xiangya Hospital, Central South University, Changsha, China.; (Chen Y) Department of Clinical Pharmacology, Xiangya Hospital, Central South University, Changsha, China.; (Liu FZ) College of Information Science and Engineering, Hunan Normal University, Changsha, China.; (Xiao J) Department of Pharmacy, Xiangya Hospital, Central South University, Changsha, China.

(Zhang L) Department of Pharmacy, Xiangya Hospital, Central South University, Changsha, China.; (McLeod HL) Geriatric Oncology Consortium, Tampa, FL, United States.; (Liu KK) Department of Pharmacy, Xiangya Hospital, Central South

University, Changsha, China.; (Liu WH) Department of Pharmacy, The Second Xiangya Hospital, Central South University, Changsha, China.; (Huang HX) Department of Pharmacy, Xiangya Hospital, Central South University, Changsha, China.; (Huang YM) Department of Pharmacy, Xiangya Hospital, Central South University, Changsha, China.; (Sun SS) Department of Pharmacy, Xiangya Hospital, Central South University, Changsha, China.; (Chen XP) Institute for Rational and Safe Medication Practices, National Clinical Research Center for Geriatric Disorders, Xiangya Hospital, Central South University, Changsha, China.; (Chen Y) Department of Clinical Pharmacology, Xiangya Hospital, Central South University, Changsha, China.; (Liu FZ) College of Information Science and Engineering, Hunan Normal University, Changsha, China.; (Xiao J) Department of Pharmacy, Xiangya Hospital, Central South University, Changsha, China.
(Zhang L) Department of Pharmacy, Xiangya Hospital, Central South University, Changsha, China.; (McLeod HL) Geriatric Oncology Consortium, Tampa, FL, United States.; (Liu KK) Department of Pharmacy, Xiangya Hospital, Central South University, Changsha, China.; (Liu WH) Department of Pharmacy, The Second Xiangya Hospital, Central South University, Changsha, China.; (Huang HX) Department of Pharmacy, Xiangya Hospital, Central South University, Changsha, China.; (Huang YM) Department of Pharmacy, Xiangya Hospital, Central South University, Changsha, China.; (Sun SS) Department of Pharmacy, Xiangya Hospital, Central South University, Changsha, China.; (Chen XP) Institute for Rational and Safe Medication Practices, National Clinical Research Center for Geriatric Disorders, Xiangya Hospital, Central South University, Changsha, China.; (Chen Y) Department of Clinical Pharmacology, Xiangya Hospital, Central South University, Changsha, China.; (Liu FZ) College of Information Science and Engineering, Hunan Normal University, Changsha, China.; (Xiao J) Department of Pharmacy, Xiangya Hospital, Central South University, Changsha, China.
(Zhang L) Department of Pharmacy, Xiangya Hospital, Central South University, Changsha, China.; (McLeod HL) Geriatric Oncology Consortium, Tampa, FL, United States.; (Liu KK) Department of Pharmacy, Xiangya Hospital, Central South University, Changsha, China.; (Liu WH) Department of Pharmacy, The Second Xiangya Hospital, Central South University, Changsha, China.; (Huang HX) Department of Pharmacy, Xiangya Hospital, Central South University, Changsha, China.; (Huang YM) Department of Pharmacy, Xiangya Hospital, Central South University, Changsha, China.; (Sun SS) Department of Pharmacy, Xiangya Hospital, Central South University, Changsha, China.; (Chen XP) Institute for Rational and Safe Medication Practices, National Clinical Research Center for Geriatric Disorders, Xiangya Hospital, Central South University, Changsha, China.; (Chen Y) Department of Clinical Pharmacology, Xiangya Hospital, Central South University, Changsha, China.; (Liu FZ) College of Information Science and Engineering, Hunan Normal University, Changsha, China.; (Xiao J) Department of Pharmacy, Xiangya Hospital, Central South University, Changsha, China.
(Zhang L) Department of Pharmacy, Xiangya Hospital, Central South University, Changsha, China.; (McLeod HL) Geriatric Oncology Consortium, Tampa, FL, United States.; (Liu KK) Department of Pharmacy, Xiangya Hospital, Central South University, Changsha, China.; (Liu WH) Department of Pharmacy, The Second Xiangya Hospital, Central South University, Changsha, China.; (Huang HX) Department of Pharmacy, Xiangya Hospital, Central South University, Changsha, China.; (Huang YM) Department of Pharmacy, Xiangya Hospital, Central South University, Changsha, China.; (Sun SS) Department of Pharmacy, Xiangya Hospital,

Central South University, Changsha, China.; (Chen XP) Institute for Rational and Safe Medication Practices, National Clinical Research Center for Geriatric Disorders, Xiangya Hospital, Central South University, Changsha, China.; (Chen Y) Department of Clinical Pharmacology, Xiangya Hospital, Central South University, Changsha, China.; (Liu FZ) College of Information Science and Engineering, Hunan Normal University, Changsha, China.; (Xiao J) Department of Pharmacy, Xiangya Hospital, Central South University, Changsha, China.
(Zhang L) Department of Pharmacy, Xiangya Hospital, Central South University, Changsha, China.; (McLeod HL) Geriatric Oncology Consortium, Tampa, FL, United States.; (Liu KK) Department of Pharmacy, Xiangya Hospital, Central South University, Changsha, China.; (Liu WH) Department of Pharmacy, The Second Xiangya Hospital, Central South University, Changsha, China.; (Huang HX) Department of Pharmacy, Xiangya Hospital, Central South University, Changsha, China.; (Huang YM) Department of Pharmacy, Xiangya Hospital, Central South University, Changsha, China.; (Sun SS) Department of Pharmacy, Xiangya Hospital, Central South University, Changsha, China.; (Chen XP) Institute for Rational and Safe Medication Practices, National Clinical Research Center for Geriatric Disorders, Xiangya Hospital, Central South University, Changsha, China.; (Chen Y) Department of Clinical Pharmacology, Xiangya Hospital, Central South University, Changsha, China.; (Liu FZ) College of Information Science and Engineering, Hunan Normal University, Changsha, China.; (Xiao J) Department of Pharmacy, Xiangya Hospital, Central South University, Changsha, China.
(Zhang L) Department of Pharmacy, Xiangya Hospital, Central South University, Changsha, China.; (McLeod HL) Geriatric Oncology Consortium, Tampa, FL, United States.; (Liu KK) Department of Pharmacy, Xiangya Hospital, Central South University, Changsha, China.; (Liu WH) Department of Pharmacy, The Second Xiangya Hospital, Central South University, Changsha, China.; (Huang HX) Department of Pharmacy, Xiangya Hospital, Central South University, Changsha, China.; (Huang YM) Department of Pharmacy, Xiangya Hospital, Central South University, Changsha, China.; (Sun SS) Department of Pharmacy, Xiangya Hospital, Central South University, Changsha, China.; (Chen XP) Institute for Rational and Safe Medication Practices, National Clinical Research Center for Geriatric Disorders, Xiangya Hospital, Central South University, Changsha, China.; (Chen Y) Department of Clinical Pharmacology, Xiangya Hospital, Central South University, Changsha, China.; (Liu FZ) College of Information Science and Engineering, Hunan Normal University, Changsha, China.; (Xiao J) Department of Pharmacy, Xiangya Hospital, Central South University, Changsha, China.
(Zhang L) Department of Pharmacy, Xiangya Hospital, Central South University, Changsha, China.; (McLeod HL) Geriatric Oncology Consortium, Tampa, FL, United States.; (Liu KK) Department of Pharmacy, Xiangya Hospital, Central South University, Changsha, China.; (Liu WH) Department of Pharmacy, The Second Xiangya Hospital, Central South University, Changsha, China.; (Huang HX) Department of Pharmacy, Xiangya Hospital, Central South University, Changsha, China.; (Huang YM) Department of Pharmacy, Xiangya Hospital, Central South University, Changsha, China.; (Sun SS) Department of Pharmacy, Xiangya Hospital, Central South University, Changsha, China.; (Chen XP) Institute for Rational and Safe Medication Practices, National Clinical Research Center for Geriatric Disorders, Xiangya Hospital, Central South University, Changsha, China.; (Chen Y) Department of Clinical Pharmacology, Xiangya Hospital, Central South University, Changsha, China.; (Liu FZ) College of Information Science and Engineering, Hunan Normal

University, Changsha, China.; (Xiao J) Department of Pharmacy, Xiangya Hospital, Central South University, Changsha, China.
(Zhang L) Department of Pharmacy, Xiangya Hospital, Central South University, Changsha, China.; (McLeod HL) Geriatric Oncology Consortium, Tampa, FL, United States.; (Liu KK) Department of Pharmacy, Xiangya Hospital, Central South University, Changsha, China.; (Liu WH) Department of Pharmacy, The Second Xiangya Hospital, Central South University, Changsha, China.; (Huang HX) Department of Pharmacy, Xiangya Hospital, Central South University, Changsha, China.; (Huang YM) Department of Pharmacy, Xiangya Hospital, Central South University, Changsha, China.; (Sun SS) Department of Pharmacy, Xiangya Hospital, Central South University, Changsha, China.; (Chen XP) Institute for Rational and Safe Medication Practices, National Clinical Research Center for Geriatric Disorders, Xiangya Hospital, Central South University, Changsha, China.; (Chen Y) Department of Clinical Pharmacology, Xiangya Hospital, Central South University, Changsha, China.; (Liu FZ) College of Information Science and Engineering, Hunan Normal University, Changsha, China.; (Xiao J) Department of Pharmacy, Xiangya Hospital, Central South University, Changsha, China.
(Zhang L) Department of Pharmacy, Xiangya Hospital, Central South University, Changsha, China.; (McLeod HL) Geriatric Oncology Consortium, Tampa, FL, United States.; (Liu KK) Department of Pharmacy, Xiangya Hospital, Central South University, Changsha, China.; (Liu WH) Department of Pharmacy, The Second Xiangya Hospital, Central South University, Changsha, China.; (Huang HX) Department of Pharmacy, Xiangya Hospital, Central South University, Changsha, China.; (Huang YM) Department of Pharmacy, Xiangya Hospital, Central South University, Changsha, China.; (Sun SS) Department of Pharmacy, Xiangya Hospital, Central South University, Changsha, China.; (Chen XP) Institute for Rational and Safe Medication Practices, National Clinical Research Center for Geriatric Disorders, Xiangya Hospital, Central South University, Changsha, China.; (Chen Y) Department of Clinical Pharmacology, Xiangya Hospital, Central South University, Changsha, China.; (Liu FZ) College of Information Science and Engineering, Hunan Normal University, Changsha, China.; (Xiao J) Department of Pharmacy, Xiangya Hospital, Central South University, Changsha, China.
(Zhang L) Department of Pharmacy, Xiangya Hospital, Central South University, Changsha, China.; (McLeod HL) Geriatric Oncology Consortium, Tampa, FL, United States.; (Liu KK) Department of Pharmacy, Xiangya Hospital, Central South University, Changsha, China.; (Liu WH) Department of Pharmacy, The Second Xiangya Hospital, Central South University, Changsha, China.; (Huang HX) Department of Pharmacy, Xiangya Hospital, Central South University, Changsha, China.; (Huang YM) Department of Pharmacy, Xiangya Hospital, Central South University, Changsha, China.; (Sun SS) Department of Pharmacy, Xiangya Hospital, Central South University, Changsha, China.; (Chen XP) Institute for Rational and Safe Medication Practices, National Clinical Research Center for Geriatric Disorders, Xiangya Hospital, Central South University, Changsha, China.; (Chen Y) Department of Clinical Pharmacology, Xiangya Hospital, Central South University, Changsha, China.; (Liu FZ) College of Information Science and Engineering, Hunan Normal University, Changsha, China.; (Xiao J) Department of Pharmacy, Xiangya Hospital, Central South University, Changsha, China.
Database: PubMed

6. [History and Present of Stereotactic and Functional Neurosurgery].

Author(s): Taira T

Source: No shinkei geka. Neurological surgery; Jul 2021; vol. 49 (no. 4); p. 698-710

Publication Date: Jul 2021

Publication Type(s): Journal Article

DOI: http://dx.doi.org/10.11477/mf.1436204448

ISSN: 0301-2603

Place of Publication: Japan

PubMedID: 34376602

Accession Number: 34376602

Abstract: Stereotactic and functional neurosurgery(SFN)is one of the oldest subspecialties of neurosurgery. In Japan, functional epilepsy surgery was performed in the Meiji era, even before World War II, when general surgeons used to operate on patients with intractable cancer pain through open myelotomy or cordotomy. Knowledge gathered from such age-old procedures provided the basic understanding of neurophysiology and hence, functional neurosurgery used to be termed as "applied neurophysiology". Human stereotactic surgery was introduced in 1947 and many Japanese neurosurgeons, particularly Hirotaro Narabayashi, Keiji Sano and Chihiro Ohye, contributed to the development of this field. Additionally, we should remember that common procedures of neurosurgery practiced today such as neuroendoscopy, navigation surgery, intraoperative monitoring, and the concept of exo-scope, which are less invasive and involves high accuracy, emerged from the field of SFN. Young doctors should learn from history and understand where we started from, where we at present, and where we are heading in the future.

Institutions:
(Taira T) Department of Neurosurgery, Tokyo Women's Medical University.
Database: PubMed

7. Barriers and facilitators encountered by family physicians prescribing opioids for chronic non-cancer pain: a qualitative study.

Author(s): Goodwin J; Kirkland S

Source: Health promotion and chronic disease prevention in Canada : research, policy and practice; Jun 2021; vol. 41 (no. 6); p. 182-189

Publication Date: Jun 2021

Publication Type(s): Journal Article

DOI: http://dx.doi.org/10.24095/hpcdp.41.6.03

ISSN: 2368-738X

Place of Publication: Canada

PubMedID: 34164970

Accession Number: 34164970

Available at Health promotion and chronic disease prevention in Canada : research, policy and practice - from Europe PubMed Central - Open Access

Available at Health promotion and chronic disease prevention in Canada : research, policy and practice - from EBSCO (MEDLINE Complete)

Available at Health promotion and chronic disease prevention in Canada : research, policy and practice - from Unpaywall

Keywords: Subject Terms: addiction; chronic pain; family physicians; opioid

Abstract:INTRODUCTION: Harms caused by prescription opioid analgesics (POAs) have been identified as a major international public health concern. Recent statistics show rising numbers of opioid-related deaths across Canada. However, Canadian family physicians appear to have inadequate resources to safely and effectively prescribe opioid analgesics to treat chronic non-cancer pain (CNCP).METHODS: We completed a qualitative study of the barriers and facilitators to safe and effective prescribing of opioid analgesics for CNCP through semi-structured interviews with eight family physicians in Nova Scotia. Thematic analysis was used to identify the barriers and facilitators.RESULTS: Family physicians identified challenges in prescribing opioid analgesics for CNCP: the complexity of CNCP management, addictions risks and prescribing tools, physician training, the physician-patient relationship, prescription monitoring and control, and systemic factors.CONCLUSION: Family physicians described themselves as inadequately supported in their prescribing of opioid analgesics for CNCP and could benefit from an integrated and coordinated approach to prescriber support.

Institutions:
(Goodwin J) Department of Medicine, Dalhousie University, Halifax, Nova Scotia, Canada.; (Kirkland S) Department of Medicine, Dalhousie University, Halifax, Nova Scotia, Canada.
(Goodwin J) Department of Medicine, Dalhousie University, Halifax, Nova Scotia, Canada.; (Kirkland S) Department of Medicine, Dalhousie University, Halifax, Nova Scotia, Canada.
Database: PubMed

8. Effect of a theory-driven educational intervention on the level of knowledge, attitudes, and assessment practices regarding breakthrough cancer pain (BTCP) management among medical nurses in Hong Kong.

Author(s): Kwok CYL; Chan DNS; So WKW

Source: European journal of oncology nursing : the official journal of European Oncology Nursing Society; Jun 2021; vol. 52 ; p. 101945

Publication Date: Jun 2021

Publication Type(s): Journal Article

DOI: http://dx.doi.org/10.1016/j.ejon.2021.101945

ISSN: 1532-2122

Place of Publication: Scotland

PubMedID: 33813183

Accession Number: 33813183

Available at European journal of oncology nursing : the official journal of European Oncology Nursing Society - from ScienceDirect

Keywords: Subject Terms: Assessment; Attitudes; Breakthrough cancer pain; Education; Knowledge; Nurses; Practices

Abstract:PURPOSE: To examine the effect of an educational intervention intended to improve medical nurses' adherence to breakthrough cancer pain (BTCP) assessment practices and their level of knowledge, attitudes and perceived assessment practices regarding BTCP management.METHODS: Nurses working in a regional hospital were recruited to this quasi-experimental study. The intervention group received a 3-h educational workshop and one session of individual clinical instruction, whilst the control group did not receive any intervention. Chart audits were performed to examine adherence to BTCP assessment practices as the primary outcome. A self-developed questionnaire was used to measure nurses' knowledge, attitudes and perceived assessment practices regarding BTCP management as the secondary outcomes. The chi-square or Fisher's exact test was used to compare the rate of adherence to BTCP assessment practices between groups. A generalised estimating equation was used to compare changes in knowledge, attitudes, and perceived assessment practices between groups over time.RESULTS: One hundred and five nurses completed the study. The chart audits revealed a significantly higher rate of adherence to BTCP assessment practices in the intervention group after the intervention ($p < .05$). The intervention group exhibited significant positive changes in scores for knowledge ($\beta = 25.49$, $p < .001$), attitude ($\beta = 0.98$ to 2.81, $p < .01$), and their perceived assessment practices ($\beta = 1.33$ to 3.14, $p < .002$) when compared with the control group.CONCLUSIONS: This theory-driven educational intervention significantly improved the medical nurses' adherence to BTCP assessment practices and their level of knowledge attitudes and perceived assessment practices regarding BTCP management.

Institutions:
(Kwok CYL) Haven of Hope Sister Annie Skau Holistic Centre, Hong Kong, SAR, China.; (Chan DNS) The Nethersole School of Nursing, The Chinese University of Hong Kong, Hong Kong, SAR, China. Electronic address: dorothycns@cuhk.edu.hk.; (So WKW) The Nethersole School of Nursing, The Chinese University of Hong Kong, Hong Kong, SAR, China.
(Kwok CYL) Haven of Hope Sister Annie Skau Holistic Centre, Hong Kong, SAR, China.; (Chan DNS) The Nethersole School of Nursing, The Chinese University of Hong Kong, Hong Kong, SAR, China. Electronic address: dorothycns@cuhk.edu.hk.; (So WKW) The Nethersole School of Nursing, The Chinese University of Hong Kong, Hong Kong, SAR, China.
(Kwok CYL) Haven of Hope Sister Annie Skau Holistic Centre, Hong Kong, SAR, China.; (Chan DNS) The Nethersole School of Nursing, The Chinese University of Hong Kong, Hong Kong, SAR, China. Electronic address: dorothycns@cuhk.edu.hk.; (So WKW) The Nethersole School of Nursing, The Chinese University of Hong Kong, Hong Kong, SAR, China.

Database: PubMed

9. European clinical practice recommendations on opioids for chronic noncancer pain - Part 2: Special situations.

Author(s): Krčevski Škvarč N; Morlion B; Vowles KE; Bannister K; Buchsner E; Casale R; Chenot JF; Chumbley G; Drewes AM; Dom G; Jutila L; O'Brien T; Pogatzki-Zahn E; Rakusa M; Suarez-Serrano C; Tölle T; Häuser W

Source: European journal of pain (London, England); 2021; vol. 25 (no. 5); p. 969-985

Publication Date: 2021

Publication Type(s): Journal Article

DOI: http://dx.doi.org/10.1002/ejp.1744

ISSN: 1532-2149

Place of Publication: England

PubMedID: 33655678

Accession Number: 33655678

Available at European journal of pain (London, England) - from Wiley Online Library Medicine and Nursing Collection 2020

Available at European journal of pain (London, England) - from Unpaywall

Abstract:BACKGROUND: Opioid use for chronic non-cancer pain (CNCP) is under debate. In the absence of pan-European guidance on this issue, a position paper was commissioned by the European Pain Federation (EFIC).METHODS: The clinical practice recommendations were developed by eight scientific societies and one patient self-help organization under the coordination of EFIC. A systematic literature search in MEDLINE (up until January 2020) was performed. Two categories of guidance are given: Evidence-based recommendations (supported by evidence from systematic reviews of randomized controlled trials or of observational studies) and Good Clinical Practice (GCP) statements (supported either by indirect evidence or by case-series, case-control studies and clinical experience). The GRADE system was applied to move from evidence to recommendations. The recommendations and GCP statements were developed by a multiprofessional task force (including nursing, service users, physicians, physiotherapy and psychology) and formal multistep procedures to reach a set of consensus recommendations. The clinical practice recommendations were reviewed by five external reviewers from North America and Europe and were also posted for public comment.RESULTS: The European Clinical Practice Recommendations give guidance for combination with other medications, the management of frequent (e.g. nausea, constipation) and rare (e.g. hyperalgesia) side effects, for special clinical populations (e.g. children and adolescents, pregnancy) and for special situations (e.g. liver cirrhosis).CONCLUSION: If a trial with opioids for chronic noncancer pain is conducted, detailed knowledge and experience are needed to adapt the opioid treatment to a special patient group and/or clinical situation and to manage side effects effectively.SIGNIFICANCE: If a trial with opioids for chronic noncancer pain is conducted, detailed knowledge and experience are needed to adapt the opioid treatment to a special patient group and/or clinical situation and to manage side effects effectively. A collaboration of medical specialties and of all health care professionals is needed for some special populations and clinical situations.

Institutions:
(Krčevski Škvarč N) Department of Anesthesiology, Intensive Care and Pain Treatment, Faculty of Medicine of University Maribor, Maribor, Slovenia.; (Morlion B) Center for Algology & Pain Management, University Hospitals Leuven, Leuven, Belgium.; (Vowles KE) School of Psychology, Queen's University Belfast, Belfast, UK.; (Bannister K) Institute of Psychiatry, Psychology and Neuroscience, King's College London, London, UK.; (Buchsner E) Pain Management and

Neuromodulation Centre EHC Hospital, Morges, Switzerland.; (Casale R) Neurorehabilitation Unit, Department of Rehabilitation, HABILITA, Bergamo, Italy.; (Chenot JF) Department of General Practice, Institute for Community Medicine, University Medicine Greifswald, Greifswald, Germany.; (Chumbley G) Imperial College Healthcare NHS Trust, Charing Cross Hospital, London, UK.; (Drewes AM) Mech-Sense, Department of Gastroenterology & Hepatology, Aalborg University Hospital, Aalborg, Denmark.; (Dom G) Collaborative Antwerp Psychiatric Research Institute (CAPRI), Antwerp University (UA), Antwerp, Belgium.; (Jutila L) Pain Alliance Europe, Finland.; (O'Brien T) College of Medicine & Health, University College Cork, Cork, Republic of Ireland.; (Pogatzki-Zahn E) Department of Anaesthesiology, Intensive Care and Pain Medicine, University Hospital Münster UKM, Munster, Germany.; (Rakusa M) Department of Neurology, University Medical Centre Maribor, Maribor, Slovenia.; (Suarez-Serrano C) Department of Physiotherapy, University of Sevilla, Sevilla, Spain.; (Tölle T) Department of Neurology, Techhnische Universität München, München, Germany.; (Häuser W) Department Internal Medicine 1, Saarbrücken, Germany.
(Krčevski Škvarč N) Department of Anesthesiology, Intensive Care and Pain Treatment, Faculty of Medicine of University Maribor, Maribor, Slovenia.; (Morlion B) Center for Algology & Pain Management, University Hospitals Leuven, Leuven, Belgium.; (Vowles KE) School of Psychology, Queen's University Belfast, Belfast, UK.; (Bannister K) Institute of Psychiatry, Psychology and Neuroscience, King's College London, London, UK.; (Buchsner E) Pain Management and Neuromodulation Centre EHC Hospital, Morges, Switzerland.; (Casale R) Neurorehabilitation Unit, Department of Rehabilitation, HABILITA, Bergamo, Italy.; (Chenot JF) Department of General Practice, Institute for Community Medicine, University Medicine Greifswald, Greifswald, Germany.; (Chumbley G) Imperial College Healthcare NHS Trust, Charing Cross Hospital, London, UK.; (Drewes AM) Mech-Sense, Department of Gastroenterology & Hepatology, Aalborg University Hospital, Aalborg, Denmark.; (Dom G) Collaborative Antwerp Psychiatric Research Institute (CAPRI), Antwerp University (UA), Antwerp, Belgium.; (Jutila L) Pain Alliance Europe, Finland.; (O'Brien T) College of Medicine & Health, University College Cork, Cork, Republic of Ireland.; (Pogatzki-Zahn E) Department of Anaesthesiology, Intensive Care and Pain Medicine, University Hospital Münster UKM, Munster, Germany.; (Rakusa M) Department of Neurology, University Medical Centre Maribor, Maribor, Slovenia.; (Suarez-Serrano C) Department of Physiotherapy, University of Sevilla, Sevilla, Spain.; (Tölle T) Department of Neurology, Techhnische Universität München, München, Germany.; (Häuser W) Department Internal Medicine 1, Saarbrücken, Germany.
(Krčevski Škvarč N) Department of Anesthesiology, Intensive Care and Pain Treatment, Faculty of Medicine of University Maribor, Maribor, Slovenia.; (Morlion B) Center for Algology & Pain Management, University Hospitals Leuven, Leuven, Belgium.; (Vowles KE) School of Psychology, Queen's University Belfast, Belfast, UK.; (Bannister K) Institute of Psychiatry, Psychology and Neuroscience, King's College London, London, UK.; (Buchsner E) Pain Management and Neuromodulation Centre EHC Hospital, Morges, Switzerland.; (Casale R) Neurorehabilitation Unit, Department of Rehabilitation, HABILITA, Bergamo, Italy.; (Chenot JF) Department of General Practice, Institute for Community Medicine, University Medicine Greifswald, Greifswald, Germany.; (Chumbley G) Imperial College Healthcare NHS Trust, Charing Cross Hospital, London, UK.; (Drewes AM) Mech-Sense, Department of Gastroenterology & Hepatology, Aalborg University

Hospital, Aalborg, Denmark.; (Dom G) Collaborative Antwerp Psychiatric Research Institute (CAPRI), Antwerp University (UA), Antwerp, Belgium.; (Jutila L) Pain Alliance Europe, Finland.; (O'Brien T) College of Medicine & Health, University College Cork, Cork, Republic of Ireland.; (Pogatzki-Zahn E) Department of Anaesthesiology, Intensive Care and Pain Medicine, University Hospital Münster UKM, Munster, Germany.; (Rakusa M) Department of Neurology, University Medical Centre Maribor, Maribor, Slovenia.; (Suarez-Serrano C) Department of Physiotherapy, University of Sevilla, Sevilla, Spain.; (Tölle T) Department of Neurology, Techhnische Universität München, München, Germany.; (Häuser W) Department Internal Medicine 1, Saarbrücken, Germany.

(Krčevski Škvarč N) Department of Anesthesiology, Intensive Care and Pain Treatment, Faculty of Medicine of University Maribor, Maribor, Slovenia.; (Morlion B) Center for Algology & Pain Management, University Hospitals Leuven, Leuven, Belgium.; (Vowles KE) School of Psychology, Queen's University Belfast, Belfast, UK.; (Bannister K) Institute of Psychiatry, Psychology and Neuroscience, King's College London, London, UK.; (Buchsner E) Pain Management and Neuromodulation Centre EHC Hospital, Morges, Switzerland.; (Casale R) Neurorehabilitation Unit, Department of Rehabilitation, HABILITA, Bergamo, Italy.; (Chenot JF) Department of General Practice, Institute for Community Medicine, University Medicine Greifswald, Greifswald, Germany.; (Chumbley G) Imperial College Healthcare NHS Trust, Charing Cross Hospital, London, UK.; (Drewes AM) Mech-Sense, Department of Gastroenterology & Hepatology, Aalborg University Hospital, Aalborg, Denmark.; (Dom G) Collaborative Antwerp Psychiatric Research Institute (CAPRI), Antwerp University (UA), Antwerp, Belgium.; (Jutila L) Pain Alliance Europe, Finland.; (O'Brien T) College of Medicine & Health, University College Cork, Cork, Republic of Ireland.; (Pogatzki-Zahn E) Department of Anaesthesiology, Intensive Care and Pain Medicine, University Hospital Münster UKM, Munster, Germany.; (Rakusa M) Department of Neurology, University Medical Centre Maribor, Maribor, Slovenia.; (Suarez-Serrano C) Department of Physiotherapy, University of Sevilla, Sevilla, Spain.; (Tölle T) Department of Neurology, Techhnische Universität München, München, Germany.; (Häuser W) Department Internal Medicine 1, Saarbrücken, Germany.

(Krčevski Škvarč N) Department of Anesthesiology, Intensive Care and Pain Treatment, Faculty of Medicine of University Maribor, Maribor, Slovenia.; (Morlion B) Center for Algology & Pain Management, University Hospitals Leuven, Leuven, Belgium.; (Vowles KE) School of Psychology, Queen's University Belfast, Belfast, UK.; (Bannister K) Institute of Psychiatry, Psychology and Neuroscience, King's College London, London, UK.; (Buchsner E) Pain Management and Neuromodulation Centre EHC Hospital, Morges, Switzerland.; (Casale R) Neurorehabilitation Unit, Department of Rehabilitation, HABILITA, Bergamo, Italy.; (Chenot JF) Department of General Practice, Institute for Community Medicine, University Medicine Greifswald, Greifswald, Germany.; (Chumbley G) Imperial College Healthcare NHS Trust, Charing Cross Hospital, London, UK.; (Drewes AM) Mech-Sense, Department of Gastroenterology & Hepatology, Aalborg University Hospital, Aalborg, Denmark.; (Dom G) Collaborative Antwerp Psychiatric Research Institute (CAPRI), Antwerp University (UA), Antwerp, Belgium.; (Jutila L) Pain Alliance Europe, Finland.; (O'Brien T) College of Medicine & Health, University College Cork, Cork, Republic of Ireland.; (Pogatzki-Zahn E) Department of Anaesthesiology, Intensive Care and Pain Medicine, University Hospital Münster UKM, Munster, Germany.; (Rakusa M) Department of Neurology, University Medical

Centre Maribor, Maribor, Slovenia.; (Suarez-Serrano C) Department of Physiotherapy, University of Sevilla, Sevilla, Spain.; (Tölle T) Department of Neurology, Techhnische Universität München, München, Germany.; (Häuser W) Department Internal Medicine 1, Saarbrücken, Germany.
(Krčevski Škvarč N) Department of Anesthesiology, Intensive Care and Pain Treatment, Faculty of Medicine of University Maribor, Maribor, Slovenia.; (Morlion B) Center for Algology & Pain Management, University Hospitals Leuven, Leuven, Belgium.; (Vowles KE) School of Psychology, Queen's University Belfast, Belfast, UK.; (Bannister K) Institute of Psychiatry, Psychology and Neuroscience, King's College London, London, UK.; (Buchsner E) Pain Management and Neuromodulation Centre EHC Hospital, Morges, Switzerland.; (Casale R) Neurorehabilitation Unit, Department of Rehabilitation, HABILITA, Bergamo, Italy.; (Chenot JF) Department of General Practice, Institute for Community Medicine, University Medicine Greifswald, Greifswald, Germany.; (Chumbley G) Imperial College Healthcare NHS Trust, Charing Cross Hospital, London, UK.; (Drewes AM) Mech-Sense, Department of Gastroenterology & Hepatology, Aalborg University Hospital, Aalborg, Denmark.; (Dom G) Collaborative Antwerp Psychiatric Research Institute (CAPRI), Antwerp University (UA), Antwerp, Belgium.; (Jutila L) Pain Alliance Europe, Finland.; (O'Brien T) College of Medicine & Health, University College Cork, Cork, Republic of Ireland.; (Pogatzki-Zahn E) Department of Anaesthesiology, Intensive Care and Pain Medicine, University Hospital Münster UKM, Munster, Germany.; (Rakusa M) Department of Neurology, University Medical Centre Maribor, Maribor, Slovenia.; (Suarez-Serrano C) Department of Physiotherapy, University of Sevilla, Sevilla, Spain.; (Tölle T) Department of Neurology, Techhnische Universität München, München, Germany.; (Häuser W) Department Internal Medicine 1, Saarbrücken, Germany.
(Krčevski Škvarč N) Department of Anesthesiology, Intensive Care and Pain Treatment, Faculty of Medicine of University Maribor, Maribor, Slovenia.; (Morlion B) Center for Algology & Pain Management, University Hospitals Leuven, Leuven, Belgium.; (Vowles KE) School of Psychology, Queen's University Belfast, Belfast, UK.; (Bannister K) Institute of Psychiatry, Psychology and Neuroscience, King's College London, London, UK.; (Buchsner E) Pain Management and Neuromodulation Centre EHC Hospital, Morges, Switzerland.; (Casale R) Neurorehabilitation Unit, Department of Rehabilitation, HABILITA, Bergamo, Italy.; (Chenot JF) Department of General Practice, Institute for Community Medicine, University Medicine Greifswald, Greifswald, Germany.; (Chumbley G) Imperial College Healthcare NHS Trust, Charing Cross Hospital, London, UK.; (Drewes AM) Mech-Sense, Department of Gastroenterology & Hepatology, Aalborg University Hospital, Aalborg, Denmark.; (Dom G) Collaborative Antwerp Psychiatric Research Institute (CAPRI), Antwerp University (UA), Antwerp, Belgium.; (Jutila L) Pain Alliance Europe, Finland.; (O'Brien T) College of Medicine & Health, University College Cork, Cork, Republic of Ireland.; (Pogatzki-Zahn E) Department of Anaesthesiology, Intensive Care and Pain Medicine, University Hospital Münster UKM, Munster, Germany.; (Rakusa M) Department of Neurology, University Medical Centre Maribor, Maribor, Slovenia.; (Suarez-Serrano C) Department of Physiotherapy, University of Sevilla, Sevilla, Spain.; (Tölle T) Department of Neurology, Techhnische Universität München, München, Germany.; (Häuser W) Department Internal Medicine 1, Saarbrücken, Germany.
(Krčevski Škvarč N) Department of Anesthesiology, Intensive Care and Pain Treatment, Faculty of Medicine of University Maribor, Maribor, Slovenia.; (Morlion B)

Center for Algology & Pain Management, University Hospitals Leuven, Leuven, Belgium.; (Vowles KE) School of Psychology, Queen's University Belfast, Belfast, UK.; (Bannister K) Institute of Psychiatry, Psychology and Neuroscience, King's College London, London, UK.; (Buchsner E) Pain Management and Neuromodulation Centre EHC Hospital, Morges, Switzerland.; (Casale R) Neurorehabilitation Unit, Department of Rehabilitation, HABILITA, Bergamo, Italy.; (Chenot JF) Department of General Practice, Institute for Community Medicine, University Medicine Greifswald, Greifswald, Germany.; (Chumbley G) Imperial College Healthcare NHS Trust, Charing Cross Hospital, London, UK.; (Drewes AM) Mech-Sense, Department of Gastroenterology & Hepatology, Aalborg University Hospital, Aalborg, Denmark.; (Dom G) Collaborative Antwerp Psychiatric Research Institute (CAPRI), Antwerp University (UA), Antwerp, Belgium.; (Jutila L) Pain Alliance Europe, Finland.; (O'Brien T) College of Medicine & Health, University College Cork, Cork, Republic of Ireland.; (Pogatzki-Zahn E) Department of Anaesthesiology, Intensive Care and Pain Medicine, University Hospital Münster UKM, Munster, Germany.; (Rakusa M) Department of Neurology, University Medical Centre Maribor, Maribor, Slovenia.; (Suarez-Serrano C) Department of Physiotherapy, University of Sevilla, Sevilla, Spain.; (Tölle T) Department of Neurology, Techhnische Universität München, München, Germany.; (Häuser W) Department Internal Medicine 1, Saarbrücken, Germany.
(Krčevski Škvarč N) Department of Anesthesiology, Intensive Care and Pain Treatment, Faculty of Medicine of University Maribor, Maribor, Slovenia.; (Morlion B)
Center for Algology & Pain Management, University Hospitals Leuven, Leuven, Belgium.; (Vowles KE) School of Psychology, Queen's University Belfast, Belfast, UK.; (Bannister K) Institute of Psychiatry, Psychology and Neuroscience, King's College London, London, UK.; (Buchsner E) Pain Management and Neuromodulation Centre EHC Hospital, Morges, Switzerland.; (Casale R) Neurorehabilitation Unit, Department of Rehabilitation, HABILITA, Bergamo, Italy.; (Chenot JF) Department of General Practice, Institute for Community Medicine, University Medicine Greifswald, Greifswald, Germany.; (Chumbley G) Imperial College Healthcare NHS Trust, Charing Cross Hospital, London, UK.; (Drewes AM) Mech-Sense, Department of Gastroenterology & Hepatology, Aalborg University Hospital, Aalborg, Denmark.; (Dom G) Collaborative Antwerp Psychiatric Research Institute (CAPRI), Antwerp University (UA), Antwerp, Belgium.; (Jutila L) Pain Alliance Europe, Finland.; (O'Brien T) College of Medicine & Health, University College Cork, Cork, Republic of Ireland.; (Pogatzki-Zahn E) Department of Anaesthesiology, Intensive Care and Pain Medicine, University Hospital Münster UKM, Munster, Germany.; (Rakusa M) Department of Neurology, University Medical Centre Maribor, Maribor, Slovenia.; (Suarez-Serrano C) Department of Physiotherapy, University of Sevilla, Sevilla, Spain.; (Tölle T) Department of Neurology, Techhnische Universität München, München, Germany.; (Häuser W) Department Internal Medicine 1, Saarbrücken, Germany.
(Krčevski Škvarč N) Department of Anesthesiology, Intensive Care and Pain Treatment, Faculty of Medicine of University Maribor, Maribor, Slovenia.; (Morlion B) Center for Algology & Pain Management, University Hospitals Leuven, Leuven, Belgium.; (Vowles KE) School of Psychology, Queen's University Belfast, Belfast, UK.; (Bannister K) Institute of Psychiatry, Psychology and Neuroscience, King's College London, London, UK.; (Buchsner E) Pain Management and Neuromodulation Centre EHC Hospital, Morges, Switzerland.; (Casale R) Neurorehabilitation Unit, Department of Rehabilitation, HABILITA, Bergamo, Italy.;

(Chenot JF) Department of General Practice, Institute for Community Medicine, University Medicine Greifswald, Greifswald, Germany.; (Chumbley G) Imperial College Healthcare NHS Trust, Charing Cross Hospital, London, UK.; (Drewes AM) Mech-Sense, Department of Gastroenterology & Hepatology, Aalborg University Hospital, Aalborg, Denmark.; (Dom G) Collaborative Antwerp Psychiatric Research Institute (CAPRI), Antwerp University (UA), Antwerp, Belgium.; (Jutila L) Pain Alliance Europe, Finland.; (O'Brien T) College of Medicine & Health, University College Cork, Cork, Republic of Ireland.; (Pogatzki-Zahn E) Department of Anaesthesiology, Intensive Care and Pain Medicine, University Hospital Münster UKM, Munster, Germany.; (Rakusa M) Department of Neurology, University Medical Centre Maribor, Maribor, Slovenia.; (Suarez-Serrano C) Department of Physiotherapy, University of Sevilla, Sevilla, Spain.; (Tölle T) Department of Neurology, Techhnische Universität München, München, Germany.; (Häuser W) Department Internal Medicine 1, Saarbrücken, Germany.
(Krčevski Škvarč N) Department of Anesthesiology, Intensive Care and Pain Treatment, Faculty of Medicine of University Maribor, Maribor, Slovenia.; (Morlion B) Center for Algology & Pain Management, University Hospitals Leuven, Leuven, Belgium.; (Vowles KE) School of Psychology, Queen's University Belfast, Belfast, UK.; (Bannister K) Institute of Psychiatry, Psychology and Neuroscience, King's College London, London, UK.; (Buchsner E) Pain Management and Neuromodulation Centre EHC Hospital, Morges, Switzerland.; (Casale R) Neurorehabilitation Unit, Department of Rehabilitation, HABILITA, Bergamo, Italy.; (Chenot JF) Department of General Practice, Institute for Community Medicine, University Medicine Greifswald, Greifswald, Germany.; (Chumbley G) Imperial College Healthcare NHS Trust, Charing Cross Hospital, London, UK.; (Drewes AM) Mech-Sense, Department of Gastroenterology & Hepatology, Aalborg University Hospital, Aalborg, Denmark.; (Dom G) Collaborative Antwerp Psychiatric Research Institute (CAPRI), Antwerp University (UA), Antwerp, Belgium.; (Jutila L) Pain Alliance Europe, Finland.; (O'Brien T) College of Medicine & Health, University College Cork, Cork, Republic of Ireland.; (Pogatzki-Zahn E) Department of Anaesthesiology, Intensive Care and Pain Medicine, University Hospital Münster UKM, Munster, Germany.; (Rakusa M) Department of Neurology, University Medical Centre Maribor, Maribor, Slovenia.; (Suarez-Serrano C) Department of Physiotherapy, University of Sevilla, Sevilla, Spain.; (Tölle T) Department of Neurology, Techhnische Universität München, München, Germany.; (Häuser W) Department Internal Medicine 1, Saarbrücken, Germany.
(Krčevski Škvarč N) Department of Anesthesiology, Intensive Care and Pain Treatment, Faculty of Medicine of University Maribor, Maribor, Slovenia.; (Morlion B) Center for Algology & Pain Management, University Hospitals Leuven, Leuven, Belgium.; (Vowles KE) School of Psychology, Queen's University Belfast, Belfast, UK.; (Bannister K) Institute of Psychiatry, Psychology and Neuroscience, King's College London, London, UK.; (Buchsner E) Pain Management and Neuromodulation Centre EHC Hospital, Morges, Switzerland.; (Casale R) Neurorehabilitation Unit, Department of Rehabilitation, HABILITA, Bergamo, Italy.; (Chenot JF) Department of General Practice, Institute for Community Medicine, University Medicine Greifswald, Greifswald, Germany.; (Chumbley G) Imperial College Healthcare NHS Trust, Charing Cross Hospital, London, UK.; (Drewes AM) Mech-Sense, Department of Gastroenterology & Hepatology, Aalborg University Hospital, Aalborg, Denmark.; (Dom G) Collaborative Antwerp Psychiatric Research Institute (CAPRI), Antwerp University (UA), Antwerp, Belgium.; (Jutila L) Pain

Alliance Europe, Finland.; (O'Brien T) College of Medicine & Health, University College Cork, Cork, Republic of Ireland.; (Pogatzki-Zahn E) Department of Anaesthesiology, Intensive Care and Pain Medicine, University Hospital Münster UKM, Munster, Germany.; (Rakusa M) Department of Neurology, University Medical Centre Maribor, Maribor, Slovenia.; (Suarez-Serrano C) Department of Physiotherapy, University of Sevilla, Sevilla, Spain.; (Tölle T) Department of Neurology, Techhnische Universität München, München, Germany.; (Häuser W) Department Internal Medicine 1, Saarbrücken, Germany.
(Krčevski Škvarč N) Department of Anesthesiology, Intensive Care and Pain Treatment, Faculty of Medicine of University Maribor, Maribor, Slovenia.; (Morlion B) Center for Algology & Pain Management, University Hospitals Leuven, Leuven, Belgium.; (Vowles KE) School of Psychology, Queen's University Belfast, Belfast, UK.; (Bannister K) Institute of Psychiatry, Psychology and Neuroscience, King's College London, London, UK.; (Buchsner E) Pain Management and Neuromodulation Centre EHC Hospital, Morges, Switzerland.; (Casale R) Neurorehabilitation Unit, Department of Rehabilitation, HABILITA, Bergamo, Italy.; (Chenot JF) Department of General Practice, Institute for Community Medicine, University Medicine Greifswald, Greifswald, Germany.; (Chumbley G) Imperial College Healthcare NHS Trust, Charing Cross Hospital, London, UK.; (Drewes AM) Mech-Sense, Department of Gastroenterology & Hepatology, Aalborg University Hospital, Aalborg, Denmark.; (Dom G) Collaborative Antwerp Psychiatric Research Institute (CAPRI), Antwerp University (UA), Antwerp, Belgium.; (Jutila L) Pain Alliance Europe, Finland.; (O'Brien T) College of Medicine & Health, University College Cork, Cork, Republic of Ireland.; (Pogatzki-Zahn E) Department of Anaesthesiology, Intensive Care and Pain Medicine, University Hospital Münster UKM, Munster, Germany.; (Rakusa M) Department of Neurology, University Medical Centre Maribor, Maribor, Slovenia.; (Suarez-Serrano C) Department of Physiotherapy, University of Sevilla, Sevilla, Spain.; (Tölle T) Department of Neurology, Techhnische Universität München, München, Germany.; (Häuser W) Department Internal Medicine 1, Saarbrücken, Germany.
(Krčevski Škvarč N) Department of Anesthesiology, Intensive Care and Pain Treatment, Faculty of Medicine of University Maribor, Maribor, Slovenia.; (Morlion B) Center for Algology & Pain Management, University Hospitals Leuven, Leuven, Belgium.; (Vowles KE) School of Psychology, Queen's University Belfast, Belfast, UK.; (Bannister K) Institute of Psychiatry, Psychology and Neuroscience, King's College London, London, UK.; (Buchsner E) Pain Management and Neuromodulation Centre EHC Hospital, Morges, Switzerland.; (Casale R) Neurorehabilitation Unit, Department of Rehabilitation, HABILITA, Bergamo, Italy.; (Chenot JF) Department of General Practice, Institute for Community Medicine, University Medicine Greifswald, Greifswald, Germany.; (Chumbley G) Imperial College Healthcare NHS Trust, Charing Cross Hospital, London, UK.; (Drewes AM) Mech-Sense, Department of Gastroenterology & Hepatology, Aalborg University Hospital, Aalborg, Denmark.; (Dom G) Collaborative Antwerp Psychiatric Research Institute (CAPRI), Antwerp University (UA), Antwerp, Belgium.; (Jutila L) Pain Alliance Europe, Finland.; (O'Brien T) College of Medicine & Health, University College Cork, Cork, Republic of Ireland.; (Pogatzki-Zahn E) Department of Anaesthesiology, Intensive Care and Pain Medicine, University Hospital Münster UKM, Munster, Germany.; (Rakusa M) Department of Neurology, University Medical Centre Maribor, Maribor, Slovenia.; (Suarez-Serrano C) Department of Physiotherapy, University of Sevilla, Sevilla, Spain.; (Tölle T) Department of

Neurology, Techhnische Universität München, München, Germany.; (Häuser W) Department Internal Medicine 1, Saarbrücken, Germany.

(Krčevski Škvarč N) Department of Anesthesiology, Intensive Care and Pain Treatment, Faculty of Medicine of University Maribor, Maribor, Slovenia.; (Morlion B) Center for Algology & Pain Management, University Hospitals Leuven, Leuven, Belgium.; (Vowles KE) School of Psychology, Queen's University Belfast, Belfast, UK.; (Bannister K) Institute of Psychiatry, Psychology and Neuroscience, King's College London, London, UK.; (Buchsner E) Pain Management and Neuromodulation Centre EHC Hospital, Morges, Switzerland.; (Casale R) Neurorehabilitation Unit, Department of Rehabilitation, HABILITA, Bergamo, Italy.; (Chenot JF) Department of General Practice, Institute for Community Medicine, University Medicine Greifswald, Greifswald, Germany.; (Chumbley G) Imperial College Healthcare NHS Trust, Charing Cross Hospital, London, UK.; (Drewes AM) Mech-Sense, Department of Gastroenterology & Hepatology, Aalborg University Hospital, Aalborg, Denmark.; (Dom G) Collaborative Antwerp Psychiatric Research Institute (CAPRI), Antwerp University (UA), Antwerp, Belgium.; (Jutila L) Pain Alliance Europe, Finland.; (O'Brien T) College of Medicine & Health, University College Cork, Cork, Republic of Ireland.; (Pogatzki-Zahn E) Department of Anaesthesiology, Intensive Care and Pain Medicine, University Hospital Münster UKM, Munster, Germany.; (Rakusa M) Department of Neurology, University Medical Centre Maribor, Maribor, Slovenia.; (Suarez-Serrano C) Department of Physiotherapy, University of Sevilla, Sevilla, Spain.; (Tölle T) Department of Neurology, Techhnische Universität München, München, Germany.; (Häuser W) Department Internal Medicine 1, Saarbrücken, Germany.

(Krčevski Škvarč N) Department of Anesthesiology, Intensive Care and Pain Treatment, Faculty of Medicine of University Maribor, Maribor, Slovenia.; (Morlion B) Center for Algology & Pain Management, University Hospitals Leuven, Leuven, Belgium.; (Vowles KE) School of Psychology, Queen's University Belfast, Belfast, UK.; (Bannister K) Institute of Psychiatry, Psychology and Neuroscience, King's College London, London, UK.; (Buchsner E) Pain Management and Neuromodulation Centre EHC Hospital, Morges, Switzerland.; (Casale R) Neurorehabilitation Unit, Department of Rehabilitation, HABILITA, Bergamo, Italy.; (Chenot JF) Department of General Practice, Institute for Community Medicine, University Medicine Greifswald, Greifswald, Germany.; (Chumbley G) Imperial College Healthcare NHS Trust, Charing Cross Hospital, London, UK.; (Drewes AM) Mech-Sense, Department of Gastroenterology & Hepatology, Aalborg University Hospital, Aalborg, Denmark.; (Dom G) Collaborative Antwerp Psychiatric Research Institute (CAPRI), Antwerp University (UA), Antwerp, Belgium.; (Jutila L) Pain Alliance Europe, Finland.; (O'Brien T) College of Medicine & Health, University College Cork, Cork, Republic of Ireland.; (Pogatzki-Zahn E) Department of Anaesthesiology, Intensive Care and Pain Medicine, University Hospital Münster UKM, Munster, Germany.; (Rakusa M) Department of Neurology, University Medical Centre Maribor, Maribor, Slovenia.; (Suarez-Serrano C) Department of Physiotherapy, University of Sevilla, Sevilla, Spain.; (Tölle T) Department of Neurology, Techhnische Universität München, München, Germany.; (Häuser W) Department Internal Medicine 1, Saarbrücken, Germany.

(Krčevski Škvarč N) Department of Anesthesiology, Intensive Care and Pain Treatment, Faculty of Medicine of University Maribor, Maribor, Slovenia.; (Morlion B) Center for Algology & Pain Management, University Hospitals Leuven, Leuven, Belgium.; (Vowles KE) School of Psychology, Queen's University Belfast, Belfast,

UK.; (Bannister K) Institute of Psychiatry, Psychology and Neuroscience, King's College London, London, UK.; (Buchsner E) Pain Management and Neuromodulation Centre EHC Hospital, Morges, Switzerland.; (Casale R) Neurorehabilitation Unit, Department of Rehabilitation, HABILITA, Bergamo, Italy.; (Chenot JF) Department of General Practice, Institute for Community Medicine, University Medicine Greifswald, Greifswald, Germany.; (Chumbley G) Imperial College Healthcare NHS Trust, Charing Cross Hospital, London, UK.; (Drewes AM) Mech-Sense, Department of Gastroenterology & Hepatology, Aalborg University Hospital, Aalborg, Denmark.; (Dom G) Collaborative Antwerp Psychiatric Research Institute (CAPRI), Antwerp University (UA), Antwerp, Belgium.; (Jutila L) Pain Alliance Europe, Finland.; (O'Brien T) College of Medicine & Health, University College Cork, Cork, Republic of Ireland.; (Pogatzki-Zahn E) Department of Anaesthesiology, Intensive Care and Pain Medicine, University Hospital Münster UKM, Munster, Germany.; (Rakusa M) Department of Neurology, University Medical Centre Maribor, Maribor, Slovenia.; (Suarez-Serrano C) Department of Physiotherapy, University of Sevilla, Sevilla, Spain.; (Tölle T) Department of Neurology, Techhnische Universität München, München, Germany.; (Häuser W) Department Internal Medicine 1, Saarbrücken, Germany.
Database: PubMed

10. Breaking the Barrier: Challenges of Methadone Use - An Introductory Observation.

Author(s): Sarma R; Sirohiya P; Ratre BK; Bhatnagar S

Source: Indian journal of palliative care; 2020; vol. 26 (no. 4); p. 495-499

Publication Date: 2020

Publication Type(s): Journal Article

DOI: http://dx.doi.org/10.4103/IJPC.IJPC_9_20

ISSN: 0973-1075

Place of Publication: United States

PubMedID: 33623311

Accession Number: 33623311

Available at Indian journal of palliative care - from Europe PubMed Central - Open Access

Available at Indian journal of palliative care - from EBSCO (CINAHL Complete)

Available at Indian journal of palliative care - from ProQuest (Health Research Premium) - NHS Version

Available at Indian journal of palliative care - from Unpaywall

Keywords: Subject Terms: Methadone; pain management; palliative care

Abstract: Background: Palliative care physicians in India have achieved access to methadone for pain relief in cancer patients. Despite being an effective drug in terms of analgesia, there are a number of reasons why this opioid medication is not as much as popular as morphine. We identified and tried to overcome a few such barriers in treating cancer pain with methadone.Methods: The clinical information of ten adult cancer patients (six males and four females), who voluntarily received methadone for their severe pain in the month of August 2019 were analysed

retrospectively. We converted morphine to methadone in all ten patients under the supervision of an experienced practitioner.Results: During the methadone therapy, eight out of ten patients who were given methadone exclusively for their pain had adequate pain relief initially. The barriers identified included difficult titration methods due to distinct pharmacology, patient selection, clinical inertia, communication and co-ordination among physicians, communication among patient and physician, and patient and caregivers, and vigilant monitoring.Conclusion: Methadone is still finding its place in India for cancer pain management. As the drug is new to Indian practitioners, we have to overcome these barriers and facilitate its judicious use in cancer pain management.

Institutions:
(Sarma R) Department of Onco-Anaesthesia and Palliative Medicine, Dr. BRA IRCH, AIIMS, New Delhi, India.; (Sirohiya P) Department of Anaesthesia, ABVIMS and Dr. RML Hospital, New Delhi, India.; (Ratre BK) Department of Onco-Anaesthesia and Palliative Medicine, Dr. BRA IRCH, AIIMS, New Delhi, India.; (Bhatnagar S) Department of Onco-Anaesthesia and Palliative Medicine, Dr. BRA IRCH, AIIMS, New Delhi, India.
(Sarma R) Department of Onco-Anaesthesia and Palliative Medicine, Dr. BRA IRCH, AIIMS, New Delhi, India.; (Sirohiya P) Department of Anaesthesia, ABVIMS and Dr. RML Hospital, New Delhi, India.; (Ratre BK) Department of Onco-Anaesthesia and Palliative Medicine, Dr. BRA IRCH, AIIMS, New Delhi, India.; (Bhatnagar S) Department of Onco-Anaesthesia and Palliative Medicine, Dr. BRA IRCH, AIIMS, New Delhi, India.
(Sarma R) Department of Onco-Anaesthesia and Palliative Medicine, Dr. BRA IRCH, AIIMS, New Delhi, India.; (Sirohiya P) Department of Anaesthesia, ABVIMS and Dr. RML Hospital, New Delhi, India.; (Ratre BK) Department of Onco-Anaesthesia and Palliative Medicine, Dr. BRA IRCH, AIIMS, New Delhi, India.; (Bhatnagar S) Department of Onco-Anaesthesia and Palliative Medicine, Dr. BRA IRCH, AIIMS, New Delhi, India.
(Sarma R) Department of Onco-Anaesthesia and Palliative Medicine, Dr. BRA IRCH, AIIMS, New Delhi, India.; (Sirohiya P) Department of Anaesthesia, ABVIMS and Dr. RML Hospital, New Delhi, India.; (Ratre BK) Department of Onco-Anaesthesia and Palliative Medicine, Dr. BRA IRCH, AIIMS, New Delhi, India.; (Bhatnagar S) Department of Onco-Anaesthesia and Palliative Medicine, Dr. BRA IRCH, AIIMS, New Delhi, India.
Database: PubMed

11. The opioid-prescribing practices of Australian general practice registrars: an interview study.

Author(s): Prathivadi P; Barton C; Mazza D

Source: Family practice; ; vol. 38 (no. 4); p. 473-478

Publication Type(s): Journal Article; Research Support, Non-U.S. Gov't

DOI: http://dx.doi.org/10.1093/fampra/cmaa148

ISSN: 1460-2229

Place of Publication: England

PubMedID: 33506867

Accession Number: 33506867

Keywords: Subject Terms: *Doctor–patient relationship; *graduate medical education/fellowship training; *pain; *prescription drug monitoring program; *primary care; *teaching methods

Abstract:BACKGROUND: Approximately half of the opioids prescribed by Australian GP and GP registrars are for chronic non-cancer pain-despite limited therapeutic benefit, and serious risks of harm. Understanding the factors driving non-evidence-based opioid prescribing may improve GP training and education.OBJECTIVE: To explore attitudes, beliefs, knowledge and self-reported factors influencing the opioid-prescribing decisions of Australian GP registrars.METHODS: Telephone interviews were undertaken with 20 GP registrars in 2018-19. Interviews were 30-60 minutes in duration, audio-recorded and de-identified. Braun and Clarke's 6-phase framework was adopted for reflexive thematic analysis of data and managed using QSR NVivo software.RESULTS: Twenty registrars were recruited; 8 men and 12 women. Three themes were identified.SUPPORT AND SUPERVISION: Difficult chronic pain consultations negatively affected the registrar well-being. Registrars role modelled their supervisors' opioid-prescribing practices, even if they perceived it to be unsafe.CONFIDENCE: Registrars lacked confidence in initiating, prescribing and weaning opioids, recognizing drug-seeking behaviours and declining to prescribe-but felt confident in their knowledge of opioid pharmacology.SAFETY: Registrars were aware of evidence-based prescribing recommendations and risk reduction strategies but struggled to translate this into practice.CONCLUSIONS: Non-evidence-based opioid prescribing by Australian GP registrars is multifactorial. Emotionally difficult pain consultations, poor supervision and low prescriber confidence may contribute to unsafe prescribing. Improving registrar prescribing may require interventions to improve risk reduction, training in communication and role modelling by supervisors.

Institutions:
(Prathivadi P) The Department of General Practice, Monash University, Notting Hill, Australia.; (Barton C) The Department of General Practice, Monash University, Notting Hill, Australia.; (Mazza D) The Department of General Practice, Monash University, Notting Hill, Australia.
(Prathivadi P) The Department of General Practice, Monash University, Notting Hill, Australia.; (Barton C) The Department of General Practice, Monash University, Notting Hill, Australia.; (Mazza D) The Department of General Practice, Monash University, Notting Hill, Australia.
(Prathivadi P) The Department of General Practice, Monash University, Notting Hill, Australia.; (Barton C) The Department of General Practice, Monash University, Notting Hill, Australia.; (Mazza D) The Department of General Practice, Monash University, Notting Hill, Australia.
Database: PubMed

12. Current Status of Cancer-Related Pain and Opioid use in South Lebanon: A Pilot Study.

Author(s): Farhat FS; Tarabey M; Chehade F; Assi T; Kattan J

Source: The Gulf journal of oncology; Sep 2020; vol. 1 (no. 34); p. 52-57

Publication Date: Sep 2020

Publication Type(s): Journal Article

ISSN: 2078-2101

Place of Publication: Kuwait

PubMedID: 33431363

Accession Number: 33431363

Abstract:OBJECTIVE: This study aimed to evaluate the patients and health providers' (doctors and nurses) knowledge and understanding of the disease-related pain, and the perception of pain drugs (opioids) in South Lebanon.PATIENTS AND METHODS: This was a pilot study conducted at different hospitals in South Lebanon among patients with confirmed cancer diagnosis and providers. Data was collected using patients' and providers' questionnaires.RESULTS: 43 patients and 42 providers were included. 22 (52%) patients were male. Nine (21%) patients were aware of their diagnosis and only 60% talked about their pain to their oncologist. Pain was not optimally controlled with 25 (58%) patients having uncontrolled pain and 18 (42%) patients having continuous pain. Morphine was negatively perceived with 55.8% of patients believing that morphine causes addiction and 59% taking pain medications only when the pain is maximal. This led to a 58% short duration control of intermittent pain. 60% of the providers were certain that cancer pain cannot be relieved by morphine while only 33% believed that morphine can cause complete relief. Addiction seemed to be the main obstacle for morphine use with 37 (89%) thinking that narcotics causes addiction and 51% considering morphine withdrawal if side effects appear. Finally, 30% suggested to discontinue morphine in the terminal stages of cancer.DISCUSSION AND CONCLUSION: Major misconceptions in cancer patients are observed in the approach to antalgic treatment in our population. With good education, better knowledge and optimal palliative care units, misconceptions about opioids can be corrected with best management of cancer pain.

Institutions:
(Farhat FS) Department of Hematology-Oncology, Hammoud Hospital University Medical Center, Saida, Lebanon.; (Tarabey M) Department of Hematology-Oncology, Hammoud Hospital University Medical Center, Saida, Lebanon.; (Chehade F) Department of Nuclear Medicine, Hammoud Hospital University Medical Center, Saida, Lebanon.; (Assi T) Department of Hematology-Oncology, Faculty of Medicine, Saint-Joseph University, Beirut, Lebanon.; (Kattan J) Department of Hematology-Oncology, Hôtel-Dieu de France University Hospital, Beirut, Lebanon.
(Farhat FS) Department of Hematology-Oncology, Hammoud Hospital University Medical Center, Saida, Lebanon.; (Tarabey M) Department of Hematology-Oncology, Hammoud Hospital University Medical Center, Saida, Lebanon.; (Chehade F) Department of Nuclear Medicine, Hammoud Hospital University Medical Center, Saida, Lebanon.; (Assi T) Department of Hematology-Oncology, Faculty of Medicine, Saint-Joseph University, Beirut, Lebanon.; (Kattan J) Department of Hematology-Oncology, Hôtel-Dieu de France University Hospital, Beirut, Lebanon.
(Farhat FS) Department of Hematology-Oncology, Hammoud Hospital University Medical Center, Saida, Lebanon.; (Tarabey M) Department of Hematology-Oncology, Hammoud Hospital University Medical Center, Saida, Lebanon.; (Chehade F) Department of Nuclear Medicine, Hammoud Hospital University Medical Center, Saida, Lebanon.; (Assi T) Department of Hematology-Oncology, Faculty of Medicine, Saint-Joseph University, Beirut, Lebanon.; (Kattan J) Department of Hematology-Oncology, Hôtel-Dieu de France University Hospital, Beirut, Lebanon.

(Farhat FS) Department of Hematology-Oncology, Hammoud Hospital University Medical Center, Saida, Lebanon.; (Tarabey M) Department of Hematology-Oncology, Hammoud Hospital University Medical Center, Saida, Lebanon.; (Chehade F) Department of Nuclear Medicine, Hammoud Hospital University Medical Center, Saida, Lebanon.; (Assi T) Department of Hematology-Oncology, Faculty of Medicine, Saint-Joseph University, Beirut, Lebanon.; (Kattan J) Department of Hematology-Oncology, Hôtel-Dieu de France University Hospital, Beirut, Lebanon.
(Farhat FS) Department of Hematology-Oncology, Hammoud Hospital University Medical Center, Saida, Lebanon.; (Tarabey M) Department of Hematology-Oncology, Hammoud Hospital University Medical Center, Saida, Lebanon.; (Chehade F) Department of Nuclear Medicine, Hammoud Hospital University Medical Center, Saida, Lebanon.; (Assi T) Department of Hematology-Oncology, Faculty of Medicine, Saint-Joseph University, Beirut, Lebanon.; (Kattan J) Department of Hematology-Oncology, Hôtel-Dieu de France University Hospital, Beirut, Lebanon.
Database: PubMed

13. Knowledge and attitudes regarding cancer pain management among oncology nurses in China.

Author(s): Li D; Gao L; Ren LY; Zeng X; Cui EP; Zhang LJ; Wu Q

Source: The Journal of international medical research; Jan 2021; vol. 49 (no. 1); p. 300060520979448

Publication Date: Jan 2021

Publication Type(s): Journal Article

DOI: http://dx.doi.org/10.1177/0300060520979448

ISSN: 1473-2300

Place of Publication: England

PubMedID: 33430662

Accession Number: 33430662

Available at The Journal of international medical research - from ProQuest (Health Research Premium) - NHS Version

Available at The Journal of international medical research - from Unpaywall

Keywords: Subject Terms: Pain management; attitudes; cross-sectional study; knowledge; oncology nurses; patients with cancer

Abstract:OBJECTIVE: There are limited studies examining knowledge and attitudes among Chinese oncology nurses regarding cancer pain management.METHODS: We conducted a cross-sectional survey among oncology nurses from 26 hospitals in China. The nurses completed the Knowledge and Attitudes Survey Regarding Pain (KASRP) questionnaire. Multivariate models were used to identify factors associated with nurses' KASRP score.RESULTS: A total of 982 nurses completed the KASRP (mean score = 21.56 ± 4.00), and 8 (0.81%) nurses had a passing score. The results of multivariate regression indicated that clinical rank and experience in cancer pain management were associated with good knowledge and attitudes regarding cancer pain management.CONCLUSION: The Chinese nurses in our study did not have adequate knowledge of or positive attitudes related to cancer pain management. Clinical rank and experience caring for patients with cancer could be used to help

identify nurses with inadequate knowledge and attitudes regarding treating cancer pain.

Institutions:
(Li D) Department of Radiotherapy, The Fifth Medical Center, Chinese PLA General Hospital (Former 307th Hospital of the PLA), Beijing, China.; (Gao L) Department of Pulmonary Oncology, The Fifth Medical Center, Chinese PLA General Hospital (Former 307th Hospital of the PLA), Beijing, China.; (Ren LY) Department of Radiotherapy, The Fifth Medical Center, Chinese PLA General Hospital (Former 307th Hospital of the PLA), Beijing, China.; (Zeng X) Department of Radiotherapy, The Fifth Medical Center, Chinese PLA General Hospital (Former 307th Hospital of the PLA), Beijing, China.; (Cui EP) Department of Pulmonary Oncology, The Fifth Medical Center, Chinese PLA General Hospital (Former 307th Hospital of the PLA), Beijing, China.; (Zhang LJ) Department of Vascular Surgery, Beijing Chaoyang Hospital, Capital Medical University, Beijing, China.; (Wu Q) Department of Nursing, The Fifth Medical Center, Chinese PLA General Hospital (Former 307th Hospital of the PLA), Beijing, China.
(Li D) Department of Radiotherapy, The Fifth Medical Center, Chinese PLA General Hospital (Former 307th Hospital of the PLA), Beijing, China.; (Gao L) Department of Pulmonary Oncology, The Fifth Medical Center, Chinese PLA General Hospital (Former 307th Hospital of the PLA), Beijing, China.; (Ren LY) Department of Radiotherapy, The Fifth Medical Center, Chinese PLA General Hospital (Former 307th Hospital of the PLA), Beijing, China.; (Zeng X) Department of Radiotherapy, The Fifth Medical Center, Chinese PLA General Hospital (Former 307th Hospital of the PLA), Beijing, China.; (Cui EP) Department of Pulmonary Oncology, The Fifth Medical Center, Chinese PLA General Hospital (Former 307th Hospital of the PLA), Beijing, China.; (Zhang LJ) Department of Vascular Surgery, Beijing Chaoyang Hospital, Capital Medical University, Beijing, China.; (Wu Q) Department of Nursing, The Fifth Medical Center, Chinese PLA General Hospital (Former 307th Hospital of the PLA), Beijing, China.
(Li D) Department of Radiotherapy, The Fifth Medical Center, Chinese PLA General Hospital (Former 307th Hospital of the PLA), Beijing, China.; (Gao L) Department of Pulmonary Oncology, The Fifth Medical Center, Chinese PLA General Hospital (Former 307th Hospital of the PLA), Beijing, China.; (Ren LY) Department of Radiotherapy, The Fifth Medical Center, Chinese PLA General Hospital (Former 307th Hospital of the PLA), Beijing, China.; (Zeng X) Department of Radiotherapy, The Fifth Medical Center, Chinese PLA General Hospital (Former 307th Hospital of the PLA), Beijing, China.; (Cui EP) Department of Pulmonary Oncology, The Fifth Medical Center, Chinese PLA General Hospital (Former 307th Hospital of the PLA), Beijing, China.; (Zhang LJ) Department of Vascular Surgery, Beijing Chaoyang Hospital, Capital Medical University, Beijing, China.; (Wu Q) Department of Nursing, The Fifth Medical Center, Chinese PLA General Hospital (Former 307th Hospital of the PLA), Beijing, China.
(Li D) Department of Radiotherapy, The Fifth Medical Center, Chinese PLA General Hospital (Former 307th Hospital of the PLA), Beijing, China.; (Gao L) Department of Pulmonary Oncology, The Fifth Medical Center, Chinese PLA General Hospital (Former 307th Hospital of the PLA), Beijing, China.; (Ren LY) Department of Radiotherapy, The Fifth Medical Center, Chinese PLA General Hospital (Former 307th Hospital of the PLA), Beijing, China.; (Zeng X) Department of Radiotherapy, The Fifth Medical Center, Chinese PLA General Hospital (Former 307th Hospital of the PLA), Beijing, China.; (Cui EP) Department of Pulmonary Oncology, The Fifth

Medical Center, Chinese PLA General Hospital (Former 307th Hospital of the PLA), Beijing, China.; (Zhang LJ) Department of Vascular Surgery, Beijing Chaoyang Hospital, Capital Medical University, Beijing, China.; (Wu Q) Department of Nursing, The Fifth Medical Center, Chinese PLA General Hospital (Former 307th Hospital of the PLA), Beijing, China.
(Li D) Department of Radiotherapy, The Fifth Medical Center, Chinese PLA General Hospital (Former 307th Hospital of the PLA), Beijing, China.; (Gao L) Department of Pulmonary Oncology, The Fifth Medical Center, Chinese PLA General Hospital (Former 307th Hospital of the PLA), Beijing, China.; (Ren LY) Department of Radiotherapy, The Fifth Medical Center, Chinese PLA General Hospital (Former 307th Hospital of the PLA), Beijing, China.; (Zeng X) Department of Radiotherapy, The Fifth Medical Center, Chinese PLA General Hospital (Former 307th Hospital of the PLA), Beijing, China.; (Cui EP) Department of Pulmonary Oncology, The Fifth Medical Center, Chinese PLA General Hospital (Former 307th Hospital of the PLA), Beijing, China.; (Zhang LJ) Department of Vascular Surgery, Beijing Chaoyang Hospital, Capital Medical University, Beijing, China.; (Wu Q) Department of Nursing, The Fifth Medical Center, Chinese PLA General Hospital (Former 307th Hospital of the PLA), Beijing, China.
(Li D) Department of Radiotherapy, The Fifth Medical Center, Chinese PLA General Hospital (Former 307th Hospital of the PLA), Beijing, China.; (Gao L) Department of Pulmonary Oncology, The Fifth Medical Center, Chinese PLA General Hospital (Former 307th Hospital of the PLA), Beijing, China.; (Ren LY) Department of Radiotherapy, The Fifth Medical Center, Chinese PLA General Hospital (Former 307th Hospital of the PLA), Beijing, China.; (Zeng X) Department of Radiotherapy, The Fifth Medical Center, Chinese PLA General Hospital (Former 307th Hospital of the PLA), Beijing, China.; (Cui EP) Department of Pulmonary Oncology, The Fifth Medical Center, Chinese PLA General Hospital (Former 307th Hospital of the PLA), Beijing, China.; (Zhang LJ) Department of Vascular Surgery, Beijing Chaoyang Hospital, Capital Medical University, Beijing, China.; (Wu Q) Department of Nursing, The Fifth Medical Center, Chinese PLA General Hospital (Former 307th Hospital of the PLA), Beijing, China.
(Li D) Department of Radiotherapy, The Fifth Medical Center, Chinese PLA General Hospital (Former 307th Hospital of the PLA), Beijing, China.; (Gao L) Department of Pulmonary Oncology, The Fifth Medical Center, Chinese PLA General Hospital (Former 307th Hospital of the PLA), Beijing, China.; (Ren LY) Department of Radiotherapy, The Fifth Medical Center, Chinese PLA General Hospital (Former 307th Hospital of the PLA), Beijing, China.; (Zeng X) Department of Radiotherapy, The Fifth Medical Center, Chinese PLA General Hospital (Former 307th Hospital of the PLA), Beijing, China.; (Cui EP) Department of Pulmonary Oncology, The Fifth Medical Center, Chinese PLA General Hospital (Former 307th Hospital of the PLA), Beijing, China.; (Zhang LJ) Department of Vascular Surgery, Beijing Chaoyang Hospital, Capital Medical University, Beijing, China.; (Wu Q) Department of Nursing, The Fifth Medical Center, Chinese PLA General Hospital (Former 307th Hospital of the PLA), Beijing, China.
Database: PubMed

14. Modular transitional nursing intervention improves pain-related self-management for cancer patients: Study protocol for a randomized controlled trial.

Author(s): Miao B; Sun Y; Gong L; Liu W
Source: Medicine; Dec 2020; vol. 99 (no. 51); p. e23867
Publication Date: Dec 2020
Publication Type(s): Journal Article
DOI: http://dx.doi.org/10.1097/MD.0000000000023867
ISSN: 1536-5964
Place of Publication: United States
PubMedID: 33371172
Accession Number: 33371172
Available at Medicine - from Europe PubMed Central - Open Access
Available at Medicine - from Ovid (Journals @ Ovid)
Available at Medicine - from Ovid (Journals @ Ovid) - London Health Libraries
Available at Medicine - from Unpaywall
Abstract:OBJECTIVE: To explore the effect of modular transitional nursing intervention on the improvement of self-management of the patients with cancer pain.METHOD: This study will be conducted from March 2021 to May 2022 at Affiliated Hospital of Beihua University. The experiment was granted through the Research Ethics Committee of Affiliated Hospital of Beihua University (4348-019). Eighty patients are analyzed in our study. The patients will be included if they are between 18 and 70 years old and are diagnosed with cancer, the pain intensity score on moderate level, the pain lasts for more than 3 days, and the patients who have signed the written informed consent. While the patients will be excluded if they have a documented history of drug or alcohol abuse, and patients with limited performance, and patients have a surgery in the past 3 days. The primary result mainly expresses as intergroup differences in self-management disorders (Barriers Questionnaire-II) associated with the cancer pain. And the secondary results include the quality of life (QOL) and pain intensity. All the analyses are implemented with SPSS for Windows Version 20.0.RESULTS: Table 1 will show the clinical outcomes between the 2 groups.CONCLUSION: A modular transitional nursing intervention appears to reduce pain in cancer patients.TRIAL REGISTRATION NUMBER: researchregistry6262.
Institutions:
(Miao B) School of nursing, Beihua University, Jilin, China.
Database: PubMed

15. Beyond the black stump: rapid reviews of health research issues affecting regional, rural and remote Australia.

Author(s): Osborne SR; Alston LV; Bolton KA; Whelan J; Reeve E; Wong Shee A; Browne J; Walker T; Versace VL; Allender S; Nichols M; Backholer K; Goodwin N; Lewis S; Dalton H; Prael G; Curtin M; Brooks R; Verdon S; Crockett J; Hodgins G; Walsh S; Lyle DM; Thompson SC; Browne LJ; Knight S; Pit SW; Jones M; Gillam MH; Leach MJ; Gonzalez-Chica DA; Muyambi K; Eshetie T; Tran K; May E; Lieschke G; Parker V; Smith A; Hayes C; Dunlop AJ; Rajappa H; White R; Oakley P; Holliday S

Source: The Medical journal of Australia; 2020 ; p. S3-S32.e1
Publication Date: 2020
Publication Type(s): Journal Article
DOI: http://dx.doi.org/10.5694/mja2.50881
ISSN: 1326-5377
Place of Publication: Australia
PubMedID: 33314144
Accession Number: 33314144
Available at The Medical journal of Australia - from Unpaywall
Abstract:CHAPTER 1: RETAIL INITIATIVES TO IMPROVE THE HEALTHINESS OF FOOD ENVIRONMENTS IN RURAL, REGIONAL AND REMOTE COMMUNITIES: Objective: To synthesise the evidence for effectiveness of initiatives aimed at improving food retail environments and consumer dietary behaviour in rural, regional and remote populations in Australia and comparable countries, and to discuss the implications for future food environment initiatives for rural, regional and remote areas of Australia.STUDY DESIGN: Rapid review of articles published between January 2000 and May 2020.DATA SOURCES: We searched MEDLINE (EBSCOhost), Health and Society Database (Informit) and Rural and Remote Health Database (Informit), and included studies undertaken in rural food environment settings in Australia and other countries.DATA SYNTHESIS: Twenty-one articles met the inclusion criteria, including five conducted in Australia. Four of the Australian studies were conducted in very remote populations and in grocery stores, and one was conducted in regional Australia. All of the overseas studies were conducted in rural North America. All of them revealed a positive influence on food environment or consumer behaviour, and all were conducted in disadvantaged, rural communities. Positive outcomes were consistently revealed by studies of initiatives that focused on promotion and awareness of healthy foods and included co-design to generate community ownership and branding.CONCLUSION: Initiatives aimed at improving rural food retail environments were effective and, when implemented in different rural settings, may encourage improvements in population diets. The paucity of studies over the past 20 years in Australia shows a need for more research into effective food retail environment initiatives, modelled on examples from overseas, with studies needed across all levels of remoteness in Australia. Several retail initiatives that were undertaken in rural North America could be replicated in rural Australia and could underpin future research. CHAPTER 2: WHICH INTERVENTIONS BEST SUPPORT THE HEALTH AND WELLBEING NEEDS OF RURAL POPULATIONS EXPERIENCING NATURAL DISASTERS?: Objective: To explore and evaluate health and social care interventions delivered to rural and remote communities experiencing natural disasters in Australia and other high income countries.STUDY DESIGN: We used systematic rapid review methods. First we identified a test set of citations and generated a frequency table of Medical Subject Headings (MeSH) to index articles. Then we used combinations of MeSH terms and keywords to search the MEDLINE (Ovid) database, and screened the titles and abstracts of the retrieved references.DATA SOURCES: We identified 1438 articles via database searches, and a further 62 articles via hand searching of key journals and reference lists. We also found four relevant grey literature resources. After removing duplicates and undertaking two stages of screening, we included 28 studies in a synthesis of

qualitative evidence.DATA SYNTHESIS: Four of us read and assessed the full text articles. We then conducted a thematic analysis using the three phases of the natural disaster response cycle.CONCLUSION: There is a lack of robust evaluation of programs and interventions supporting the health and wellbeing of people in rural communities affected by natural disasters. To address the cumulative and long term impacts, evidence suggests that continuous support of people's health and wellbeing is needed. By using a lens of rural adversity, the complexity of the lived experience of natural disasters by rural residents can be better understood and can inform development of new models of community-based and integrated care services. CHAPTER 3: THE IMPACT OF BUSHFIRE ON THE WELLBEING OF CHILDREN LIVING IN RURAL AND REMOTE AUSTRALIA: Objective: To investigate the impact of bushfire events on the wellbeing of children living in rural and remote Australia.STUDY DESIGN: Literature review completed using rapid realist review methods, and taking into consideration the PRISMA (Preferred Reporting Items for Systematic Reviews and Meta-Analyses) statement for systematic reviews.DATA SOURCES: We sourced data from six databases: EBSCOhost (Education), EBSCOhost (Health), EBSCOhost (Psychology), Informit, MEDLINE and PsycINFO. We developed search terms to identify articles that could address the research question based on the inclusion criteria of peer reviewed full text journal articles published in English between 1983 and 2020. We initially identified 60 studies and, following closer review, extracted data from eight studies that met the inclusion criteria.DATA SYNTHESIS: Children exposed to bushfires may be at increased risk of poorer wellbeing outcomes. Findings suggest that the impact of bushfire exposure may not be apparent in the short term but may become more pronounced later in life. Children particularly at risk are those from more vulnerable backgrounds who may have compounding factors that limit their ability to overcome bushfire trauma.CONCLUSION: We identified the short, medium and long term impacts of bushfire exposure on the wellbeing of children in Australia. We did not identify any evidence-based interventions for supporting outcomes for this population. Given the likely increase in bushfire events in Australia, research into effective interventions should be a priority. CHAPTER 4: THE ROLE OF NATIONAL POLICIES TO ADDRESS RURAL ALLIED HEALTH, NURSING AND DENTISTRY WORKFORCE MALDISTRIBUTION: Objective: Maldistribution of the health workforce between rural, remote and metropolitan communities contributes to longstanding health inequalities. Many developed countries have implemented policies to encourage health care professionals to work in rural and remote communities. This scoping review is an international synthesis of those policies, examining their effectiveness at recruiting and retaining nursing, dental and allied health professionals in rural communities.STUDY DESIGN: Using scoping review methods, we included primary research - published between 1 September 2009 and 30 June 2020 - that reported an evaluation of existing policy initiatives to address workforce maldistribution in high income countries with a land mass greater than 100 000 km2 .DATA SOURCES: We searched MEDLINE, Ovid Embase, Ovid Emcare, Informit, Scopus, and Web of Science. We screened 5169 articles for inclusion by title and abstract, of which we included 297 for full text screening. We then extracted data on 51 studies that had been conducted in Australia, the United States, Canada, United Kingdom and Norway.DATA SYNTHESIS: We grouped the studies based on World Health Organization recommendations on recruitment and retention of health care workers: education strategies (n = 27), regulatory change (n = 11), financial incentives (n = 6), personal and professional support (n = 4), and approaches with multiple components

(n = 3).CONCLUSION: Considerable work has occurred to address workforce maldistribution at a local level, underpinned by good practice guidelines, but rarely at scale or with explicit links to coherent overarching policy. To achieve policy aspirations, multiple synergistic evidence-based initiatives are needed, and implementation must be accompanied by well designed longitudinal evaluations that assess the effectiveness of policy objectives. CHAPTER 5: AVAILABILITY AND CHARACTERISTICS OF PUBLICLY AVAILABLE HEALTH WORKFORCE DATA SOURCES IN AUSTRALIA: Objective: Many data sources are used in Australia to inform health workforce planning, but their characteristics in terms of rele

Database: PubMed

16. Differences in nurses' perceptions of self-reported pain and the administered morphine dose according to the patient's facial expression in Korea

Author(s): Park JY; Lee DI

Source: Journal of educational evaluation for health professions; 2020; vol. 17 ; p. 38

Publication Date: 2020

Publication Type(s): Journal Article

DOI: http://dx.doi.org/10.3352/jeehp.2020.17.38

ISSN: 1975-5937

Place of Publication: Korea (South)

PubMedID: 33264827

Accession Number: 33264827

Available at Journal of educational evaluation for health professions - from Europe PubMed Central - Open Access

Available at Journal of educational evaluation for health professions - from Unpaywall

Keywords: Subject Terms: *Cancer pain; *Facial expression; *Morphine; *Pain management; *Republic of Korea

Abstract:PURPOSE: This study aimed to compare nurses' perceptions of self-reported pain, the recorded pain score, and pain treatment according to the patient's facial expression.METHODS: In this descriptive cross-sectional survey, the participants were 472 nurses working at a tertiary hospital in Seoul, Korea. A self-report questionnaire presented nurses with a smiling patient complaining of acute post-surgical pain and a grimacing patient with cancer pain, both of whom reported a pain level of 8 out of 10, and asked nurses to indicate their perception of the pain intensity, the pain score that they would record, and the medication that they would provide for each patient.RESULTS: The pain intensity perceived by nurses for the grimacing patient was significantly higher than that for the smiling patient (P<0.001). The recorded pain score was likewise significantly higher for the grimacing patient than for the smiling patient (P<0.001). There was a significant difference in the amount of morphine chosen by the nurses for pain interventions between the smiling and grimacing patients (P=0.040). Higher perceived pain intensity and score were associated with higher administered doses of morphine.CONCLUSION: These

findings suggest that nurses might be affected by patients' facial expressions when treating pain. A pain management program should be developed that trains nurses to accurately recognize pain hidden in patients' faces and provides them with the knowledge of how to appropriately assess and manage patients' pain.

Institutions:
(Park JY) Department of Clinical Nursing, University of Ulsan, Seoul, Korea.; (Lee DI) Department of Nursing, Seoil University, Seoul, Korea .
(Park JY) Department of Clinical Nursing, University of Ulsan, Seoul, Korea.; (Lee DI) Department of Nursing, Seoil University, Seoul, Korea .
Database: PubMed

17. Exploration of Patient-Related Barriers to Effective Cancer Pain Management in a Diverse Multicultural Developing Country.

Author(s): Kiu DKL; Lee ZFD; Voon PJ

Source: Journal of pain and symptom management; 2021; vol. 62 (no. 1); p. 75-80

Publication Date: 2021

Publication Type(s): Journal Article

DOI: http://dx.doi.org/10.1016/j.jpainsymman.2020.11.011

ISSN: 1873-6513

Place of Publication: United States

PubMedID: 33197524

Accession Number: 33197524

Available at Journal of pain and symptom management - from ScienceDirect

Keywords: Subject Terms: *Cancer pain management barriers; *cross-sectional survey; *effective cancer pain management; *fatalism

Abstract:CONTEXT: Cancer pain prevalence is high despite well-established international guidelines on pain management and improved accessibility to treatment. Inadequate cancer pain management can be attributed to barriers related to patients, health care professionals, and health care system.OBJECTIVES: To identify patient-related barriers to effective cancer pain management in a diverse multicultural developing country.DESIGN: A cross-sectional survey study was carried out using Brief Pain Inventory-Short Form to measure effectiveness of pain management and Barriers Questionnaire II to explore patient-related barriers to effective pain management.SETTING/PARTICIPANTS: Patients on strong opioids treated in a comprehensive cancer unit of a public hospital in Sarawak, Malaysia.RESULTS: Among 133 subjects surveyed, 66% reported no pain or mild pain, 34% moderate pain, and 10% severe pain. Despite good pain control, 71% of patients still reported moderate-to-severe interference with daily activities. Fatalism scored the highest median Barriers Questionnaire II score among the four domains of patient-related barriers followed by harmful effects, physiological effects, and communication factor.CONCLUSION: Cancer pain is generally well controlled with more than half of patients reporting mild pain. However, degree of interference with daily activities is still high despite good cancer pain control. Fatalistic mentality need to be addressed for effective cancer pain management. Further studies on health

care professional-related barriers and health system-related barriers are urgently needed to provide a comprehensive approach of holistic pain management.

Institutions:
(Kiu DKL) Department of Radiotherapy and Oncology, Hospital Umum Sarawak, Kuching, Sarawak, Malaysia. Electronic address: dkiu85@gmail.com.; (Lee ZFD) Department of Radiotherapy and Oncology, Hospital Umum Sarawak, Kuching, Sarawak, Malaysia.; (Voon PJ) Department of Radiotherapy and Oncology, Hospital Umum Sarawak, Kuching, Sarawak, Malaysia.
(Kiu DKL) Department of Radiotherapy and Oncology, Hospital Umum Sarawak, Kuching, Sarawak, Malaysia. Electronic address: dkiu85@gmail.com.; (Lee ZFD) Department of Radiotherapy and Oncology, Hospital Umum Sarawak, Kuching, Sarawak, Malaysia.; (Voon PJ) Department of Radiotherapy and Oncology, Hospital Umum Sarawak, Kuching, Sarawak, Malaysia.
(Kiu DKL) Department of Radiotherapy and Oncology, Hospital Umum Sarawak, Kuching, Sarawak, Malaysia. Electronic address: dkiu85@gmail.com.; (Lee ZFD) Department of Radiotherapy and Oncology, Hospital Umum Sarawak, Kuching, Sarawak, Malaysia.; (Voon PJ) Department of Radiotherapy and Oncology, Hospital Umum Sarawak, Kuching, Sarawak, Malaysia.
Database: PubMed

18. Effectiveness of advanced nursing care (ANC) on bone cancer pain, psychological disorders and quality of life in patients with primary bone cancers: A protocol for a PRISMA-compliant meta-analysis.

Author(s): Li L; Liu Y; Ren X; Qu K; Liu X

Source: Medicine; Oct 2020; vol. 99 (no. 43); p. e22711

Publication Date: Oct 2020

Publication Type(s): Journal Article

DOI: http://dx.doi.org/10.1097/MD.0000000000022711

ISSN: 1536-5964

Place of Publication: United States

PubMedID: 33120765

Accession Number: 33120765

Available at Medicine - from Europe PubMed Central - Open Access

Available at Medicine - from Ovid (Journals @ Ovid)

Available at Medicine - from Ovid (Journals @ Ovid) - London Health Libraries

Available at Medicine - from Unpaywall

Abstract:BACKGROUND: Advanced nursing care (ANC) has been reported to effectively relieve bone cancer pain, prevent psychological disorders and improve the quality of life (QoL) in patients with primary bone cancers (PBC) during the treatment. However, the exact effect of ANC remains controversial. This systematic review will aimed to assess the effectiveness of ANC on bone cancer pain, psychological disorders and QoL in patients with PBC.METHODS: Eligible randomized controlled trials (RCTs) and high-quality prospective cohort studies were searched from Excerpt Medica Database (Embase), PubMed, Google Scholar, Medline, Cochrane Library, Web of Science (WOS), China National Knowledge

Infrastructure (CNKI), Chinese Bio Medical Database (CBM), China Scientific Journal Database (CSJD), and Wanfang Database. Papers in English or Chinese published from January 2000 to July 2020 will be included without any restrictions. The clinical outcomes including bone cancer pain, psychological disorders, QoL, and adverse events of ANC in patients with PBC were systematically evaluated.Two reviewers will separately carry out study selection and data extraction. Stata 14.0 and Review Manager 5.3 were used for data analysis. Methodological quality for each eligible clinical trial will be assessed by using Cochrane risk of bias tool. Subgroup and meta-regression analysis will be carried out depending on the availability of sufficient data.RESULTS: This study will comprehensively summarize all potential evidence to systematically investigate the effects and safety of ANC on bone cancer pain, psychological disorders and QoL in patients with PBC.CONCLUSION: The findings of this study will help to determine whether ANC is effective or not on bone cancer pain, psychological disorders and QoL in patients with PBC.INPLASY REGISTRATION NUMBER: INPLASY202090037.

Institutions:
(Li L) Department of Spinal Surgery.; (Liu Y) Department of Spinal Surgery.; (Ren X) Department of Nursing, Liaocheng People's Hospital, Liaocheng, Shandong Province.; (Qu K) Department of Hepatobiliary Surgery, The First Affiliated Hospital of Xi'an Jiaotong University, Xi'an, Shaanxi Province, China.; (Liu X) Department of Spinal Surgery.
(Li L) Department of Spinal Surgery.; (Liu Y) Department of Spinal Surgery.; (Ren X) Department of Nursing, Liaocheng People's Hospital, Liaocheng, Shandong Province.; (Qu K) Department of Hepatobiliary Surgery, The First Affiliated Hospital of Xi'an Jiaotong University, Xi'an, Shaanxi Province, China.; (Liu X) Department of Spinal Surgery.
(Li L) Department of Spinal Surgery.; (Liu Y) Department of Spinal Surgery.; (Ren X) Department of Nursing, Liaocheng People's Hospital, Liaocheng, Shandong Province.; (Qu K) Department of Hepatobiliary Surgery, The First Affiliated Hospital of Xi'an Jiaotong University, Xi'an, Shaanxi Province, China.; (Liu X) Department of Spinal Surgery.
(Li L) Department of Spinal Surgery.; (Liu Y) Department of Spinal Surgery.; (Ren X) Department of Nursing, Liaocheng People's Hospital, Liaocheng, Shandong Province.; (Qu K) Department of Hepatobiliary Surgery, The First Affiliated Hospital of Xi'an Jiaotong University, Xi'an, Shaanxi Province, China.; (Liu X) Department of Spinal Surgery.
(Li L) Department of Spinal Surgery.; (Liu Y) Department of Spinal Surgery.; (Ren X) Department of Nursing, Liaocheng People's Hospital, Liaocheng, Shandong Province.; (Qu K) Department of Hepatobiliary Surgery, The First Affiliated Hospital of Xi'an Jiaotong University, Xi'an, Shaanxi Province, China.; (Liu X) Department of Spinal Surgery.
Database: PubMed

19. Effectiveness of Education Program on Nursing Knowledge and Attitude toward Pain Management.

Author(s): El-Aqoul A; Obaid A; Jarrah I; Al-Rawashdeh K; Al Hroub A

Source: Asia-Pacific journal of oncology nursing; 2020; vol. 7 (no. 4); p. 382-388

Publication Date: 2020

Publication Type(s): Journal Article
DOI: http://dx.doi.org/10.4103/apjon.apjon_17_20
ISSN: 2347-5625
Place of Publication: India
PubMedID: 33062835
Accession Number: 33062835
Available at Asia-Pacific journal of oncology nursing - from Europe PubMed Central - Open Access
Available at Asia-Pacific journal of oncology nursing - from Unpaywall
Keywords: Subject Terms: Attitude; cancer pain; education program; knowledge
Abstract: Objective: Nurses have an integral role in pain assessment and management. Adequate knowledge and positive attitudes toward pain management are essential to provide high-quality nursing care for cancer pain. The purposes of this study are to evaluate nurses' knowledge and attitude toward cancer-related pain and to assess the effectiveness of a pain management education program on nurses' knowledge and attitude toward pain.Methods: A quantitative, experimental design was used.Results: The total number of participants who were surveyed at three measurement points was 131, with a completion rate of 87.3%. Findings revealed that the score of knowledge and attitude toward cancer-related pain ranged from 14 to 35, with a mean of 23.6 (standard deviation [SD] = 4.38). The mean scores of the intervention group and the control group at two measurement points regarding knowledge and attitude toward cancer-related pain were 32.7 (SD = 2.8) and 32.8 (SD = 4.3) and 23 (SD = 5.5) and 22.2 (SD = 3.8), respectively. There were significant differences at three measurement points among the intervention group (F = 114.3, P < 0.0005). There were no differences in the three measurement points among the control group (F = 3.4, P = 0.055).Conclusions: Nurses have essential roles in cancer pain. A pain management education program can improve nurses' knowledge and attitude toward cancer-related pain.
Institutions:
(El-Aqoul A) Department of Nursing, King Hussein Cancer Center, Amman, Jordan.; (Obaid A) Department of Nursing, King Hussein Cancer Center, Amman, Jordan.; (Jarrah I) Department of Nursing, King Hussein Cancer Center, Amman, Jordan.; (Al-Rawashdeh K) Department of Nursing, King Hussein Cancer Center, Amman, Jordan.; (Al Hroub A) Department of Nursing, King Hussein Cancer Center, Amman, Jordan.
(El-Aqoul A) Department of Nursing, King Hussein Cancer Center, Amman, Jordan.; (Obaid A) Department of Nursing, King Hussein Cancer Center, Amman, Jordan.; (Jarrah I) Department of Nursing, King Hussein Cancer Center, Amman, Jordan.; (Al-Rawashdeh K) Department of Nursing, King Hussein Cancer Center, Amman, Jordan.; (Al Hroub A) Department of Nursing, King Hussein Cancer Center, Amman, Jordan.
(El-Aqoul A) Department of Nursing, King Hussein Cancer Center, Amman, Jordan.; (Obaid A) Department of Nursing, King Hussein Cancer Center, Amman, Jordan.; (Jarrah I) Department of Nursing, King Hussein Cancer Center, Amman, Jordan.; (Al-Rawashdeh K) Department of Nursing, King Hussein Cancer Center, Amman, Jordan.; (Al Hroub A) Department of Nursing, King Hussein Cancer Center, Amman, Jordan.

(El-Aqoul A) Department of Nursing, King Hussein Cancer Center, Amman, Jordan.; (Obaid A) Department of Nursing, King Hussein Cancer Center, Amman, Jordan.; (Jarrah I) Department of Nursing, King Hussein Cancer Center, Amman, Jordan.; (Al-Rawashdeh K) Department of Nursing, King Hussein Cancer Center, Amman, Jordan.; (Al Hroub A) Department of Nursing, King Hussein Cancer Center, Amman, Jordan.
(El-Aqoul A) Department of Nursing, King Hussein Cancer Center, Amman, Jordan.; (Obaid A) Department of Nursing, King Hussein Cancer Center, Amman, Jordan.; (Jarrah I) Department of Nursing, King Hussein Cancer Center, Amman, Jordan.; (Al-Rawashdeh K) Department of Nursing, King Hussein Cancer Center, Amman, Jordan.; (Al Hroub A) Department of Nursing, King Hussein Cancer Center, Amman, Jordan.
Database: PubMed

20. Knowledge and Attitude of Ethiopian Oncology Nurses About Cancer Pain Management: National Survey.

Author(s): Admass BA; Endalew NS; Tawuye HY; Mersha AT

Source: Cancer management and research; 2020; vol. 12 ; p. 9045-9055

Publication Date: 2020

Publication Type(s): Journal Article

DOI: http://dx.doi.org/10.2147/CMAR.S261172

ISSN: 1179-1322

Place of Publication: New Zealand

PubMedID: 33061599

Accession Number: 33061599

Available at Cancer management and research - from Europe PubMed Central - Open Access

Available at Cancer management and research - from DOAJ - Directory of Open Access Journals

Available at Cancer management and research - from Unpaywall

Keywords: Subject Terms: attitude; cancer; knowledge; nurses; pain management

Abstract: Background: Cancer is becoming a leading cause of death worldwide. Pain is a common and devastating symptom of cancer patients that can significantly affect the patient's quality of life. Optimal cancer pain control requires adequate knowledge and positive attitudes of nurses. Little is known about the knowledge and attitudes of oncology nurses towards cancer pain management in Ethiopia. The current study aimed to assess the knowledge and attitude of nurses and determinants of cancer pain management in all oncology centers in Ethiopia.Methods: A nationwide cross-sectional survey was conducted on 138 nurses in all oncology centers in Ethiopia. Self-completed survey questionnaires were distributed using the 'Knowledge and Attitudes Survey Regarding Pain (KASRP)' tool. Both bivariable and multivariable logistic regression analyses were used. Both crude odds ratio and adjusted odds ratio with the corresponding 95% CI were calculated to show the strength of association. Variables with a p-value of <0.05 were considered as statistically significant.Results: Only 7.2% (95% CI: 2.9, 11.6) of oncology nurses had good

knowledge and attitude about cancer pain management. Among 41 items, the mean number of correctly answered questions was 20.4 (SD = 5.13). Nurses who had a master's degree in nursing were positively associated with good knowledge and attitude about cancer pain management.Conclusion: In this nationwide study, the overall knowledge and attitude level of oncology nurses towards cancer pain management were poor. Nurses who had a master's degree in nursing were significantly associated with good knowledge and attitude towards cancer pain management. Regular training and revision of the contents of pain management education in the academic curriculum of nursing education are recommended.

Institutions:
(Admass BA) Department of Anaesthesia, School of Medicine, College of Medicine and Health Science University of Gondar, Gondar, Ethiopia.; (Endalew NS) Department of Anaesthesia, School of Medicine, College of Medicine and Health Science University of Gondar, Gondar, Ethiopia.; (Tawuye HY) Department of Anaesthesia, School of Medicine, College of Medicine and Health Science University of Gondar, Gondar, Ethiopia.; (Mersha AT) Department of Anaesthesia, School of Medicine, College of Medicine and Health Science University of Gondar, Gondar, Ethiopia.
(Admass BA) Department of Anaesthesia, School of Medicine, College of Medicine and Health Science University of Gondar, Gondar, Ethiopia.; (Endalew NS) Department of Anaesthesia, School of Medicine, College of Medicine and Health Science University of Gondar, Gondar, Ethiopia.; (Tawuye HY) Department of Anaesthesia, School of Medicine, College of Medicine and Health Science University of Gondar, Gondar, Ethiopia.; (Mersha AT) Department of Anaesthesia, School of Medicine, College of Medicine and Health Science University of Gondar, Gondar, Ethiopia.
(Admass BA) Department of Anaesthesia, School of Medicine, College of Medicine and Health Science University of Gondar, Gondar, Ethiopia.; (Endalew NS) Department of Anaesthesia, School of Medicine, College of Medicine and Health Science University of Gondar, Gondar, Ethiopia.; (Tawuye HY) Department of Anaesthesia, School of Medicine, College of Medicine and Health Science University of Gondar, Gondar, Ethiopia.; (Mersha AT) Department of Anaesthesia, School of Medicine, College of Medicine and Health Science University of Gondar, Gondar, Ethiopia.
(Admass BA) Department of Anaesthesia, School of Medicine, College of Medicine and Health Science University of Gondar, Gondar, Ethiopia.; (Endalew NS) Department of Anaesthesia, School of Medicine, College of Medicine and Health Science University of Gondar, Gondar, Ethiopia.; (Tawuye HY) Department of Anaesthesia, School of Medicine, College of Medicine and Health Science University of Gondar, Gondar, Ethiopia.; (Mersha AT) Department of Anaesthesia, School of Medicine, College of Medicine and Health Science University of Gondar, Gondar, Ethiopia.

Database: PubMed

21. Time trends and prescribing patterns of opioid drugs in UK primary care patients with non-cancer pain: A retrospective cohort study.

Author(s): Jani M; Birlie Yimer B; Sheppard T; Lunt M; Dixon WG

Source: PLoS medicine; 2020; vol. 17 (no. 10); p. e1003270

Publication Date: 2020

Publication Type(s): Historical Article; Journal Article; Research Support, Non-U.S. Gov't

DOI: http://dx.doi.org/10.1371/journal.pmed.1003270

ISSN: 1549-1676

Place of Publication: United States

PubMedID: 33057368

Accession Number: 33057368

Available at PLoS medicine - from Europe PubMed Central - Open Access

Available at PLoS medicine - from Public Library of Science (PLoS)

Available at PLoS medicine - from DOAJ - Directory of Open Access Journals

Available at PLoS medicine - from EBSCO (MEDLINE Complete)

Available at PLoS medicine - from ProQuest (MEDLINE with Full Text) - NHS Version

Available at PLoS medicine - from ProQuest (Health Research Premium) - NHS Version

Available at PLoS medicine - from Unpaywall

Abstract:BACKGROUND: The US opioid epidemic has led to similar concerns about prescribed opioids in the UK. In new users, initiation of or escalation to more potent and high dose opioids may contribute to long-term use. Additionally, physician prescribing behaviour has been described as a key driver of rising opioid prescriptions and long-term opioid use. No studies to our knowledge have investigated the extent to which regions, practices, and prescribers vary in opioid prescribing whilst accounting for case mix. This study sought to (i) describe prescribing trends between 2006 and 2017, (ii) evaluate the transition of opioid dose and potency in the first 2 years from initial prescription, (iii) quantify and identify risk factors for long-term opioid use, and (iv) quantify the variation of long-term use attributed to region, practice, and prescriber, accounting for case mix and chance variation.METHODS AND FINDINGS: A retrospective cohort study using UK primary care electronic health records from the Clinical Practice Research Datalink was performed. Adult patients without cancer with a new prescription of an opioid were included; 1,968,742 new users of opioids were identified. Mean age was 51 ± 19 years, and 57% were female. Codeine was the most commonly prescribed opioid, with use increasing 5-fold from 2006 to 2017, reaching 2,456 prescriptions/10,000 people/year. Morphine, buprenorphine, and oxycodone prescribing rates continued to rise steadily throughout the study period. Of those who started on high dose (120-199 morphine milligram equivalents [MME]/day) or very high dose opioids (≥200 MME/day), 10.3% and 18.7% remained in the same MME/day category or higher at 2 years, respectively. Following opioid initiation, 14.6% became long-term opioid users in the first year. In the fully adjusted model, the following were associated with the highest adjusted odds ratios (aORs) for long-term use: older age (≥75 years, aOR 4.59, 95% CI 4.48-4.70, $p < 0.001$; 65-74 years, aOR 3.77, 95% CI 3.68-3.85, $p < 0.001$, compared to <35 years), social deprivation (Townsend score quintile 5/most deprived, aOR 1.56, 95% CI 1.52-1.59, $p < 0.001$, compared to quintile 1/least deprived), fibromyalgia (aOR 1.81, 95% CI 1.49-2.19, $p < 0.001$), substance abuse (aOR 1.72, 95% CI 1.65-1.79, $p < 0.001$), suicide/self-harm (aOR 1.56, 95% CI 1.52-

1.61, p < 0.001), rheumatological conditions (aOR 1.53, 95% CI 1.48-1.58, p < 0.001), gabapentinoid use (aOR 2.52, 95% CI 2.43-2.61, p < 0.001), and MME/day at initiation (aOR 1.08, 95% CI 1.07-1.08, p < 0.001). After adjustment for case mix, 3 of the 10 UK regions (North West [16%], Yorkshire and the Humber [15%], and South West [15%]), 103 practices (25.6%), and 540 prescribers (3.5%) had a higher proportion of patients with long-term use compared to the population average. This study was limited to patients prescribed opioids in primary care and does not include opioids available over the counter or prescribed in hospitals or drug treatment centres.CONCLUSIONS: Of patients commencing opioids on very high MME/day (≥200), a high proportion stayed in the same category for a subsequent 2 years. Age, deprivation, prescribing factors, comorbidities such as fibromyalgia, rheumatological conditions, recent major surgery, and history of substance abuse, alcohol abuse, and self-harm/suicide were associated with long-term opioid use. Despite adjustment for case mix, variation across regions and especially practices and prescribers in high-risk prescribing was observed. Our findings support greater calls for action for reduction in practice and prescriber variation by promoting safe practice in opioid prescribing.

Institutions:

(Jani M) Centre for Epidemiology Versus Arthritis, Centre for Musculoskeletal Research, University of Manchester, Manchester, United Kingdom.; (Birlie Yimer B) Centre for Epidemiology Versus Arthritis, Centre for Musculoskeletal Research, University of Manchester, Manchester, United Kingdom.; (Sheppard T) Centre for Epidemiology Versus Arthritis, Centre for Musculoskeletal Research, University of Manchester, Manchester, United Kingdom.; (Lunt M) Centre for Epidemiology Versus Arthritis, Centre for Musculoskeletal Research, University of Manchester, Manchester, United Kingdom.; (Dixon WG) Centre for Epidemiology Versus Arthritis, Centre for Musculoskeletal Research, University of Manchester, Manchester, United Kingdom.

(Jani M) Centre for Epidemiology Versus Arthritis, Centre for Musculoskeletal Research, University of Manchester, Manchester, United Kingdom.; (Birlie Yimer B) Centre for Epidemiology Versus Arthritis, Centre for Musculoskeletal Research, University of Manchester, Manchester, United Kingdom.; (Sheppard T) Centre for Epidemiology Versus Arthritis, Centre for Musculoskeletal Research, University of Manchester, Manchester, United Kingdom.; (Lunt M) Centre for Epidemiology Versus Arthritis, Centre for Musculoskeletal Research, University of Manchester, Manchester, United Kingdom.; (Dixon WG) Centre for Epidemiology Versus Arthritis, Centre for Musculoskeletal Research, University of Manchester, Manchester, United Kingdom.

(Jani M) Centre for Epidemiology Versus Arthritis, Centre for Musculoskeletal Research, University of Manchester, Manchester, United Kingdom.; (Birlie Yimer B) Centre for Epidemiology Versus Arthritis, Centre for Musculoskeletal Research, University of Manchester, Manchester, United Kingdom.; (Sheppard T) Centre for Epidemiology Versus Arthritis, Centre for Musculoskeletal Research, University of Manchester, Manchester, United Kingdom.; (Lunt M) Centre for Epidemiology Versus Arthritis, Centre for Musculoskeletal Research, University of Manchester, Manchester, United Kingdom.; (Dixon WG) Centre for Epidemiology Versus Arthritis, Centre for Musculoskeletal Research, University of Manchester, Manchester, United Kingdom.

(Jani M) Centre for Epidemiology Versus Arthritis, Centre for Musculoskeletal Research, University of Manchester, Manchester, United Kingdom.; (Birlie Yimer B)

Centre for Epidemiology Versus Arthritis, Centre for Musculoskeletal Research, University of Manchester, Manchester, United Kingdom.; (Sheppard T) Centre for Epidemiology Versus Arthritis, Centre for Musculoskeletal Research, University of Manchester, Manchester, United Kingdom.; (Lunt M) Centre for Epidemiology Versus Arthritis, Centre for Musculoskeletal Research, University of Manchester, Manchester, United Kingdom.; (Dixon WG) Centre for Epidemiology Versus Arthritis, Centre for Musculoskeletal Research, University of Manchester, Manchester, United Kingdom.
(Jani M) Centre for Epidemiology Versus Arthritis, Centre for Musculoskeletal Research, University of Manchester, Manchester, United Kingdom.; (Birlie Yimer B) Centre for Epidemiology Versus Arthritis, Centre for Musculoskeletal Research, University of Manchester, Manchester, United Kingdom.; (Sheppard T) Centre for Epidemiology Versus Arthritis, Centre for Musculoskeletal Research, University of Manchester, Manchester, United Kingdom.; (Lunt M) Centre for Epidemiology Versus Arthritis, Centre for Musculoskeletal Research, University of Manchester, Manchester, United Kingdom.; (Dixon WG) Centre for Epidemiology Versus Arthritis, Centre for Musculoskeletal Research, University of Manchester, Manchester, United Kingdom.
Database: PubMed

22. Cancer Pain Treatment and Management: An Interprofessional Learning Module for Prelicensure Health Professional Students.

Author(s): Fishman SM; Copenhaver D; Mongoven JM; Lorenzen K; Schlingmann E; Young HM

Source: MedEdPORTAL : the journal of teaching and learning resources; ; vol. 16 ; p. 10953

Publication Type(s): Journal Article; Research Support, Non-U.S. Gov't

DOI: http://dx.doi.org/10.15766/mep_2374-8265.10953

ISSN: 2374-8265

Place of Publication: United States

PubMedID: 32934978

Accession Number: 32934978

Available at MedEdPORTAL : the journal of teaching and learning resources - from Unpaywall

Keywords: Subject Terms: *Cancer Pain Treatment; *Case-Based Learning; *Flipped Classroom; *Interprofessional; *Interprofessional Relations; *Online/Distance Education; *Opioids; *Pain Management; *Patient-Centered

Abstract: Introduction: The imperative of medicine is to treat suffering and to cure when possible. This learning module has been designed to expand providers' knowledge of how to sustain life, restore health, relieve suffering, and provide comfort for people who are experiencing cancer-induced pain. The module uses cancer pain as the context through which students can learn interprofessional, team-based, and person-centered approaches to delivery of care.Methods: Using the facilitator's guide, handouts, and other materials developed for this project, the module can be delivered as an in-person training session (approximately 120 minutes) for small groups of learners (teams of eight to 12 students drawn from

multiple health care professions or schools). Prelearning materials and postsession activities are included that can enhance the experience.Results: This module was developed and tested with two pilot programs that were evaluated with focus groups, direct observation, and a postsession survey completed by learners. Data demonstrated high approval of and appreciation for the content and structure of the module by both learners and facilitators.Discussion: Many learners work with other health care professionals in their clinical experiences but have not had opportunities to effectively work in interprofessional collaborative practice. This interprofessional education activity allows students from disparate health professions to work together to identify patient-centered treatment options through interprofessional collaborative teamwork in a classroom setting.

Institutions:

(Fishman SM) Professor and Fullerton Endowed Chair in Pain Medicine, Department of Anesthesiology and Pain Medicine, School of Medicine, University of California, Davis; Director, Center for Advancing Pain Relief, Betty Irene Moore School of Nursing and School of Medicine, University of California, Davis.; (Copenhaver D) Associate Professor and Chief, Department of Anesthesiology and Pain Medicine, Division of Pain Medicine, School of Medicine, University of California, Davis; Associate Director, Center for Advancing Pain Relief, Betty Irene Moore School of Nursing and School of Medicine, University of California, Davis.; (Mongoven JM) Associate Director of Operations, Family Caregiving Institute, Betty Irene Moore School of Nursing, University of California, Davis.; (Lorenzen K) Associate Director, Center for Advancing Pain Relief, Betty Irene Moore School of Nursing and School of Medicine, University of California, Davis.; (Schlingmann E) Research Associate, Center for Advancing Pain Relief, Betty Irene Moore School of Nursing and School of Medicine, University of California, Davis.; (Young HM) Dignity Health Dean's Chair for Nursing Leadership, Associate Vice Chancellor for Nursing, Dean, and Professor, Betty Irene Moore School of Nursing, University of California, Davis.
(Fishman SM) Professor and Fullerton Endowed Chair in Pain Medicine, Department of Anesthesiology and Pain Medicine, School of Medicine, University of California, Davis; Director, Center for Advancing Pain Relief, Betty Irene Moore School of Nursing and School of Medicine, University of California, Davis.; (Copenhaver D) Associate Professor and Chief, Department of Anesthesiology and Pain Medicine, Division of Pain Medicine, School of Medicine, University of California, Davis; Associate Director, Center for Advancing Pain Relief, Betty Irene Moore School of Nursing and School of Medicine, University of California, Davis.; (Mongoven JM) Associate Director of Operations, Family Caregiving Institute, Betty Irene Moore School of Nursing, University of California, Davis.; (Lorenzen K) Associate Director, Center for Advancing Pain Relief, Betty Irene Moore School of Nursing and School of Medicine, University of California, Davis.; (Schlingmann E) Research Associate, Center for Advancing Pain Relief, Betty Irene Moore School of Nursing and School of Medicine, University of California, Davis.; (Young HM) Dignity Health Dean's Chair for Nursing Leadership, Associate Vice Chancellor for Nursing, Dean, and Professor, Betty Irene Moore School of Nursing, University of California, Davis.
(Fishman SM) Professor and Fullerton Endowed Chair in Pain Medicine, Department of Anesthesiology and Pain Medicine, School of Medicine, University of California, Davis; Director, Center for Advancing Pain Relief, Betty Irene Moore School of Nursing and School of Medicine, University of California, Davis.; (Copenhaver D) Associate Professor and Chief, Department of Anesthesiology and Pain Medicine, Division of Pain Medicine, School of Medicine, University of California, Davis;

Associate Director, Center for Advancing Pain Relief, Betty Irene Moore School of
Nursing and School of Medicine, University of California, Davis.; (Mongoven JM)
Associate Director of Operations, Family Caregiving Institute, Betty Irene Moore
School of Nursing, University of California, Davis.; (Lorenzen K) Associate Director,
Center for Advancing Pain Relief, Betty Irene Moore School of Nursing and School of
Medicine, University of California, Davis.; (Schlingmann E) Research Associate,
Center for Advancing Pain Relief, Betty Irene Moore School of Nursing and School of
Medicine, University of California, Davis.; (Young HM) Dignity Health Dean's Chair
for Nursing Leadership, Associate Vice Chancellor for Nursing, Dean, and Professor,
Betty Irene Moore School of Nursing, University of California, Davis.
(Fishman SM) Professor and Fullerton Endowed Chair in Pain Medicine, Department
of Anesthesiology and Pain Medicine, School of Medicine, University of California,
Davis; Director, Center for Advancing Pain Relief, Betty Irene Moore School of
Nursing and School of Medicine, University of California, Davis.; (Copenhaver D)
Associate Professor and Chief, Department of Anesthesiology and Pain Medicine,
Division of Pain Medicine, School of Medicine, University of California, Davis;
Associate Director, Center for Advancing Pain Relief, Betty Irene Moore School of
Nursing and School of Medicine, University of California, Davis.; (Mongoven JM)
Associate Director of Operations, Family Caregiving Institute, Betty Irene Moore
School of Nursing, University of California, Davis.; (Lorenzen K) Associate Director,
Center for Advancing Pain Relief, Betty Irene Moore School of Nursing and School of
Medicine, University of California, Davis.; (Schlingmann E) Research Associate,
Center for Advancing Pain Relief, Betty Irene Moore School of Nursing and School of
Medicine, University of California, Davis.; (Young HM) Dignity Health Dean's Chair
for Nursing Leadership, Associate Vice Chancellor for Nursing, Dean, and Professor,
Betty Irene Moore School of Nursing, University of California, Davis.
(Fishman SM) Professor and Fullerton Endowed Chair in Pain Medicine, Department
of Anesthesiology and Pain Medicine, School of Medicine, University of California,
Davis; Director, Center for Advancing Pain Relief, Betty Irene Moore School of
Nursing and School of Medicine, University of California, Davis.; (Copenhaver D)
Associate Professor and Chief, Department of Anesthesiology and Pain Medicine,
Division of Pain Medicine, School of Medicine, University of California, Davis;
Associate Director, Center for Advancing Pain Relief, Betty Irene Moore School of
Nursing and School of Medicine, University of California, Davis.; (Mongoven JM)
Associate Director of Operations, Family Caregiving Institute, Betty Irene Moore
School of Nursing, University of California, Davis.; (Lorenzen K) Associate Director,
Center for Advancing Pain Relief, Betty Irene Moore School of Nursing and School of
Medicine, University of California, Davis.; (Schlingmann E) Research Associate,
Center for Advancing Pain Relief, Betty Irene Moore School of Nursing and School of
Medicine, University of California, Davis.; (Young HM) Dignity Health Dean's Chair
for Nursing Leadership, Associate Vice Chancellor for Nursing, Dean, and Professor,
Betty Irene Moore School of Nursing, University of California, Davis.
(Fishman SM) Professor and Fullerton Endowed Chair in Pain Medicine, Department
of Anesthesiology and Pain Medicine, School of Medicine, University of California,
Davis; Director, Center for Advancing Pain Relief, Betty Irene Moore School of
Nursing and School of Medicine, University of California, Davis.; (Copenhaver D)
Associate Professor and Chief, Department of Anesthesiology and Pain Medicine,
Division of Pain Medicine, School of Medicine, University of California, Davis;
Associate Director, Center for Advancing Pain Relief, Betty Irene Moore School of
Nursing and School of Medicine, University of California, Davis.; (Mongoven JM)

Associate Director of Operations, Family Caregiving Institute, Betty Irene Moore School of Nursing, University of California, Davis.; (Lorenzen K) Associate Director, Center for Advancing Pain Relief, Betty Irene Moore School of Nursing and School of Medicine, University of California, Davis.; (Schlingmann E) Research Associate, Center for Advancing Pain Relief, Betty Irene Moore School of Nursing and School of Medicine, University of California, Davis.; (Young HM) Dignity Health Dean's Chair for Nursing Leadership, Associate Vice Chancellor for Nursing, Dean, and Professor, Betty Irene Moore School of Nursing, University of California, Davis.
Database: PubMed

23. Chronic non-cancer pain management by nurses in specialist pain clinics.

Author(s): Fernández-Castillo RJ; Gil-García E; Vázquez-Santiago MS; Barrientos-Trigo S

Source: British journal of nursing (Mark Allen Publishing); Sep 2020; vol. 29 (no. 16); p. 954-959

Publication Date: Sep 2020

Publication Type(s): Journal Article

DOI: http://dx.doi.org/10.12968/bjon.2020.29.16.954

ISSN: 0966-0461

Place of Publication: England

PubMedID: 32901547

Accession Number: 32901547

Available at British journal of nursing (Mark Allen Publishing) - from MAG Online Library

Available at British journal of nursing (Mark Allen Publishing) - from EBSCO (CINAHL Complete)

Keywords: Subject Terms: Chronic pain; Pain clinics; Pain management; Patient care; Qualitative research

Abstract:BACKGROUND: Chronic non-cancer pain (CNCP) is one of the major causes of disability globally, and patients who suffer from it are a complex population, which makes it difficult to provide effective care. Specialist pain clinics and nursing professionals in them are the main care providers, but there is little research conducted in this field.AIM: To explore the attitudes and knowledge of nurses working in specialist pain clinics regarding care of CNCP patients.METHODS: Qualitative phenomenological approach. Sixteen semi-structured interviews were conducted in 2017 with nurses who worked in specialist pain clinics in six hospitals in southern Spain.RESULTS: Data analysis led to the formation of two categories, 'being trained and improving knowledge in CNCP' and 'the challenge of caring for patients with CNCP', and five subcategories.CONCLUSION: The need for care in CNCP is not covered by nurses in all the areas it requires. Lack of time, staffing issues, and specific training in this area makes it difficult to provide care. However, some areas for improvement are proposed, such as psychological interventions, group workshops, continuous training, and multidisciplinary teams.

Institutions:

(Fernández-Castillo RJ) Posgraduate Teaching Assistant, Department of Nursing, Faculty of Nursing, Physiotherapy and Podiatry, University of Seville, Spain.; (Gil-García E) Senior Lecturer in Nursing, Department of Nursing, Faculty of Nursing, Physiotherapy and Podiatry, University of Seville, Spain.; (Vázquez-Santiago MS) Senior Lecturer in Nursing, Department of Nursing, Faculty of Nursing, Physiotherapy and Podiatry, University of Seville, Spain.; (Barrientos-Trigo S) Assistant Professor in Nursing, Department of Nursing, Faculty of Nursing, Physiotherapy and Podiatry, University of Seville, Spain.
(Fernández-Castillo RJ) Posgraduate Teaching Assistant, Department of Nursing, Faculty of Nursing, Physiotherapy and Podiatry, University of Seville, Spain.; (Gil-García E) Senior Lecturer in Nursing, Department of Nursing, Faculty of Nursing, Physiotherapy and Podiatry, University of Seville, Spain.; (Vázquez-Santiago MS) Senior Lecturer in Nursing, Department of Nursing, Faculty of Nursing, Physiotherapy and Podiatry, University of Seville, Spain.; (Barrientos-Trigo S) Assistant Professor in Nursing, Department of Nursing, Faculty of Nursing, Physiotherapy and Podiatry, University of Seville, Spain.
(Fernández-Castillo RJ) Posgraduate Teaching Assistant, Department of Nursing, Faculty of Nursing, Physiotherapy and Podiatry, University of Seville, Spain.; (Gil-García E) Senior Lecturer in Nursing, Department of Nursing, Faculty of Nursing, Physiotherapy and Podiatry, University of Seville, Spain.; (Vázquez-Santiago MS) Senior Lecturer in Nursing, Department of Nursing, Faculty of Nursing, Physiotherapy and Podiatry, University of Seville, Spain.; (Barrientos-Trigo S) Assistant Professor in Nursing, Department of Nursing, Faculty of Nursing, Physiotherapy and Podiatry, University of Seville, Spain.
(Fernández-Castillo RJ) Posgraduate Teaching Assistant, Department of Nursing, Faculty of Nursing, Physiotherapy and Podiatry, University of Seville, Spain.; (Gil-García E) Senior Lecturer in Nursing, Department of Nursing, Faculty of Nursing, Physiotherapy and Podiatry, University of Seville, Spain.; (Vázquez-Santiago MS) Senior Lecturer in Nursing, Department of Nursing, Faculty of Nursing, Physiotherapy and Podiatry, University of Seville, Spain.; (Barrientos-Trigo S) Assistant Professor in Nursing, Department of Nursing, Faculty of Nursing, Physiotherapy and Podiatry, University of Seville, Spain.
Database: PubMed

24. Manifestation and parental assessment of children's cancer pain at home: An exploratory mixed-methods study.

Author(s): Parker R; Wiseman T; Twycross A; McKeever S

Source: Journal of clinical nursing; Nov 2020; vol. 29 (no. 21-22); p. 4128-4147

Publication Date: Nov 2020

Publication Type(s): Journal Article

DOI: http://dx.doi.org/10.1111/jocn.15442

ISSN: 1365-2702

Place of Publication: England

PubMedID: 32767621

Accession Number: 32767621

Available at Journal of clinical nursing - from Wiley Online Library Medicine and Nursing Collection 2020

Keywords: Subject Terms: cancer; mixed methods; nursing; oncology; pain; parenting; pediatric nursing

Abstract:AIMS AND OBJECTIVES: To describe pain manifestation in children with cancer at home and understand how parents assess this pain.BACKGROUND: Pain is experienced by children with cancer throughout their cancer journey. Short-term, and into survivorship, pain has negative physical and psychological consequences. Changes in treatment location mean children with cancer spend more time at home. Little is known about pain experienced by children at home or how parents assess this pain.DESIGN: A mixed-methods convergent parallel study was reported using STROBE.METHOD: Parents of children with cancer on active treatment were recruited from one tertiary cancer centre. Parental attitudes towards pain expression were assessed using surveys. Parents recorded their child's pain manifestation in pain diaries kept for one month. Interviews captured a deeper understanding of pain manifestation and how parents assess this pain at home. Integration occurred after each data collection method was analysed separately.RESULTS: Predominantly children were not in pain at home. However, most children experienced at least one episode of problematic pain over the pain diary period. Surveys showed parents held misconceptions regarding children's pain expression. Interviews diverge from surveys and suggest parents used a range of information sources to assess pain.CONCLUSION: Children with cancer may differ from one another in the manifestation of pain at home resulting in multiple pain trajectories. Parents of children with cancer are able to adequately assess their child's pain using information from multiple sources.RELEVANCE TO CLINICAL PRACTICE: It is not currently possible to predict which children will experience problematic pain at home, so all parents require pain management education prior to discharge. Teaching parents to use bundled approaches to pain assessment may accelerate their learning. Healthcare professionals may benefit from using multiple information sources to assess pain.

Institutions:
(Parker R) School of Health and Social Care, London South Bank University, London, UK.; (Wiseman T) The Royal Marsden NHS Foundation Trust, London, UK.; (Twycross A) The Open University, Milton Keynes, UK.; (McKeever S) Faculty of Health, Social Care and Education, Kingston University and St George's, University London, London, UK.
(Parker R) School of Health and Social Care, London South Bank University, London, UK.; (Wiseman T) The Royal Marsden NHS Foundation Trust, London, UK.; (Twycross A) The Open University, Milton Keynes, UK.; (McKeever S) Faculty of Health, Social Care and Education, Kingston University and St George's, University London, London, UK.
(Parker R) School of Health and Social Care, London South Bank University, London, UK.; (Wiseman T) The Royal Marsden NHS Foundation Trust, London, UK.; (Twycross A) The Open University, Milton Keynes, UK.; (McKeever S) Faculty of Health, Social Care and Education, Kingston University and St George's, University London, London, UK.
(Parker R) School of Health and Social Care, London South Bank University, London, UK.; (Wiseman T) The Royal Marsden NHS Foundation Trust, London, UK.; (Twycross A) The Open University, Milton Keynes, UK.; (McKeever S) Faculty of

Health, Social Care and Education, Kingston University and St George's, University London, London, UK.
Database: PubMed

25. Breakthrough cancer pain treatment in Spain: physicians' perception of current opioids utilization and prescription.

Author(s): Villegas Estévez F; López Alarcón MD; Alonso Babarro A; Olay Gayoso L; de Castro J; Lería-Gelabert M; Melogno-Klinkas M

Source: Current medical research and opinion; 2020; vol. 36 (no. 8); p. 1383-1391

Publication Date: 2020

Publication Type(s): Journal Article; Observational Study; Research Support, Non-U.S. Gov't

DOI: http://dx.doi.org/10.1080/03007995.2020.1775073

ISSN: 1473-4877

Place of Publication: England

PubMedID: 32453602

Accession Number: 32453602

Keywords: Subject Terms: *Breakthrough cancer pain; *barriers; *misuse; *opioids

Abstract: Objectives: Multiple reasons for suboptimal treatment of breakthrough cancer pain (BTcP) have been reported in the literature. We aimed to ascertain the perception of physicians on the potential inappropriate use and prescription of rapid-onset opioids (ROOs) for breakthrough cancer pain (BTcP) and the causes thereof.Methods: Observational study based on an online survey addressed to doctors from different specialties (radiation oncology, medical oncology, anesthesia, palliative care and general practitioners) with experience in the management of BTcP in the Spanish public health setting.Results: A total of 114 eligible specialists mainly from radiation oncology (37.7%), medical oncology (24.6%) and pain units (18.4%) participated in the study. Most agreed on important aspects of BTcP management, such as their preference for ROOs or the need for early follow-up after treatment initiation. However, their answers revealed a lack of standardization of BTcP diagnosis. Half of respondents believed that their BTcP patients might misuse ROOs. Physicians polled believed that lack of training in pain management (71.9%) and inadequate BTcP diagnosis and evaluation (66.7%) were the greatest obstacles for prescribing opioids. Specialists also thought that they do not provide the necessary information to patients (51.8%) and caregivers (57.9%) to guarantee the correct use of these drugs.Conclusions: These results are of utmost importance as they highlight the need to increase physicians' awareness of BTcP and its management and the need to improve communication with patients and their caregivers. Our findings also indicate the need for future research on the possible misuse of opioids in BTcP patients and its causes.

Institutions:
(Villegas Estévez F) Pain Unit, Consorcio Hospital Provincial de Castellón, Castellón, Spain.; (López Alarcón MD) Pain Unit, Hospital General Universitario de Valencia, Valencia, Spain.; (Alonso Babarro A) Palliative Care Unit, Hospital Universitario la Paz, Madrid, Spain.; (Olay Gayoso L) Radiation Oncology Department, Hospital Universitario Central de Asturias, Oviedo, Spain.; (de Castro J)

Medical Oncology Department, Hospital Universitario la Paz, Madrid, Spain.; (Lería-Gelabert M) Medical Department, Mylan, Madrid, Spain.; (Melogno-Klinkas M) Medical Department, Mylan, Madrid, Spain.
(Villegas Estévez F) Pain Unit, Consorcio Hospital Provincial de Castellón, Castellón, Spain.; (López Alarcón MD) Pain Unit, Hospital General Universitario de Valencia, Valencia, Spain.; (Alonso Babarro A) Palliative Care Unit, Hospital Universitario la Paz, Madrid, Spain.; (Olay Gayoso L) Radiation Oncology Department, Hospital Universitario Central de Asturias, Oviedo, Spain.; (de Castro J) Medical Oncology Department, Hospital Universitario la Paz, Madrid, Spain.; (Lería-Gelabert M) Medical Department, Mylan, Madrid, Spain.; (Melogno-Klinkas M) Medical Department, Mylan, Madrid, Spain.
(Villegas Estévez F) Pain Unit, Consorcio Hospital Provincial de Castellón, Castellón, Spain.; (López Alarcón MD) Pain Unit, Hospital General Universitario de Valencia, Valencia, Spain.; (Alonso Babarro A) Palliative Care Unit, Hospital Universitario la Paz, Madrid, Spain.; (Olay Gayoso L) Radiation Oncology Department, Hospital Universitario Central de Asturias, Oviedo, Spain.; (de Castro J) Medical Oncology Department, Hospital Universitario la Paz, Madrid, Spain.; (Lería-Gelabert M) Medical Department, Mylan, Madrid, Spain.; (Melogno-Klinkas M) Medical Department, Mylan, Madrid, Spain.
(Villegas Estévez F) Pain Unit, Consorcio Hospital Provincial de Castellón, Castellón, Spain.; (López Alarcón MD) Pain Unit, Hospital General Universitario de Valencia, Valencia, Spain.; (Alonso Babarro A) Palliative Care Unit, Hospital Universitario la Paz, Madrid, Spain.; (Olay Gayoso L) Radiation Oncology Department, Hospital Universitario Central de Asturias, Oviedo, Spain.; (de Castro J) Medical Oncology Department, Hospital Universitario la Paz, Madrid, Spain.; (Lería-Gelabert M) Medical Department, Mylan, Madrid, Spain.; (Melogno-Klinkas M) Medical Department, Mylan, Madrid, Spain.
(Villegas Estévez F) Pain Unit, Consorcio Hospital Provincial de Castellón, Castellón, Spain.; (López Alarcón MD) Pain Unit, Hospital General Universitario de Valencia, Valencia, Spain.; (Alonso Babarro A) Palliative Care Unit, Hospital Universitario la Paz, Madrid, Spain.; (Olay Gayoso L) Radiation Oncology Department, Hospital Universitario Central de Asturias, Oviedo, Spain.; (de Castro J) Medical Oncology Department, Hospital Universitario la Paz, Madrid, Spain.; (Lería-Gelabert M) Medical Department, Mylan, Madrid, Spain.; (Melogno-Klinkas M) Medical Department, Mylan, Madrid, Spain.
(Villegas Estévez F) Pain Unit, Consorcio Hospital Provincial de Castellón, Castellón, Spain.; (López Alarcón MD) Pain Unit, Hospital General Universitario de Valencia, Valencia, Spain.; (Alonso Babarro A) Palliative Care Unit, Hospital Universitario la Paz, Madrid, Spain.; (Olay Gayoso L) Radiation Oncology Department, Hospital Universitario Central de Asturias, Oviedo, Spain.; (de Castro J) Medical Oncology Department, Hospital Universitario la Paz, Madrid, Spain.; (Lería-Gelabert M) Medical Department, Mylan, Madrid, Spain.; (Melogno-Klinkas M) Medical Department, Mylan, Madrid, Spain.
(Villegas Estévez F) Pain Unit, Consorcio Hospital Provincial de Castellón, Castellón, Spain.; (López Alarcón MD) Pain Unit, Hospital General Universitario de Valencia, Valencia, Spain.; (Alonso Babarro A) Palliative Care Unit, Hospital Universitario la Paz, Madrid, Spain.; (Olay Gayoso L) Radiation Oncology Department, Hospital Universitario Central de Asturias, Oviedo, Spain.; (de Castro J) Medical Oncology Department, Hospital Universitario la Paz, Madrid, Spain.; (Lería-

Gelabert M) Medical Department, Mylan, Madrid, Spain.; (Melogno-Klinkas M) Medical Department, Mylan, Madrid, Spain.
Database: PubMed

26. Opioid Induced Hyperalgesia, a Research Phenomenon or a Clinical Reality? Results of a Canadian Survey.

Author(s): Vargas-Schaffer G; Paquet S; Neron A; Cogan J

Source: Journal of personalized medicine; Apr 2020; vol. 10 (no. 2)

Publication Date: Apr 2020

Publication Type(s): Journal Article

DOI: http://dx.doi.org/10.3390/jpm10020027

ISSN: 2075-4426

Place of Publication: Switzerland

PubMedID: 32326188

Accession Number: 32326188

Available at Journal of personalized medicine - from Europe PubMed Central - Open Access

Available at Journal of personalized medicine - from Unpaywall

Keywords: Subject Terms: acute pain; cancer pain; chronic non-cancer pain; opioid induced hyperalgesia; opioid tolerance

Abstract:BACKGROUND: Very little is known regarding the prevalence of opioid induced hyperalgesia (OIH) in day to day medical practice. The aim of this study was to evaluate the physician's perception of the prevalence of OIH within their practice, and to assess the level of physician's knowledge with respect to the identification and treatment of this problem.METHODS: An electronic questionnaire was distributed to physicians who work in anesthesiology, chronic pain, and/or palliative care in Canada.RESULTS: Of the 462 responses received, most were from male (69%) anesthesiologists (89.6%), in the age range of 36 to 64 years old (79.8%). In this study, the suspected prevalence of OIH using the average number of patients treated per year with opioids was 0.002% per patient per physician practice year for acute pain, and 0.01% per patient per physician practice year for chronic pain. Most physicians (70.2%) did not use clinical tests to help make a diagnosis of OIH. The treatment modalities most frequently used were the addition of an NMDA antagonist, combined with lowering the opioid doses and using opioid rotation.CONCLUSIONS: The perceived prevalence of OIH in clinical practice is a relatively rare phenomenon. Furthermore, more than half of physicians did not use a clinical test to confirm the diagnosis of OIH. The two main treatment modalities used were NMDA antagonists and opioid rotation. The criteria for the diagnosis of OIH still need to be accurately defined.

Institutions:
(Vargas-Schaffer G) Pain Center. Centre Universitaire de l'Université de Montréal, CHUM, Montreal, QC H2X 3E4, Canada.; (Paquet S) Pain Center. Centre Universitaire de l'Université de Montréal, CHUM, Montreal, QC H2X 3E4, Canada.; (Neron A) Pain Center. Centre Universitaire de l'Université de Montréal, CHUM,

Montreal, QC H2X 3E4, Canada.; (Cogan J) Montreal Hearth Institute, Montreal, QC H1T 1C8, Canada.
(Vargas-Schaffer G) Pain Center. Centre Universitaire de l'Université de Montréal, CHUM, Montreal, QC H2X 3E4, Canada.; (Paquet S) Pain Center. Centre Universitaire de l'Université de Montréal, CHUM, Montreal, QC H2X 3E4, Canada.; (Neron A) Pain Center. Centre Universitaire de l'Université de Montréal, CHUM, Montreal, QC H2X 3E4, Canada.; (Cogan J) Montreal Hearth Institute, Montreal, QC H1T 1C8, Canada.
(Vargas-Schaffer G) Pain Center. Centre Universitaire de l'Université de Montréal, CHUM, Montreal, QC H2X 3E4, Canada.; (Paquet S) Pain Center. Centre Universitaire de l'Université de Montréal, CHUM, Montreal, QC H2X 3E4, Canada.; (Neron A) Pain Center. Centre Universitaire de l'Université de Montréal, CHUM, Montreal, QC H2X 3E4, Canada.; (Cogan J) Montreal Hearth Institute, Montreal, QC H1T 1C8, Canada.
(Vargas-Schaffer G) Pain Center. Centre Universitaire de l'Université de Montréal, CHUM, Montreal, QC H2X 3E4, Canada.; (Paquet S) Pain Center. Centre Universitaire de l'Université de Montréal, CHUM, Montreal, QC H2X 3E4, Canada.; (Neron A) Pain Center. Centre Universitaire de l'Université de Montréal, CHUM, Montreal, QC H2X 3E4, Canada.; (Cogan J) Montreal Hearth Institute, Montreal, QC H1T 1C8, Canada.
Database: PubMed

27. Knowledge and Attitudes of Chinese Oncology Nurses Regarding Cancer Pain Management-a Cross-Sectional Study.

Author(s): Yu W; Li D; Lu Y; Yang H; Ma X

Source: Journal of cancer education : the official journal of the American Association for Cancer Education; Apr 2020

Publication Date: Apr 2020

Publication Type(s): Journal Article

DOI: http://dx.doi.org/10.1007/s13187-020-01743-z

ISSN: 1543-0154

Place of Publication: England

PubMedID: 32318978

Accession Number: 32318978

Available at Journal of cancer education : the official journal of the American Association for Cancer Education - from EBSCO (MEDLINE Complete)

Keywords: Subject Terms: Attitude; Knowledge; Management; Neoplasms; Nursing; Pain

Abstract:OBJECTIVE: To measure the knowledge and attitudes of Chinese oncology nurses regarding cancer pain management and explore related factors.METHODS: A cross-sectional survey was conducted. A convenience sample of 505 Chinese oncology nurses from 20 provinces, 4 municipalities, and 4 autonomous regions was recruited between April and October 2018. A Chinese version of the Knowledge and Attitudes Survey Regarding Pain (KASRP) was used. Socio-demographic characteristics were also measured by a multi-item questionnaire.RESULTS: The mean accuracy of KASRP in all participants was

56.11% ± 11.05%. According to univariate analysis, oncology nurses' cancer pain knowledge and attitudes were positively correlated with age, region, education level, years of oncology nursing, clinical practice of cancer pain management, average monthly number of patients with cancer pain, and the experience of cancer pain-related training. The results of multiple linear regression revealed that nurses' age, education level, experience of cancer pain-related training and clinical practice of cancer pain management were independent influencing factors (all $p < 0.05$).CONCLUSIONS: Most Chinese oncology nurses had misconceptions about cancer pain management and lacked relevant knowledge. We should assign importance to targeted cancer pain training and develop training methods that closely relate to clinical practices.

Institutions:

(Yu W) Nursing Department, Key laboratory of Carcinogenesis and Translational Research (Ministry of Education), Peking University Cancer Hospital & Institute, 52 Fucheng road, Haidian district, Beijing, 100142, China.; (Li D) Department of Radiotherapy, The Fifth Medical Center, PLA General Hospital, 100 West Fourth Ring Road, Fengtai District, Beijing, 100039, China.; (Lu Y) Nursing Department, Key laboratory of Carcinogenesis and Translational Research (Ministry of Education), Peking University Cancer Hospital & Institute, 52 Fucheng road, Haidian district, Beijing, 100142, China. lu_yuhan@sina.com.; (Yang H) Nursing Department, Key laboratory of Carcinogenesis and Translational Research (Ministry of Education), Peking University Cancer Hospital & Institute, 52 Fucheng road, Haidian district, Beijing, 100142, China.; (Ma X) Nursing Department, Key laboratory of Carcinogenesis and Translational Research (Ministry of Education), Peking University Cancer Hospital & Institute, 52 Fucheng road, Haidian district, Beijing, 100142, China.

(Yu W) Nursing Department, Key laboratory of Carcinogenesis and Translational Research (Ministry of Education), Peking University Cancer Hospital & Institute, 52 Fucheng road, Haidian district, Beijing, 100142, China.; (Li D) Department of Radiotherapy, The Fifth Medical Center, PLA General Hospital, 100 West Fourth Ring Road, Fengtai District, Beijing, 100039, China.; (Lu Y) Nursing Department, Key laboratory of Carcinogenesis and Translational Research (Ministry of Education), Peking University Cancer Hospital & Institute, 52 Fucheng road, Haidian district, Beijing, 100142, China. lu_yuhan@sina.com.; (Yang H) Nursing Department, Key laboratory of Carcinogenesis and Translational Research (Ministry of Education), Peking University Cancer Hospital & Institute, 52 Fucheng road, Haidian district, Beijing, 100142, China.; (Ma X) Nursing Department, Key laboratory of Carcinogenesis and Translational Research (Ministry of Education), Peking University Cancer Hospital & Institute, 52 Fucheng road, Haidian district, Beijing, 100142, China.

(Yu W) Nursing Department, Key laboratory of Carcinogenesis and Translational Research (Ministry of Education), Peking University Cancer Hospital & Institute, 52 Fucheng road, Haidian district, Beijing, 100142, China.; (Li D) Department of Radiotherapy, The Fifth Medical Center, PLA General Hospital, 100 West Fourth Ring Road, Fengtai District, Beijing, 100039, China.; (Lu Y) Nursing Department, Key laboratory of Carcinogenesis and Translational Research (Ministry of Education), Peking University Cancer Hospital & Institute, 52 Fucheng road, Haidian district, Beijing, 100142, China. lu_yuhan@sina.com.; (Yang H) Nursing Department, Key laboratory of Carcinogenesis and Translational Research (Ministry of Education), Peking University Cancer Hospital & Institute, 52 Fucheng road, Haidian

district, Beijing, 100142, China.; (Ma X) Nursing Department, Key laboratory of Carcinogenesis and Translational Research (Ministry of Education), Peking University Cancer Hospital & Institute, 52 Fucheng road, Haidian district, Beijing, 100142, China.
(Yu W) Nursing Department, Key laboratory of Carcinogenesis and Translational Research (Ministry of Education), Peking University Cancer Hospital & Institute, 52 Fucheng road, Haidian district, Beijing, 100142, China.; (Li D) Department of Radiotherapy, The Fifth Medical Center, PLA General Hospital, 100 West Fourth Ring Road, Fengtai District, Beijing, 100039, China.; (Lu Y) Nursing Department, Key laboratory of Carcinogenesis and Translational Research (Ministry of Education), Peking University Cancer Hospital & Institute, 52 Fucheng road, Haidian district, Beijing, 100142, China. lu_yuhan@sina.com.; (Yang H) Nursing Department, Key laboratory of Carcinogenesis and Translational Research (Ministry of Education), Peking University Cancer Hospital & Institute, 52 Fucheng road, Haidian district, Beijing, 100142, China.; (Ma X) Nursing Department, Key laboratory of Carcinogenesis and Translational Research (Ministry of Education), Peking University Cancer Hospital & Institute, 52 Fucheng road, Haidian district, Beijing, 100142, China.
(Yu W) Nursing Department, Key laboratory of Carcinogenesis and Translational Research (Ministry of Education), Peking University Cancer Hospital & Institute, 52 Fucheng road, Haidian district, Beijing, 100142, China.; (Li D) Department of Radiotherapy, The Fifth Medical Center, PLA General Hospital, 100 West Fourth Ring Road, Fengtai District, Beijing, 100039, China.; (Lu Y) Nursing Department, Key laboratory of Carcinogenesis and Translational Research (Ministry of Education), Peking University Cancer Hospital & Institute, 52 Fucheng road, Haidian district, Beijing, 100142, China. lu_yuhan@sina.com.; (Yang H) Nursing Department, Key laboratory of Carcinogenesis and Translational Research (Ministry of Education), Peking University Cancer Hospital & Institute, 52 Fucheng road, Haidian district, Beijing, 100142, China.; (Ma X) Nursing Department, Key laboratory of Carcinogenesis and Translational Research (Ministry of Education), Peking University Cancer Hospital & Institute, 52 Fucheng road, Haidian district, Beijing, 100142, China.
Database: PubMed

28. Morphine use for cancer pain: A strong analgesic used only at the end of life? A qualitative study on attitudes and perceptions of morphine in patients with advanced cancer and their caregivers.

Author(s): Ho JFV; Yaakup H; Low GSH; Wong SL; Tho LM; Tan SB

Source: Palliative medicine; 2020; vol. 34 (no. 5); p. 619-629

Publication Date: 2020

Publication Type(s): Journal Article; Research Support, Non-U.S. Gov't

DOI: http://dx.doi.org/10.1177/0269216320904905

ISSN: 1477-030X

Place of Publication: England

PubMedID: 32103707

Accession Number: 32103707

Available at Palliative Medicine - from Unpaywall

Keywords: Subject Terms: *Morphine; *attitude; *cancer pain; *palliative care; *perception; *qualitative research

Abstract:BACKGROUND: The prevalence of undertreated cancer pain remains high. Suboptimal pain control affects quality of life and results in psychological and emotional distress. Barriers to adequate pain control include fear of opioid dependence and its side effects.AIM: To investigate the attitudes and perceptions of morphine use in cancer pain in advanced cancer patients and their caregivers and to examine the influence of caregivers' attitudes and perceptions on patients' acceptance of morphine.DESIGN: Qualitative study involving semi-structured individual interviews transcribed verbatim and analyzed thematically.SETTING/PARTICIPANTS: A total of 18 adult opioid-naïve patients with advanced cancer and 13 caregivers (n = 31) were recruited at a private tertiary hospital via convenience sampling.RESULTS: Attitudes and perceptions of morphine were influenced by previous experiences. Prevalent themes were similar in both groups, including perceptions that morphine was a strong analgesic that reduced suffering, but associated with end-stage illness and dependence. Most participants were open to future morphine use for comfort and effective pain control. Trust in doctors' recommendations was also an important factor. However, many preferred morphine as a last resort because of concerns about side effects and dependence, and the perception that morphine was only used at the terminal stage. Caregivers' attitudes toward morphine did not affect patients' acceptance of morphine use.CONCLUSION: Most participants were open to future morphine use despite negative perceptions as they prioritized optimal pain control and reduction of suffering. Focused education programs addressing morphine misperceptions might increase patient and caregiver acceptance of opioid analgesics and improve cancer pain control.

Institutions:
(Ho JFV) Supportive and Palliative Care Service, Sunway Medical Centre, Bandar Sunway, Malaysia.; (Yaakup H) Supportive and Palliative Care Service, Sunway Medical Centre, Bandar Sunway, Malaysia.; (Low GSH) Supportive and Palliative Care Service, Sunway Medical Centre, Bandar Sunway, Malaysia.; (Wong SL) Department of Oncology, Sunway Medical Centre, Bandar Sunway, Malaysia.; (Tho LM) Department of Oncology, Sunway Medical Centre, Bandar Sunway, Malaysia.; (Tan SB) Department of Palliative Medicine, University of Malaya Medical Centre, Kuala Lumpur, Malaysia.
(Ho JFV) Supportive and Palliative Care Service, Sunway Medical Centre, Bandar Sunway, Malaysia.; (Yaakup H) Supportive and Palliative Care Service, Sunway Medical Centre, Bandar Sunway, Malaysia.; (Low GSH) Supportive and Palliative Care Service, Sunway Medical Centre, Bandar Sunway, Malaysia.; (Wong SL) Department of Oncology, Sunway Medical Centre, Bandar Sunway, Malaysia.; (Tho LM) Department of Oncology, Sunway Medical Centre, Bandar Sunway, Malaysia.; (Tan SB) Department of Palliative Medicine, University of Malaya Medical Centre, Kuala Lumpur, Malaysia.
(Ho JFV) Supportive and Palliative Care Service, Sunway Medical Centre, Bandar Sunway, Malaysia.; (Yaakup H) Supportive and Palliative Care Service, Sunway Medical Centre, Bandar Sunway, Malaysia.; (Low GSH) Supportive and Palliative Care Service, Sunway Medical Centre, Bandar Sunway, Malaysia.; (Wong SL) Department of Oncology, Sunway Medical Centre, Bandar Sunway, Malaysia.; (Tho

LM) Department of Oncology, Sunway Medical Centre, Bandar Sunway, Malaysia.; (Tan SB) Department of Palliative Medicine, University of Malaya Medical Centre, Kuala Lumpur, Malaysia.
(Ho JFV) Supportive and Palliative Care Service, Sunway Medical Centre, Bandar Sunway, Malaysia.; (Yaakup H) Supportive and Palliative Care Service, Sunway Medical Centre, Bandar Sunway, Malaysia.; (Low GSH) Supportive and Palliative Care Service, Sunway Medical Centre, Bandar Sunway, Malaysia.; (Wong SL) Department of Oncology, Sunway Medical Centre, Bandar Sunway, Malaysia.; (Tho LM) Department of Oncology, Sunway Medical Centre, Bandar Sunway, Malaysia.; (Tan SB) Department of Palliative Medicine, University of Malaya Medical Centre, Kuala Lumpur, Malaysia.
(Ho JFV) Supportive and Palliative Care Service, Sunway Medical Centre, Bandar Sunway, Malaysia.; (Yaakup H) Supportive and Palliative Care Service, Sunway Medical Centre, Bandar Sunway, Malaysia.; (Low GSH) Supportive and Palliative Care Service, Sunway Medical Centre, Bandar Sunway, Malaysia.; (Wong SL) Department of Oncology, Sunway Medical Centre, Bandar Sunway, Malaysia.; (Tho LM) Department of Oncology, Sunway Medical Centre, Bandar Sunway, Malaysia.; (Tan SB) Department of Palliative Medicine, University of Malaya Medical Centre, Kuala Lumpur, Malaysia.
(Ho JFV) Supportive and Palliative Care Service, Sunway Medical Centre, Bandar Sunway, Malaysia.; (Yaakup H) Supportive and Palliative Care Service, Sunway Medical Centre, Bandar Sunway, Malaysia.; (Low GSH) Supportive and Palliative Care Service, Sunway Medical Centre, Bandar Sunway, Malaysia.; (Wong SL) Department of Oncology, Sunway Medical Centre, Bandar Sunway, Malaysia.; (Tho LM) Department of Oncology, Sunway Medical Centre, Bandar Sunway, Malaysia.; (Tan SB) Department of Palliative Medicine, University of Malaya Medical Centre, Kuala Lumpur, Malaysia.
Database: PubMed

29. Law, Culture, and Fear: A Qualitative Study of Health Professionals' Perceptions of Narcotic Use Related to Cancer Pain.

Author(s): Al-Masri D; Wilbur K; Elazzazy S; Hassan AA; Wilby KJ

Source: Journal of pain & palliative care pharmacotherapy; Jun 2020; vol. 34 (no. 2); p. 55-62

Publication Date: Jun 2020

Publication Type(s): Journal Article

DOI: http://dx.doi.org/10.1080/15360288.2019.1704340

ISSN: 1536-0539

Place of Publication: England

PubMedID: 32091944

Accession Number: 32091944

Available at Journal of pain & palliative care pharmacotherapy - from EBSCO (MEDLINE Complete)

Keywords: Subject Terms: Cancer pain; Middle East; narcotic utilization; opioid

Abstract: Studies have shown barriers to appropriate narcotic use in the Middle East have negatively impacted patient outcomes. This study aimed to explore health

professionals' perspectives regarding opioid use for cancer patients in Qatar. Eight focus groups were conducted with physicians, pharmacists, and nurses. An eight-question topic guide framed discussions and targeted contextual barriers and cultural beliefs. Focus groups were audio-recorded and transcribed verbatim. Thematic analysis was used to identify the following themes: narcotic use process, patient-related factors, and healthcare professional-related factors. Laws and regulations were identified as major barriers to appropriate narcotic access, prescribing, and administration. Government-imposed restrictions on permitted dispensed quantities and associated paperwork impeded continuity of patient care and pain relief. The influence of a patient's culture underpinned patient-related barriers, including fear of addiction and family members discouraging opioid use. Fear of prescribing for patient addiction and accusation of inappropriate prescribing by authorities were identified as health professional-related barriers. Facilitators included patient and provider education, as well as the availability of specialized teams to assess and treat cancer-related pain. Findings show narcotic utilization is not simply influenced by a single factor or subset of factors but by a multitude of factors that can be both independent and interrelated.

Institutions:
(Al-Masri D) Dania Al-Masri, BSc(Pharm), MSc(Pharm), is with the College of Pharmacy, Qatar University, Doha, Qatar; Kerry Wilbur, BSc(Pharm), ACPR, PharmD, MPH, is with the Faculty of Pharmaceutical Sciences, University of British Columbia, Vancouver, Canada; Shereen Elazzazy, BSc(Pharm), PharmD, is with the Department of Pharmacy - Clinical Services, National Center for Cancer Care and Research, Hamad Medical Corporation, Doha, Qatar; Azza A. Hassan, MB BCh, MSc, MD, is with the Supportive & Palliative Care Unit, National Center for Cancer Care and Research, Hamad Medical Corporation, Doha, Qatar; Kyle J. Wilby, BSP, ACPR, PharmD, PhD, is with the School of Pharmacy, University of Otago, Dunedin, New Zealand.; (Wilbur K) Dania Al-Masri, BSc(Pharm), MSc(Pharm), is with the College of Pharmacy, Qatar University, Doha, Qatar; Kerry Wilbur, BSc(Pharm), ACPR, PharmD, MPH, is with the Faculty of Pharmaceutical Sciences, University of British Columbia, Vancouver, Canada; Shereen Elazzazy, BSc(Pharm), PharmD, is with the Department of Pharmacy - Clinical Services, National Center for Cancer Care and Research, Hamad Medical Corporation, Doha, Qatar; Azza A. Hassan, MB BCh, MSc, MD, is with the Supportive & Palliative Care Unit, National Center for Cancer Care and Research, Hamad Medical Corporation, Doha, Qatar; Kyle J. Wilby, BSP, ACPR, PharmD, PhD, is with the School of Pharmacy, University of Otago, Dunedin, New Zealand.; (Elazzazy S) Dania Al-Masri, BSc(Pharm), MSc(Pharm), is with the College of Pharmacy, Qatar University, Doha, Qatar; Kerry Wilbur, BSc(Pharm), ACPR, PharmD, MPH, is with the Faculty of Pharmaceutical Sciences, University of British Columbia, Vancouver, Canada; Shereen Elazzazy, BSc(Pharm), PharmD, is with the Department of Pharmacy - Clinical Services, National Center for Cancer Care and Research, Hamad Medical Corporation, Doha, Qatar; Azza A. Hassan, MB BCh, MSc, MD, is with the Supportive & Palliative Care Unit, National Center for Cancer Care and Research, Hamad Medical Corporation, Doha, Qatar; Kyle J. Wilby, BSP, ACPR, PharmD, PhD, is with the School of Pharmacy, University of Otago, Dunedin, New Zealand.; (Hassan AA) Dania Al-Masri, BSc(Pharm), MSc(Pharm), is with the College of Pharmacy, Qatar University, Doha, Qatar; Kerry Wilbur, BSc(Pharm), ACPR, PharmD, MPH, is with the Faculty of Pharmaceutical Sciences, University of British Columbia, Vancouver, Canada; Shereen Elazzazy, BSc(Pharm), PharmD, is with the Department of Pharmacy -

Clinical Services, National Center for Cancer Care and Research, Hamad Medical Corporation, Doha, Qatar; Azza A. Hassan, MB BCh, MSc, MD, is with the Supportive & Palliative Care Unit, National Center for Cancer Care and Research, Hamad Medical Corporation, Doha, Qatar; Kyle J. Wilby, BSP, ACPR, PharmD, PhD, is with the School of Pharmacy, University of Otago, Dunedin, New Zealand.; (Wilby KJ) Dania Al-Masri, BSc(Pharm), MSc(Pharm), is with the College of Pharmacy, Qatar University, Doha, Qatar; Kerry Wilbur, BSc(Pharm), ACPR, PharmD, MPH, is with the Faculty of Pharmaceutical Sciences, University of British Columbia, Vancouver, Canada; Shereen Elazzazy, BSc(Pharm), PharmD, is with the Department of Pharmacy - Clinical Services, National Center for Cancer Care and Research, Hamad Medical Corporation, Doha, Qatar; Azza A. Hassan, MB BCh, MSc, MD, is with the Supportive & Palliative Care Unit, National Center for Cancer Care and Research, Hamad Medical Corporation, Doha, Qatar; Kyle J. Wilby, BSP, ACPR, PharmD, PhD, is with the School of Pharmacy, University of Otago, Dunedin, New Zealand.
(Al-Masri D) Dania Al-Masri, BSc(Pharm), MSc(Pharm), is with the College of Pharmacy, Qatar University, Doha, Qatar; Kerry Wilbur, BSc(Pharm), ACPR, PharmD, MPH, is with the Faculty of Pharmaceutical Sciences, University of British Columbia, Vancouver, Canada; Shereen Elazzazy, BSc(Pharm), PharmD, is with the Department of Pharmacy - Clinical Services, National Center for Cancer Care and Research, Hamad Medical Corporation, Doha, Qatar; Azza A. Hassan, MB BCh, MSc, MD, is with the Supportive & Palliative Care Unit, National Center for Cancer Care and Research, Hamad Medical Corporation, Doha, Qatar; Kyle J. Wilby, BSP, ACPR, PharmD, PhD, is with the School of Pharmacy, University of Otago, Dunedin, New Zealand.; (Wilbur K) Dania Al-Masri, BSc(Pharm), MSc(Pharm), is with the College of Pharmacy, Qatar University, Doha, Qatar; Kerry Wilbur, BSc(Pharm), ACPR, PharmD, MPH, is with the Faculty of Pharmaceutical Sciences, University of British Columbia, Vancouver, Canada; Shereen Elazzazy, BSc(Pharm), PharmD, is with the Department of Pharmacy - Clinical Services, National Center for Cancer Care and Research, Hamad Medical Corporation, Doha, Qatar; Azza A. Hassan, MB BCh, MSc, MD, is with the Supportive & Palliative Care Unit, National Center for Cancer Care and Research, Hamad Medical Corporation, Doha, Qatar; Kyle J. Wilby, BSP, ACPR, PharmD, PhD, is with the School of Pharmacy, University of Otago, Dunedin, New Zealand.; (Elazzazy S) Dania Al-Masri, BSc(Pharm), MSc(Pharm), is with the College of Pharmacy, Qatar University, Doha, Qatar; Kerry Wilbur, BSc(Pharm), ACPR, PharmD, MPH, is with the Faculty of Pharmaceutical Sciences, University of British Columbia, Vancouver, Canada; Shereen Elazzazy, BSc(Pharm), PharmD, is with the Department of Pharmacy - Clinical Services, National Center for Cancer Care and Research, Hamad Medical Corporation, Doha, Qatar; Azza A. Hassan, MB BCh, MSc, MD, is with the Supportive & Palliative Care Unit, National Center for Cancer Care and Research, Hamad Medical Corporation, Doha, Qatar; Kyle J. Wilby, BSP, ACPR, PharmD, PhD, is with the School of Pharmacy, University of Otago, Dunedin, New Zealand.; (Hassan AA) Dania Al-Masri, BSc(Pharm), MSc(Pharm), is with the College of Pharmacy, Qatar University, Doha, Qatar; Kerry Wilbur, BSc(Pharm), ACPR, PharmD, MPH, is with the Faculty of Pharmaceutical Sciences, University of British Columbia, Vancouver, Canada; Shereen Elazzazy, BSc(Pharm), PharmD, is with the Department of Pharmacy - Clinical Services, National Center for Cancer Care and Research, Hamad Medical Corporation, Doha, Qatar; Azza A. Hassan, MB BCh, MSc, MD, is with the Supportive & Palliative Care Unit, National Center for Cancer Care and Research,

Hamad Medical Corporation, Doha, Qatar; Kyle J. Wilby, BSP, ACPR, PharmD, PhD, is with the School of Pharmacy, University of Otago, Dunedin, New Zealand.; (Wilby KJ) Dania Al-Masri, BSc(Pharm), MSc(Pharm), is with the College of Pharmacy, Qatar University, Doha, Qatar; Kerry Wilbur, BSc(Pharm), ACPR, PharmD, MPH, is with the Faculty of Pharmaceutical Sciences, University of British Columbia, Vancouver, Canada; Shereen Elazzazy, BSc(Pharm), PharmD, is with the Department of Pharmacy - Clinical Services, National Center for Cancer Care and Research, Hamad Medical Corporation, Doha, Qatar; Azza A. Hassan, MB BCh, MSc, MD, is with the Supportive & Palliative Care Unit, National Center for Cancer Care and Research, Hamad Medical Corporation, Doha, Qatar; Kyle J. Wilby, BSP, ACPR, PharmD, PhD, is with the School of Pharmacy, University of Otago, Dunedin, New Zealand.
(Al-Masri D) Dania Al-Masri, BSc(Pharm), MSc(Pharm), is with the College of Pharmacy, Qatar University, Doha, Qatar; Kerry Wilbur, BSc(Pharm), ACPR, PharmD, MPH, is with the Faculty of Pharmaceutical Sciences, University of British Columbia, Vancouver, Canada; Shereen Elazzazy, BSc(Pharm), PharmD, is with the Department of Pharmacy - Clinical Services, National Center for Cancer Care and Research, Hamad Medical Corporation, Doha, Qatar; Azza A. Hassan, MB BCh, MSc, MD, is with the Supportive & Palliative Care Unit, National Center for Cancer Care and Research, Hamad Medical Corporation, Doha, Qatar; Kyle J. Wilby, BSP, ACPR, PharmD, PhD, is with the School of Pharmacy, University of Otago, Dunedin, New Zealand.; (Wilbur K) Dania Al-Masri, BSc(Pharm), MSc(Pharm), is with the College of Pharmacy, Qatar University, Doha, Qatar; Kerry Wilbur, BSc(Pharm), ACPR, PharmD, MPH, is with the Faculty of Pharmaceutical Sciences, University of British Columbia, Vancouver, Canada; Shereen Elazzazy, BSc(Pharm), PharmD, is with the Department of Pharmacy - Clinical Services, National Center for Cancer Care and Research, Hamad Medical Corporation, Doha, Qatar; Azza A. Hassan, MB BCh, MSc, MD, is with the Supportive & Palliative Care Unit, National Center for Cancer Care and Research, Hamad Medical Corporation, Doha, Qatar; Kyle J. Wilby, BSP, ACPR, PharmD, PhD, is with the School of Pharmacy, University of Otago, Dunedin, New Zealand.; (Elazzazy S) Dania Al-Masri, BSc(Pharm), MSc(Pharm), is with the College of Pharmacy, Qatar University, Doha, Qatar; Kerry Wilbur, BSc(Pharm), ACPR, PharmD, MPH, is with the Faculty of Pharmaceutical Sciences, University of British Columbia, Vancouver, Canada; Shereen Elazzazy, BSc(Pharm), PharmD, is with the Department of Pharmacy - Clinical Services, National Center for Cancer Care and Research, Hamad Medical Corporation, Doha, Qatar; Azza A. Hassan, MB BCh, MSc, MD, is with the Supportive & Palliative Care Unit, National Center for Cancer Care and Research, Hamad Medical Corporation, Doha, Qatar; Kyle J. Wilby, BSP, ACPR, PharmD, PhD, is with the School of Pharmacy, University of Otago, Dunedin, New Zealand.; (Hassan AA) Dania Al-Masri, BSc(Pharm), MSc(Pharm), is with the College of Pharmacy, Qatar University, Doha, Qatar; Kerry Wilbur, BSc(Pharm), ACPR, PharmD, MPH, is with the Faculty of Pharmaceutical Sciences, University of British Columbia, Vancouver, Canada; Shereen Elazzazy, BSc(Pharm), PharmD, is with the Department of Pharmacy - Clinical Services, National Center for Cancer Care and Research, Hamad Medical Corporation, Doha, Qatar; Azza A. Hassan, MB BCh, MSc, MD, is with the Supportive & Palliative Care Unit, National Center for Cancer Care and Research, Hamad Medical Corporation, Doha, Qatar; Kyle J. Wilby, BSP, ACPR, PharmD, PhD, is with the School of Pharmacy, University of Otago, Dunedin, New Zealand.; (Wilby KJ) Dania Al-Masri, BSc(Pharm), MSc(Pharm), is with the College of Pharmacy,

Qatar University, Doha, Qatar; Kerry Wilbur, BSc(Pharm), ACPR, PharmD, MPH, is with the Faculty of Pharmaceutical Sciences, University of British Columbia, Vancouver, Canada; Shereen Elazzazy, BSc(Pharm), PharmD, is with the Department of Pharmacy - Clinical Services, National Center for Cancer Care and Research, Hamad Medical Corporation, Doha, Qatar; Azza A. Hassan, MB BCh, MSc, MD, is with the Supportive & Palliative Care Unit, National Center for Cancer Care and Research, Hamad Medical Corporation, Doha, Qatar; Kyle J. Wilby, BSP, ACPR, PharmD, PhD, is with the School of Pharmacy, University of Otago, Dunedin, New Zealand.
(Al-Masri D) Dania Al-Masri, BSc(Pharm), MSc(Pharm), is with the College of Pharmacy, Qatar University, Doha, Qatar; Kerry Wilbur, BSc(Pharm), ACPR, PharmD, MPH, is with the Faculty of Pharmaceutical Sciences, University of British Columbia, Vancouver, Canada; Shereen Elazzazy, BSc(Pharm), PharmD, is with the Department of Pharmacy - Clinical Services, National Center for Cancer Care and Research, Hamad Medical Corporation, Doha, Qatar; Azza A. Hassan, MB BCh, MSc, MD, is with the Supportive & Palliative Care Unit, National Center for Cancer Care and Research, Hamad Medical Corporation, Doha, Qatar; Kyle J. Wilby, BSP, ACPR, PharmD, PhD, is with the School of Pharmacy, University of Otago, Dunedin, New Zealand.; (Wilbur K) Dania Al-Masri, BSc(Pharm), MSc(Pharm), is with the College of Pharmacy, Qatar University, Doha, Qatar; Kerry Wilbur, BSc(Pharm), ACPR, PharmD, MPH, is with the Faculty of Pharmaceutical Sciences, University of British Columbia, Vancouver, Canada; Shereen Elazzazy, BSc(Pharm), PharmD, is with the Department of Pharmacy - Clinical Services, National Center for Cancer Care and Research, Hamad Medical Corporation, Doha, Qatar; Azza A. Hassan, MB BCh, MSc, MD, is with the Supportive & Palliative Care Unit, National Center for Cancer Care and Research, Hamad Medical Corporation, Doha, Qatar; Kyle J. Wilby, BSP, ACPR, PharmD, PhD, is with the School of Pharmacy, University of Otago, Dunedin, New Zealand.; (Elazzazy S) Dania Al-Masri, BSc(Pharm), MSc(Pharm), is with the College of Pharmacy, Qatar University, Doha, Qatar; Kerry Wilbur, BSc(Pharm), ACPR, PharmD, MPH, is with the Faculty of Pharmaceutical Sciences, University of British Columbia, Vancouver, Canada; Shereen Elazzazy, BSc(Pharm), PharmD, is with the Department of Pharmacy - Clinical Services, National Center for Cancer Care and Research, Hamad Medical Corporation, Doha, Qatar; Azza A. Hassan, MB BCh, MSc, MD, is with the Supportive & Palliative Care Unit, National Center for Cancer Care and Research, Hamad Medical Corporation, Doha, Qatar; Kyle J. Wilby, BSP, ACPR, PharmD, PhD, is with the School of Pharmacy, University of Otago, Dunedin, New Zealand.; (Hassan AA) Dania Al-Masri, BSc(Pharm), MSc(Pharm), is with the College of Pharmacy, Qatar University, Doha, Qatar; Kerry Wilbur, BSc(Pharm), ACPR, PharmD, MPH, is with the Faculty of Pharmaceutical Sciences, University of British Columbia, Vancouver, Canada; Shereen Elazzazy, BSc(Pharm), PharmD, is with the Department of Pharmacy - Clinical Services, National Center for Cancer Care and Research, Hamad Medical Corporation, Doha, Qatar; Azza A. Hassan, MB BCh, MSc, MD, is with the Supportive & Palliative Care Unit, National Center for Cancer Care and Research, Hamad Medical Corporation, Doha, Qatar; Kyle J. Wilby, BSP, ACPR, PharmD, PhD, is with the School of Pharmacy, University of Otago, Dunedin, New Zealand.; (Wilby KJ) Dania Al-Masri, BSc(Pharm), MSc(Pharm), is with the College of Pharmacy, Qatar University, Doha, Qatar; Kerry Wilbur, BSc(Pharm), ACPR, PharmD, MPH, is with the Faculty of Pharmaceutical Sciences, University of British Columbia, Vancouver, Canada; Shereen Elazzazy, BSc(Pharm), PharmD, is with the

Department of Pharmacy - Clinical Services, National Center for Cancer Care and Research, Hamad Medical Corporation, Doha, Qatar; Azza A. Hassan, MB BCh, MSc, MD, is with the Supportive & Palliative Care Unit, National Center for Cancer Care and Research, Hamad Medical Corporation, Doha, Qatar; Kyle J. Wilby, BSP, ACPR, PharmD, PhD, is with the School of Pharmacy, University of Otago, Dunedin, New Zealand.
(Al-Masri D) Dania Al-Masri, BSc(Pharm), MSc(Pharm), is with the College of Pharmacy, Qatar University, Doha, Qatar; Kerry Wilbur, BSc(Pharm), ACPR, PharmD, MPH, is with the Faculty of Pharmaceutical Sciences, University of British Columbia, Vancouver, Canada; Shereen Elazzazy, BSc(Pharm), PharmD, is with the Department of Pharmacy - Clinical Services, National Center for Cancer Care and Research, Hamad Medical Corporation, Doha, Qatar; Azza A. Hassan, MB BCh, MSc, MD, is with the Supportive & Palliative Care Unit, National Center for Cancer Care and Research, Hamad Medical Corporation, Doha, Qatar; Kyle J. Wilby, BSP, ACPR, PharmD, PhD, is with the School of Pharmacy, University of Otago, Dunedin, New Zealand.; (Wilbur K) Dania Al-Masri, BSc(Pharm), MSc(Pharm), is with the College of Pharmacy, Qatar University, Doha, Qatar; Kerry Wilbur, BSc(Pharm), ACPR, PharmD, MPH, is with the Faculty of Pharmaceutical Sciences, University of British Columbia, Vancouver, Canada; Shereen Elazzazy, BSc(Pharm), PharmD, is with the Department of Pharmacy - Clinical Services, National Center for Cancer Care and Research, Hamad Medical Corporation, Doha, Qatar; Azza A. Hassan, MB BCh, MSc, MD, is with the Supportive & Palliative Care Unit, National Center for Cancer Care and Research, Hamad Medical Corporation, Doha, Qatar; Kyle J. Wilby, BSP, ACPR, PharmD, PhD, is with the School of Pharmacy, University of Otago, Dunedin, New Zealand.; (Elazzazy S) Dania Al-Masri, BSc(Pharm), MSc(Pharm), is with the College of Pharmacy, Qatar University, Doha, Qatar; Kerry Wilbur, BSc(Pharm), ACPR, PharmD, MPH, is with the Faculty of Pharmaceutical Sciences, University of British Columbia, Vancouver, Canada; Shereen Elazzazy, BSc(Pharm), PharmD, is with the Department of Pharmacy - Clinical Services, National Center for Cancer Care and Research, Hamad Medical Corporation, Doha, Qatar; Azza A. Hassan, MB BCh, MSc, MD, is with the Supportive & Palliative Care Unit, National Center for Cancer Care and Research, Hamad Medical Corporation, Doha, Qatar; Kyle J. Wilby, BSP, ACPR, PharmD, PhD, is with the School of Pharmacy, University of Otago, Dunedin, New Zealand.; (Hassan AA) Dania Al-Masri, BSc(Pharm), MSc(Pharm), is with the College of Pharmacy, Qatar University, Doha, Qatar; Kerry Wilbur, BSc(Pharm), ACPR, PharmD, MPH, is with the Faculty of Pharmaceutical Sciences, University of British Columbia, Vancouver, Canada; Shereen Elazzazy, BSc(Pharm), PharmD, is with the Department of Pharmacy - Clinical Services, National Center for Cancer Care and Research, Hamad Medical Corporation, Doha, Qatar; Azza A. Hassan, MB BCh, MSc, MD, is with the Supportive & Palliative Care Unit, National Center for Cancer Care and Research, Hamad Medical Corporation, Doha, Qatar; Kyle J. Wilby, BSP, ACPR, PharmD, PhD, is with the School of Pharmacy, University of Otago, Dunedin, New Zealand.; (Wilby KJ) Dania Al-Masri, BSc(Pharm), MSc(Pharm), is with the College of Pharmacy, Qatar University, Doha, Qatar; Kerry Wilbur, BSc(Pharm), ACPR, PharmD, MPH, is with the Faculty of Pharmaceutical Sciences, University of British Columbia, Vancouver, Canada; Shereen Elazzazy, BSc(Pharm), PharmD, is with the Department of Pharmacy - Clinical Services, National Center for Cancer Care and Research, Hamad Medical Corporation, Doha, Qatar; Azza A. Hassan, MB BCh, MSc, MD, is with the Supportive & Palliative Care Unit, National Center for Cancer

Care and Research, Hamad Medical Corporation, Doha, Qatar; Kyle J. Wilby, BSP, ACPR, PharmD, PhD, is with the School of Pharmacy, University of Otago, Dunedin, New Zealand.
Database: PubMed

30. Risk of opioid misuse in people with cancer and pain and related clinical considerations: a qualitative study of the perspectives of Australian general practitioners.

Author(s): Luckett T; Newton-John T; Phillips J; Holliday S; Giannitrapani K; Powell-Davies G; Lovell M; Liauw W; Rowett D; Pearson SA; Raymond B; Heneka N; Lorenz K

Source: BMJ open; ; vol. 10 (no. 2); p. e034363

Publication Type(s): Journal Article; Research Support, Non-U.S. Gov't

DOI: http://dx.doi.org/10.1136/bmjopen-2019-034363

ISSN: 2044-6055

Place of Publication: England

PubMedID: 32071185

Accession Number: 32071185

Available at BMJ open - from Europe PubMed Central - Open Access

Available at BMJ open - from HighWire - Free Full Text

Available at BMJ open - from ProQuest (Health Research Premium) - NHS Version

Available at BMJ open - from Unpaywall

Keywords: Subject Terms: *cancer pain; *pain management; *primary care; *qualitative research

Abstract:OBJECTIVE: To explore the perspectives of general practitioners (GPs) concerning the risk of opioid misuse in people with cancer and pain and related clinical considerations.DESIGN: A qualitative approach using semistructured telephone interviews. Analysis used an integrative approach.SETTING: Primary care.PARTICIPANTS: Australian GPs with experience of prescribing opioids for people with cancer and pain.RESULTS: Twenty-two GPs participated, and three themes emerged. Theme 1 (Misuse is not the main problem) contextualised misuse as a relatively minor concern compared with pain control and toxicity, and highlighted underlying systemic factors, including limitations in continuity of care and doctor expertise. Theme 2 ('A different mindset' for cancer pain) captured participants' relative comfort in prescribing opioids for pain in cancer versus non-cancer contexts, and acknowledgement that compassion and greater perceived community acceptance were driving factors, in addition to scientific support for mechanisms and clinical efficacy. Participant attitudes towards prescribing for people with cancer versus non-cancer pain differed most when cancer was in the palliative phase, when they were unconcerned by misuse. Participants were equivocal about the risk-benefit ratio of long-term opioid therapy in the chronic phase of cancer, and were reluctant to prescribe for disease-free survivors. Theme 3 ('The question is always, 'how lazy have you been?') captured participants' acknowledgement that they sometimes prescribed opioids for cancer pain as a default, easier option compared with more holistic pain management.CONCLUSIONS: Findings highlight the role of specific

clinical considerations in distinguishing risk of opioid misuse in the cancer versus non-cancer population, rather than diagnosis per se. Further efforts are needed to ensure continuity of care where opioid prescribing is shared. Greater evidence is needed to guide opioid prescribing in disease-free survivors and the chronic phase of cancer, especially in the context of new treatments for metastatic disease.

Institutions:

(Luckett T) IMPACCT (Improving Palliative, Aged and Chronic Care through Clinical Research and Translation), Faculty of Health, University of Technology Sydney, Ultimo, New South Wales, Australia tim.luckett@uts.edu.au.; (Newton-John T) Graduate School of Health, University of Technology Sydney, Sydney, New South Wales, Australia.; (Phillips J) IMPACCT (Improving Palliative, Aged and Chronic Care through Clinical Research and Translation), Faculty of Health, University of Technology Sydney, Ultimo, New South Wales, Australia.; (Holliday S) School of Medicine and Public Health, University of Newcastle, Newcastle, New South Wales, Australia.; (Giannitrapani K) Medicine - Primary Care and Population Health, Stanford University, Stanford, California, USA.; (Powell-Davies G) Centre for Primary Health Care and Equity, University of New South Wales, Kensington, New South Wales, Australia.; (Lovell M) Palliative Care, Greenwich Hospital, Greenwich, New South Wales, Australia.; (Liauw W) Saint George and Sutherland Clinical School, University of New South Wales, Kogarah, New South Wales, Australia.; (Rowett D) School of Pharmacy and Medical Sciences, University of South Australia, Adelaide, South Australia, Australia.; (Pearson SA) Medicines Policy Research Unit, University of New South Wales, Sydney, New South Wales, Australia.; (Raymond B) IMPACCT (Improving Palliative, Aged and Chronic Care through Clinical Research and Translation), Faculty of Health, University of Technology Sydney, Ultimo, New South Wales, Australia.; (Heneka N) IMPACCT (Improving Palliative, Aged and Chronic Care through Clinical Research and Translation), Faculty of Health, University of Technology Sydney, Ultimo, New South Wales, Australia.; (Lorenz K) Medicine - Primary Care and Population Health, Stanford University, Stanford, California, USA.
(Luckett T) IMPACCT (Improving Palliative, Aged and Chronic Care through Clinical Research and Translation), Faculty of Health, University of Technology Sydney, Ultimo, New South Wales, Australia tim.luckett@uts.edu.au.; (Newton-John T) Graduate School of Health, University of Technology Sydney, Sydney, New South Wales, Australia.; (Phillips J) IMPACCT (Improving Palliative, Aged and Chronic Care through Clinical Research and Translation), Faculty of Health, University of Technology Sydney, Ultimo, New South Wales, Australia.; (Holliday S) School of Medicine and Public Health, University of Newcastle, Newcastle, New South Wales, Australia.; (Giannitrapani K) Medicine - Primary Care and Population Health, Stanford University, Stanford, California, USA.; (Powell-Davies G) Centre for Primary Health Care and Equity, University of New South Wales, Kensington, New South Wales, Australia.; (Lovell M) Palliative Care, Greenwich Hospital, Greenwich, New South Wales, Australia.; (Liauw W) Saint George and Sutherland Clinical School, University of New South Wales, Kogarah, New South Wales, Australia.; (Rowett D) School of Pharmacy and Medical Sciences, University of South Australia, Adelaide, South Australia, Australia.; (Pearson SA) Medicines Policy Research Unit, University of New South Wales, Sydney, New South Wales, Australia.; (Raymond B) IMPACCT (Improving Palliative, Aged and Chronic Care through Clinical Research and Translation), Faculty of Health, University of Technology Sydney, Ultimo, New South Wales, Australia.; (Heneka N) IMPACCT (Improving Palliative, Aged and Chronic Care through Clinical Research and Translation), Faculty of Health, University of

Technology Sydney, Ultimo, New South Wales, Australia.; (Lorenz K) Medicine - Primary Care and Population Health, Stanford University, Stanford, California, USA. (Luckett T) IMPACCT (Improving Palliative, Aged and Chronic Care through Clinical Research and Translation), Faculty of Health, University of Technology Sydney, Ultimo, New South Wales, Australia tim.luckett@uts.edu.au.; (Newton-John T) Graduate School of Health, University of Technology Sydney, Sydney, New South Wales, Australia.; (Phillips J) IMPACCT (Improving Palliative, Aged and Chronic Care through Clinical Research and Translation), Faculty of Health, University of Technology Sydney, Ultimo, New South Wales, Australia.; (Holliday S) School of Medicine and Public Health, University of Newcastle, Newcastle, New South Wales, Australia.; (Giannitrapani K) Medicine - Primary Care and Population Health, Stanford University, Stanford, California, USA.; (Powell-Davies G) Centre for Primary Health Care and Equity, University of New South Wales, Kensington, New South Wales, Australia.; (Lovell M) Palliative Care, Greenwich Hospital, Greenwich, New South Wales, Australia.; (Liauw W) Saint George and Sutherland Clinical School, University of New South Wales, Kogarah, New South Wales, Australia.; (Rowett D) School of Pharmacy and Medical Sciences, University of South Australia, Adelaide, South Australia, Australia.; (Pearson SA) Medicines Policy Research Unit, University of New South Wales, Sydney, New South Wales, Australia.; (Raymond B) IMPACCT (Improving Palliative, Aged and Chronic Care through Clinical Research and Translation), Faculty of Health, University of Technology Sydney, Ultimo, New South Wales, Australia.; (Heneka N) IMPACCT (Improving Palliative, Aged and Chronic Care through Clinical Research and Translation), Faculty of Health, University of Technology Sydney, Ultimo, New South Wales, Australia.; (Lorenz K) Medicine - Primary Care and Population Health, Stanford University, Stanford, California, USA. (Luckett T) IMPACCT (Improving Palliative, Aged and Chronic Care through Clinical Research and Translation), Faculty of Health, University of Technology Sydney, Ultimo, New South Wales, Australia tim.luckett@uts.edu.au.; (Newton-John T) Graduate School of Health, University of Technology Sydney, Sydney, New South Wales, Australia.; (Phillips J) IMPACCT (Improving Palliative, Aged and Chronic Care through Clinical Research and Translation), Faculty of Health, University of Technology Sydney, Ultimo, New South Wales, Australia.; (Holliday S) School of Medicine and Public Health, University of Newcastle, Newcastle, New South Wales, Australia.; (Giannitrapani K) Medicine - Primary Care and Population Health, Stanford University, Stanford, California, USA.; (Powell-Davies G) Centre for Primary Health Care and Equity, University of New South Wales, Kensington, New South Wales, Australia.; (Lovell M) Palliative Care, Greenwich Hospital, Greenwich, New South Wales, Australia.; (Liauw W) Saint George and Sutherland Clinical School, University of New South Wales, Kogarah, New South Wales, Australia.; (Rowett D) School of Pharmacy and Medical Sciences, University of South Australia, Adelaide, South Australia, Australia.; (Pearson SA) Medicines Policy Research Unit, University of New South Wales, Sydney, New South Wales, Australia.; (Raymond B) IMPACCT (Improving Palliative, Aged and Chronic Care through Clinical Research and Translation), Faculty of Health, University of Technology Sydney, Ultimo, New South Wales, Australia.; (Heneka N) IMPACCT (Improving Palliative, Aged and Chronic Care through Clinical Research and Translation), Faculty of Health, University of Technology Sydney, Ultimo, New South Wales, Australia.; (Lorenz K) Medicine - Primary Care and Population Health, Stanford University, Stanford, California, USA. (Luckett T) IMPACCT (Improving Palliative, Aged and Chronic Care through Clinical Research and Translation), Faculty of Health, University of Technology Sydney,

Ultimo, New South Wales, Australia tim.luckett@uts.edu.au.; (Newton-John T) Graduate School of Health, University of Technology Sydney, Sydney, New South Wales, Australia.; (Phillips J) IMPACCT (Improving Palliative, Aged and Chronic Care through Clinical Research and Translation), Faculty of Health, University of Technology Sydney, Ultimo, New South Wales, Australia.; (Holliday S) School of Medicine and Public Health, University of Newcastle, Newcastle, New South Wales, Australia.; (Giannitrapani K) Medicine - Primary Care and Population Health, Stanford University, Stanford, California, USA.; (Powell-Davies G) Centre for Primary Health Care and Equity, University of New South Wales, Kensington, New South Wales, Australia.; (Lovell M) Palliative Care, Greenwich Hospital, Greenwich, New South Wales, Australia.; (Liauw W) Saint George and Sutherland Clinical School, University of New South Wales, Kogarah, New South Wales, Australia.; (Rowett D) School of Pharmacy and Medical Sciences, University of South Australia, Adelaide, South Australia, Australia.; (Pearson SA) Medicines Policy Research Unit, University of New South Wales, Sydney, New South Wales, Australia.; (Raymond B) IMPACCT (Improving Palliative, Aged and Chronic Care through Clinical Research and Translation), Faculty of Health, University of Technology Sydney, Ultimo, New South Wales, Australia.; (Heneka N) IMPACCT (Improving Palliative, Aged and Chronic Care through Clinical Research and Translation), Faculty of Health, University of Technology Sydney, Ultimo, New South Wales, Australia.; (Lorenz K) Medicine - Primary Care and Population Health, Stanford University, Stanford, California, USA.
(Luckett T) IMPACCT (Improving Palliative, Aged and Chronic Care through Clinical Research and Translation), Faculty of Health, University of Technology Sydney, Ultimo, New South Wales, Australia tim.luckett@uts.edu.au.; (Newton-John T) Graduate School of Health, University of Technology Sydney, Sydney, New South Wales, Australia.; (Phillips J) IMPACCT (Improving Palliative, Aged and Chronic Care through Clinical Research and Translation), Faculty of Health, University of Technology Sydney, Ultimo, New South Wales, Australia.; (Holliday S) School of Medicine and Public Health, University of Newcastle, Newcastle, New South Wales, Australia.; (Giannitrapani K) Medicine - Primary Care and Population Health, Stanford University, Stanford, California, USA.; (Powell-Davies G) Centre for Primary Health Care and Equity, University of New South Wales, Kensington, New South Wales, Australia.; (Lovell M) Palliative Care, Greenwich Hospital, Greenwich, New South Wales, Australia.; (Liauw W) Saint George and Sutherland Clinical School, University of New South Wales, Kogarah, New South Wales, Australia.; (Rowett D) School of Pharmacy and Medical Sciences, University of South Australia, Adelaide, South Australia, Australia.; (Pearson SA) Medicines Policy Research Unit, University of New South Wales, Sydney, New South Wales, Australia.; (Raymond B) IMPACCT (Improving Palliative, Aged and Chronic Care through Clinical Research and Translation), Faculty of Health, University of Technology Sydney, Ultimo, New South Wales, Australia.; (Heneka N) IMPACCT (Improving Palliative, Aged and Chronic Care through Clinical Research and Translation), Faculty of Health, University of Technology Sydney, Ultimo, New South Wales, Australia.; (Lorenz K) Medicine - Primary Care and Population Health, Stanford University, Stanford, California, USA.
(Luckett T) IMPACCT (Improving Palliative, Aged and Chronic Care through Clinical Research and Translation), Faculty of Health, University of Technology Sydney, Ultimo, New South Wales, Australia tim.luckett@uts.edu.au.; (Newton-John T) Graduate School of Health, University of Technology Sydney, Sydney, New South Wales, Australia.; (Phillips J) IMPACCT (Improving Palliative, Aged and Chronic Care through Clinical Research and Translation), Faculty of Health, University of

Technology Sydney, Ultimo, New South Wales, Australia.; (Holliday S) School of Medicine and Public Health, University of Newcastle, Newcastle, New South Wales, Australia.; (Giannitrapani K) Medicine - Primary Care and Population Health, Stanford University, Stanford, California, USA.; (Powell-Davies G) Centre for Primary Health Care and Equity, University of New South Wales, Kensington, New South Wales, Australia.; (Lovell M) Palliative Care, Greenwich Hospital, Greenwich, New South Wales, Australia.; (Liauw W) Saint George and Sutherland Clinical School, University of New South Wales, Kogarah, New South Wales, Australia.; (Rowett D) School of Pharmacy and Medical Sciences, University of South Australia, Adelaide, South Australia, Australia.; (Pearson SA) Medicines Policy Research Unit, University of New South Wales, Sydney, New South Wales, Australia.; (Raymond B) IMPACCT (Improving Palliative, Aged and Chronic Care through Clinical Research and Translation), Faculty of Health, University of Technology Sydney, Ultimo, New South Wales, Australia.; (Heneka N) IMPACCT (Improving Palliative, Aged and Chronic Care through Clinical Research and Translation), Faculty of Health, University of Technology Sydney, Ultimo, New South Wales, Australia.; (Lorenz K) Medicine - Primary Care and Population Health, Stanford University, Stanford, California, USA. (Luckett T) IMPACCT (Improving Palliative, Aged and Chronic Care through Clinical Research and Translation), Faculty of Health, University of Technology Sydney, Ultimo, New South Wales, Australia tim.luckett@uts.edu.au.; (Newton-John T) Graduate School of Health, University of Technology Sydney, Sydney, New South Wales, Australia.; (Phillips J) IMPACCT (Improving Palliative, Aged and Chronic Care through Clinical Research and Translation), Faculty of Health, University of Technology Sydney, Ultimo, New South Wales, Australia.; (Holliday S) School of Medicine and Public Health, University of Newcastle, Newcastle, New South Wales, Australia.; (Giannitrapani K) Medicine - Primary Care and Population Health, Stanford University, Stanford, California, USA.; (Powell-Davies G) Centre for Primary Health Care and Equity, University of New South Wales, Kensington, New South Wales, Australia.; (Lovell M) Palliative Care, Greenwich Hospital, Greenwich, New South Wales, Australia.; (Liauw W) Saint George and Sutherland Clinical School, University of New South Wales, Kogarah, New South Wales, Australia.; (Rowett D) School of Pharmacy and Medical Sciences, University of South Australia, Adelaide, South Australia, Australia.; (Pearson SA) Medicines Policy Research Unit, University of New South Wales, Sydney, New South Wales, Australia.; (Raymond B) IMPACCT (Improving Palliative, Aged and Chronic Care through Clinical Research and Translation), Faculty of Health, University of Technology Sydney, Ultimo, New South Wales, Australia.; (Heneka N) IMPACCT (Improving Palliative, Aged and Chronic Care through Clinical Research and Translation), Faculty of Health, University of Technology Sydney, Ultimo, New South Wales, Australia.; (Lorenz K) Medicine - Primary Care and Population Health, Stanford University, Stanford, California, USA. (Luckett T) IMPACCT (Improving Palliative, Aged and Chronic Care through Clinical Research and Translation), Faculty of Health, University of Technology Sydney, Ultimo, New South Wales, Australia tim.luckett@uts.edu.au.; (Newton-John T) Graduate School of Health, University of Technology Sydney, Sydney, New South Wales, Australia.; (Phillips J) IMPACCT (Improving Palliative, Aged and Chronic Care through Clinical Research and Translation), Faculty of Health, University of Technology Sydney, Ultimo, New South Wales, Australia.; (Holliday S) School of Medicine and Public Health, University of Newcastle, Newcastle, New South Wales, Australia.; (Giannitrapani K) Medicine - Primary Care and Population Health, Stanford University, Stanford, California, USA.; (Powell-Davies G) Centre for Primary

Health Care and Equity, University of New South Wales, Kensington, New South Wales, Australia.; (Lovell M) Palliative Care, Greenwich Hospital, Greenwich, New South Wales, Australia.; (Liauw W) Saint George and Sutherland Clinical School, University of New South Wales, Kogarah, New South Wales, Australia.; (Rowett D) School of Pharmacy and Medical Sciences, University of South Australia, Adelaide, South Australia, Australia.; (Pearson SA) Medicines Policy Research Unit, University of New South Wales, Sydney, New South Wales, Australia.; (Raymond B) IMPACCT (Improving Palliative, Aged and Chronic Care through Clinical Research and Translation), Faculty of Health, University of Technology Sydney, Ultimo, New South Wales, Australia.; (Heneka N) IMPACCT (Improving Palliative, Aged and Chronic Care through Clinical Research and Translation), Faculty of Health, University of Technology Sydney, Ultimo, New South Wales, Australia.; (Lorenz K) Medicine - Primary Care and Population Health, Stanford University, Stanford, California, USA. (Luckett T) IMPACCT (Improving Palliative, Aged and Chronic Care through Clinical Research and Translation), Faculty of Health, University of Technology Sydney, Ultimo, New South Wales, Australia tim.luckett@uts.edu.au.; (Newton-John T) Graduate School of Health, University of Technology Sydney, Sydney, New South Wales, Australia.; (Phillips J) IMPACCT (Improving Palliative, Aged and Chronic Care through Clinical Research and Translation), Faculty of Health, University of Technology Sydney, Ultimo, New South Wales, Australia.; (Holliday S) School of Medicine and Public Health, University of Newcastle, Newcastle, New South Wales, Australia.; (Giannitrapani K) Medicine - Primary Care and Population Health, Stanford University, Stanford, California, USA.; (Powell-Davies G) Centre for Primary
Health Care and Equity, University of New South Wales, Kensington, New South Wales, Australia.; (Lovell M) Palliative Care, Greenwich Hospital, Greenwich, New South Wales, Australia.; (Liauw W) Saint George and Sutherland Clinical School, University of New South Wales, Kogarah, New South Wales, Australia.; (Rowett D) School of Pharmacy and Medical Sciences, University of South Australia, Adelaide, South Australia, Australia.; (Pearson SA) Medicines Policy Research Unit, University of New South Wales, Sydney, New South Wales, Australia.; (Raymond B) IMPACCT (Improving Palliative, Aged and Chronic Care through Clinical Research and Translation), Faculty of Health, University of Technology Sydney, Ultimo, New South Wales, Australia.; (Heneka N) IMPACCT (Improving Palliative, Aged and Chronic Care through Clinical Research and Translation), Faculty of Health, University of Technology Sydney, Ultimo, New South Wales, Australia.; (Lorenz K) Medicine - Primary Care and Population Health, Stanford University, Stanford, California, USA. (Luckett T) IMPACCT (Improving Palliative, Aged and Chronic Care through Clinical Research and Translation), Faculty of Health, University of Technology Sydney, Ultimo, New South Wales, Australia tim.luckett@uts.edu.au.; (Newton-John T) Graduate School of Health, University of Technology Sydney, Sydney, New South Wales, Australia.; (Phillips J) IMPACCT (Improving Palliative, Aged and Chronic Care through Clinical Research and Translation), Faculty of Health, University of Technology Sydney, Ultimo, New South Wales, Australia.; (Holliday S) School of Medicine and Public Health, University of Newcastle, Newcastle, New South Wales, Australia.; (Giannitrapani K) Medicine - Primary Care and Population Health, Stanford University, Stanford, California, USA.; (Powell-Davies G) Centre for Primary Health Care and Equity, University of New South Wales, Kensington, New South Wales, Australia.; (Lovell M) Palliative Care, Greenwich Hospital, Greenwich, New South Wales, Australia.; (Liauw W) Saint George and Sutherland Clinical School, University of New South Wales, Kogarah, New South Wales, Australia.; (Rowett D)

School of Pharmacy and Medical Sciences, University of South Australia, Adelaide, South Australia, Australia.; (Pearson SA) Medicines Policy Research Unit, University of New South Wales, Sydney, New South Wales, Australia.; (Raymond B) IMPACCT (Improving Palliative, Aged and Chronic Care through Clinical Research and Translation), Faculty of Health, University of Technology Sydney, Ultimo, New South Wales, Australia.; (Heneka N) IMPACCT (Improving Palliative, Aged and Chronic Care through Clinical Research and Translation), Faculty of Health, University of Technology Sydney, Ultimo, New South Wales, Australia.; (Lorenz K) Medicine - Primary Care and Population Health, Stanford University, Stanford, California, USA. (Luckett T) IMPACCT (Improving Palliative, Aged and Chronic Care through Clinical Research and Translation), Faculty of Health, University of Technology Sydney, Ultimo, New South Wales, Australia tim.luckett@uts.edu.au.; (Newton-John T) Graduate School of Health, University of Technology Sydney, Sydney, New South Wales, Australia.; (Phillips J) IMPACCT (Improving Palliative, Aged and Chronic Care through Clinical Research and Translation), Faculty of Health, University of Technology Sydney, Ultimo, New South Wales, Australia.; (Holliday S) School of Medicine and Public Health, University of Newcastle, Newcastle, New South Wales, Australia.; (Giannitrapani K) Medicine - Primary Care and Population Health, Stanford University, Stanford, California, USA.; (Powell-Davies G) Centre for Primary Health Care and Equity, University of New South Wales, Kensington, New South Wales, Australia.; (Lovell M) Palliative Care, Greenwich Hospital, Greenwich, New South Wales, Australia.; (Liauw W) Saint George and Sutherland Clinical School, University of New South Wales, Kogarah, New South Wales, Australia.; (Rowett D) School of Pharmacy and Medical Sciences, University of South Australia, Adelaide, South Australia, Australia.; (Pearson SA) Medicines Policy Research Unit, University of New South Wales, Sydney, New South Wales, Australia.; (Raymond B) IMPACCT (Improving Palliative, Aged and Chronic Care through Clinical Research and Translation), Faculty of Health, University of Technology Sydney, Ultimo, New South Wales, Australia.; (Heneka N) IMPACCT (Improving Palliative, Aged and Chronic Care through Clinical Research and Translation), Faculty of Health, University of Technology Sydney, Ultimo, New South Wales, Australia.; (Lorenz K) Medicine - Primary Care and Population Health, Stanford University, Stanford, California, USA. (Luckett T) IMPACCT (Improving Palliative, Aged and Chronic Care through Clinical Research and Translation), Faculty of Health, University of Technology Sydney, Ultimo, New South Wales, Australia tim.luckett@uts.edu.au.; (Newton-John T) Graduate School of Health, University of Technology Sydney, Sydney, New South Wales, Australia.; (Phillips J) IMPACCT (Improving Palliative, Aged and Chronic Care through Clinical Research and Translation), Faculty of Health, University of Technology Sydney, Ultimo, New South Wales, Australia.; (Holliday S) School of Medicine and Public Health, University of Newcastle, Newcastle, New South Wales, Australia.; (Giannitrapani K) Medicine - Primary Care and Population Health, Stanford University, Stanford, California, USA.; (Powell-Davies G) Centre for Primary Health Care and Equity, University of New South Wales, Kensington, New South Wales, Australia.; (Lovell M) Palliative Care, Greenwich Hospital, Greenwich, New South Wales, Australia.; (Liauw W) Saint George and Sutherland Clinical School, University of New South Wales, Kogarah, New South Wales, Australia.; (Rowett D) School of Pharmacy and Medical Sciences, University of South Australia, Adelaide, South Australia, Australia.; (Pearson SA) Medicines Policy Research Unit, University of New South Wales, Sydney, New South Wales, Australia.; (Raymond B) IMPACCT (Improving Palliative, Aged and Chronic Care through Clinical Research and

Translation), Faculty of Health, University of Technology Sydney, Ultimo, New South Wales, Australia.; (Heneka N) IMPACCT (Improving Palliative, Aged and Chronic Care through Clinical Research and Translation), Faculty of Health, University of Technology Sydney, Ultimo, New South Wales, Australia.; (Lorenz K) Medicine - Primary Care and Population Health, Stanford University, Stanford, California, USA.
Database: PubMed

31. Mindfulness-Based stress reduction in early palliative care for people with metastatic cancer: A mixed-method study.

Author(s): Poletti S; Razzini G; Ferrari R; Ricchieri MP; Spedicato GA; Pasqualini A; Buzzega C; Artioli F; Petropulacos K; Luppi M; Bandieri E

Source: Complementary therapies in medicine; Dec 2019; vol. 47 ; p. 102218

Publication Date: Dec 2019

Publication Type(s): Journal Article

DOI: http://dx.doi.org/10.1016/j.ctim.2019.102218

ISSN: 1873-6963

Place of Publication: Scotland

PubMedID: 31780005

Accession Number: 31780005

Keywords: Subject Terms: Early palliative care; Integrative healthcare; Interpretive-phenomenological analysis; Metastatic cancer; Mindfulness-based stress reduction

Abstract:OBJECTIVES: To explore the impact of a Mindfulness-Based Stress Reduction (MBSR) intervention for people with metastatic cancer integrated in Early Palliative Care (EPC).DESIGN: Mixed-method study.SETTINGS/LOCATION: EPC Service integrated with Oncology Unit, Carpi General Hospital, Italy from January to October 2017. The MBSR intervention took place inside the hospital.SUBJECTS: Study participation was offered to 25 consecutive people referred to the EPC service.INCLUSION CRITERIA: people with metastatic cancer between 18 and 75 years old; informed consent.EXCLUSION CRITERIA: Performance Status <60% according to Karnofsky scale; active psychiatric disorder. 20 patients were included in the study.INTERVENTION: The adapted program consists of 8 meetings for 2.5 h once a week, a 4.5 h session between the 6th and 7th weeks and 0.5 h home practice daily. The following mindfulness practices were included during the training: formal sitting meditation, body scan, light yoga, walking meditation, and Aikido exercises. Participants were provided with materials for home practice. A qualified MBSR instructor conducted the program. Sessions were attended by a clinical psychologist and a physician trained in meditation, together with the palliative nurse as facilitators.OUTCOME MEASURES: Feasibility and acceptability were assessed on 16 participants. In addition, pre-post measures of cancer pain and mood state were collected. Semi-structured, in-depth interviews were conducted on a subset of 8 participants at the end of the study and analysed using the Interpretative-Phenomenological approach.RESULTS: MBSR attendance to meetings and adherence to home practice were 75%. MBSR intervention helped participants to develop an accepting attitude in respect to metastatic cancer disease helping them to face anxiety and cancer pain. MBSR improves self-regulation of mood state engendering feelings of compassion MBSR program supports participants in

questioning and reconnecting with their values and spiritual beliefs.CONCLUSIONS: A Mindfulness intervention integrated into EPC setting is feasible, well accepted and could help metastatic cancer patients to control cancer pain together with an opportunity of emotional and spiritual relief.

Institutions:

(Poletti S) INSERM U1028, CRNL-DYCOG, Lyon, France. Electronic address: stefano.poletti@phd.unipd.it.; (Razzini G) Unit of Medical Oncology, Carpi Civil Hospital, Italy. Electronic address: g.razzini-ext@ausl.mo.it.; (Ferrari R) "Mente&Vita" Mindfulness Center, Italy. Electronic address: roberto.ferrari1@gmail.com.; (Ricchieri MP) Local Health Unit of Modena, MBSR Instructor, Italy. Electronic address: m.ricchieri@ausl.mo.it.; (Spedicato GA) FCAS, FSA, C. Stat. Unipol Group, Bologna, Italy. Electronic address: spedygiorgio@gmail.com.; (Pasqualini A) Unit of Medical Oncology, Carpi Civil Hospital, Italy. Electronic address: a.pasqualini@ausl.mo.it.; (Buzzega C) Unit of Early Palliative Care, Carpi Civil Hospital, Italy. Electronic address: c.buzzega@ausl.mo.it.; (Artioli F) Unit of Medical Oncology, Carpi Civil Hospital, Italy. Electronic address: f.artioli@ausl.mo.it.; (Petropulacos K) General Director Health Councillorship Emilia Romagna Region, Bologna, Italy. Electronic address: Kyriakoula.Petropulacos@regione.emilia-romagna.it.; (Luppi M) Department of Medical and Surgical Sciences, AOU, UNIMORE, Italy. Electronic address: mario.luppi@unimore.it.; (Bandieri E) Unit of Early Palliative Care, Carpi Civil Hospital, Italy. Electronic address: e.bandieri@ausl.mo.it.
(Poletti S) INSERM U1028, CRNL-DYCOG, Lyon, France. Electronic address: stefano.poletti@phd.unipd.it.; (Razzini G) Unit of Medical Oncology, Carpi Civil Hospital, Italy. Electronic address: g.razzini-ext@ausl.mo.it.; (Ferrari R) "Mente&Vita" Mindfulness Center, Italy. Electronic address: roberto.ferrari1@gmail.com.; (Ricchieri MP) Local Health Unit of Modena, MBSR Instructor, Italy. Electronic address: m.ricchieri@ausl.mo.it.; (Spedicato GA) FCAS, FSA, C. Stat. Unipol Group, Bologna, Italy. Electronic address: spedygiorgio@gmail.com.; (Pasqualini A) Unit of Medical Oncology, Carpi Civil Hospital, Italy. Electronic address: a.pasqualini@ausl.mo.it.; (Buzzega C) Unit of Early Palliative Care, Carpi Civil Hospital, Italy. Electronic address: c.buzzega@ausl.mo.it.; (Artioli F) Unit of Medical Oncology, Carpi Civil Hospital, Italy. Electronic address: f.artioli@ausl.mo.it.; (Petropulacos K) General Director Health Councillorship Emilia Romagna Region, Bologna, Italy. Electronic address: Kyriakoula.Petropulacos@regione.emilia-romagna.it.; (Luppi M) Department of Medical and Surgical Sciences, AOU, UNIMORE, Italy. Electronic address: mario.luppi@unimore.it.; (Bandieri E) Unit of Early Palliative Care, Carpi Civil Hospital, Italy. Electronic address: e.bandieri@ausl.mo.it.
(Poletti S) INSERM U1028, CRNL-DYCOG, Lyon, France. Electronic address: stefano.poletti@phd.unipd.it.; (Razzini G) Unit of Medical Oncology, Carpi Civil Hospital, Italy. Electronic address: g.razzini-ext@ausl.mo.it.; (Ferrari R) "Mente&Vita" Mindfulness Center, Italy. Electronic address: roberto.ferrari1@gmail.com.; (Ricchieri MP) Local Health Unit of Modena, MBSR Instructor, Italy. Electronic address: m.ricchieri@ausl.mo.it.; (Spedicato GA) FCAS, FSA, C. Stat. Unipol Group, Bologna, Italy. Electronic address: spedygiorgio@gmail.com.; (Pasqualini A) Unit of Medical Oncology, Carpi Civil Hospital, Italy. Electronic address: a.pasqualini@ausl.mo.it.; (Buzzega C) Unit of Early Palliative Care, Carpi Civil Hospital, Italy. Electronic address: c.buzzega@ausl.mo.it.; (Artioli F) Unit of Medical Oncology, Carpi Civil Hospital, Italy. Electronic address: f.artioli@ausl.mo.it.; (Petropulacos K) General Director Health Councillorship Emilia Romagna Region, Bologna, Italy. Electronic address: Kyriakoula.Petropulacos@regione.emilia-romagna.it.; (Luppi M)

Department of Medical and Surgical Sciences, AOU, UNIMORE, Italy. Electronic address: mario.luppi@unimore.it.; (Bandieri E) Unit of Early Palliative Care, Carpi Civil Hospital, Italy. Electronic address: e.bandieri@ausl.mo.it.
(Poletti S) INSERM U1028, CRNL-DYCOG, Lyon, France. Electronic address: stefano.poletti@phd.unipd.it.; (Razzini G) Unit of Medical Oncology, Carpi Civil Hospital, Italy. Electronic address: g.razzini-ext@ausl.mo.it.; (Ferrari R) "Mente&Vita" Mindfulness Center, Italy. Electronic address: roberto.ferrari1@gmail.com.; (Ricchieri MP) Local Health Unit of Modena, MBSR Instructor, Italy. Electronic address: m.ricchieri@ausl.mo.it.; (Spedicato GA) FCAS, FSA, C. Stat. Unipol Group, Bologna, Italy. Electronic address: spedygiorgio@gmail.com.; (Pasqualini A) Unit of Medical Oncology, Carpi Civil Hospital, Italy. Electronic address: a.pasqualini@ausl.mo.it.; (Buzzega C) Unit of Early Palliative Care, Carpi Civil Hospital, Italy. Electronic address: c.buzzega@ausl.mo.it.; (Artioli F) Unit of Medical Oncology, Carpi Civil Hospital, Italy. Electronic address: f.artioli@ausl.mo.it.; (Petropulacos K) General Director Health Councillorship Emilia Romagna Region, Bologna, Italy. Electronic address: Kyriakoula.Petropulacos@regione.emilia-romagna.it.; (Luppi M) Department of Medical and Surgical Sciences, AOU, UNIMORE, Italy. Electronic address: mario.luppi@unimore.it.; (Bandieri E) Unit of Early Palliative Care, Carpi Civil Hospital, Italy. Electronic address: e.bandieri@ausl.mo.it.
(Poletti S) INSERM U1028, CRNL-DYCOG, Lyon, France. Electronic address: stefano.poletti@phd.unipd.it.; (Razzini G) Unit of Medical Oncology, Carpi Civil Hospital, Italy. Electronic address: g.razzini-ext@ausl.mo.it.; (Ferrari R) "Mente&Vita" Mindfulness Center, Italy. Electronic address: roberto.ferrari1@gmail.com.; (Ricchieri MP) Local Health Unit of Modena, MBSR Instructor, Italy. Electronic address: m.ricchieri@ausl.mo.it.; (Spedicato GA) FCAS, FSA, C. Stat. Unipol Group, Bologna, Italy. Electronic address: spedygiorgio@gmail.com.; (Pasqualini A) Unit of Medical Oncology, Carpi Civil Hospital, Italy. Electronic address: a.pasqualini@ausl.mo.it.; (Buzzega C) Unit of Early Palliative Care, Carpi Civil Hospital, Italy. Electronic address: c.buzzega@ausl.mo.it.; (Artioli F) Unit of Medical Oncology, Carpi Civil Hospital, Italy. Electronic address: f.artioli@ausl.mo.it.; (Petropulacos K) General Director Health Councillorship Emilia Romagna Region, Bologna, Italy. Electronic address: Kyriakoula.Petropulacos@regione.emilia-romagna.it.; (Luppi M) Department of Medical and Surgical Sciences, AOU, UNIMORE, Italy. Electronic address: mario.luppi@unimore.it.; (Bandieri E) Unit of Early Palliative Care, Carpi Civil Hospital, Italy. Electronic address: e.bandieri@ausl.mo.it.
(Poletti S) INSERM U1028, CRNL-DYCOG, Lyon, France. Electronic address: stefano.poletti@phd.unipd.it.; (Razzini G) Unit of Medical Oncology, Carpi Civil Hospital, Italy. Electronic address: g.razzini-ext@ausl.mo.it.; (Ferrari R) "Mente&Vita" Mindfulness Center, Italy. Electronic address: roberto.ferrari1@gmail.com.; (Ricchieri MP) Local Health Unit of Modena, MBSR Instructor, Italy. Electronic address: m.ricchieri@ausl.mo.it.; (Spedicato GA) FCAS, FSA, C. Stat. Unipol Group, Bologna, Italy. Electronic address: spedygiorgio@gmail.com.; (Pasqualini A) Unit of Medical Oncology, Carpi Civil Hospital, Italy. Electronic address: a.pasqualini@ausl.mo.it.; (Buzzega C) Unit of Early Palliative Care, Carpi Civil Hospital, Italy. Electronic address: c.buzzega@ausl.mo.it.; (Artioli F) Unit of Medical Oncology, Carpi Civil Hospital, Italy. Electronic address: f.artioli@ausl.mo.it.; (Petropulacos K) General Director Health Councillorship Emilia Romagna Region, Bologna, Italy. Electronic address: Kyriakoula.Petropulacos@regione.emilia-romagna.it.; (Luppi M) Department of Medical and Surgical Sciences, AOU, UNIMORE, Italy. Electronic

address: mario.luppi@unimore.it.; (Bandieri E) Unit of Early Palliative Care, Carpi Civil Hospital, Italy. Electronic address: e.bandieri@ausl.mo.it.
(Poletti S) INSERM U1028, CRNL-DYCOG, Lyon, France. Electronic address: stefano.poletti@phd.unipd.it.; (Razzini G) Unit of Medical Oncology, Carpi Civil Hospital, Italy. Electronic address: g.razzini-ext@ausl.mo.it.; (Ferrari R) "Mente&Vita" Mindfulness Center, Italy. Electronic address: roberto.ferrari1@gmail.com.; (Ricchieri MP) Local Health Unit of Modena, MBSR Instructor, Italy. Electronic address: m.ricchieri@ausl.mo.it.; (Spedicato GA) FCAS, FSA, C. Stat. Unipol Group, Bologna, Italy. Electronic address: spedygiorgio@gmail.com.; (Pasqualini A) Unit of Medical Oncology, Carpi Civil Hospital, Italy. Electronic address: a.pasqualini@ausl.mo.it.; (Buzzega C) Unit of Early Palliative Care, Carpi Civil Hospital, Italy. Electronic address: c.buzzega@ausl.mo.it.; (Artioli F) Unit of Medical Oncology, Carpi Civil Hospital, Italy. Electronic address: f.artioli@ausl.mo.it.; (Petropulacos K) General Director Health Councillorship Emilia Romagna Region, Bologna, Italy. Electronic address: Kyriakoula.Petropulacos@regione.emilia-romagna.it.; (Luppi M) Department of Medical and Surgical Sciences, AOU, UNIMORE, Italy. Electronic address: mario.luppi@unimore.it.; (Bandieri E) Unit of Early Palliative Care, Carpi Civil Hospital, Italy. Electronic address: e.bandieri@ausl.mo.it.
(Poletti S) INSERM U1028, CRNL-DYCOG, Lyon, France. Electronic address: stefano.poletti@phd.unipd.it.; (Razzini G) Unit of Medical Oncology, Carpi Civil Hospital, Italy. Electronic address: g.razzini-ext@ausl.mo.it.; (Ferrari R) "Mente&Vita" Mindfulness Center, Italy. Electronic address: roberto.ferrari1@gmail.com.; (Ricchieri MP) Local Health Unit of Modena, MBSR Instructor, Italy. Electronic address: m.ricchieri@ausl.mo.it.; (Spedicato GA) FCAS, FSA, C. Stat. Unipol Group, Bologna, Italy. Electronic address: spedygiorgio@gmail.com.; (Pasqualini A) Unit of Medical Oncology, Carpi Civil Hospital, Italy. Electronic address: a.pasqualini@ausl.mo.it.; (Buzzega C) Unit of Early Palliative Care, Carpi Civil Hospital, Italy. Electronic address: c.buzzega@ausl.mo.it.; (Artioli F) Unit of Medical Oncology, Carpi Civil Hospital, Italy. Electronic address: f.artioli@ausl.mo.it.; (Petropulacos K) General Director Health Councillorship Emilia Romagna Region, Bologna, Italy. Electronic address: Kyriakoula.Petropulacos@regione.emilia-romagna.it.; (Luppi M) Department of Medical and Surgical Sciences, AOU, UNIMORE, Italy. Electronic address: mario.luppi@unimore.it.; (Bandieri E) Unit of Early Palliative Care, Carpi Civil Hospital, Italy. Electronic address: e.bandieri@ausl.mo.it.

(Poletti S) INSERM U1028, CRNL-DYCOG, Lyon, France. Electronic address: stefano.poletti@phd.unipd.it.; (Razzini G) Unit of Medical Oncology, Carpi Civil Hospital, Italy. Electronic address: g.razzini-ext@ausl.mo.it.; (Ferrari R) "Mente&Vita" Mindfulness Center, Italy. Electronic address: roberto.ferrari1@gmail.com.; (Ricchieri MP) Local Health Unit of Modena, MBSR Instructor, Italy. Electronic address: m.ricchieri@ausl.mo.it.; (Spedicato GA) FCAS, FSA, C. Stat. Unipol Group, Bologna, Italy. Electronic address: spedygiorgio@gmail.com.; (Pasqualini A) Unit of Medical Oncology, Carpi Civil Hospital, Italy. Electronic address: a.pasqualini@ausl.mo.it.; (Buzzega C) Unit of Early Palliative Care, Carpi Civil Hospital, Italy. Electronic address: c.buzzega@ausl.mo.it.; (Artioli F) Unit of Medical Oncology, Carpi Civil Hospital, Italy. Electronic address: f.artioli@ausl.mo.it.; (Petropulacos K) General Director Health Councillorship Emilia Romagna Region, Bologna, Italy. Electronic address: Kyriakoula.Petropulacos@regione.emilia-romagna.it.; (Luppi M) Department of Medical and Surgical Sciences, AOU, UNIMORE, Italy. Electronic address: mario.luppi@unimore.it.; (Bandieri E) Unit of Early Palliative Care, Carpi Civil Hospital, Italy. Electronic address: e.bandieri@ausl.mo.it.
(Poletti S) INSERM U1028, CRNL-DYCOG, Lyon, France. Electronic address: stefano.poletti@phd.unipd.it.; (Razzini G) Unit of Medical Oncology, Carpi Civil Hospital, Italy. Electronic address: g.razzini-ext@ausl.mo.it.; (Ferrari R) "Mente&Vita" Mindfulness Center, Italy. Electronic address: roberto.ferrari1@gmail.com.; (Ricchieri MP) Local Health Unit of Modena, MBSR Instructor, Italy. Electronic address: m.ricchieri@ausl.mo.it.; (Spedicato GA) FCAS, FSA, C. Stat. Unipol Group, Bologna, Italy. Electronic address: spedygiorgio@gmail.com.; (Pasqualini A) Unit of Medical Oncology, Carpi Civil Hospital, Italy. Electronic address: a.pasqualini@ausl.mo.it.; (Buzzega C) Unit of Early Palliative Care, Carpi Civil Hospital, Italy. Electronic address: c.buzzega@ausl.mo.it.; (Artioli F) Unit of Medical Oncology, Carpi Civil Hospital, Italy. Electronic address: f.artioli@ausl.mo.it.; (Petropulacos K) General Director Health Councillorship Emilia Romagna Region, Bologna, Italy. Electronic address: Kyriakoula.Petropulacos@regione.emilia-romagna.it.; (Luppi M) Department of Medical and Surgical Sciences, AOU, UNIMORE, Italy. Electronic address: mario.luppi@unimore.it.; (Bandieri E) Unit of Early Palliative Care, Carpi Civil Hospital, Italy. Electronic address: e.bandieri@ausl.mo.it.
Database: PubMed

32. Barriers to Cancer Pain Management Among Nurses in Kenya: A Focused Ethnography.

Author(s): Onsongo LN

Source: Pain management nursing : official journal of the American Society of Pain Management Nurses; 2020; vol. 21 (no. 3); p. 283-289

Publication Date: 2020

Publication Type(s): Journal Article

DOI: http://dx.doi.org/10.1016/j.pmn.2019.08.006

ISSN: 1532-8635

Place of Publication: United States

PubMedID: 31561974

Accession Number: 31561974

Abstract:BACKGROUND: Up to 80% of cancer patients in Kenya suffer from untreated moderate to severe pain.AIM: This study explored barriers to cancer pain management among nurses caring for oncology patients in Kenya. This was part of a larger study whose primary objective was to understand the role of nursing subculture on cancer pain management.DESIGN: A focused ethnographic was used in this study.SETTINGS: An oncology private unit in large referral hospital in Kenya.PARTICIPANTS: Twenty-five (n = 25) nurses participated in this study.METHODS: Semi- structured interviews and observations were used to collect data. Nurses were recruited through purposive, snowball sampling strategy. Content analysis led to identification of key barriers to optimal cancer pain management.RESULTS: Organizational, cognitive, professional and patient/family related barriers to cancer pain management were noted. Specifically, barriers such as lack of accessibility to pain management guidelines and training, professional collaboration, restrictive dispensing guidelines, and opioid related fears were identified.CONCLUSIONS: Interventions should streamline palliative care training and implementation of pain management guidelines in both units. Interventions should consider the influence of different subcultures while implementing pain management policies and training.

Institutions:
(Onsongo LN) From the Lecturer School of Nursing, Kenyatta University, Kenya. Electronic address: Onsongo.lister@ku.ac.ke.
Database: PubMed

33. Maternal Pain Management at Home in Children with Cancer: A Turkish Sample.

Author(s): Boztepe H; Ay A; Akyüz C

Source: Journal of pediatric nursing; 2020; vol. 50 ; p. e99-e106

Publication Date: 2020

Publication Type(s): Journal Article

DOI: http://dx.doi.org/10.1016/j.pedn.2019.08.007

ISSN: 1532-8449

Place of Publication: United States

PubMedID: 31434636

Accession Number: 31434636

Keywords: Subject Terms: Home care; Mother; Pain management; Pediatric nursing; Solid tumor

Abstract:PURPOSE: The purpose of the present study was to examine maternal pain management in children with cancer and the associated factors.DESIGN AND METHODS: The present work is a descriptive and cross-sectional study. Data for the study were obtained from mothers of children in the age group of 0 to 18 years undergoing treatment for solid tumors in Pediatric Oncology Service and Outpatient Clinics (n = 112). We used a questionnaire on parental pain management practices at home, the knowledge about pain and analgesic drugs, Spielberger State-Trait Anxiety Inventory (STAI), and Pain Catastrophizing Scale (PCS) to collect the data.RESULTS: Several mothers taking part in the study reported various misconceptions about the assessment of children's pain, analgesic drugs, and usage

of limited non-pharmacological methods for managing pain in children with cancer. No significant relationships were found between mothers' pain management practices, knowledge of pain assessment and analgesic drugs, and mothers' and children's sociodemographic characteristics or mothers' pain catastrophizing and anxiety about their own pain.CONCLUSIONS: The findings of the study revealed that the majority of mothers of children with cancer had misconceptions regarding knowledge of pain assessment and analgesic drugs; these misconceptions potentially lead to manage children's pain associated with cancer ineffectively. Findings indicate mothers' information and support needs for children's cancer pain management in the home settings.PRACTICE IMPLICATIONS: A further understanding of barriers to parental pain management in children with cancer in the home setting will contribute immensely in developing appropriate management practices.

Institutions:

(Boztepe H) Department of Nursing, Faculty of Health Sciences, Atılım University, İncek, Ankara, Turkey.; (Ay A) Pediatric Nursing Department, Faculty of Nursing, Hacettepe University, Sıhhiye, Ankara, Turkey. Electronic address: ayse0526@gmail.com.; (Akyüz C) Professor Pediatric Oncology Department, Hacettepe University Institute of Oncology, Hacettepe University, Sıhhiye, Ankara, Turkey.
(Boztepe H) Department of Nursing, Faculty of Health Sciences, Atılım University, İncek, Ankara, Turkey.; (Ay A) Pediatric Nursing Department, Faculty of Nursing, Hacettepe University, Sıhhiye, Ankara, Turkey. Electronic address: ayse0526@gmail.com.; (Akyüz C) Professor Pediatric Oncology Department, Hacettepe University Institute of Oncology, Hacettepe University, Sıhhiye, Ankara, Turkey.
(Boztepe H) Department of Nursing, Faculty of Health Sciences, Atılım University, İncek, Ankara, Turkey.; (Ay A) Pediatric Nursing Department, Faculty of Nursing, Hacettepe University, Sıhhiye, Ankara, Turkey. Electronic address: ayse0526@gmail.com.; (Akyüz C) Professor Pediatric Oncology Department, Hacettepe University Institute of Oncology, Hacettepe University, Sıhhiye, Ankara, Turkey.

Database: PubMed

34. Evaluating recruitment methods of patients with advanced cancer: a pragmatic opportunistic comparison.

Author(s): Edwards Z; Bennett MI; Petty D; Blenkinsopp A

Source: The International journal of pharmacy practice; Dec 2019; vol. 27 (no. 6); p. 536-544

Publication Date: Dec 2019

Publication Type(s): Journal Article

DOI: http://dx.doi.org/10.1111/ijpp.12562

ISSN: 2042-7174

Place of Publication: England

PubMedID: 31287212

Accession Number: 31287212

Available at The International journal of pharmacy practice - from Wiley Online Library Medicine and Nursing Collection 2020

Available at The International journal of pharmacy practice - from EBSCO (CINAHL Complete)

Available at The International journal of pharmacy practice - from Unpaywall

Keywords: Subject Terms: cancer; end-of-life; methods; palliative care; recruitment

Abstract:BACKGROUND: Recruitment of patients with advanced cancer into studies is challenging.OBJECTIVE: To evaluate recruitment methods in a study of pharmacist-led cancer pain medicine consultations and produce recommendations for future studies.METHOD: Two methods of recruitment were employed: (1) community-based (general practitioner computer search, identification by general practitioner, community pharmacist or district nurse and hospital outpatient list search) and (2) hospice-based (in and outpatient list search). Patients identified in method 1 were invited by post and in method 2 were invited face-to-face. Information was designed in collaboration with patients and carers.RESULTS: A total of 128 patients were identified (85 from the community and 43 from the hospice), and 47 met the inclusion criteria. Twenty-three agreed to take part and 19 completed the study, 17 of whom were already under specialist palliative care. Recruitment rates were 7% for community-based methods and 40% for hospice. The recruitment methods differed in intensity of resource use. Recruitment via letter and a lack of engagement by healthcare professionals were found to be barriers. Facilitators included the researcher having personal involvement in recruitment.CONCLUSION: The overall recruitment rate was in line with other studies for this patient cohort. Attempts to identify and engage patients through community-based postal contact were less effective than where personal contact with patients was both possible and occurred. Methods were less successful at recruiting patients who were not already engaged with hospice services.

Institutions:
(Edwards Z) University of Bradford, Bradford, West Yorkshire, UK.; (Bennett MI) University of Leeds, Leeds, UK.; (Petty D) University of Bradford, Bradford, West Yorkshire, UK.; (Blenkinsopp A) University of Bradford, Bradford, West Yorkshire, UK.
(Edwards Z) University of Bradford, Bradford, West Yorkshire, UK.; (Bennett MI) University of Leeds, Leeds, UK.; (Petty D) University of Bradford, Bradford, West Yorkshire, UK.; (Blenkinsopp A) University of Bradford, Bradford, West Yorkshire, UK.
(Edwards Z) University of Bradford, Bradford, West Yorkshire, UK.; (Bennett MI) University of Leeds, Leeds, UK.; (Petty D) University of Bradford, Bradford, West Yorkshire, UK.; (Blenkinsopp A) University of Bradford, Bradford, West Yorkshire, UK.
(Edwards Z) University of Bradford, Bradford, West Yorkshire, UK.; (Bennett MI) University of Leeds, Leeds, UK.; (Petty D) University of Bradford, Bradford, West Yorkshire, UK.; (Blenkinsopp A) University of Bradford, Bradford, West Yorkshire, UK.

Database: PubMed

35. Associated factors with the knowledge of nurses of a high complexity oncology centre in Brazil, on the management of cancer pain.

Author(s): Dos Santos Ferreira F; Meira KC; Félix RS; de Oliveira IRS; Pinto CMI; Dos Santos Silva MA; Dos Santos J

Source: Ecancermedicalscience; 2019; vol. 13 ; p. 928

Publication Date: 2019

Publication Type(s): Journal Article

DOI: http://dx.doi.org/10.3332/ecancer.2019.928

ISSN: 1754-6605

Place of Publication: England

PubMedID: 31281425

Accession Number: 31281425

Available at Ecancermedicalscience - from Europe PubMed Central - Open Access

Available at Ecancermedicalscience - from DOAJ - Directory of Open Access Journals

Available at Ecancermedicalscience - from Unpaywall

Keywords: Subject Terms: nursing care; nursing education; oncology nursing; pain management; teaching

Abstract: Pain is one of the most prevalent symptoms in cancer patients and may be directly related to cancer or to the procedures needed for its diagnosis and treatment. It is estimated that about 40% of cancer patients receive inadequate treatment for painful conditions. Among the barriers to adequate pain management are inadequate knowledge and the dysfunctional beliefs of healthcare professionals. Therefore, the present study aims to assess the knowledge of oncology nurses on the management of pain, as well as the factors associated with it. It is a cross-sectional study with 126 nurses working at a High Complexity Oncology Centre in Brazil. Knowledge about the management of cancer pain was evaluated through the instrument 'Nurses' Knowledge on Cancer Pain Management-World Health Organization-developed by Ramos (1994). In the analysis of the association between knowledge about pain management and the independent variables, Poisson regression was used with robust variance, and values of $p \leq 0.05$ were considered statistically significant. Adequate knowledge prevalence was 54.1% confidence intervals (CI 5.40%-62.80%). These nurses differed in relation to those with inadequate knowledge regarding the source of knowledge about pain, the ethical aspects in the treatment of the patient with oncologic pain, and non-pharmacological methods (coeliac plexus neuroleptic block) for pain control. Also, the factors associated with adequate knowledge were longer professional experience time ([10-19 years (ratio prevalence (RP) = 1.72, 95% CI: 1.05-2.81), 20-29 years (RP = 2.56, 95% CI: 1.63-4.02), 30-39 years (RP = 3.45, 95% CI: 2.25-5.29]), and not believing that the use of opioids causes harm to patients corresponded with a greater chance prevalence ratio (PR = 1.20, 95% CI: 1.12-1.20) of having adequate knowledge. The findings of the study point to the need for continuing education, updated education, and reflection, especially for nurses with less professional experience.

Institutions:
(Dos Santos Ferreira F) Florence Clinic: Rehabilitation and Palliative Care, Bela Vista do Cabral Street, 271, Nazaré, BA, Brazil.; (Meira KC) Health School Rio Grande do Norte Federal University, Senador Salgado Filho, Avenue, s/n Lagoa

Nova, Natal, RN, Brazil.; (Félix RS) Health School Rio Grande do Norte Federal University, Senador Salgado Filho, Avenue, s/n Lagoa Nova, Natal, RN, Brazil.; (de Oliveira IRS) Health School Rio Grande do Norte Federal University, Senador Salgado Filho, Avenue, s/n Lagoa Nova, Natal, RN, Brazil.; (Pinto CMI) Medical-surgical Nursing Department, Anna Néry Nursing School/UFRJ, Afonso Cavalcanti Street, 275-Cidade Nova, Rio de Janeiro, RJ, Brazil.; (Dos Santos Silva MA) Nursing Department of Paulista University- Jacery Street, 247-Morumbi, São Paulo, SP, Brazil.; (Dos Santos J) José de Alencar Gomes da Silva National Institute of Cancer-INCA Cruz Vermelha Square, 23-Centro, Rio de Janeiro, RJ, Brazil.

(Dos Santos Ferreira F) Florence Clinic: Rehabilitation and Palliative Care, Bela Vista do Cabral Street, 271, Nazaré, BA, Brazil.; (Meira KC) Health School Rio Grande do Norte Federal University, Senador Salgado Filho, Avenue, s/n Lagoa Nova, Natal, RN, Brazil.; (Félix RS) Health School Rio Grande do Norte Federal University, Senador Salgado Filho, Avenue, s/n Lagoa Nova, Natal, RN, Brazil.; (de Oliveira IRS) Health School Rio Grande do Norte Federal University, Senador Salgado Filho, Avenue, s/n Lagoa Nova, Natal, RN, Brazil.; (Pinto CMI) Medical-surgical Nursing Department, Anna Néry Nursing School/UFRJ, Afonso Cavalcanti Street, 275-Cidade Nova, Rio de Janeiro, RJ, Brazil.; (Dos Santos Silva MA) Nursing Department of Paulista University- Jacery Street, 247-Morumbi, São Paulo, SP, Brazil.; (Dos Santos J) José de Alencar Gomes da Silva National Institute of Cancer-INCA Cruz Vermelha Square, 23-Centro, Rio de Janeiro, RJ, Brazil.

(Dos Santos Ferreira F) Florence Clinic: Rehabilitation and Palliative Care, Bela Vista do Cabral Street, 271, Nazaré, BA, Brazil.; (Meira KC) Health School Rio Grande do Norte Federal University, Senador Salgado Filho, Avenue, s/n Lagoa Nova, Natal, RN, Brazil.; (Félix RS) Health School Rio Grande do Norte Federal University, Senador Salgado Filho, Avenue, s/n Lagoa Nova, Natal, RN, Brazil.; (de Oliveira IRS) Health School Rio Grande do Norte Federal University, Senador Salgado Filho, Avenue, s/n Lagoa Nova, Natal, RN, Brazil.; (Pinto CMI) Medical-surgical Nursing Department, Anna Néry Nursing School/UFRJ, Afonso Cavalcanti Street, 275-Cidade Nova, Rio de Janeiro, RJ, Brazil.; (Dos Santos Silva MA) Nursing Department of Paulista University- Jacery Street, 247-Morumbi, São Paulo, SP, Brazil.; (Dos Santos J) José de Alencar Gomes da Silva National Institute of Cancer-INCA Cruz Vermelha Square, 23-Centro, Rio de Janeiro, RJ, Brazil.

(Dos Santos Ferreira F) Florence Clinic: Rehabilitation and Palliative Care, Bela Vista do Cabral Street, 271, Nazaré, BA, Brazil.; (Meira KC) Health School Rio Grande do Norte Federal University, Senador Salgado Filho, Avenue, s/n Lagoa Nova, Natal, RN, Brazil.; (Félix RS) Health School Rio Grande do Norte Federal University, Senador Salgado Filho, Avenue, s/n Lagoa Nova, Natal, RN, Brazil.; (Pinto CMI) Medical-surgical Nursing Department, Anna Néry Nursing School/UFRJ, Afonso Cavalcanti Street, 275-Cidade Nova, Rio de Janeiro, RJ, Brazil.; (Dos Santos Silva MA) Nursing Department of Paulista University- Jacery Street, 247-Morumbi, São Paulo, SP, Brazil.; (Dos Santos J) José de Alencar Gomes da Silva National Institute of Cancer-INCA Cruz Vermelha Square, 23-Centro, Rio de Janeiro, RJ, Brazil.

(Dos Santos Ferreira F) Florence Clinic: Rehabilitation and Palliative Care, Bela Vista do Cabral Street, 271, Nazaré, BA, Brazil.; (Meira KC) Health School Rio Grande do Norte Federal University, Senador Salgado Filho, Avenue, s/n Lagoa Nova, Natal, RN, Brazil.; (Félix RS) Health School Rio Grande do Norte Federal University, Senador Salgado Filho, Avenue, s/n Lagoa Nova, Natal, RN, Brazil.; (de

Oliveira IRS) Health School Rio Grande do Norte Federal University, Senador Salgado Filho, Avenue, s/n Lagoa Nova, Natal, RN, Brazil.; (Pinto CMI) Medical-surgical Nursing Department, Anna Néry Nursing School/UFRJ, Afonso Cavalcanti Street, 275-Cidade Nova, Rio de Janeiro, RJ, Brazil.; (Dos Santos Silva MA) Nursing Department of Paulista University- Jacery Street, 247-Morumbi, São Paulo, SP, Brazil.; (Dos Santos J) José de Alencar Gomes da Silva National Institute of Cancer-INCA Cruz Vermelha Square, 23-Centro, Rio de Janeiro, RJ, Brazil.
(Dos Santos Ferreira F) Florence Clinic: Rehabilitation and Palliative Care, Bela Vista do Cabral Street, 271, Nazaré, BA, Brazil.; (Meira KC) Health School Rio Grande do Norte Federal University, Senador Salgado Filho, Avenue, s/n Lagoa Nova, Natal, RN, Brazil.; (Félix RS) Health School Rio Grande do Norte Federal University, Senador Salgado Filho, Avenue, s/n Lagoa Nova, Natal, RN, Brazil.; (de Oliveira IRS) Health School Rio Grande do Norte Federal University, Senador Salgado Filho, Avenue, s/n Lagoa Nova, Natal, RN, Brazil.; (Pinto CMI) Medical-surgical Nursing Department, Anna Néry Nursing School/UFRJ, Afonso Cavalcanti Street, 275-Cidade Nova, Rio de Janeiro, RJ, Brazil.; (Dos Santos Silva MA) Nursing Department of Paulista University- Jacery Street, 247-Morumbi, São Paulo, SP, Brazil.; (Dos Santos J) José de Alencar Gomes da Silva National Institute of Cancer-INCA Cruz Vermelha Square, 23-Centro, Rio de Janeiro, RJ, Brazil.
(Dos Santos Ferreira F) Florence Clinic: Rehabilitation and Palliative Care, Bela Vista do Cabral Street, 271, Nazaré, BA, Brazil.; (Meira KC) Health School Rio Grande do Norte Federal University, Senador Salgado Filho, Avenue, s/n Lagoa Nova, Natal, RN, Brazil.; (Félix RS) Health School Rio Grande do Norte Federal University, Senador Salgado Filho, Avenue, s/n Lagoa Nova, Natal, RN, Brazil.; (de Oliveira IRS) Health School Rio Grande do Norte Federal University, Senador Salgado Filho, Avenue, s/n Lagoa Nova, Natal, RN, Brazil.; (Pinto CMI) Medical-surgical Nursing Department, Anna Néry Nursing School/UFRJ, Afonso Cavalcanti Street, 275-Cidade Nova, Rio de Janeiro, RJ, Brazil.; (Dos Santos Silva MA) Nursing Department of Paulista University- Jacery Street, 247-Morumbi, São Paulo, SP, Brazil.; (Dos Santos J) José de Alencar Gomes da Silva National Institute of Cancer-INCA Cruz Vermelha Square, 23-Centro, Rio de Janeiro, RJ, Brazil.
Database: PubMed

36. Attitudes of Hospice Providers Regarding Intrathecal Targeted Drug Delivery for Patients With Cancer.

Author(s): Warner LL; Moeschler SS; Pittelkow TP; Strand JJ

Source: The American journal of hospice & palliative care; Nov 2019; vol. 36 (no. 11); p. 955-958

Publication Date: Nov 2019

Publication Type(s): Journal Article

DOI: http://dx.doi.org/10.1177/1049909119852928

ISSN: 1938-2715

Place of Publication: United States

PubMedID: 31132860

Accession Number: 31132860

Keywords: Subject Terms: cancer pain; hospice pain; hospice survey; intrathecal drug delivery system; intrathecal targeted drug delivery; pain pump; palliative pain

Abstract: Pain is one of the most commonly experienced and feared symptoms faced by patients with a serious illness. For these patients, intrathecal drug delivery systems (IDDSs) provide greater potency and/or few systemic side effects. However, despite these benefits, the integration and management of IDDS for patients receiving hospice care has not been previous studied. An electronic, 18-question survey was sent to 200 hospice practitioners (physicians, nurse practitioners and nurses) in the state of Minnesota to explore their experience, confidence, and the perceived barriers to caring for patients with IDDS while being cared for on hospice. Providers were identified though mailing lists from the Minnesota Network of Hospice and Palliative Care organization. The survey was administered by the Mayo Clinic Survey Research Center with institutional review board approval. Slightly more than 50% of respondents have ever cared for a patient with an intrathecal pump. If a patient had a pump in place, only 28% of providers expressed confidence in managing their pain. Additionally, only 3 of 10 respondents felt that adjusting an intrathecal pump should be the first option when a patient with an IDDS in place had increased pain. Indeed, the vast majority (over 80%) of respondents preferred the use of systemic therapies for primary pain management. Access to IDDS vendors for changes/refills in the home is identified as another barrier with over 50% of respondents either unaware of an available vendor or reporting no vendor available. There are numerous self-reported barriers to ongoing use of IDDS with patients receiving hospice care.

Institutions:
(Warner LL) 1 Department of Anesthesiology, Mayo Clinic, Rochester, MN, USA.; (Moeschler SS) 2 Division of Pain Medicine, Department of Anesthesiology, Mayo Clinic, Rochester, MN, USA.; (Pittelkow TP) 2 Division of Pain Medicine, Department of Anesthesiology, Mayo Clinic, Rochester, MN, USA.; (Strand JJ) 3 Division of Palliative Care, Department of Internal Medicine, Mayo Clinic, Rochester, MN, USA.
(Warner LL) 1 Department of Anesthesiology, Mayo Clinic, Rochester, MN, USA.; (Moeschler SS) 2 Division of Pain Medicine, Department of Anesthesiology, Mayo Clinic, Rochester, MN, USA.; (Pittelkow TP) 2 Division of Pain Medicine, Department of Anesthesiology, Mayo Clinic, Rochester, MN, USA.; (Strand JJ) 3 Division of Palliative Care, Department of Internal Medicine, Mayo Clinic, Rochester, MN, USA.
(Warner LL) 1 Department of Anesthesiology, Mayo Clinic, Rochester, MN, USA.; (Moeschler SS) 2 Division of Pain Medicine, Department of Anesthesiology, Mayo Clinic, Rochester, MN, USA.; (Pittelkow TP) 2 Division of Pain Medicine, Department of Anesthesiology, Mayo Clinic, Rochester, MN, USA.; (Strand JJ) 3 Division of Palliative Care, Department of Internal Medicine, Mayo Clinic, Rochester, MN, USA.
(Warner LL) 1 Department of Anesthesiology, Mayo Clinic, Rochester, MN, USA.; (Moeschler SS) 2 Division of Pain Medicine, Department of Anesthesiology, Mayo Clinic, Rochester, MN, USA.; (Pittelkow TP) 2 Division of Pain Medicine, Department of Anesthesiology, Mayo Clinic, Rochester, MN, USA.; (Strand JJ) 3 Division of Palliative Care, Department of Internal Medicine, Mayo Clinic, Rochester, MN, USA.

Database: PubMed

37. Nurses' knowledge, perceived barriers, and practices regarding cancer pain management: a cross-sectional study from Palestine.

Author(s): Toba HA; Samara AM; Zyoud SH
Source: BMC medical education; May 2019; vol. 19 (no. 1); p. 167
Publication Date: May 2019
Publication Type(s): Journal Article
DOI: http://dx.doi.org/10.1186/s12909-019-1613-z
ISSN: 1472-6920
Place of Publication: England
PubMedID: 31122222
Accession Number: 31122222
Available at BMC medical education - from BioMed Central
Available at BMC medical education - from Europe PubMed Central - Open Access
Available at BMC medical education - from ProQuest (Health Research Premium) - NHS Version
Available at BMC medical education - from EBSCO (MEDLINE Complete)
Available at BMC medical education - from Unpaywall
Keywords: Subject Terms: Cancer pain; Knowledge; Nurses; Palestine; Perceived barriers; Practices
Abstract:BACKGROUND: Accurate knowledge and good pain evaluation and documentation practices should be present for efficient pain management. In this study, we aimed to assess the knowledge and practices of nurses relating to the management of cancer pain in Palestine, and to determine the barriers to efficient pain control in cancer patients.METHODS: A cross-sectional survey took place at 8 hospitals across Northern West Bank. A convenience sample of 220 Nurses working in governmental and private hospitals in West Bank/Palestine was studied. For that purpose, a questionnaire was developed to assess knowledge, practices, perceived barriers, and delaying processes relating to cancer pain management (CPM).RESULTS: In total, 220 questionnaires were completed with a response rate of 88%. Participants' mean age was 30.34 years. Overall, 69.5% worked in governmental hospitals, 26.8% worked in the private sector and the remainder worked in both governmental and private sectors. The correct response rate to questions that assess knowledge relating to cancer pain control was calculated and a mean knowledge score was found to be 5.1 with a standard deviation of 2.1. A relationship between the knowledge score and the sample characteristics was made and showed that males scored significantly higher ($p = 0.001$) than females with median scores of 6 [4-7] and 5 [3-6] for males and females, respectively. Inadequate pain assessment (76.8%), insufficient knowledge of pain control (70.5%) and strict regulation on opioid use (69.5%) were the most frequently perceived barriers. Nurses reported that they would assess pain on every round and check all items related to pain assessment. Contacting the physician for the prescription of opioids was cited as the main delaying process by 56.4% of participants.CONCLUSIONS: This study allowed us to recognise the knowledge deficit and the barriers to effective management. On the other hand, the analysis has shown good pain documentation practices among nurses. Those knowledge deficits demonstrate the need for more education about CPM. The improvement of coordination and communication between physicians and nurses seems to play a crucial role in CPM, as contacting physicians was cited as the most delaying process in CPM by nurses.

Institutions:
(Toba HA) Department of Medicine, College of Medicine and Health Sciences, An-Najah National University, Nablus, 44839, Palestine.; (Samara AM) Department of Medicine, College of Medicine and Health Sciences, An-Najah National University, Nablus, 44839, Palestine.; (Zyoud SH) Poison Control and Drug Information Center (PCDIC), College of Medicine and Health Sciences, An-Najah National University, Nablus, 44839, Palestine. saedzyoud@yahoo.com.
(Toba HA) Department of Medicine, College of Medicine and Health Sciences, An-Najah National University, Nablus, 44839, Palestine.; (Samara AM) Department of Medicine, College of Medicine and Health Sciences, An-Najah National University, Nablus, 44839, Palestine.; (Zyoud SH) Poison Control and Drug Information Center (PCDIC), College of Medicine and Health Sciences, An-Najah National University, Nablus, 44839, Palestine. saedzyoud@yahoo.com.
(Toba HA) Department of Medicine, College of Medicine and Health Sciences, An-Najah National University, Nablus, 44839, Palestine.; (Samara AM) Department of Medicine, College of Medicine and Health Sciences, An-Najah National University, Nablus, 44839, Palestine.; (Zyoud SH) Poison Control and Drug Information Center (PCDIC), College of Medicine and Health Sciences, An-Najah National University, Nablus, 44839, Palestine. saedzyoud@yahoo.com.
Database: PubMed

38. Managing Pain in People with Cancer-a Systematic Review of the Attitudes and Knowledge of Professionals, Patients, Caregivers and Public.

Author(s): Makhlouf SM; Pini S; Ahmed S; Bennett MI

Source: Journal of cancer education : the official journal of the American Association for Cancer Education; 2020; vol. 35 (no. 2); p. 214-240

Publication Date: 2020

Publication Type(s): Journal Article; Research Support, Non-U.S. Gov't; Systematic Review

DOI: http://dx.doi.org/10.1007/s13187-019-01548-9

ISSN: 1543-0154

Place of Publication: England

PubMedID: 31119708

Accession Number: 31119708

Available at Journal of cancer education : the official journal of the American Association for Cancer Education - from EBSCO (MEDLINE Complete)

Available at Journal of cancer education : the official journal of the American Association for Cancer Education - from ProQuest (MEDLINE with Full Text) - NHS Version

Available at Journal of cancer education : the official journal of the American Association for Cancer Education - from ProQuest (Health Research Premium) - NHS Version

Available at Journal of cancer education : the official journal of the American Association for Cancer Education - from Unpaywall

Keywords: Subject Terms: *Attitudes and knowledge; *Cancer pain management; *Caregivers; *Patients; *Professionals; *Public; *Systematic review

Abstract: Cancer pain is a common symptom experienced by patients, caused either by the disease or its treatment. Morphine remains the most effective and recommended treatment for cancer pain. However, cancer patients still do not receive appropriate management for their pain, and under-treatment is common. Lack of knowledge and negative attitudes towards cancer pain and analgesia among professionals, patients and family caregivers are reported as one of the most common barriers to effective cancer pain management (CPM). To systematically review research on the nature and impact of attitudes and knowledge towards CPM, a systematic literature search of 6 databases (the Cochrane library, MEDLINE, PsycINFO, CINAHL, Web of Science and EMBASE) was undertaken in July 2018. Additionally, hand-searching of Google, Google Scholar and reference lists was conducted. The inclusion criteria were adult (18-65 years of age), studies which included attitudes and knowledge towards CPM, studies written in English, published literature only and cross-sectional design. Included studies were critically appraised by two researchers independently using the Joanna Briggs Institute Analytical Cross Sectional Studies Assessment (JBI-ACSSA). A total of 36 studies met the inclusion criteria. The main finding was that among professionals, patients, caregivers and the public there were similar attitudinal barriers to effective CPM. The most commonly cited barriers were fear of drug addiction, tolerance of medication and side effects of opioids. We also found differences between professional groups (physicians versus nurses) and between different countries based on their potential exposure to palliative care training and services. There are still barriers to effective CPM, which might result in unrelieved cancer pain. Therefore, more educational programmes and training for professionals on CPM are needed. Furthermore, patients, caregivers, and the public need more general awareness and adequate level of knowledge about CPM.

Institutions:
(Makhlouf SM) Academic Unit of Palliative Care, Leeds Institute of Health Sciences, School of Medicine, University of Leeds, Level 10 Worsley Building, Clarendon Way, Leeds, LS2 9NL, UK. umsmam@leeds.ac.uk.; (Pini S) Academic Unit of Palliative Care, Leeds Institute of Health Sciences, School of Medicine, University of Leeds, Level 10 Worsley Building, Clarendon Way, Leeds, LS2 9NL, UK.; (Ahmed S) Academic Unit of Palliative Care, Leeds Institute of Health Sciences, School of Medicine, University of Leeds, Level 10 Worsley Building, Clarendon Way, Leeds, LS2 9NL, UK.; (Bennett MI) Academic Unit of Palliative Care, Leeds Institute of Health Sciences, School of Medicine, University of Leeds, Level 10 Worsley Building, Clarendon Way, Leeds, LS2 9NL, UK.
(Makhlouf SM) Academic Unit of Palliative Care, Leeds Institute of Health Sciences, School of Medicine, University of Leeds, Level 10 Worsley Building, Clarendon Way, Leeds, LS2 9NL, UK. umsmam@leeds.ac.uk.; (Pini S) Academic Unit of Palliative Care, Leeds Institute of Health Sciences, School of Medicine, University of Leeds, Level 10 Worsley Building, Clarendon Way, Leeds, LS2 9NL, UK.; (Ahmed S) Academic Unit of Palliative Care, Leeds Institute of Health Sciences, School of Medicine, University of Leeds, Level 10 Worsley Building, Clarendon Way, Leeds, LS2 9NL, UK.; (Bennett MI) Academic Unit of Palliative Care, Leeds Institute of Health Sciences, School of Medicine, University of Leeds, Level 10 Worsley Building, Clarendon Way, Leeds, LS2 9NL, UK.

(Makhlouf SM) Academic Unit of Palliative Care, Leeds Institute of Health Sciences, School of Medicine, University of Leeds, Level 10 Worsley Building, Clarendon Way, Leeds, LS2 9NL, UK. umsmam@leeds.ac.uk.; (Pini S) Academic Unit of Palliative Care, Leeds Institute of Health Sciences, School of Medicine, University of Leeds, Level 10 Worsley Building, Clarendon Way, Leeds, LS2 9NL, UK.; (Ahmed S) Academic Unit of Palliative Care, Leeds Institute of Health Sciences, School of Medicine, University of Leeds, Level 10 Worsley Building, Clarendon Way, Leeds, LS2 9NL, UK.; (Bennett MI) Academic Unit of Palliative Care, Leeds Institute of Health Sciences, School of Medicine, University of Leeds, Level 10 Worsley Building, Clarendon Way, Leeds, LS2 9NL, UK.
(Makhlouf SM) Academic Unit of Palliative Care, Leeds Institute of Health Sciences, School of Medicine, University of Leeds, Level 10 Worsley Building, Clarendon Way, Leeds, LS2 9NL, UK. umsmam@leeds.ac.uk.; (Pini S) Academic Unit of Palliative Care, Leeds Institute of Health Sciences, School of Medicine, University of Leeds, Level 10 Worsley Building, Clarendon Way, Leeds, LS2 9NL, UK.; (Ahmed S) Academic Unit of Palliative Care, Leeds Institute of Health Sciences, School of Medicine, University of Leeds, Level 10 Worsley Building, Clarendon Way, Leeds, LS2 9NL, UK.; (Bennett MI) Academic Unit of Palliative Care, Leeds Institute of Health Sciences, School of Medicine, University of Leeds, Level 10 Worsley Building, Clarendon Way, Leeds, LS2 9NL, UK.
Database: PubMed

39. An Overview of Cancer Pain: Epidemiology and Pathophysiology.

Author(s): Russo MM; Sundaramurthi T

Source: Seminars in oncology nursing; 2019; vol. 35 (no. 3); p. 223-228

Publication Date: 2019

Publication Type(s): Journal Article; Review

DOI: http://dx.doi.org/10.1016/j.soncn.2019.04.002

ISSN: 1878-3449

Place of Publication: United States

PubMedID: 31085106

Accession Number: 31085106

Available at Seminars in Oncology Nursing - from ScienceDirect

Keywords: Subject Terms: *Cancer pain; *Cancer pain epidemiology; *Cancer pain pathophysiology; *Cancer pain syndromes

Abstract:OBJECTIVE: To present an overview of the epidemiology and pathophysiology of cancer pain related to disease and treatment.DATA SOURCES: Published manuscripts, Web sites, and textbook chapters.CONCLUSION: Current knowledge of cancer pain epidemiology and pathophysiology widens and focuses the opportunities to prevent, limit, and treat cancer pain.IMPLICATIONS FOR NURSING PRACTICE: Nurses' knowledge of transduction, transmission, perception, and modulation in cancer pain pathophysiology outlines pathways for multimodal approaches to treat complex and diverse pain experiences. Use of standard vocabulary of pain terms and definitions facilitates pain assessment and management across different disciplines.

Institutions:
(Russo MM) University of Maryland Medical Center and University of Maryland Graduate School, Baltimore MD. Electronic address: margueriterusso@umm.edu.; (Sundaramurthi T) Veterans Affairs Medical Center, Washington DC.
(Russo MM) University of Maryland Medical Center and University of Maryland Graduate School, Baltimore MD. Electronic address: margueriterusso@umm.edu.; (Sundaramurthi T) Veterans Affairs Medical Center, Washington DC.
Database: PubMed

40. Genetic Variants Associated with Cancer Pain and Response to Opioid Analgesics: Implications for Precision Pain Management.

Author(s): Yang GS; Barnes NM; Lyon DE; Dorsey SG

Source: Seminars in oncology nursing; 2019; vol. 35 (no. 3); p. 291-299

Publication Date: 2019

Publication Type(s): Journal Article; Research Support, N.I.H., Extramural; Review

DOI: http://dx.doi.org/10.1016/j.soncn.2019.04.011

ISSN: 1878-3449

Place of Publication: United States

PubMedID: 31085105

Accession Number: 31085105

Available at Seminars in oncology nursing - from ScienceDirect

Available at Seminars in oncology nursing - from Unpaywall

Keywords: Subject Terms: *Biomarker; *Cancer; *Genetic polymorphism; *Genetic variant; *Pain; *Precision medicine

Abstract:OBJECTIVE: To review the current knowledge on the association of genetic variants with cancer pain.DATA SOURCES: Data-based publications and review articles retrieved from PubMed, CINAHL, and Web of Science, as well as an additional search in Google Scholar.CONCLUSION: Genetic variability can influence differential pain perception and response to opioids in cancer patients, which will have implications in the optimal personalized treatment of cancer pain. More studies are warranted to replicate findings.IMPLICATIONS FOR NURSING PRACTICE: Nurses are poised to educate patients on biomarker testing and interpretation and to use precision pain management strategies based on this information.

Institutions:
(Yang GS) University of Florida College of Nursing, Gainesville, FL. Electronic address: gyang1@ufl.edu.; (Barnes NM) University of Florida College of Nursing, Gainesville, FL.; (Lyon DE) University of Florida College of Nursing, Gainesville, FL.; (Dorsey SG) University of Maryland School of Nursing, Department of Pain and Translational Symptom Science, Baltimore, MD. Electronic address: sdorsey@umaryland.edu.
(Yang GS) University of Florida College of Nursing, Gainesville, FL. Electronic address: gyang1@ufl.edu.; (Barnes NM) University of Florida College of Nursing, Gainesville, FL.; (Lyon DE) University of Florida College of Nursing, Gainesville, FL.; (Dorsey SG) University of Maryland School of Nursing, Department of Pain and

Translational Symptom Science, Baltimore, MD. Electronic address: sdorsey@umaryland.edu.
(Yang GS) University of Florida College of Nursing, Gainesville, FL. Electronic address: gyang1@ufl.edu.; (Barnes NM) University of Florida College of Nursing, Gainesville, FL.; (Lyon DE) University of Florida College of Nursing, Gainesville, FL.; (Dorsey SG) University of Maryland School of Nursing, Department of Pain and Translational Symptom Science, Baltimore, MD. Electronic address: sdorsey@umaryland.edu.
(Yang GS) University of Florida College of Nursing, Gainesville, FL. Electronic address: gyang1@ufl.edu.; (Barnes NM) University of Florida College of Nursing, Gainesville, FL.; (Lyon DE) University of Florida College of Nursing, Gainesville, FL.; (Dorsey SG) University of Maryland School of Nursing, Department of Pain and Translational Symptom Science, Baltimore, MD. Electronic address: sdorsey@umaryland.edu.
Database: PubMed

41. Understanding the behavioural determinants of opioid prescribing among family physicians: a qualitative study.

Author(s): Desveaux L; Saragosa M; Kithulegoda N; Ivers NM

Source: BMC family practice; ; vol. 20 (no. 1); p. 59

Publication Type(s): Journal Article; Research Support, Non-U.S. Gov't

DOI: http://dx.doi.org/10.1186/s12875-019-0947-2

ISSN: 1471-2296

Place of Publication: England

PubMedID: 31077137

Accession Number: 31077137

Available at BMC Family Practice - from BioMed Central

Available at BMC Family Practice - from Europe PubMed Central - Open Access

Available at BMC Family Practice - from ProQuest (Health Research Premium) - NHS Version

Available at BMC Family Practice - from EBSCO (MEDLINE Complete)

Available at BMC Family Practice - from Unpaywall

Keywords: Subject Terms: *Opioid; *Prescribing; *Qualitative; *Theoretical domains framework

Abstract:BACKGROUND: Longstanding variation in the views of family physicians (FPs) on the role of opioids seems to translate into widely varying prescribing rates. Improvement interventions are unlikely to achieve change if they do not understand and explicitly target the factors that determine physician prescribing behaviour. The aim of this work was to understand (1) the perspectives of FPs as it relates to opioid prescribing, and (2) the perceived barriers and enablers to guideline-adherent opioid prescribing and management of chronic non-cancer pain.METHODS: A qualitative study involving one-on-one, semi-structured interviews with a sample of FPs in Ontario, Canada. Interviews were analyzed using a directed content analysis informed by the Theoretical Domains Framework. A framework approach was used to explore interaction across behavioural determinants (factors influencing

behaviour) as well as demographic sources of variation. The behaviour of interest for the current study was the prescribing of opioid medications (including initiation, renewal, and dose reduction) for patients with chronic, non-cancer pain. Associated issues in the overall management of such patients were also explored.RESULTS: Interviews were conducted with 22 FPs. Behavioural determinants interacted with one another to influence FPs prescribing behavior. The TDF domain Beliefs about Consequences played a central role in explaining physician prescribing behaviours as they related to the management of chronic non-cancer pain. Individual beliefs about prescribing consequences and patient behaviour interacted with prescriber beliefs about capabilities and perceptions of the FP's professional role to influence prescriber behaviour. Emotion and the environmental context influenced the impact of these determinants on opioid prescribing and the management of chronic non-cancer pain.CONCLUSIONS: FPs face a wide range of complex (and often interacting) challenges when prescribing opioid therapy to their patients. Solution-based strategies should target these determinants directly using evidence-based strategies that move beyond guideline dissemination and general education. Shared decision-making strategies and patient-facing decision aids are likely to decrease the tension experienced in challenging conversations.
Institutions:
(Desveaux L) Women's College Research Institute, Women's College Hospital, 76 Grenville Ave, Toronto, ON, M5S 1B2, Canada. laura.desveaux@wchospital.ca.; (Saragosa M) Women's College Research Institute, Women's College Hospital, 76 Grenville Ave, Toronto, ON, M5S 1B2, Canada.; (Kithulegoda N) Women's College Research Institute, Women's College Hospital, 76 Grenville Ave, Toronto, ON, M5S 1B2, Canada.; (Ivers NM) Women's College Research Institute, Women's College Hospital, 76 Grenville Ave, Toronto, ON, M5S 1B2, Canada.
(Desveaux L) Women's College Research Institute, Women's College Hospital, 76 Grenville Ave, Toronto, ON, M5S 1B2, Canada. laura.desveaux@wchospital.ca.; (Saragosa M) Women's College Research Institute, Women's College Hospital, 76 Grenville Ave, Toronto, ON, M5S 1B2, Canada.; (Kithulegoda N) Women's College Research Institute, Women's College Hospital, 76 Grenville Ave, Toronto, ON, M5S 1B2, Canada.; (Ivers NM) Women's College Research Institute, Women's College Hospital, 76 Grenville Ave, Toronto, ON, M5S 1B2, Canada.
(Desveaux L) Women's College Research Institute, Women's College Hospital, 76 Grenville Ave, Toronto, ON, M5S 1B2, Canada. laura.desveaux@wchospital.ca.; (Saragosa M) Women's College Research Institute, Women's College Hospital, 76 Grenville Ave, Toronto, ON, M5S 1B2, Canada.; (Kithulegoda N) Women's College Research Institute, Women's College Hospital, 76 Grenville Ave, Toronto, ON, M5S 1B2, Canada.; (Ivers NM) Women's College Research Institute, Women's College Hospital, 76 Grenville Ave, Toronto, ON, M5S 1B2, Canada.
(Desveaux L) Women's College Research Institute, Women's College Hospital, 76 Grenville Ave, Toronto, ON, M5S 1B2, Canada. laura.desveaux@wchospital.ca.; (Saragosa M) Women's College Research Institute, Women's College Hospital, 76 Grenville Ave, Toronto, ON, M5S 1B2, Canada.; (Kithulegoda N) Women's College Research Institute, Women's College Hospital, 76 Grenville Ave, Toronto, ON, M5S 1B2, Canada.; (Ivers NM) Women's College Research Institute, Women's College Hospital, 76 Grenville Ave, Toronto, ON, M5S 1B2, Canada.
Database: PubMed

42. The Global Burden of Cancer Pain.

Author(s): Can G; Mushani T; Rajhi BHA; Brant JM

Source: Seminars in oncology nursing; 2019; vol. 35 (no. 3); p. 315-321

Publication Date: 2019

Publication Type(s): Journal Article; Review

DOI: http://dx.doi.org/10.1016/j.soncn.2019.04.014

ISSN: 1878-3449

Place of Publication: United States

PubMedID: 31076099

Accession Number: 31076099

Available at Seminars in oncology nursing - from ScienceDirect

Keywords: Subject Terms: *Cancer pain; *Culture; *Global health; *Opioid availability; *Religion

Abstract:OBJECTIVES: To provide an overview of the global disparities in cancer pain management. To discuss cultural, religious, and spiritual considerations in cancer pain assessment and management.DATA SOURCES: Peer-reviewed articles, book chapters, Internet.CONCLUSION: Significant disparities in pain management exist globally, especially in developing countries. Cultural and religious differences influence pain care and opioid availability is lacking in many countries. Significant barriers impede good pain management; however, some countries have made positive strides in improving pain management for their population.IMPLICATIONS FOR NURSING PRACTICE: Globally, nurses have a vital role in recognizing and addressing barriers to good pain management and can be ambassadors to advocate for improved pain assessment and management globally.

Institutions:
(Can G) Istanbul University - Cerrahpasa, Florence Nightingale Nursing Faculty, Abide-i Hurriyet Cad, Caglayan, Istanbul, Turkey.; (Mushani T) University Health Network, Toronto, ON, Canada; Aga Khan University School of Nursing and Midwifery, Nairobi.; (Rajhi BHA) Jalan Bani Bu Ali Hospital, South Sharqiya Governorate, Sultanate of Oman.; (Brant JM) Collaborative Science and Innovation, Billings Clinic, Billings, MT, USA. Electronic address: jbrant@billingsclinic.org.
(Can G) Istanbul University - Cerrahpasa, Florence Nightingale Nursing Faculty, Abide-i Hurriyet Cad, Caglayan, Istanbul, Turkey.; (Mushani T) University Health Network, Toronto, ON, Canada; Aga Khan University School of Nursing and Midwifery, Nairobi.; (Rajhi BHA) Jalan Bani Bu Ali Hospital, South Sharqiya Governorate, Sultanate of Oman.; (Brant JM) Collaborative Science and Innovation, Billings Clinic, Billings, MT, USA. Electronic address: jbrant@billingsclinic.org.
(Can G) Istanbul University - Cerrahpasa, Florence Nightingale Nursing Faculty, Abide-i Hurriyet Cad, Caglayan, Istanbul, Turkey.; (Mushani T) University Health Network, Toronto, ON, Canada; Aga Khan University School of Nursing and Midwifery, Nairobi.; (Rajhi BHA) Jalan Bani Bu Ali Hospital, South Sharqiya Governorate, Sultanate of Oman.; (Brant JM) Collaborative Science and Innovation, Billings Clinic, Billings, MT, USA. Electronic address: jbrant@billingsclinic.org.
(Can G) Istanbul University - Cerrahpasa, Florence Nightingale Nursing Faculty, Abide-i Hurriyet Cad, Caglayan, Istanbul, Turkey.; (Mushani T) University Health Network, Toronto, ON, Canada; Aga Khan University School of Nursing and

Midwifery, Nairobi.; (Rajhi BHA) Jalan Bani Bu Ali Hospital, South Sharqiya Governorate, Sultanate of Oman.; (Brant JM) Collaborative Science and Innovation, Billings Clinic, Billings, MT, USA. Electronic address: jbrant@billingsclinic.org.
Database: PubMed

43. Physician-pharmacist collaboration on chronic non-cancer pain management during the opioid crisis: A qualitative interview study.

Author(s): Kang I; Urick B; Vohra R; Ives TJ

Source: Research in social & administrative pharmacy : RSAP; 2019; vol. 15 (no. 8); p. 1027-1031

Publication Date: 2019

Publication Type(s): Journal Article

DOI: http://dx.doi.org/10.1016/j.sapharm.2019.04.052

ISSN: 1934-8150

Place of Publication: United States

PubMedID: 31053466

Accession Number: 31053466

Keywords: Subject Terms: *Attitudes; *Chronic non-cancer pain; *Collaboration; *Opioid use; *Pharmacist; *Physician

Abstract:BACKGROUND: Management of chronic non-cancer pain is complex, requiring clinicians to balance pain management with the risk of opioid abuse. The role of ambulatory care pharmacists in chronic pain management is well-established, but little research has explored the feasibility of building collaboration on chronic pain and opioid management between physicians and community pharmacists.OBJECTIVE: To explore physician and pharmacist perspectives on the opioid crisis and the possibility of physician and community pharmacist collaborations to manage chronic non-cancer pain in the context of the opioid crisis.METHODS: Semi-structured interviews were performed with a snowball convenience sample 15 physicians and 25 pharmacists in North Carolina between November 2016 and April 2017. Transcribed data were analyzed using applied thematic analysis, and resulting codes were organized into themes and domains which emerged from analysis.RESULTS: Both physicians and pharmacists described current care deficiencies and steps needed to mitigate opioid abuse and diversion. Physicians discussed the need for additional supports and resources for chronic pain management and regarded positively the role of the community pharmacist in chronic pain management and mitigating opioid abuse. Pharmacists identified cost as the major barrier to implementing new services, and expressed willingness to participate in new chronic pain and opioid interventions.CONCLUSION: Within the study sample, strong interest exists for collaboration between physicians and community pharmacists. This highlights a potential opportunity to expand care for patients with chronic non-cancer pain.

Institutions:
(Kang I) Eshelman School of Pharmacy, CB #7574, University of North Carolina at Chapel Hill, Chapel Hill, NC, 27599-7574, USA. Electronic address: isabell.kang@gmail.com.; (Urick B) Eshelman School of Pharmacy, CB #7574, University of North Carolina at Chapel Hill, Chapel Hill, NC, 27599-7574, USA.

Electronic address: benurick@email.unc.edu.; (Vohra R) Eshelman School of Pharmacy, CB #7574, University of North Carolina at Chapel Hill, Chapel Hill, NC, 27599-7574, USA. Electronic address: rinievohra@gmail.com.; (Ives TJ) Eshelman School of Pharmacy, CB #7574, University of North Carolina at Chapel Hill, Chapel Hill, NC, 27599-7574, USA; Division of General Medicine and Clinical Epidemiology, Department of Medicine, School of Medicine, University of North Carolina at Chapel Hill, Chapel Hill, NC, USA. Electronic address: Timothy_Ives@med.unc.edu.
(Kang I) Eshelman School of Pharmacy, CB #7574, University of North Carolina at Chapel Hill, Chapel Hill, NC, 27599-7574, USA. Electronic address: isabell.kang@gmail.com.; (Urick B) Eshelman School of Pharmacy, CB #7574, University of North Carolina at Chapel Hill, Chapel Hill, NC, 27599-7574, USA. Electronic address: benurick@email.unc.edu.; (Vohra R) Eshelman School of Pharmacy, CB #7574, University of North Carolina at Chapel Hill, Chapel Hill, NC, 27599-7574, USA. Electronic address: rinievohra@gmail.com.; (Ives TJ) Eshelman School of Pharmacy, CB #7574, University of North Carolina at Chapel Hill, Chapel Hill, NC, 27599-7574, USA; Division of General Medicine and Clinical Epidemiology, Department of Medicine, School of Medicine, University of North Carolina at Chapel Hill, Chapel Hill, NC, USA. Electronic address: Timothy_Ives@med.unc.edu.
(Kang I) Eshelman School of Pharmacy, CB #7574, University of North Carolina at Chapel Hill, Chapel Hill, NC, 27599-7574, USA. Electronic address: isabell.kang@gmail.com.; (Urick B) Eshelman School of Pharmacy, CB #7574, University of North Carolina at Chapel Hill, Chapel Hill, NC, 27599-7574, USA. Electronic address: benurick@email.unc.edu.; (Vohra R) Eshelman School of Pharmacy, CB #7574, University of North Carolina at Chapel Hill, Chapel Hill, NC, 27599-7574, USA. Electronic address: rinievohra@gmail.com.; (Ives TJ) Eshelman School of Pharmacy, CB #7574, University of North Carolina at Chapel Hill, Chapel Hill, NC, 27599-7574, USA; Division of General Medicine and Clinical Epidemiology, Department of Medicine, School of Medicine, University of North Carolina at Chapel Hill, Chapel Hill, NC, USA. Electronic address: Timothy_Ives@med.unc.edu.
Database: PubMed

44. Opioids and Cancer Pain Management in the United States: Public Policy and Legal Challenges.

Author(s): Foxwell AM; Uritsky T; Meghani SH

Source: Seminars in oncology nursing; 2019; vol. 35 (no. 3); p. 322-326

Publication Date: 2019

Publication Type(s): Journal Article; Review

DOI: http://dx.doi.org/10.1016/j.soncn.2019.04.015

ISSN: 1878-3449

Place of Publication: United States

PubMedID: 31053394

Accession Number: 31053394

Available at Seminars in Oncology Nursing - from ScienceDirect

Keywords: Subject Terms: *CDC guidelines; *cancer pain; *legislation; *opioid; *opioid regulation; *pain guidelines; *policy

Abstract:OBJECTIVE: To review current opioid guidelines, public policy, and legal challenges that can threaten optimal management of cancer pain.DATA SOURCES: National guidelines, professional web sites, journal articles, essays.CONCLUSION: Recent opioid legislation and increasing scrutiny from regulatory agencies have created multiple barriers for providers and patients to achieve pain control. These challenges include prescription limitations and practitioner fear of litigation, which can result in the under-prescribing or refusal to prescribe opioids.IMPLICATIONS FOR NURSING PRACTICE: To provide excellent care, nurses must understand current policies affecting delivery of pain care to oncology patients and serve as patient advocates in the evolving policy debates.

Institutions:
(Foxwell AM) Department of Medicine, Palliative Care Program, Hospital of the University of Pennsylvania, Philadelphia, PA. Electronic address: anessa.foxwell@uphs.upenn.edu.; (Uritsky T) Department of Pharmacy, Hospital of University of Pennsylvania, Philadelphia, PA.; (Meghani SH) Department of Biobehavioral Health Sciences, NewCourtland Center for Transitions and Health, School of Nursing, University of Pennsylvania, Philadelphia, PA.
(Foxwell AM) Department of Medicine, Palliative Care Program, Hospital of the University of Pennsylvania, Philadelphia, PA. Electronic address: anessa.foxwell@uphs.upenn.edu.; (Uritsky T) Department of Pharmacy, Hospital of University of Pennsylvania, Philadelphia, PA.; (Meghani SH) Department of Biobehavioral Health Sciences, NewCourtland Center for Transitions and Health, School of Nursing, University of Pennsylvania, Philadelphia, PA.
(Foxwell AM) Department of Medicine, Palliative Care Program, Hospital of the University of Pennsylvania, Philadelphia, PA. Electronic address: anessa.foxwell@uphs.upenn.edu.; (Uritsky T) Department of Pharmacy, Hospital of University of Pennsylvania, Philadelphia, PA.; (Meghani SH) Department of Biobehavioral Health Sciences, NewCourtland Center for Transitions and Health, School of Nursing, University of Pennsylvania, Philadelphia, PA.
Database: PubMed

45. Cancer Pain Assessment and Measurement.

Author(s): Fink RM; Gallagher E

Source: Seminars in oncology nursing; 2019; vol. 35 (no. 3); p. 229-234

Publication Date: 2019

Publication Type(s): Journal Article; Review

DOI: http://dx.doi.org/10.1016/j.soncn.2019.04.003

ISSN: 1878-3449

Place of Publication: United States

PubMedID: 31036386

Accession Number: 31036386

Available at Seminars in oncology nursing - from ScienceDirect

Keywords: Subject Terms: *Barriers; *Breakthrough pain; *Cancer; *Cognitively impaired; *Nonverbal; *Pain assessment

Abstract:OBJECTIVES: To review the incidence of cancer pain; assessment of acute, chronic, and breakthrough pain; and provide insight on assessment approaches and reliable and valid instruments for clinical and research settings.DATA SOURCES: Peer-reviewed journal articles, book chapters, Internet.CONCLUSION: Quality pain management for patients with cancer is dependent on an accurate pain assessment and ongoing reassessment, considering the whole person. Being knowledgeable about evidence-based pain assessment practices is key.IMPLICATIONS FOR NURSING PRACTICE: Concentrated efforts to address pain assessment barriers and effectively report pain assessments in diverse populations are warranted, especially in this current health care environment when pain assessment is challenging.

Institutions:
(Fink RM) College of Nursing and School of Medicine, University of Colorado Anschutz Medical Campus, Aurora, CO. Electronic address: regina.fink@ucdenver.edu.; (Gallagher E) Agios Pharmaceuticals; Georgetown University - School of Nursing & Health Studies, Excelsior, MN.
(Fink RM) College of Nursing and School of Medicine, University of Colorado Anschutz Medical Campus, Aurora, CO. Electronic address: regina.fink@ucdenver.edu.; (Gallagher E) Agios Pharmaceuticals; Georgetown University - School of Nursing & Health Studies, Excelsior, MN.
Database: PubMed

46. Telephone Follow-up Design and Practice for Advanced Cancer Pain Patients.

Author(s): Zou B; Li X; Huang X; Xiong D; Liu Y

Source: Journal of cancer education : the official journal of the American Association for Cancer Education; 2020; vol. 35 (no. 4); p. 751-759

Publication Date: 2020

Publication Type(s): Journal Article; Research Support, Non-U.S. Gov't

DOI: http://dx.doi.org/10.1007/s13187-019-01523-4

ISSN: 1543-0154

Place of Publication: England

PubMedID: 30989478

Accession Number: 30989478

Available at Journal of cancer education : the official journal of the American Association for Cancer Education - from EBSCO (MEDLINE Complete)

Available at Journal of cancer education : the official journal of the American Association for Cancer Education - from ProQuest (MEDLINE with Full Text) - NHS Version

Available at Journal of cancer education : the official journal of the American Association for Cancer Education - from ProQuest (Health Research Premium) - NHS Version

Keywords: Subject Terms: *Advanced cancer patients; *Follow-up; *Pain; *Practice

Abstract: To describe the design of a telephone follow-up protocol and to evaluate the feasibility of this protocol for advanced cancer pain patients. A series of nine telephone follow-up calls was implemented with 40 advanced cancer pain patients within 3 months after their discharge from the Department of Chemotherapy. Cancer pain information and the pain-related knowledge of the patients were collected by nurses using pain follow-up information sheets and the Patient Pain Questionnaire (PPQ); pain self-efficacy and the quality of life were reported by patients using the Chronic Pain Self-Efficacy Scale (CPSS) Chinese version and the European Organization for Research and Treatment of Cancer Quality of Life Questionnaire-Core 30 (EORTC-QLQ-C30) Chinese version. The average score assessed by advanced cancer pain patients of the need for pain care from nurses was 24.28 (SD = 4.90). Twenty-one and eight patients completed all nine telephone follow-up calls and seven self-reported questionnaires, respectively. The pain intensity of patients at the time of follow-up was mild, but there had been breakthrough pain in the previous week. All patients were satisfied with the nurses' pain follow-up practices. There was a highly positive correlation between the time of follow-up and the patients' pain-related knowledge scores ($r = 0.963$**, $p < 0.01$). Patients' pain self-efficacy scores and quality of life scores varied across different dimensions. The baseline pain self-efficacy subscales were associated with all dimensions of quality of life ($p < 0.05$ or $p < 0.01$). Telephone follow-up can be an effective method of transitional care. For advanced cancer pain patients, it is still necessary to further explore the cost effectiveness of this method, including the appropriate follow-up duration, endpoints, and outcome measures based on government requirements and policies.

Institutions:
(Zou B) The Department of Chemotherapy, Sun Yat-sen University Cancer Center, Phase I ward, 21th floor, Building 1, 651 Dongfeng East Rd, Guangzhou, 510060, Guangdong, People's Republic of China.; (Li X) The Department of Chemotherapy, Sun Yat-sen University Cancer Center, Phase I ward, 21th floor, Building 1, 651 Dongfeng East Rd, Guangzhou, 510060, Guangdong, People's Republic of China.; (Huang X) The Department of Chemotherapy, Sun Yat-sen University Cancer Center, Phase I ward, 21th floor, Building 1, 651 Dongfeng East Rd, Guangzhou, 510060, Guangdong, People's Republic of China.; (Xiong D) The Department of Chemotherapy, Sun Yat-sen University Cancer Center, Phase I ward, 21th floor, Building 1, 651 Dongfeng East Rd, Guangzhou, 510060, Guangdong, People's Republic of China.; (Liu Y) The Department of Chemotherapy, Sun Yat-sen University Cancer Center, Phase I ward, 21th floor, Building 1, 651 Dongfeng East Rd, Guangzhou, 510060, Guangdong, People's Republic of China. liuyu@sysucc.org.cn.
(Zou B) The Department of Chemotherapy, Sun Yat-sen University Cancer Center, Phase I ward, 21th floor, Building 1, 651 Dongfeng East Rd, Guangzhou, 510060, Guangdong, People's Republic of China.; (Li X) The Department of Chemotherapy,

Sun Yat-sen University Cancer Center, Phase I ward, 21th floor, Building 1, 651 Dongfeng East Rd, Guangzhou, 510060, Guangdong, People's Republic of China.; (Huang X) The Department of Chemotherapy, Sun Yat-sen University Cancer Center, Phase I ward, 21th floor, Building 1, 651 Dongfeng East Rd, Guangzhou, 510060, Guangdong, People's Republic of China.; (Xiong D) The Department of Chemotherapy, Sun Yat-sen University Cancer Center, Phase I ward, 21th floor, Building 1, 651 Dongfeng East Rd, Guangzhou, 510060, Guangdong, People's Republic of China.; (Liu Y) The Department of Chemotherapy, Sun Yat-sen University Cancer Center, Phase I ward, 21th floor, Building 1, 651 Dongfeng East Rd, Guangzhou, 510060, Guangdong, People's Republic of China. liuyu@sysucc.org.cn.
(Zou B) The Department of Chemotherapy, Sun Yat-sen University Cancer Center, Phase I ward, 21th floor, Building 1, 651 Dongfeng East Rd, Guangzhou, 510060, Guangdong, People's Republic of China.; (Li X) The Department of Chemotherapy, Sun Yat-sen University Cancer Center, Phase I ward, 21th floor, Building 1, 651 Dongfeng East Rd, Guangzhou, 510060, Guangdong, People's Republic of China.; (Huang X) The Department of Chemotherapy, Sun Yat-sen University Cancer Center, Phase I ward, 21th floor, Building 1, 651 Dongfeng East Rd, Guangzhou, 510060, Guangdong, People's Republic of China.; (Xiong D) The Department of Chemotherapy, Sun Yat-sen University Cancer Center, Phase I ward, 21th floor, Building 1, 651 Dongfeng East Rd, Guangzhou, 510060, Guangdong, People's Republic of China.; (Liu Y) The Department of Chemotherapy, Sun Yat-sen University Cancer Center, Phase I ward, 21th floor, Building 1, 651 Dongfeng East Rd, Guangzhou, 510060, Guangdong, People's Republic of China. liuyu@sysucc.org.cn.
(Zou B) The Department of Chemotherapy, Sun Yat-sen University Cancer Center, Phase I ward, 21th floor, Building 1, 651 Dongfeng East Rd, Guangzhou, 510060, Guangdong, People's Republic of China.; (Li X) The Department of Chemotherapy, Sun Yat-sen University Cancer Center, Phase I ward, 21th floor, Building 1, 651 Dongfeng East Rd, Guangzhou, 510060, Guangdong, People's Republic of China.; (Huang X) The Department of Chemotherapy, Sun Yat-sen University Cancer Center, Phase I ward, 21th floor, Building 1, 651 Dongfeng East Rd, Guangzhou, 510060, Guangdong, People's Republic of China.; (Xiong D) The Department of Chemotherapy, Sun Yat-sen University Cancer Center, Phase I ward, 21th floor, Building 1, 651 Dongfeng East Rd, Guangzhou, 510060, Guangdong, People's Republic of China.; (Liu Y) The Department of Chemotherapy, Sun Yat-sen University Cancer Center, Phase I ward, 21th floor, Building 1, 651 Dongfeng East Rd, Guangzhou, 510060, Guangdong, People's Republic of China. liuyu@sysucc.org.cn.
(Zou B) The Department of Chemotherapy, Sun Yat-sen University Cancer Center, Phase I ward, 21th floor, Building 1, 651 Dongfeng East Rd, Guangzhou, 510060, Guangdong, People's Republic of China.; (Li X) The Department of Chemotherapy, Sun Yat-sen University Cancer Center, Phase I ward, 21th floor, Building 1, 651 Dongfeng East Rd, Guangzhou, 510060, Guangdong, People's Republic of China.; (Huang X) The Department of Chemotherapy, Sun Yat-sen University Cancer Center, Phase I ward, 21th floor, Building 1, 651 Dongfeng East Rd, Guangzhou, 510060, Guangdong, People's Republic of China.; (Xiong D) The Department of Chemotherapy, Sun Yat-sen University Cancer Center, Phase I ward, 21th floor, Building 1, 651 Dongfeng East Rd, Guangzhou, 510060, Guangdong, People's Republic of China.; (Liu Y) The Department of Chemotherapy, Sun Yat-sen

University Cancer Center, Phase I ward, 21th floor, Building 1, 651 Dongfeng East Rd, Guangzhou, 510060, Guangdong, People's Republic of China. liuyu@sysucc.org.cn.
Database: PubMed

47. Care management for the hospitalized child with chronic cancer pain: intervening conditions.

Author(s): Silva TPD; Silva LJD; Rodrigues BMRD; Silva ÍR; Chistoffel MM; Leite JL

Source: Revista brasileira de enfermagem; Feb 2019; vol. 72 (no. suppl 1); p. 181-188

Publication Date: Feb 2019

Publication Type(s): Journal Article

DOI: http://dx.doi.org/10.1590/0034-7167-2017-0514

ISSN: 1984-0446

Place of Publication: Brazil

PubMedID: 30942361

Accession Number: 30942361

Available at Revista brasileira de enfermagem - from EBSCO (CINAHL Complete)

Available at Revista brasileira de enfermagem - from ProQuest (MEDLINE with Full Text) - NHS Version

Available at Revista brasileira de enfermagem - from ProQuest (Health Research Premium) - NHS Version

Available at Revista brasileira de enfermagem - from Unpaywall

Abstract:OBJECTIVE: To understand the intervening conditions of care management for the hospitalized child with chronic cancer pain.METHOD: Qualitative research, anchored in the methodological and theoretical frameworks, respectively, Grounded Theory and Complex Thinking. The semi-structured interview and non-participant observation were used to collect the data. Twenty-one health professionals, organized in three sample groups: nurses; nursing technicians; and professionals of the multiprofessional health staff.RESULTS: They emerged as intervening conditions of care management: human resources and materials, teamwork, absenteeism, professional relocation, professional qualification, family, playful, dialogue, empathy and caring relationship.CONCLUSION: It was understood as limiting conditions for care management: deficits of human resources and materials, absenteeism, ineffective teamwork, professional relocation, and insufficient professional qualification. On the other hand, they were presented as facilitating conditions: adequate professional knowledge, effective teamwork, dialogue, empathy, playful and affective relationship with the child.

Institutions:
(Silva TPD) Universidade Federal do Rio de Janeiro, Escola de Enfermagem Anna Nery. Rio de Janeiro, Rio de Janeiro, Brazil.; (Silva LJD) Universidade Federal do Estado do Rio de Janeiro, Escola de Enfermagem Alfredo Pinto. Rio de Janeiro, Rio de Janeiro, Brazil.; (Rodrigues BMRD) Universidade do Estado do Rio de Janeiro. Rio de Janeiro, Rio de Janeiro, Brazil.; (Silva ÍR) Universidade Federal do Rio de Janeiro, Escola de Enfermagem Anna Nery. Rio de Janeiro, Rio de Janeiro, Brazil.;

(Chistoffel MM) Universidade Federal do Rio de Janeiro, Escola de Enfermagem Anna Nery. Rio de Janeiro, Rio de Janeiro, Brazil.; (Leite JL) Universidade Federal do Estado do Rio de Janeiro, Escola de Enfermagem Alfredo Pinto. Rio de Janeiro, Rio de Janeiro, Brazil.
(Silva TPD) Universidade Federal do Rio de Janeiro, Escola de Enfermagem Anna Nery. Rio de Janeiro, Rio de Janeiro, Brazil.; (Silva LJD) Universidade Federal do Estado do Rio de Janeiro, Escola de Enfermagem Alfredo Pinto. Rio de Janeiro, Rio de Janeiro, Brazil.; (Rodrigues BMRD) Universidade do Estado do Rio de Janeiro. Rio de Janeiro, Rio de Janeiro, Brazil.; (Silva ÍR) Universidade Federal do Rio de Janeiro, Escola de Enfermagem Anna Nery. Rio de Janeiro, Rio de Janeiro, Brazil.;
(Chistoffel MM) Universidade Federal do Rio de Janeiro, Escola de Enfermagem Anna Nery. Rio de Janeiro, Rio de Janeiro, Brazil.; (Leite JL) Universidade Federal do Estado do Rio de Janeiro, Escola de Enfermagem Alfredo Pinto. Rio de Janeiro, Rio de Janeiro, Brazil.
(Silva TPD) Universidade Federal do Rio de Janeiro, Escola de Enfermagem Anna Nery. Rio de Janeiro, Rio de Janeiro, Brazil.; (Silva LJD) Universidade Federal do Estado do Rio de Janeiro, Escola de Enfermagem Alfredo Pinto. Rio de Janeiro, Rio de Janeiro, Brazil.; (Rodrigues BMRD) Universidade do Estado do Rio de Janeiro. Rio de Janeiro, Rio de Janeiro, Brazil.; (Silva ÍR) Universidade Federal do Rio de Janeiro, Escola de Enfermagem Anna Nery. Rio de Janeiro, Rio de Janeiro, Brazil.;
(Chistoffel MM) Universidade Federal do Rio de Janeiro, Escola de Enfermagem Anna Nery. Rio de Janeiro, Rio de Janeiro, Brazil.; (Leite JL) Universidade Federal do Estado do Rio de Janeiro, Escola de Enfermagem Alfredo Pinto. Rio de Janeiro, Rio de Janeiro, Brazil.
(Silva TPD) Universidade Federal do Rio de Janeiro, Escola de Enfermagem Anna Nery. Rio de Janeiro, Rio de Janeiro, Brazil.; (Silva LJD) Universidade Federal do Estado do Rio de Janeiro, Escola de Enfermagem Alfredo Pinto. Rio de Janeiro, Rio de Janeiro, Brazil.; (Rodrigues BMRD) Universidade do Estado do Rio de Janeiro. Rio de Janeiro, Rio de Janeiro, Brazil.; (Silva ÍR) Universidade Federal do Rio de Janeiro, Escola de Enfermagem Anna Nery. Rio de Janeiro, Rio de Janeiro, Brazil.;
(Chistoffel MM) Universidade Federal do Rio de Janeiro, Escola de Enfermagem Anna Nery. Rio de Janeiro, Rio de Janeiro, Brazil.; (Leite JL) Universidade Federal do Estado do Rio de Janeiro, Escola de Enfermagem Alfredo Pinto. Rio de Janeiro, Rio de Janeiro, Brazil.
(Silva TPD) Universidade Federal do Rio de Janeiro, Escola de Enfermagem Anna Nery. Rio de Janeiro, Rio de Janeiro, Brazil.; (Silva LJD) Universidade Federal do Estado do Rio de Janeiro, Escola de Enfermagem Alfredo Pinto. Rio de Janeiro, Rio de Janeiro, Brazil.; (Rodrigues BMRD) Universidade do Estado do Rio de Janeiro. Rio de Janeiro, Rio de Janeiro, Brazil.; (Silva ÍR) Universidade Federal do Rio de Janeiro, Escola de Enfermagem Anna Nery. Rio de Janeiro, Rio de Janeiro, Brazil.;
(Chistoffel MM) Universidade Federal do Rio de Janeiro, Escola de Enfermagem Anna Nery. Rio de Janeiro, Rio de Janeiro, Brazil.; (Leite JL) Universidade Federal do Estado do Rio de Janeiro, Escola de Enfermagem Alfredo Pinto. Rio de Janeiro, Rio de Janeiro, Brazil.
(Silva TPD) Universidade Federal do Rio de Janeiro, Escola de Enfermagem Anna Nery. Rio de Janeiro, Rio de Janeiro, Brazil.; (Silva LJD) Universidade Federal do Estado do Rio de Janeiro, Escola de Enfermagem Alfredo Pinto. Rio de Janeiro, Rio de Janeiro, Brazil.; (Rodrigues BMRD) Universidade do Estado do Rio de Janeiro. Rio de Janeiro, Rio de Janeiro, Brazil.; (Silva ÍR) Universidade Federal do Rio de Janeiro, Escola de Enfermagem Anna Nery. Rio de Janeiro, Rio de Janeiro, Brazil.;

(Chistoffel MM) Universidade Federal do Rio de Janeiro, Escola de Enfermagem Anna Nery. Rio de Janeiro, Rio de Janeiro, Brazil.; (Leite JL) Universidade Federal do Estado do Rio de Janeiro, Escola de Enfermagem Alfredo Pinto. Rio de Janeiro, Rio de Janeiro, Brazil.
Database: PubMed

48. Cancer Pain and Quality of Life.

Author(s): Rodriguez C; Ji M; Wang HL; Padhya T; McMillan SC

Source: Journal of hospice and palliative nursing : JHPN : the official journal of the Hospice and Palliative Nurses Association; 2019; vol. 21 (no. 2); p. 116-123

Publication Date: 2019

Publication Type(s): Journal Article; Research Support, Non-U.S. Gov't

DOI: http://dx.doi.org/10.1097/NJH.0000000000000507

ISSN: 1539-0705

Place of Publication: United States

PubMedID: 30829932

Accession Number: 30829932

Abstract: Cancer pain is an unrelenting symptom with the potential to alter the quality of life of patients. To adequately manage pain, nurses caring for cancer patients need to fully understand each patient's pain experience. The purpose of this study was to identify the intensity, distress, frequency, or constancy of pain in patients treated for cancer or cancer symptoms and to better understand patient barriers to pain management. This cross-sectional study included patients (N = 105) treated for cancer or cancer symptoms at 2 outpatient medical centers. Assessments included the Pain Barriers Scale, the Cancer Symptom Scale, and the Multidimensional QOL Scale-Cancer. Descriptive statistics and Spearman correlations were used to analyze the data. Sixty-nine percent of patients reported present pain of moderate to severe intensity that caused distress, was frequent/constant, or interfered with their lives. Patients with the greatest pain distress reported the greatest intensity of pain (r = 0.77) and the greatest interference (r = 0.78) with daily lives. Cancer pain was associated with significant distress and interference with life activities and occurred frequently or constantly for many study patients.

Institutions:
(Rodriguez C) Carmen Rodriguez, PhD, ANP-BC, AOCN, is associate professor, University of South Florida-College of Nursing, Tampa. Ming Ji, PhD, is professor, University of South Florida-College of Nursing, Tampa. Hsiao-Lan Wang, PhD, RN, CMSRN, ACSM EP-C, is associate professor, University of South Florida-College of Nursing, Tampa. Tapan Padhya, MD, is professor, College of Medicine, University of South Florida-Health, H. Lee Moffitt Cancer Center, Tampa. Susan C. McMillan, PhD, ARNP, FAAN, is distinguished professor, University of South Florida-College of Nursing, Tampa.
Database: PubMed

49. Addressing Educational Needs in Managing Complex Pain in Cancer Populations: Evaluation of APAM: An Online Educational Intervention for Nurses.

Author(s): Leung YW; Wong J; Kiteley C; Ellis J; Esplen MJ

Source: The American journal of hospice & palliative care; Jul 2019; vol. 36 (no. 7); p. 587-597

Publication Date: Jul 2019

Publication Type(s): Journal Article

DOI: http://dx.doi.org/10.1177/1049909119832819

ISSN: 1938-2715

Place of Publication: United States

PubMedID: 30813737

Accession Number: 30813737

Keywords: Subject Terms: and nurses; cancer; continued education and training; online learning; pain assessment; pain management

Abstract:CONTEXT: Cancer-related pain is associated with significant suffering and is one of the most challenging symptoms to manage. Studies indicate that front-line clinicians often lack the knowledge on best practices in cancer pain management.OBJECTIVES: The current project, a quality improvement (QI) initiative, evaluated the outcome of an online educational intervention for nurses on complex cancer pain management.METHODS: An online 7-module educational intervention, Advanced Pain Assessment and Management, was offered from 2012 to 2017. Pre-post course evaluations included self-reported knowledge and confidence across cancer pain management domains. In-course competency assessments included knowledge examination, online discussion forum participation, opioid dosage calculation assignment, and small-group-based case study. A mixed-model statistical analysis was used to assess pre-post course change in pain management confidence level.RESULTS: In all, 306 nurses from 89 hospitals in Ontario, Canada, were enrolled in the course; 81.4% returned the precourse survey and 71.9% successfully completed the course. The average confidence level on pain management was low at baseline (57.5%) but improved significantly post-course. In-course competency assessments ranged from 81% to 89%. Mixed-model results showed post-course improvements in confidence levels, independent of sociodemographic background, clinical role, and professional educational level. Nurses with longer years of practice and more cancer cases reported greater confidence.CONCLUSION: A facilitator-led online educational intervention focusing on complex cancer pain management can significantly improve nurses' knowledge, confidence, and skills. Low baseline knowledge among nurses highlights the pressing need for health-care organizations to implement cancer pain management training as an integral part of health-care QI initiative.

Institutions:
(Leung YW) 1 de Souza Institute, University Health Network, Toronto, Ontario, Canada.; (Wong J) 1 de Souza Institute, University Health Network, Toronto, Ontario, Canada.; (Kiteley C) 1 de Souza Institute, University Health Network, Toronto, Ontario, Canada.; (Ellis J) 1 de Souza Institute, University Health Network, Toronto, Ontario, Canada.; (Esplen MJ) 1 de Souza Institute, University Health Network, Toronto, Ontario, Canada.

(Leung YW) 1 de Souza Institute, University Health Network, Toronto, Ontario, Canada.; (Wong J) 1 de Souza Institute, University Health Network, Toronto, Ontario, Canada.; (Kiteley C) 1 de Souza Institute, University Health Network, Toronto, Ontario, Canada.; (Ellis J) 1 de Souza Institute, University Health Network, Toronto, Ontario, Canada.; (Esplen MJ) 1 de Souza Institute, University Health Network, Toronto, Ontario, Canada.
(Leung YW) 1 de Souza Institute, University Health Network, Toronto, Ontario, Canada.; (Wong J) 1 de Souza Institute, University Health Network, Toronto, Ontario, Canada.; (Kiteley C) 1 de Souza Institute, University Health Network, Toronto, Ontario, Canada.; (Ellis J) 1 de Souza Institute, University Health Network, Toronto, Ontario, Canada.; (Esplen MJ) 1 de Souza Institute, University Health Network, Toronto, Ontario, Canada.
(Leung YW) 1 de Souza Institute, University Health Network, Toronto, Ontario, Canada.; (Wong J) 1 de Souza Institute, University Health Network, Toronto, Ontario, Canada.; (Kiteley C) 1 de Souza Institute, University Health Network, Toronto, Ontario, Canada.; (Ellis J) 1 de Souza Institute, University Health Network, Toronto, Ontario, Canada.; (Esplen MJ) 1 de Souza Institute, University Health Network, Toronto, Ontario, Canada.
(Leung YW) 1 de Souza Institute, University Health Network, Toronto, Ontario, Canada.; (Wong J) 1 de Souza Institute, University Health Network, Toronto, Ontario, Canada.; (Kiteley C) 1 de Souza Institute, University Health Network, Toronto, Ontario, Canada.; (Ellis J) 1 de Souza Institute, University Health Network, Toronto, Ontario, Canada.; (Esplen MJ) 1 de Souza Institute, University Health Network, Toronto, Ontario, Canada.
Database: PubMed

50. Therapeutic alternatives for supporting GPs to deprescribe opioids: a cross-sectional survey.

Author(s): White RA; Hayes C; Boyes AW; Chiu S; Paul CL

Source: BJGP open; Dec 2018; vol. 2 (no. 4); p. bjgpopen18X101609

Publication Date: Dec 2018

Publication Type(s): Journal Article

DOI: http://dx.doi.org/10.3399/bjgpopen18X101609

ISSN: 2398-3795

Place of Publication: England

PubMedID: 30723795

Accession Number: 30723795

Available at BJGP open - from Unpaywall

Abstract: Background: GPs are central to opioid strategy in chronic non-cancer pain (CNCP). Lack of treatment alternatives and providers are common reasons cited for not deprescribing opioids. There are limited data about availability of multidisciplinary healthcare providers (MHCPs), such as psychologists, physiotherapists, or dietitians, who can provide broader treatments.Aim: To explore availability of MHCPs, and the association with GP opioid deprescribing and transition to therapeutic alternatives for CNCP.Design & setting: Cross-sectional survey of all practising GPs (N = 1480) in one mixed urban and regional Australian primary health network.Method: A self-

report mailed questionnaire assessed the availability of MHCPs and management of their most recent patient on long-term opioids for CNCP.Results: Six hundred and eighty-one (46%) valid responses were received. Most GPs (71%) had access to a pain specialist and MHCPs within 50 km. GPs' previous referral for specialist support was significantly associated with access to a greater number of MHCPs (P = 0.001). Employment of a nurse increased the rate ratio of available MHCPs by 12.5% (incidence rate ratio [IRR] 1.125, 95% confidence interval [CI] = 1.001 to 1.264). Only one-third (32%) of GPs reported willingness to deprescribe and shift to broader CNCP treatments. Availability of MHCPs was not significantly associated with deprescribing decisions.Conclusion: Lack of geographical access to known MHCPs does not appear to be a major barrier to opioid deprescribing and shifting toward non-pharmacological treatments for CNCP. Considerable opportunity remains to encourage GPs' decision to deprescribe, with employment of a practice nurse appearing to play a role.

Institutions:

(White RA) Pain Physiotherapist, School of Medicine and Public Health, University of Newcastle, Newcastle, Australia.; (Hayes C) Director, Hunter Integrated Pain Service, Hunter New England Health, Newcastle, Australia.; (Boyes AW) NHMRC Early Career Fellow, Faculty of Health & Medicine, School of Medicine and Public Health, University of Newcastle, Newcastle, Australia.; (Chiu S) Statistician, Hunter Medical Research Institute, Newcastle, Australia.; (Paul CL) Associate Dean, School of Medicine and Public Health, University of Newcastle, Newcastle, Australia.
(White RA) Pain Physiotherapist, School of Medicine and Public Health, University of Newcastle, Newcastle, Australia.; (Hayes C) Director, Hunter Integrated Pain Service, Hunter New England Health, Newcastle, Australia.; (Boyes AW) NHMRC Early Career Fellow, Faculty of Health & Medicine, School of Medicine and Public Health, University of Newcastle, Newcastle, Australia.; (Chiu S) Statistician, Hunter Medical Research Institute, Newcastle, Australia.; (Paul CL) Associate Dean, School of Medicine and Public Health, University of Newcastle, Newcastle, Australia.
(White RA) Pain Physiotherapist, School of Medicine and Public Health, University of Newcastle, Newcastle, Australia.; (Hayes C) Director, Hunter Integrated Pain Service, Hunter New England Health, Newcastle, Australia.; (Boyes AW) NHMRC Early Career Fellow, Faculty of Health & Medicine, School of Medicine and Public Health, University of Newcastle, Newcastle, Australia.; (Chiu S) Statistician, Hunter Medical Research Institute, Newcastle, Australia.; (Paul CL) Associate Dean, School of Medicine and Public Health, University of Newcastle, Newcastle, Australia.
(White RA) Pain Physiotherapist, School of Medicine and Public Health, University of Newcastle, Newcastle, Australia.; (Hayes C) Director, Hunter Integrated Pain Service, Hunter New England Health, Newcastle, Australia.; (Boyes AW) NHMRC Early Career Fellow, Faculty of Health & Medicine, School of Medicine and Public Health, University of Newcastle, Newcastle, Australia.; (Chiu S) Statistician, Hunter Medical Research Institute, Newcastle, Australia.; (Paul CL) Associate Dean, School of Medicine and Public Health, University of Newcastle, Newcastle, Australia.
(White RA) Pain Physiotherapist, School of Medicine and Public Health, University of Newcastle, Newcastle, Australia.; (Hayes C) Director, Hunter Integrated Pain Service, Hunter New England Health, Newcastle, Australia.; (Boyes AW) NHMRC Early Career Fellow, Faculty of Health & Medicine, School of Medicine and Public Health, University of Newcastle, Newcastle, Australia.; (Chiu S) Statistician, Hunter Medical Research Institute, Newcastle, Australia.; (Paul CL) Associate Dean, School of Medicine and Public Health, University of Newcastle, Newcastle, Australia.

Database: PubMed

51. Family caregiver beliefs and barriers to effective pain management of cancer patients in home care settings.

Author(s): Konstantis A; Exiara T

Source: Journal of B.U.ON. : official journal of the Balkan Union of Oncology; 2018; vol. 23 (no. 7); p. 144-152

Publication Date: 2018

Publication Type(s): Journal Article

ISSN: 1107-0625

Place of Publication: Cyprus

PubMedID: 30722124

Accession Number: 30722124

Abstract:PURPOSE: Pain is one of the most common symptoms in cancer patients, and its management is a significant goal in supportive care. Many barriers interfere with its effective control. Nowadays, with a shift in care from the hospital to the home, there is an increasing tendency to involve family caregivers in pain management. Their beliefs may act as barriers to effective pain management in these homecare settings. This study aimed to validate and explore these beliefs using Barriers Questionnaire II (BQ II).METHODS: A cross-sectional survey of 202 individuals from a cohort of family caregivers in Greece.RESULTS: The reliability index Cronbach, a value for the translated version of the BQ II, calculated >0.9. Most participants reported worries about the side effects of analgesics. They often assumed these effects were irreversible, and equally, there were concerns about addiction to these drugs. They agreed on the effectiveness of analgesics in treating cancer pain but disagreed that reporting pain is a distracting factor in active cancer treatment.CONCLUSIONS: This is the first time BQ II has been used in this population. The results are consistent with the international academic studies in this area, but more research is needed. BQ II was found to be a valid and reliable scale for defining caregiver attitudes and barriers to effective pain management in homecare settings. Health professional training, interventions targeted to caregivers and trained home care teams may improve the quality of cancer care in these settings.

Institutions:
(Konstantis A) University College London Hospitals, UK.
Database: PubMed

52. [An assessment of physicians attitudes toward opioid usage and opiophobia: Results of a survey from a training and research hospital].

Author(s): Baldemir R; Akçaboy EY; Noyan Ö; Akçaboy ZN; Baydar M; Çelik Ş

Source: Agri : Agri (Algoloji) Dernegi'nin Yayin organidir = The journal of the Turkish Society of Algology; Jan 2019; vol. 31 (no. 1); p. 23-31

Publication Date: Jan 2019

Publication Type(s): Journal Article

DOI: http://dx.doi.org/10.5505/agri.2018.03411

ISSN: 1300-0012

Place of Publication: Turkey

PubMedID: 30633310

Accession Number: 30633310

Abstract:OBJECTIVES: When researches examined, cancer pain and other cronic pain, serious mismanagement and undermedication in treating chronic pain especially using opioids continuing problem. This study was designed to examine the barriers to adequate opioid usage, especially as they could be associated with experience and medical discipline.METHODS: The survey conducted among phycians working in Ankara Numune education and research hospital on July of 2015. A 29 item survey was used to measure physicians attitudes and knowledge about using opioids.RESULTS: We have included 156 doctors in our study.We found that %82.7 of doctors have not been educated in the pain subject.%48.8 of doctors see themselves capable of prescribing opioid medication and %67.9 of doctors are feeling uneasy when prescribing opioids also a lot of doctors are not willing to give prescriptions of opioids.When analyzing our data we have found that opioids related prejudices are not related with title or experience.İt is interesting that the doctors who were anxious when prescribing opioids to their patients they want for themselves when in need opioid precriptions and again when in need they want increasing doses of opioid.CONCLUSION: Resembling the studies in the literature in our study, we've found that doctors have poor knowledge about opioids and opiophobia it's frequent among the doctors.Poor knowledge of opioids and overcoming the opiophobia and medicating with opioids when medical status of patients emposes made us think that in service training should be started immeadiately.

Institutions:
(Baldemir R) Department of Anesthesiology and Reanimation, Ankara Atatürk Chest Diseases and Chest Surgery Training and Research Hospital, Arkara, Turkey. baldemir23@yahoo.com.
Database: PubMed

53. Attitudinal Barriers to Pain Management and Associated Factors Among Cancer Patients in Mainland China: Implications for Cancer Education.

Author(s): Zeng D; Li K; Lin X; Mizuno M

Source: Journal of cancer education : the official journal of the American Association for Cancer Education; 2020; vol. 35 (no. 2); p. 284-291

Publication Date: 2020

Publication Type(s): Journal Article

DOI: http://dx.doi.org/10.1007/s13187-018-1463-0

ISSN: 1543-0154

Place of Publication: England

PubMedID: 30607804

Accession Number: 30607804

Available at Journal of cancer education : the official journal of the American Association for Cancer Education - from EBSCO (MEDLINE Complete)

Available at Journal of cancer education : the official journal of the American Association for Cancer Education - from ProQuest (MEDLINE with Full Text) - NHS Version

Available at Journal of cancer education : the official journal of the American Association for Cancer Education - from ProQuest (Health Research Premium) - NHS Version

Keywords: Subject Terms: *Attitude; *Barriers questionnaire; *Cancer; *Mainland China; *Pain management

Abstract: Attitudinal barriers to pain management are supposed to contribute to the uncontrolled cancer pain in mainland China. The purpose of this study was (1) to investigate the attitudinal barriers to pain management among cancer patients in mainland China, (2) to examine relationships between the attitudinal barriers and patients' pain management conditions in the light of medication adherence and adequacy of analgesic use, and (3) to identify factors associated with the attitudinal barriers. A cross-sectional questionnaire survey, including the Barriers Questionnaire-Chinese (BQ-C) and two scales that measure the medication adherence and the adequacy of analgesic use, was carried out among patients with a variety of cancers. The questionnaires were completed by 246 cancer patients (response rate 94.6%); their mean age was 51.5 years (SD = 11.7). Almost all the patients had various attitudinal barriers to pain management. The mean scores for the total scale and several subscales of the BQ-C were significantly different by the patients' characteristics, the medication adherence, and the adequacy of analgesic use. The associations with these variables for a given subscale, e.g., the subscale regarding concerns about side effects, were different from those for other subscales, e.g., the subscale regarding fatalism that cancer pain is uncontrollable. The findings suggest that a nurse-led educational program in the light of patients' characteristics is required for overcoming the attitudinal barriers to pain management among cancer patients in mainland China.

Institutions:
(Zeng D) Graduate School of Comprehensive Human Sciences, University of Tsukuba, Tsukuba, Ibaraki, 305-8577, Japan.; (Li K) School of Nursing, Sun Yat-sen University, Guangzhou, 510080, People's Republic of China. likun22@mail.sysu.edu.cn.; (Lin X) School of Nursing, Sun Yat-sen University, Guangzhou, 510080, People's Republic of China.; (Mizuno M) Faculty of Medicine, University of Tsukuba, Tsukuba, Ibaraki, 305-8577, Japan.
(Zeng D) Graduate School of Comprehensive Human Sciences, University of Tsukuba, Tsukuba, Ibaraki, 305-8577, Japan.; (Li K) School of Nursing, Sun Yat-sen University, Guangzhou, 510080, People's Republic of China. likun22@mail.sysu.edu.cn.; (Lin X) School of Nursing, Sun Yat-sen University, Guangzhou, 510080, People's Republic of China.; (Mizuno M) Faculty of Medicine, University of Tsukuba, Tsukuba, Ibaraki, 305-8577, Japan.
(Zeng D) Graduate School of Comprehensive Human Sciences, University of Tsukuba, Tsukuba, Ibaraki, 305-8577, Japan.; (Li K) School of Nursing, Sun Yat-sen University, Guangzhou, 510080, People's Republic of China. likun22@mail.sysu.edu.cn.; (Lin X) School of Nursing, Sun Yat-sen University, Guangzhou, 510080, People's Republic of China.; (Mizuno M) Faculty of Medicine, University of Tsukuba, Tsukuba, Ibaraki, 305-8577, Japan.

(Zeng D) Graduate School of Comprehensive Human Sciences, University of Tsukuba, Tsukuba, Ibaraki, 305-8577, Japan.; (Li K) School of Nursing, Sun Yat-sen University, Guangzhou, 510080, People's Republic of China. likun22@mail.sysu.edu.cn.; (Lin X) School of Nursing, Sun Yat-sen University, Guangzhou, 510080, People's Republic of China.; (Mizuno M) Faculty of Medicine, University of Tsukuba, Tsukuba, Ibaraki, 305-8577, Japan.
Database: PubMed

54. Factors That Hinder and Facilitate Cancer Patients' Knowledge About Pain Management-A Qualitative Study.

Author(s): Ekstedt M; Rustøen T

Source: Journal of pain and symptom management; 2019; vol. 57 (no. 4); p. 753-760.e1

Publication Date: 2019

Publication Type(s): Journal Article; Research Support, Non-U.S. Gov't

DOI: http://dx.doi.org/10.1016/j.jpainsymman.2018.12.334

ISSN: 1873-6513

Place of Publication: United States

PubMedID: 30593908

Accession Number: 30593908

Available at Journal of pain and symptom management - from ScienceDirect

Available at Journal of pain and symptom management - from Unpaywall

Keywords: Subject Terms: *Cancer pain; *PRO-SELF Pain Control Program; *barriers; *facilitators; *interview

Abstract:CONTEXT: Pain management education may improve pain control for some patients, whereas individual differences exist.OBJECTIVES: To evaluate possible critical components, facilitators, and hindrances for improved knowledge about pain management, in not hospitalized adult oncology patients with pain from bone metastasis participating in a pain management intervention.METHODS: This substudy is a qualitative evaluation of the PRO-SELF Pain Control Program, tested in a randomized controlled trial. During six weeks, 87 participants in the intervention group received tailored coaching encounters by a trained oncology nurse. Three encounters for each patient were audio recorded. The encounter between patient's with the largest (n = 12) and lowest (n = 8) change in knowledge about pain management from before to after the intervention was transcribed verbatim and analyzed with qualitative content analysis.RESULTS: The critical components of the intervention were repetition of information, struggling with resistance, use of peer experiences, and keeping track of variations. Facilitators of improvement were patients' trust and preparedness to try new procedures, the patient's self-awareness and body awareness, and taking active role in own care. Difficulties in processing complex information, culturally conditioned behaviors, fear, and lack of knowledge were the most important barriers to the success of the intervention.CONCLUSION: Education in pain management in cancer patients requires repeated information, allowing time for overcoming resistance related to dysfunctional beliefs and fear. To

facilitate the patient's involvement in their pain management, tailored and person-centered education is needed.

Institutions:
(Ekstedt M) Faculty of Health and Life Sciences, Linnaeus University, Kalmar, Sweden; Medical Management Centre, Department of Learning, Informatics, Management and Ethics, Karolinska Institutet, Stockholm, Sweden.; (Rustøen T) Department of Research and Development, Division of Emergencies and Critical Care, Oslo University Hospital, Oslo, Norway; Department of Nursing Science, Institute of Health and Society, Faculty of Medicine, University of Oslo, Oslo, Norway. Electronic address: tone.rustoen@medisin.uio.no.
(Ekstedt M) Faculty of Health and Life Sciences, Linnaeus University, Kalmar, Sweden; Medical Management Centre, Department of Learning, Informatics, Management and Ethics, Karolinska Institutet, Stockholm, Sweden.; (Rustøen T) Department of Research and Development, Division of Emergencies and Critical Care, Oslo University Hospital, Oslo, Norway; Department of Nursing Science, Institute of Health and Society, Faculty of Medicine, University of Oslo, Oslo, Norway. Electronic address: tone.rustoen@medisin.uio.no.
Database: PubMed

55. Testing a pain self-management intervention by exploring reduction of analgesics' side effects in cancer outpatients and the involvement of family caregivers: a study protocol (PEINCA-FAM).

Author(s): Valenta S; Spirig R; Miaskowski C; Zaugg K; Spichiger E

Source: BMC nursing; 2018; vol. 17 ; p. 54

Publication Date: 2018

Publication Type(s): Journal Article

DOI: http://dx.doi.org/10.1186/s12912-018-0323-x

ISSN: 1472-6955

Place of Publication: England

PubMedID: 30559603

Accession Number: 30559603

Available at BMC Nursing - from BioMed Central

Available at BMC Nursing - from Europe PubMed Central - Open Access

Available at BMC Nursing - from DOAJ - Directory of Open Access Journals

Available at BMC Nursing - from EBSCO (CINAHL Complete)

Available at BMC Nursing - from ProQuest (Health Research Premium) - NHS Version

Available at BMC Nursing - from Unpaywall

Keywords: Subject Terms: Adverse effects; Caregivers; Health behaviour; Health knowledge, attitudes, practice; Neoplasms; Pain management; Patient education; Randomized controlled trial; Self-care

Abstract: Background: Pain is one of cancer patients' most frequent and distressing symptoms; however, analgesics' side effects often increase symptom burden. Further, with the home rapidly becoming the primary cancer care setting, family

caregivers (FCs) commonly play central roles in patients' pain self-management, but with little or no preparation. One US-tested intervention, the PRO-SELF© Plus Pain Control Program (PCP), designed to support cancer outpatients and their FCs in pain self-management, is currently being tested in the Swiss multi-centre PEINCA study. The current PEINCA-FAM study is a sub-study of PEINCA. The aims of PEINCA-FAM are: a) to test the efficacy of the adapted German PRO-SELF © Plus PCP to reduce side effects of analgesics; b) to enhance patients'/FCs' knowledge regarding cancer pain; and c) to explore FCs' involvement in patients' pain self-management.Methods: This mixed methods project combines a multi-centre randomized controlled clinical trial with qualitative data collection techniques and includes 210 patients recruited from three oncology outpatient clinics. FCs involved in patients' pain self-management are also invited to participate. After baseline evaluation, eligible participants are randomized to a 6-week intervention group and a control group. Both groups complete a daily pain and symptom diary. Intervention group patients/FCs receive the weekly psychoeducational PRO-SELF© Plus PCP interventions; control group patients receive usual care. After completing the six-week study procedures, a subsample of 7-10 patients/FCs per group and hospital (N = 42-60) will be interviewed regarding their pain management experiences. Data collection will take place from April 2016 until December 2018. An intent-to-treat analysis and generalized linear mixed models will be applied. Qualitative data will be analysed by using interpretive description. Quantitative and qualitative results will be combined within a mixed method matrix.Discussion: In clinical practice, specially trained oncology nurses in outpatient clinics could apply the intervention to reduce side effects and to enhance patients'/FCs' self-efficacy and pain management knowledge.Trial registration: The PEINCA study is registered in the Clinical Trials.gov site (code: NCT02713919, 08 March 2016).

Institutions:

(Valenta S) 1Nursing Science, Department Public Health, University of Basel, Bernoullistrasse 28, CH-4056 Basel, Switzerland.; (Spirig R) 1Nursing Science, Department Public Health, University of Basel, Bernoullistrasse 28, CH-4056 Basel, Switzerland.; (Miaskowski C) 4School of Nursing, University of California San Francisco (UCSF), San Francisco, USA.; (Zaugg K) 5Department of Radiation Oncology, Stadtspital Triemli, Zurich, Switzerland.; (Spichiger E) 1Nursing Science, Department Public Health, University of Basel, Bernoullistrasse 28, CH-4056 Basel, Switzerland.

(Valenta S) 1Nursing Science, Department Public Health, University of Basel, Bernoullistrasse 28, CH-4056 Basel, Switzerland.; (Spirig R) 1Nursing Science, Department Public Health, University of Basel, Bernoullistrasse 28, CH-4056 Basel, Switzerland.; (Miaskowski C) 4School of Nursing, University of California San Francisco (UCSF), San Francisco, USA.; (Zaugg K) 5Department of Radiation Oncology, Stadtspital Triemli, Zurich, Switzerland.; (Spichiger E) 1Nursing Science, Department Public Health, University of Basel, Bernoullistrasse 28, CH-4056 Basel, Switzerland.

(Valenta S) 1Nursing Science, Department Public Health, University of Basel, Bernoullistrasse 28, CH-4056 Basel, Switzerland.; (Spirig R) 1Nursing Science, Department Public Health, University of Basel, Bernoullistrasse 28, CH-4056 Basel, Switzerland.; (Miaskowski C) 4School of Nursing, University of California San Francisco (UCSF), San Francisco, USA.; (Zaugg K) 5Department of Radiation Oncology, Stadtspital Triemli, Zurich, Switzerland.; (Spichiger E) 1Nursing Science,

Department Public Health, University of Basel, Bernoullistrasse 28, CH-4056 Basel, Switzerland.
(Valenta S) 1Nursing Science, Department Public Health, University of Basel, Bernoullistrasse 28, CH-4056 Basel, Switzerland.; (Spirig R) 1Nursing Science, Department Public Health, University of Basel, Bernoullistrasse 28, CH-4056 Basel, Switzerland.; (Miaskowski C) 4School of Nursing, University of California San Francisco (UCSF), San Francisco, USA.; (Zaugg K) 5Department of Radiation Oncology, Stadtspital Triemli, Zurich, Switzerland.; (Spichiger E) 1Nursing Science, Department Public Health, University of Basel, Bernoullistrasse 28, CH-4056 Basel, Switzerland.
(Valenta S) 1Nursing Science, Department Public Health, University of Basel, Bernoullistrasse 28, CH-4056 Basel, Switzerland.; (Spirig R) 1Nursing Science, Department Public Health, University of Basel, Bernoullistrasse 28, CH-4056 Basel, Switzerland.; (Miaskowski C) 4School of Nursing, University of California San Francisco (UCSF), San Francisco, USA.; (Zaugg K) 5Department of Radiation Oncology, Stadtspital Triemli, Zurich, Switzerland.; (Spichiger E) 1Nursing Science, Department Public Health, University of Basel, Bernoullistrasse 28, CH-4056 Basel, Switzerland.
Database: PubMed

56. A Survey of the Knowledge and Attitudes of Oncology Nurses toward Pain in United Arab Emirates Oncology Settings.

Author(s): Al-Atiyyat N; Salim NA; Tuffaha MG; Abu Nigim HA; Saleh MM; Alkhodary ME; Brant JM

Source: Pain management nursing : official journal of the American Society of Pain Management Nurses; 2019; vol. 20 (no. 3); p. 276-283

Publication Date: 2019

Publication Type(s): Journal Article; Research Support, Non-U.S. Gov't

DOI: http://dx.doi.org/10.1016/j.pmn.2018.08.005

ISSN: 1532-8635

Place of Publication: United States

PubMedID: 30527855

Accession Number: 30527855

Abstract:BACKGROUND: Effective cancer pain management mandates precise attitude, assessment, skills, and knowledge. Health professionals' knowledge and attitudes concerning cancer pain management have often been referred to as insufficient.AIMS: This study explored pain knowledge and attitudes of nurses working in oncology settings.SETTING AND PARTICIPANTS: Population 115 oncology nurses working at 2 hospitals in the United Arab Emirates.METHODS: A descriptive, correlational, cross-sectional design was used to examine nurse knowledge and attitudes about pain using the Nurses' Attitude and Knowledge Survey Regarding Pain (NKASRP) survey. NKASRP score differences were examined among nurses with varying demographics, levels of pain education and experience.RESULTS: The mean KASRP was 45%, significantly below the passing score of 80%. Pain management education was not found to have a significant impact on KASRP thus suggesting the need for more effective educational

approaches to developing appropriate knowledge and attitudes towards pain among the nurses. No significant differences between sex, educational level, nursing and oncology experience, and nationality or religion were found.INTERPRETATION AND CONCLUSIONS: The study highlights the need for new initiatives targeting nurses working with cancer patients who are likely to experience significant pain. An ongoing need exists for more effective evidence-based educational programs in cancer pain management. Interactive teaching strategies such as on the job training, improvisational learning, and case studies should be tested for their influence on pain knowledge and attitudes and patient outcomes.

Institutions:

(Al-Atiyyat N) Hashemite University, Nursing Faculty, Adult Health Department, Zarqa, Jordan.; (Salim NA) Dubai Health Authority, Dubai Hospital, Dubai, United Arab Emirates.; (Tuffaha MG) Dubai Health Authority, Dubai Hospital, Dubai, United Arab Emirates.; (Abu Nigim HA) Dubai Health Authority, Dubai Hospital, Dubai, United Arab Emirates.; (Saleh MM) Tawam Hospital, Al-Ain, Abu Dhabi, United Arab Emirates.; (Alkhodary ME) Dubai Health Authority, Dubai Hospital, Dubai, United Arab Emirates.; (Brant JM) Billings Clinic, Billings, Montana. Electronic address: jbrant@billingsclinic.org.
(Al-Atiyyat N) Hashemite University, Nursing Faculty, Adult Health Department, Zarqa, Jordan.; (Salim NA) Dubai Health Authority, Dubai Hospital, Dubai, United Arab Emirates.; (Tuffaha MG) Dubai Health Authority, Dubai Hospital, Dubai, United Arab Emirates.; (Abu Nigim HA) Dubai Health Authority, Dubai Hospital, Dubai, United Arab Emirates.; (Saleh MM) Tawam Hospital, Al-Ain, Abu Dhabi, United Arab Emirates.; (Alkhodary ME) Dubai Health Authority, Dubai Hospital, Dubai, United Arab Emirates.; (Brant JM) Billings Clinic, Billings, Montana. Electronic address: jbrant@billingsclinic.org.
(Al-Atiyyat N) Hashemite University, Nursing Faculty, Adult Health Department, Zarqa, Jordan.; (Salim NA) Dubai Health Authority, Dubai Hospital, Dubai, United Arab Emirates.; (Tuffaha MG) Dubai Health Authority, Dubai Hospital, Dubai, United Arab Emirates.; (Abu Nigim HA) Dubai Health Authority, Dubai Hospital, Dubai, United Arab Emirates.; (Saleh MM) Tawam Hospital, Al-Ain, Abu Dhabi, United Arab Emirates.; (Alkhodary ME) Dubai Health Authority, Dubai Hospital, Dubai, United Arab Emirates.; (Brant JM) Billings Clinic, Billings, Montana. Electronic address: jbrant@billingsclinic.org.
(Al-Atiyyat N) Hashemite University, Nursing Faculty, Adult Health Department, Zarqa, Jordan.; (Salim NA) Dubai Health Authority, Dubai Hospital, Dubai, United Arab Emirates.; (Tuffaha MG) Dubai Health Authority, Dubai Hospital, Dubai, United Arab Emirates.; (Abu Nigim HA) Dubai Health Authority, Dubai Hospital, Dubai, United Arab Emirates.; (Saleh MM) Tawam Hospital, Al-Ain, Abu Dhabi, United Arab Emirates.; (Alkhodary ME) Dubai Health Authority, Dubai Hospital, Dubai, United Arab Emirates.; (Brant JM) Billings Clinic, Billings, Montana. Electronic address: jbrant@billingsclinic.org.
(Al-Atiyyat N) Hashemite University, Nursing Faculty, Adult Health Department, Zarqa, Jordan.; (Salim NA) Dubai Health Authority, Dubai Hospital, Dubai, United Arab Emirates.; (Tuffaha MG) Dubai Health Authority, Dubai Hospital, Dubai, United Arab Emirates.; (Abu Nigim HA) Dubai Health Authority, Dubai Hospital, Dubai, United Arab Emirates.; (Saleh MM) Tawam Hospital, Al-Ain, Abu Dhabi, United Arab Emirates.; (Alkhodary ME) Dubai Health Authority, Dubai Hospital, Dubai, United Arab Emirates.; (Brant JM) Billings Clinic, Billings, Montana. Electronic address: jbrant@billingsclinic.org.

(Al-Atiyyat N) Hashemite University, Nursing Faculty, Adult Health Department, Zarqa, Jordan.; (Salim NA) Dubai Health Authority, Dubai Hospital, Dubai, United Arab Emirates.; (Tuffaha MG) Dubai Health Authority, Dubai Hospital, Dubai, United Arab Emirates.; (Abu Nigim HA) Dubai Health Authority, Dubai Hospital, Dubai, United Arab Emirates.; (Saleh MM) Tawam Hospital, Al-Ain, Abu Dhabi, United Arab Emirates.; (Alkhodary ME) Dubai Health Authority, Dubai Hospital, Dubai, United Arab Emirates.; (Brant JM) Billings Clinic, Billings, Montana. Electronic address: jbrant@billingsclinic.org.
(Al-Atiyyat N) Hashemite University, Nursing Faculty, Adult Health Department, Zarqa, Jordan.; (Salim NA) Dubai Health Authority, Dubai Hospital, Dubai, United Arab Emirates.; (Tuffaha MG) Dubai Health Authority, Dubai Hospital, Dubai, United Arab Emirates.; (Abu Nigim HA) Dubai Health Authority, Dubai Hospital, Dubai, United Arab Emirates.; (Saleh MM) Tawam Hospital, Al-Ain, Abu Dhabi, United Arab Emirates.; (Alkhodary ME) Dubai Health Authority, Dubai Hospital, Dubai, United Arab Emirates.; (Brant JM) Billings Clinic, Billings, Montana. Electronic address: jbrant@billingsclinic.org.
Database: PubMed

57. Healthcare provider knowledge, attitudes, beliefs, and practices surrounding the prescription of opioids for chronic non-cancer pain in North America: protocol for a mixed-method systematic review.

Author(s): Rash JA; Buckley N; Busse JW; Campbell TS; Corace K; Cooper L; Flusk D; Iorio A; Lavoie KL; Poulin PA; Skidmore B

Source: Systematic reviews; ; vol. 7 (no. 1); p. 189

Publication Type(s): Journal Article; Research Support, Non-U.S. Gov't; Systematic Review

DOI: http://dx.doi.org/10.1186/s13643-018-0858-7

ISSN: 2046-4053

Place of Publication: England

PubMedID: 30424800

Accession Number: 30424800

Available at Systematic reviews - from BioMed Central

Available at Systematic reviews - from Europe PubMed Central - Open Access

Available at Systematic reviews - from ProQuest (MEDLINE with Full Text) - NHS Version

Available at Systematic reviews - from ProQuest (Health Research Premium) - NHS Version

Available at Systematic reviews - from Unpaywall

Keywords: Subject Terms: *Chronic pain; *Clinical inertia; *Clinical practice guideline adherence; *Opioids; *Systematic review

Abstract:BACKGROUND: Evidence from diverse areas of medicine (e.g., cardiovascular disease, diabetes) indicates that healthcare providers (HCPs) often do not adhere to clinical practice guidelines (CPGs) despite a clear indication to implement recommendations-a phenomenon commonly termed clinical inertia. There are a variety of reasons for clinical inertia, but HCP-related factors (e.g., knowledge,

motivation, agreement with guidelines) are the most salient and amenable to intervention aimed to improve adherence. CPGs have been developed to support the safe and effective prescription of opioid medication for the management of chronic non-cancer pain. The extent of physician uptake and adherence to such guidelines is not yet well understood. The purpose of this review is to synthesize the published evidence about knowledge, attitudes, beliefs, and practices that HCPs hold regarding the prescription of opioids for chronic non-cancer pain.METHODS: An experienced information specialist will perform searches of CINAHL, Embase, MEDLINE, and PsycINFO bibliographic databases. The Cochrane library, PROSPERO, and the Joanna Briggs Institute will be searched for systematic reviews. Searches will be performed from inception to the present. Quantitative and qualitative study designs that report on HCP knowledge, attitudes, beliefs, or practices in North America will be eligible for inclusion. Studies reporting on interventions to improve HCP adherence to opioid prescribing CPGs will also be eligible for inclusion. Two trained graduate-level research assistants will independently screen articles for inclusion, perform data extraction, and perform risk of bias and quality assessment using recommended tools. Confidence in qualitative evidence will be evaluated using the Grades of Recommendation, Assessment, Development, and Evaluation-Confidence in the Evidence from Qualitative Reviews (GRADE-CERQual) approach. Confidence in quantitative evidence will be assessed using the GRADE approach.DISCUSSION: The ultimate goal of this work is to support interventions aiming to optimize opioid prescribing practices in order to prevent opioid-related morbidity and mortality without restricting a HCP's ability to select the most appropriate treatment for an individual patient.SYSTEMATIC REVIEW REGISTRATION: PROSPERO CRD42018091640 .

Institutions:

(Rash JA) Department of Psychology, Memorial University of Newfoundland, 230 Elizabeth Ave, St. John's, NL, A1B 3X9, Canada. jarash@mun.ca.; (Buckley N) Department of Anesthesia, McMaster University, Hamilton, ON, Canada.; (Busse JW) Department of Anesthesia, McMaster University, Hamilton, ON, Canada.; (Campbell TS) Department of Psychology, University of Calgary, Calgary, AB, Canada.; (Corace K) The Royal Ottawa Mental Health Centre, Ottawa, ON, Canada.; (Cooper L) Canadian Injured Workers Alliance, Thunder Bay, ON, Canada.; (Flusk D) Department of Anesthesia, Memorial University of Newfoundland, St. John's, NL, Canada.; (Iorio A) Department of Health Research Methods, Evidence and Impact, McMaster University, Hamilton, ON, Canada.; (Lavoie KL) Department of Psychology, University of Quebec at Montreal, Montreal, QC, Canada.; (Poulin PA) The Ottawa Hospital Research Institute, Ottawa, ON, Canada.; (Skidmore B) Independent Information Specialist, Ottawa, ON, Canada.
(Rash JA) Department of Psychology, Memorial University of Newfoundland, 230 Elizabeth Ave, St. John's, NL, A1B 3X9, Canada. jarash@mun.ca.; (Buckley N) Department of Anesthesia, McMaster University, Hamilton, ON, Canada.; (Busse JW) Department of Anesthesia, McMaster University, Hamilton, ON, Canada.; (Campbell TS) Department of Psychology, University of Calgary, Calgary, AB, Canada.; (Corace K) The Royal Ottawa Mental Health Centre, Ottawa, ON, Canada.; (Cooper L) Canadian Injured Workers Alliance, Thunder Bay, ON, Canada.; (Flusk D) Department of Anesthesia, Memorial University of Newfoundland, St. John's, NL, Canada.; (Iorio A) Department of Health Research Methods, Evidence and Impact, McMaster University, Hamilton, ON, Canada.; (Lavoie KL) Department of Psychology, University of Quebec at Montreal, Montreal, QC, Canada.; (Poulin PA)

The Ottawa Hospital Research Institute, Ottawa, ON, Canada.; (Skidmore B) Independent Information Specialist, Ottawa, ON, Canada.
(Rash JA) Department of Psychology, Memorial University of Newfoundland, 230 Elizabeth Ave, St. John's, NL, A1B 3X9, Canada. jarash@mun.ca.; (Buckley N) Department of Anesthesia, McMaster University, Hamilton, ON, Canada.; (Busse JW) Department of Anesthesia, McMaster University, Hamilton, ON, Canada.; (Campbell TS) Department of Psychology, University of Calgary, Calgary, AB, Canada.; (Corace K) The Royal Ottawa Mental Health Centre, Ottawa, ON, Canada.; (Cooper L) Canadian Injured Workers Alliance, Thunder Bay, ON, Canada.; (Flusk D) Department of Anesthesia, Memorial University of Newfoundland, St. John's, NL, Canada.; (Iorio A) Department of Health Research Methods, Evidence and Impact, McMaster University, Hamilton, ON, Canada.; (Lavoie KL) Department of Psychology, University of Quebec at Montreal, Montreal, QC, Canada.; (Poulin PA) The Ottawa Hospital Research Institute, Ottawa, ON, Canada.; (Skidmore B) Independent Information Specialist, Ottawa, ON, Canada.
(Rash JA) Department of Psychology, Memorial University of Newfoundland, 230 Elizabeth Ave, St. John's, NL, A1B 3X9, Canada. jarash@mun.ca.; (Buckley N) Department of Anesthesia, McMaster University, Hamilton, ON, Canada.; (Busse JW) Department of Anesthesia, McMaster University, Hamilton, ON, Canada.; (Campbell TS) Department of Psychology, University of Calgary, Calgary, AB, Canada.; (Corace K) The Royal Ottawa Mental Health Centre, Ottawa, ON, Canada.; (Cooper L) Canadian Injured Workers Alliance, Thunder Bay, ON, Canada.; (Flusk D) Department of Anesthesia, Memorial University of Newfoundland, St. John's, NL, Canada.; (Iorio A) Department of Health Research Methods, Evidence and Impact, McMaster University, Hamilton, ON, Canada.; (Lavoie KL) Department of Psychology, University of Quebec at Montreal, Montreal, QC, Canada.; (Poulin PA) The Ottawa Hospital Research Institute, Ottawa, ON, Canada.; (Skidmore B) Independent Information Specialist, Ottawa, ON, Canada.
(Rash JA) Department of Psychology, Memorial University of Newfoundland, 230 Elizabeth Ave, St. John's, NL, A1B 3X9, Canada. jarash@mun.ca.; (Buckley N) Department of Anesthesia, McMaster University, Hamilton, ON, Canada.; (Busse JW) Department of Anesthesia, McMaster University, Hamilton, ON, Canada.; (Campbell TS) Department of Psychology, University of Calgary, Calgary, AB, Canada.; (Corace K) The Royal Ottawa Mental Health Centre, Ottawa, ON, Canada.; (Cooper L) Canadian Injured Workers Alliance, Thunder Bay, ON, Canada.; (Flusk D) Department of Anesthesia, Memorial University of Newfoundland, St. John's, NL, Canada.; (Iorio A) Department of Health Research Methods, Evidence and Impact, McMaster University, Hamilton, ON, Canada.; (Lavoie KL) Department of Psychology, University of Quebec at Montreal, Montreal, QC, Canada.; (Poulin PA) The Ottawa Hospital Research Institute, Ottawa, ON, Canada.; (Skidmore B) Independent Information Specialist, Ottawa, ON, Canada.
(Rash JA) Department of Psychology, Memorial University of Newfoundland, 230 Elizabeth Ave, St. John's, NL, A1B 3X9, Canada. jarash@mun.ca.; (Buckley N) Department of Anesthesia, McMaster University, Hamilton, ON, Canada.; (Busse JW) Department of Anesthesia, McMaster University, Hamilton, ON, Canada.; (Campbell TS) Department of Psychology, University of Calgary, Calgary, AB, Canada.; (Corace K) The Royal Ottawa Mental Health Centre, Ottawa, ON, Canada.; (Cooper L) Canadian Injured Workers Alliance, Thunder Bay, ON, Canada.; (Flusk D) Department of Anesthesia, Memorial University of Newfoundland, St. John's, NL, Canada.; (Iorio A) Department of Health Research Methods, Evidence and Impact,

McMaster University, Hamilton, ON, Canada.; (Lavoie KL) Department of Psychology, University of Quebec at Montreal, Montreal, QC, Canada.; (Poulin PA) The Ottawa Hospital Research Institute, Ottawa, ON, Canada.; (Skidmore B) Independent Information Specialist, Ottawa, ON, Canada.
(Rash JA) Department of Psychology, Memorial University of Newfoundland, 230 Elizabeth Ave, St. John's, NL, A1B 3X9, Canada. jarash@mun.ca.; (Buckley N) Department of Anesthesia, McMaster University, Hamilton, ON, Canada.; (Busse JW) Department of Anesthesia, McMaster University, Hamilton, ON, Canada.; (Campbell TS) Department of Psychology, University of Calgary, Calgary, AB, Canada.; (Corace K) The Royal Ottawa Mental Health Centre, Ottawa, ON, Canada.; (Cooper L) Canadian Injured Workers Alliance, Thunder Bay, ON, Canada.; (Flusk D) Department of Anesthesia, Memorial University of Newfoundland, St. John's, NL, Canada.; (Iorio A) Department of Health Research Methods, Evidence and Impact, McMaster University, Hamilton, ON, Canada.; (Lavoie KL) Department of Psychology, University of Quebec at Montreal, Montreal, QC, Canada.; (Poulin PA) The Ottawa Hospital Research Institute, Ottawa, ON, Canada.; (Skidmore B) Independent Information Specialist, Ottawa, ON, Canada.
(Rash JA) Department of Psychology, Memorial University of Newfoundland, 230 Elizabeth Ave, St. John's, NL, A1B 3X9, Canada. jarash@mun.ca.; (Buckley N) Department of Anesthesia, McMaster University, Hamilton, ON, Canada.; (Busse JW) Department of Anesthesia, McMaster University, Hamilton, ON, Canada.; (Campbell TS) Department of Psychology, University of Calgary, Calgary, AB, Canada.; (Corace K) The Royal Ottawa Mental Health Centre, Ottawa, ON, Canada.; (Cooper L) Canadian Injured Workers Alliance, Thunder Bay, ON, Canada.; (Flusk D) Department of Anesthesia, Memorial University of Newfoundland, St. John's, NL, Canada.; (Iorio A) Department of Health Research Methods, Evidence and Impact, McMaster University, Hamilton, ON, Canada.; (Lavoie KL) Department of Psychology, University of Quebec at Montreal, Montreal, QC, Canada.; (Poulin PA) The Ottawa Hospital Research Institute, Ottawa, ON, Canada.; (Skidmore B) Independent Information Specialist, Ottawa, ON, Canada.
(Rash JA) Department of Psychology, Memorial University of Newfoundland, 230 Elizabeth Ave, St. John's, NL, A1B 3X9, Canada. jarash@mun.ca.; (Buckley N) Department of Anesthesia, McMaster University, Hamilton, ON, Canada.; (Busse JW) Department of Anesthesia, McMaster University, Hamilton, ON, Canada.; (Campbell TS) Department of Psychology, University of Calgary, Calgary, AB, Canada.; (Corace K) The Royal Ottawa Mental Health Centre, Ottawa, ON, Canada.; (Cooper L) Canadian Injured Workers Alliance, Thunder Bay, ON, Canada.; (Flusk D) Department of Anesthesia, Memorial University of Newfoundland, St. John's, NL, Canada.; (Iorio A) Department of Health Research Methods, Evidence and Impact, McMaster University, Hamilton, ON, Canada.; (Lavoie KL) Department of Psychology, University of Quebec at Montreal, Montreal, QC, Canada.; (Poulin PA) The Ottawa Hospital Research Institute, Ottawa, ON, Canada.; (Skidmore B) Independent Information Specialist, Ottawa, ON, Canada.
(Rash JA) Department of Psychology, Memorial University of Newfoundland, 230 Elizabeth Ave, St. John's, NL, A1B 3X9, Canada. jarash@mun.ca.; (Buckley N) Department of Anesthesia, McMaster University, Hamilton, ON, Canada.; (Busse JW) Department of Anesthesia, McMaster University, Hamilton, ON, Canada.; (Campbell TS) Department of Psychology, University of Calgary, Calgary, AB, Canada.; (Corace K) The Royal Ottawa Mental Health Centre, Ottawa, ON, Canada.; (Cooper L) Canadian Injured Workers Alliance, Thunder Bay, ON, Canada.; (Flusk D)

Department of Anesthesia, Memorial University of Newfoundland, St. John's, NL, Canada.; (Iorio A) Department of Health Research Methods, Evidence and Impact, McMaster University, Hamilton, ON, Canada.; (Lavoie KL) Department of Psychology, University of Quebec at Montreal, Montreal, QC, Canada.; (Poulin PA) The Ottawa Hospital Research Institute, Ottawa, ON, Canada.; (Skidmore B) Independent Information Specialist, Ottawa, ON, Canada.
(Rash JA) Department of Psychology, Memorial University of Newfoundland, 230 Elizabeth Ave, St. John's, NL, A1B 3X9, Canada. jarash@mun.ca.; (Buckley N) Department of Anesthesia, McMaster University, Hamilton, ON, Canada.; (Busse JW) Department of Anesthesia, McMaster University, Hamilton, ON, Canada.; (Campbell TS) Department of Psychology, University of Calgary, Calgary, AB, Canada.; (Corace K) The Royal Ottawa Mental Health Centre, Ottawa, ON, Canada.; (Cooper L) Canadian Injured Workers Alliance, Thunder Bay, ON, Canada.; (Flusk D) Department of Anesthesia, Memorial University of Newfoundland, St. John's, NL, Canada.; (Iorio A) Department of Health Research Methods, Evidence and Impact, McMaster University, Hamilton, ON, Canada.; (Lavoie KL) Department of Psychology, University of Quebec at Montreal, Montreal, QC, Canada.; (Poulin PA) The Ottawa Hospital Research Institute, Ottawa, ON, Canada.; (Skidmore B) Independent Information Specialist, Ottawa, ON, Canada.
Database: PubMed

58. [Elaboration and evaluation of a therapeutic education program in cancer pain management].

Author(s): Prevost V; Clarisse B; Heutte N; Leconte A; Bisson C; Bignon R; Cauchin S; Feuillet M; Gehanne S; Gicquère M; Grach MC; Guillaumé C; Le Gal C; Le Garrec J; Lecaer F; Lepleux I; Millet AL; Ropartz MC; Roux N; Sep Hieng V; Van Delook C; Bechet C; Le Chevalier A; Delorme C

Source: Bulletin du cancer; Nov 2018; vol. 105 (no. 11); p. 1074-1083

Publication Date: Nov 2018

Publication Type(s): Journal Article; Review

DOI: http://dx.doi.org/10.1016/j.bulcan.2018.08.012

ISSN: 1769-6917

Place of Publication: France

PubMedID: 30327192

Accession Number: 30327192

Available at Bulletin du cancer - from Unpaywall

Keywords: Subject Terms: Cancer pain; Douleur liée au cancer; Gestion de la douleur; Pain assessment; Pain management; Therapeutic patient education; Éducation thérapeutique du patient; Évaluation de la douleur

Abstract: Pain, one of the most feared symptoms for patients with cancer, remains insufficiently alleviated and impairs quality of life. Therapeutic patient education (TPE) is a relevant approach to this problem while allowing patients to develop skills to better manage their pain. In the "Basse-Normandie" French region, the management of pain relies on two organized networks, thus allowing proximity and accessibility for all concerned. In this context, our team has begun a broad five-step research program that is part of a regional health policy: (1) training in TPE of 10

doctor/nurse pairs; (2) identification of educational expectations of patients and their relatives in the field of cancer pain; (3) design and optimization of a TPE program dedicated to cancer pain; (4) regional pilot study aiming to assess the feasibility, quality and transferability of the program; (5) evaluation of the TPE program by interventional comparative randomization at the national level. This article aims to present the program which originality and strengths are based on collaborative work between health stakeholders. Objectives, methodology and expected results of the research phase (stages 2, 4, 5) are notably developed. The main expected outcomes are to prove the effectiveness of the program in improving the knowledge and skills of patients in the field of pain cancer in order to promote their adherence to treatment and, consequently, to enable them to better manage it. The long-term objective is to disseminate the educational approach by modifying practices that provide a mutual benefit for caregivers and patients.

Institutions:
(Prevost V) Université de Caen Normandie, UMR 1086 Inserm, unité de recherche interdisciplinaire pour la prévention et le traitement des cancers « ANTICIPE », 14000 Caen, France; Centre régional de lutte contre le cancer François-Baclesse, 14000 Caen, France. Electronic address: virginie.prevost@unicaen.fr.; (Clarisse B) Centre régional de lutte contre le cancer François-Baclesse, 14000 Caen, France.; (Heutte N) Centre régional de lutte contre le cancer François-Baclesse, 14000 Caen, France; Normandie université, UNIROUEN, CETAPS EA 3832, 76130 Mont-Saint-Aignan, France.; (Leconte A) Centre régional de lutte contre le cancer François-Baclesse, 14000 Caen, France.; (Bisson C) Centre hospitalier, 14400 Bayeux, France; Réseau régional douleur en Basse-Normandie, 14400 Bayeux, France.; (Bignon R) Réseau régional douleur en Basse-Normandie, 14400 Bayeux, France; Centre hospitalier, 14100 Lisieux, France.; (Cauchin S) Réseau régional douleur en Basse-Normandie, 14400 Bayeux, France; Centre hospitalier intercommunal Alençon-Mamers, 61000 Alençon, France.; (Feuillet M) Réseau régional douleur en Basse-Normandie, 14400 Bayeux, France; Centre hospitalier, 50000 Saint-Lô, France.; (Gehanne S) Réseau régional douleur en Basse-Normandie, 14400 Bayeux, France; Centre hospitalier, 50000 Saint-Lô, France.; (Gicquère M) Centre régional de lutte contre le cancer François-Baclesse, 14000 Caen, France; Réseau régional douleur en Basse-Normandie, 14400 Bayeux, France.; (Grach MC) Centre régional de lutte contre le cancer François-Baclesse, 14000 Caen, France; Réseau régional douleur en Basse-Normandie, 14400 Bayeux, France.; (Guillaumé C) Réseau régional douleur en Basse-Normandie, 14400 Bayeux, France; CHU, 14000 Caen, France.; (Le Gal C) Réseau régional douleur en Basse-Normandie, 14400 Bayeux, France; Centre hospitalier, 61200 Argentan, France.; (Le Garrec J) Réseau régional douleur en Basse-Normandie, 14400 Bayeux, France; Centre hospitalier intercommunal Alençon-Mamers, 61000 Alençon, France.; (Lecaer F) Réseau régional douleur en Basse-Normandie, 14400 Bayeux, France; Centre hospitalier, 61100 Flers, France.; (Lepleux I) Réseau régional douleur en Basse-Normandie, 14400 Bayeux, France; Centre hospitalier, 50100 Cherbourg, France.; (Millet AL) Réseau régional douleur en Basse-Normandie, 14400 Bayeux, France; Centre hospitalier, 61100 Flers, France.; (Ropartz MC) Réseau régional douleur en Basse-Normandie, 14400 Bayeux, France; Centre hospitalier, 50400 Avranches-Granville, France.; (Roux N) Réseau régional douleur en Basse-Normandie, 14400 Bayeux, France; CHU, 14000 Caen, France.; (Sep Hieng V) Réseau régional douleur en Basse-Normandie, 14400 Bayeux, France; Centre hospitalier, 14100 Lisieux, France.; (Van Delook C) Réseau régional douleur en Basse-Normandie, 14400

Bayeux, France; Centre hospitalier, 61200 Argentan, France.; (Bechet C) Pharmacie de la Croix d'Or, 75017 Paris, France.; (Le Chevalier A) Réseau régional douleur en Basse-Normandie, 14400 Bayeux, France; Centre hospitalier, 50400 Avranches-Granville, France.; (Delorme C) Centre hospitalier, 14400 Bayeux, France; Réseau régional douleur en Basse-Normandie, 14400 Bayeux, France.

(Prevost V) Université de Caen Normandie, UMR 1086 Inserm, unité de recherche interdisciplinaire pour la prévention et le traitement des cancers « ANTICIPE », 14000 Caen, France; Centre régional de lutte contre le cancer François-Baclesse, 14000 Caen, France. Electronic address: virginie.prevost@unicaen.fr.; (Clarisse B) Centre régional de lutte contre le cancer François-Baclesse, 14000 Caen, France.; (Heutte N) Centre régional de lutte contre le cancer François-Baclesse, 14000 Caen, France; Normandie université, UNIROUEN, CETAPS EA 3832, 76130 Mont-Saint-Aignan, France.; (Leconte A) Centre régional de lutte contre le cancer François-Baclesse, 14000 Caen, France.; (Bisson C) Centre hospitalier, 14400 Bayeux, France; Réseau régional douleur en Basse-Normandie, 14400 Bayeux, France.; (Bignon R) Réseau régional douleur en Basse-Normandie, 14400 Bayeux, France; Centre hospitalier, 14100 Lisieux, France.; (Cauchin S) Réseau régional douleur en Basse-Normandie, 14400 Bayeux, France; Centre hospitalier intercommunal Alençon-Mamers, 61000 Alençon, France.; (Feuillet M) Réseau régional douleur en Basse-Normandie, 14400 Bayeux, France; Centre hospitalier, 50000 Saint-Lô, France.; (Gehanne S) Réseau régional douleur en Basse-Normandie, 14400 Bayeux, France; Centre hospitalier, 50000 Saint-Lô, France.; (Gicquère M) Centre régional de lutte contre le cancer François-Baclesse, 14000 Caen, France; Réseau régional douleur en Basse-Normandie, 14400 Bayeux, France.; (Grach MC) Centre régional de lutte contre le cancer François-Baclesse, 14000 Caen, France; Réseau régional douleur en Basse-Normandie, 14400 Bayeux, France.; (Guillaumé C) Réseau régional douleur en Basse-Normandie, 14400 Bayeux, France; CHU, 14000 Caen, France.; (Le Gal C) Réseau régional douleur en Basse-Normandie, 14400 Bayeux, France; Centre hospitalier, 61200 Argentan, France.; (Le Garrec J) Réseau régional douleur en Basse-Normandie, 14400 Bayeux, France; Centre hospitalier intercommunal Alençon-Mamers, 61000 Alençon, France.; (Lecaer F) Réseau régional douleur en Basse-Normandie, 14400 Bayeux, France; Centre hospitalier, 61100 Flers, France.; (Lepleux I) Réseau régional douleur en Basse-Normandie, 14400 Bayeux, France; Centre hospitalier, 50100 Cherbourg, France.; (Millet AL) Réseau régional douleur en Basse-Normandie, 14400 Bayeux, France; Centre hospitalier, 61100 Flers, France.; (Ropartz MC) Réseau régional douleur en Basse-Normandie, 14400 Bayeux, France; Centre hospitalier, 50400 Avranches-Granville, France.; (Roux N) Réseau régional douleur en Basse-Normandie, 14400 Bayeux, France; CHU, 14000 Caen, France.; (Sep Hieng V) Réseau régional douleur en Basse-Normandie, 14400 Bayeux, France; Centre hospitalier, 14100 Lisieux, France.; (Van Delook C) Réseau régional douleur en Basse-Normandie, 14400
Bayeux, France; Centre hospitalier, 61200 Argentan, France.; (Bechet C) Pharmacie de la Croix d'Or, 75017 Paris, France.; (Le Chevalier A) Réseau régional douleur en Basse-Normandie, 14400 Bayeux, France; Centre hospitalier, 50400 Avranches-Granville, France.; (Delorme C) Centre hospitalier, 14400 Bayeux, France; Réseau régional douleur en Basse-Normandie, 14400 Bayeux, France.

(Prevost V) Université de Caen Normandie, UMR 1086 Inserm, unité de recherche interdisciplinaire pour la prévention et le traitement des cancers « ANTICIPE », 14000 Caen, France; Centre régional de lutte contre le cancer François-Baclesse, 14000 Caen, France. Electronic address: virginie.prevost@unicaen.fr.; (Clarisse B)

Centre régional de lutte contre le cancer François-Baclesse, 14000 Caen, France.; (Heutte N) Centre régional de lutte contre le cancer François-Baclesse, 14000 Caen, France; Normandie université, UNIROUEN, CETAPS EA 3832, 76130 Mont-Saint-Aignan, France.; (Leconte A) Centre régional de lutte contre le cancer François-Baclesse, 14000 Caen, France.; (Bisson C) Centre hospitalier, 14400 Bayeux, France; Réseau régional douleur en Basse-Normandie, 14400 Bayeux, France.; (Bignon R) Réseau régional douleur en Basse-Normandie, 14400 Bayeux, France; Centre hospitalier, 14100 Lisieux, France.; (Cauchin S) Réseau régional douleur en Basse-Normandie, 14400 Bayeux, France; Centre hospitalier intercommunal Alençon-Mamers, 61000 Alençon, France.; (Feuillet M) Réseau régional douleur en Basse-Normandie, 14400 Bayeux, France; Centre hospitalier, 50000 Saint-Lô, France.; (Gehanne S) Réseau régional douleur en Basse-Normandie, 14400 Bayeux, France; Centre hospitalier, 50000 Saint-Lô, France.; (Gicquère M) Centre régional de lutte contre le cancer François-Baclesse, 14000 Caen, France; Réseau régional douleur en Basse-Normandie, 14400 Bayeux, France.; (Grach MC) Centre régional de lutte contre le cancer François-Baclesse, 14000 Caen, France; Réseau régional douleur en Basse-Normandie, 14400 Bayeux, France.; (Guillaumé C) Réseau régional douleur en Basse-Normandie, 14400 Bayeux, France; CHU, 14000 Caen, France.; (Le Gal C) Réseau régional douleur en Basse-Normandie, 14400 Bayeux, France; Centre hospitalier, 61200 Argentan, France.; (Le Garrec J) Réseau régional douleur en Basse-Normandie, 14400 Bayeux, France; Centre hospitalier intercommunal Alençon-Mamers, 61000 Alençon, France.; (Lecaer F) Réseau régional douleur en Basse-Normandie, 14400 Bayeux, France; Centre hospitalier, 61100 Flers, France.; (Lepleux I) Réseau régional douleur en Basse-Normandie, 14400 Bayeux, France; Centre hospitalier, 50100 Cherbourg, France.; (Millet AL) Réseau régional douleur en Basse-Normandie, 14400 Bayeux, France; Centre hospitalier, 61100 Flers, France.; (Ropartz MC) Réseau régional douleur en Basse-Normandie, 14400 Bayeux, France; Centre hospitalier, 50400 Avranches-Granville, France.; (Roux N) Réseau régional douleur en Basse-Normandie, 14400 Bayeux, France; CHU, 14000 Caen, France.; (Sep Hieng V) Réseau régional douleur en Basse-Normandie, 14400 Bayeux, France; Centre hospitalier, 14100 Lisieux, France.; (Van Delook C) Réseau régional douleur en Basse-Normandie, 14400 Bayeux, France; Centre hospitalier, 61200 Argentan, France.; (Bechet C) Pharmacie de la Croix d'Or, 75017 Paris, France.; (Le Chevalier A) Réseau régional douleur en Basse-Normandie, 14400 Bayeux, France; Centre hospitalier, 50400 Avranches-Granville, France.; (Delorme C) Centre hospitalier, 14400 Bayeux, France; Réseau régional douleur en Basse-Normandie, 14400 Bayeux, France.
(Prevost V) Université de Caen Normandie, UMR 1086 Inserm, unité de recherche interdisciplinaire pour la prévention et le traitement des cancers « ANTICIPE », 14000 Caen, France; Centre régional de lutte contre le cancer François-Baclesse, 14000 Caen, France. Electronic address: virginie.prevost@unicaen.fr.; (Clarisse B) Centre régional de lutte contre le cancer François-Baclesse, 14000 Caen, France.; (Heutte N) Centre régional de lutte contre le cancer François-Baclesse, 14000 Caen, France; Normandie université, UNIROUEN, CETAPS EA 3832, 76130 Mont-Saint-Aignan, France.; (Leconte A) Centre régional de lutte contre le cancer François-Baclesse, 14000 Caen, France.; (Bisson C) Centre hospitalier, 14400 Bayeux, France; Réseau régional douleur en Basse-Normandie, 14400 Bayeux, France.; (Bignon R) Réseau régional douleur en Basse-Normandie, 14400 Bayeux, France; Centre hospitalier, 14100 Lisieux, France.; (Cauchin S) Réseau régional douleur en Basse-Normandie, 14400 Bayeux, France; Centre hospitalier intercommunal

Alençon-Mamers, 61000 Alençon, France.; (Feuillet M) Réseau régional douleur en Basse-Normandie, 14400 Bayeux, France; Centre hospitalier, 50000 Saint-Lô, France.; (Gehanne S) Réseau régional douleur en Basse-Normandie, 14400 Bayeux, France; Centre hospitalier, 50000 Saint-Lô, France.; (Gicquère M) Centre régional de lutte contre le cancer François-Baclesse, 14000 Caen, France; Réseau régional douleur en Basse-Normandie, 14400 Bayeux, France.; (Grach MC) Centre régional de lutte contre le cancer François-Baclesse, 14000 Caen, France; Réseau régional douleur en Basse-Normandie, 14400 Bayeux, France.; (Guillaumé C) Réseau régional douleur en Basse-Normandie, 14400 Bayeux, France; CHU, 14000 Caen, France.; (Le Gal C) Réseau régional douleur en Basse-Normandie, 14400 Bayeux, France; Centre hospitalier, 61200 Argentan, France.; (Le Garrec J) Réseau régional douleur en Basse-Normandie, 14400 Bayeux, France; Centre hospitalier intercommunal Alençon-Mamers, 61000 Alençon, France.; (Lecaer F) Réseau régional douleur en Basse-Normandie, 14400 Bayeux, France; Centre hospitalier, 61100 Flers, France.; (Lepleux I) Réseau régional douleur en Basse-Normandie, 14400 Bayeux, France; Centre hospitalier, 50100 Cherbourg, France.; (Millet AL) Réseau régional douleur en Basse-Normandie, 14400 Bayeux, France; Centre hospitalier, 61100 Flers, France.; (Ropartz MC) Réseau régional douleur en Basse-Normandie, 14400 Bayeux, France; Centre hospitalier, 50400 Avranches-Granville, France.; (Roux N) Réseau régional douleur en Basse-Normandie, 14400 Bayeux, France; CHU, 14000 Caen, France.; (Sep Hieng V) Réseau régional douleur en Basse-Normandie, 14400 Bayeux, France; Centre hospitalier, 14100 Lisieux, France.; (Van Delook C) Réseau régional douleur en Basse-Normandie, 14400 Bayeux, France; Centre hospitalier, 61200 Argentan, France.; (Bechet C) Pharmacie de la Croix d'Or, 75017 Paris, France.; (Le Chevalier A) Réseau régional douleur en Basse-Normandie, 14400 Bayeux, France; Centre hospitalier, 50400 Avranches-Granville, France.; (Delorme C) Centre hospitalier, 14400 Bayeux, France; Réseau régional douleur en Basse-Normandie, 14400 Bayeux, France.
(Prevost V) Université de Caen Normandie, UMR 1086 Inserm, unité de recherche interdisciplinaire pour la prévention et le traitement des cancers « ANTICIPE », 14000 Caen, France; Centre régional de lutte contre le cancer François-Baclesse, 14000 Caen, France. Electronic address: virginie.prevost@unicaen.fr.; (Clarisse B) Centre régional de lutte contre le cancer François-Baclesse, 14000 Caen, France.; (Heutte N) Centre régional de lutte contre le cancer François-Baclesse, 14000 Caen, France; Normandie université, UNIROUEN, CETAPS EA 3832, 76130 Mont-Saint-Aignan, France.; (Leconte A) Centre régional de lutte contre le cancer François-Baclesse, 14000 Caen, France.; (Bisson C) Centre hospitalier, 14400 Bayeux, France; Réseau régional douleur en Basse-Normandie, 14400 Bayeux, France.; (Bignon R) Réseau régional douleur en Basse-Normandie, 14400 Bayeux, France; Centre hospitalier, 14100 Lisieux, France.; (Cauchin S) Réseau régional douleur en Basse-Normandie, 14400 Bayeux, France; Centre hospitalier intercommunal
Alençon-Mamers, 61000 Alençon, France.; (Feuillet M) Réseau régional douleur en Basse-Normandie, 14400 Bayeux, France; Centre hospitalier, 50000 Saint-Lô, France.; (Gehanne S) Réseau régional douleur en Basse-Normandie, 14400 Bayeux, France; Centre hospitalier, 50000 Saint-Lô, France.; (Gicquère M) Centre régional de lutte contre le cancer François-Baclesse, 14000 Caen, France; Réseau régional douleur en Basse-Normandie, 14400 Bayeux, France.; (Grach MC) Centre régional de lutte contre le cancer François-Baclesse, 14000 Caen, France; Réseau régional douleur en Basse-Normandie, 14400 Bayeux, France.; (Guillaumé C) Réseau régional douleur en Basse-Normandie, 14400 Bayeux, France; CHU, 14000

Caen, France.; (Le Gal C) Réseau régional douleur en Basse-Normandie, 14400 Bayeux, France; Centre hospitalier, 61200 Argentan, France.; (Le Garrec J) Réseau régional douleur en Basse-Normandie, 14400 Bayeux, France; Centre hospitalier intercommunal Alençon-Mamers, 61000 Alençon, France.; (Lecaer F) Réseau régional douleur en Basse-Normandie, 14400 Bayeux, France; Centre hospitalier, 61100 Flers, France.; (Lepleux I) Réseau régional douleur en Basse-Normandie, 14400 Bayeux, France; Centre hospitalier, 50100 Cherbourg, France.; (Millet AL) Réseau régional douleur en Basse-Normandie, 14400 Bayeux, France; Centre hospitalier, 61100 Flers, France.; (Ropartz MC) Réseau régional douleur en Basse-Normandie, 14400 Bayeux, France; Centre hospitalier, 50400 Avranches-Granville, France.; (Roux N) Réseau régional douleur en Basse-Normandie, 14400 Bayeux, France; CHU, 14000 Caen, France.; (Sep Hieng V) Réseau régional douleur en Basse-Normandie, 14400 Bayeux, France; Centre hospitalier, 14100 Lisieux, France.; (Van Delook C) Réseau régional douleur en Basse-Normandie, 14400 Bayeux, France; Centre hospitalier, 61200 Argentan, France.; (Bechet C) Pharmacie de la Croix d'Or, 75017 Paris, France.; (Le Chevalier A) Réseau régional douleur en Basse-Normandie, 14400 Bayeux, France; Centre hospitalier, 50400 Avranches-Granville, France.; (Delorme C) Centre hospitalier, 14400 Bayeux, France; Réseau régional douleur en Basse-Normandie, 14400 Bayeux, France.
(Prevost V) Université de Caen Normandie, UMR 1086 Inserm, unité de recherche interdisciplinaire pour la prévention et le traitement des cancers « ANTICIPE », 14000 Caen, France; Centre régional de lutte contre le cancer François-Baclesse, 14000 Caen, France. Electronic address: virginie.prevost@unicaen.fr.; (Clarisse B) Centre régional de lutte contre le cancer François-Baclesse, 14000 Caen, France.; (Heutte N) Centre régional de lutte contre le cancer François-Baclesse, 14000 Caen, France; Normandie université, UNIROUEN, CETAPS EA 3832, 76130 Mont-Saint-Aignan, France.; (Leconte A) Centre régional de lutte contre le cancer François-Baclesse, 14000 Caen, France.; (Bisson C) Centre hospitalier, 14400 Bayeux, France; Réseau régional douleur en Basse-Normandie, 14400 Bayeux, France.; (Bignon R) Réseau régional douleur en Basse-Normandie, 14400 Bayeux, France; Centre hospitalier, 14100 Lisieux, France.; (Cauchin S) Réseau régional douleur en Basse-Normandie, 14400 Bayeux, France; Centre hospitalier intercommunal Alençon-Mamers, 61000 Alençon, France.; (Feuillet M) Réseau régional douleur en Basse-Normandie, 14400 Bayeux, France; Centre hospitalier, 50000 Saint-Lô, France.; (Gehanne S) Réseau régional douleur en Basse-Normandie, 14400 Bayeux, France; Centre hospitalier, 50000 Saint-Lô, France.; (Gicquère M) Centre régional de lutte contre le cancer François-Baclesse, 14000 Caen, France; Réseau régional douleur en Basse-Normandie, 14400 Bayeux, France.; (Grach MC) Centre régional de lutte contre le cancer François-Baclesse, 14000 Caen, France; Réseau régional douleur en Basse-Normandie, 14400 Bayeux, France.; (Guillaumé C) Réseau régional douleur en Basse-Normandie, 14400 Bayeux, France; CHU, 14000 Caen, France.; (Le Gal C) Réseau régional douleur en Basse-Normandie, 14400 Bayeux, France; Centre hospitalier, 61200 Argentan, France.; (Le Garrec J) Réseau régional douleur en Basse-Normandie, 14400 Bayeux, France; Centre hospitalier intercommunal Alençon-Mamers, 61000 Alençon, France.; (Lecaer F) Réseau régional douleur en Basse-Normandie, 14400 Bayeux, France; Centre hospitalier, 61100 Flers, France.; (Lepleux I) Réseau régional douleur en Basse-Normandie, 14400 Bayeux, France; Centre hospitalier, 50100 Cherbourg, France.; (Millet AL) Réseau régional douleur en Basse-Normandie, 14400 Bayeux, France; Centre hospitalier, 61100 Flers, France.; (Ropartz MC) Réseau régional douleur en Basse-

Normandie, 14400 Bayeux, France; Centre hospitalier, 50400 Avranches-Granville, France.; (Roux N) Réseau régional douleur en Basse-Normandie, 14400 Bayeux, France; CHU, 14000 Caen, France.; (Sep Hieng V) Réseau régional douleur en Basse-Normandie, 14400 Bayeux, France; Centre hospitalier, 14100 Lisieux, France.; (Van Delook C) Réseau régional douleur en Basse-Normandie, 14400 Bayeux, France; Centre hospitalier, 61200 Argentan, France.; (Bechet C) Pharmacie de la Croix d'Or, 75017 Paris, France.; (Le Chevalier A) Réseau régional douleur en Basse-Normandie, 14400 Bayeux, France; Centre hospitalier, 50400 Avranches-Granville, France.; (Delorme C) Centre hospitalier, 14400 Bayeux, France; Réseau régional douleur en Basse-Normandie, 14400 Bayeux, France.
(Prevost V) Université de Caen Normandie, UMR 1086 Inserm, unité de recherche interdisciplinaire pour la prévention et le traitement des cancers « ANTICIPE », 14000 Caen, France; Centre régional de lutte contre le cancer François-Baclesse, 14000 Caen, France. Electronic address: virginie.prevost@unicaen.fr.; (Clarisse B) Centre régional de lutte contre le cancer François-Baclesse, 14000 Caen, France.; (Heutte N) Centre régional de lutte contre le cancer François-Baclesse, 14000 Caen, France; Normandie université, UNIROUEN, CETAPS EA 3832, 76130 Mont-Saint-Aignan, France.; (Leconte A) Centre régional de lutte contre le cancer François-Baclesse, 14000 Caen, France.; (Bisson C) Centre hospitalier, 14400 Bayeux, France; Réseau régional douleur en Basse-Normandie, 14400 Bayeux, France.; (Bignon R) Réseau régional douleur en Basse-Normandie, 14400 Bayeux, France; Centre hospitalier, 14100 Lisieux, France.; (Cauchin S) Réseau régional douleur en Basse-Normandie, 14400 Bayeux, France; Centre hospitalier intercommunal Alençon-Mamers, 61000 Alençon, France.; (Feuillet M) Réseau régional douleur en Basse-Normandie, 14400 Bayeux, France; Centre hospitalier, 50000 Saint-Lô, France.; (Gehanne S) Réseau régional douleur en Basse-Normandie, 14400 Bayeux, France; Centre hospitalier, 50000 Saint-Lô, France.; (Gicquère M) Centre régional de lutte contre le cancer François-Baclesse, 14000 Caen, France; Réseau régional douleur en Basse-Normandie, 14400 Bayeux, France.; (Grach MC) Centre régional de lutte contre le cancer François-Baclesse, 14000 Caen, France; Réseau régional douleur en Basse-Normandie, 14400 Bayeux, France.; (Guillaumé C) Réseau régional douleur en Basse-Normandie, 14400 Bayeux, France; CHU, 14000 Caen, France.; (Le Gal C) Réseau régional douleur en Basse-Normandie, 14400 Bayeux, France; Centre hospitalier, 61200 Argentan, France.; (Le Garrec J) Réseau régional douleur en Basse-Normandie, 14400 Bayeux, France; Centre hospitalier intercommunal Alençon-Mamers, 61000 Alençon, France.; (Lecaer F) Réseau régional douleur en Basse-Normandie, 14400 Bayeux, France; Centre hospitalier, 61100 Flers, France.; (Lepleux I) Réseau régional douleur en Basse-Normandie, 14400 Bayeux, France; Centre hospitalier, 50100 Cherbourg, France.; (Millet AL) Réseau régional douleur en Basse-Normandie, 14400 Bayeux, France; Centre hospitalier, 61100 Flers, France.; (Ropartz MC) Réseau régional douleur en Basse-Normandie, 14400 Bayeux, France; Centre hospitalier, 50400 Avranches-Granville, France.; (Roux N) Réseau régional douleur en Basse-Normandie, 14400 Bayeux, France; CHU, 14000 Caen, France.; (Sep Hieng V) Réseau régional douleur en Basse-Normandie, 14400 Bayeux, France; Centre hospitalier, 14100 Lisieux, France.; (Van Delook C) Réseau régional douleur en Basse-Normandie, 14400 Bayeux, France; Centre hospitalier, 61200 Argentan, France.; (Bechet C) Pharmacie de la Croix d'Or, 75017 Paris, France.; (Le Chevalier A) Réseau régional douleur en Basse-Normandie, 14400 Bayeux, France; Centre hospitalier, 50400 Avranches-

Granville, France.; (Delorme C) Centre hospitalier, 14400 Bayeux, France; Réseau régional douleur en Basse-Normandie, 14400 Bayeux, France.
(Prevost V) Université de Caen Normandie, UMR 1086 Inserm, unité de recherche interdisciplinaire pour la prévention et le traitement des cancers « ANTICIPE », 14000 Caen, France; Centre régional de lutte contre le cancer François-Baclesse, 14000 Caen, France. Electronic address: virginie.prevost@unicaen.fr.; (Clarisse B) Centre régional de lutte contre le cancer François-Baclesse, 14000 Caen, France.; (Heutte N) Centre régional de lutte contre le cancer François-Baclesse, 14000 Caen, France; Normandie université, UNIROUEN, CETAPS EA 3832, 76130 Mont-Saint-Aignan, France.; (Leconte A) Centre régional de lutte contre le cancer François-Baclesse, 14000 Caen, France.; (Bisson C) Centre hospitalier, 14400 Bayeux, France; Réseau régional douleur en Basse-Normandie, 14400 Bayeux, France.; (Bignon R) Réseau régional douleur en Basse-Normandie, 14400 Bayeux, France; Centre hospitalier, 14100 Lisieux, France.; (Cauchin S) Réseau régional douleur en Basse-Normandie, 14400 Bayeux, France; Centre hospitalier intercommunal Alençon-Mamers, 61000 Alençon, France.; (Feuillet M) Réseau régional douleur en Basse-Normandie, 14400 Bayeux, France; Centre hospitalier, 50000 Saint-Lô, France.; (Gehanne S) Réseau régional douleur en Basse-Normandie, 14400 Bayeux, France; Centre hospitalier, 50000 Saint-Lô, France.; (Gicquère M) Centre régional de lutte contre le cancer François-Baclesse, 14000 Caen, France; Réseau régional douleur en Basse-Normandie, 14400 Bayeux, France.; (Grach MC) Centre régional de lutte contre le cancer François-Baclesse, 14000 Caen, France; Réseau régional douleur en Basse-Normandie, 14400 Bayeux, France.; (Guillaumé C) Réseau régional douleur en Basse-Normandie, 14400 Bayeux, France; CHU, 14000 Caen, France.; (Le Gal C) Réseau régional douleur en Basse-Normandie, 14400 Bayeux, France; Centre hospitalier, 61200 Argentan, France.; (Le Garrec J) Réseau régional douleur en Basse-Normandie, 14400 Bayeux, France; Centre hospitalier intercommunal Alençon-Mamers, 61000 Alençon, France.; (Lecaer F) Réseau régional douleur en Basse-Normandie, 14400 Bayeux, France; Centre hospitalier, 61100 Flers, France.; (Lepleux I) Réseau régional douleur en Basse-Normandie, 14400 Bayeux, France; Centre hospitalier, 50100 Cherbourg, France.; (Millet AL) Réseau régional douleur en Basse-Normandie, 14400 Bayeux, France; Centre hospitalier, 61100 Flers, France.; (Ropartz MC) Réseau régional douleur en Basse-Normandie, 14400 Bayeux, France; Centre hospitalier, 50400 Avranches-Granville, France.; (Roux N) Réseau régional douleur en Basse-Normandie, 14400 Bayeux, France; CHU, 14000 Caen, France.; (Sep Hieng V) Réseau régional douleur en Basse-Normandie, 14400 Bayeux, France; Centre hospitalier, 14100 Lisieux, France.; (Van Delook C) Réseau régional douleur en Basse-Normandie, 14400 Bayeux, France; Centre hospitalier, 61200 Argentan, France.; (Bechet C) Pharmacie de la Croix d'Or, 75017 Paris, France.; (Le Chevalier A) Réseau régional douleur en Basse-Normandie, 14400 Bayeux, France; Centre hospitalier, 50400 Avranches-Granville, France.; (Delorme C) Centre hospitalier, 14400 Bayeux, France; Réseau régional douleur en Basse-Normandie, 14400 Bayeux, France.
(Prevost V) Université de Caen Normandie, UMR 1086 Inserm, unité de recherche interdisciplinaire pour la prévention et le traitement des cancers « ANTICIPE », 14000 Caen, France; Centre régional de lutte contre le cancer François-Baclesse, 14000 Caen, France. Electronic address: virginie.prevost@unicaen.fr.; (Clarisse B) Centre régional de lutte contre le cancer François-Baclesse, 14000 Caen, France.; (Heutte N) Centre régional de lutte contre le cancer François-Baclesse, 14000 Caen, France; Normandie université, UNIROUEN, CETAPS EA 3832, 76130 Mont-Saint-

Aignan, France.; (Leconte A) Centre régional de lutte contre le cancer François-Baclesse, 14000 Caen, France.; (Bisson C) Centre hospitalier, 14400 Bayeux, France; Réseau régional douleur en Basse-Normandie, 14400 Bayeux, France.; (Bignon R) Réseau régional douleur en Basse-Normandie, 14400 Bayeux, France; Centre hospitalier, 14100 Lisieux, France.; (Cauchin S) Réseau régional douleur en Basse-Normandie, 14400 Bayeux, France; Centre hospitalier intercommunal Alençon-Mamers, 61000 Alençon, France.; (Feuillet M) Réseau régional douleur en Basse-Normandie, 14400 Bayeux, France; Centre hospitalier, 50000 Saint-Lô, France.; (Gehanne S) Réseau régional douleur en Basse-Normandie, 14400 Bayeux, France; Centre hospitalier, 50000 Saint-Lô, France.; (Gicquère M) Centre régional de lutte contre le cancer François-Baclesse, 14000 Caen, France; Réseau régional douleur en Basse-Normandie, 14400 Bayeux, France.; (Grach MC) Centre régional de lutte contre le cancer François-Baclesse, 14000 Caen, France; Réseau régional douleur en Basse-Normandie, 14400 Bayeux, France.; (Guillaumé C) Réseau régional douleur en Basse-Normandie, 14400 Bayeux, France; CHU, 14000 Caen, France.; (Le Gal C) Réseau régional douleur en Basse-Normandie, 14400 Bayeux, France; Centre hospitalier, 61200 Argentan, France.; (Le Garrec J) Réseau régional douleur en Basse-Normandie, 14400 Bayeux, France; Centre hospitalier intercommunal Alençon-Mamers, 61000 Alençon, France.; (Lecaer F) Réseau régional douleur en Basse-Normandie, 14400 Bayeux, France; Centre hospitalier, 61100 Flers, France.; (Lepleux I) Réseau régional douleur en Basse-Normandie, 14400 Bayeux, France; Centre hospitalier, 50100 Cherbourg, France.; (Millet AL) Réseau régional douleur en Basse-Normandie, 14400 Bayeux, France; Centre hospitalier, 61100 Flers, France.; (Ropartz MC) Réseau régional douleur en Basse-Normandie, 14400 Bayeux, France; Centre hospitalier, 50400 Avranches-Granville, France.; (Roux N) Réseau régional douleur en Basse-Normandie, 14400 Bayeux, France; CHU, 14000 Caen, France.; (Sep Hieng V) Réseau régional douleur en Basse-Normandie, 14400 Bayeux, France; Centre hospitalier, 14100 Lisieux, France.; (Van Delook C) Réseau régional douleur en Basse-Normandie, 14400 Bayeux, France; Centre hospitalier, 61200 Argentan, France.; (Bechet C) Pharmacie de la Croix d'Or, 75017 Paris, France.; (Le Chevalier A) Réseau régional douleur en Basse-Normandie, 14400 Bayeux, France; Centre hospitalier, 50400 Avranches-Granville, France.; (Delorme C) Centre hospitalier, 14400 Bayeux, France; Réseau régional douleur en Basse-Normandie, 14400 Bayeux, France.

(Prevost V) Université de Caen Normandie, UMR 1086 Inserm, unité de recherche interdisciplinaire pour la prévention et le traitement des cancers « ANTICIPE », 14000 Caen, France; Centre régional de lutte contre le cancer François-Baclesse, 14000 Caen, France. Electronic address: virginie.prevost@unicaen.fr.; (Clarisse B) Centre régional de lutte contre le cancer François-Baclesse, 14000 Caen, France.; (Heutte N) Centre régional de lutte contre le cancer François-Baclesse, 14000 Caen, France; Normandie université, UNIROUEN, CETAPS EA 3832, 76130 Mont-Saint-Aignan, France.; (Leconte A) Centre régional de lutte contre le cancer François-Baclesse, 14000 Caen, France.; (Bisson C) Centre hospitalier, 14400 Bayeux, France; Réseau régional douleur en Basse-Normandie, 14400 Bayeux, France.; (Bignon R) Réseau régional douleur en Basse-Normandie, 14400 Bayeux, France; Centre hospitalier, 14100 Lisieux, France.; (Cauchin S) Réseau régional douleur en Basse-Normandie, 14400 Bayeux, France; Centre hospitalier intercommunal Alençon-Mamers, 61000 Alençon, France.; (Feuillet M) Réseau régional douleur en Basse-Normandie, 14400 Bayeux, France; Centre hospitalier, 50000 Saint-Lô, France.; (Gehanne S) Réseau régional douleur en Basse-Normandie, 14400

Bayeux, France; Centre hospitalier, 50000 Saint-Lô, France.; (Gicquère M) Centre régional de lutte contre le cancer François-Baclesse, 14000 Caen, France; Réseau régional douleur en Basse-Normandie, 14400 Bayeux, France.; (Grach MC) Centre régional de lutte contre le cancer François-Baclesse, 14000 Caen, France; Réseau régional douleur en Basse-Normandie, 14400 Bayeux, France.; (Guillaumé C) Réseau régional douleur en Basse-Normandie, 14400 Bayeux, France; CHU, 14000 Caen, France.; (Le Gal C) Réseau régional douleur en Basse-Normandie, 14400 Bayeux, France; Centre hospitalier, 61200 Argentan, France.; (Le Garrec J) Réseau régional douleur en Basse-Normandie, 14400 Bayeux, France; Centre hospitalier intercommunal Alençon-Mamers, 61000 Alençon, France.; (Lecaer F) Réseau régional douleur en Basse-Normandie, 14400 Bayeux, France; Centre hospitalier, 61100 Flers, France.; (Lepleux I) Réseau régional douleur en Basse-Normandie, 14400 Bayeux, France; Centre hospitalier, 50100 Cherbourg, France.; (Millet AL) Réseau régional douleur en Basse-Normandie, 14400 Bayeux, France; Centre hospitalier, 61100 Flers, France.; (Ropartz MC) Réseau régional douleur en Basse-Normandie, 14400 Bayeux, France; Centre hospitalier, 50400 Avranches-Granville, France.; (Roux N) Réseau régional douleur en Basse-Normandie, 14400 Bayeux, France; CHU, 14000 Caen, France.; (Sep Hieng V) Réseau régional douleur en Basse-Normandie, 14400 Bayeux, France; Centre hospitalier, 14100 Lisieux, France.; (Van Delook C) Réseau régional douleur en Basse-Normandie, 14400 Bayeux, France; Centre hospitalier, 61200 Argentan, France.; (Bechet C) Pharmacie de la Croix d'Or, 75017 Paris, France.; (Le Chevalier A) Réseau régional douleur en Basse-Normandie, 14400 Bayeux, France; Centre hospitalier, 50400 Avranches-Granville, France.; (Delorme C) Centre hospitalier, 14400 Bayeux, France; Réseau régional douleur en Basse-Normandie, 14400 Bayeux, France.
(Prevost V) Université de Caen Normandie, UMR 1086 Inserm, unité de recherche interdisciplinaire pour la prévention et le traitement des cancers « ANTICIPE », 14000 Caen, France; Centre régional de lutte contre le cancer François-Baclesse, 14000 Caen, France. Electronic address: virginie.prevost@unicaen.fr.; (Clarisse B) Centre régional de lutte contre le cancer François-Baclesse, 14000 Caen, France.; (Heutte N) Centre régional de lutte contre le cancer François-Baclesse, 14000 Caen, France; Normandie université, UNIROUEN, CETAPS EA 3832, 76130 Mont-Saint-Aignan, France.; (Leconte A) Centre régional de lutte contre le cancer François-Baclesse, 14000 Caen, France.; (Bisson C) Centre hospitalier, 14400 Bayeux, France; Réseau régional douleur en Basse-Normandie, 14400 Bayeux, France.; (Bignon R) Réseau régional douleur en Basse-Normandie, 14400 Bayeux, France; Centre hospitalier, 14100 Lisieux, France.; (Cauchin S) Réseau régional douleur en Basse-Normandie, 14400 Bayeux, France; Centre hospitalier intercommunal Alençon-Mamers, 61000 Alençon, France.; (Feuillet M) Réseau régional douleur en Basse-Normandie, 14400 Bayeux, France; Centre hospitalier, 50000 Saint-Lô, France.; (Gehanne S) Réseau régional douleur en Basse-Normandie, 14400 Bayeux, France; Centre hospitalier, 50000 Saint-Lô, France.; (Gicquère M) Centre régional de lutte contre le cancer François-Baclesse, 14000 Caen, France; Réseau régional douleur en Basse-Normandie, 14400 Bayeux, France.; (Grach MC) Centre régional de lutte contre le cancer François-Baclesse, 14000 Caen, France; Réseau régional douleur en Basse-Normandie, 14400 Bayeux, France.; (Guillaumé C) Réseau régional douleur en Basse-Normandie, 14400 Bayeux, France; CHU, 14000 Caen, France.; (Le Gal C) Réseau régional douleur en Basse-Normandie, 14400 Bayeux, France; Centre hospitalier, 61200 Argentan, France.; (Le Garrec J) Réseau régional douleur en Basse-Normandie, 14400 Bayeux, France; Centre hospitalier

intercommunal Alençon-Mamers, 61000 Alençon, France.; (Lecaer F) Réseau régional douleur en Basse-Normandie, 14400 Bayeux, France; Centre hospitalier, 61100 Flers, France.; (Lepleux I) Réseau régional douleur en Basse-Normandie, 14400 Bayeux, France; Centre hospitalier, 50100 Cherbourg, France.; (Millet AL) Réseau régional douleur en Basse-Normandie, 14400 Bayeux, France; Centre hospitalier, 61100 Flers, France.; (Ropartz MC) Réseau régional douleur en Basse-Normandie, 14400 Bayeux, France; Centre hospitalier, 50400 Avranches-Granville, France.; (Roux N) Réseau régional douleur en Basse-Normandie, 14400 Bayeux, France; CHU, 14000 Caen, France.; (Sep Hieng V) Réseau régional douleur en Basse-Normandie, 14400 Bayeux, France; Centre hospitalier, 14100 Lisieux, France.; (Van Delook C) Réseau régional douleur en Basse-Normandie, 14400 Bayeux, France; Centre hospitalier, 61200 Argentan, France.; (Bechet C) Pharmacie de la Croix d'Or, 75017 Paris, France.; (Le Chevalier A) Réseau régional douleur en Basse-Normandie, 14400 Bayeux, France; Centre hospitalier, 50400 Avranches-Granville, France.; (Delorme C) Centre hospitalier, 14400 Bayeux, France; Réseau régional douleur en Basse-Normandie, 14400 Bayeux, France.

(Prevost V) Université de Caen Normandie, UMR 1086 Inserm, unité de recherche interdisciplinaire pour la prévention et le traitement des cancers « ANTICIPE », 14000 Caen, France; Centre régional de lutte contre le cancer François-Baclesse, 14000 Caen, France. Electronic address: virginie.prevost@unicaen.fr.; (Clarisse B) Centre régional de lutte contre le cancer François-Baclesse, 14000 Caen, France.; (Heutte N) Centre régional de lutte contre le cancer François-Baclesse, 14000 Caen, France; Normandie université, UNIROUEN, CETAPS EA 3832, 76130 Mont-Saint-Aignan, France.; (Leconte A) Centre régional de lutte contre le cancer François-Baclesse, 14000 Caen, France.; (Bisson C) Centre hospitalier, 14400 Bayeux, France; Réseau régional douleur en Basse-Normandie, 14400 Bayeux, France.; (Bignon R) Réseau régional douleur en Basse-Normandie, 14400 Bayeux, France; Centre hospitalier, 14100 Lisieux, France.; (Cauchin S) Réseau régional douleur en Basse-Normandie, 14400 Bayeux, France; Centre hospitalier intercommunal Alençon-Mamers, 61000 Alençon, France.; (Feuillet M) Réseau régional douleur en Basse-Normandie, 14400 Bayeux, France; Centre hospitalier, 50000 Saint-Lô, France.; (Gehanne S) Réseau régional douleur en Basse-Normandie, 14400 Bayeux, France; Centre hospitalier, 50000 Saint-Lô, France.; (Gicquère M) Centre régional de lutte contre le cancer François-Baclesse, 14000 Caen, France; Réseau régional douleur en Basse-Normandie, 14400 Bayeux, France.; (Grach MC) Centre régional de lutte contre le cancer François-Baclesse, 14000 Caen, France; Réseau régional douleur en Basse-Normandie, 14400 Bayeux, France.; (Guillaumé C) Réseau régional douleur en Basse-Normandie, 14400 Bayeux, France; CHU, 14000 Caen, France.; (Le Gal C) Réseau régional douleur en Basse-Normandie, 14400 Bayeux, France; Centre hospitalier, 61200 Argentan, France.; (Le Garrec J) Réseau régional douleur en Basse-Normandie, 14400 Bayeux, France; Centre hospitalier intercommunal Alençon-Mamers, 61000 Alençon, France.; (Lecaer F) Réseau régional douleur en Basse-Normandie, 14400 Bayeux, France; Centre hospitalier, 61100 Flers, France.; (Lepleux I) Réseau régional douleur en Basse-Normandie, 14400 Bayeux, France; Centre hospitalier, 50100 Cherbourg, France.; (Millet AL) Réseau régional douleur en Basse-Normandie, 14400 Bayeux, France; Centre hospitalier, 61100 Flers, France.; (Ropartz MC) Réseau régional douleur en Basse-Normandie, 14400 Bayeux, France; Centre hospitalier, 50400 Avranches-Granville, France.; (Roux N) Réseau régional douleur en Basse-Normandie, 14400 Bayeux, France; CHU, 14000 Caen, France.; (Sep Hieng V) Réseau régional douleur en

Basse-Normandie, 14400 Bayeux, France; Centre hospitalier, 14100 Lisieux, France.; (Van Delook C) Réseau régional douleur en Basse-Normandie, 14400 Bayeux, France; Centre hospitalier, 61200 Argentan, France.; (Bechet C) Pharmacie de la Croix d'Or, 75017 Paris, France.; (Le Chevalier A) Réseau régional douleur en Basse-Normandie, 14400 Bayeux, France; Centre hospitalier, 50400 Avranches-Granville, France.; (Delorme C) Centre hospitalier, 14400 Bayeux, France; Réseau régional douleur en Basse-Normandie, 14400 Bayeux, France.
(Prevost V) Université de Caen Normandie, UMR 1086 Inserm, unité de recherche interdisciplinaire pour la prévention et le traitement des cancers « ANTICIPE », 14000 Caen, France; Centre régional de lutte contre le cancer François-Baclesse, 14000 Caen, France. Electronic address: virginie.prevost@unicaen.fr.; (Clarisse B) Centre régional de lutte contre le cancer François-Baclesse, 14000 Caen, France.; (Heutte N) Centre régional de lutte contre le cancer François-Baclesse, 14000 Caen, France; Normandie université, UNIROUEN, CETAPS EA 3832, 76130 Mont-Saint-Aignan, France.; (Leconte A) Centre régional de lutte contre le cancer François-Baclesse, 14000 Caen, France.; (Bisson C) Centre hospitalier, 14400 Bayeux, France; Réseau régional douleur en Basse-Normandie, 14400 Bayeux, France.; (Bignon R) Réseau régional douleur en Basse-Normandie, 14400 Bayeux, France; Centre hospitalier, 14100 Lisieux, France.; (Cauchin S) Réseau régional douleur en Basse-Normandie, 14400 Bayeux, France; Centre hospitalier intercommunal Alençon-Mamers, 61000 Alençon, France.; (Feuillet M) Réseau régional douleur en Basse-Normandie, 14400 Bayeux, France; Centre hospitalier, 50000 Saint-Lô, France.; (Gehanne S) Réseau régional douleur en Basse-Normandie, 14400 Bayeux, France; Centre hospitalier, 50000 Saint-Lô, France.; (Gicquère M) Centre régional de lutte contre le cancer François-Baclesse, 14000 Caen, France; Réseau régional douleur en Basse-Normandie, 14400 Bayeux, France.; (Grach MC) Centre régional de lutte contre le cancer François-Baclesse, 14000 Caen, France; Réseau régional douleur en Basse-Normandie, 14400 Bayeux, France.; (Guillaumé C) Réseau régional douleur en Basse-Normandie, 14400 Bayeux, France; CHU, 14000 Caen, France.; (Le Gal C) Réseau régional douleur en Basse-Normandie, 14400 Bayeux, France; Centre hospitalier, 61200 Argentan, France.; (Le Garrec J) Réseau régional douleur en Basse-Normandie, 14400 Bayeux, France; Centre hospitalier intercommunal Alençon-Mamers, 61000 Alençon, France.; (Lecaer F) Réseau régional douleur en Basse-Normandie, 14400 Bayeux, France; Centre hospitalier, 61100 Flers, France.; (Lepleux I) Réseau régional douleur en Basse-Normandie, 14400 Bayeux, France; Centre hospitalier, 50100 Cherbourg, France.; (Millet AL) Réseau régional douleur en Basse-Normandie, 14400 Bayeux, France; Centre hospitalier, 61100 Flers, France.; (Ropartz MC) Réseau régional douleur en Basse-Normandie, 14400 Bayeux, France; Centre hospitalier, 50400 Avranches-Granville, France.; (Roux N) Réseau régional douleur en Basse-Normandie, 14400 Bayeux, France; CHU, 14000 Caen, France.; (Sep Hieng V) Réseau régional douleur en Basse-Normandie, 14400 Bayeux, France; Centre hospitalier, 14100 Lisieux, France.; (Van Delook C) Réseau régional douleur en Basse-Normandie, 14400 Bayeux, France; Centre hospitalier, 61200 Argentan, France.; (Bechet C) Pharmacie de la Croix d'Or, 75017 Paris, France.; (Le Chevalier A) Réseau régional douleur en Basse-Normandie, 14400 Bayeux, France; Centre hospitalier, 50400 Avranches-Granville, France.; (Delorme C) Centre hospitalier, 14400 Bayeux, France; Réseau régional douleur en Basse-Normandie, 14400 Bayeux, France.
(Prevost V) Université de Caen Normandie, UMR 1086 Inserm, unité de recherche interdisciplinaire pour la prévention et le traitement des cancers « ANTICIPE »,

14000 Caen, France; Centre régional de lutte contre le cancer François-Baclesse, 14000 Caen, France. Electronic address: virginie.prevost@unicaen.fr.; (Clarisse B) Centre régional de lutte contre le cancer François-Baclesse, 14000 Caen, France.; (Heutte N) Centre régional de lutte contre le cancer François-Baclesse, 14000 Caen, France; Normandie université, UNIROUEN, CETAPS EA 3832, 76130 Mont-Saint-Aignan, France.; (Leconte A) Centre régional de lutte contre le cancer François-Baclesse, 14000 Caen, France.; (Bisson C) Centre hospitalier, 14400 Bayeux, France; Réseau régional douleur en Basse-Normandie, 14400 Bayeux, France.; (Bignon R) Réseau régional douleur en Basse-Normandie, 14400 Bayeux, France; Centre hospitalier, 14100 Lisieux, France.; (Cauchin S) Réseau régional douleur en Basse-Normandie, 14400 Bayeux, France; Centre hospitalier intercommunal Alençon-Mamers, 61000 Alençon, France.; (Feuillet M) Réseau régional douleur en Basse-Normandie, 14400 Bayeux, France; Centre hospitalier, 50000 Saint-Lô, France.; (Gehanne S) Réseau régional douleur en Basse-Normandie, 14400 Bayeux, France; Centre hospitalier, 50000 Saint-Lô, France.; (Gicquère M) Centre régional de lutte contre le cancer François-Baclesse, 14000 Caen, France; Réseau régional douleur en Basse-Normandie, 14400 Bayeux, France.; (Grach MC) Centre régional de lutte contre le cancer François-Baclesse, 14000 Caen, France; Réseau régional douleur en Basse-Normandie, 14400 Bayeux, France.; (Guillaumé C) Réseau régional douleur en Basse-Normandie, 14400 Bayeux, France; CHU, 14000 Caen, France.; (Le Gal C) Réseau régional douleur en Basse-Normandie, 14400 Bayeux, France; Centre hospitalier, 61200 Argentan, France.; (Le Garrec J) Réseau régional douleur en Basse-Normandie, 14400 Bayeux, France; Centre hospitalier intercommunal Alençon-Mamers, 61000 Alençon, France.; (Lecaer F) Réseau régional douleur en Basse-Normandie, 14400 Bayeux, France; Centre hospitalier, 61100 Flers, France.; (Lepleux I) Réseau régional douleur en Basse-Normandie, 14400 Bayeux, France; Centre hospitalier, 50100 Cherbourg, France.; (Millet AL) Réseau régional douleur en Basse-Normandie, 14400 Bayeux, France; Centre hospitalier, 61100 Flers, France.; (Ropartz MC) Réseau régional douleur en Basse-Normandie, 14400 Bayeux, France; Centre hospitalier, 50400 Avranches-Granville, France.; (Roux N) Réseau régional douleur en Basse-Normandie, 14400 Bayeux, France; CHU, 14000 Caen, France.; (Sep Hieng V) Réseau régional douleur en Basse-Normandie, 14400 Bayeux, France; Centre hospitalier, 14100 Lisieux, France.; (Van Delook C) Réseau régional douleur en Basse-Normandie, 14400 Bayeux, France; Centre hospitalier, 61200 Argentan, France.; (Bechet C) Pharmacie de la Croix d'Or, 75017 Paris, France.; (Le Chevalier A) Réseau régional douleur en Basse-Normandie, 14400 Bayeux, France; Centre hospitalier, 50400 Avranches-Granville, France.; (Delorme C) Centre hospitalier, 14400 Bayeux, France; Réseau régional douleur en Basse-Normandie, 14400 Bayeux, France.
(Prevost V) Université de Caen Normandie, UMR 1086 Inserm, unité de recherche interdisciplinaire pour la prévention et le traitement des cancers « ANTICIPE »,
14000 Caen, France; Centre régional de lutte contre le cancer François-Baclesse, 14000 Caen, France. Electronic address: virginie.prevost@unicaen.fr.; (Clarisse B) Centre régional de lutte contre le cancer François-Baclesse, 14000 Caen, France.; (Heutte N) Centre régional de lutte contre le cancer François-Baclesse, 14000 Caen, France; Normandie université, UNIROUEN, CETAPS EA 3832, 76130 Mont-Saint-Aignan, France.; (Leconte A) Centre régional de lutte contre le cancer François-Baclesse, 14000 Caen, France.; (Bisson C) Centre hospitalier, 14400 Bayeux, France; Réseau régional douleur en Basse-Normandie, 14400 Bayeux, France.; (Bignon R) Réseau régional douleur en Basse-Normandie, 14400 Bayeux, France;

Centre hospitalier, 14100 Lisieux, France.; (Cauchin S) Réseau régional douleur en Basse-Normandie, 14400 Bayeux, France; Centre hospitalier intercommunal Alençon-Mamers, 61000 Alençon, France.; (Feuillet M) Réseau régional douleur en Basse-Normandie, 14400 Bayeux, France; Centre hospitalier, 50000 Saint-Lô, France.; (Gehanne S) Réseau régional douleur en Basse-Normandie, 14400 Bayeux, France; Centre hospitalier, 50000 Saint-Lô, France.; (Gicquère M) Centre régional de lutte contre le cancer François-Baclesse, 14000 Caen, France; Réseau régional douleur en Basse-Normandie, 14400 Bayeux, France.; (Grach MC) Centre régional de lutte contre le cancer François-Baclesse, 14000 Caen, France; Réseau régional douleur en Basse-Normandie, 14400 Bayeux, France.; (Guillaumé C) Réseau régional douleur en Basse-Normandie, 14400 Bayeux, France; CHU, 14000 Caen, France.; (Le Gal C) Réseau régional douleur en Basse-Normandie, 14400 Bayeux, France; Centre hospitalier, 61200 Argentan, France.; (Le Garrec J) Réseau régional douleur en Basse-Normandie, 14400 Bayeux, France; Centre hospitalier intercommunal Alençon-Mamers, 61000 Alençon, France.; (Lecaer F) Réseau régional douleur en Basse-Normandie, 14400 Bayeux, France; Centre hospitalier, 61100 Flers, France.; (Lepleux I) Réseau régional douleur en Basse-Normandie, 14400 Bayeux, France; Centre hospitalier, 50100 Cherbourg, France.; (Millet AL) Réseau régional douleur en Basse-Normandie, 14400 Bayeux, France; Centre hospitalier, 61100 Flers, France.; (Ropartz MC) Réseau régional douleur en Basse-Normandie, 14400 Bayeux, France; Centre hospitalier, 50400 Avranches-Granville, France.; (Roux N) Réseau régional douleur en Basse-Normandie, 14400 Bayeux, France; CHU, 14000 Caen, France.; (Sep Hieng V) Réseau régional douleur en Basse-Normandie, 14400 Bayeux, France; Centre hospitalier, 14100 Lisieux, France.; (Van Delook C) Réseau régional douleur en Basse-Normandie, 14400 Bayeux, France; Centre hospitalier, 61200 Argentan, France.; (Bechet C) Pharmacie de la Croix d'Or, 75017 Paris, France.; (Le Chevalier A) Réseau régional douleur en Basse-Normandie, 14400 Bayeux, France; Centre hospitalier, 50400 Avranches-Granville, France.; (Delorme C) Centre hospitalier, 14400 Bayeux, France; Réseau régional douleur en Basse-Normandie, 14400 Bayeux, France.

(Prevost V) Université de Caen Normandie, UMR 1086 Inserm, unité de recherche interdisciplinaire pour la prévention et le traitement des cancers « ANTICIPE », 14000 Caen, France; Centre régional de lutte contre le cancer François-Baclesse, 14000 Caen, France. Electronic address: virginie.prevost@unicaen.fr.; (Clarisse B) Centre régional de lutte contre le cancer François-Baclesse, 14000 Caen, France.; (Heutte N) Centre régional de lutte contre le cancer François-Baclesse, 14000 Caen, France; Normandie université, UNIROUEN, CETAPS EA 3832, 76130 Mont-Saint-Aignan, France.; (Leconte A) Centre régional de lutte contre le cancer François-Baclesse, 14000 Caen, France.; (Bisson C) Centre hospitalier, 14400 Bayeux, France; Réseau régional douleur en Basse-Normandie, 14400 Bayeux, France.; (Bignon R) Réseau régional douleur en Basse-Normandie, 14400 Bayeux, France; Centre hospitalier, 14100 Lisieux, France.; (Cauchin S) Réseau régional douleur en Basse-Normandie, 14400 Bayeux, France; Centre hospitalier intercommunal Alençon-Mamers, 61000 Alençon, France.; (Feuillet M) Réseau régional douleur en Basse-Normandie, 14400 Bayeux, France; Centre hospitalier, 50000 Saint-Lô, France.; (Gehanne S) Réseau régional douleur en Basse-Normandie, 14400 Bayeux, France; Centre hospitalier, 50000 Saint-Lô, France.; (Gicquère M) Centre régional de lutte contre le cancer François-Baclesse, 14000 Caen, France; Réseau régional douleur en Basse-Normandie, 14400 Bayeux, France.; (Grach MC) Centre régional de lutte contre le cancer François-Baclesse, 14000 Caen, France; Réseau

régional douleur en Basse-Normandie, 14400 Bayeux, France.; (Guillaumé C) Réseau régional douleur en Basse-Normandie, 14400 Bayeux, France; CHU, 14000 Caen, France.; (Le Gal C) Réseau régional douleur en Basse-Normandie, 14400 Bayeux, France; Centre hospitalier, 61200 Argentan, France.; (Le Garrec J) Réseau régional douleur en Basse-Normandie, 14400 Bayeux, France; Centre hospitalier intercommunal Alençon-Mamers, 61000 Alençon, France.; (Lecaer F) Réseau régional douleur en Basse-Normandie, 14400 Bayeux, France; Centre hospitalier, 61100 Flers, France.; (Lepleux I) Réseau régional douleur en Basse-Normandie, 14400 Bayeux, France; Centre hospitalier, 50100 Cherbourg, France.; (Millet AL) Réseau régional douleur en Basse-Normandie, 14400 Bayeux, France; Centre hospitalier, 61100 Flers, France.; (Ropartz MC) Réseau régional douleur en Basse-Normandie, 14400 Bayeux, France; Centre hospitalier, 50400 Avranches-Granville, France.; (Roux N) Réseau régional douleur en Basse-Normandie, 14400 Bayeux, France; CHU, 14000 Caen, France.; (Sep Hieng V) Réseau régional douleur en Basse-Normandie, 14400 Bayeux, France; Centre hospitalier, 14100 Lisieux, France.; (Van Delook C) Réseau régional douleur en Basse-Normandie, 14400 Bayeux, France; Centre hospitalier, 61200 Argentan, France.; (Bechet C) Pharmacie de la Croix d'Or, 75017 Paris, France.; (Le Chevalier A) Réseau régional douleur en Basse-Normandie, 14400 Bayeux, France; Centre hospitalier, 50400 Avranches-Granville, France.; (Delorme C) Centre hospitalier, 14400 Bayeux, France; Réseau régional douleur en Basse-Normandie, 14400 Bayeux, France.
(Prevost V) Université de Caen Normandie, UMR 1086 Inserm, unité de recherche interdisciplinaire pour la prévention et le traitement des cancers « ANTICIPE », 14000 Caen, France; Centre régional de lutte contre le cancer François-Baclesse, 14000 Caen, France. Electronic address: virginie.prevost@unicaen.fr.; (Clarisse B) Centre régional de lutte contre le cancer François-Baclesse, 14000 Caen, France.; (Heutte N) Centre régional de lutte contre le cancer François-Baclesse, 14000 Caen, France; Normandie université, UNIROUEN, CETAPS EA 3832, 76130 Mont-Saint-Aignan, France.; (Leconte A) Centre régional de lutte contre le cancer François-Baclesse, 14000 Caen, France.; (Bisson C) Centre hospitalier, 14400 Bayeux, France; Réseau régional douleur en Basse-Normandie, 14400 Bayeux, France.; (Bignon R) Réseau régional douleur en Basse-Normandie, 14400 Bayeux, France; Centre hospitalier, 14100 Lisieux, France.; (Cauchin S) Réseau régional douleur en Basse-Normandie, 14400 Bayeux, France; Centre hospitalier intercommunal Alençon-Mamers, 61000 Alençon, France.; (Feuillet M) Réseau régional douleur en Basse-Normandie, 14400 Bayeux, France; Centre hospitalier, 50000 Saint-Lô, France.; (Gehanne S) Réseau régional douleur en Basse-Normandie, 14400 Bayeux, France; Centre hospitalier, 50000 Saint-Lô, France.; (Gicquère M) Centre régional de lutte contre le cancer François-Baclesse, 14000 Caen, France; Réseau régional douleur en Basse-Normandie, 14400 Bayeux, France.; (Grach MC) Centre régional de lutte contre le cancer François-Baclesse, 14000 Caen, France; Réseau régional douleur en Basse-Normandie, 14400 Bayeux, France.; (Guillaumé C) Réseau régional douleur en Basse-Normandie, 14400 Bayeux, France; CHU, 14000 Caen, France.; (Le Gal C) Réseau régional douleur en Basse-Normandie, 14400 Bayeux, France; Centre hospitalier, 61200 Argentan, France.; (Le Garrec J) Réseau régional douleur en Basse-Normandie, 14400 Bayeux, France; Centre hospitalier intercommunal Alençon-Mamers, 61000 Alençon, France.; (Lecaer F) Réseau régional douleur en Basse-Normandie, 14400 Bayeux, France; Centre hospitalier, 61100 Flers, France.; (Lepleux I) Réseau régional douleur en Basse-Normandie, 14400 Bayeux, France; Centre hospitalier, 50100 Cherbourg, France.; (Millet AL)

Réseau régional douleur en Basse-Normandie, 14400 Bayeux, France; Centre hospitalier, 61100 Flers, France.; (Ropartz MC) Réseau régional douleur en Basse-Normandie, 14400 Bayeux, France; Centre hospitalier, 50400 Avranches-Granville, France.; (Roux N) Réseau régional douleur en Basse-Normandie, 14400 Bayeux, France; CHU, 14000 Caen, France.; (Sep Hieng V) Réseau régional douleur en Basse-Normandie, 14400 Bayeux, France; Centre hospitalier, 14100 Lisieux, France.; (Van Delook C) Réseau régional douleur en Basse-Normandie, 14400 Bayeux, France; Centre hospitalier, 61200 Argentan, France.; (Bechet C) Pharmacie de la Croix d'Or, 75017 Paris, France.; (Le Chevalier A) Réseau régional douleur en Basse-Normandie, 14400 Bayeux, France; Centre hospitalier, 50400 Avranches-Granville, France.; (Delorme C) Centre hospitalier, 14400 Bayeux, France; Réseau régional douleur en Basse-Normandie, 14400 Bayeux, France.
(Prevost V) Université de Caen Normandie, UMR 1086 Inserm, unité de recherche interdisciplinaire pour la prévention et le traitement des cancers « ANTICIPE », 14000 Caen, France; Centre régional de lutte contre le cancer François-Baclesse, 14000 Caen, France. Electronic address: virginie.prevost@unicaen.fr.; (Clarisse B) Centre régional de lutte contre le cancer François-Baclesse, 14000 Caen, France.; (Heutte N) Centre régional de lutte contre le cancer François-Baclesse, 14000 Caen, France; Normandie université, UNIROUEN, CETAPS EA 3832, 76130 Mont-Saint-Aignan, France.; (Leconte A) Centre régional de lutte contre le cancer François-Baclesse, 14000 Caen, France.; (Bisson C) Centre hospitalier, 14400 Bayeux, France; Réseau régional douleur en Basse-Normandie, 14400 Bayeux, France.; (Bignon R) Réseau régional douleur en Basse-Normandie, 14400 Bayeux, France; Centre hospitalier, 14100 Lisieux, France.; (Cauchin S) Réseau régional douleur en Basse-Normandie, 14400 Bayeux, France; Centre hospitalier intercommunal Alençon-Mamers, 61000 Alençon, France.; (Feuillet M) Réseau régional douleur en Basse-Normandie, 14400 Bayeux, France; Centre hospitalier, 50000 Saint-Lô, France.; (Gehanne S) Réseau régional douleur en Basse-Normandie, 14400 Bayeux, France; Centre hospitalier, 50000 Saint-Lô, France.; (Gicquère M) Centre régional de lutte contre le cancer François-Baclesse, 14000 Caen, France; Réseau régional douleur en Basse-Normandie, 14400 Bayeux, France.; (Grach MC) Centre régional de lutte contre le cancer François-Baclesse, 14000 Caen, France; Réseau régional douleur en Basse-Normandie, 14400 Bayeux, France.; (Guillaumé C) Réseau régional douleur en Basse-Normandie, 14400 Bayeux, France; CHU, 14000 Caen, France.; (Le Gal C) Réseau régional douleur en Basse-Normandie, 14400 Bayeux, France; Centre hospitalier, 61200 Argentan, France.; (Le Garrec J) Réseau régional douleur en Basse-Normandie, 14400 Bayeux, France; Centre hospitalier intercommunal Alençon-Mamers, 61000 Alençon, France.; (Lecaer F) Réseau régional douleur en Basse-Normandie, 14400 Bayeux, France; Centre hospitalier, 61100 Flers, France.; (Lepleux I) Réseau régional douleur en Basse-Normandie, 14400 Bayeux, France; Centre hospitalier, 50100 Cherbourg, France.; (Millet AL) Réseau régional douleur en Basse-Normandie, 14400 Bayeux, France; Centre hospitalier, 61100 Flers, France.; (Ropartz MC) Réseau régional douleur en Basse-Normandie, 14400 Bayeux, France; Centre hospitalier, 50400 Avranches-Granville, France.; (Roux N) Réseau régional douleur en Basse-Normandie, 14400 Bayeux, France; CHU, 14000 Caen, France.; (Sep Hieng V) Réseau régional douleur en Basse-Normandie, 14400 Bayeux, France; Centre hospitalier, 14100 Lisieux, France.; (Van Delook C) Réseau régional douleur en Basse-Normandie, 14400 Bayeux, France; Centre hospitalier, 61200 Argentan, France.; (Bechet C) Pharmacie de la Croix d'Or, 75017 Paris, France.; (Le Chevalier A) Réseau régional douleur en

Basse-Normandie, 14400 Bayeux, France; Centre hospitalier, 50400 Avranches-Granville, France.; (Delorme C) Centre hospitalier, 14400 Bayeux, France; Réseau régional douleur en Basse-Normandie, 14400 Bayeux, France.
(Prevost V) Université de Caen Normandie, UMR 1086 Inserm, unité de recherche interdisciplinaire pour la prévention et le traitement des cancers « ANTICIPE », 14000 Caen, France; Centre régional de lutte contre le cancer François-Baclesse, 14000 Caen, France. Electronic address: virginie.prevost@unicaen.fr.; (Clarisse B) Centre régional de lutte contre le cancer François-Baclesse, 14000 Caen, France.; (Heutte N) Centre régional de lutte contre le cancer François-Baclesse, 14000 Caen, France; Normandie université, UNIROUEN, CETAPS EA 3832, 76130 Mont-Saint-Aignan, France.; (Leconte A) Centre régional de lutte contre le cancer François-Baclesse, 14000 Caen, France.; (Bisson C) Centre hospitalier, 14400 Bayeux, France; Réseau régional douleur en Basse-Normandie, 14400 Bayeux, France.; (Bignon R) Réseau régional douleur en Basse-Normandie, 14400 Bayeux, France; Centre hospitalier, 14100 Lisieux, France.; (Cauchin S) Réseau régional douleur en Basse-Normandie, 14400 Bayeux, France; Centre hospitalier intercommunal Alençon-Mamers, 61000 Alençon, France.; (Feuillet M) Réseau régional douleur en Basse-Normandie, 14400 Bayeux, France; Centre hospitalier, 50000 Saint-Lô, France.; (Gehanne S) Réseau régional douleur en Basse-Normandie, 14400 Bayeux, France; Centre hospitalier, 50000 Saint-Lô, France.; (Gicquère M) Centre régional de lutte contre le cancer François-Baclesse, 14000 Caen, France; Réseau régional douleur en Basse-Normandie, 14400 Bayeux, France.; (Grach MC) Centre régional de lutte contre le cancer François-Baclesse, 14000 Caen, France; Réseau régional douleur en Basse-Normandie, 14400 Bayeux, France.; (Guillaumé C) Réseau régional douleur en Basse-Normandie, 14400 Bayeux, France; CHU, 14000 Caen, France.; (Le Gal C) Réseau régional douleur en Basse-Normandie, 14400 Bayeux, France; Centre hospitalier, 61200 Argentan, France.; (Le Garrec J) Réseau régional douleur en Basse-Normandie, 14400 Bayeux, France; Centre hospitalier intercommunal Alençon-Mamers, 61000 Alençon, France.; (Lecaer F) Réseau régional douleur en Basse-Normandie, 14400 Bayeux, France; Centre hospitalier, 61100 Flers, France.; (Lepleux I) Réseau régional douleur en Basse-Normandie, 14400 Bayeux, France; Centre hospitalier, 50100 Cherbourg, France.; (Millet AL) Réseau régional douleur en Basse-Normandie, 14400 Bayeux, France; Centre hospitalier, 61100 Flers, France.; (Ropartz MC) Réseau régional douleur en Basse-Normandie, 14400 Bayeux, France; Centre hospitalier, 50400 Avranches-Granville, France.; (Roux N) Réseau régional douleur en Basse-Normandie, 14400 Bayeux, France; CHU, 14000 Caen, France.; (Sep Hieng V) Réseau régional douleur en Basse-Normandie, 14400 Bayeux, France; Centre hospitalier, 14100 Lisieux, France.; (Van Delook C) Réseau régional douleur en Basse-Normandie, 14400 Bayeux, France; Centre hospitalier, 61200 Argentan, France.; (Bechet C) Pharmacie de la Croix d'Or, 75017 Paris, France.; (Le Chevalier A) Réseau régional douleur en Basse-Normandie, 14400 Bayeux, France; Centre hospitalier, 50400 Avranches-Granville, France.; (Delorme C) Centre hospitalier, 14400 Bayeux, France; Réseau régional douleur en Basse-Normandie, 14400 Bayeux, France.
(Prevost V) Université de Caen Normandie, UMR 1086 Inserm, unité de recherche interdisciplinaire pour la prévention et le traitement des cancers « ANTICIPE », 14000 Caen, France; Centre régional de lutte contre le cancer François-Baclesse, 14000 Caen, France. Electronic address: virginie.prevost@unicaen.fr.; (Clarisse B) Centre régional de lutte contre le cancer François-Baclesse, 14000 Caen, France.; (Heutte N) Centre régional de lutte contre le cancer François-Baclesse, 14000 Caen,

France; Normandie université, UNIROUEN, CETAPS EA 3832, 76130 Mont-Saint-Aignan, France.; (Leconte A) Centre régional de lutte contre le cancer François-Baclesse, 14000 Caen, France.; (Bisson C) Centre hospitalier, 14400 Bayeux, France; Réseau régional douleur en Basse-Normandie, 14400 Bayeux, France.; (Bignon R) Réseau régional douleur en Basse-Normandie, 14400 Bayeux, France; Centre hospitalier, 14100 Lisieux, France.; (Cauchin S) Réseau régional douleur en Basse-Normandie, 14400 Bayeux, France; Centre hospitalier intercommunal Alençon-Mamers, 61000 Alençon, France.; (Feuillet M) Réseau régional douleur en Basse-Normandie, 14400 Bayeux, France; Centre hospitalier, 50000 Saint-Lô, France.; (Gehanne S) Réseau régional douleur en Basse-Normandie, 14400 Bayeux, France; Centre hospitalier, 50000 Saint-Lô, France.; (Gicquère M) Centre régional de lutte contre le cancer François-Baclesse, 14000 Caen, France; Réseau régional douleur en Basse-Normandie, 14400 Bayeux, France.; (Grach MC) Centre régional de lutte contre le cancer François-Baclesse, 14000 Caen, France; Réseau régional douleur en Basse-Normandie, 14400 Bayeux, France.; (Guillaumé C) Réseau régional douleur en Basse-Normandie, 14400 Bayeux, France; CHU, 14000 Caen, France.; (Le Gal C) Réseau régional douleur en Basse-Normandie, 14400 Bayeux, France; Centre hospitalier, 61200 Argentan, France.; (Le Garrec J) Réseau régional douleur en Basse-Normandie, 14400 Bayeux, France; Centre hospitalier intercommunal Alençon-Mamers, 61000 Alençon, France.; (Lecaer F) Réseau régional douleur en Basse-Normandie, 14400 Bayeux, France; Centre hospitalier, 61100 Flers, France.; (Lepleux I) Réseau régional douleur en Basse-Normandie, 14400 Bayeux, France; Centre hospitalier, 50100 Cherbourg, France.; (Millet AL) Réseau régional douleur en Basse-Normandie, 14400 Bayeux, France; Centre hospitalier, 61100 Flers, France.; (Ropartz MC) Réseau régional douleur en Basse-Normandie, 14400 Bayeux, France; Centre hospitalier, 50400 Avranches-Granville, France.; (Roux N) Réseau régional douleur en Basse-Normandie, 14400 Bayeux, France; CHU, 14000 Caen, France.; (Sep Hieng V) Réseau régional douleur en Basse-Normandie, 14400 Bayeux, France; Centre hospitalier, 14100 Lisieux, France.; (Van Delook C) Réseau régional douleur en Basse-Normandie, 14400 Bayeux, France; Centre hospitalier, 61200 Argentan, France.; (Bechet C) Pharmacie de la Croix d'Or, 75017 Paris, France.; (Le Chevalier A) Réseau régional douleur en Basse-Normandie, 14400 Bayeux, France; Centre hospitalier, 50400 Avranches-Granville, France.; (Delorme C) Centre hospitalier, 14400 Bayeux, France; Réseau régional douleur en Basse-Normandie, 14400 Bayeux, France.
(Prevost V) Université de Caen Normandie, UMR 1086 Inserm, unité de recherche interdisciplinaire pour la prévention et le traitement des cancers « ANTICIPE », 14000 Caen, France; Centre régional de lutte contre le cancer François-Baclesse, 14000 Caen, France. Electronic address: virginie.prevost@unicaen.fr.; (Clarisse B) Centre régional de lutte contre le cancer François-Baclesse, 14000 Caen, France.; (Heutte N) Centre régional de lutte contre le cancer François-Baclesse, 14000 Caen, France; Normandie université, UNIROUEN, CETAPS EA 3832, 76130 Mont-Saint-Aignan, France.; (Leconte A) Centre régional de lutte contre le cancer François-Baclesse, 14000 Caen, France.; (Bisson C) Centre hospitalier, 14400 Bayeux, France; Réseau régional douleur en Basse-Normandie, 14400 Bayeux, France.; (Bignon R) Réseau régional douleur en Basse-Normandie, 14400 Bayeux, France; Centre hospitalier, 14100 Lisieux, France.; (Cauchin S) Réseau régional douleur en Basse-Normandie, 14400 Bayeux, France; Centre hospitalier intercommunal Alençon-Mamers, 61000 Alençon, France.; (Feuillet M) Réseau régional douleur en Basse-Normandie, 14400 Bayeux, France; Centre hospitalier, 50000 Saint-Lô,

France.; (Gehanne S) Réseau régional douleur en Basse-Normandie, 14400 Bayeux, France; Centre hospitalier, 50000 Saint-Lô, France.; (Gicquère M) Centre régional de lutte contre le cancer François-Baclesse, 14000 Caen, France; Réseau régional douleur en Basse-Normandie, 14400 Bayeux, France.; (Grach MC) Centre régional de lutte contre le cancer François-Baclesse, 14000 Caen, France; Réseau régional douleur en Basse-Normandie, 14400 Bayeux, France.; (Guillaumé C) Réseau régional douleur en Basse-Normandie, 14400 Bayeux, France; CHU, 14000 Caen, France.; (Le Gal C) Réseau régional douleur en Basse-Normandie, 14400 Bayeux, France; Centre hospitalier, 61200 Argentan, France.; (Le Garrec J) Réseau régional douleur en Basse-Normandie, 14400 Bayeux, France; Centre hospitalier intercommunal Alençon-Mamers, 61000 Alençon, France.; (Lecaer F) Réseau régional douleur en Basse-Normandie, 14400 Bayeux, France; Centre hospitalier, 61100 Flers, France.; (Lepleux I) Réseau régional douleur en Basse-Normandie, 14400 Bayeux, France; Centre hospitalier, 50100 Cherbourg, France.; (Millet AL) Réseau régional douleur en Basse-Normandie, 14400 Bayeux, France; Centre hospitalier, 61100 Flers, France.; (Ropartz MC) Réseau régional douleur en Basse-Normandie, 14400 Bayeux, France; Centre hospitalier, 50400 Avranches-Granville, France.; (Roux N) Réseau régional douleur en Basse-Normandie, 14400 Bayeux, France; CHU, 14000 Caen, France.; (Sep Hieng V) Réseau régional douleur en Basse-Normandie, 14400 Bayeux, France; Centre hospitalier, 14100 Lisieux, France.; (Van Delook C) Réseau régional douleur en Basse-Normandie, 14400 Bayeux, France; Centre hospitalier, 61200 Argentan, France.; (Bechet C) Pharmacie de la Croix d'Or, 75017 Paris, France.; (Le Chevalier A) Réseau régional douleur en Basse-Normandie, 14400 Bayeux, France; Centre hospitalier, 50400 Avranches-Granville, France.; (Delorme C) Centre hospitalier, 14400 Bayeux, France; Réseau régional douleur en Basse-Normandie, 14400 Bayeux, France.
(Prevost V) Université de Caen Normandie, UMR 1086 Inserm, unité de recherche interdisciplinaire pour la prévention et le traitement des cancers « ANTICIPE », 14000 Caen, France; Centre régional de lutte contre le cancer François-Baclesse, 14000 Caen, France. Electronic address: virginie.prevost@unicaen.fr.; (Clarisse B) Centre régional de lutte contre le cancer François-Baclesse, 14000 Caen, France.; (Heutte N) Centre régional de lutte contre le cancer François-Baclesse, 14000 Caen, France; Normandie université, UNIROUEN, CETAPS EA 3832, 76130 Mont-Saint-Aignan, France.; (Leconte A) Centre régional de lutte contre le cancer François-Baclesse, 14000 Caen, France.; (Bisson C) Centre hospitalier, 14400 Bayeux, France; Réseau régional douleur en Basse-Normandie, 14400 Bayeux, France.; (Bignon R) Réseau régional douleur en Basse-Normandie, 14400 Bayeux, France; Centre hospitalier, 14100 Lisieux, France.; (Cauchin S) Réseau régional douleur en Basse-Normandie, 14400 Bayeux, France; Centre hospitalier intercommunal Alençon-Mamers, 61000 Alençon, France.; (Feuillet M) Réseau régional douleur en Basse-Normandie, 14400 Bayeux, France; Centre hospitalier, 50000 Saint-Lô, France.; (Gehanne S) Réseau régional douleur en Basse-Normandie, 14400 Bayeux, France; Centre hospitalier, 50000 Saint-Lô, France.; (Gicquère M) Centre régional de lutte contre le cancer François-Baclesse, 14000 Caen, France; Réseau régional douleur en Basse-Normandie, 14400 Bayeux, France.; (Grach MC) Centre régional de lutte contre le cancer François-Baclesse, 14000 Caen, France; Réseau régional douleur en Basse-Normandie, 14400 Bayeux, France.; (Guillaumé C) Réseau régional douleur en Basse-Normandie, 14400 Bayeux, France; CHU, 14000 Caen, France.; (Le Gal C) Réseau régional douleur en Basse-Normandie, 14400 Bayeux, France; Centre hospitalier, 61200 Argentan, France.; (Le Garrec J) Réseau

régional douleur en Basse-Normandie, 14400 Bayeux, France; Centre hospitalier intercommunal Alençon-Mamers, 61000 Alençon, France.; (Lecaer F) Réseau régional douleur en Basse-Normandie, 14400 Bayeux, France; Centre hospitalier, 61100 Flers, France.; (Lepleux I) Réseau régional douleur en Basse-Normandie, 14400 Bayeux, France; Centre hospitalier, 50100 Cherbourg, France.; (Millet AL) Réseau régional douleur en Basse-Normandie, 14400 Bayeux, France; Centre hospitalier, 61100 Flers, France.; (Ropartz MC) Réseau régional douleur en Basse-Normandie, 14400 Bayeux, France; Centre hospitalier, 50400 Avranches-Granville, France.; (Roux N) Réseau régional douleur en Basse-Normandie, 14400 Bayeux, France; CHU, 14000 Caen, France.; (Sep Hieng V) Réseau régional douleur en Basse-Normandie, 14400 Bayeux, France; Centre hospitalier, 14100 Lisieux, France.; (Van Delook C) Réseau régional douleur en Basse-Normandie, 14400 Bayeux, France; Centre hospitalier, 61200 Argentan, France.; (Bechet C) Pharmacie de la Croix d'Or, 75017 Paris, France.; (Le Chevalier A) Réseau régional douleur en Basse-Normandie, 14400 Bayeux, France; Centre hospitalier, 50400 Avranches-Granville, France.; (Delorme C) Centre hospitalier, 14400 Bayeux, France; Réseau régional douleur en Basse-Normandie, 14400 Bayeux, France.
(Prevost V) Université de Caen Normandie, UMR 1086 Inserm, unité de recherche interdisciplinaire pour la prévention et le traitement des cancers « ANTICIPE », 14000 Caen, France; Centre régional de lutte contre le cancer François-Baclesse, 14000 Caen, France. Electronic address: virginie.prevost@unicaen.fr.; (Clarisse B) Centre régional de lutte contre le cancer François-Baclesse, 14000 Caen, France.; (Heutte N) Centre régional de lutte contre le cancer François-Baclesse, 14000 Caen, France; Normandie université, UNIROUEN, CETAPS EA 3832, 76130 Mont-Saint-Aignan, France.; (Leconte A) Centre régional de lutte contre le cancer François-Baclesse, 14000 Caen, France.; (Bisson C) Centre hospitalier, 14400 Bayeux, France; Réseau régional douleur en Basse-Normandie, 14400 Bayeux, France.; (Bignon R) Réseau régional douleur en Basse-Normandie, 14400 Bayeux, France; Centre hospitalier, 14100 Lisieux, France.; (Cauchin S) Réseau régional douleur en Basse-Normandie, 14400 Bayeux, France; Centre hospitalier intercommunal Alençon-Mamers, 61000 Alençon, France.; (Feuillet M) Réseau régional douleur en Basse-Normandie, 14400 Bayeux, France; Centre hospitalier, 50000 Saint-Lô, France.; (Gehanne S) Réseau régional douleur en Basse-Normandie, 14400 Bayeux, France; Centre hospitalier, 50000 Saint-Lô, France.; (Gicquère M) Centre régional de lutte contre le cancer François-Baclesse, 14000 Caen, France; Réseau régional douleur en Basse-Normandie, 14400 Bayeux, France.; (Grach MC) Centre régional de lutte contre le cancer François-Baclesse, 14000 Caen, France; Réseau régional douleur en Basse-Normandie, 14400 Bayeux, France.; (Guillaumé C) Réseau régional douleur en Basse-Normandie, 14400 Bayeux, France; CHU, 14000 Caen, France.; (Le Gal C) Réseau régional douleur en Basse-Normandie, 14400 Bayeux, France; Centre hospitalier, 61200 Argentan, France.; (Le Garrec J) Réseau régional douleur en Basse-Normandie, 14400 Bayeux, France; Centre hospitalier intercommunal Alençon-Mamers, 61000 Alençon, France.; (Lecaer F) Réseau régional douleur en Basse-Normandie, 14400 Bayeux, France; Centre hospitalier, 61100 Flers, France.; (Lepleux I) Réseau régional douleur en Basse-Normandie, 14400 Bayeux, France; Centre hospitalier, 50100 Cherbourg, France.; (Millet AL) Réseau régional douleur en Basse-Normandie, 14400 Bayeux, France; Centre hospitalier, 61100 Flers, France.; (Ropartz MC) Réseau régional douleur en Basse-Normandie, 14400 Bayeux, France; Centre hospitalier, 50400 Avranches-Granville, France.; (Roux N) Réseau régional douleur en Basse-Normandie, 14400 Bayeux,

France; CHU, 14000 Caen, France.; (Sep Hieng V) Réseau régional douleur en Basse-Normandie, 14400 Bayeux, France; Centre hospitalier, 14100 Lisieux, France.; (Van Delook C) Réseau régional douleur en Basse-Normandie, 14400 Bayeux, France; Centre hospitalier, 61200 Argentan, France.; (Bechet C) Pharmacie de la Croix d'Or, 75017 Paris, France.; (Le Chevalier A) Réseau régional douleur en Basse-Normandie, 14400 Bayeux, France; Centre hospitalier, 50400 Avranches-Granville, France.; (Delorme C) Centre hospitalier, 14400 Bayeux, France; Réseau régional douleur en Basse-Normandie, 14400 Bayeux, France.
(Prevost V) Université de Caen Normandie, UMR 1086 Inserm, unité de recherche interdisciplinaire pour la prévention et le traitement des cancers « ANTICIPE », 14000 Caen, France; Centre régional de lutte contre le cancer François-Baclesse, 14000 Caen, France. Electronic address: virginie.prevost@unicaen.fr.; (Clarisse B) Centre régional de lutte contre le cancer François-Baclesse, 14000 Caen, France.; (Heutte N) Centre régional de lutte contre le cancer François-Baclesse, 14000 Caen, France; Normandie université, UNIROUEN, CETAPS EA 3832, 76130 Mont-Saint-Aignan, France.; (Leconte A) Centre régional de lutte contre le cancer François-Baclesse, 14000 Caen, France.; (Bisson C) Centre hospitalier, 14400 Bayeux, France; Réseau régional douleur en Basse-Normandie, 14400 Bayeux, France.; (Bignon R) Réseau régional douleur en Basse-Normandie, 14400 Bayeux, France; Centre hospitalier, 14100 Lisieux, France.; (Cauchin S) Réseau régional douleur en Basse-Normandie, 14400 Bayeux, France; Centre hospitalier intercommunal Alençon-Mamers, 61000 Alençon, France.; (Feuillet M) Réseau régional douleur en Basse-Normandie, 14400 Bayeux, France; Centre hospitalier, 50000 Saint-Lô, France.; (Gehanne S) Réseau régional douleur en Basse-Normandie, 14400 Bayeux, France; Centre hospitalier, 50000 Saint-Lô, France.; (Gicquère M) Centre régional de lutte contre le cancer François-Baclesse, 14000 Caen, France; Réseau régional douleur en Basse-Normandie, 14400 Bayeux, France.; (Grach MC) Centre régional de lutte contre le cancer François-Baclesse, 14000 Caen, France; Réseau régional douleur en Basse-Normandie, 14400 Bayeux, France.; (Guillaumé C) Réseau régional douleur en Basse-Normandie, 14400 Bayeux, France; CHU, 14000 Caen, France.; (Le Gal C) Réseau régional douleur en Basse-Normandie, 14400 Bayeux, France; Centre hospitalier, 61200 Argentan, France.; (Le Garrec J) Réseau régional douleur en Basse-Normandie, 14400 Bayeux, France; Centre hospitalier intercommunal Alençon-Mamers, 61000 Alençon, France.; (Lecaer F) Réseau régional douleur en Basse-Normandie, 14400 Bayeux, France; Centre hospitalier, 61100 Flers, France.; (Lepleux I) Réseau régional douleur en Basse-Normandie, 14400 Bayeux, France; Centre hospitalier, 50100 Cherbourg, France.; (Millet AL) Réseau régional douleur en Basse-Normandie, 14400 Bayeux, France; Centre hospitalier, 61100 Flers, France.; (Ropartz MC) Réseau régional douleur en Basse-Normandie, 14400 Bayeux, France; Centre hospitalier, 50400 Avranches-Granville, France.; (Roux N) Réseau régional douleur en Basse-Normandie, 14400 Bayeux,
France; CHU, 14000 Caen, France.; (Sep Hieng V) Réseau régional douleur en Basse-Normandie, 14400 Bayeux, France; Centre hospitalier, 14100 Lisieux, France.; (Van Delook C) Réseau régional douleur en Basse-Normandie, 14400 Bayeux, France; Centre hospitalier, 61200 Argentan, France.; (Bechet C) Pharmacie de la Croix d'Or, 75017 Paris, France.; (Le Chevalier A) Réseau régional douleur en Basse-Normandie, 14400 Bayeux, France; Centre hospitalier, 50400 Avranches-Granville, France.; (Delorme C) Centre hospitalier, 14400 Bayeux, France; Réseau régional douleur en Basse-Normandie, 14400 Bayeux, France.
Database: PubMed

59. Cancer Pain Management Among Oncology Nurses: Knowledge, Attitude, Related Factors, and Clinical Recommendations: a Systematic Review.

Author(s): Bouya S; Balouchi A; Maleknejad A; Koochakzai M; AlKhasawneh E; Abdollahimohammad A

Source: Journal of cancer education : the official journal of the American Association for Cancer Education; Oct 2019; vol. 34 (no. 5); p. 839-846

Publication Date: Oct 2019

Publication Type(s): Journal Article; Systematic Review

DOI: http://dx.doi.org/10.1007/s13187-018-1433-6

ISSN: 1543-0154

Place of Publication: England

PubMedID: 30315497

Accession Number: 30315497

Available at Journal of cancer education : the official journal of the American Association for Cancer Education - from EBSCO (MEDLINE Complete)

Available at Journal of cancer education : the official journal of the American Association for Cancer Education - from ProQuest (MEDLINE with Full Text) - NHS Version

Available at Journal of cancer education : the official journal of the American Association for Cancer Education - from ProQuest (Health Research Premium) - NHS Version

Keywords: Subject Terms: Attitude; Cancer pain management; Knowledge; Nurses; Systematic review

Abstract: The current study evaluated the oncology nurse's knowledge, attitude, related factors of cancer-related pain management (CPM), and clinical recommendations for improving knowledge and attitude. In this systematic review, international databases (PubMed, EMBASE, Web of science (WOS), Science Direct, and Scopus) were searched for relevant studies published in English language from March 30, 2000 to March 30, 2018. The quality of the studies was evaluated using the Hoy instrument. Out of 888 initial studies, 12 studies performed on 3574 participants were included in the final stage of the review. Based on the results, most studies indicated that nurses had a poor (n = 4) or moderate (n = 4) knowledge of CPM. The lowest and the highest knowledge levels were 28.5% and 75%, respectively. According to most studies, nurses had a fair (average) (n = 4) or negative (n = 3) attitude toward CPM. The important factors related to the nurses' knowledge of CPM included previous pain-related education programs (n = 7) and having work experience with cancer patients (n = 4). The most important barrier was the deficit in staff's knowledge of pain (n = 2). The important clinical recommendations for improving nurses' levels of knowledge included the implementation of educational programs (n = 9), training programs (n = 3) on CPM and including CPM topics in nursing curricula (n = 5). This systematic review showed that most nurses had poor knowledge of CPM and a fair attitude toward CPM, indicating the importance of considering the barriers to knowledge, strengthening the positive relevant factors, and using clinical recommendations based on clinical

guidelines such as including CPM topics in nursing curricula and implementing educational programs on CPM to improve the knowledge, attitude, and skills of oncology nurses. The results of the present study could be used by policymakers to provide care for cancer patients and manage their pain.

Institutions:
(Bouya S) Internal Medicine and Nephrology, Clinical Immunology Research Center, Ali-Ebne Abitaleb Hospital, Zahedan University of Medical Sciences, Zahedan, Iran.; (Balouchi A) Student Research Committee, Nursing and Midwifery School, Iran University of Medical Sciences, Tehran, Iran.; (Maleknejad A) Clinical Immunology Research Center, Ali-Ebne Abitaleb Hospital, Zahedan University of Medical Sciences, Zahedan, Iran. abasetmalekrn@gmail.com.; (Koochakzai M) Department of Midwifery, Zabol University of Medical Science, Zabol, Iran.; (AlKhasawneh E) Department of Maternal & Child Health Nursing, College of Nursing, Sultan Qaboos University, Muscat, Oman.; (Abdollahimohammad A) Zabol University of Medical Sciences, Zabol, Iran.
(Bouya S) Internal Medicine and Nephrology, Clinical Immunology Research Center, Ali-Ebne Abitaleb Hospital, Zahedan University of Medical Sciences, Zahedan, Iran.; (Balouchi A) Student Research Committee, Nursing and Midwifery School, Iran University of Medical Sciences, Tehran, Iran.; (Maleknejad A) Clinical Immunology Research Center, Ali-Ebne Abitaleb Hospital, Zahedan University of Medical Sciences, Zahedan, Iran. abasetmalekrn@gmail.com.; (Koochakzai M) Department of Midwifery, Zabol University of Medical Science, Zabol, Iran.; (AlKhasawneh E) Department of Maternal & Child Health Nursing, College of Nursing, Sultan Qaboos University, Muscat, Oman.; (Abdollahimohammad A) Zabol University of Medical Sciences, Zabol, Iran.
(Bouya S) Internal Medicine and Nephrology, Clinical Immunology Research Center, Ali-Ebne Abitaleb Hospital, Zahedan University of Medical Sciences, Zahedan, Iran.; (Balouchi A) Student Research Committee, Nursing and Midwifery School, Iran University of Medical Sciences, Tehran, Iran.; (Maleknejad A) Clinical Immunology Research Center, Ali-Ebne Abitaleb Hospital, Zahedan University of Medical Sciences, Zahedan, Iran. abasetmalekrn@gmail.com.; (Koochakzai M) Department of Midwifery, Zabol University of Medical Science, Zabol, Iran.; (AlKhasawneh E) Department of Maternal & Child Health Nursing, College of Nursing, Sultan Qaboos University, Muscat, Oman.; (Abdollahimohammad A) Zabol University of Medical Sciences, Zabol, Iran.
(Bouya S) Internal Medicine and Nephrology, Clinical Immunology Research Center, Ali-Ebne Abitaleb Hospital, Zahedan University of Medical Sciences, Zahedan, Iran.; (Balouchi A) Student Research Committee, Nursing and Midwifery School, Iran University of Medical Sciences, Tehran, Iran.; (Maleknejad A) Clinical Immunology Research Center, Ali-Ebne Abitaleb Hospital, Zahedan University of Medical Sciences, Zahedan, Iran. abasetmalekrn@gmail.com.; (Koochakzai M) Department of Midwifery, Zabol University of Medical Science, Zabol, Iran.; (AlKhasawneh E) Department of Maternal & Child Health Nursing, College of Nursing, Sultan Qaboos University, Muscat, Oman.; (Abdollahimohammad A) Zabol University of Medical Sciences, Zabol, Iran.
(Bouya S) Internal Medicine and Nephrology, Clinical Immunology Research Center, Ali-Ebne Abitaleb Hospital, Zahedan University of Medical Sciences, Zahedan, Iran.; (Balouchi A) Student Research Committee, Nursing and Midwifery School, Iran University of Medical Sciences, Tehran, Iran.; (Maleknejad A) Clinical Immunology Research Center, Ali-Ebne Abitaleb Hospital, Zahedan University of Medical

Sciences, Zahedan, Iran. abasetmalekrn@gmail.com.; (Koochakzai M) Department of Midwifery, Zabol University of Medical Science, Zabol, Iran.; (AlKhasawneh E) Department of Maternal & Child Health Nursing, College of Nursing, Sultan Qaboos University, Muscat, Oman.; (Abdollahimohammad A) Zabol University of Medical Sciences, Zabol, Iran.
(Bouya S) Internal Medicine and Nephrology, Clinical Immunology Research Center, Ali-Ebne Abitaleb Hospital, Zahedan University of Medical Sciences, Zahedan, Iran.; (Balouchi A) Student Research Committee, Nursing and Midwifery School, Iran University of Medical Sciences, Tehran, Iran.; (Maleknejad A) Clinical Immunology Research Center, Ali-Ebne Abitaleb Hospital, Zahedan University of Medical
Sciences, Zahedan, Iran. abasetmalekrn@gmail.com.; (Koochakzai M) Department of Midwifery, Zabol University of Medical Science, Zabol, Iran.; (AlKhasawneh E) Department of Maternal & Child Health Nursing, College of Nursing, Sultan Qaboos University, Muscat, Oman.; (Abdollahimohammad A) Zabol University of Medical Sciences, Zabol, Iran.
Database: PubMed

60. Enhancing Cancer Pain Assessment and Management in Hospice.

Author(s): Miner MB; Stephens K; Swanson-Biearman B; Leone V; Whiteman K

Source: Journal of hospice and palliative nursing : JHPN : the official journal of the Hospice and Palliative Nurses Association; Oct 2018; vol. 20 (no. 5); p. 452-458

Publication Date: Oct 2018

Publication Type(s): Journal Article

DOI: http://dx.doi.org/10.1097/NJH.0000000000000467

ISSN: 1539-0705

Place of Publication: United States

PubMedID: 30188438

Accession Number: 30188438

Abstract: Pain is one of the most common symptoms in individuals with cancer and is directly associated with significantly reduced quality of life. The purpose of this project was to enhance assessment and management of cancer-related pain for patients in the hospice setting. Nurse attitudes and knowledge about pain were evaluated using the Nurses' Knowledge and Attitudes Survey Regarding Pain before and 6 weeks after an educational program. Nurses completed a pain assessment at each home visit and followed an algorithm based on the National Comprehensive Cancer Network Clinical Practice Guidelines to manage pain. Baseline data were collected on the last 30 patients admitted into hospice with cancer as the primary diagnosis before project implementation. Following the practice change, chart audits of the patients' reported pain and adherence to recommended management were manually extracted from 26 patient records. Results revealed statistically significant differences in acquired knowledge ($t = 3.95$, $P < .05$) and attainment of patient-identified pain goals ($t = 23.904$, $P < .05$). Patient-reported pain levels decreased by 21%, and comprehensive pain assessment completion rates increased by 10% during the project. Current knowledge of evidence-based pain interventions and a management algorithm improved pain control in patients with cancer.

Institutions:

(Miner MB) Melissa B. Miner, DNP, RN, CNE, is a graduate, Waynesburg University, Pennsylvania. Kimberly Stephens, DNP, RN, is assistant professor, Waynesburg University, Pennsylvania. Brenda Swanson-Biearman, DNP, MPH, RN, is assistant professor, Duquesne University, Pittsburgh, Pennsylvania. Vickie Leone, DNP, RN, is vice president, Fayette Home Care & Hospice, Lemont Furnace, Pennsylvania. Kimberly Whiteman, DNP, RN, is assistant professor, Waynesburg University, Pennsylvania.
Database: PubMed

61. PROMs for Pain in Adult Cancer Patients: A Systematic Review of Measurement Properties.

Author(s): Abahussin AA; West RM; Wong DC; Ziegler LE

Source: Pain practice : the official journal of World Institute of Pain; 2019; vol. 19 (no. 1); p. 93-117

Publication Date: 2019

Publication Type(s): Journal Article; Research Support, Non-U.S. Gov't; Systematic Review

DOI: http://dx.doi.org/10.1111/papr.12711

ISSN: 1533-2500

Place of Publication: United States

PubMedID: 29772118

Accession Number: 29772118

Available at Pain Practice - from Wiley Online Library Medicine and Nursing Collection 2020

Available at Pain Practice - from EBSCO (CINAHL Complete)

Available at Pain Practice - from EBSCO (Psychology and Behavioral Sciences Collection)

Available at Pain Practice - from Unpaywall

Keywords: Subject Terms: *PROMs; *adult; *cancer; *measurement properties; *pain measurement; *psychometrics; *systematic review

Abstract:CONTEXT: Pain is one of the most devastating symptoms for cancer patients. One third of patients who experience pain do not receive effective treatment. A key barrier to effective pain management is lack of routine measurement and monitoring of pain. Patient-reported outcome measures (PROMs) are recommended for measuring cancer pain. However, evidence to guide the selection of the most appropriate measure to identify and monitor cancer pain is limited. A systematic review of measurement properties of PROMs for pain in cancer patients is needed to identify the best validated measure for adoption to an electronic platform.OBJECTIVES: To systematically review measurement properties of PROMs used for adult cancer patients to measure pain and, as a secondary goal, to investigate the evidence of validated mobile health (mHealth) applications used to measure pain (registration number: CRD42017065575).METHODS: Medline, Embase, and the Cumulative Index to Nursing and Allied Health Literature (CINAHL) were systematically searched in March 2018 for studies examining measurement properties for PROMs for pain in adult cancer patients. The methodological quality of

the studies and their results were appraised using the Consensus-based Standards for the Selection of Health Measurement Instruments (COSMIN) checklist and specific measurement properties criteria, respectively.RESULTS: Sixteen studies evaluating 8 instruments were included. No studies using a PROM in an mHealth application were identified. The methodological quality of the measurement properties ranged between poor and fair. No instrument showed strong positive evidence for all the evaluated measurement properties. Based on the available evidence, the Brief Pain Inventory-Short Form (BPI-SF) had the strongest evidence to support its selection for the measurement of cancer pain.CONCLUSION: The BPI-SF was the best performing measure across all properties evaluated through COSMIN. Better quality validation studies of PROMs for cancer pain are needed to explore the full range of measurement properties. Utilizing mHealth applications to measure pain in cancer patients is an innovative approach worthy of further investigation.

Institutions:
(Abahussin AA) Leeds institute of Health Sciences, School of Medicine, University of Leeds, Leeds, U.K.; (West RM) Leeds institute of Health Sciences, School of Medicine, University of Leeds, Leeds, U.K.; (Wong DC) Leeds institute of Health Sciences, School of Medicine, University of Leeds, Leeds, U.K.; (Ziegler LE) Leeds institute of Health Sciences, School of Medicine, University of Leeds, Leeds, U.K.
(Abahussin AA) Leeds institute of Health Sciences, School of Medicine, University of Leeds, Leeds, U.K.; (West RM) Leeds institute of Health Sciences, School of Medicine, University of Leeds, Leeds, U.K.; (Wong DC) Leeds institute of Health Sciences, School of Medicine, University of Leeds, Leeds, U.K.; (Ziegler LE) Leeds institute of Health Sciences, School of Medicine, University of Leeds, Leeds, U.K.
(Abahussin AA) Leeds institute of Health Sciences, School of Medicine, University of Leeds, Leeds, U.K.; (West RM) Leeds institute of Health Sciences, School of Medicine, University of Leeds, Leeds, U.K.; (Wong DC) Leeds institute of Health Sciences, School of Medicine, University of Leeds, Leeds, U.K.; (Ziegler LE) Leeds institute of Health Sciences, School of Medicine, University of Leeds, Leeds, U.K.
(Abahussin AA) Leeds institute of Health Sciences, School of Medicine, University of Leeds, Leeds, U.K.; (West RM) Leeds institute of Health Sciences, School of Medicine, University of Leeds, Leeds, U.K.; (Wong DC) Leeds institute of Health Sciences, School of Medicine, University of Leeds, Leeds, U.K.; (Ziegler LE) Leeds institute of Health Sciences, School of Medicine, University of Leeds, Leeds, U.K.
Database: PubMed

62. Parents' Voice in Managing the Pain of Children with Cancer during Palliative Care.

Author(s): Mariyana R; Allenidekania A; Nurhaeni N

Source: Indian journal of palliative care; 2018; vol. 24 (no. 2); p. 156-161

Publication Date: 2018

Publication Type(s): Journal Article

DOI: http://dx.doi.org/10.4103/IJPC.IJPC_198_17

ISSN: 0973-1075

Place of Publication: United States

PubMedID: 29736117

Accession Number: 29736117

Available at Indian Journal of Palliative Care - from Europe PubMed Central - Open Access

Available at Indian Journal of Palliative Care - from EBSCO (CINAHL Complete)

Available at Indian Journal of Palliative Care - from ProQuest (Health Research Premium) - NHS Version

Available at Indian Journal of Palliative Care - from Unpaywall

Keywords: Subject Terms: Cancer; Indonesia; caring for pain; child; lived experience; palliative care

Abstract: Context: Pain experienced by children can adversely affect their growth and development. Pain is a major health problem for cancer patients and remains an unresolved problem.Aim: To know how the experiences of mothers managing their children's pain during palliative care following cancer diagnosis.Background: Pain experienced by children can adversely affect their growth and development.Subject and Methods: Using qualitative methods within a descriptive phenomenological approach, in-depth interviews were conducted with parents (mostly mothers) of eight children diagnosed with cancer. The data were collected using the snowball sampling method.Results: Participants experienced in managing the pain of children with cancer. Analysis of the results identified 8 themes: the dimensions of pain experienced by children undergoing palliative care; mothers' physical and psychological responses; mothers' emotional responses; barriers encountered by mothers when taking care of their child at home; mothers' interventions to reduce their child's pain; mothers' efforts to distract their child from pain; giving encouragement when the child is in pain; and mothers' efforts and prayers to make their child comfort.Conclusion: It can be concluded that the child's pain is the main cause of mothers' stress and pressure and also affects the daily lives of mothers and children. Along with the most effective intervention, nurses need to provide mothers and children with adequate information about cancer pain.

Institutions:
(Mariyana R) Department of Pediatric Nursing, Faculty of Nursing, Universitas Indonesia, Depok, Indonesia.; (Allenidekania A) Department of Pediatric Nursing, Faculty of Nursing, Universitas Indonesia, Depok, Indonesia.; (Nurhaeni N) Department of Pediatric Nursing, Faculty of Nursing, Universitas Indonesia, Depok, Indonesia.
(Mariyana R) Department of Pediatric Nursing, Faculty of Nursing, Universitas Indonesia, Depok, Indonesia.; (Allenidekania A) Department of Pediatric Nursing, Faculty of Nursing, Universitas Indonesia, Depok, Indonesia.; (Nurhaeni N) Department of Pediatric Nursing, Faculty of Nursing, Universitas Indonesia, Depok, Indonesia.
(Mariyana R) Department of Pediatric Nursing, Faculty of Nursing, Universitas Indonesia, Depok, Indonesia.; (Allenidekania A) Department of Pediatric Nursing, Faculty of Nursing, Universitas Indonesia, Depok, Indonesia.; (Nurhaeni N) Department of Pediatric Nursing, Faculty of Nursing, Universitas Indonesia, Depok, Indonesia.
Database: PubMed

63. Oncology nurse reflection on the necessity of a pain assessment tool for Indonesian cancer patients.

Author(s): Nur Rachmawati I; Afiyanti Y

Source: Enfermeria clinica; Feb 2018 ; p. 207-211

Publication Date: Feb 2018

Publication Type(s): Journal Article

DOI: http://dx.doi.org/10.1016/S1130-8621(18)30069-X

ISSN: 1579-2013

Place of Publication: Spain

PubMedID: 29650188

Accession Number: 29650188

Keywords: Subject Terms: Cancer pain; Oncology nursing; Pain assessment

Abstract:OBJECTIVE: The purpose of this study was to obtain an overview on nurses' opinions and perceptions related to pain assessment and other problems experienced in evaluating pain in cancer patients.METHOD: This descriptive qualitative study was part of a series of studies to develop a pain assessment tool for cancer patients in Indonesia. Data were collected by semi-structured interviews from 16 oncology nurses. A cross-case comparison of the interview responses was used to analyze the data from verbatim interview transcripts.RESULTS: Six themes were identified: pain as the main complaint reported by cancer patients; pain assessment using a pain scale; an awareness of the absence of nurses' role in pain management; a lack of understanding of pain conditions; acknowledgement that a pain assessment tool should be practical and user friendly; and the need to develop a pain assessment tool. We also explored the knowledge of nurses in pain management and achieved an in-depth understanding of the nurses' desire to seek additional knowledge related to pain assessment.CONCLUSIONS: A pain assessment tool should be developed to assist nurses to independently interpret data about pain and allow them to select or modify interventions consistent with the patients' needs. Pain management for cancer patients is one of the essential nurses' responsibilities in providing comprehensive meaningful nursing care.

Institutions:
(Nur Rachmawati I) Faculty of Nursing, Universitas Indonesia, Depok, Jawa Barat, Indonesia.; (Afiyanti Y) Faculty of Nursing, Universitas Indonesia, Depok, Jawa Barat, Indonesia. Electronic address: yatikris@ui.ac.id.
(Nur Rachmawati I) Faculty of Nursing, Universitas Indonesia, Depok, Jawa Barat, Indonesia.; (Afiyanti Y) Faculty of Nursing, Universitas Indonesia, Depok, Jawa Barat, Indonesia. Electronic address: yatikris@ui.ac.id.
Database: PubMed

64. Improving the management of pain from advanced cancer in the community: study protocol for a pragmatic multicentre randomised controlled trial.

Author(s): Allsop MJ; Wright-Hughes A; Black K; Hartley S; Fletcher M; Ziegler LE; Bewick BM; Meads D; Hughes ND; Closs SJ; Hulme C; Taylor S; Flemming K; Hackett J; O'Dwyer JL; Brown JM; Bennett MI

Source: BMJ open; ; vol. 8 (no. 3); p. e021965

Publication Type(s): Journal Article; Multicenter Study; Pragmatic Clinical Trial; Research Support, Non-U.S. Gov't

DOI: http://dx.doi.org/10.1136/bmjopen-2018-021965

ISSN: 2044-6055

Place of Publication: England

PubMedID: 29572400

Accession Number: 29572400

Available at BMJ open - from Europe PubMed Central - Open Access

Available at BMJ open - from HighWire - Free Full Text

Available at BMJ open - from ProQuest (Health Research Premium) - NHS Version

Available at BMJ open - from Unpaywall

Keywords: Subject Terms: *cancer pain; *education intervention; *ehealth; *palliative care; *routine monitoring; *self-management

Abstract:INTRODUCTION: For patients with advanced cancer, research shows that pain is frequent, burdensome and undertreated. Evidence-based approaches to support cancer pain management have been developed but have not been implemented within the context of the UK National Health Service. This protocol is for a pragmatic multicentre randomised controlled trial (RCT) to assess feasibility, acceptability, effectiveness and cost-effectiveness for a multicomponent intervention for pain management in patients with advanced cancer.METHODS AND ANALYSIS: This trial will assess the feasibility of implementation and uptake of evidence-based interventions, developed and piloted as part of the Improving the Management of Pain from Advanced Cancer in the Community Programme grant, into routine clinical practice and determine whether there are potential differences with respect to patient-rated pain, patient pain knowledge and experience, healthcare use, quality of life and cost-effectiveness. 160 patients will receive either the intervention (usual care plus supported self-management) delivered within the oncology clinic and palliative care services by locally assigned community palliative care nurses, consisting of a self-management educational intervention and eHealth intervention for routine pain assessment and monitoring; or usual care. The primary outcomes are to assess implementation and uptake of the interventions, and differences in terms of pain severity. Secondary outcomes include pain interference, participant pain knowledge and experience, and cost-effectiveness. Outcome assessment will be blinded and patient-reported outcome measures collected via post at 6 and 12 weeks following randomisation.ETHICS AND DISSEMINATION: This RCT has the potential to significantly influence National Health Service delivery to community-based patients with pain from advanced cancer. We aim to provide definitive evidence of whether two simple interventions delivered by community palliative care nurse in palliative care that support-self-management are clinically effective and cost-effective additions to standard community palliative care.TRIAL REGISTRATION NUMBER: ISRCTN18281271; Pre-results.

Institutions:
(Allsop MJ) Academic Unit of Palliative Care, Leeds Institute of Health Sciences, University of Leeds, Leeds, UK.; (Wright-Hughes A) Clinical Trials Research Unit, Leeds Institute of Clinical Trials Research, University of Leeds, Leeds, UK.; (Black K)

Academic Unit of Palliative Care, Leeds Institute of Health Sciences, University of Leeds, Leeds, UK.; (Hartley S) Clinical Trials Research Unit, Leeds Institute of Clinical Trials Research, University of Leeds, Leeds, UK.; (Fletcher M) Clinical Trials Research Unit, Leeds Institute of Clinical Trials Research, University of Leeds, Leeds, UK.; (Ziegler LE) Academic Unit of Palliative Care, Leeds Institute of Health Sciences, University of Leeds, Leeds, UK.; (Bewick BM) Division of Psychological and Social Medicine, Leeds Institute of Health Sciences, University of Leeds, Leeds, UK.; (Meads D) Academic Unit of Health Economics, Leeds Institute of Health Sciences, University of Leeds, Leeds, UK.; (Hughes ND) School of Healthcare, Baines Wing, University of Leeds, Leeds, UK.; (Closs SJ) School of Healthcare, Baines Wing, University of Leeds, Leeds, UK.; (Hulme C) Academic Unit of Health Economics, Leeds Institute of Health Sciences, University of Leeds, Leeds, UK.; (Taylor S) Academic Unit of Palliative Care, Leeds Institute of Health Sciences, University of Leeds, Leeds, UK.; (Flemming K) Department of Health Sciences, University of York, Heslington, UK.; (Hackett J) Academic Unit of Palliative Care, Leeds Institute of Health Sciences, University of Leeds, Leeds, UK.; (O'Dwyer JL) Academic Unit of Health Economics, Leeds Institute of Health Sciences, University of Leeds, Leeds, UK.; (Brown JM) Clinical Trials Research Unit, Leeds Institute of Clinical Trials Research, University of Leeds, Leeds, UK.; (Bennett MI) Academic Unit of Palliative Care, Leeds Institute of Health Sciences, University of Leeds, Leeds, UK.

(Allsop MJ) Academic Unit of Palliative Care, Leeds Institute of Health Sciences, University of Leeds, Leeds, UK.; (Wright-Hughes A) Clinical Trials Research Unit, Leeds Institute of Clinical Trials Research, University of Leeds, Leeds, UK.; (Black K) Academic Unit of Palliative Care, Leeds Institute of Health Sciences, University of Leeds, Leeds, UK.; (Hartley S) Clinical Trials Research Unit, Leeds Institute of Clinical Trials Research, University of Leeds, Leeds, UK.; (Fletcher M) Clinical Trials Research Unit, Leeds Institute of Clinical Trials Research, University of Leeds, Leeds, UK.; (Ziegler LE) Academic Unit of Palliative Care, Leeds Institute of Health Sciences, University of Leeds, Leeds, UK.; (Bewick BM) Division of Psychological and Social Medicine, Leeds Institute of Health Sciences, University of Leeds, Leeds, UK.; (Meads D) Academic Unit of Health Economics, Leeds Institute of Health Sciences, University of Leeds, Leeds, UK.; (Hughes ND) School of Healthcare, Baines Wing, University of Leeds, Leeds, UK.; (Closs SJ) School of Healthcare, Baines Wing, University of Leeds, Leeds, UK.; (Hulme C) Academic Unit of Health Economics, Leeds Institute of Health Sciences, University of Leeds, Leeds, UK.; (Taylor S) Academic Unit of Palliative Care, Leeds Institute of Health Sciences, University of Leeds, Leeds, UK.; (Flemming K) Department of Health Sciences, University of York, Heslington, UK.; (Hackett J) Academic Unit of Palliative Care, Leeds Institute of Health Sciences, University of Leeds, Leeds, UK.; (O'Dwyer JL) Academic Unit of Health Economics, Leeds Institute of Health Sciences, University of Leeds, Leeds, UK.; (Brown JM) Clinical Trials Research Unit, Leeds Institute of Clinical Trials Research, University of Leeds, Leeds, UK.; (Bennett MI) Academic Unit of Palliative Care, Leeds Institute of Health Sciences, University of Leeds, Leeds, UK.

(Allsop MJ) Academic Unit of Palliative Care, Leeds Institute of Health Sciences, University of Leeds, Leeds, UK.; (Wright-Hughes A) Clinical Trials Research Unit, Leeds Institute of Clinical Trials Research, University of Leeds, Leeds, UK.; (Black K) Academic Unit of Palliative Care, Leeds Institute of Health Sciences, University of Leeds, Leeds, UK.; (Hartley S) Clinical Trials Research Unit, Leeds Institute of

Clinical Trials Research, University of Leeds, Leeds, UK.; (Fletcher M) Clinical Trials Research Unit, Leeds Institute of Clinical Trials Research, University of Leeds, Leeds, UK.; (Ziegler LE) Academic Unit of Palliative Care, Leeds Institute of Health Sciences, University of Leeds, Leeds, UK.; (Bewick BM) Division of Psychological and Social Medicine, Leeds Institute of Health Sciences, University of Leeds, Leeds, UK.; (Meads D) Academic Unit of Health Economics, Leeds Institute of Health Sciences, University of Leeds, Leeds, UK.; (Hughes ND) School of Healthcare, Baines Wing, University of Leeds, Leeds, UK.; (Closs SJ) School of Healthcare, Baines Wing, University of Leeds, Leeds, UK.; (Hulme C) Academic Unit of Health Economics, Leeds Institute of Health Sciences, University of Leeds, Leeds, UK.; (Taylor S) Academic Unit of Palliative Care, Leeds Institute of Health Sciences, University of Leeds, Leeds, UK.; (Flemming K) Department of Health Sciences, University of York, Heslington, UK.; (Hackett J) Academic Unit of Palliative Care, Leeds Institute of Health Sciences, University of Leeds, Leeds, UK.; (O'Dwyer JL) Academic Unit of Health Economics, Leeds Institute of Health Sciences, University of Leeds, Leeds, UK.; (Brown JM) Clinical Trials Research Unit, Leeds Institute of Clinical Trials Research, University of Leeds, Leeds, UK.; (Bennett MI) Academic Unit of Palliative Care, Leeds Institute of Health Sciences, University of Leeds, Leeds, UK.

(Allsop MJ) Academic Unit of Palliative Care, Leeds Institute of Health Sciences, University of Leeds, Leeds, UK.; (Wright-Hughes A) Clinical Trials Research Unit, Leeds Institute of Clinical Trials Research, University of Leeds, Leeds, UK.; (Black K) Academic Unit of Palliative Care, Leeds Institute of Health Sciences, University of Leeds, Leeds, UK.; (Hartley S) Clinical Trials Research Unit, Leeds Institute of Clinical Trials Research, University of Leeds, Leeds, UK.; (Fletcher M) Clinical Trials Research Unit, Leeds Institute of Clinical Trials Research, University of Leeds, Leeds, UK.; (Ziegler LE) Academic Unit of Palliative Care, Leeds Institute of Health Sciences, University of Leeds, Leeds, UK.; (Bewick BM) Division of Psychological and Social Medicine, Leeds Institute of Health Sciences, University of Leeds, Leeds, UK.; (Meads D) Academic Unit of Health Economics, Leeds Institute of Health Sciences, University of Leeds, Leeds, UK.; (Hughes ND) School of Healthcare, Baines Wing, University of Leeds, Leeds, UK.; (Closs SJ) School of Healthcare, Baines Wing, University of Leeds, Leeds, UK.; (Hulme C) Academic Unit of Health Economics, Leeds Institute of Health Sciences, University of Leeds, Leeds, UK.; (Taylor S) Academic Unit of Palliative Care, Leeds Institute of Health Sciences, University of Leeds, Leeds, UK.; (Flemming K) Department of Health Sciences, University of York, Heslington, UK.; (Hackett J) Academic Unit of Palliative Care, Leeds Institute of Health Sciences, University of Leeds, Leeds, UK.; (O'Dwyer JL) Academic Unit of Health Economics, Leeds Institute of Health Sciences, University of Leeds, Leeds, UK.; (Brown JM) Clinical Trials Research Unit, Leeds Institute of Clinical Trials Research, University of Leeds, Leeds, UK.; (Bennett MI) Academic Unit of Palliative Care, Leeds Institute of Health Sciences, University of Leeds, Leeds, UK.

(Allsop MJ) Academic Unit of Palliative Care, Leeds Institute of Health Sciences, University of Leeds, Leeds, UK.; (Wright-Hughes A) Clinical Trials Research Unit, Leeds Institute of Clinical Trials Research, University of Leeds, Leeds, UK.; (Black K) Academic Unit of Palliative Care, Leeds Institute of Health Sciences, University of Leeds, Leeds, UK.; (Hartley S) Clinical Trials Research Unit, Leeds Institute of Clinical Trials Research, University of Leeds, Leeds, UK.; (Fletcher M) Clinical Trials Research Unit, Leeds Institute of Clinical Trials Research, University of Leeds,

Leeds, UK.; (Ziegler LE) Academic Unit of Palliative Care, Leeds Institute of Health Sciences, University of Leeds, Leeds, UK.; (Bewick BM) Division of Psychological and Social Medicine, Leeds Institute of Health Sciences, University of Leeds, Leeds, UK.; (Meads D) Academic Unit of Health Economics, Leeds Institute of Health Sciences, University of Leeds, Leeds, UK.; (Hughes ND) School of Healthcare, Baines Wing, University of Leeds, Leeds, UK.; (Closs SJ) School of Healthcare, Baines Wing, University of Leeds, Leeds, UK.; (Hulme C) Academic Unit of Health Economics, Leeds Institute of Health Sciences, University of Leeds, Leeds, UK.; (Taylor S) Academic Unit of Palliative Care, Leeds Institute of Health Sciences, University of Leeds, Leeds, UK.; (Flemming K) Department of Health Sciences, University of York, Heslington, UK.; (Hackett J) Academic Unit of Palliative Care, Leeds Institute of Health Sciences, University of Leeds, Leeds, UK.; (O'Dwyer JL) Academic Unit of Health Economics, Leeds Institute of Health Sciences, University of Leeds, Leeds, UK.; (Brown JM) Clinical Trials Research Unit, Leeds Institute of Clinical Trials Research, University of Leeds, Leeds, UK.; (Bennett MI) Academic Unit of Palliative Care, Leeds Institute of Health Sciences, University of Leeds, Leeds, UK.
(Allsop MJ) Academic Unit of Palliative Care, Leeds Institute of Health Sciences, University of Leeds, Leeds, UK.; (Wright-Hughes A) Clinical Trials Research Unit, Leeds Institute of Clinical Trials Research, University of Leeds, Leeds, UK.; (Black K) Academic Unit of Palliative Care, Leeds Institute of Health Sciences, University of Leeds, Leeds, UK.; (Hartley S) Clinical Trials Research Unit, Leeds Institute of Clinical Trials Research, University of Leeds, Leeds, UK.; (Fletcher M) Clinical Trials Research Unit, Leeds Institute of Clinical Trials Research, University of Leeds, Leeds, UK.; (Ziegler LE) Academic Unit of Palliative Care, Leeds Institute of Health Sciences, University of Leeds, Leeds, UK.; (Bewick BM) Division of Psychological and Social Medicine, Leeds Institute of Health Sciences, University of Leeds, Leeds, UK.; (Meads D) Academic Unit of Health Economics, Leeds Institute of Health Sciences, University of Leeds, Leeds, UK.; (Hughes ND) School of Healthcare, Baines Wing, University of Leeds, Leeds, UK.; (Closs SJ) School of Healthcare, Baines Wing, University of Leeds, Leeds, UK.; (Hulme C) Academic Unit of Health Economics, Leeds Institute of Health Sciences, University of Leeds, Leeds, UK.; (Taylor S) Academic Unit of Palliative Care, Leeds Institute of Health Sciences, University of Leeds, Leeds, UK.; (Flemming K) Department of Health Sciences, University of York, Heslington, UK.; (Hackett J) Academic Unit of Palliative Care, Leeds Institute of Health Sciences, University of Leeds, Leeds, UK.; (O'Dwyer JL) Academic Unit of Health Economics, Leeds Institute of Health Sciences, University of Leeds, Leeds, UK.; (Brown JM) Clinical Trials Research Unit, Leeds Institute of Clinical Trials Research, University of Leeds, Leeds, UK.; (Bennett MI) Academic Unit of Palliative Care, Leeds Institute of Health Sciences, University of Leeds, Leeds, UK.
(Allsop MJ) Academic Unit of Palliative Care, Leeds Institute of Health Sciences, University of Leeds, Leeds, UK.; (Wright-Hughes A) Clinical Trials Research Unit, Leeds Institute of Clinical Trials Research, University of Leeds, Leeds, UK.; (Black K) Academic Unit of Palliative Care, Leeds Institute of Health Sciences, University of Leeds, Leeds, UK.; (Hartley S) Clinical Trials Research Unit, Leeds Institute of Clinical Trials Research, University of Leeds, Leeds, UK.; (Fletcher M) Clinical Trials Research Unit, Leeds Institute of Clinical Trials Research, University of Leeds, Leeds, UK.; (Ziegler LE) Academic Unit of Palliative Care, Leeds Institute of Health Sciences, University of Leeds, Leeds, UK.; (Bewick BM) Division of Psychological

and Social Medicine, Leeds Institute of Health Sciences, University of Leeds, Leeds, UK.; (Meads D) Academic Unit of Health Economics, Leeds Institute of Health Sciences, University of Leeds, Leeds, UK.; (Hughes ND) School of Healthcare, Baines Wing, University of Leeds, Leeds, UK.; (Closs SJ) School of Healthcare, Baines Wing, University of Leeds, Leeds, UK.; (Hulme C) Academic Unit of Health Economics, Leeds Institute of Health Sciences, University of Leeds, Leeds, UK.; (Taylor S) Academic Unit of Palliative Care, Leeds Institute of Health Sciences, University of Leeds, Leeds, UK.; (Flemming K) Department of Health Sciences, University of York, Heslington, UK.; (Hackett J) Academic Unit of Palliative Care, Leeds Institute of Health Sciences, University of Leeds, Leeds, UK.; (O'Dwyer JL) Academic Unit of Health Economics, Leeds Institute of Health Sciences, University of Leeds, Leeds, UK.; (Brown JM) Clinical Trials Research Unit, Leeds Institute of Clinical Trials Research, University of Leeds, Leeds, UK.; (Bennett MI) Academic Unit of Palliative Care, Leeds Institute of Health Sciences, University of Leeds, Leeds, UK.
(Allsop MJ) Academic Unit of Palliative Care, Leeds Institute of Health Sciences, University of Leeds, Leeds, UK.; (Wright-Hughes A) Clinical Trials Research Unit, Leeds Institute of Clinical Trials Research, University of Leeds, Leeds, UK.; (Black K) Academic Unit of Palliative Care, Leeds Institute of Health Sciences, University of Leeds, Leeds, UK.; (Hartley S) Clinical Trials Research Unit, Leeds Institute of Clinical Trials Research, University of Leeds, Leeds, UK.; (Fletcher M) Clinical Trials Research Unit, Leeds Institute of Clinical Trials Research, University of Leeds, Leeds, UK.; (Ziegler LE) Academic Unit of Palliative Care, Leeds Institute of Health Sciences, University of Leeds, Leeds, UK.; (Bewick BM) Division of Psychological and Social Medicine, Leeds Institute of Health Sciences, University of Leeds, Leeds, UK.; (Meads D) Academic Unit of Health Economics, Leeds Institute of Health Sciences, University of Leeds, Leeds, UK.; (Hughes ND) School of Healthcare, Baines Wing, University of Leeds, Leeds, UK.; (Closs SJ) School of Healthcare, Baines Wing, University of Leeds, Leeds, UK.; (Hulme C) Academic Unit of Health Economics, Leeds Institute of Health Sciences, University of Leeds, Leeds, UK.; (Taylor S) Academic Unit of Palliative Care, Leeds Institute of Health Sciences, University of Leeds, Leeds, UK.; (Flemming K) Department of Health Sciences, University of York, Heslington, UK.; (Hackett J) Academic Unit of Palliative Care, Leeds Institute of Health Sciences, University of Leeds, Leeds, UK.; (O'Dwyer JL) Academic Unit of Health Economics, Leeds Institute of Health Sciences, University of Leeds, Leeds, UK.; (Brown JM) Clinical Trials Research Unit, Leeds Institute of Clinical Trials Research, University of Leeds, Leeds, UK.; (Bennett MI) Academic Unit of Palliative Care, Leeds Institute of Health Sciences, University of Leeds, Leeds, UK.
(Allsop MJ) Academic Unit of Palliative Care, Leeds Institute of Health Sciences, University of Leeds, Leeds, UK.; (Wright-Hughes A) Clinical Trials Research Unit, Leeds Institute of Clinical Trials Research, University of Leeds, Leeds, UK.; (Black K) Academic Unit of Palliative Care, Leeds Institute of Health Sciences, University of Leeds, Leeds, UK.; (Hartley S) Clinical Trials Research Unit, Leeds Institute of Clinical Trials Research, University of Leeds, Leeds, UK.; (Fletcher M) Clinical Trials Research Unit, Leeds Institute of Clinical Trials Research, University of Leeds, Leeds, UK.; (Ziegler LE) Academic Unit of Palliative Care, Leeds Institute of Health Sciences, University of Leeds, Leeds, UK.; (Bewick BM) Division of Psychological and Social Medicine, Leeds Institute of Health Sciences, University of Leeds, Leeds, UK.; (Meads D) Academic Unit of Health Economics, Leeds Institute of Health

Sciences, University of Leeds, Leeds, UK.; (Hughes ND) School of Healthcare, Baines Wing, University of Leeds, Leeds, UK.; (Closs SJ) School of Healthcare, Baines Wing, University of Leeds, Leeds, UK.; (Hulme C) Academic Unit of Health Economics, Leeds Institute of Health Sciences, University of Leeds, Leeds, UK.; (Taylor S) Academic Unit of Palliative Care, Leeds Institute of Health Sciences, University of Leeds, Leeds, UK.; (Flemming K) Department of Health Sciences, University of York, Heslington, UK.; (Hackett J) Academic Unit of Palliative Care, Leeds Institute of Health Sciences, University of Leeds, Leeds, UK.; (O'Dwyer JL) Academic Unit of Health Economics, Leeds Institute of Health Sciences, University of Leeds, Leeds, UK.; (Brown JM) Clinical Trials Research Unit, Leeds Institute of Clinical Trials Research, University of Leeds, Leeds, UK.; (Bennett MI) Academic Unit of Palliative Care, Leeds Institute of Health Sciences, University of Leeds, Leeds, UK.
(Allsop MJ) Academic Unit of Palliative Care, Leeds Institute of Health Sciences, University of Leeds, Leeds, UK.; (Wright-Hughes A) Clinical Trials Research Unit, Leeds Institute of Clinical Trials Research, University of Leeds, Leeds, UK.; (Black K) Academic Unit of Palliative Care, Leeds Institute of Health Sciences, University of Leeds, Leeds, UK.; (Hartley S) Clinical Trials Research Unit, Leeds Institute of Clinical Trials Research, University of Leeds, Leeds, UK.; (Fletcher M) Clinical Trials Research Unit, Leeds Institute of Clinical Trials Research, University of Leeds, Leeds, UK.; (Ziegler LE) Academic Unit of Palliative Care, Leeds Institute of Health Sciences, University of Leeds, Leeds, UK.; (Bewick BM) Division of Psychological and Social Medicine, Leeds Institute of Health Sciences, University of Leeds, Leeds, UK.; (Meads D) Academic Unit of Health Economics, Leeds Institute of Health Sciences, University of Leeds, Leeds, UK.; (Hughes ND) School of Healthcare, Baines Wing, University of Leeds, Leeds, UK.; (Closs SJ) School of Healthcare, Baines Wing, University of Leeds, Leeds, UK.; (Hulme C) Academic Unit of Health Economics, Leeds Institute of Health Sciences, University of Leeds, Leeds, UK.; (Taylor S) Academic Unit of Palliative Care, Leeds Institute of Health Sciences, University of Leeds, Leeds, UK.; (Flemming K) Department of Health Sciences, University of York, Heslington, UK.; (Hackett J) Academic Unit of Palliative Care, Leeds Institute of Health Sciences, University of Leeds, Leeds, UK.; (O'Dwyer JL) Academic Unit of Health Economics, Leeds Institute of Health Sciences, University of Leeds, Leeds, UK.; (Brown JM) Clinical Trials Research Unit, Leeds Institute of Clinical Trials Research, University of Leeds, Leeds, UK.; (Bennett MI) Academic Unit of Palliative Care, Leeds Institute of Health Sciences, University of Leeds, Leeds, UK.
(Allsop MJ) Academic Unit of Palliative Care, Leeds Institute of Health Sciences, University of Leeds, Leeds, UK.; (Wright-Hughes A) Clinical Trials Research Unit, Leeds Institute of Clinical Trials Research, University of Leeds, Leeds, UK.; (Black K) Academic Unit of Palliative Care, Leeds Institute of Health Sciences, University of Leeds, Leeds, UK.; (Hartley S) Clinical Trials Research Unit, Leeds Institute of Clinical Trials Research, University of Leeds, Leeds, UK.; (Fletcher M) Clinical Trials Research Unit, Leeds Institute of Clinical Trials Research, University of Leeds, Leeds, UK.; (Ziegler LE) Academic Unit of Palliative Care, Leeds Institute of Health Sciences, University of Leeds, Leeds, UK.; (Bewick BM) Division of Psychological and Social Medicine, Leeds Institute of Health Sciences, University of Leeds, Leeds, UK.; (Meads D) Academic Unit of Health Economics, Leeds Institute of Health Sciences, University of Leeds, Leeds, UK.; (Hughes ND) School of Healthcare, Baines Wing, University of Leeds, Leeds, UK.; (Closs SJ) School of Healthcare,

Baines Wing, University of Leeds, Leeds, UK.; (Hulme C) Academic Unit of Health Economics, Leeds Institute of Health Sciences, University of Leeds, Leeds, UK.; (Taylor S) Academic Unit of Palliative Care, Leeds Institute of Health Sciences, University of Leeds, Leeds, UK.; (Flemming K) Department of Health Sciences, University of York, Heslington, UK.; (Hackett J) Academic Unit of Palliative Care, Leeds Institute of Health Sciences, University of Leeds, Leeds, UK.; (O'Dwyer JL) Academic Unit of Health Economics, Leeds Institute of Health Sciences, University of Leeds, Leeds, UK.; (Brown JM) Clinical Trials Research Unit, Leeds Institute of Clinical Trials Research, University of Leeds, Leeds, UK.; (Bennett MI) Academic Unit of Palliative Care, Leeds Institute of Health Sciences, University of Leeds, Leeds, UK.
(Allsop MJ) Academic Unit of Palliative Care, Leeds Institute of Health Sciences, University of Leeds, Leeds, UK.; (Wright-Hughes A) Clinical Trials Research Unit, Leeds Institute of Clinical Trials Research, University of Leeds, Leeds, UK.; (Black K) Academic Unit of Palliative Care, Leeds Institute of Health Sciences, University of Leeds, Leeds, UK.; (Hartley S) Clinical Trials Research Unit, Leeds Institute of Clinical Trials Research, University of Leeds, Leeds, UK.; (Fletcher M) Clinical Trials Research Unit, Leeds Institute of Clinical Trials Research, University of Leeds, Leeds, UK.; (Ziegler LE) Academic Unit of Palliative Care, Leeds Institute of Health Sciences, University of Leeds, Leeds, UK.; (Bewick BM) Division of Psychological and Social Medicine, Leeds Institute of Health Sciences, University of Leeds, Leeds, UK.; (Meads D) Academic Unit of Health Economics, Leeds Institute of Health Sciences, University of Leeds, Leeds, UK.; (Hughes ND) School of Healthcare, Baines Wing, University of Leeds, Leeds, UK.; (Closs SJ) School of Healthcare, Baines Wing, University of Leeds, Leeds, UK.; (Hulme C) Academic Unit of Health Economics, Leeds Institute of Health Sciences, University of Leeds, Leeds, UK.; (Taylor S) Academic Unit of Palliative Care, Leeds Institute of Health Sciences, University of Leeds, Leeds, UK.; (Flemming K) Department of Health Sciences, University of York, Heslington, UK.; (Hackett J) Academic Unit of Palliative Care, Leeds Institute of Health Sciences, University of Leeds, Leeds, UK.; (O'Dwyer JL) Academic Unit of Health Economics, Leeds Institute of Health Sciences, University of Leeds, Leeds, UK.; (Brown JM) Clinical Trials Research Unit, Leeds Institute of Clinical Trials Research, University of Leeds, Leeds, UK.; (Bennett MI) Academic Unit of Palliative Care, Leeds Institute of Health Sciences, University of Leeds, Leeds, UK.
(Allsop MJ) Academic Unit of Palliative Care, Leeds Institute of Health Sciences, University of Leeds, Leeds, UK.; (Wright-Hughes A) Clinical Trials Research Unit, Leeds Institute of Clinical Trials Research, University of Leeds, Leeds, UK.; (Black K) Academic Unit of Palliative Care, Leeds Institute of Health Sciences, University of Leeds, Leeds, UK.; (Hartley S) Clinical Trials Research Unit, Leeds Institute of Clinical Trials Research, University of Leeds, Leeds, UK.; (Fletcher M) Clinical Trials Research Unit, Leeds Institute of Clinical Trials Research, University of Leeds, Leeds, UK.; (Ziegler LE) Academic Unit of Palliative Care, Leeds Institute of Health Sciences, University of Leeds, Leeds, UK.; (Bewick BM) Division of Psychological and Social Medicine, Leeds Institute of Health Sciences, University of Leeds, Leeds, UK.; (Meads D) Academic Unit of Health Economics, Leeds Institute of Health Sciences, University of Leeds, Leeds, UK.; (Hughes ND) School of Healthcare, Baines Wing, University of Leeds, Leeds, UK.; (Closs SJ) School of Healthcare, Baines Wing, University of Leeds, Leeds, UK.; (Hulme C) Academic Unit of Health Economics, Leeds Institute of Health Sciences, University of Leeds, Leeds, UK.;

(Taylor S) Academic Unit of Palliative Care, Leeds Institute of Health Sciences, University of Leeds, Leeds, UK.; (Flemming K) Department of Health Sciences, University of York, Heslington, UK.; (Hackett J) Academic Unit of Palliative Care, Leeds Institute of Health Sciences, University of Leeds, Leeds, UK.; (O'Dwyer JL) Academic Unit of Health Economics, Leeds Institute of Health Sciences, University of Leeds, Leeds, UK.; (Brown JM) Clinical Trials Research Unit, Leeds Institute of Clinical Trials Research, University of Leeds, Leeds, UK.; (Bennett MI) Academic Unit of Palliative Care, Leeds Institute of Health Sciences, University of Leeds, Leeds, UK.

(Allsop MJ) Academic Unit of Palliative Care, Leeds Institute of Health Sciences, University of Leeds, Leeds, UK.; (Wright-Hughes A) Clinical Trials Research Unit, Leeds Institute of Clinical Trials Research, University of Leeds, Leeds, UK.; (Black K) Academic Unit of Palliative Care, Leeds Institute of Health Sciences, University of Leeds, Leeds, UK.; (Hartley S) Clinical Trials Research Unit, Leeds Institute of Clinical Trials Research, University of Leeds, Leeds, UK.; (Fletcher M) Clinical Trials Research Unit, Leeds Institute of Clinical Trials Research, University of Leeds, Leeds, UK.; (Ziegler LE) Academic Unit of Palliative Care, Leeds Institute of Health Sciences, University of Leeds, Leeds, UK.; (Bewick BM) Division of Psychological and Social Medicine, Leeds Institute of Health Sciences, University of Leeds, Leeds, UK.; (Meads D) Academic Unit of Health Economics, Leeds Institute of Health Sciences, University of Leeds, Leeds, UK.; (Hughes ND) School of Healthcare, Baines Wing, University of Leeds, Leeds, UK.; (Closs SJ) School of Healthcare, Baines Wing, University of Leeds, Leeds, UK.; (Hulme C) Academic Unit of Health Economics, Leeds Institute of Health Sciences, University of Leeds, Leeds, UK.; (Taylor S) Academic Unit of Palliative Care, Leeds Institute of Health Sciences, University of Leeds, Leeds, UK.; (Flemming K) Department of Health Sciences, University of York, Heslington, UK.; (Hackett J) Academic Unit of Palliative Care, Leeds Institute of Health Sciences, University of Leeds, Leeds, UK.; (O'Dwyer JL) Academic Unit of Health Economics, Leeds Institute of Health Sciences, University of Leeds, Leeds, UK.; (Brown JM) Clinical Trials Research Unit, Leeds Institute of Clinical Trials Research, University of Leeds, Leeds, UK.; (Bennett MI) Academic Unit of Palliative Care, Leeds Institute of Health Sciences, University of Leeds, Leeds, UK.

(Allsop MJ) Academic Unit of Palliative Care, Leeds Institute of Health Sciences, University of Leeds, Leeds, UK.; (Wright-Hughes A) Clinical Trials Research Unit, Leeds Institute of Clinical Trials Research, University of Leeds, Leeds, UK.; (Black K) Academic Unit of Palliative Care, Leeds Institute of Health Sciences, University of Leeds, Leeds, UK.; (Hartley S) Clinical Trials Research Unit, Leeds Institute of Clinical Trials Research, University of Leeds, Leeds, UK.; (Fletcher M) Clinical Trials Research Unit, Leeds Institute of Clinical Trials Research, University of Leeds, Leeds, UK.; (Ziegler LE) Academic Unit of Palliative Care, Leeds Institute of Health Sciences, University of Leeds, Leeds, UK.; (Bewick BM) Division of Psychological and Social Medicine, Leeds Institute of Health Sciences, University of Leeds, Leeds, UK.; (Meads D) Academic Unit of Health Economics, Leeds Institute of Health Sciences, University of Leeds, Leeds, UK.; (Hughes ND) School of Healthcare, Baines Wing, University of Leeds, Leeds, UK.; (Closs SJ) School of Healthcare, Baines Wing, University of Leeds, Leeds, UK.; (Hulme C) Academic Unit of Health Economics, Leeds Institute of Health Sciences, University of Leeds, Leeds, UK.; (Taylor S) Academic Unit of Palliative Care, Leeds Institute of Health Sciences, University of Leeds, Leeds, UK.; (Flemming K) Department of Health Sciences,

University of York, Heslington, UK.; (Hackett J) Academic Unit of Palliative Care, Leeds Institute of Health Sciences, University of Leeds, Leeds, UK.; (O'Dwyer JL) Academic Unit of Health Economics, Leeds Institute of Health Sciences, University of Leeds, Leeds, UK.; (Brown JM) Clinical Trials Research Unit, Leeds Institute of Clinical Trials Research, University of Leeds, Leeds, UK.; (Bennett MI) Academic Unit of Palliative Care, Leeds Institute of Health Sciences, University of Leeds, Leeds, UK.
(Allsop MJ) Academic Unit of Palliative Care, Leeds Institute of Health Sciences, University of Leeds, Leeds, UK.; (Wright-Hughes A) Clinical Trials Research Unit, Leeds Institute of Clinical Trials Research, University of Leeds, Leeds, UK.; (Black K) Academic Unit of Palliative Care, Leeds Institute of Health Sciences, University of Leeds, Leeds, UK.; (Hartley S) Clinical Trials Research Unit, Leeds Institute of Clinical Trials Research, University of Leeds, Leeds, UK.; (Fletcher M) Clinical Trials Research Unit, Leeds Institute of Clinical Trials Research, University of Leeds, Leeds, UK.; (Ziegler LE) Academic Unit of Palliative Care, Leeds Institute of Health Sciences, University of Leeds, Leeds, UK.; (Bewick BM) Division of Psychological and Social Medicine, Leeds Institute of Health Sciences, University of Leeds, Leeds, UK.; (Meads D) Academic Unit of Health Economics, Leeds Institute of Health Sciences, University of Leeds, Leeds, UK.; (Hughes ND) School of Healthcare, Baines Wing, University of Leeds, Leeds, UK.; (Closs SJ) School of Healthcare, Baines Wing, University of Leeds, Leeds, UK.; (Hulme C) Academic Unit of Health Economics, Leeds Institute of Health Sciences, University of Leeds, Leeds, UK.; (Taylor S) Academic Unit of Palliative Care, Leeds Institute of Health Sciences, University of Leeds, Leeds, UK.; (Flemming K) Department of Health Sciences, University of York, Heslington, UK.; (Hackett J) Academic Unit of Palliative Care, Leeds Institute of Health Sciences, University of Leeds, Leeds, UK.; (O'Dwyer JL) Academic Unit of Health Economics, Leeds Institute of Health Sciences, University of Leeds, Leeds, UK.; (Brown JM) Clinical Trials Research Unit, Leeds Institute of Clinical Trials Research, University of Leeds, Leeds, UK.; (Bennett MI) Academic Unit of Palliative Care, Leeds Institute of Health Sciences, University of Leeds, Leeds, UK.
(Allsop MJ) Academic Unit of Palliative Care, Leeds Institute of Health Sciences, University of Leeds, Leeds, UK.; (Wright-Hughes A) Clinical Trials Research Unit, Leeds Institute of Clinical Trials Research, University of Leeds, Leeds, UK.; (Black K) Academic Unit of Palliative Care, Leeds Institute of Health Sciences, University of Leeds, Leeds, UK.; (Hartley S) Clinical Trials Research Unit, Leeds Institute of Clinical Trials Research, University of Leeds, Leeds, UK.; (Fletcher M) Clinical Trials Research Unit, Leeds Institute of Clinical Trials Research, University of Leeds, Leeds, UK.; (Ziegler LE) Academic Unit of Palliative Care, Leeds Institute of Health Sciences, University of Leeds, Leeds, UK.; (Bewick BM) Division of Psychological and Social Medicine, Leeds Institute of Health Sciences, University of Leeds, Leeds, UK.; (Meads D) Academic Unit of Health Economics, Leeds Institute of Health Sciences, University of Leeds, Leeds, UK.; (Hughes ND) School of Healthcare, Baines Wing, University of Leeds, Leeds, UK.; (Closs SJ) School of Healthcare, Baines Wing, University of Leeds, Leeds, UK.; (Hulme C) Academic Unit of Health Economics, Leeds Institute of Health Sciences, University of Leeds, Leeds, UK.; (Taylor S) Academic Unit of Palliative Care, Leeds Institute of Health Sciences, University of Leeds, Leeds, UK.; (Flemming K) Department of Health Sciences, University of York, Heslington, UK.; (Hackett J) Academic Unit of Palliative Care, Leeds Institute of Health Sciences, University of Leeds, Leeds, UK.; (O'Dwyer JL)

Academic Unit of Health Economics, Leeds Institute of Health Sciences, University of Leeds, Leeds, UK.; (Brown JM) Clinical Trials Research Unit, Leeds Institute of Clinical Trials Research, University of Leeds, Leeds, UK.; (Bennett MI) Academic Unit of Palliative Care, Leeds Institute of Health Sciences, University of Leeds, Leeds, UK.
Database: PubMed

65. [Challenges for home care services in the pain management of cancer patients : A qualitative study].

Author(s): Gnass I; Krutter S; Nestler N

Source: Schmerz (Berlin, Germany); Oct 2018; vol. 32 (no. 5); p. 339-347

Publication Date: Oct 2018

Publication Type(s): Journal Article; Review

DOI: http://dx.doi.org/10.1007/s00482-018-0284-8

ISSN: 1432-2129

Place of Publication: Germany

PubMedID: 29564633

Accession Number: 29564633

Available at Schmerz (Berlin, Germany) - from EBSCO (MEDLINE Complete)

Available at Schmerz (Berlin, Germany) - from Unpaywall

Keywords: Subject Terms: Cancer pain; Home care service; Pain management; Palliative care; Qualitative research

Abstract:BACKGROUND: People with cancer are increasingly supported by home care services. Pain is a relevant symptom of these diseases and nurses of home care services are involved in the treatment. The German National Expert Standard "Pain management in nursing" includes evidence-based recommendations for the implementation of adequate pain management. Considering the given structural conditions of home care services, nurses describe both barriers and challenges with the implementation.METHODS: By means of five guideline-based discussion groups, nurses of 14 home care services were questioned about the challenges they had experienced in pain management. The questioning focuses on the level of implementation of the recommendation for each aspect: pain assessment, pharmacological pain therapy, non-pharmacological pain therapy, pain-related side effects, information, training, and counseling in the care of people with cancer. A qualitative content analysis was conducted.RESULTS: On the one hand, the results illustrate a need for further knowledge and possibilities, e.g., for the assessment of pain as a multidimensional phenomenon and, on the other hand, that the conditions for continuous pain monitoring of cancer patients in home care services are limited. The need for short-term reconciliation with the treatment team and the practitioners proved to be more difficult than the cooperation with the palliative care network. Involvement of family members is important to ensure uninterrupted treatment.CONCLUSIONS: Beside knowledge and competencies regarding nursing care, structures and processes for interprofessional pain management need further development and research.

Institutions:

(Gnass I) Institut für Pflegewissenschaft und -praxis, Paracelsus Medizinische Privatuniversität, Strubergasse 21, 5020, Salzburg, Österreich. irmela.gnass@pmu.ac.at.; (Krutter S) Institut für Pflegewissenschaft und -praxis, Paracelsus Medizinische Privatuniversität, Strubergasse 21, 5020, Salzburg, Österreich.; (Nestler N) Institut für Pflegewissenschaft und -praxis, Paracelsus Medizinische Privatuniversität, Strubergasse 21, 5020, Salzburg, Österreich.
(Gnass I) Institut für Pflegewissenschaft und -praxis, Paracelsus Medizinische Privatuniversität, Strubergasse 21, 5020, Salzburg, Österreich. irmela.gnass@pmu.ac.at.; (Krutter S) Institut für Pflegewissenschaft und -praxis, Paracelsus Medizinische Privatuniversität, Strubergasse 21, 5020, Salzburg, Österreich.; (Nestler N) Institut für Pflegewissenschaft und -praxis, Paracelsus Medizinische Privatuniversität, Strubergasse 21, 5020, Salzburg, Österreich.
(Gnass I) Institut für Pflegewissenschaft und -praxis, Paracelsus Medizinische Privatuniversität, Strubergasse 21, 5020, Salzburg, Österreich. irmela.gnass@pmu.ac.at.; (Krutter S) Institut für Pflegewissenschaft und -praxis, Paracelsus Medizinische Privatuniversität, Strubergasse 21, 5020, Salzburg, Österreich.; (Nestler N) Institut für Pflegewissenschaft und -praxis, Paracelsus Medizinische Privatuniversität, Strubergasse 21, 5020, Salzburg, Österreich.
Database: PubMed

66. The Use of Comfort Kits to Optimize Adult Cancer Pain Management.

Author(s): Blackburn LM; Abel S; Green L; Johnson K; Panda S

Source: Pain management nursing : official journal of the American Society of Pain Management Nurses; 2019; vol. 20 (no. 1); p. 25-31

Publication Date: 2019

Publication Type(s): Journal Article; Research Support, Non-U.S. Gov't

DOI: http://dx.doi.org/10.1016/j.pmn.2018.01.004

ISSN: 1532-8635

Place of Publication: United States

PubMedID: 29501361

Accession Number: 29501361

Abstract:BACKGROUND: Pain is one of the most feared of all symptoms for the cancer patient. Some studies estimate that up to 90% of all cancer patients experience pain. Advances in pharmaceuticals and expert provider knowledge have improved pain management overall for the patient with cancer; however, complementary therapies can synergize medications to provide optimal pain relief while decreasing the side effect profile. Despite this, nurses may have limited access to such resources. Many therapies can be administered directly by the bedside/chairside nurse with minimal training and the nurse can then teach the patient and family how to use the selected complementary therapy after leaving the hospital or clinic.OBJECTIVES: The oncology nurse will be able to identify several easy-to-implement complementary therapies that can supplement pharmacologic pain management for cancer patients.METHODS: As a quality project, comfort kits, containing such items as handheld massagers, guided imagery audiotapes, and aromatherapy essential oils, were distributed for use with patients through unit-based pain resource nurses.ANALYSIS: More than 500 comfort kit items were tracked by

the pain clinical nurse specialist during the comfort kit trial, both by medical record review and by follow-up phone calls to patients. During the comfort kit trial, average pain intensity decreased by 2.25 points on a 0-10 scale in the 24-hour period after use of the item from the comfort kit. Patients also had an overall decrease in the use of pharmacologic pain interventions and an increase in ambulation in the 24-hour period after implementation.CONCLUSIONS: Comfort kits allow nurses easy access to inexpensive tools to supplement pharmaceutical pain management. Optimizing nonpharmacologic pain management can increase patient and nurse satisfaction, improve overall pain management, and decrease untoward side effects.

Institutions:
(Blackburn LM) The Ohio State University Comprehensive Cancer Center, Arthur G. James Cancer Hospital and Richard J. Solove Research Institute, Columbus, Ohio. Electronic address: lisa.blackburn@osumc.edu.; (Abel S) Department of Pharmacy, The Ohio State University Comprehensive Cancer Center, Arthur G. James Cancer Hospital and Richard J. Solove Research Institute, Columbus, Ohio.; (Green L) Radiation Oncology Department, The Ohio State University Comprehensive Cancer Center, Arthur G. James Cancer Hospital and Richard J. Solove Research Institute, Columbus, Ohio.; (Johnson K) Cancer Program Analytics, The Ohio State University Comprehensive Cancer Center, Arthur G. James Cancer Hospital and Richard J. Solove Research Institute, Columbus, Ohio.; (Panda S) Medical Oncology Department, The Ohio State University Comprehensive Cancer Center, Arthur G. James Cancer Hospital and Richard J. Solove Research Institute, Columbus, Ohio.
(Blackburn LM) The Ohio State University Comprehensive Cancer Center, Arthur G. James Cancer Hospital and Richard J. Solove Research Institute, Columbus, Ohio. Electronic address: lisa.blackburn@osumc.edu.; (Abel S) Department of Pharmacy, The Ohio State University Comprehensive Cancer Center, Arthur G. James Cancer Hospital and Richard J. Solove Research Institute, Columbus, Ohio.; (Green L) Radiation Oncology Department, The Ohio State University Comprehensive Cancer Center, Arthur G. James Cancer Hospital and Richard J. Solove Research Institute, Columbus, Ohio.; (Johnson K) Cancer Program Analytics, The Ohio State University Comprehensive Cancer Center, Arthur G. James Cancer Hospital and Richard J. Solove Research Institute, Columbus, Ohio.; (Panda S) Medical Oncology Department, The Ohio State University Comprehensive Cancer Center, Arthur G. James Cancer Hospital and Richard J. Solove Research Institute, Columbus, Ohio.
(Blackburn LM) The Ohio State University Comprehensive Cancer Center, Arthur G. James Cancer Hospital and Richard J. Solove Research Institute, Columbus, Ohio. Electronic address: lisa.blackburn@osumc.edu.; (Abel S) Department of Pharmacy, The Ohio State University Comprehensive Cancer Center, Arthur G. James Cancer Hospital and Richard J. Solove Research Institute, Columbus, Ohio.; (Green L) Radiation Oncology Department, The Ohio State University Comprehensive Cancer Center, Arthur G. James Cancer Hospital and Richard J. Solove Research Institute, Columbus, Ohio.; (Johnson K) Cancer Program Analytics, The Ohio State University Comprehensive Cancer Center, Arthur G. James Cancer Hospital and Richard J. Solove Research Institute, Columbus, Ohio.; (Panda S) Medical Oncology Department, The Ohio State University Comprehensive Cancer Center, Arthur G. James Cancer Hospital and Richard J. Solove Research Institute, Columbus, Ohio.
(Blackburn LM) The Ohio State University Comprehensive Cancer Center, Arthur G. James Cancer Hospital and Richard J. Solove Research Institute, Columbus, Ohio. Electronic address: lisa.blackburn@osumc.edu.; (Abel S) Department of Pharmacy, The Ohio State University Comprehensive Cancer Center, Arthur G. James Cancer

Hospital and Richard J. Solove Research Institute, Columbus, Ohio.; (Green L) Radiation Oncology Department, The Ohio State University Comprehensive Cancer Center, Arthur G. James Cancer Hospital and Richard J. Solove Research Institute, Columbus, Ohio.; (Johnson K) Cancer Program Analytics, The Ohio State University Comprehensive Cancer Center, Arthur G. James Cancer Hospital and Richard J. Solove Research Institute, Columbus, Ohio.; (Panda S) Medical Oncology Department, The Ohio State University Comprehensive Cancer Center, Arthur G. James Cancer Hospital and Richard J. Solove Research Institute, Columbus, Ohio. (Blackburn LM) The Ohio State University Comprehensive Cancer Center, Arthur G. James Cancer Hospital and Richard J. Solove Research Institute, Columbus, Ohio. Electronic address: lisa.blackburn@osumc.edu.; (Abel S) Department of Pharmacy, The Ohio State University Comprehensive Cancer Center, Arthur G. James Cancer Hospital and Richard J. Solove Research Institute, Columbus, Ohio.; (Green L) Radiation Oncology Department, The Ohio State University Comprehensive Cancer Center, Arthur G. James Cancer Hospital and Richard J. Solove Research Institute, Columbus, Ohio.; (Johnson K) Cancer Program Analytics, The Ohio State University Comprehensive Cancer Center, Arthur G. James Cancer Hospital and Richard J. Solove Research Institute, Columbus, Ohio.; (Panda S) Medical Oncology Department, The Ohio State University Comprehensive Cancer Center, Arthur G. James Cancer Hospital and Richard J. Solove Research Institute, Columbus, Ohio.
Database: PubMed

67. How do patients with cancer pain view community pharmacy services? An interview study.

Author(s): Edwards Z; Blenkinsopp A; Ziegler L; Bennett MI

Source: Health & social care in the community; 2018; vol. 26 (no. 4); p. 507-518

Publication Date: 2018

Publication Type(s): Journal Article; Research Support, Non-U.S. Gov't

DOI: http://dx.doi.org/10.1111/hsc.12549

ISSN: 1365-2524

Place of Publication: England

PubMedID: 29479766

Accession Number: 29479766

Available at Health & Social Care in the Community - from Wiley Online Library Medicine and Nursing Collection 2020

Available at Health & Social Care in the Community - from EBSCO (MEDLINE Complete)

Available at Health & Social Care in the Community - from EBSCO (CINAHL Complete)

Available at Health & Social Care in the Community - from Unpaywall

Keywords: Subject Terms: *cancer; *community pharmacy; *medicines; *needs and experiences of cancer patients; *palliative care; *pharmacy practice research

Abstract: Pain experienced by many patients with advanced cancer is often not well controlled and community pharmacists are potentially well placed to provide support. The study objective was to explore the views and experiences of patients with

advanced cancer about community pharmacies, their services and attitudes towards having a community pharmacist pain medicines consultation. Purposive sampling of GP clinical information systems was used to recruit patients with advanced cancer, living in the community and receiving opioid analgesics in one area of England, UK between January 2015 and July 2016. Thirteen patients had a semi-structured interview which was audio-recorded and transcribed verbatim. Data were analysed deductively and inductively using Framework analysis and incorporating new themes as they emerged. The framework comprised Pain management, Experiences and expectations, Access to care and Communication. All patients reported using one regular community pharmacy citing convenience, service and staff friendliness as influential factors. The idea of a community pharmacy medicines consultation was acceptable to most patients. The idea of telephone consultations was positively received but electronic media such as Skype was not feasible or acceptable for most. Patients perceived a hierarchy of health professionals with specialist palliative care nurses at the top (due to their combined knowledge of their condition and medicines) followed by GPs then pharmacists. Patients receiving specialist palliative care described pain that was better controlled than those who were not. They thought medicines consultations with a pharmacist could be useful for patients before referral for palliative care. There is a need for pain medicines support for patients with advanced cancer, and unmet need appears greater for those not under the care of specialist services. Medicines consultations, in principle, are acceptable to patients both in person and by telephone, and the latter was perceived to be of particular benefit to patients less able to leave the house.

Institutions:
(Edwards Z) School of Pharmacy and Medical Sciences, University of Bradford, Bradford, UK.; (Blenkinsopp A) School of Pharmacy and Medical Sciences, University of Bradford, Bradford, UK.; (Ziegler L) Institute of Health Sciences, University of Leeds, Leeds, UK.; (Bennett MI) Institute of Health Sciences, University of Leeds, Leeds, UK.
(Edwards Z) School of Pharmacy and Medical Sciences, University of Bradford, Bradford, UK.; (Blenkinsopp A) School of Pharmacy and Medical Sciences, University of Bradford, Bradford, UK.; (Ziegler L) Institute of Health Sciences, University of Leeds, Leeds, UK.; (Bennett MI) Institute of Health Sciences, University of Leeds, Leeds, UK.
(Edwards Z) School of Pharmacy and Medical Sciences, University of Bradford, Bradford, UK.; (Blenkinsopp A) School of Pharmacy and Medical Sciences, University of Bradford, Bradford, UK.; (Ziegler L) Institute of Health Sciences, University of Leeds, Leeds, UK.; (Bennett MI) Institute of Health Sciences, University of Leeds, Leeds, UK.
(Edwards Z) School of Pharmacy and Medical Sciences, University of Bradford, Bradford, UK.; (Blenkinsopp A) School of Pharmacy and Medical Sciences, University of Bradford, Bradford, UK.; (Ziegler L) Institute of Health Sciences, University of Leeds, Leeds, UK.; (Bennett MI) Institute of Health Sciences, University of Leeds, Leeds, UK.

Database: PubMed

68. New frontier: cancer pain management clinical fellowship.

Author(s): Hochberg U; Perez J; Borod M

Source: Supportive care in cancer : official journal of the Multinational Association of Supportive Care in Cancer; Jul 2018; vol. 26 (no. 7); p. 2453-2457

Publication Date: Jul 2018

Publication Type(s): Journal Article

DOI: http://dx.doi.org/10.1007/s00520-018-4085-5

ISSN: 1433-7339

Place of Publication: Germany

PubMedID: 29429005

Accession Number: 29429005

Available at Supportive care in cancer : official journal of the Multinational Association of Supportive Care in Cancer - from SpringerLink

Available at Supportive care in cancer : official journal of the Multinational Association of Supportive Care in Cancer - from EBSCO (MEDLINE Complete)

Available at Supportive care in cancer : official journal of the Multinational Association of Supportive Care in Cancer - from EBSCO (CINAHL Complete)

Available at Supportive care in cancer : official journal of the Multinational Association of Supportive Care in Cancer - from ProQuest (MEDLINE with Full Text) - NHS Version

Available at Supportive care in cancer : official journal of the Multinational Association of Supportive Care in Cancer - from ProQuest (Health Research Premium) - NHS Version

Keywords: Subject Terms: Cancer pain; Fellowship; Interventional pain management; Medical education; Pain management

Abstract: Cancer pain is a multi-dimensional experience, varies from person to person both physically and psycho-socially, and impacts all aspects of the patients' quality of life. Majority of patients with an advanced or metastatic cancer will experience pain. It is estimated that as many as half of cancer patients are under-treated and as many as 20% experience pain refractory to the conventional WHO ladder of pain management. The McGill University Health Centre (MUHC) Cancer Pain Clinic (CPC) was created to meet the needs of those patients with a diagnosis of cancer whose pain had become a main symptom and those who failed to respond to conventional treatment. The clinic offers a unique interdisciplinary approach with a core team that includes an anesthesiologist, a palliative care physician, a radiation oncologist, a nurse clinician specialist in oncology and palliative care, and, recently, also an interventional radiologist. A cancer pain clinical fellowship was offered for the first time in July 2016. It provides intense training in the classification, epidemiology, pathophysiology, and treatment of cancer pain. Through our education program, the fellow learns to appreciate, weigh, and respond to the full spectrum of factors influencing a specific patient's condition and to develop a tailor-made care plan. To our knowledge, it is the only fellowship program in existence that focuses exclusively on cancer pain. We see it as a beacon and hope that our graduate fellows become professional leaders with a quest not only to provide the best possible care but also to raise awareness of the humanitarian need to control cancer pain.

Institutions:
(Hochberg U) McGill University, Montreal, Canada. urihochberg@hotmail.com.;
(Perez J) Cancer Pain Clinic, Division of Supportive and Palliative Care, McGill

University Health Centre, Montreal, QC, Canada.; (Borod M) Division of Supportive and Palliative Care, McGill University Health Centre, Montreal, QC, Canada. (Hochberg U) McGill University, Montreal, Canada. urihochberg@hotmail.com.; (Perez J) Cancer Pain Clinic, Division of Supportive and Palliative Care, McGill University Health Centre, Montreal, QC, Canada.; (Borod M) Division of Supportive and Palliative Care, McGill University Health Centre, Montreal, QC, Canada. (Hochberg U) McGill University, Montreal, Canada. urihochberg@hotmail.com.; (Perez J) Cancer Pain Clinic, Division of Supportive and Palliative Care, McGill University Health Centre, Montreal, QC, Canada.; (Borod M) Division of Supportive and Palliative Care, McGill University Health Centre, Montreal, QC, Canada.
Database: PubMed

69. Experiences of Urban African Americans with Cancer Pain.

Author(s): Maly A; Singh N; Vallerand AH

Source: Pain management nursing : official journal of the American Society of Pain Management Nurses; 2018; vol. 19 (no. 1); p. 72-78

Publication Date: 2018

Publication Type(s): Journal Article; Research Support, N.I.H., Extramural

DOI: http://dx.doi.org/10.1016/j.pmn.2017.11.007

ISSN: 1532-8635

Place of Publication: United States

PubMedID: 29422124

Accession Number: 29422124

Abstract: The experience of cancer pain is poorly understood from the perspective of African Americans, who experience higher levels of pain, more pain-related distress, and poorer function than Caucasians. Decreased perceived control over pain may play a greater role for African American patients, affecting pain-related distress and function. The purpose of this study was to add to the understanding of cancer pain and perceived control over pain in African Americans, from the patients' perspective. This qualitative inquiry was part of a larger mixed-methods study testing an intervention to improve pain, pain-related distress, and functional status through increasing perceived control over pain. Participants were recruited from the waiting room of an urban comprehensive cancer and interviewed in their homes. Interviews with 18 adult cancer patients who self-identified as African American and reported experiencing moderate to severe pain (>4 on a 0-10 scale) within the past two weeks were included. Qualitative interviews were audiotaped, transcribed, and analyzed using a constant comparative method. Two major themes emerged from this qualitative inquiry: struggles of the chronic pain experience and benefits of perceived control over pain. Each theme contained several categories. The study unveiled the participants account of both struggles of the chronic pain experience and barriers of perceived control that can be assessed for and targeted in nursing intervention. Benefits to having perceived control over pain were also illustrated in the participants' narratives.

Institutions:
(Maly A) Wayne State University College of Nursing, Detroit, Michigan. Electronic address: angelika.maly@wayne.edu.; (Singh N) Wayne State University College of

Nursing, Detroit, Michigan.; (Vallerand AH) Wayne State University College of Nursing, Detroit, Michigan.
(Maly A) Wayne State University College of Nursing, Detroit, Michigan. Electronic address: angelika.maly@wayne.edu.; (Singh N) Wayne State University College of Nursing, Detroit, Michigan.; (Vallerand AH) Wayne State University College of Nursing, Detroit, Michigan.
(Maly A) Wayne State University College of Nursing, Detroit, Michigan. Electronic address: angelika.maly@wayne.edu.; (Singh N) Wayne State University College of Nursing, Detroit, Michigan.; (Vallerand AH) Wayne State University College of Nursing, Detroit, Michigan.
Database: PubMed

70. Communicating Caregivers' Challenges With Cancer Pain Management: An Analysis of Home Hospice Visits.

Author(s): Han CJ; Chi NC; Han S; Demiris G; Parker-Oliver D; Washington K; Clayton MF; Reblin M; Ellington L

Source: Journal of pain and symptom management; 2018; vol. 55 (no. 5); p. 1296-1303

Publication Date: 2018

Publication Type(s): Journal Article; Research Support, N.I.H., Extramural

DOI: http://dx.doi.org/10.1016/j.jpainsymman.2018.01.004

ISSN: 1873-6513

Place of Publication: United States

PubMedID: 29360571

Accession Number: 29360571

Available at Journal of Pain & Symptom Management - from ScienceDirect

Available at Journal of Pain & Symptom Management - from Unpaywall

Keywords: Subject Terms: *Caregivers; *cancer; *health communication; *hospice; *pain management

Abstract:CONTEXT: Family caregivers (FCGs) of hospice cancer patients face significant challenges related to pain management. Addressing many of these challenges requires effective communication between FCGs and hospice nurses, yet little empirical evidence exists on the nature of communication about pain management between hospice nurses and FCGs.OBJECTIVES: We identified ways in which FCGs of hospice cancer patients communicated their pain management challenges to nurses during home visits and explored nurses' responses when pain management concerns were raised.METHODS: Using secondary data from audio recordings of hospice nurses' home visits, a deductive content analysis was conducted. We coded caregivers' pain management challenges and immediate nurses' responses to these challenges.RESULTS: From 63 hospice nurse visits, 101 statements describing caregivers' pain management challenges were identified. Thirty percent of these statements pertained to communication and teamwork issues. Twenty-seven percent concerned caregivers' medication skills and knowledge. In 52% of the cases, nurses responded to caregivers' pain management challenges with a validating statement. They provided information in 42% of the

cases. Nurses did not address 14% of the statements made by caregivers reflecting pain management challenges.CONCLUSION: To optimize hospice patients' comfort and reduce caregivers' anxiety and burden related to pain management, hospice nurses need to assess and address caregivers' pain management challenges during home visits. Communication and educational tools designed to reduce caregivers' barriers to pain management would likely improve clinical practice and both patient- and caregiver-related outcomes.

Institutions:

(Han CJ) Biobehavioral Cancer Prevention and Control Training Program, University of Washington, School of Public Health, Seattle, Washington, USA. Electronic address: jyh0908@uw.edu.; (Chi NC) University of Iowa, College of Nursing, Iowa City, Iowa, USA.; (Han S) University of Washington, School of Nursing, Seattle, Washington, USA.; (Demiris G) University of Pennsylvania, School of Nursing, Philadelphia, Pennsylvania, USA.; (Parker-Oliver D) University of Missouri, School of Medicine, Family and Community Medicine, Columbia, Missouri, USA.; (Washington K) University of Missouri, School of Medicine, Family and Community Medicine, Columbia, Missouri, USA.; (Clayton MF) University of Utah, College of Nursing, Salt Lake City, Utah, USA.; (Reblin M) Department of Health Outcomes and Behavior, Moffitt Cancer Center, Tampa, Florida, USA.; (Ellington L) University of Utah, College of Nursing, Salt Lake City, Utah, USA.
(Han CJ) Biobehavioral Cancer Prevention and Control Training Program, University of Washington, School of Public Health, Seattle, Washington, USA. Electronic address: jyh0908@uw.edu.; (Chi NC) University of Iowa, College of Nursing, Iowa City, Iowa, USA.; (Han S) University of Washington, School of Nursing, Seattle, Washington, USA.; (Demiris G) University of Pennsylvania, School of Nursing, Philadelphia, Pennsylvania, USA.; (Parker-Oliver D) University of Missouri, School of Medicine, Family and Community Medicine, Columbia, Missouri, USA.; (Washington K) University of Missouri, School of Medicine, Family and Community Medicine, Columbia, Missouri, USA.; (Clayton MF) University of Utah, College of Nursing, Salt Lake City, Utah, USA.; (Reblin M) Department of Health Outcomes and Behavior, Moffitt Cancer Center, Tampa, Florida, USA.; (Ellington L) University of Utah, College of Nursing, Salt Lake City, Utah, USA.
(Han CJ) Biobehavioral Cancer Prevention and Control Training Program, University of Washington, School of Public Health, Seattle, Washington, USA. Electronic address: jyh0908@uw.edu.; (Chi NC) University of Iowa, College of Nursing, Iowa City, Iowa, USA.; (Han S) University of Washington, School of Nursing, Seattle, Washington, USA.; (Demiris G) University of Pennsylvania, School of Nursing, Philadelphia, Pennsylvania, USA.; (Parker-Oliver D) University of Missouri, School of Medicine, Family and Community Medicine, Columbia, Missouri, USA.; (Washington K) University of Missouri, School of Medicine, Family and Community Medicine, Columbia, Missouri, USA.; (Clayton MF) University of Utah, College of Nursing, Salt Lake City, Utah, USA.; (Reblin M) Department of Health Outcomes and Behavior, Moffitt Cancer Center, Tampa, Florida, USA.; (Ellington L) University of Utah, College of Nursing, Salt Lake City, Utah, USA.
(Han CJ) Biobehavioral Cancer Prevention and Control Training Program, University of Washington, School of Public Health, Seattle, Washington, USA. Electronic address: jyh0908@uw.edu.; (Chi NC) University of Iowa, College of Nursing, Iowa City, Iowa, USA.; (Han S) University of Washington, School of Nursing, Seattle, Washington, USA.; (Demiris G) University of Pennsylvania, School of Nursing, Philadelphia, Pennsylvania, USA.; (Parker-Oliver D) University of Missouri, School of

Medicine, Family and Community Medicine, Columbia, Missouri, USA.; (Washington K) University of Missouri, School of Medicine, Family and Community Medicine, Columbia, Missouri, USA.; (Clayton MF) University of Utah, College of Nursing, Salt Lake City, Utah, USA.; (Reblin M) Department of Health Outcomes and Behavior, Moffitt Cancer Center, Tampa, Florida, USA.; (Ellington L) University of Utah, College of Nursing, Salt Lake City, Utah, USA.
(Han CJ) Biobehavioral Cancer Prevention and Control Training Program, University of Washington, School of Public Health, Seattle, Washington, USA. Electronic address: jyh0908@uw.edu.; (Chi NC) University of Iowa, College of Nursing, Iowa City, Iowa, USA.; (Han S) University of Washington, School of Nursing, Seattle, Washington, USA.; (Demiris G) University of Pennsylvania, School of Nursing, Philadelphia, Pennsylvania, USA.; (Parker-Oliver D) University of Missouri, School of Medicine, Family and Community Medicine, Columbia, Missouri, USA.; (Washington K) University of Missouri, School of Medicine, Family and Community Medicine, Columbia, Missouri, USA.; (Clayton MF) University of Utah, College of Nursing, Salt Lake City, Utah, USA.; (Reblin M) Department of Health Outcomes and Behavior, Moffitt Cancer Center, Tampa, Florida, USA.; (Ellington L) University of Utah, College of Nursing, Salt Lake City, Utah, USA.
(Han CJ) Biobehavioral Cancer Prevention and Control Training Program, University of Washington, School of Public Health, Seattle, Washington, USA. Electronic address: jyh0908@uw.edu.; (Chi NC) University of Iowa, College of Nursing, Iowa City, Iowa, USA.; (Han S) University of Washington, School of Nursing, Seattle, Washington, USA.; (Demiris G) University of Pennsylvania, School of Nursing, Philadelphia, Pennsylvania, USA.; (Parker-Oliver D) University of Missouri, School of Medicine, Family and Community Medicine, Columbia, Missouri, USA.; (Washington K) University of Missouri, School of Medicine, Family and Community Medicine, Columbia, Missouri, USA.; (Clayton MF) University of Utah, College of Nursing, Salt Lake City, Utah, USA.; (Reblin M) Department of Health Outcomes and Behavior, Moffitt Cancer Center, Tampa, Florida, USA.; (Ellington L) University of Utah, College of Nursing, Salt Lake City, Utah, USA.
(Han CJ) Biobehavioral Cancer Prevention and Control Training Program, University of Washington, School of Public Health, Seattle, Washington, USA. Electronic address: jyh0908@uw.edu.; (Chi NC) University of Iowa, College of Nursing, Iowa City, Iowa, USA.; (Han S) University of Washington, School of Nursing, Seattle, Washington, USA.; (Demiris G) University of Pennsylvania, School of Nursing, Philadelphia, Pennsylvania, USA.; (Parker-Oliver D) University of Missouri, School of Medicine, Family and Community Medicine, Columbia, Missouri, USA.; (Washington K) University of Missouri, School of Medicine, Family and Community Medicine, Columbia, Missouri, USA.; (Clayton MF) University of Utah, College of Nursing, Salt Lake City, Utah, USA.; (Reblin M) Department of Health Outcomes and Behavior, Moffitt Cancer Center, Tampa, Florida, USA.; (Ellington L) University of Utah, College of Nursing, Salt Lake City, Utah, USA.
(Han CJ) Biobehavioral Cancer Prevention and Control Training Program, University of Washington, School of Public Health, Seattle, Washington, USA. Electronic address: jyh0908@uw.edu.; (Chi NC) University of Iowa, College of Nursing, Iowa City, Iowa, USA.; (Han S) University of Washington, School of Nursing, Seattle, Washington, USA.; (Demiris G) University of Pennsylvania, School of Nursing, Philadelphia, Pennsylvania, USA.; (Parker-Oliver D) University of Missouri, School of Medicine, Family and Community Medicine, Columbia, Missouri, USA.; (Washington K) University of Missouri, School of Medicine, Family and Community Medicine,

Columbia, Missouri, USA.; (Clayton MF) University of Utah, College of Nursing, Salt Lake City, Utah, USA.; (Reblin M) Department of Health Outcomes and Behavior, Moffitt Cancer Center, Tampa, Florida, USA.; (Ellington L) University of Utah, College of Nursing, Salt Lake City, Utah, USA.
(Han CJ) Biobehavioral Cancer Prevention and Control Training Program, University of Washington, School of Public Health, Seattle, Washington, USA. Electronic address: jyh0908@uw.edu.; (Chi NC) University of Iowa, College of Nursing, Iowa City, Iowa, USA.; (Han S) University of Washington, School of Nursing, Seattle, Washington, USA.; (Demiris G) University of Pennsylvania, School of Nursing, Philadelphia, Pennsylvania, USA.; (Parker-Oliver D) University of Missouri, School of Medicine, Family and Community Medicine, Columbia, Missouri, USA.; (Washington K) University of Missouri, School of Medicine, Family and Community Medicine, Columbia, Missouri, USA.; (Clayton MF) University of Utah, College of Nursing, Salt Lake City, Utah, USA.; (Reblin M) Department of Health Outcomes and Behavior, Moffitt Cancer Center, Tampa, Florida, USA.; (Ellington L) University of Utah, College of Nursing, Salt Lake City, Utah, USA.
Database: PubMed

71. Knowledge and beliefs about chronic non cancer pain management for family medicine group nurses.

Author(s): Fernández Castillo RJ

Source: Enfermeria clinica (English Edition); Feb 2018

Publication Date: Feb 2018

Publication Type(s): Journal Article

DOI: http://dx.doi.org/10.1016/j.enfcli.2017.11.006

ISSN: 2445-1479

PubMedID: 29358065

Accession Number: 29358065

Institutions:
(Fernández Castillo RJ) Universidad de Sevilla, Sevilla, España. Electronic address: rafafernancastillo92@gmail.com.
Database: PubMed

72. Cancer pain management needs and perspectives of patients from Chinese backgrounds: a systematic review of the Chinese and English literature.

Author(s): Xu X; Luckett T; Wang AY; Lovell M; Phillips JL

Source: Palliative & supportive care; 2018; vol. 16 (no. 6); p. 785-799

Publication Date: 2018

Publication Type(s): Journal Article; Research Support, Non-U.S. Gov't; Systematic Review

DOI: http://dx.doi.org/10.1017/S1478951517001171

ISSN: 1478-9523

Place of Publication: England

PubMedID: 29338806

Accession Number: 29338806

Available at Palliative & supportive care - from Cambridge Journals Online

Available at Palliative & supportive care - from ProQuest (MEDLINE with Full Text) - NHS Version

Available at Palliative & supportive care - from ProQuest (Health Research Premium) - NHS Version

Available at Palliative & supportive care - from Unpaywall

Keywords: Subject Terms: *Cancer pain; *Chinese; *cultural influence; *pain management

Abstract:OBJECTIVE: More than half of all cancer patients experience unrelieved pain. Culture can significantly affect patients' cancer pain-related beliefs and behaviors. Little is known about cultural impact on Chinese cancer patients' pain management. The objective of this review was to describe pain management experiences of cancer patients from Chinese backgrounds and to identify barriers affecting their pain management.METHOD: A systematic review was conducted adhering to Preferred Reporting Items for Systematic Reviews and Meta-Analyses guidelines. Studies were included if they reported pain management experiences of adult cancer patients from Chinese backgrounds. Five databases were searched for peer-reviewed articles published in English or Chinese journals between1990 and 2015. The quality of included studies was assessed using Joanna Briggs Institution's appraisal tools.RESULTS: Of 3,904 identified records, 23 articles met criteria and provided primary data from 6,110 patients. Suboptimal analgesic use, delays in receiving treatment, reluctance to report pain, and/or poor adherence to prescribed analgesics contributed to the patients' inadequate pain control. Patient-related barriers included fatalism, desire to be good, low pain control belief, pain endurance beliefs, and negative effect beliefs. Patients and family shared barriers about fear of addiction and concerns on analgesic side effects and disease progression. Health professional-related barriers were poor communication, ineffective management of pain, and analgesic side effects. Healthcare system-related barriers included limited access to analgesics and/or after hour pain services and lack of health insurance.Significance of resultsChinese cancer patients' misconceptions regarding pain and analgesics may present as the main barriers to optimal pain relief. Findings of this review may inform health interventions to improve cancer pain management outcomes for patients from Chinese backgrounds. Future studies on patients' nonpharmacology intervention-related experiences are required to inform multidisciplinary and biopsychosocial approaches for culturally appropriate pain management.

Institutions:
(Xu X) Faculty of Health,University of Technology,Sydney,NSW,Australia.; (Luckett T) Faculty of Health,University of Technology,Sydney,NSW,Australia.; (Wang AY) Faculty of Health,University of Technology,Sydney,NSW,Australia.; (Lovell M) Greenwich Palliative and Supportive Care Services,Greenwich,NSW,Australia.; (Phillips JL) Faculty of Health,University of Technology,Sydney,NSW,Australia.
(Xu X) Faculty of Health,University of Technology,Sydney,NSW,Australia.; (Luckett T) Faculty of Health,University of Technology,Sydney,NSW,Australia.; (Wang AY) Faculty of Health,University of Technology,Sydney,NSW,Australia.; (Lovell M)

Greenwich Palliative and Supportive Care Services,Greenwich,NSW,Australia.; (Phillips JL) Faculty of Health,University of Technology,Sydney,NSW,Australia.
(Xu X) Faculty of Health,University of Technology,Sydney,NSW,Australia.; (Luckett T) Faculty of Health,University of Technology,Sydney,NSW,Australia.; (Wang AY) Faculty of Health,University of Technology,Sydney,NSW,Australia.; (Lovell M) Greenwich Palliative and Supportive Care Services,Greenwich,NSW,Australia.; (Phillips JL) Faculty of Health,University of Technology,Sydney,NSW,Australia.
(Xu X) Faculty of Health,University of Technology,Sydney,NSW,Australia.; (Luckett T) Faculty of Health,University of Technology,Sydney,NSW,Australia.; (Wang AY) Faculty of Health,University of Technology,Sydney,NSW,Australia.; (Lovell M) Greenwich Palliative and Supportive Care Services,Greenwich,NSW,Australia.; (Phillips JL) Faculty of Health,University of Technology,Sydney,NSW,Australia.
(Xu X) Faculty of Health,University of Technology,Sydney,NSW,Australia.; (Luckett T) Faculty of Health,University of Technology,Sydney,NSW,Australia.; (Wang AY) Faculty of Health,University of Technology,Sydney,NSW,Australia.; (Lovell M) Greenwich Palliative and Supportive Care Services,Greenwich,NSW,Australia.; (Phillips JL) Faculty of Health,University of Technology,Sydney,NSW,Australia.
Database: PubMed

73. Cancer Pain Management at Oncology Units: Comparing Knowledge, Attitudes and Perceived Barriers Between Physicians and Nurses.

Author(s): Darawad M; Alnajar MK; Abdalrahim MS; El-Aqoul AM

Source: Journal of cancer education : the official journal of the American Association for Cancer Education; 2019; vol. 34 (no. 2); p. 366-374

Publication Date: 2019

Publication Type(s): Journal Article

DOI: http://dx.doi.org/10.1007/s13187-017-1314-4

ISSN: 1543-0154

Place of Publication: England

PubMedID: 29288475

Accession Number: 29288475

Available at Journal of cancer education : the official journal of the American Association for Cancer Education - from EBSCO (MEDLINE Complete)

Available at Journal of cancer education : the official journal of the American Association for Cancer Education - from ProQuest (MEDLINE with Full Text) - NHS Version

Available at Journal of cancer education : the official journal of the American Association for Cancer Education - from ProQuest (Health Research Premium) - NHS Version

Keywords: Subject Terms: *Barriers; *Cancer pain management; *Jordan; *Knowledge and attitudes; *Nurses; *Oncology; *Physicians

Abstract: Pain is a major symptom that causes suffering among patients diagnosed with cancer. Identifying physicians' and nurses' knowledge, attitudes, and their perceived barriers of cancer pain management is considered an essential step in improving cancer pain relief. The purposes of this study are to compare physicians'

and nurses' knowledge and attitudes toward cancer pain management (CPM) and describe their perceived barriers to CPM at oncology units. A descriptive cross-sectional design was utilized to obtain data through self-report questionnaire. The total number of sample size was 207 participants (72 physicians and 135 nurses). Findings revealed that both physicians and nurses had fair knowledge and attitudes toward CPM. Physicians had significantly higher knowledge and better attitudes than nurses (62.3 vs. 51.5%, respectively). Physicians were knowledgeable about pharmacological pain management and opioid addiction but had negative attitudes toward pain assessment. Nurses' knowledge was better in regard of CPM guidelines, while they had poor knowledge about pharmacological pain management and opioid addiction. Physicians and nurses perceived knowledge deficit, lack of pain assessment, opioid unavailability, and lack of psychological interventions as the most common barriers to CPM. It is recommended to integrate recent evidence-based guidelines about CPM in oncology units that aim to improve practice. Offering continuing education courses in hospitals guided by pain teams is another essential recommendation for effective CPM.

Institutions:
(Darawad M) Clinical Nursing Department, School of Nursing-The University of Jordan, Amman, 11942, Jordan. m.darawad@ju.edu.jo.; (Alnajar MK) Clinical Nursing Department, School of Nursing-The University of Jordan, Amman, 11942, Jordan.; (Abdalrahim MS) Clinical Nursing Department, School of Nursing-The University of Jordan, Amman, 11942, Jordan.; (El-Aqoul AM) King Hussein Cancer Center, Queen Rania Al-Abdullah Street, P.O. Box 1269, Al-Jubeiha, Amman, 11941, Jordan.
(Darawad M) Clinical Nursing Department, School of Nursing-The University of Jordan, Amman, 11942, Jordan. m.darawad@ju.edu.jo.; (Alnajar MK) Clinical Nursing Department, School of Nursing-The University of Jordan, Amman, 11942, Jordan.; (Abdalrahim MS) Clinical Nursing Department, School of Nursing-The University of Jordan, Amman, 11942, Jordan.; (El-Aqoul AM) King Hussein Cancer Center, Queen Rania Al-Abdullah Street, P.O. Box 1269, Al-Jubeiha, Amman, 11941, Jordan.
(Darawad M) Clinical Nursing Department, School of Nursing-The University of Jordan, Amman, 11942, Jordan. m.darawad@ju.edu.jo.; (Alnajar MK) Clinical Nursing Department, School of Nursing-The University of Jordan, Amman, 11942, Jordan.; (Abdalrahim MS) Clinical Nursing Department, School of Nursing-The University of Jordan, Amman, 11942, Jordan.; (El-Aqoul AM) King Hussein Cancer Center, Queen Rania Al-Abdullah Street, P.O. Box 1269, Al-Jubeiha, Amman, 11941, Jordan.
(Darawad M) Clinical Nursing Department, School of Nursing-The University of Jordan, Amman, 11942, Jordan. m.darawad@ju.edu.jo.; (Alnajar MK) Clinical Nursing Department, School of Nursing-The University of Jordan, Amman, 11942, Jordan.; (Abdalrahim MS) Clinical Nursing Department, School of Nursing-The University of Jordan, Amman, 11942, Jordan.; (El-Aqoul AM) King Hussein Cancer Center, Queen Rania Al-Abdullah Street, P.O. Box 1269, Al-Jubeiha, Amman, 11941, Jordan.
Database: PubMed

74. Update in Hospital Palliative Care: Symptom Management, Communication, Caregiver Outcomes, and Moral Distress.

Author(s): Havyer RD; Pomerantz DH; Jayes RL; Harris PF; Harman SM; Ansari AA
Source: Journal of hospital medicine; ; vol. 13 (no. 6); p. 419-423
Publication Type(s): Journal Article; Review
DOI: http://dx.doi.org/10.12788/jhm.2895
ISSN: 1553-5606
Place of Publication: United States
PubMedID: 29261818
Accession Number: 29261818
Available at Journal of hospital medicine - from Unpaywall
Abstract:BACKGROUND: Updated knowledge of the palliative care (PC) literature is needed to maintain competency and best address the PC needs of hospitalized patients. We critiqued the recent PC literature with the highest potential to impact hospital practice.METHODS: We reviewed articles published between January 2016 and December 2016, which were identified through a handsearch of leading journals and a MEDLINE search. The final 9 articles selected were determined by consensus based on scientific rigor, relevance to hospital medicine, and impact on practice.RESULTS: Key findings include the following: scheduled antipsychotics were inferior to a placebo for nonterminal delirium; a low-dose morphine was superior to a weak opioid for moderate cancer pain; methadone as a coanalgesic improved high-intensity cancer pain; many hospitalized patients on comfort care still receive antimicrobials; video decision aids improved the rates of advance care planning (ACP) and hospice use and decreased costs; standardized, PC-led intervention did not improve psychological outcomes in families of patients with a chronic critical illness; caregivers of patients surviving a prolonged critical illness experienced high and persistent rates of depression; people with non-normative sexuality or gender faced additional stressors with partner loss; and physician trainees experienced significant moral distress with futile treatments.CONCLUSIONS: Recent research provides important guidance for clinicians caring for hospitalized patients with serious illnesses, including symptom management, ACP, moral distress, and outcomes of critical illness.
Institutions:
(Havyer RD) Division of Primary Care Internal Medicine and Center for Palliative Medicine, Mayo Clinic, Rochester, Minnesota, USA.; (Pomerantz DH) Division of General Internal Medicine and Department of Family Medicine (Palliative Care), Albert Einstein College of Medicine, Bronx, New York, and Department of Medicine, Montefiore New Rochelle Hospital, New Rochelle, New York, USA.; (Jayes RL) Division of Geriatrics and Palliative Medicine, George Washington University Medical Faculty Associates, Washington, D.C., USA.; (Harris PF) Division of Geriatrics, Department of Medicine, David Geffen School of Medicine, University of California, Los Angeles, Los Angeles, California, USA.; (Harman SM) Department of Medicine, School of Medicine, Stanford University, Stanford, California, USA.; (Ansari AA) Division of Hospital Medicine, Loyola University Medical Center, Maywood, Illinois, USA. aansar1@lumc.edu.
(Havyer RD) Division of Primary Care Internal Medicine and Center for Palliative Medicine, Mayo Clinic, Rochester, Minnesota, USA.; (Pomerantz DH) Division of General Internal Medicine and Department of Family Medicine (Palliative Care), Albert Einstein College of Medicine, Bronx, New York, and Department of Medicine,

Montefiore New Rochelle Hospital, New Rochelle, New York, USA.; (Jayes RL) Division of Geriatrics and Palliative Medicine, George Washington University Medical Faculty Associates, Washington, D.C., USA.; (Harris PF) Division of Geriatrics, Department of Medicine, David Geffen School of Medicine, University of California, Los Angeles, Los Angeles, California, USA.; (Harman SM) Department of Medicine, School of Medicine, Stanford University, Stanford, California, USA.; (Ansari AA) Division of Hospital Medicine, Loyola University Medical Center, Maywood, Illinois, USA. aansar1@lumc.edu.
(Havyer RD) Division of Primary Care Internal Medicine and Center for Palliative Medicine, Mayo Clinic, Rochester, Minnesota, USA.; (Pomerantz DH) Division of General Internal Medicine and Department of Family Medicine (Palliative Care), Albert Einstein College of Medicine, Bronx, New York, and Department of Medicine, Montefiore New Rochelle Hospital, New Rochelle, New York, USA.; (Jayes RL) Division of Geriatrics and Palliative Medicine, George Washington University Medical Faculty Associates, Washington, D.C., USA.; (Harris PF) Division of Geriatrics, Department of Medicine, David Geffen School of Medicine, University of California, Los Angeles, Los Angeles, California, USA.; (Harman SM) Department of Medicine, School of Medicine, Stanford University, Stanford, California, USA.; (Ansari AA) Division of Hospital Medicine, Loyola University Medical Center, Maywood, Illinois, USA. aansar1@lumc.edu.
(Havyer RD) Division of Primary Care Internal Medicine and Center for Palliative Medicine, Mayo Clinic, Rochester, Minnesota, USA.; (Pomerantz DH) Division of General Internal Medicine and Department of Family Medicine (Palliative Care), Albert Einstein College of Medicine, Bronx, New York, and Department of Medicine, Montefiore New Rochelle Hospital, New Rochelle, New York, USA.; (Jayes RL) Division of Geriatrics and Palliative Medicine, George Washington University Medical Faculty Associates, Washington, D.C., USA.; (Harris PF) Division of Geriatrics, Department of Medicine, David Geffen School of Medicine, University of California, Los Angeles, Los Angeles, California, USA.; (Harman SM) Department of Medicine, School of Medicine, Stanford University, Stanford, California, USA.; (Ansari AA) Division of Hospital Medicine, Loyola University Medical Center, Maywood, Illinois, USA. aansar1@lumc.edu.
(Havyer RD) Division of Primary Care Internal Medicine and Center for Palliative Medicine, Mayo Clinic, Rochester, Minnesota, USA.; (Pomerantz DH) Division of General Internal Medicine and Department of Family Medicine (Palliative Care), Albert Einstein College of Medicine, Bronx, New York, and Department of Medicine, Montefiore New Rochelle Hospital, New Rochelle, New York, USA.; (Jayes RL) Division of Geriatrics and Palliative Medicine, George Washington University Medical Faculty Associates, Washington, D.C., USA.; (Harris PF) Division of Geriatrics, Department of Medicine, David Geffen School of Medicine, University of California, Los Angeles, Los Angeles, California, USA.; (Harman SM) Department of Medicine, School of Medicine, Stanford University, Stanford, California, USA.; (Ansari AA) Division of Hospital Medicine, Loyola University Medical Center, Maywood, Illinois, USA. aansar1@lumc.edu.
(Havyer RD) Division of Primary Care Internal Medicine and Center for Palliative Medicine, Mayo Clinic, Rochester, Minnesota, USA.; (Pomerantz DH) Division of General Internal Medicine and Department of Family Medicine (Palliative Care), Albert Einstein College of Medicine, Bronx, New York, and Department of Medicine, Montefiore New Rochelle Hospital, New Rochelle, New York, USA.; (Jayes RL) Division of Geriatrics and Palliative Medicine, George Washington University Medical

Faculty Associates, Washington, D.C., USA.; (Harris PF) Division of Geriatrics, Department of Medicine, David Geffen School of Medicine, University of California, Los Angeles, Los Angeles, California, USA.; (Harman SM) Department of Medicine, School of Medicine, Stanford University, Stanford, California, USA.; (Ansari AA) Division of Hospital Medicine, Loyola University Medical Center, Maywood, Illinois, USA. aansar1@lumc.edu.
Database: PubMed

75. Cancer-related pain: a nationwide survey of patients' treatment modification and satisfaction in Taiwan.

Author(s): Rau KM; Chen JS; Wu HB; Lin SF; Huang ML; Tai CJ; Hwang WL; Lu YC; Wang CC; Kuen Hsieh R

Source: Japanese journal of clinical oncology; Nov 2017; vol. 47 (no. 11); p. 1060-1065

Publication Date: Nov 2017

Publication Type(s): Journal Article

DOI: http://dx.doi.org/10.1093/jjco/hyx124

ISSN: 1465-3621

Place of Publication: England

PubMedID: 28973687

Accession Number: 28973687

Available at Japanese journal of clinical oncology - from HighWire - Free Full Text

Available at Japanese journal of clinical oncology - from Unpaywall

Keywords: Subject Terms: cancer pain; guidelines; outpatient department; pain control; quality of life; satisfaction

Abstract: Background: We have limited knowledge about cancer patients' pain control satisfaction in outpatient departments in Taiwan and doctors' practice of adjusting analgesics according to their pain status. This survey examined pain management and satisfaction among cancer outpatients with pain and obtained information on their quality of life and treatment management for different pain intensities.Methods: The Short version of the Brief Pain Inventory was used as the outcome questionnaire. Participants comprised 2075 patients with different cancers and disease statuses at 14 oncological outpatient departments, of which 1051 reported pain within the week prior to testing. The impact of pain management on physical and psychological functioning, and satisfaction with doctors were evaluated. Information about doctors' prescriptions was collected. Logistic regression analyses were conducted to evaluate whether the interference scale performed identically in the different analgesic ladders.Results: Pain was significantly linked to disease status and affected patients' physical and psychiatric functioning. Almost 100% of patients were satisfied with their pain control, but more than 70% of doctors did not change analgesics based on patients' current pain status. The results show that although patients were satisfied with their physicians, treatment of cancer pain was still suboptimal.Conclusion: Pain assessment and treatment need to be more thorough and management guidelines should be revised to improve pain control in patients with cancer.

Institutions:
(Rau KM) Division of Hematology-Oncology, Department of Internal Medicine, Kaohsiung Chang Gung Memorial Hospital, Kaohsiung.; (Chen JS) Chang Gung University, College of Medicine.; (Wu HB) Division of Hematology Oncology, Department of Internal Medicine, Taipei City Hospital, Renai branch, Taipei.; (Lin SF) Faculty of Medicine, College of Medicine, Kaohsiung Medical University, Kaohsiung.; (Huang ML) Division of Oncology, Department of Oncology and Cancer Study, Da Chien Hospital, Miaoli.; (Tai CJ) Division of Hematology and Oncology, Department of Internal Medicine, Taipei Medicine University Hospital, Taipei, Taiwan.; (Hwang WL) Division of Hematology/Medical Oncology, Department of Medicine, Taichung Veterans General Hospital, Taichung.; (Lu YC) Division of Hematology-Oncology, Ditmanson Medical Foundation Chia-Yi Christian Hospital, Chia-Yi.; (Wang CC) Division of Medical Oncology in the Hematology-Oncology, Department of Internal Medicine, Changhua Christian Hospital, Changhua.; (Kuen Hsieh R) Division of Hematology and Oncology, Department of Internal Medicine, Mackay Memorial Hospital, Taiwan.
(Rau KM) Division of Hematology-Oncology, Department of Internal Medicine, Kaohsiung Chang Gung Memorial Hospital, Kaohsiung.; (Chen JS) Chang Gung University, College of Medicine.; (Wu HB) Division of Hematology Oncology, Department of Internal Medicine, Taipei City Hospital, Renai branch, Taipei.; (Lin SF) Faculty of Medicine, College of Medicine, Kaohsiung Medical University, Kaohsiung.; (Huang ML) Division of Oncology, Department of Oncology and Cancer Study, Da Chien Hospital, Miaoli.; (Tai CJ) Division of Hematology and Oncology, Department of Internal Medicine, Taipei Medicine University Hospital, Taipei, Taiwan.; (Hwang WL) Division of Hematology/Medical Oncology, Department of Medicine, Taichung Veterans General Hospital, Taichung.; (Lu YC) Division of Hematology-Oncology, Ditmanson Medical Foundation Chia-Yi Christian Hospital, Chia-Yi.; (Wang CC) Division of Medical Oncology in the Hematology-Oncology, Department of Internal Medicine, Changhua Christian Hospital, Changhua.; (Kuen Hsieh R) Division of Hematology and Oncology, Department of Internal Medicine, Mackay Memorial Hospital, Taiwan.
(Rau KM) Division of Hematology-Oncology, Department of Internal Medicine, Kaohsiung Chang Gung Memorial Hospital, Kaohsiung.; (Chen JS) Chang Gung University, College of Medicine.; (Wu HB) Division of Hematology Oncology, Department of Internal Medicine, Taipei City Hospital, Renai branch, Taipei.; (Lin SF) Faculty of Medicine, College of Medicine, Kaohsiung Medical University, Kaohsiung.; (Huang ML) Division of Oncology, Department of Oncology and Cancer Study, Da Chien Hospital, Miaoli.; (Tai CJ) Division of Hematology and Oncology, Department of Internal Medicine, Taipei Medicine University Hospital, Taipei, Taiwan.; (Hwang WL) Division of Hematology/Medical Oncology, Department of Medicine, Taichung Veterans General Hospital, Taichung.; (Lu YC) Division of Hematology-Oncology, Ditmanson Medical Foundation Chia-Yi Christian Hospital, Chia-Yi.; (Wang CC) Division of Medical Oncology in the Hematology-Oncology, Department of Internal Medicine, Changhua Christian Hospital, Changhua.; (Kuen Hsieh R) Division of Hematology and Oncology, Department of Internal Medicine, Mackay Memorial Hospital, Taiwan.
(Rau KM) Division of Hematology-Oncology, Department of Internal Medicine, Kaohsiung Chang Gung Memorial Hospital, Kaohsiung.; (Chen JS) Chang Gung University, College of Medicine.; (Wu HB) Division of Hematology Oncology, Department of Internal Medicine, Taipei City Hospital, Renai branch, Taipei.; (Lin SF)

Faculty of Medicine, College of Medicine, Kaohsiung Medical University, Kaohsiung.; (Huang ML) Division of Oncology, Department of Oncology and Cancer Study, Da Chien Hospital, Miaoli.; (Tai CJ) Division of Hematology and Oncology, Department of Internal Medicine, Taipei Medicine University Hospital, Taipei, Taiwan.; (Hwang WL) Division of Hematology/Medical Oncology, Department of Medicine, Taichung Veterans General Hospital, Taichung.; (Lu YC) Division of Hematology-Oncology, Ditmanson Medical Foundation Chia-Yi Christian Hospital, Chia-Yi.; (Wang CC) Division of Medical Oncology in the Hematology-Oncology, Department of Internal Medicine, Changhua Christian Hospital, Changhua.; (Kuen Hsieh R) Division of Hematology and Oncology, Department of Internal Medicine, Mackay Memorial Hospital, Taiwan.
(Rau KM) Division of Hematology-Oncology, Department of Internal Medicine, Kaohsiung Chang Gung Memorial Hospital, Kaohsiung.; (Chen JS) Chang Gung University, College of Medicine.; (Wu HB) Division of Hematology Oncology, Department of Internal Medicine, Taipei City Hospital, Renai branch, Taipei.; (Lin SF)
Faculty of Medicine, College of Medicine, Kaohsiung Medical University, Kaohsiung.; (Huang ML) Division of Oncology, Department of Oncology and Cancer Study, Da Chien Hospital, Miaoli.; (Tai CJ) Division of Hematology and Oncology, Department of Internal Medicine, Taipei Medicine University Hospital, Taipei, Taiwan.; (Hwang WL) Division of Hematology/Medical Oncology, Department of Medicine, Taichung Veterans General Hospital, Taichung.; (Lu YC) Division of Hematology-Oncology, Ditmanson Medical Foundation Chia-Yi Christian Hospital, Chia-Yi.; (Wang CC) Division of Medical Oncology in the Hematology-Oncology, Department of Internal Medicine, Changhua Christian Hospital, Changhua.; (Kuen Hsieh R) Division of Hematology and Oncology, Department of Internal Medicine, Mackay Memorial Hospital, Taiwan.
(Rau KM) Division of Hematology-Oncology, Department of Internal Medicine, Kaohsiung Chang Gung Memorial Hospital, Kaohsiung.; (Chen JS) Chang Gung University, College of Medicine.; (Wu HB) Division of Hematology Oncology, Department of Internal Medicine, Taipei City Hospital, Renai branch, Taipei.; (Lin SF) Faculty of Medicine, College of Medicine, Kaohsiung Medical University, Kaohsiung.; (Huang ML) Division of Oncology, Department of Oncology and Cancer Study, Da Chien Hospital, Miaoli.; (Tai CJ) Division of Hematology and Oncology, Department of Internal Medicine, Taipei Medicine University Hospital, Taipei, Taiwan.; (Hwang WL) Division of Hematology/Medical Oncology, Department of Medicine, Taichung Veterans General Hospital, Taichung.; (Lu YC) Division of Hematology-Oncology, Ditmanson Medical Foundation Chia-Yi Christian Hospital, Chia-Yi.; (Wang CC) Division of Medical Oncology in the Hematology-Oncology, Department of Internal Medicine, Changhua Christian Hospital, Changhua.; (Kuen Hsieh R) Division of Hematology and Oncology, Department of Internal Medicine, Mackay Memorial Hospital, Taiwan.
(Rau KM) Division of Hematology-Oncology, Department of Internal Medicine, Kaohsiung Chang Gung Memorial Hospital, Kaohsiung.; (Chen JS) Chang Gung University, College of Medicine.; (Wu HB) Division of Hematology Oncology, Department of Internal Medicine, Taipei City Hospital, Renai branch, Taipei.; (Lin SF) Faculty of Medicine, College of Medicine, Kaohsiung Medical University, Kaohsiung.; (Huang ML) Division of Oncology, Department of Oncology and Cancer Study, Da Chien Hospital, Miaoli.; (Tai CJ) Division of Hematology and Oncology, Department of Internal Medicine, Taipei Medicine University Hospital, Taipei, Taiwan.; (Hwang WL) Division of Hematology/Medical Oncology, Department of Medicine, Taichung

Veterans General Hospital, Taichung.; (Lu YC) Division of Hematology-Oncology, Ditmanson Medical Foundation Chia-Yi Christian Hospital, Chia-Yi.; (Wang CC) Division of Medical Oncology in the Hematology-Oncology, Department of Internal Medicine, Changhua Christian Hospital, Changhua.; (Kuen Hsieh R) Division of Hematology and Oncology, Department of Internal Medicine, Mackay Memorial Hospital, Taiwan.
(Rau KM) Division of Hematology-Oncology, Department of Internal Medicine, Kaohsiung Chang Gung Memorial Hospital, Kaohsiung.; (Chen JS) Chang Gung University, College of Medicine.; (Wu HB) Division of Hematology Oncology, Department of Internal Medicine, Taipei City Hospital, Renai branch, Taipei.; (Lin SF) Faculty of Medicine, College of Medicine, Kaohsiung Medical University, Kaohsiung.; (Huang ML) Division of Oncology, Department of Oncology and Cancer Study, Da Chien Hospital, Miaoli.; (Tai CJ) Division of Hematology and Oncology, Department of Internal Medicine, Taipei Medicine University Hospital, Taipei, Taiwan.; (Hwang WL) Division of Hematology/Medical Oncology, Department of Medicine, Taichung Veterans General Hospital, Taichung.; (Lu YC) Division of Hematology-Oncology, Ditmanson Medical Foundation Chia-Yi Christian Hospital, Chia-Yi.; (Wang CC) Division of Medical Oncology in the Hematology-Oncology, Department of Internal Medicine, Changhua Christian Hospital, Changhua.; (Kuen Hsieh R) Division of Hematology and Oncology, Department of Internal Medicine, Mackay Memorial Hospital, Taiwan.
(Rau KM) Division of Hematology-Oncology, Department of Internal Medicine, Kaohsiung Chang Gung Memorial Hospital, Kaohsiung.; (Chen JS) Chang Gung University, College of Medicine.; (Wu HB) Division of Hematology Oncology, Department of Internal Medicine, Taipei City Hospital, Renai branch, Taipei.; (Lin SF) Faculty of Medicine, College of Medicine, Kaohsiung Medical University, Kaohsiung.; (Huang ML) Division of Oncology, Department of Oncology and Cancer Study, Da Chien Hospital, Miaoli.; (Tai CJ) Division of Hematology and Oncology, Department of Internal Medicine, Taipei Medicine University Hospital, Taipei, Taiwan.; (Hwang WL) Division of Hematology/Medical Oncology, Department of Medicine, Taichung Veterans General Hospital, Taichung.; (Lu YC) Division of Hematology-Oncology, Ditmanson Medical Foundation Chia-Yi Christian Hospital, Chia-Yi.; (Wang CC) Division of Medical Oncology in the Hematology-Oncology, Department of Internal Medicine, Changhua Christian Hospital, Changhua.; (Kuen Hsieh R) Division of Hematology and Oncology, Department of Internal Medicine, Mackay Memorial Hospital, Taiwan.
(Rau KM) Division of Hematology-Oncology, Department of Internal Medicine, Kaohsiung Chang Gung Memorial Hospital, Kaohsiung.; (Chen JS) Chang Gung University, College of Medicine.; (Wu HB) Division of Hematology Oncology, Department of Internal Medicine, Taipei City Hospital, Renai branch, Taipei.; (Lin SF) Faculty of Medicine, College of Medicine, Kaohsiung Medical University, Kaohsiung.; (Huang ML) Division of Oncology, Department of Oncology and Cancer Study, Da Chien Hospital, Miaoli.; (Tai CJ) Division of Hematology and Oncology, Department of Internal Medicine, Taipei Medicine University Hospital, Taipei, Taiwan.; (Hwang WL) Division of Hematology/Medical Oncology, Department of Medicine, Taichung Veterans General Hospital, Taichung.; (Lu YC) Division of Hematology-Oncology, Ditmanson Medical Foundation Chia-Yi Christian Hospital, Chia-Yi.; (Wang CC) Division of Medical Oncology in the Hematology-Oncology, Department of Internal Medicine, Changhua Christian Hospital, Changhua.; (Kuen Hsieh R) Division of

Hematology and Oncology, Department of Internal Medicine, Mackay Memorial Hospital, Taiwan.
Database: PubMed

76. Knowledge and Attitudes Toward Cancer Pain Management Among Nurses at Oncology Units.

Author(s): Alnajar MK; Darawad MW; Alshahwan SS; Samarkandi OA

Source: Journal of cancer education : the official journal of the American Association for Cancer Education; 2019; vol. 34 (no. 1); p. 186-193

Publication Date: 2019

Publication Type(s): Journal Article; Research Support, Non-U.S. Gov't

DOI: http://dx.doi.org/10.1007/s13187-017-1285-5

ISSN: 1543-0154

Place of Publication: England

PubMedID: 28944405

Accession Number: 28944405

Available at Journal of cancer education : the official journal of the American Association for Cancer Education - from EBSCO (MEDLINE Complete)

Available at Journal of cancer education : the official journal of the American Association for Cancer Education - from ProQuest (MEDLINE with Full Text) - NHS Version

Available at Journal of cancer education : the official journal of the American Association for Cancer Education - from ProQuest (Health Research Premium) - NHS Version

Keywords: Subject Terms: *Attitudes; *Cancer pain; *Jordan; *Knowledge; *Nurses; *Oncology; *Pain management

Abstract: Nurses have major responsibilities to treat cancer pain in an optimal way. Their knowledge and attitudes are the key to success cancer pain management (CPM) process and impact the outcomes of pain treatment. This study aimed to evaluate the knowledge and attitudes toward CPM among Jordanian nurses working at oncology units. A cross-sectional descriptive design was used to collect data from 135 nurses who were working at four oncology units using Knowledge and Attitudes Survey Regarding Pain. The percentage of correct answers was 51.5% indicating that participants had fair knowledge and attitudes toward CPM. Nurses appeared knowledgeable about CPM guidelines but were unfamiliar regarding pharmacological management and had negative attitudes toward opioids addiction and pain assessment. Significantly, knowledge and attitudes were higher among nurses who had previous education programs ($P < .001$) and worked in a pain team ($P < .001$). Therefore, including CPM topics in nursing curricula and postgraduate educational programs is needed. Additionally, initiating pain management teams and using CPM guidelines will contribute to effective treatment of cancer pain.

Institutions:
(Alnajar MK) School of Nursing, The University of Jordan, Amman, 11942, Jordan.; (Darawad MW) School of Nursing, The University of Jordan, Amman, 11942, Jordan. m.darawad@ju.edu.jo.; (Alshahwan SS) School of Nursing, The University of Jordan,

Amman, 11942, Jordan.; (Samarkandi OA) Prince Sultan bin Abdulaziz College for Emergency Medical Services, King Saud University, Riyadh, Saudi Arabia.
(Alnajar MK) School of Nursing, The University of Jordan, Amman, 11942, Jordan.; (Darawad MW) School of Nursing, The University of Jordan, Amman, 11942, Jordan. m.darawad@ju.edu.jo.; (Alshahwan SS) School of Nursing, The University of Jordan, Amman, 11942, Jordan.; (Samarkandi OA) Prince Sultan bin Abdulaziz College for Emergency Medical Services, King Saud University, Riyadh, Saudi Arabia.
(Alnajar MK) School of Nursing, The University of Jordan, Amman, 11942, Jordan.; (Darawad MW) School of Nursing, The University of Jordan, Amman, 11942, Jordan. m.darawad@ju.edu.jo.; (Alshahwan SS) School of Nursing, The University of Jordan, Amman, 11942, Jordan.; (Samarkandi OA) Prince Sultan bin Abdulaziz College for Emergency Medical Services, King Saud University, Riyadh, Saudi Arabia.
(Alnajar MK) School of Nursing, The University of Jordan, Amman, 11942, Jordan.; (Darawad MW) School of Nursing, The University of Jordan, Amman, 11942, Jordan. m.darawad@ju.edu.jo.; (Alshahwan SS) School of Nursing, The University of Jordan, Amman, 11942, Jordan.; (Samarkandi OA) Prince Sultan bin Abdulaziz College for Emergency Medical Services, King Saud University, Riyadh, Saudi Arabia.
Database: PubMed

77. Developing a Short Form of the German Barriers Questionnaire II: A Validation Study in Four Steps.

Author(s): Koller A; Jahn P

Source: Journal of pain and symptom management; 2018; vol. 55 (no. 2); p. 458-467

Publication Date: 2018

Publication Type(s): Journal Article; Research Support, Non-U.S. Gov't; Validation Study

DOI: http://dx.doi.org/10.1016/j.jpainsymman.2017.09.019

ISSN: 1873-6513

Place of Publication: United States

PubMedID: 28943361

Accession Number: 28943361

Available at Journal of pain and symptom management - from ScienceDirect

Available at Journal of pain and symptom management - from Unpaywall

Keywords: Subject Terms: *Patient education; *barriers toward pain management; *factor analysis; *neoplasms; *pain management; *surveys and questionnaires

Abstract:CONTEXT: Patient-related barriers to cancer pain management are most commonly assessed with the Barriers Questionnaire II (BQII; 27 items).OBJECTIVES: The aim of this study was to develop a valid short form of the BQII-German version (BQII-G) to increase usability in clinical routines and reduce patient burden.METHODS: The validation study comprised a stepwise approach. In the first step, the linguistic validated version of the BQII-G was psychometrically tested for internal consistency and factor structure (N = 207). The second step included an independent peer review in terms of expert ratings (four nurses and two patients) of each of the BQII-G items regarding (rather) include or (rather) not

include, according to the content validity index. The third step comprised a consensus process to integrate the expert ratings into a short form of the BQII-G (BQII-G12). The fourth step included a preliminary psychometric exploration of the short version of BQII-G12.RESULTS: Cronbach's α was 0.92 for the BQII-G. Steps 1-3 resulted in the BQII-G12 (12 items). The correlation showed that the BQII-G12 explains 84.3% (r = 0.92) of the variance of the BQII-G. Cronbach's alpha of the BQII-G12 was 0.833.CONCLUSION: The BQII-G12 showed excellent psychometric properties in the preliminary testing, providing a new option for practice and research. Patient-related barriers to cancer pain management are crucial for adequate pain treatment. The new valid and reliable short BQII-G12 supports clinical practice and research by substantially reducing patient burden and resources needed to measure these barriers.

Institutions:
(Koller A) Institute of Nursing Science, Faculty of Social Sciences, University of Vienna, Vienna, Austria. Electronic address: antje.koller@univie.ac.at.; (Jahn P) Institute for Health and Nursing Science, Medical Faculty, Martin-Luther-University Halle-Wittenberg, Halle (Saale), Germany; Nursing Research Unit, University Hospital Halle (Saale), Halle (Saale), Germany.
(Koller A) Institute of Nursing Science, Faculty of Social Sciences, University of Vienna, Vienna, Austria. Electronic address: antje.koller@univie.ac.at.; (Jahn P) Institute for Health and Nursing Science, Medical Faculty, Martin-Luther-University Halle-Wittenberg, Halle (Saale), Germany; Nursing Research Unit, University Hospital Halle (Saale), Halle (Saale), Germany.
Database: PubMed

78. Cancer pain management in China: current status and practice implications based on the ACHEON survey.

Author(s): Xia Z

Source: Journal of pain research; 2017; vol. 10 ; p. 1943-1952

Publication Date: 2017

Publication Type(s): Journal Article

DOI: http://dx.doi.org/10.2147/JPR.S128533

ISSN: 1178-7090

Place of Publication: New Zealand

PubMedID: 28860849

Accession Number: 28860849

Available at Journal of pain research - from Europe PubMed Central - Open Access

Available at Journal of pain research - from Unpaywall

Keywords: Subject Terms: cancer pain; opioid drugs; pain management; questionnaires

Abstract:PURPOSE: Cancer pain can seriously impact the quality of life (QoL) of patients, and optimal management practices are therefore of paramount importance. The ACHEON survey queried physicians and patients from 10 Asian countries/regions to assess current clinical practices in cancer pain management in Asia. This study presents the data obtained for cancer pain management in mainland

China, with an emphasis on practices related to opioid drugs.MATERIALS AND METHODS: In several tertiary hospitals across China, 250 patients experiencing cancer pain and 100 physicians were surveyed on questions designed to assess current cancer pain management practices and cancer pain impact on QoL.RESULTS: The patient survey showed that 88% of patients reported moderate-to-severe cancer pain, with a median duration of 6 months. The physician survey showed that medical school/residency training with regard to cancer pain management was inadequate in ~80% of physicians. A total of 80% of physicians and 67.2% of patients reported that pain scale was used during pain assessment; 84% of physicians expressed that physician-perceived pain severity was not completely consistent with actual pain the patient experienced. Of the 147 patients who recalled the medication received, 83.7% were administered opioid prescriptions. Of the 240 patients who received treatment, 43.8% perceived the inadequacy of controlling pain. The primary barriers from physicians perceived to optimal pain management included patients' fear of side effects (58%), patients' fear of addiction (53%), patients' reluctance to report pain (43%), physicians' reluctance to prescribe (29%), physicians' inadequacy of pain assessment (27%) and excessive regulation of opioid analgesics (47%).CONCLUSION: Knowledge of cancer pain management should be strengthened among physicians. Quantitative pain assessment and principle-based pain management should be combined to achieve pain relief. Misconceptions about opioids in patients and physicians and poor report about pain should be overcome through training/education to improve QoL of patients impacted by pain.

Institutions:
(Xia Z) Sun Yat-Sen University Cancer Center, Guangzhou, Guangdong, China.
Database: PubMed

79. Multimodal intrathecal analgesia in refractory cancer pain.

Author(s): Mastenbroek TC; Kramp-Hendriks BJ; Kallewaard JW; Vonk JM

Source: Scandinavian journal of pain; 2017; vol. 14 ; p. 39-43

Publication Date: 2017

Publication Type(s): Journal Article

DOI: http://dx.doi.org/10.1016/j.sjpain.2016.10.002

ISSN: 1877-8879

Place of Publication: Germany

PubMedID: 28850428

Accession Number: 28850428

Keywords: Subject Terms: *Clonidine; *Intractable cancer pain; *Intrathecal therapy; *Multimodal analgesia; *Neoplasms

Abstract:BACKGROUND AND AIMS: Cancer pain treatment has improved over the last decades. The majority of this population can be treated effectively with analgesics following the Guidelines of the original World Health Organisation (WHO). Unfortunately 10-15% of these patients still suffer from severe and refractory cancer pain, especially in the terminal phases of disease and require additional pain management modalities. Therefore, end-stage clinical interventions are particularly needed to minimize the perception of pain. With intrathecal therapy (ITT), drugs are

delivered close to their site of action in the central nervous system avoiding first-pass metabolism and blood-brain barrier. It may improve analgesia with a smaller dose and possibly achieve a reduction in systemic or cerebral side effects compared to oral supplied medication alone. Multimodal analgesia enables further dose reduction with improved analgesia and fewer side effects.METHODS: In this retrospective research we investigated the effectiveness and side-effect profile of intrathecal morphine, bupivacaine and clonidine. Patients were followed until death occurred. Pain scores and side effects were recorded before initiating ITT (T0), just after initiating ITT (T1), at hospital discharge (T2), in the ambulant setting (T3) and the last obtained scores before death occurred (T4).RESULTS: Nine patients were included who suffered from severe and refractory cancer pain, not reacting to conventional pain management or had intolerable side effects. Primary tumour location was pancreatic (4), urothelial (3) and prostate (2). Primary pain was considered neuropathic or mixed neuropathic-nociceptive. The treatment team consisted of an anaesthetist, specialized nurse in coordination with primary physician, treating oncologist and specialized home care. All patients were free of pain after initiation of the intrathecal therapy. The average follow-up period was 11 weeks in which there was a slight increase in NRS-score. In the last days before death occurred, half the patients were still free of pain. There were no problems during insertion of the catheter, device malfunction or infection. No severe adverse events defined as hypotension requiring inotropes, respiratory depression or neurological deficits were observed. Three patients experienced mild hypotension which gradually decreased after clonidine dose adjustment. Lower extremity weakness occurred in three patients as well. After bupivacaine dose adjustment the weakness disappeared in two patients and in one patient the lower extremity weakness persisted as a result of conus compression by tumour.CONCLUSION AND IMPLICATIONS: Multimodal IT treatment with morphine, bupivacaine and clonidine is effective and safe for treating refractory cancer pain in the terminal phase of disease. The study offers an important contribution to literature where there is still lack of convincing evidence about the benefits and harms of this type of pain management in patients with otherwise refractory cancer pain.

Institutions:

(Mastenbroek TC) Department of Anaesthesiology, Pain and Palliative Medicine, Radboud University Nijmegen Medical Centre, Geert Grooteplein Zuid 10, 6525 GA, Nijmegen, The Netherlands.; (Kramp-Hendriks BJ) Department of Anaesthesiology and Pain Management, Rijnstate Hospital, Wagnerlaan 55, 6815 AD, Arnhem, The Netherlands.; (Kallewaard JW) Department of Anaesthesiology and Pain Management, Rijnstate Hospital, Wagnerlaan 55, 6815 AD, Arnhem, The Netherlands.; (Vonk JM) Department of Anaesthesiology and Pain Management, Rijnstate Hospital, Wagnerlaan 55, 6815 AD, Arnhem, The Netherlands.
(Mastenbroek TC) Department of Anaesthesiology, Pain and Palliative Medicine, Radboud University Nijmegen Medical Centre, Geert Grooteplein Zuid 10, 6525 GA, Nijmegen, The Netherlands.; (Kramp-Hendriks BJ) Department of Anaesthesiology and Pain Management, Rijnstate Hospital, Wagnerlaan 55, 6815 AD, Arnhem, The Netherlands.; (Kallewaard JW) Department of Anaesthesiology and Pain Management, Rijnstate Hospital, Wagnerlaan 55, 6815 AD, Arnhem, The Netherlands.; (Vonk JM) Department of Anaesthesiology and Pain Management, Rijnstate Hospital, Wagnerlaan 55, 6815 AD, Arnhem, The Netherlands.
(Mastenbroek TC) Department of Anaesthesiology, Pain and Palliative Medicine, Radboud University Nijmegen Medical Centre, Geert Grooteplein Zuid 10, 6525 GA,

Nijmegen, The Netherlands.; (Kramp-Hendriks BJ) Department of Anaesthesiology and Pain Management, Rijnstate Hospital, Wagnerlaan 55, 6815 AD, Arnhem, The Netherlands.; (Kallewaard JW) Department of Anaesthesiology and Pain Management, Rijnstate Hospital, Wagnerlaan 55, 6815 AD, Arnhem, The Netherlands.; (Vonk JM) Department of Anaesthesiology and Pain Management, Rijnstate Hospital, Wagnerlaan 55, 6815 AD, Arnhem, The Netherlands.
(Mastenbroek TC) Department of Anaesthesiology, Pain and Palliative Medicine, Radboud University Nijmegen Medical Centre, Geert Grooteplein Zuid 10, 6525 GA, Nijmegen, The Netherlands.; (Kramp-Hendriks BJ) Department of Anaesthesiology and Pain Management, Rijnstate Hospital, Wagnerlaan 55, 6815 AD, Arnhem, The Netherlands.; (Kallewaard JW) Department of Anaesthesiology and Pain Management, Rijnstate Hospital, Wagnerlaan 55, 6815 AD, Arnhem, The Netherlands.; (Vonk JM) Department of Anaesthesiology and Pain Management, Rijnstate Hospital, Wagnerlaan 55, 6815 AD, Arnhem, The Netherlands.
Database: PubMed

80. Pain and pain management in hospitalized patients before and after an intervention.

Author(s): Andersson V; Bergman S; Henoch I; Ene KW; Otterström-Rydberg E; Simonsson H; Ahlberg K

Source: Scandinavian journal of pain; 2017; vol. 15 ; p. 22-29

Publication Date: 2017

Publication Type(s): Journal Article; Research Support, Non-U.S. Gov't

DOI: http://dx.doi.org/10.1016/j.sjpain.2016.11.006

ISSN: 1877-8879

Place of Publication: Germany

PubMedID: 28850341

Accession Number: 28850341

Keywords: Subject Terms: *Acute pain; *Analgesia; *Cancer pain; *Chronic pain; *Pain management; *Pain organisation

Abstract:BACKGROUND AND AIM: Studies have shown that pain is common among hospitalized patients and that there is a lack of compliance with pain management guidelines. Improving pain management does not only involve developing new drugs or technology; even more important is an effective organisation that utilises existing expertise. The aim of this study was to investigate whether pain in hospitalized patients can be reduced by implementing evidence-based pain management guidelines, providing education for staff and an organisation that includes pain responsibility nurses.METHODS: A cross-sectional study was carried out between 2009 and 2010 at two hospitals in southwest Sweden, comprising a baseline survey followed by an intervention. The study involved 306 patients, who answered questions about pain intensity at rest and while moving, disturbed sleep due to pain and whether they had used a pain rating scale while in hospital. Medical records were scrutinised for analgesic prescriptions. An intervention then took place, involving implementation of evidence-based guidelines, staff education and the introduction of pain responsibility nurses. A follow-up survey was carried out in 2012, in which 293 patients answered the same questions and

their medical records were also reviewed. The baseline results were then compared with those of the follow-up survey.RESULTS: When compared with the baseline survey, the follow-up survey revealed significant differences in the use of validated pain rating instruments as well as the prescription of more appropriate analgesics. Prescription of paracetamol increased significantly in the follow-up survey; 56% of the patients were prescribed paracetamol on a regular basis, compared with 42% at baseline. There was also a significant increase in the use of strong opioids, from 38% at baseline to 55% at follow-up. Prescriptions of weak opioids decreased from 16% at baseline to 4% at follow-up. No significant differences were observed in patient pain levels in the follow-up survey. At baseline, 29% of the patients reported moderate to severe pain at rest (NRS 4-10) and at follow-up that figure was 24% (NRS 4-10). In both surveys, 41% reported moderate to severe pain (NRS 5-10) during movement. Thirty-nine percent reported disturbed sleep at night at both baseline and follow-up.CONCLUSIONS: This study demonstrates that evidence-based guidelines made accessible to all staff as a pocket size booklet and on the intranet, in combination with staff education, pain responsibility nurses who informed other staff on their own wards, improved the prescription of analgesics in the hospitals studied. In order to achieve a noticeable effect for patients, i.e., reduced pain levels, an intervention containing more components than those employed in the present study is required.IMPLICATIONS: Nurses and physicians need greater knowledge about the importance of pain rating. A vital part of pain management at hospitals is continuous evaluation of treatment outcomes to prevent severe pain and disturbed sleep. The complexity of pain and pain management requires commitment, time and knowledge on the part of healthcare staff. Multi-professional pain teams that support ward staff in pain management are necessary in order to reduce suffering and unnecessary pain in hospitalized patients.

Institutions:

(Andersson V) The Sahlgrenska Academy, University of Gothenburg, Institute of Health and Care Sciences, Box 457, 405 30 Gothenborg, Gothenburg, Sweden.; (Bergman S) Primary Health Care Unit, Department of Public Health and Community Medicine, Institute of Medicine, The Sahlgrenska Academy, University of Gothenburg, Box 457, 405 30, Gothenborg, Sweden.; (Henoch I) The Sahlgrenska Academy, University of Gothenburg, Institute of Health and Care Sciences, Box 457, 405 30 Gothenborg, Gothenburg, Sweden.; (Ene KW) Department of Research, Development and Education, Hallands Hospital, Varberg, Träslövsvägen 68, 432 37 Varberg, Sweden.; (Otterström-Rydberg E) Department of Anesthesia and Intensive Care, Hallands Hospital, Varberg, Träslövsvägen 68, 432 37 Varberg, Sweden.; (Simonsson H) Department of Surgery, Hallands Hospital, Halmstad, Lasarettsvägen, 302 33 Halmstad, Sweden.; (Ahlberg K) The Sahlgrenska Academy, University of Gothenburg, Institute of Health and Care Sciences, Box 457, 405 30 Gothenborg, Gothenburg, Sweden.

(Andersson V) The Sahlgrenska Academy, University of Gothenburg, Institute of Health and Care Sciences, Box 457, 405 30 Gothenborg, Gothenburg, Sweden.; (Bergman S) Primary Health Care Unit, Department of Public Health and Community Medicine, Institute of Medicine, The Sahlgrenska Academy, University of Gothenburg, Box 457, 405 30, Gothenborg, Sweden.; (Henoch I) The Sahlgrenska Academy, University of Gothenburg, Institute of Health and Care Sciences, Box 457, 405 30 Gothenborg, Gothenburg, Sweden.; (Ene KW) Department of Research, Development and Education, Hallands Hospital, Varberg, Träslövsvägen 68, 432 37 Varberg, Sweden.; (Otterström-Rydberg E) Department of Anesthesia and Intensive

Care, Hallands Hospital, Varberg, Träslövsvägen 68, 432 37 Varberg, Sweden.; (Simonsson H) Department of Surgery, Hallands Hospital, Halmstad, Lasarettsvägen, 302 33 Halmstad, Sweden.; (Ahlberg K) The Sahlgrenska Academy, University of Gothenburg, Institute of Health and Care Sciences, Box 457, 405 30 Gothenborg, Gothenburg, Sweden.
(Andersson V) The Sahlgrenska Academy, University of Gothenburg, Institute of Health and Care Sciences, Box 457, 405 30 Gothenborg, Gothenburg, Sweden.; (Bergman S) Primary Health Care Unit, Department of Public Health and Community Medicine, Institute of Medicine, The Sahlgrenska Academy, University of Gothenburg, Box 457, 405 30, Gothenborg, Sweden.; (Henoch I) The Sahlgrenska Academy, University of Gothenburg, Institute of Health and Care Sciences, Box 457, 405 30 Gothenborg, Gothenburg, Sweden.; (Ene KW) Department of Research, Development and Education, Hallands Hospital, Varberg, Träslövsvägen 68, 432 37 Varberg, Sweden.; (Otterström-Rydberg E) Department of Anesthesia and Intensive Care, Hallands Hospital, Varberg, Träslövsvägen 68, 432 37 Varberg, Sweden.; (Simonsson H) Department of Surgery, Hallands Hospital, Halmstad, Lasarettsvägen, 302 33 Halmstad, Sweden.; (Ahlberg K) The Sahlgrenska Academy, University of Gothenburg, Institute of Health and Care Sciences, Box 457, 405 30 Gothenborg, Gothenburg, Sweden.
(Andersson V) The Sahlgrenska Academy, University of Gothenburg, Institute of Health and Care Sciences, Box 457, 405 30 Gothenborg, Gothenburg, Sweden.; (Bergman S) Primary Health Care Unit, Department of Public Health and Community Medicine, Institute of Medicine, The Sahlgrenska Academy, University of Gothenburg, Box 457, 405 30, Gothenborg, Sweden.; (Henoch I) The Sahlgrenska Academy, University of Gothenburg, Institute of Health and Care Sciences, Box 457, 405 30 Gothenborg, Gothenburg, Sweden.; (Ene KW) Department of Research, Development and Education, Hallands Hospital, Varberg, Träslövsvägen 68, 432 37 Varberg, Sweden.; (Otterström-Rydberg E) Department of Anesthesia and Intensive Care, Hallands Hospital, Varberg, Träslövsvägen 68, 432 37 Varberg, Sweden.; (Simonsson H) Department of Surgery, Hallands Hospital, Halmstad, Lasarettsvägen, 302 33 Halmstad, Sweden.; (Ahlberg K) The Sahlgrenska Academy, University of Gothenburg, Institute of Health and Care Sciences, Box 457, 405 30 Gothenborg, Gothenburg, Sweden.
(Andersson V) The Sahlgrenska Academy, University of Gothenburg, Institute of Health and Care Sciences, Box 457, 405 30 Gothenborg, Gothenburg, Sweden.; (Bergman S) Primary Health Care Unit, Department of Public Health and Community Medicine, Institute of Medicine, The Sahlgrenska Academy, University of Gothenburg, Box 457, 405 30, Gothenborg, Sweden.; (Henoch I) The Sahlgrenska Academy, University of Gothenburg, Institute of Health and Care Sciences, Box 457, 405 30 Gothenborg, Gothenburg, Sweden.; (Ene KW) Department of Research, Development and Education, Hallands Hospital, Varberg, Träslövsvägen 68, 432 37 Varberg, Sweden.; (Otterström-Rydberg E) Department of Anesthesia and Intensive Care, Hallands Hospital, Varberg, Träslövsvägen 68, 432 37 Varberg, Sweden.; (Simonsson H) Department of Surgery, Hallands Hospital, Halmstad, Lasarettsvägen, 302 33 Halmstad, Sweden.; (Ahlberg K) The Sahlgrenska Academy, University of Gothenburg, Institute of Health and Care Sciences, Box 457, 405 30 Gothenborg, Gothenburg, Sweden.
(Andersson V) The Sahlgrenska Academy, University of Gothenburg, Institute of Health and Care Sciences, Box 457, 405 30 Gothenborg, Gothenburg, Sweden.; (Bergman S) Primary Health Care Unit, Department of Public Health and Community

Medicine, Institute of Medicine, The Sahlgrenska Academy, University of Gothenburg, Box 457, 405 30, Gothenborg, Sweden.; (Henoch I) The Sahlgrenska Academy, University of Gothenburg, Institute of Health and Care Sciences, Box 457, 405 30 Gothenborg, Gothenburg, Sweden.; (Ene KW) Department of Research, Development and Education, Hallands Hospital, Varberg, Träslövsvägen 68, 432 37 Varberg, Sweden.; (Otterström-Rydberg E) Department of Anesthesia and Intensive Care, Hallands Hospital, Varberg, Träslövsvägen 68, 432 37 Varberg, Sweden.; (Simonsson H) Department of Surgery, Hallands Hospital, Halmstad, Lasarettsvägen, 302 33 Halmstad, Sweden.; (Ahlberg K) The Sahlgrenska Academy, University of Gothenburg, Institute of Health and Care Sciences, Box 457, 405 30 Gothenborg, Gothenburg, Sweden.
(Andersson V) The Sahlgrenska Academy, University of Gothenburg, Institute of Health and Care Sciences, Box 457, 405 30 Gothenborg, Gothenburg, Sweden.; (Bergman S) Primary Health Care Unit, Department of Public Health and Community
Medicine, Institute of Medicine, The Sahlgrenska Academy, University of Gothenburg, Box 457, 405 30, Gothenborg, Sweden.; (Henoch I) The Sahlgrenska Academy, University of Gothenburg, Institute of Health and Care Sciences, Box 457, 405 30 Gothenborg, Gothenburg, Sweden.; (Ene KW) Department of Research, Development and Education, Hallands Hospital, Varberg, Träslövsvägen 68, 432 37 Varberg, Sweden.; (Otterström-Rydberg E) Department of Anesthesia and Intensive Care, Hallands Hospital, Varberg, Träslövsvägen 68, 432 37 Varberg, Sweden.; (Simonsson H) Department of Surgery, Hallands Hospital, Halmstad, Lasarettsvägen, 302 33 Halmstad, Sweden.; (Ahlberg K) The Sahlgrenska Academy, University of Gothenburg, Institute of Health and Care Sciences, Box 457, 405 30 Gothenborg, Gothenburg, Sweden.
Database: PubMed

81. The social and behavioral influences (SBI) study: study design and rationale for studying the effects of race and activation on cancer pain management.

Author(s): Elias CM; Shields CG; Griggs JJ; Fiscella K; Christ SL; Colbert J; Henry SG; Hoh BG; Hunte HER; Marshall M; Mohile SG; Plumb S; Tejani MA; Venuti A; Epstein RM

Source: BMC cancer; Aug 2017; vol. 17 (no. 1); p. 575

Publication Date: Aug 2017

Publication Type(s): Clinical Trial; Journal Article

DOI: http://dx.doi.org/10.1186/s12885-017-3564-2

ISSN: 1471-2407

Place of Publication: England

PubMedID: 28841847

Accession Number: 28841847

Available at BMC cancer - from BioMed Central

Available at BMC cancer - from Europe PubMed Central - Open Access

Available at BMC cancer - from DOAJ - Directory of Open Access Journals

Available at BMC cancer - from ProQuest (Health Research Premium) - NHS Version

Available at BMC cancer - from EBSCO (MEDLINE Complete)

Keywords: Subject Terms: Cancer; End of life care; Field experiment; Implicit bias; Pain management; Palliative care; Patient-centered communication; Racial disparities; Randomized clinical trial; Standardized patients

Abstract:BACKGROUND: Racial disparities exist in the care provided to advanced cancer patients. This article describes an investigation designed to advance the science of healthcare disparities by isolating the effects of patient race and patient activation on physician behavior using novel standardized patient (SP) methodology.METHODS/DESIGN: The Social and Behavioral Influences (SBI) Study is a National Cancer Institute sponsored trial conducted in Western New York State, Northern/Central Indiana, and lower Michigan. The trial uses an incomplete randomized block design, randomizing physicians to see patients who are either black or white and who are "typical" or "activated" (e.g., ask questions, express opinions, ask for clarification, etc.). The study will enroll 91 physicians.DISCUSSION: The SBI study addresses important gaps in our knowledge about racial disparities and methods to reduce them in patients with advanced cancer by using standardized patient methodology. This study is innovative in aims, design, and methodology and will point the way to interventions that can reduce racial disparities and discrimination and draw links between implicit attitudes and physician behaviors.TRIAL REGISTRATION: https://clinicaltrials.gov/ , #NCT01501006, November 30, 2011.

Institutions:
(Elias CM) Department of Statistics, West Lafayette, Purdue University, Human Development & Family Studies, Indiana, 47906, USA.; (Shields CG) Purdue University Center for Cancer Research, Regenstrief Center for Healthcare Engineering, Human Development & Family Studies, Fowler Memorial House, 1200 W State Street, West Lafayette, IN, 47906, USA. cgshields@purdue.edu.; (Griggs JJ) Department of Internal Medicine, Hematology & Oncology Division and Department of Health Management & Policy Ann Arbor, University of Michigan School of Medicine, Ann Arbor, MI, 48109-0419, USA.; (Fiscella K) Department of Public Health Sciences, University of Rochester School of Medicine, Family Medicine, Rochester, NY, 14642, USA.; (Christ SL) Department of Statistics, West Lafayette, Purdue University, Human Development & Family Studies, Indiana, 47906, USA.; (Colbert J) Biostatistics Department, School of Public Health, University of Michigan, Ann Arbor, MI, 48109, 14642, USA.; (Henry SG) Department of Internal Medicine, University of California Davis School of Medicine, Sacramento, CA, University of Rochester Medical Center, Rochester, NY, USA.; (Hoh BG) Department of Internal Medicine, University of California Davis School of Medicine, Sacramento, CA, University of Rochester Medical Center, Rochester, NY, USA.; (Hunte HER) West Virginia University, Robert C. Byrd Health Sciences Center, Morgantown, West VA, 26506, USA.; (Marshall M) Department of Statistics, West Lafayette, Purdue University, Human Development & Family Studies, Indiana, 47906, USA.; (Mohile SG) Center for Communication and Disparities Research, University of Rochester School of Medicine, Family Medicine, James P Wilmot Cancer Center, Rochester, NY, 14642, USA.; (Plumb S) University of Rochester School of Medicine, Family Medicine, Rochester, NY, 14642, USA.; (Tejani MA) James P Wilmot Cancer Center, University of Rochester Medical Center, Rochester, NY, 14642, USA.; (Venuti A) University of Rochester School of Medicine, Family

Medicine, Rochester, NY, 14642, USA.; (Epstein RM) Center for Communication and Disparities Research, University of Rochester School of Medicine, Family Medicine, James P Wilmot Cancer Center, Rochester, NY, 14642, USA.
(Elias CM) Department of Statistics, West Lafayette, Purdue University, Human Development & Family Studies, Indiana, 47906, USA.; (Shields CG) Purdue University Center for Cancer Research, Regenstrief Center for Healthcare Engineering, Human Development & Family Studies, Fowler Memorial House, 1200 W State Street, West Lafayette, IN, 47906, USA. cgshields@purdue.edu.; (Griggs JJ) Department of Internal Medicine, Hematology & Oncology Division and Department of Health Management & Policy Ann Arbor, University of Michigan School of Medicine, Ann Arbor, MI, 48109-0419, USA.; (Fiscella K) Department of Public Health Sciences, University of Rochester School of Medicine, Family Medicine, Rochester, NY, 14642, USA.; (Christ SL) Department of Statistics, West Lafayette, Purdue University, Human Development & Family Studies, Indiana, 47906, USA.; (Colbert J) Biostatistics Department, School of Public Health, University of Michigan, Ann Arbor, MI, 48109, 14642, USA.; (Henry SG) Department of Internal Medicine, University of California Davis School of Medicine, Sacramento, CA, University of Rochester Medical Center, Rochester, NY, USA.; (Hoh BG) Department of Internal Medicine, University of California Davis School of Medicine, Sacramento, CA, University of Rochester Medical Center, Rochester, NY, USA.; (Hunte HER) West Virginia University, Robert C. Byrd Health Sciences Center, Morgantown, West VA, 26506, USA.; (Marshall M) Department of Statistics, West Lafayette, Purdue University, Human Development & Family Studies, Indiana, 47906, USA.; (Mohile SG) Center for Communication and Disparities Research, University of Rochester School of Medicine, Family Medicine, James P Wilmot Cancer Center, Rochester, NY, 14642, USA.; (Plumb S) University of Rochester School of Medicine, Family Medicine, Rochester, NY, 14642, USA.; (Tejani MA) James P Wilmot Cancer Center, University of Rochester Medical Center, Rochester, NY, 14642, USA.; (Venuti A) University of Rochester School of Medicine, Family Medicine, Rochester, NY, 14642, USA.; (Epstein RM) Center for Communication and Disparities Research, University of Rochester School of Medicine, Family Medicine, James P Wilmot Cancer Center, Rochester, NY, 14642, USA.
(Elias CM) Department of Statistics, West Lafayette, Purdue University, Human Development & Family Studies, Indiana, 47906, USA.; (Shields CG) Purdue University Center for Cancer Research, Regenstrief Center for Healthcare Engineering, Human Development & Family Studies, Fowler Memorial House, 1200 W State Street, West Lafayette, IN, 47906, USA. cgshields@purdue.edu.; (Griggs JJ) Department of Internal Medicine, Hematology & Oncology Division and Department of Health Management & Policy Ann Arbor, University of Michigan School of Medicine, Ann Arbor, MI, 48109-0419, USA.; (Fiscella K) Department of Public Health Sciences, University of Rochester School of Medicine, Family Medicine, Rochester, NY, 14642, USA.; (Christ SL) Department of Statistics, West Lafayette, Purdue University, Human Development & Family Studies, Indiana, 47906, USA.; (Colbert J) Biostatistics Department, School of Public Health, University of Michigan, Ann Arbor, MI, 48109, 14642, USA.; (Henry SG) Department of Internal Medicine, University of California Davis School of Medicine, Sacramento, CA, University of Rochester Medical Center, Rochester, NY, USA.; (Hoh BG) Department of Internal Medicine, University of California Davis School of Medicine, Sacramento, CA, University of Rochester Medical Center, Rochester, NY, USA.; (Hunte HER) West Virginia University, Robert C. Byrd Health Sciences Center,

Morgantown, West VA, 26506, USA.; (Marshall M) Department of Statistics, West Lafayette, Purdue University, Human Development & Family Studies, Indiana, 47906, USA.; (Mohile SG) Center for Communication and Disparities Research, University of Rochester School of Medicine, Family Medicine, James P Wilmot Cancer Center, Rochester, NY, 14642, USA.; (Plumb S) University of Rochester School of Medicine, Family Medicine, Rochester, NY, 14642, USA.; (Tejani MA) James P Wilmot Cancer Center, University of Rochester Medical Center, Rochester, NY, 14642, USA.; (Venuti A) University of Rochester School of Medicine, Family Medicine, Rochester, NY, 14642, USA.; (Epstein RM) Center for Communication and Disparities Research, University of Rochester School of Medicine, Family Medicine, James P Wilmot Cancer Center, Rochester, NY, 14642, USA.
(Elias CM) Department of Statistics, West Lafayette, Purdue University, Human Development & Family Studies, Indiana, 47906, USA.; (Shields CG) Purdue University Center for Cancer Research, Regenstrief Center for Healthcare Engineering, Human Development & Family Studies, Fowler Memorial House, 1200 W State Street, West Lafayette, IN, 47906, USA. cgshields@purdue.edu.; (Griggs JJ) Department of Internal Medicine, Hematology & Oncology Division and Department of Health Management & Policy Ann Arbor, University of Michigan School of Medicine, Ann Arbor, MI, 48109-0419, USA.; (Fiscella K) Department of Public Health Sciences, University of Rochester School of Medicine, Family Medicine, Rochester, NY, 14642, USA.; (Christ SL) Department of Statistics, West Lafayette, Purdue University, Human Development & Family Studies, Indiana, 47906, USA.; (Colbert J) Biostatistics Department, School of Public Health, University of Michigan, Ann Arbor, MI, 48109, 14642, USA.; (Henry SG) Department of Internal Medicine, University of California Davis School of Medicine, Sacramento, CA, University of Rochester Medical Center, Rochester, NY, USA.; (Hoh BG) Department of Internal Medicine, University of California Davis School of Medicine, Sacramento, CA, University of Rochester Medical Center, Rochester, NY, USA.; (Hunte HER) West Virginia University, Robert C. Byrd Health Sciences Center, Morgantown, West VA, 26506, USA.; (Marshall M) Department of Statistics, West Lafayette, Purdue University, Human Development & Family Studies, Indiana, 47906, USA.; (Mohile SG) Center for Communication and Disparities Research, University of Rochester School of Medicine, Family Medicine, James P Wilmot Cancer Center, Rochester, NY, 14642, USA.; (Plumb S) University of Rochester School of Medicine, Family Medicine, Rochester, NY, 14642, USA.; (Tejani MA) James P Wilmot Cancer Center, University of Rochester Medical Center, Rochester, NY, 14642, USA.; (Venuti A) University of Rochester School of Medicine, Family Medicine, Rochester, NY, 14642, USA.; (Epstein RM) Center for Communication and Disparities Research, University of Rochester School of Medicine, Family Medicine, James P Wilmot Cancer Center, Rochester, NY, 14642, USA.
(Elias CM) Department of Statistics, West Lafayette, Purdue University, Human Development & Family Studies, Indiana, 47906, USA.; (Shields CG) Purdue University Center for Cancer Research, Regenstrief Center for Healthcare Engineering, Human Development & Family Studies, Fowler Memorial House, 1200 W State Street, West Lafayette, IN, 47906, USA. cgshields@purdue.edu.; (Griggs JJ) Department of Internal Medicine, Hematology & Oncology Division and Department of Health Management & Policy Ann Arbor, University of Michigan School of Medicine, Ann Arbor, MI, 48109-0419, USA.; (Fiscella K) Department of Public Health Sciences, University of Rochester School of Medicine, Family Medicine, Rochester, NY, 14642, USA.; (Christ SL) Department of Statistics, West

Lafayette, Purdue University, Human Development & Family Studies, Indiana, 47906, USA.; (Colbert J) Biostatistics Department, School of Public Health, University of Michigan, Ann Arbor, MI, 48109, 14642, USA.; (Henry SG) Department of Internal Medicine, University of California Davis School of Medicine, Sacramento, CA, University of Rochester Medical Center, Rochester, NY, USA.; (Hoh BG) Department of Internal Medicine, University of California Davis School of Medicine, Sacramento, CA, University of Rochester Medical Center, Rochester, NY, USA.; (Hunte HER) West Virginia University, Robert C. Byrd Health Sciences Center, Morgantown, West VA, 26506, USA.; (Marshall M) Department of Statistics, West Lafayette, Purdue University, Human Development & Family Studies, Indiana, 47906, USA.; (Mohile SG) Center for Communication and Disparities Research, University of Rochester School of Medicine, Family Medicine, James P Wilmot Cancer Center, Rochester, NY, 14642, USA.; (Plumb S) University of Rochester School of Medicine, Family Medicine, Rochester, NY, 14642, USA.; (Tejani MA) James P Wilmot Cancer Center, University of Rochester Medical Center, Rochester, NY, 14642, USA.; (Venuti A) University of Rochester School of Medicine, Family Medicine, Rochester, NY, 14642, USA.; (Epstein RM) Center for Communication and Disparities Research, University of Rochester School of Medicine, Family Medicine, James P Wilmot Cancer Center, Rochester, NY, 14642, USA.
(Elias CM) Department of Statistics, West Lafayette, Purdue University, Human Development & Family Studies, Indiana, 47906, USA.; (Shields CG) Purdue University Center for Cancer Research, Regenstrief Center for Healthcare Engineering, Human Development & Family Studies, Fowler Memorial House, 1200 W State Street, West Lafayette, IN, 47906, USA. cgshields@purdue.edu.; (Griggs JJ) Department of Internal Medicine, Hematology & Oncology Division and Department of Health Management & Policy Ann Arbor, University of Michigan School of Medicine, Ann Arbor, MI, 48109-0419, USA.; (Fiscella K) Department of Public Health Sciences, University of Rochester School of Medicine, Family Medicine, Rochester, NY, 14642, USA.; (Christ SL) Department of Statistics, West Lafayette, Purdue University, Human Development & Family Studies, Indiana, 47906, USA.; (Colbert J) Biostatistics Department, School of Public Health, University of Michigan, Ann Arbor, MI, 48109, 14642, USA.; (Henry SG) Department of Internal Medicine, University of California Davis School of Medicine, Sacramento, CA, University of Rochester Medical Center, Rochester, NY, USA.; (Hoh BG) Department of Internal Medicine, University of California Davis School of Medicine, Sacramento, CA, University of Rochester Medical Center, Rochester, NY, USA.; (Hunte HER) West Virginia University, Robert C. Byrd Health Sciences Center, Morgantown, West VA, 26506, USA.; (Marshall M) Department of Statistics, West Lafayette, Purdue University, Human Development & Family Studies, Indiana, 47906, USA.; (Mohile SG) Center for Communication and Disparities Research, University of Rochester School of Medicine, Family Medicine, James P Wilmot Cancer Center, Rochester, NY, 14642, USA.; (Plumb S) University of Rochester School of Medicine, Family Medicine, Rochester, NY, 14642, USA.; (Tejani MA) James P Wilmot Cancer Center, University of Rochester Medical Center, Rochester, NY, 14642, USA.; (Venuti A) University of Rochester School of Medicine, Family Medicine, Rochester, NY, 14642, USA.; (Epstein RM) Center for Communication and Disparities Research, University of Rochester School of Medicine, Family Medicine, James P Wilmot Cancer Center, Rochester, NY, 14642, USA.
(Elias CM) Department of Statistics, West Lafayette, Purdue University, Human Development & Family Studies, Indiana, 47906, USA.; (Shields CG) Purdue

University Center for Cancer Research, Regenstrief Center for Healthcare Engineering, Human Development & Family Studies, Fowler Memorial House, 1200 W State Street, West Lafayette, IN, 47906, USA. cgshields@purdue.edu.; (Griggs JJ) Department of Internal Medicine, Hematology & Oncology Division and Department of Health Management & Policy Ann Arbor, University of Michigan School of Medicine, Ann Arbor, MI, 48109-0419, USA.; (Fiscella K) Department of Public Health Sciences, University of Rochester School of Medicine, Family Medicine, Rochester, NY, 14642, USA.; (Christ SL) Department of Statistics, West Lafayette, Purdue University, Human Development & Family Studies, Indiana, 47906, USA.; (Colbert J) Biostatistics Department, School of Public Health, University of Michigan, Ann Arbor, MI, 48109, 14642, USA.; (Henry SG) Department of Internal Medicine, University of California Davis School of Medicine, Sacramento, CA, University of Rochester Medical Center, Rochester, NY, USA.; (Hoh BG) Department of Internal Medicine, University of California Davis School of Medicine, Sacramento, CA, University of Rochester Medical Center, Rochester, NY, USA.; (Hunte HER) West Virginia University, Robert C. Byrd Health Sciences Center, Morgantown, West VA, 26506, USA.; (Marshall M) Department of Statistics, West Lafayette, Purdue University, Human Development & Family Studies, Indiana, 47906, USA.; (Mohile SG) Center for Communication and Disparities Research, University of Rochester School of Medicine, Family Medicine, James P Wilmot Cancer Center, Rochester, NY, 14642, USA.; (Plumb S) University of Rochester School of Medicine, Family Medicine, Rochester, NY, 14642, USA.; (Tejani MA) James P Wilmot Cancer Center, University of Rochester Medical Center, Rochester, NY, 14642, USA.; (Venuti A) University of Rochester School of Medicine, Family Medicine, Rochester, NY, 14642, USA.; (Epstein RM) Center for Communication and Disparities Research, University of Rochester School of Medicine, Family Medicine, James P Wilmot Cancer Center, Rochester, NY, 14642, USA.

(Elias CM) Department of Statistics, West Lafayette, Purdue University, Human Development & Family Studies, Indiana, 47906, USA.; (Shields CG) Purdue University Center for Cancer Research, Regenstrief Center for Healthcare Engineering, Human Development & Family Studies, Fowler Memorial House, 1200 W State Street, West Lafayette, IN, 47906, USA. cgshields@purdue.edu.; (Griggs JJ) Department of Internal Medicine, Hematology & Oncology Division and Department of Health Management & Policy Ann Arbor, University of Michigan School of Medicine, Ann Arbor, MI, 48109-0419, USA.; (Fiscella K) Department of Public Health Sciences, University of Rochester School of Medicine, Family Medicine, Rochester, NY, 14642, USA.; (Christ SL) Department of Statistics, West Lafayette, Purdue University, Human Development & Family Studies, Indiana, 47906, USA.; (Colbert J) Biostatistics Department, School of Public Health, University of Michigan, Ann Arbor, MI, 48109, 14642, USA.; (Henry SG) Department of Internal Medicine, University of California Davis School of Medicine, Sacramento, CA, University of Rochester Medical Center, Rochester, NY, USA.; (Hoh BG) Department of Internal Medicine, University of California Davis School of Medicine, Sacramento, CA, University of Rochester Medical Center, Rochester, NY, USA.; (Hunte HER) West Virginia University, Robert C. Byrd Health Sciences Center, Morgantown, West VA, 26506, USA.; (Marshall M) Department of Statistics, West Lafayette, Purdue University, Human Development & Family Studies, Indiana, 47906, USA.; (Mohile SG) Center for Communication and Disparities Research, University of Rochester School of Medicine, Family Medicine, James P Wilmot Cancer Center, Rochester, NY, 14642, USA.; (Plumb S) University of Rochester

School of Medicine, Family Medicine, Rochester, NY, 14642, USA.; (Tejani MA) James P Wilmot Cancer Center, University of Rochester Medical Center, Rochester, NY, 14642, USA.; (Venuti A) University of Rochester School of Medicine, Family Medicine, Rochester, NY, 14642, USA.; (Epstein RM) Center for Communication and Disparities Research, University of Rochester School of Medicine, Family Medicine, James P Wilmot Cancer Center, Rochester, NY, 14642, USA.
(Elias CM) Department of Statistics, West Lafayette, Purdue University, Human Development & Family Studies, Indiana, 47906, USA.; (Shields CG) Purdue University Center for Cancer Research, Regenstrief Center for Healthcare Engineering, Human Development & Family Studies, Fowler Memorial House, 1200 W State Street, West Lafayette, IN, 47906, USA. cgshields@purdue.edu.; (Griggs JJ) Department of Internal Medicine, Hematology & Oncology Division and Department of Health Management & Policy Ann Arbor, University of Michigan School of Medicine, Ann Arbor, MI, 48109-0419, USA.; (Fiscella K) Department of Public Health Sciences, University of Rochester School of Medicine, Family Medicine, Rochester, NY, 14642, USA.; (Christ SL) Department of Statistics, West Lafayette, Purdue University, Human Development & Family Studies, Indiana, 47906, USA.; (Colbert J) Biostatistics Department, School of Public Health, University of Michigan, Ann Arbor, MI, 48109, 14642, USA.; (Henry SG) Department of Internal Medicine, University of California Davis School of Medicine, Sacramento, CA, University of Rochester Medical Center, Rochester, NY, USA.; (Hoh BG) Department of Internal Medicine, University of California Davis School of Medicine, Sacramento, CA, University of Rochester Medical Center, Rochester, NY, USA.; (Hunte HER) West Virginia University, Robert C. Byrd Health Sciences Center, Morgantown, West VA, 26506, USA.; (Marshall M) Department of Statistics, West Lafayette, Purdue University, Human Development & Family Studies, Indiana, 47906, USA.; (Mohile SG) Center for Communication and Disparities Research, University of Rochester School of Medicine, Family Medicine, James P Wilmot Cancer Center, Rochester, NY, 14642, USA.; (Plumb S) University of Rochester School of Medicine, Family Medicine, Rochester, NY, 14642, USA.; (Tejani MA) James P Wilmot Cancer Center, University of Rochester Medical Center, Rochester, NY, 14642, USA.; (Venuti A) University of Rochester School of Medicine, Family Medicine, Rochester, NY, 14642, USA.; (Epstein RM) Center for Communication and Disparities Research, University of Rochester School of Medicine, Family Medicine, James P Wilmot Cancer Center, Rochester, NY, 14642, USA.
(Elias CM) Department of Statistics, West Lafayette, Purdue University, Human Development & Family Studies, Indiana, 47906, USA.; (Shields CG) Purdue University Center for Cancer Research, Regenstrief Center for Healthcare Engineering, Human Development & Family Studies, Fowler Memorial House, 1200 W State Street, West Lafayette, IN, 47906, USA. cgshields@purdue.edu.; (Griggs JJ) Department of Internal Medicine, Hematology & Oncology Division and Department of Health Management & Policy Ann Arbor, University of Michigan School of Medicine, Ann Arbor, MI, 48109-0419, USA.; (Fiscella K) Department of Public Health Sciences, University of Rochester School of Medicine, Family Medicine, Rochester, NY, 14642, USA.; (Christ SL) Department of Statistics, West Lafayette, Purdue University, Human Development & Family Studies, Indiana, 47906, USA.; (Colbert J) Biostatistics Department, School of Public Health, University of Michigan, Ann Arbor, MI, 48109, 14642, USA.; (Henry SG) Department of Internal Medicine, University of California Davis School of Medicine, Sacramento, CA, University of Rochester Medical Center, Rochester, NY, USA.; (Hoh BG)

Department of Internal Medicine, University of California Davis School of Medicine, Sacramento, CA, University of Rochester Medical Center, Rochester, NY, USA.; (Hunte HER) West Virginia University, Robert C. Byrd Health Sciences Center, Morgantown, West VA, 26506, USA.; (Marshall M) Department of Statistics, West Lafayette, Purdue University, Human Development & Family Studies, Indiana, 47906, USA.; (Mohile SG) Center for Communication and Disparities Research, University of Rochester School of Medicine, Family Medicine, James P Wilmot Cancer Center, Rochester, NY, 14642, USA.; (Plumb S) University of Rochester School of Medicine, Family Medicine, Rochester, NY, 14642, USA.; (Tejani MA) James P Wilmot Cancer Center, University of Rochester Medical Center, Rochester, NY, 14642, USA.; (Venuti A) University of Rochester School of Medicine, Family Medicine, Rochester, NY, 14642, USA.; (Epstein RM) Center for Communication and Disparities Research, University of Rochester School of Medicine, Family Medicine, James P Wilmot Cancer Center, Rochester, NY, 14642, USA.
(Elias CM) Department of Statistics, West Lafayette, Purdue University, Human Development & Family Studies, Indiana, 47906, USA.; (Shields CG) Purdue University Center for Cancer Research, Regenstrief Center for Healthcare Engineering, Human Development & Family Studies, Fowler Memorial House, 1200 W State Street, West Lafayette, IN, 47906, USA. cgshields@purdue.edu.; (Griggs JJ) Department of Internal Medicine, Hematology & Oncology Division and Department of Health Management & Policy Ann Arbor, University of Michigan School of Medicine, Ann Arbor, MI, 48109-0419, USA.; (Fiscella K) Department of Public Health Sciences, University of Rochester School of Medicine, Family Medicine, Rochester, NY, 14642, USA.; (Christ SL) Department of Statistics, West Lafayette, Purdue University, Human Development & Family Studies, Indiana, 47906, USA.; (Colbert J) Biostatistics Department, School of Public Health, University of Michigan, Ann Arbor, MI, 48109, 14642, USA.; (Henry SG) Department of Internal Medicine, University of California Davis School of Medicine, Sacramento, CA, University of Rochester Medical Center, Rochester, NY, USA.; (Hoh BG) Department of Internal Medicine, University of California Davis School of Medicine, Sacramento, CA, University of Rochester Medical Center, Rochester, NY, USA.; (Hunte HER) West Virginia University, Robert C. Byrd Health Sciences Center, Morgantown, West VA, 26506, USA.; (Marshall M) Department of Statistics, West Lafayette, Purdue University, Human Development & Family Studies, Indiana, 47906, USA.; (Mohile SG) Center for Communication and Disparities Research, University of Rochester School of Medicine, Family Medicine, James P Wilmot Cancer Center, Rochester, NY, 14642, USA.; (Plumb S) University of Rochester School of Medicine, Family Medicine, Rochester, NY, 14642, USA.; (Tejani MA) James P Wilmot Cancer Center, University of Rochester Medical Center, Rochester, NY, 14642, USA.; (Venuti A) University of Rochester School of Medicine, Family Medicine, Rochester, NY, 14642, USA.; (Epstein RM) Center for Communication and Disparities Research, University of Rochester School of Medicine, Family Medicine, James P Wilmot Cancer Center, Rochester, NY, 14642, USA.
(Elias CM) Department of Statistics, West Lafayette, Purdue University, Human Development & Family Studies, Indiana, 47906, USA.; (Shields CG) Purdue University Center for Cancer Research, Regenstrief Center for Healthcare Engineering, Human Development & Family Studies, Fowler Memorial House, 1200 W State Street, West Lafayette, IN, 47906, USA. cgshields@purdue.edu.; (Griggs JJ) Department of Internal Medicine, Hematology & Oncology Division and Department of Health Management & Policy Ann Arbor, University of Michigan

School of Medicine, Ann Arbor, MI, 48109-0419, USA.; (Fiscella K) Department of Public Health Sciences, University of Rochester School of Medicine, Family Medicine, Rochester, NY, 14642, USA.; (Christ SL) Department of Statistics, West Lafayette, Purdue University, Human Development & Family Studies, Indiana, 47906, USA.; (Colbert J) Biostatistics Department, School of Public Health, University of Michigan, Ann Arbor, MI, 48109, 14642, USA.; (Henry SG) Department of Internal Medicine, University of California Davis School of Medicine, Sacramento, CA, University of Rochester Medical Center, Rochester, NY, USA.; (Hoh BG) Department of Internal Medicine, University of California Davis School of Medicine, Sacramento, CA, University of Rochester Medical Center, Rochester, NY, USA.; (Hunte HER) West Virginia University, Robert C. Byrd Health Sciences Center, Morgantown, West VA, 26506, USA.; (Marshall M) Department of Statistics, West Lafayette, Purdue University, Human Development & Family Studies, Indiana, 47906, USA.; (Mohile SG) Center for Communication and Disparities Research, University of Rochester School of Medicine, Family Medicine, James P Wilmot Cancer Center, Rochester, NY, 14642, USA.; (Plumb S) University of Rochester School of Medicine, Family Medicine, Rochester, NY, 14642, USA.; (Tejani MA) James P Wilmot Cancer Center, University of Rochester Medical Center, Rochester, NY, 14642, USA.; (Venuti A) University of Rochester School of Medicine, Family Medicine, Rochester, NY, 14642, USA.; (Epstein RM) Center for Communication and Disparities Research, University of Rochester School of Medicine, Family Medicine, James P Wilmot Cancer Center, Rochester, NY, 14642, USA.
(Elias CM) Department of Statistics, West Lafayette, Purdue University, Human Development & Family Studies, Indiana, 47906, USA.; (Shields CG) Purdue University Center for Cancer Research, Regenstrief Center for Healthcare Engineering, Human Development & Family Studies, Fowler Memorial House, 1200 W State Street, West Lafayette, IN, 47906, USA. cgshields@purdue.edu.; (Griggs JJ) Department of Internal Medicine, Hematology & Oncology Division and Department of Health Management & Policy Ann Arbor, University of Michigan School of Medicine, Ann Arbor, MI, 48109-0419, USA.; (Fiscella K) Department of Public Health Sciences, University of Rochester School of Medicine, Family Medicine, Rochester, NY, 14642, USA.; (Christ SL) Department of Statistics, West Lafayette, Purdue University, Human Development & Family Studies, Indiana, 47906, USA.; (Colbert J) Biostatistics Department, School of Public Health, University of Michigan, Ann Arbor, MI, 48109, 14642, USA.; (Henry SG) Department of Internal Medicine, University of California Davis School of Medicine, Sacramento, CA, University of Rochester Medical Center, Rochester, NY, USA.; (Hoh BG) Department of Internal Medicine, University of California Davis School of Medicine, Sacramento, CA, University of Rochester Medical Center, Rochester, NY, USA.; (Hunte HER) West Virginia University, Robert C. Byrd Health Sciences Center, Morgantown, West VA, 26506, USA.; (Marshall M) Department of Statistics, West Lafayette, Purdue University, Human Development & Family Studies, Indiana, 47906, USA.; (Mohile SG) Center for Communication and Disparities Research, University of Rochester School of Medicine, Family Medicine, James P Wilmot Cancer Center, Rochester, NY, 14642, USA.; (Plumb S) University of Rochester School of Medicine, Family Medicine, Rochester, NY, 14642, USA.; (Tejani MA) James P Wilmot Cancer Center, University of Rochester Medical Center, Rochester, NY, 14642, USA.; (Venuti A) University of Rochester School of Medicine, Family Medicine, Rochester, NY, 14642, USA.; (Epstein RM) Center for Communication and

Disparities Research, University of Rochester School of Medicine, Family Medicine, James P Wilmot Cancer Center, Rochester, NY, 14642, USA.
(Elias CM) Department of Statistics, West Lafayette, Purdue University, Human Development & Family Studies, Indiana, 47906, USA.; (Shields CG) Purdue University Center for Cancer Research, Regenstrief Center for Healthcare Engineering, Human Development & Family Studies, Fowler Memorial House, 1200 W State Street, West Lafayette, IN, 47906, USA. cgshields@purdue.edu.; (Griggs JJ) Department of Internal Medicine, Hematology & Oncology Division and Department of Health Management & Policy Ann Arbor, University of Michigan School of Medicine, Ann Arbor, MI, 48109-0419, USA.; (Fiscella K) Department of Public Health Sciences, University of Rochester School of Medicine, Family Medicine, Rochester, NY, 14642, USA.; (Christ SL) Department of Statistics, West Lafayette, Purdue University, Human Development & Family Studies, Indiana, 47906, USA.; (Colbert J) Biostatistics Department, School of Public Health, University of Michigan, Ann Arbor, MI, 48109, 14642, USA.; (Henry SG) Department of Internal Medicine, University of California Davis School of Medicine, Sacramento, CA, University of Rochester Medical Center, Rochester, NY, USA.; (Hoh BG) Department of Internal Medicine, University of California Davis School of Medicine, Sacramento, CA, University of Rochester Medical Center, Rochester, NY, USA.; (Hunte HER) West Virginia University, Robert C. Byrd Health Sciences Center, Morgantown, West VA, 26506, USA.; (Marshall M) Department of Statistics, West Lafayette, Purdue University, Human Development & Family Studies, Indiana, 47906, USA.; (Mohile SG) Center for Communication and Disparities Research, University of Rochester School of Medicine, Family Medicine, James P Wilmot Cancer Center, Rochester, NY, 14642, USA.; (Plumb S) University of Rochester School of Medicine, Family Medicine, Rochester, NY, 14642, USA.; (Tejani MA) James P Wilmot Cancer Center, University of Rochester Medical Center, Rochester, NY, 14642, USA.; (Venuti A) University of Rochester School of Medicine, Family Medicine, Rochester, NY, 14642, USA.; (Epstein RM) Center for Communication and Disparities Research, University of Rochester School of Medicine, Family Medicine, James P Wilmot Cancer Center, Rochester, NY, 14642, USA.
(Elias CM) Department of Statistics, West Lafayette, Purdue University, Human Development & Family Studies, Indiana, 47906, USA.; (Shields CG) Purdue University Center for Cancer Research, Regenstrief Center for Healthcare Engineering, Human Development & Family Studies, Fowler Memorial House, 1200 W State Street, West Lafayette, IN, 47906, USA. cgshields@purdue.edu.; (Griggs JJ) Department of Internal Medicine, Hematology & Oncology Division and Department of Health Management & Policy Ann Arbor, University of Michigan School of Medicine, Ann Arbor, MI, 48109-0419, USA.; (Fiscella K) Department of Public Health Sciences, University of Rochester School of Medicine, Family Medicine, Rochester, NY, 14642, USA.; (Christ SL) Department of Statistics, West Lafayette, Purdue University, Human Development & Family Studies, Indiana, 47906, USA.; (Colbert J) Biostatistics Department, School of Public Health, University of Michigan, Ann Arbor, MI, 48109, 14642, USA.; (Henry SG) Department of Internal Medicine, University of California Davis School of Medicine, Sacramento, CA, University of Rochester Medical Center, Rochester, NY, USA.; (Hoh BG) Department of Internal Medicine, University of California Davis School of Medicine, Sacramento, CA, University of Rochester Medical Center, Rochester, NY, USA.; (Hunte HER) West Virginia University, Robert C. Byrd Health Sciences Center, Morgantown, West VA, 26506, USA.; (Marshall M) Department of Statistics, West

Lafayette, Purdue University, Human Development & Family Studies, Indiana, 47906, USA.; (Mohile SG) Center for Communication and Disparities Research, University of Rochester School of Medicine, Family Medicine, James P Wilmot Cancer Center, Rochester, NY, 14642, USA.; (Plumb S) University of Rochester School of Medicine, Family Medicine, Rochester, NY, 14642, USA.; (Tejani MA) James P Wilmot Cancer Center, University of Rochester Medical Center, Rochester, NY, 14642, USA.; (Venuti A) University of Rochester School of Medicine, Family Medicine, Rochester, NY, 14642, USA.; (Epstein RM) Center for Communication and Disparities Research, University of Rochester School of Medicine, Family Medicine, James P Wilmot Cancer Center, Rochester, NY, 14642, USA.
Database: PubMed

82. Development and Testing of an Intelligent Pain Management System (IPMS) on Mobile Phones Through a Randomized Trial Among Chinese Cancer Patients: A New Approach in Cancer Pain Management.

Author(s): Sun Y; Jiang F; Gu JJ; Wang YK; Hua H; Li J; Cheng Z; Liao Z; Huang Q; Hu W; Ding G

Source: JMIR mHealth and uHealth; Jul 2017; vol. 5 (no. 7); p. e108

Publication Date: Jul 2017

Publication Type(s): Journal Article

DOI: http://dx.doi.org/10.2196/mhealth.7178

ISSN: 2291-5222

Place of Publication: Canada

PubMedID: 28743681

Accession Number: 28743681

Available at JMIR mHealth and uHealth - from Europe PubMed Central - Open Access

Available at JMIR mHealth and uHealth - from ProQuest (Health Research Premium) - NHS Version

Available at JMIR mHealth and uHealth - from Unpaywall

Keywords: Subject Terms: cancer pain; intelligent pain management system; intervention; smart phone

Abstract:BACKGROUND: Cancer has become increasingly prevalent in China over the past few decades. Among the factors that determine the quality of life of cancer patients, pain has commonly been recognized as a most critical one; it could also lead to the ineffective treatment of the cancer. Driven by the need for better pain management for cancer patients, our research team developed a mobile-based Intelligent Pain Management System (IPMS).OBJECTIVE: Our objective was to design, develop, and test the IPMS to facilitate real-time pain recording and timely intervention among cancer patients with pain. The system's usability, feasibility, compliance, and satisfaction were also assessed.METHODS: A sample of 46 patients with cancer pain symptoms were recruited at the Oncology Center of Xinhua Hospital affiliated to Shanghai Jiao Tong University School of Medicine, Chongming Branch (hereinafter referred to as "the Oncology Center"). In a pretest, participants completed a pain management knowledge questionnaire and were evaluated using

the baseline cancer pain assessment and Karnofsky Performance Status (KPS) evaluation. The participants were then randomly assigned into two groups (the trial group and the control group). After a 14-day trial period, another round of cancer pain assessment, KPS evaluation and pain management knowledge assessment were repeated. In the trial group, the data were fully automatically collected by the IPMS. In the control group, the data were collected using conventional methods, such as phone interviews or door-to-door visits by physicians. The participants were also asked to complete a satisfaction questionnaire on the use of the IPMS.RESULTS: All participants successfully completed the trial. First, the feasibility of IPMS by observing the number of daily pain assessments recorded among patients was assessed. Second, the users' satisfaction, effectiveness of pain management, and changes in the quality of their lives were evaluated. All the participants gave high satisfaction score after they used IMPS. Both groups reported similar pain scores and KPS scores at the baseline. At the end of the trial, the mean pain score of the trial group was significantly lower than of the control group (P<.001). The ending KPS score of the trial group was significantly higher than of the control group (P<.001). The improvement of pain management knowledge score in the trial group was more pronounced than that in the control group (P<.001).CONCLUSIONS: This study provided preliminary data to support the potentials of using IPMS in cancer pain communication between patients and doctors and to provide real-time supportive intervention on a convenient basis at a low cost. Overall, the IPMS can serve as a reliable and effective approach to control cancer pain and improve quality of life for patients with cancer pain.TRIAL REGISTRATION: Clinicaltrials.gov NCT02765269; http://clinicaltrials.gov/ct2/show/NCT02765269 (Archived by WebCite at http://www.webcitation.org/6rnwsgDgv).

Institutions:

(Sun Y) Xinhua Hospital Affiliated to Shanghai Jiao Tong University School of Medicine, Shanghai, China.; (Jiang F) Xinhua Hospital Affiliated to Shanghai Jiao Tong University School of Medicine, Chongming Branch, Shanghai, China.; (Gu JJ) Lymphoma Translational Research Laboratory, Department of Medicine, Roswell Park Cancer Institute, Buffalo, NY, United States.; (Wang YK) Division of Management and Education, University of Pittsburgh at Bradford, Bradford, PA, United States.; (Hua H) Xinhua Hospital Affiliated to Shanghai Jiao Tong University School of Medicine, Shanghai, China.; (Li J) Xinhua Hospital Affiliated to Shanghai Jiao Tong University School of Medicine, Shanghai, China.; (Cheng Z) Xinhua Hospital Affiliated to Shanghai Jiao Tong University School of Medicine, Chongming Branch, Shanghai, China.; (Liao Z) Xinhua Hospital Affiliated to Shanghai Jiao Tong University School of Medicine, Chongming Branch, Shanghai, China.; (Huang Q) Xinhua Hospital Affiliated to Shanghai Jiao Tong University School of Medicine, Chongming Branch, Shanghai, China.; (Hu W) Xinhua Hospital Affiliated to Shanghai Jiao Tong University School of Medicine, Shanghai, China.; (Ding G) Xinhua Hospital Affiliated to Shanghai Jiao Tong University School of Medicine, Shanghai, China.
(Sun Y) Xinhua Hospital Affiliated to Shanghai Jiao Tong University School of Medicine, Shanghai, China.; (Jiang F) Xinhua Hospital Affiliated to Shanghai Jiao Tong University School of Medicine, Chongming Branch, Shanghai, China.; (Gu JJ) Lymphoma Translational Research Laboratory, Department of Medicine, Roswell Park Cancer Institute, Buffalo, NY, United States.; (Wang YK) Division of Management and Education, University of Pittsburgh at Bradford, Bradford, PA, United States.; (Hua H) Xinhua Hospital Affiliated to Shanghai Jiao Tong University

School of Medicine, Shanghai, China.; (Li J) Xinhua Hospital Affiliated to Shanghai Jiao Tong University School of Medicine, Shanghai, China.; (Cheng Z) Xinhua Hospital Affiliated to Shanghai Jiao Tong University School of Medicine, Chongming Branch, Shanghai, China.; (Liao Z) Xinhua Hospital Affiliated to Shanghai Jiao Tong University School of Medicine, Chongming Branch, Shanghai, China.; (Huang Q) Xinhua Hospital Affiliated to Shanghai Jiao Tong University School of Medicine, Chongming Branch, Shanghai, China.; (Hu W) Xinhua Hospital Affiliated to Shanghai Jiao Tong University School of Medicine, Shanghai, China.; (Ding G) Xinhua Hospital Affiliated to Shanghai Jiao Tong University School of Medicine, Shanghai, China.
(Sun Y) Xinhua Hospital Affiliated to Shanghai Jiao Tong University School of Medicine, Shanghai, China.; (Jiang F) Xinhua Hospital Affiliated to Shanghai Jiao Tong University School of Medicine, Chongming Branch, Shanghai, China.; (Gu JJ) Lymphoma Translational Research Laboratory, Department of Medicine, Roswell Park Cancer Institute, Buffalo, NY, United States.; (Wang YK) Division of Management and Education, University of Pittsburgh at Bradford, Bradford, PA, United States.; (Hua H) Xinhua Hospital Affiliated to Shanghai Jiao Tong University School of Medicine, Shanghai, China.; (Li J) Xinhua Hospital Affiliated to Shanghai Jiao Tong University School of Medicine, Shanghai, China.; (Cheng Z) Xinhua Hospital Affiliated to Shanghai Jiao Tong University School of Medicine, Chongming Branch, Shanghai, China.; (Liao Z) Xinhua Hospital Affiliated to Shanghai Jiao Tong University School of Medicine, Chongming Branch, Shanghai, China.; (Huang Q) Xinhua Hospital Affiliated to Shanghai Jiao Tong University School of Medicine, Chongming Branch, Shanghai, China.; (Hu W) Xinhua Hospital Affiliated to Shanghai Jiao Tong University School of Medicine, Shanghai, China.; (Ding G) Xinhua Hospital Affiliated to Shanghai Jiao Tong University School of Medicine, Shanghai, China.
(Sun Y) Xinhua Hospital Affiliated to Shanghai Jiao Tong University School of Medicine, Shanghai, China.; (Jiang F) Xinhua Hospital Affiliated to Shanghai Jiao Tong University School of Medicine, Chongming Branch, Shanghai, China.; (Gu JJ) Lymphoma Translational Research Laboratory, Department of Medicine, Roswell Park Cancer Institute, Buffalo, NY, United States.; (Wang YK) Division of Management and Education, University of Pittsburgh at Bradford, Bradford, PA, United States.; (Hua H) Xinhua Hospital Affiliated to Shanghai Jiao Tong University School of Medicine, Shanghai, China.; (Li J) Xinhua Hospital Affiliated to Shanghai Jiao Tong University School of Medicine, Shanghai, China.; (Cheng Z) Xinhua Hospital Affiliated to Shanghai Jiao Tong University School of Medicine, Chongming Branch, Shanghai, China.; (Liao Z) Xinhua Hospital Affiliated to Shanghai Jiao Tong University School of Medicine, Chongming Branch, Shanghai, China.; (Huang Q) Xinhua Hospital Affiliated to Shanghai Jiao Tong University School of Medicine, Chongming Branch, Shanghai, China.; (Hu W) Xinhua Hospital Affiliated to Shanghai Jiao Tong University School of Medicine, Shanghai, China.; (Ding G) Xinhua Hospital Affiliated to Shanghai Jiao Tong University School of Medicine, Shanghai, China.
(Sun Y) Xinhua Hospital Affiliated to Shanghai Jiao Tong University School of Medicine, Shanghai, China.; (Jiang F) Xinhua Hospital Affiliated to Shanghai Jiao Tong University School of Medicine, Chongming Branch, Shanghai, China.; (Gu JJ) Lymphoma Translational Research Laboratory, Department of Medicine, Roswell Park Cancer Institute, Buffalo, NY, United States.; (Wang YK) Division of Management and Education, University of Pittsburgh at Bradford, Bradford, PA, United States.; (Hua H) Xinhua Hospital Affiliated to Shanghai Jiao Tong University School of Medicine, Shanghai, China.; (Li J) Xinhua Hospital Affiliated to Shanghai Jiao Tong University School of Medicine, Shanghai, China.; (Cheng Z) Xinhua

Hospital Affiliated to Shanghai Jiao Tong University School of Medicine, Chongming Branch, Shanghai, China.; (Liao Z) Xinhua Hospital Affiliated to Shanghai Jiao Tong University School of Medicine, Chongming Branch, Shanghai, China.; (Huang Q) Xinhua Hospital Affiliated to Shanghai Jiao Tong University School of Medicine, Chongming Branch, Shanghai, China.; (Hu W) Xinhua Hospital Affiliated to Shanghai Jiao Tong University School of Medicine, Shanghai, China.; (Ding G) Xinhua Hospital Affiliated to Shanghai Jiao Tong University School of Medicine, Shanghai, China.
(Sun Y) Xinhua Hospital Affiliated to Shanghai Jiao Tong University School of Medicine, Shanghai, China.; (Jiang F) Xinhua Hospital Affiliated to Shanghai Jiao Tong University School of Medicine, Chongming Branch, Shanghai, China.; (Gu JJ) Lymphoma Translational Research Laboratory, Department of Medicine, Roswell Park Cancer Institute, Buffalo, NY, United States.; (Wang YK) Division of Management and Education, University of Pittsburgh at Bradford, Bradford, PA, United States.; (Hua H) Xinhua Hospital Affiliated to Shanghai Jiao Tong University School of Medicine, Shanghai, China.; (Li J) Xinhua Hospital Affiliated to Shanghai Jiao Tong University School of Medicine, Shanghai, China.; (Cheng Z) Xinhua Hospital Affiliated to Shanghai Jiao Tong University School of Medicine, Chongming Branch, Shanghai, China.; (Liao Z) Xinhua Hospital Affiliated to Shanghai Jiao Tong University School of Medicine, Chongming Branch, Shanghai, China.; (Huang Q) Xinhua Hospital Affiliated to Shanghai Jiao Tong University School of Medicine, Chongming Branch, Shanghai, China.; (Hu W) Xinhua Hospital Affiliated to Shanghai Jiao Tong University School of Medicine, Shanghai, China.; (Ding G) Xinhua Hospital Affiliated to Shanghai Jiao Tong University School of Medicine, Shanghai, China.
(Sun Y) Xinhua Hospital Affiliated to Shanghai Jiao Tong University School of Medicine, Shanghai, China.; (Jiang F) Xinhua Hospital Affiliated to Shanghai Jiao Tong University School of Medicine, Chongming Branch, Shanghai, China.; (Gu JJ) Lymphoma Translational Research Laboratory, Department of Medicine, Roswell Park Cancer Institute, Buffalo, NY, United States.; (Wang YK) Division of Management and Education, University of Pittsburgh at Bradford, Bradford, PA, United States.; (Hua H) Xinhua Hospital Affiliated to Shanghai Jiao Tong University School of Medicine, Shanghai, China.; (Li J) Xinhua Hospital Affiliated to Shanghai Jiao Tong University School of Medicine, Shanghai, China.; (Cheng Z) Xinhua Hospital Affiliated to Shanghai Jiao Tong University School of Medicine, Chongming Branch, Shanghai, China.; (Liao Z) Xinhua Hospital Affiliated to Shanghai Jiao Tong University School of Medicine, Chongming Branch, Shanghai, China.; (Huang Q) Xinhua Hospital Affiliated to Shanghai Jiao Tong University School of Medicine, Chongming Branch, Shanghai, China.; (Hu W) Xinhua Hospital Affiliated to Shanghai Jiao Tong University School of Medicine, Shanghai, China.; (Ding G) Xinhua Hospital Affiliated to Shanghai Jiao Tong University School of Medicine, Shanghai, China.
(Sun Y) Xinhua Hospital Affiliated to Shanghai Jiao Tong University School of Medicine, Shanghai, China.; (Jiang F) Xinhua Hospital Affiliated to Shanghai Jiao Tong University School of Medicine, Chongming Branch, Shanghai, China.; (Gu JJ) Lymphoma Translational Research Laboratory, Department of Medicine, Roswell Park Cancer Institute, Buffalo, NY, United States.; (Wang YK) Division of Management and Education, University of Pittsburgh at Bradford, Bradford, PA, United States.; (Hua H) Xinhua Hospital Affiliated to Shanghai Jiao Tong University School of Medicine, Shanghai, China.; (Li J) Xinhua Hospital Affiliated to Shanghai Jiao Tong University School of Medicine, Shanghai, China.; (Cheng Z) Xinhua Hospital Affiliated to Shanghai Jiao Tong University School of Medicine, Chongming Branch, Shanghai, China.; (Liao Z) Xinhua Hospital Affiliated to Shanghai Jiao Tong

University School of Medicine, Chongming Branch, Shanghai, China.; (Huang Q)
Xinhua Hospital Affiliated to Shanghai Jiao Tong University School of Medicine,
Chongming Branch, Shanghai, China.; (Hu W) Xinhua Hospital Affiliated to Shanghai
Jiao Tong University School of Medicine, Shanghai, China.; (Ding G) Xinhua Hospital
Affiliated to Shanghai Jiao Tong University School of Medicine, Shanghai, China.
(Sun Y) Xinhua Hospital Affiliated to Shanghai Jiao Tong University School of
Medicine, Shanghai, China.; (Jiang F) Xinhua Hospital Affiliated to Shanghai Jiao
Tong University School of Medicine, Chongming Branch, Shanghai, China.; (Gu JJ)
Lymphoma Translational Research Laboratory, Department of Medicine, Roswell
Park Cancer Institute, Buffalo, NY, United States.; (Wang YK) Division of
Management and Education, University of Pittsburgh at Bradford, Bradford, PA,
United States.; (Hua H) Xinhua Hospital Affiliated to Shanghai Jiao Tong University
School of Medicine, Shanghai, China.; (Li J) Xinhua Hospital Affiliated to Shanghai
Jiao Tong University School of Medicine, Shanghai, China.; (Cheng Z) Xinhua
Hospital Affiliated to Shanghai Jiao Tong University School of Medicine, Chongming
Branch, Shanghai, China.; (Liao Z) Xinhua Hospital Affiliated to Shanghai Jiao Tong
University School of Medicine, Chongming Branch, Shanghai, China.; (Huang Q)
Xinhua Hospital Affiliated to Shanghai Jiao Tong University School of Medicine,
Chongming Branch, Shanghai, China.; (Hu W) Xinhua Hospital Affiliated to Shanghai
Jiao Tong University School of Medicine, Shanghai, China.; (Ding G) Xinhua Hospital
Affiliated to Shanghai Jiao Tong University School of Medicine, Shanghai, China.
(Sun Y) Xinhua Hospital Affiliated to Shanghai Jiao Tong University School of
Medicine, Shanghai, China.; (Jiang F) Xinhua Hospital Affiliated to Shanghai Jiao
Tong University School of Medicine, Chongming Branch, Shanghai, China.; (Gu JJ)
Lymphoma Translational Research Laboratory, Department of Medicine, Roswell
Park Cancer Institute, Buffalo, NY, United States.; (Wang YK) Division of
Management and Education, University of Pittsburgh at Bradford, Bradford, PA,
United States.; (Hua H) Xinhua Hospital Affiliated to Shanghai Jiao Tong University
School of Medicine, Shanghai, China.; (Li J) Xinhua Hospital Affiliated to Shanghai
Jiao Tong University School of Medicine, Shanghai, China.; (Cheng Z) Xinhua
Hospital Affiliated to Shanghai Jiao Tong University School of Medicine, Chongming
Branch, Shanghai, China.; (Liao Z) Xinhua Hospital Affiliated to Shanghai Jiao Tong
University School of Medicine, Chongming Branch, Shanghai, China.; (Huang Q)
Xinhua Hospital Affiliated to Shanghai Jiao Tong University School of Medicine,
Chongming Branch, Shanghai, China.; (Hu W) Xinhua Hospital Affiliated to Shanghai
Jiao Tong University School of Medicine, Shanghai, China.; (Ding G) Xinhua Hospital
Affiliated to Shanghai Jiao Tong University School of Medicine, Shanghai, China.
(Sun Y) Xinhua Hospital Affiliated to Shanghai Jiao Tong University School of
Medicine, Shanghai, China.; (Jiang F) Xinhua Hospital Affiliated to Shanghai Jiao
Tong University School of Medicine, Chongming Branch, Shanghai, China.; (Gu JJ)
Lymphoma Translational Research Laboratory, Department of Medicine, Roswell
Park Cancer Institute, Buffalo, NY, United States.; (Wang YK) Division of
Management and Education, University of Pittsburgh at Bradford, Bradford, PA,
United States.; (Hua H) Xinhua Hospital Affiliated to Shanghai Jiao Tong University
School of Medicine, Shanghai, China.; (Li J) Xinhua Hospital Affiliated to Shanghai
Jiao Tong University School of Medicine, Shanghai, China.; (Cheng Z) Xinhua
Hospital Affiliated to Shanghai Jiao Tong University School of Medicine, Chongming
Branch, Shanghai, China.; (Liao Z) Xinhua Hospital Affiliated to Shanghai Jiao Tong
University School of Medicine, Chongming Branch, Shanghai, China.; (Huang Q)
Xinhua Hospital Affiliated to Shanghai Jiao Tong University School of Medicine,

Chongming Branch, Shanghai, China.; (Hu W) Xinhua Hospital Affiliated to Shanghai Jiao Tong University School of Medicine, Shanghai, China.; (Ding G) Xinhua Hospital Affiliated to Shanghai Jiao Tong University School of Medicine, Shanghai, China.
Database: PubMed

83. Co-creative development of an eHealth nursing intervention: Self-management support for outpatients with cancer pain.

Author(s): Hochstenbach LMJ; Courtens AM; Zwakhalen SMG; Vermeulen J; van Kleef M; de Witte LP

Source: Applied nursing research : ANR; 2017; vol. 36 ; p. 1-8

Publication Date: 2017

Publication Type(s): Journal Article; Research Support, Non-U.S. Gov't

DOI: http://dx.doi.org/10.1016/j.apnr.2017.03.004

ISSN: 1532-8201

Place of Publication: United States

PubMedID: 28720227

Accession Number: 28720227

Available at Applied Nursing Research - from Unpaywall

Keywords: Subject Terms: *Cancer pain; *Intervention development; *Nursing; *Outpatients; *Self-management; *eHealth

Abstract:INTRODUCTION: Co-creative methods, having an iterative character and including different perspectives, allow for the development of complex nursing interventions. Information about the development process is essential in providing justification for the ultimate intervention and crucial in interpreting the outcomes of subsequent evaluations. This paper describes a co-creative method directed towards the development of an eHealth intervention delivered by registered nurses to support self-management in outpatients with cancer pain.METHODS: Intervention development was divided into three consecutive phases (exploration of context, specification of content, organisation of care). In each phase, researchers and technicians addressed five iterative steps: research, ideas, prototyping, evaluation, and documentation. Health professionals and patients were consulted during research and evaluation steps.RESULTS: Collaboration of researchers, health professionals, patients and technicians was positive and valuable in optimising outcomes. The intervention includes a mobile application for patients and a web application for nurses. Patients are requested to monitor pain, adverse effects and medication intake, while being provided with graphical feedback, education and contact possibilities. Nurses monitor data, advise patients, and collaborate with the treating physician.CONCLUSION: Integration of patient self-management and professional care by means of eHealth key into well-known barriers and seem promising in improving cancer pain follow-up. Nurses are able to make substantial contributions because of their expertise, focus on daily living, and their bridging function between patients and health professionals in different care settings. Insights from the intervention development as well as the intervention content give thought for applications in different patients and care settings.

Institutions:

(Hochstenbach LMJ) Care and Public Health Research Institute (CAPHRI), Department of Health Services Research, Maastricht University, Maastricht, The Netherlands; Centre of Expertise for Innovative Care and Technology (EIZT), Zuyd University of Applied Sciences, Heerlen, The Netherlands. Electronic address: laura.hochstenbach@zuyd.nl.; (Courtens AM) Centre of Expertise for Palliative Care (EPZM), Department of Patient and Care, Maastricht University Medical Centre (MUMC+), Maastricht, The Netherlands.; (Zwakhalen SMG) Care and Public Health Research Institute (CAPHRI), Department of Health Services Research, Maastricht University, Maastricht, The Netherlands.; (Vermeulen J) Care and Public Health Research Institute (CAPHRI), Department of Health Services Research, Maastricht University, Maastricht, The Netherlands.; (van Kleef M) School for Mental Health and Neuroscience (MHeNs), Department of Anaesthesiology, Maastricht University Medical Centre (MUMC+), Maastricht, The Netherlands.; (de Witte LP) Care and Public Health Research Institute (CAPHRI), Department of Health Services Research, Maastricht University, Maastricht, The Netherlands; Centre of Expertise for Innovative Care and Technology (EIZT), Zuyd University of Applied Sciences, Heerlen, The Netherlands; Centre for Assistive Technology and Connected Healthcare (CATCH), University of Sheffield, Sheffield, United Kingdom.
(Hochstenbach LMJ) Care and Public Health Research Institute (CAPHRI), Department of Health Services Research, Maastricht University, Maastricht, The Netherlands; Centre of Expertise for Innovative Care and Technology (EIZT), Zuyd University of Applied Sciences, Heerlen, The Netherlands. Electronic address: laura.hochstenbach@zuyd.nl.; (Courtens AM) Centre of Expertise for Palliative Care (EPZM), Department of Patient and Care, Maastricht University Medical Centre (MUMC+), Maastricht, The Netherlands.; (Zwakhalen SMG) Care and Public Health Research Institute (CAPHRI), Department of Health Services Research, Maastricht University, Maastricht, The Netherlands.; (Vermeulen J) Care and Public Health Research Institute (CAPHRI), Department of Health Services Research, Maastricht University, Maastricht, The Netherlands.; (van Kleef M) School for Mental Health and Neuroscience (MHeNs), Department of Anaesthesiology, Maastricht University Medical Centre (MUMC+), Maastricht, The Netherlands.; (de Witte LP) Care and Public Health Research Institute (CAPHRI), Department of Health Services Research, Maastricht University, Maastricht, The Netherlands; Centre of Expertise for Innovative Care and Technology (EIZT), Zuyd University of Applied Sciences, Heerlen, The Netherlands; Centre for Assistive Technology and Connected Healthcare (CATCH), University of Sheffield, Sheffield, United Kingdom.
(Hochstenbach LMJ) Care and Public Health Research Institute (CAPHRI), Department of Health Services Research, Maastricht University, Maastricht, The Netherlands; Centre of Expertise for Innovative Care and Technology (EIZT), Zuyd University of Applied Sciences, Heerlen, The Netherlands. Electronic address: laura.hochstenbach@zuyd.nl.; (Courtens AM) Centre of Expertise for Palliative Care (EPZM), Department of Patient and Care, Maastricht University Medical Centre (MUMC+), Maastricht, The Netherlands.; (Zwakhalen SMG) Care and Public Health Research Institute (CAPHRI), Department of Health Services Research, Maastricht University, Maastricht, The Netherlands.; (Vermeulen J) Care and Public Health Research Institute (CAPHRI), Department of Health Services Research, Maastricht University, Maastricht, The Netherlands.; (van Kleef M) School for Mental Health and Neuroscience (MHeNs), Department of Anaesthesiology, Maastricht University Medical Centre (MUMC+), Maastricht, The Netherlands.; (de Witte LP) Care and Public Health Research Institute (CAPHRI), Department of Health Services

Research, Maastricht University, Maastricht, The Netherlands; Centre of Expertise for Innovative Care and Technology (EIZT), Zuyd University of Applied Sciences, Heerlen, The Netherlands; Centre for Assistive Technology and Connected Healthcare (CATCH), University of Sheffield, Sheffield, United Kingdom.
(Hochstenbach LMJ) Care and Public Health Research Institute (CAPHRI), Department of Health Services Research, Maastricht University, Maastricht, The Netherlands; Centre of Expertise for Innovative Care and Technology (EIZT), Zuyd University of Applied Sciences, Heerlen, The Netherlands. Electronic address: laura.hochstenbach@zuyd.nl.; (Courtens AM) Centre of Expertise for Palliative Care (EPZM), Department of Patient and Care, Maastricht University Medical Centre (MUMC+), Maastricht, The Netherlands.; (Zwakhalen SMG) Care and Public Health Research Institute (CAPHRI), Department of Health Services Research, Maastricht University, Maastricht, The Netherlands.; (Vermeulen J) Care and Public Health Research Institute (CAPHRI), Department of Health Services Research, Maastricht University, Maastricht, The Netherlands.; (van Kleef M) School for Mental Health and Neuroscience (MHeNs), Department of Anaesthesiology, Maastricht University Medical Centre (MUMC+), Maastricht, The Netherlands.; (de Witte LP) Care and Public Health Research Institute (CAPHRI), Department of Health Services Research, Maastricht University, Maastricht, The Netherlands; Centre of Expertise for Innovative Care and Technology (EIZT), Zuyd University of Applied Sciences, Heerlen, The Netherlands; Centre for Assistive Technology and Connected Healthcare (CATCH), University of Sheffield, Sheffield, United Kingdom.
(Hochstenbach LMJ) Care and Public Health Research Institute (CAPHRI), Department of Health Services Research, Maastricht University, Maastricht, The Netherlands; Centre of Expertise for Innovative Care and Technology (EIZT), Zuyd University of Applied Sciences, Heerlen, The Netherlands. Electronic address: laura.hochstenbach@zuyd.nl.; (Courtens AM) Centre of Expertise for Palliative Care (EPZM), Department of Patient and Care, Maastricht University Medical Centre (MUMC+), Maastricht, The Netherlands.; (Zwakhalen SMG) Care and Public Health Research Institute (CAPHRI), Department of Health Services Research, Maastricht University, Maastricht, The Netherlands.; (Vermeulen J) Care and Public Health Research Institute (CAPHRI), Department of Health Services Research, Maastricht University, Maastricht, The Netherlands.; (van Kleef M) School for Mental Health and Neuroscience (MHeNs), Department of Anaesthesiology, Maastricht University Medical Centre (MUMC+), Maastricht, The Netherlands.; (de Witte LP) Care and Public Health Research Institute (CAPHRI), Department of Health Services Research, Maastricht University, Maastricht, The Netherlands; Centre of Expertise for Innovative Care and Technology (EIZT), Zuyd University of Applied Sciences, Heerlen, The Netherlands; Centre for Assistive Technology and Connected Healthcare (CATCH), University of Sheffield, Sheffield, United Kingdom.
(Hochstenbach LMJ) Care and Public Health Research Institute (CAPHRI), Department of Health Services Research, Maastricht University, Maastricht, The Netherlands; Centre of Expertise for Innovative Care and Technology (EIZT), Zuyd University of Applied Sciences, Heerlen, The Netherlands. Electronic address: laura.hochstenbach@zuyd.nl.; (Courtens AM) Centre of Expertise for Palliative Care (EPZM), Department of Patient and Care, Maastricht University Medical Centre (MUMC+), Maastricht, The Netherlands.; (Zwakhalen SMG) Care and Public Health Research Institute (CAPHRI), Department of Health Services Research, Maastricht University, Maastricht, The Netherlands.; (Vermeulen J) Care and Public Health Research Institute (CAPHRI), Department of Health Services Research, Maastricht

University, Maastricht, The Netherlands.; (van Kleef M) School for Mental Health and Neuroscience (MHeNs), Department of Anaesthesiology, Maastricht University Medical Centre (MUMC+), Maastricht, The Netherlands.; (de Witte LP) Care and Public Health Research Institute (CAPHRI), Department of Health Services Research, Maastricht University, Maastricht, The Netherlands; Centre of Expertise for Innovative Care and Technology (EIZT), Zuyd University of Applied Sciences, Heerlen, The Netherlands; Centre for Assistive Technology and Connected Healthcare (CATCH), University of Sheffield, Sheffield, United Kingdom.
Database: PubMed

84. Testing the Implementation of a Pain Self-management Support Intervention for Oncology Patients in Clinical Practice: A Randomized Controlled Pilot Study (ANtiPain).

Author(s): Koller A; Gaertner J; De Geest S; Hasemann M; Becker G

Source: Cancer nursing; 2018; vol. 41 (no. 5); p. 367-378

Publication Date: 2018

Publication Type(s): Journal Article; Randomized Controlled Trial; Research Support, Non-U.S. Gov't

DOI: http://dx.doi.org/10.1097/NCC.0000000000000502

ISSN: 1538-9804

Place of Publication: United States

PubMedID: 28537957

Accession Number: 28537957

Available at Cancer Nursing - from Ovid (Journals @ Ovid)

Abstract:BACKGROUND: In oncology, pain control is a persistent problem. Significant barriers to cancer pain management are patient related. Pain self-management support interventions have shown to reduce pain intensity and patient-related barriers. Comparative effectiveness research is a suitable approach to test whether effects are sustained in clinical practice.OBJECTIVE: In this pilot randomized controlled trial, the implementation of the ANtiPain intervention into clinical practice was tested to assess the effects on pain intensity, function-related outcomes, self-efficacy, and patient-related barriers to pain management to prepare a larger effectiveness trial.METHODS: Within 14 months, 39 adult oncology patients with pain scores of 3 or higher on a 10-point numeric rating scale were recruited in an academic comprehensive cancer center in Southern Germany. Patients in the control group (n = 19) received standard care. Patients in the intervention group (n = 20) received ANtiPain, a cancer pain self-management support intervention based on 3 key strategies: provision of information, skill building, and nurse coaching. An intervention session was performed in-hospital. After discharge, follow-up was provided via telephone calls. Data were collected at baseline and 1 and 6 weeks after discharge. Effect sizes were calculated for all outcomes.RESULTS: Large effects were found for activity hindrance (Cohen d = 0.90), barriers (d = 0.91), and self-efficacy (d = 0.90). Small to moderate effects were found for average and worst pain (Cohen d = 0.17-0.45).CONCLUSIONS: Key findings of this study involved function-related outcomes and self-efficacy.IMPLICATIONS FOR PRACTICE: Because these outcomes are particularly meaningful for patients, the integration of

ANtiPain to routine clinical practice may be substantial. A larger study will be based on these findings.

Institutions:
(Koller A) Author Affiliations: Department of Internal Medicine, University Medical Center Freiburg, Germany (Dr Koller and Mrs Hasemann); Clinic for Palliative Care, Medical Center, University of Freiburg, Faculty of Medicine, University of Freiburg, Germany (Drs Koller, Gaertner, and Becker and Mrs Hasemann); Palliative Care Center Hildegard, Basel, Switzerland (Dr Gaertner); Institute of Nursing Science, Faculty of Social Sciences, University of Vienna, Austria (Dr Koller); Institute of Nursing Science, Faculty of Medicine, University of Basel, Switzerland (Drs Koller and De Geest); and Academic Center for Nursing and Midwifery, KU Leuven, Belgium (Dr De Geest).
Database: PubMed

85. Cancer-Related Pain Assessment: Monitoring the Effectiveness of Interventions.

Author(s): Gallagher E; Rogers BB; Brant JM

Source: Clinical journal of oncology nursing; ; vol. 21 (no. 3 Suppl); p. 8-12

Publication Type(s): Journal Article; Review

DOI: http://dx.doi.org/10.1188/17.CJON.S3.8-12

ISSN: 1538-067X

Place of Publication: United States

PubMedID: 28524910

Accession Number: 28524910

Available at Clinical journal of oncology nursing - from Ovid (Journals @ Ovid)

Available at Clinical journal of oncology nursing - from EBSCO (CINAHL Complete)

Available at Clinical journal of oncology nursing - from ProQuest (Health Research Premium) - NHS Version

Available at Clinical journal of oncology nursing - from ProQuest (MEDLINE with Full Text) - NHS Version

Keywords: Subject Terms: *Assessment; *cancer-related pain; *management ; *verbal and nonverbal patients

Abstract:BACKGROUND: Cancer-related pain is a major health problem because of its magnitude, the subjective nature of the pain experience, and the complexity of the disease, making it difficult to assess and control. When assessment is not performed, poor pain control can result. .OBJECTIVES: This article provides an overview of the components of a comprehensive cancer pain assessment. .METHODS: A review of the healthcare literature was performed. .FINDINGS: Nurses play a key role in pain assessment by establishing patient trust and rapport, which helps to break down barriers that may stand in the way of effective pain assessment and management.

Institutions:
(Gallagher E) Agios Pharmaceuticals.; (Rogers BB) Fox Chase Cancer Center, Philadelphia, PA.; (Brant JM) Billings Clinic Hospital.

(Gallagher E) Agios Pharmaceuticals.; (Rogers BB) Fox Chase Cancer Center, Philadelphia, PA.; (Brant JM) Billings Clinic Hospital.
(Gallagher E) Agios Pharmaceuticals.; (Rogers BB) Fox Chase Cancer Center, Philadelphia, PA.; (Brant JM) Billings Clinic Hospital.
Database: PubMed

86. [Assessment and Strategy for Nociceptive Pain in Cancer].

Author(s): Osawa G; Aruga E

Source: Gan to kagaku ryoho. Cancer & chemotherapy; Apr 2017; vol. 44 (no. 4); p. 278-282

Publication Date: Apr 2017

Publication Type(s): Journal Article

ISSN: 0385-0684

Place of Publication: Japan

PubMedID: 28428504

Accession Number: 28428504

Abstract: Pain is classified as either nociceptive pain, which results from a nociceptor stimulation, or neuropathic pain, which results from a lesion of the neural pathway. Clinically, in many cases, pain consists of a single origin but multiple origins are also possible. In this paper, we outline an assessment and strategy for nociceptive pain in cancer. The onset time, location, feature, strength, and etiology were included as categories for the assessment of pain. Furthermore, we added a psychosocial assessment to the physical assessment, which ensured comprehensive pain evaluation. We performed the investigation according to the WHO method for cancer pain relief. According to the effects and adverse effects, we chose a non-opioid analgesic and an opioid analgesic as medications. However, nonpharmacological therapy, such as radiotherapy and nerve block, is not a concern with the WHO analgesic ladder. It is important that a multi-disciplinary team, which includes physicians, nurses, physiotherapists, and pharmacists, support the patients to manage their pain by themselves through the knowledge of the factors affecting their pain and the medication methods for easing it when it gets worse. Although we cannot ease pain completely, it is important to define the goal of treatment with patients and to examine the strategy for maximum pain relief.

Institutions:
(Osawa G) Dept. of Palliative Medicine, Teikyo University School of Medicine.
Database: PubMed

87. Mapping French people and health professionals' positions regarding the circumstances of morphine use to relieve cancer pain.

Author(s): Mazoyer J; Sastre MTM; Sorum PC; Mullet E

Source: Supportive care in cancer : official journal of the Multinational Association of Supportive Care in Cancer; 2017; vol. 25 (no. 9); p. 2723-2731

Publication Date: 2017

Publication Type(s): Journal Article

DOI: http://dx.doi.org/10.1007/s00520-017-3682-z
ISSN: 1433-7339
Place of Publication: Germany
PubMedID: 28364172
Accession Number: 28364172

Available at Supportive care in cancer : official journal of the Multinational Association of Supportive Care in Cancer - from SpringerLink

Available at Supportive care in cancer : official journal of the Multinational Association of Supportive Care in Cancer - from EBSCO (MEDLINE Complete)

Available at Supportive care in cancer : official journal of the Multinational Association of Supportive Care in Cancer - from EBSCO (CINAHL Complete)

Available at Supportive care in cancer : official journal of the Multinational Association of Supportive Care in Cancer - from ProQuest (MEDLINE with Full Text) - NHS Version

Available at Supportive care in cancer : official journal of the Multinational Association of Supportive Care in Cancer - from ProQuest (Health Research Premium) - NHS Version

Keywords: Subject Terms: *French people; *Health professionals; *Morphine use

Abstract:BACKGROUND: Cancer patients suffer in part because some health professionals prescribe or administer amounts of analgesics, namely opioids, which are too small or too widely spaced to be fully effective. Patients' reluctance to use opioids for pain treatment is often mentioned as a reason not to apply the official guidelines, but very few studies have been conducted on people's attitudes about opioid use to relieve cancer pain.METHODS: One hundred twenty lay participants and 30 health professionals (7 physicians and 23 nurses) were presented with a set of vignettes describing a terminally ill woman with cancer who is in pain. The vignettes were composed according to a four within-subject factor design: (a) level of pain reported by the patient, (b) patient's explicit request for additional administration of analgesics, (c) the physicians' final decision (e.g., to use a stronger analgesic combining paracetamol and codeine), and (d) the way the decision was made (collectively or not). Participants were asked to assess the extent to which the physician's decision was, in their view, acceptable.RESULTS: Seven qualitatively different positions were found among participants. They were called as follows: tend to disagree with any decision (9%), increase the strength of the painkiller in any case (16%), give morphine preferentially (9%), partly depends on pain level (23%), fully depends on pain level (6%), depends on decision process and on pain level (22%), and tend not to disagree with any collective decision (25%).CONCLUSIONS: Overall, 91% of participants agreed with the use of morphine in terminally ill cancer patients when the pain level was high (score of 7) and the decision to increase the strength of the painkiller was taken collectively. This percentage dropped to 69% when the team was not involved in the decision and to 40% when the pain level was lower (score of 4). If opposition to the use of morphine exists, it is not opposition to morphine itself but opposition to the circumstances of its use.

Institutions:
(Mazoyer J) Jean-Jaurès University of Toulouse, Toulouse, France.; (Sastre MTM) Jean-Jaurès University of Toulouse, Toulouse, France.; (Sorum PC) Albany Medical

College, Albany, NY, USA.; (Mullet E) Institute of Advanced Studies (EPHE), Paris, France. etienne.mullet@wanadoo.fr.
(Mazoyer J) Jean-Jaurès University of Toulouse, Toulouse, France.; (Sastre MTM) Jean-Jaurès University of Toulouse, Toulouse, France.; (Sorum PC) Albany Medical College, Albany, NY, USA.; (Mullet E) Institute of Advanced Studies (EPHE), Paris, France. etienne.mullet@wanadoo.fr.
(Mazoyer J) Jean-Jaurès University of Toulouse, Toulouse, France.; (Sastre MTM) Jean-Jaurès University of Toulouse, Toulouse, France.; (Sorum PC) Albany Medical College, Albany, NY, USA.; (Mullet E) Institute of Advanced Studies (EPHE), Paris, France. etienne.mullet@wanadoo.fr.
(Mazoyer J) Jean-Jaurès University of Toulouse, Toulouse, France.; (Sastre MTM) Jean-Jaurès University of Toulouse, Toulouse, France.; (Sorum PC) Albany Medical College, Albany, NY, USA.; (Mullet E) Institute of Advanced Studies (EPHE), Paris, France. etienne.mullet@wanadoo.fr.
Database: PubMed

88. Can A Complex Online Intervention Improve Cancer Nurses' Pain Screening and Assessment Practices? Results from a Multicenter, Pre-post Test Pilot Study.

Author(s): Phillips JL; Heneka N; Hickman L; Lam L; Shaw T

Source: Pain management nursing : official journal of the American Society of Pain Management Nurses; Apr 2017; vol. 18 (no. 2); p. 75-89

Publication Date: Apr 2017

Publication Type(s): Journal Article; Multicenter Study

DOI: http://dx.doi.org/10.1016/j.pmn.2017.01.003

ISSN: 1532-8635

Place of Publication: United States

PubMedID: 28363326

Accession Number: 28363326

Available at Pain Management Nursing - from Unpaywall

Abstract: Unrelieved cancer pain has an adverse impact on quality of life. While routine screening and assessment forms the basis of effective cancer pain management, it is often poorly done, thus contributing to the burden of unrelieved cancer pain. The aim of this study was to test the impact of an online, complex, evidence-based educational intervention on cancer nurses' pain assessment capabilities and adherence to cancer pain screening and assessment guidelines. Specialist inpatient cancer nurses in five Australian acute care settings participated in an intervention combining an online spaced learning cancer pain assessment module with audit and feedback of pain assessment practices. Participants' self-perceived pain assessment competencies were measured at three time points. Prospective, consecutive chart audits were undertaken to appraise nurses' adherence with pain screening and assessment guidelines. The differences in documented pre-post pain assessment practices were benchmarked and fed back to all sites post intervention. Data were analyzed using inferential statistics. Participants who completed the intervention (n = 44) increased their pain assessment knowledge, assessment tool knowledge, and confidence undertaking a pain assessment

(p < .001). The positive changes in nurses' pain assessment capabilities translated into a significant increasing linear trend in the proportion of documented pain assessments in patients' charts at the three time points (χ2 trend = 18.28, df = 1, p < .001). There is evidence that learning content delivered using a spaced learning format, augmented with pain assessment audit and feedback data, improves inpatient cancer nurses' self-perceived pain screening and assessment capabilities and strengthens cancer pain guideline adherence.

Institutions:
(Phillips JL) University of Technology Sydney, Centre for Cardiovascular and Chronic Care, Sydney, Australia. Electronic address: jane.phillips@uts.edu.au.; (Heneka N) University of Technology Sydney, Centre for Cardiovascular and Chronic Care, Sydney, Australia.; (Hickman L) University of Technology Sydney, Centre for Cardiovascular and Chronic Care, Sydney, Australia.; (Lam L) University of Technology Sydney, Centre for Cardiovascular and Chronic Care, Sydney, Australia.; (Shaw T) University of Technology Sydney, Centre for Cardiovascular and Chronic Care, Sydney, Australia.
(Phillips JL) University of Technology Sydney, Centre for Cardiovascular and Chronic Care, Sydney, Australia. Electronic address: jane.phillips@uts.edu.au.; (Heneka N) University of Technology Sydney, Centre for Cardiovascular and Chronic Care, Sydney, Australia.; (Hickman L) University of Technology Sydney, Centre for Cardiovascular and Chronic Care, Sydney, Australia.; (Lam L) University of Technology Sydney, Centre for Cardiovascular and Chronic Care, Sydney, Australia.; (Shaw T) University of Technology Sydney, Centre for Cardiovascular and Chronic Care, Sydney, Australia.
(Phillips JL) University of Technology Sydney, Centre for Cardiovascular and Chronic Care, Sydney, Australia. Electronic address: jane.phillips@uts.edu.au.; (Heneka N) University of Technology Sydney, Centre for Cardiovascular and Chronic Care, Sydney, Australia.; (Hickman L) University of Technology Sydney, Centre for Cardiovascular and Chronic Care, Sydney, Australia.; (Lam L) University of Technology Sydney, Centre for Cardiovascular and Chronic Care, Sydney, Australia.; (Shaw T) University of Technology Sydney, Centre for Cardiovascular and Chronic Care, Sydney, Australia.
(Phillips JL) University of Technology Sydney, Centre for Cardiovascular and Chronic Care, Sydney, Australia. Electronic address: jane.phillips@uts.edu.au.; (Heneka N) University of Technology Sydney, Centre for Cardiovascular and Chronic Care, Sydney, Australia.; (Hickman L) University of Technology Sydney, Centre for Cardiovascular and Chronic Care, Sydney, Australia.; (Lam L) University of Technology Sydney, Centre for Cardiovascular and Chronic Care, Sydney, Australia.; (Shaw T) University of Technology Sydney, Centre for Cardiovascular and Chronic Care, Sydney, Australia.
(Phillips JL) University of Technology Sydney, Centre for Cardiovascular and Chronic Care, Sydney, Australia. Electronic address: jane.phillips@uts.edu.au.; (Heneka N) University of Technology Sydney, Centre for Cardiovascular and Chronic Care, Sydney, Australia.; (Hickman L) University of Technology Sydney, Centre for Cardiovascular and Chronic Care, Sydney, Australia.; (Lam L) University of Technology Sydney, Centre for Cardiovascular and Chronic Care, Sydney, Australia.; (Shaw T) University of Technology Sydney, Centre for Cardiovascular and Chronic Care, Sydney, Australia.

Database: PubMed

89. Prescription Opioid Abuse in Chronic Pain: An Updated Review of Opioid Abuse Predictors and Strategies to Curb Opioid Abuse: Part 1.

Author(s): Kaye AD; Jones MR; Kaye AM; Ripoll JG; Galan V; Beakley BD; Calixto F; Bolden JL; Urman RD; Manchikanti L

Source: Pain physician; 2017; vol. 20 (no. 2S); p. S93-S109

Publication Date: 2017

Publication Type(s): Journal Article; Review

ISSN: 2150-1149

Place of Publication: United States

PubMedID: 28226333

Accession Number: 28226333

Available at Pain physician - from EBSCO (MEDLINE Complete)

Abstract: Chronic pain and prescription opioid abuse are extremely prevalent both in this country and worldwide. Consequences of opioid misuse can be life-threatening with significant morbidity and mortality, exacting a heavy toll on patients, physicians, and society. Individuals with chronic pain and co-occurring substance use disorders and/or mental health disorders, are at a higher risk for misuse of prescribed opioids. Opioid abuse and misuse occurs for a variety of reasons, including self-medication, use for reward, compulsive use because of addiction, and diversion for profit. There is a significant need for treatment approaches that balance treating chronic pain; while minimizing risks for opioid abuse, misuse, and diversion. The use of chronic opioid therapy for chronic non-cancer pain has increased dramatically in the past 2 decades in conjunction with associated increases in the abuse of prescribed opioids and accidental opioid overdoses. Consequently, a validated screening instrument which provides an effective and rational method of selecting patients for opioid therapy, predicting risk, and identifying problems once they arise could be of enormous benefit in clinical practice. Such an instrument could potentially curb the risk of iatrogenic addiction. Although several screening instruments and strategies have been introduced in recent years, there is no single test or instrument which can reliably and accurately predict those patients not suitable for opioid therapy or identify those who need increased vigilance or monitoring during therapy. At present, screening for opioid abuse includes assessment of premorbid and comorbid substance abuse; assessment of aberrant drug-related behaviors; risk factor stratification; and utilization of opioid assessment screening tools. Multiple opioid assessment screening tools and instruments have been developed by various authors. In addition, urine drug testing, monitoring of prescribing practices, prescription monitoring programs, opioid treatment agreements, and utilization of universal precautions are essential. Presently, a combination of strategies is recommended to stratify risk, to identify and understand aberrant drug related behaviors, and to tailor treatments accordingly. This manuscript builds on the 2012 opioid guidelines published in Pain Physician and the 2016 guidelines released by the Centers for Disease Control and Prevention. It reviews the current state of knowledge regarding the growing problem of opioid abuse and misuse; known risk factors; and methods of predicting, assessing, monitoring, and addressing opioid abuse and misuse in patients with chronic non-cancer pain.Key words: Opioids,

misuse, abuse, chronic pain, prevalence, risk assessment, risk management, drug monitoring, aberrant drug-related behavior.

Institutions:
(Kaye AD) Department of Anesthesiology, Louisiana State University Health New Orleans.; (Jones MR) Department of Anesthesiology, Tulane School of Medicine, New Orleans, LA.; (Kaye AM) Georgia Pain Care, Atlanta, GA.; (Ripoll JG) Department of Anesthesiology, Tulane School of Medicine, New Orleans, LA.; (Galan V) Department of Anesthesiology, Tulane School of Medicine, New Orleans, LA.; (Beakley BD) Louisiana State University.
(Kaye AD) Department of Anesthesiology, Louisiana State University Health New Orleans.; (Jones MR) Department of Anesthesiology, Tulane School of Medicine, New Orleans, LA.; (Kaye AM) Georgia Pain Care, Atlanta, GA.; (Ripoll JG) Department of Anesthesiology, Tulane School of Medicine, New Orleans, LA.; (Galan V) Department of Anesthesiology, Tulane School of Medicine, New Orleans, LA.; (Beakley BD) Louisiana State University.
(Kaye AD) Department of Anesthesiology, Louisiana State University Health New Orleans.; (Jones MR) Department of Anesthesiology, Tulane School of Medicine, New Orleans, LA.; (Kaye AM) Georgia Pain Care, Atlanta, GA.; (Ripoll JG) Department of Anesthesiology, Tulane School of Medicine, New Orleans, LA.; (Galan V) Department of Anesthesiology, Tulane School of Medicine, New Orleans, LA.; (Beakley BD) Louisiana State University.
(Kaye AD) Department of Anesthesiology, Louisiana State University Health New Orleans.; (Jones MR) Department of Anesthesiology, Tulane School of Medicine, New Orleans, LA.; (Kaye AM) Georgia Pain Care, Atlanta, GA.; (Ripoll JG) Department of Anesthesiology, Tulane School of Medicine, New Orleans, LA.; (Galan V) Department of Anesthesiology, Tulane School of Medicine, New Orleans, LA.; (Beakley BD) Louisiana State University.
(Kaye AD) Department of Anesthesiology, Louisiana State University Health New Orleans.; (Jones MR) Department of Anesthesiology, Tulane School of Medicine, New Orleans, LA.; (Kaye AM) Georgia Pain Care, Atlanta, GA.; (Ripoll JG) Department of Anesthesiology, Tulane School of Medicine, New Orleans, LA.; (Galan V) Department of Anesthesiology, Tulane School of Medicine, New Orleans, LA.; (Beakley BD) Louisiana State University.
(Kaye AD) Department of Anesthesiology, Louisiana State University Health New Orleans.; (Jones MR) Department of Anesthesiology, Tulane School of Medicine, New Orleans, LA.; (Kaye AM) Georgia Pain Care, Atlanta, GA.; (Ripoll JG) Department of Anesthesiology, Tulane School of Medicine, New Orleans, LA.; (Galan V) Department of Anesthesiology, Tulane School of Medicine, New Orleans, LA.; (Beakley BD) Louisiana State University.

Database: PubMed

90. Barriers to venipuncture-induced pain prevention in cancer patients: a qualitative study.

Author(s): Filbet M; Larkin P; Chabloz C; Chirac A; Monsarrat L; Ruer M; Rhondali W; Collin C

Source: BMC palliative care; Jan 2017; vol. 16 (no. 1); p. 5

Publication Date: Jan 2017

Publication Type(s): Journal Article

DOI: http://dx.doi.org/10.1186/s12904-016-0180-x

ISSN: 1472-684X

Place of Publication: England

PubMedID: 28095834

Accession Number: 28095834

Available at BMC palliative care - from BioMed Central

Available at BMC palliative care - from Europe PubMed Central - Open Access

Available at BMC palliative care - from ProQuest (Health Research Premium) - NHS Version

Available at BMC palliative care - from Unpaywall

Keywords: Subject Terms: Cancer; Incidental pain; Nursing; Procedural pain; Prophylaxis

Abstract:BACKGROUND: Procedural pain reduces the quality of life of cancer patients. Although there are recommendations for its prevention, there are some obstacles for its management. The purpose of this study was to analyze the barriers to procedural pain prophylaxis in cancer patients reflecting the views of the nurses.METHODS: We used qualitative methodology based on semi-structured interviews conducted with nurses, focusing on practices of venipuncture-induced and needle change for implantable central venous access port (ICVAP) pain management in cancer patients. A thematic analysis approach informed the data analysis.RESULTS: Interviews were conducted with 17 nurses. The study highlighted 4 main themes; technical and relational obstacles, nurses' professional recognition, the role of the team, and organizational issues. Participants understood the painful nature of venipuncture. Despite being aware of the benefits of the anesthetic patch, they did not utilize it in a systematic way. We identified several barriers at different levels: technical, relational and previous experience of incident pain. Several organizational issues were also highlighted (e.g. lack of protocol, lack of time).CONCLUSIONS: The prevention of venipuncture-induced cancer pain requires a structured training program, which should reflect the views of nurses in clinical practice.

Institutions:
(Filbet M) Department of Palliative Care, Centre Hospitalier de Lyon-Sud, Hospices Civils de Lyon, 165 Chemin du Grand Revoyet, 69310, Pierre-Bénite, France. marilene.filbet@chu-lyon.fr.; (Larkin P) UCD School of Nursing, Midwifery and Health Systems & Our Lady's Hospice and Care Services University College Dublin, Stillorgan Rd, Belfield, Co., Dublin, Ireland.; (Chabloz C) Coordination for the Evaluation of Professional Practices in Healthcare in the Rhône-Alpes Region, 162 Avenue Lacassagne Bâtiment A - 7ème étage, 69424, Lyon Cedex 03, France.; (Chirac A) Department of Palliative Care, Centre Hospitalier de Lyon-Sud, Hospices Civils de Lyon, 165 Chemin du Grand Revoyet, 69310, Pierre-Bénite, France.; (Monsarrat L) Department of Palliative Care, Centre Hospitalier de Lyon-Sud, Hospices Civils de Lyon, 165 Chemin du Grand Revoyet, 69310, Pierre-Bénite, France.; (Ruer M) Department of Palliative Care, Centre Hospitalier de Lyon-Sud, Hospices Civils de Lyon, 165 Chemin du Grand Revoyet, 69310, Pierre-Bénite, France.; (Rhondali W) Department of Palliative Care, Centre Hospitalier de Lyon-Sud, Hospices Civils de Lyon, 165 Chemin du Grand Revoyet, 69310, Pierre-Bénite, France.; (Collin C) Coordination for the Evaluation of Professional Practices in

Healthcare in the Rhône-Alpes Region, 162 Avenue Lacassagne Bâtiment A - 7ème étage, 69424, Lyon Cedex 03, France.
(Filbet M) Department of Palliative Care, Centre Hospitalier de Lyon-Sud, Hospices Civils de Lyon, 165 Chemin du Grand Revoyet, 69310, Pierre-Bénite, France. marilene.filbet@chu-lyon.fr.; (Larkin P) UCD School of Nursing, Midwifery and Health Systems & Our Lady's Hospice and Care Services University College Dublin, Stillorgan Rd, Belfield, Co., Dublin, Ireland.; (Chabloz C) Coordination for the Evaluation of Professional Practices in Healthcare in the Rhône-Alpes Region, 162 Avenue Lacassagne Bâtiment A - 7ème étage, 69424, Lyon Cedex 03, France.; (Chirac A) Department of Palliative Care, Centre Hospitalier de Lyon-Sud, Hospices Civils de Lyon, 165 Chemin du Grand Revoyet, 69310, Pierre-Bénite, France.; (Monsarrat L) Department of Palliative Care, Centre Hospitalier de Lyon-Sud, Hospices Civils de Lyon, 165 Chemin du Grand Revoyet, 69310, Pierre-Bénite, France.; (Ruer M) Department of Palliative Care, Centre Hospitalier de Lyon-Sud, Hospices Civils de Lyon, 165 Chemin du Grand Revoyet, 69310, Pierre-Bénite, France.; (Rhondali W) Department of Palliative Care, Centre Hospitalier de Lyon-Sud, Hospices Civils de Lyon, 165 Chemin du Grand Revoyet, 69310, Pierre-Bénite, France.; (Collin C) Coordination for the Evaluation of Professional Practices in Healthcare in the Rhône-Alpes Region, 162 Avenue Lacassagne Bâtiment A - 7ème étage, 69424, Lyon Cedex 03, France.
(Filbet M) Department of Palliative Care, Centre Hospitalier de Lyon-Sud, Hospices Civils de Lyon, 165 Chemin du Grand Revoyet, 69310, Pierre-Bénite, France. marilene.filbet@chu-lyon.fr.; (Larkin P) UCD School of Nursing, Midwifery and Health Systems & Our Lady's Hospice and Care Services University College Dublin, Stillorgan Rd, Belfield, Co., Dublin, Ireland.; (Chabloz C) Coordination for the Evaluation of Professional Practices in Healthcare in the Rhône-Alpes Region, 162 Avenue Lacassagne Bâtiment A - 7ème étage, 69424, Lyon Cedex 03, France.; (Chirac A) Department of Palliative Care, Centre Hospitalier de Lyon-Sud, Hospices Civils de Lyon, 165 Chemin du Grand Revoyet, 69310, Pierre-Bénite, France.; (Monsarrat L) Department of Palliative Care, Centre Hospitalier de Lyon-Sud, Hospices Civils de Lyon, 165 Chemin du Grand Revoyet, 69310, Pierre-Bénite, France.; (Ruer M) Department of Palliative Care, Centre Hospitalier de Lyon-Sud, Hospices Civils de Lyon, 165 Chemin du Grand Revoyet, 69310, Pierre-Bénite, France.; (Rhondali W) Department of Palliative Care, Centre Hospitalier de Lyon-Sud, Hospices Civils de Lyon, 165 Chemin du Grand Revoyet, 69310, Pierre-Bénite, France.; (Collin C) Coordination for the Evaluation of Professional Practices in Healthcare in the Rhône-Alpes Region, 162 Avenue Lacassagne Bâtiment A - 7ème étage, 69424, Lyon Cedex 03, France.
(Filbet M) Department of Palliative Care, Centre Hospitalier de Lyon-Sud, Hospices Civils de Lyon, 165 Chemin du Grand Revoyet, 69310, Pierre-Bénite, France. marilene.filbet@chu-lyon.fr.; (Larkin P) UCD School of Nursing, Midwifery and Health Systems & Our Lady's Hospice and Care Services University College Dublin, Stillorgan Rd, Belfield, Co., Dublin, Ireland.; (Chabloz C) Coordination for the Evaluation of Professional Practices in Healthcare in the Rhône-Alpes Region, 162 Avenue Lacassagne Bâtiment A - 7ème étage, 69424, Lyon Cedex 03, France.; (Chirac A) Department of Palliative Care, Centre Hospitalier de Lyon-Sud, Hospices Civils de Lyon, 165 Chemin du Grand Revoyet, 69310, Pierre-Bénite, France.; (Monsarrat L) Department of Palliative Care, Centre Hospitalier de Lyon-Sud, Hospices Civils de Lyon, 165 Chemin du Grand Revoyet, 69310, Pierre-Bénite, France.; (Ruer M) Department of Palliative Care, Centre Hospitalier de Lyon-Sud,

Hospices Civils de Lyon, 165 Chemin du Grand Revoyet, 69310, Pierre-Bénite, France.; (Rhondali W) Department of Palliative Care, Centre Hospitalier de Lyon-Sud, Hospices Civils de Lyon, 165 Chemin du Grand Revoyet, 69310, Pierre-Bénite, France.; (Collin C) Coordination for the Evaluation of Professional Practices in Healthcare in the Rhône-Alpes Region, 162 Avenue Lacassagne Bâtiment A - 7ème étage, 69424, Lyon Cedex 03, France.
(Filbet M) Department of Palliative Care, Centre Hospitalier de Lyon-Sud, Hospices Civils de Lyon, 165 Chemin du Grand Revoyet, 69310, Pierre-Bénite, France. marilene.filbet@chu-lyon.fr.; (Larkin P) UCD School of Nursing, Midwifery and Health Systems & Our Lady's Hospice and Care Services University College Dublin, Stillorgan Rd, Belfield, Co., Dublin, Ireland.; (Chabloz C) Coordination for the Evaluation of Professional Practices in Healthcare in the Rhône-Alpes Region, 162 Avenue Lacassagne Bâtiment A - 7ème étage, 69424, Lyon Cedex 03, France.; (Chirac A) Department of Palliative Care, Centre Hospitalier de Lyon-Sud, Hospices Civils de Lyon, 165 Chemin du Grand Revoyet, 69310, Pierre-Bénite, France.; (Monsarrat L) Department of Palliative Care, Centre Hospitalier de Lyon-Sud, Hospices Civils de Lyon, 165 Chemin du Grand Revoyet, 69310, Pierre-Bénite, France.; (Ruer M) Department of Palliative Care, Centre Hospitalier de Lyon-Sud, Hospices Civils de Lyon, 165 Chemin du Grand Revoyet, 69310, Pierre-Bénite, France.; (Rhondali W) Department of Palliative Care, Centre Hospitalier de Lyon-Sud, Hospices Civils de Lyon, 165 Chemin du Grand Revoyet, 69310, Pierre-Bénite, France.; (Collin C) Coordination for the Evaluation of Professional Practices in Healthcare in the Rhône-Alpes Region, 162 Avenue Lacassagne Bâtiment A - 7ème étage, 69424, Lyon Cedex 03, France.
(Filbet M) Department of Palliative Care, Centre Hospitalier de Lyon-Sud, Hospices Civils de Lyon, 165 Chemin du Grand Revoyet, 69310, Pierre-Bénite, France. marilene.filbet@chu-lyon.fr.; (Larkin P) UCD School of Nursing, Midwifery and Health Systems & Our Lady's Hospice and Care Services University College Dublin, Stillorgan Rd, Belfield, Co., Dublin, Ireland.; (Chabloz C) Coordination for the Evaluation of Professional Practices in Healthcare in the Rhône-Alpes Region, 162 Avenue Lacassagne Bâtiment A - 7ème étage, 69424, Lyon Cedex 03, France.; (Chirac A) Department of Palliative Care, Centre Hospitalier de Lyon-Sud, Hospices Civils de Lyon, 165 Chemin du Grand Revoyet, 69310, Pierre-Bénite, France.; (Monsarrat L) Department of Palliative Care, Centre Hospitalier de Lyon-Sud, Hospices Civils de Lyon, 165 Chemin du Grand Revoyet, 69310, Pierre-Bénite, France.; (Ruer M) Department of Palliative Care, Centre Hospitalier de Lyon-Sud, Hospices Civils de Lyon, 165 Chemin du Grand Revoyet, 69310, Pierre-Bénite, France.; (Rhondali W) Department of Palliative Care, Centre Hospitalier de Lyon-Sud, Hospices Civils de Lyon, 165 Chemin du Grand Revoyet, 69310, Pierre-Bénite, France.; (Collin C) Coordination for the Evaluation of Professional Practices in Healthcare in the Rhône-Alpes Region, 162 Avenue Lacassagne Bâtiment A - 7ème étage, 69424, Lyon Cedex 03, France.
(Filbet M) Department of Palliative Care, Centre Hospitalier de Lyon-Sud, Hospices Civils de Lyon, 165 Chemin du Grand Revoyet, 69310, Pierre-Bénite, France. marilene.filbet@chu-lyon.fr.; (Larkin P) UCD School of Nursing, Midwifery and Health Systems & Our Lady's Hospice and Care Services University College Dublin, Stillorgan Rd, Belfield, Co., Dublin, Ireland.; (Chabloz C) Coordination for the Evaluation of Professional Practices in Healthcare in the Rhône-Alpes Region, 162 Avenue Lacassagne Bâtiment A - 7ème étage, 69424, Lyon Cedex 03, France.; (Chirac A) Department of Palliative Care, Centre Hospitalier de Lyon-Sud, Hospices

Civils de Lyon, 165 Chemin du Grand Revoyet, 69310, Pierre-Bénite, France.; (Monsarrat L) Department of Palliative Care, Centre Hospitalier de Lyon-Sud, Hospices Civils de Lyon, 165 Chemin du Grand Revoyet, 69310, Pierre-Bénite, France.; (Ruer M) Department of Palliative Care, Centre Hospitalier de Lyon-Sud, Hospices Civils de Lyon, 165 Chemin du Grand Revoyet, 69310, Pierre-Bénite, France.; (Rhondali W) Department of Palliative Care, Centre Hospitalier de Lyon-Sud, Hospices Civils de Lyon, 165 Chemin du Grand Revoyet, 69310, Pierre-Bénite, France.; (Collin C) Coordination for the Evaluation of Professional Practices in Healthcare in the Rhône-Alpes Region, 162 Avenue Lacassagne Bâtiment A - 7ème étage, 69424, Lyon Cedex 03, France.
(Filbet M) Department of Palliative Care, Centre Hospitalier de Lyon-Sud, Hospices Civils de Lyon, 165 Chemin du Grand Revoyet, 69310, Pierre-Bénite, France. marilene.filbet@chu-lyon.fr.; (Larkin P) UCD School of Nursing, Midwifery and Health Systems & Our Lady's Hospice and Care Services University College Dublin, Stillorgan Rd, Belfield, Co., Dublin, Ireland.; (Chabloz C) Coordination for the Evaluation of Professional Practices in Healthcare in the Rhône-Alpes Region, 162 Avenue Lacassagne Bâtiment A - 7ème étage, 69424, Lyon Cedex 03, France.; (Chirac A) Department of Palliative Care, Centre Hospitalier de Lyon-Sud, Hospices Civils de Lyon, 165 Chemin du Grand Revoyet, 69310, Pierre-Bénite, France.; (Monsarrat L) Department of Palliative Care, Centre Hospitalier de Lyon-Sud, Hospices Civils de Lyon, 165 Chemin du Grand Revoyet, 69310, Pierre-Bénite, France.; (Ruer M) Department of Palliative Care, Centre Hospitalier de Lyon-Sud, Hospices Civils de Lyon, 165 Chemin du Grand Revoyet, 69310, Pierre-Bénite, France.; (Rhondali W) Department of Palliative Care, Centre Hospitalier de Lyon-Sud, Hospices Civils de Lyon, 165 Chemin du Grand Revoyet, 69310, Pierre-Bénite, France.; (Collin C) Coordination for the Evaluation of Professional Practices in Healthcare in the Rhône-Alpes Region, 162 Avenue Lacassagne Bâtiment A - 7ème étage, 69424, Lyon Cedex 03, France.
Database: PubMed

91. Healthcare Providers' Knowledge and Current Practice of Pain Assessment and Management: How Much Progress Have We Made?

Author(s): Nuseir K; Kassab M; Almomani B

Source: Pain research & management; 2016; vol. 2016 ; p. 8432973

Publication Date: 2016

Publication Type(s): Journal Article; Research Support, Non-U.S. Gov't

DOI: http://dx.doi.org/10.1155/2016/8432973

ISSN: 1918-1523

Place of Publication: United States

PubMedID: 27965524

Accession Number: 27965524

Available at Pain research & management - from Europe PubMed Central - Open Access

Available at Pain research & management - from Hindawi Open Access Journals

Available at Pain research & management - from EBSCO (MEDLINE Complete)

Available at Pain research & management - from Unpaywall

Abstract: Context. Despite improvement in pain management and availability of clinical treatment guidelines, patients in Jordan are still suffering from pain. Negative consequences of undertreated pain are being recognized as a reason for further illnesses and poor quality of life. Healthcare providers (HCPs) are responsible for relieving pain of their patients. Objective. To evaluate the knowledge and attitudes of HCPs toward pain management in Jordan. Methods. A 16-item questionnaire with agree or disagree options was given to 662 HCPs in seven hospitals in Jordan who volunteered to participate in the study. Following data collection, the responses were coded and entered into SPSS. Results. There was a statistically significant difference ($p < 0.004$) in percentage scores between physicians (36%) and pharmacists (36%) versus nurses (24%). The level of knowledge was the best among physicians, followed by pharmacists specifically in the area of cancer pain management. Nurses scored the lowest for knowledge of pain assessment and management among HCPs. However, HCPs overall scores indicated insufficient knowledge specifically in relation to pain assessment and management among children.

Institutions:
(Nuseir K) Faculty of Pharmacy, Jordan University of Science and Technology, Irbid, Jordan.; (Kassab M) Faculty of Nursing, Jordan University of Science and Technology, Irbid, Jordan; Faculty of Health, University of Technology, Sydney, NSW, Australia; Faculty of Nursing, University of Western Sydney, Sydney, NSW, Australia.; (Almomani B) Faculty of Pharmacy, Jordan University of Science and Technology, Irbid, Jordan.
(Nuseir K) Faculty of Pharmacy, Jordan University of Science and Technology, Irbid, Jordan.; (Kassab M) Faculty of Nursing, Jordan University of Science and Technology, Irbid, Jordan; Faculty of Health, University of Technology, Sydney, NSW, Australia; Faculty of Nursing, University of Western Sydney, Sydney, NSW, Australia.; (Almomani B) Faculty of Pharmacy, Jordan University of Science and Technology, Irbid, Jordan.
(Nuseir K) Faculty of Pharmacy, Jordan University of Science and Technology, Irbid, Jordan.; (Kassab M) Faculty of Nursing, Jordan University of Science and Technology, Irbid, Jordan; Faculty of Health, University of Technology, Sydney, NSW, Australia; Faculty of Nursing, University of Western Sydney, Sydney, NSW, Australia.; (Almomani B) Faculty of Pharmacy, Jordan University of Science and Technology, Irbid, Jordan.

Database: PubMed

92. Pain and social processes for hospice cancer patients: An integrative review.

Author(s): Ehrlich O; Walker RK

Source: European journal of oncology nursing : the official journal of European Oncology Nursing Society; Dec 2016; vol. 25 ; p. 83-89

Publication Date: Dec 2016

Publication Type(s): Journal Article; Review

DOI: http://dx.doi.org/10.1016/j.ejon.2016.10.001

ISSN: 1532-2122

Place of Publication: Scotland

PubMedID: 27865257

Accession Number: 27865257

Available at European journal of oncology nursing : the official journal of European Oncology Nursing Society - from ScienceDirect

Abstract:PURPOSE: Hospice cancer patients experience poorly-controlled pain in spite of widely-disseminated evidence-based guidelines for use by hospice care practitioners. Pain management occurs in the context of the interdisciplinary team, centered on a caring triad in the home: the person with pain, their caregiver, and their nurse. This review: 1) Summarizes what is known about differing ways that members of the hospice caring triad (patients, caregivers, and nurses) interpret and respond to cancer pain, in order to develop a cancer pain social processes theoretical framework, 2) Identifies gaps in understanding of hospice cancer pain social processes, and 3) Identifies framework concepts for research-based clinical practice with potential to improve pain outcomes.METHODS: Our integrative review of the literature resulted in the identification and synthesis of 21 unique studies of cancer pain social processes, which were categorized according to a social processes framework and hospice caring triad member roles, using a social processes concepts matrix.RESULTS: Pain meanings, goals, and related responses vary for persons with pain, caregivers, and nurses. Studies have explored individual social processes concepts or triad member roles. Studies identify the need for pain meaning to be included in hospice pain management plans.CONCLUSIONS: To our knowledge, no single study has generated a framework for hospice cancer pain social processes addressing and incorporating the roles of all three caring triad members. Therefore, comprehensive hospice cancer pain clinical evaluation and interventions plans may be missing key elements of pain management, especially for persons with ongoing poorly controlled pain.

Institutions:
(Ehrlich O) Skinner Hall, Room 122, 651 North Pleasant Street, University of Massachusetts, Amherst, MA 01003-9299, USA. Electronic address: oehrlich@nursing.umass.edu.; (Walker RK) Skinner Hall, Room 122, 651 North Pleasant Street, University of Massachusetts, Amherst, MA 01003-9299, USA. Electronic address: r.walker@nursing.umass.edu.
(Ehrlich O) Skinner Hall, Room 122, 651 North Pleasant Street, University of Massachusetts, Amherst, MA 01003-9299, USA. Electronic address: oehrlich@nursing.umass.edu.; (Walker RK) Skinner Hall, Room 122, 651 North Pleasant Street, University of Massachusetts, Amherst, MA 01003-9299, USA. Electronic address: r.walker@nursing.umass.edu.

Database: PubMed

93. Cancer Pain Management in Developing Countries.

Author(s): Saini S; Bhatnagar S

Source: Indian journal of palliative care; 2016; vol. 22 (no. 4); p. 373-377

Publication Date: 2016

Publication Type(s): Review; Journal Article

DOI: http://dx.doi.org/10.4103/0973-1075.191742

ISSN: 0973-1075

Place of Publication: United States

PubMedID: 27803557

Accession Number: 27803557

Available at Indian journal of palliative care - from Europe PubMed Central - Open Access

Available at Indian journal of palliative care - from EBSCO (CINAHL Complete)

Available at Indian journal of palliative care - from ProQuest (Health Research Premium) - NHS Version

Available at Indian journal of palliative care - from Unpaywall

Keywords: Subject Terms: Cancer pain; Developing countries; Opioid; Palliative care

Abstract: The World Health Organization estimated that more than 60% of the 14 million new cancer cases worldwide in 2012 were reported in the developing part of the world, including Asia, Africa, Central and South America. Cancer survival rate is poorer in developing countries due to diagnosis at late stage and limited access to timely treatment. Since the disease per se cannot be treated even with the best available treatment modalities, what remains important is symptom management and providing comfort care to these patients. The incidence of pain in advanced stages of cancer approaches 70-80%. Lack of preventive strategies, poverty, illiteracy, and social stigma are the biggest cause of pain suffering and patient presenting in advance stage of their disease. The need for palliative care is expanding due to aging of world's population and increase in the rate of cancer in developed and developing countries. A huge gap remains between demand and current palliative care services. Overcoming barriers to palliative care is a major global health agenda that need immediate attention. Main causes of inadequate pain relief remain lack of knowledge among physician and patients, lack of adequate supply of opioids and other drugs for pain relief, strong bureaucracy involved in terms of procurement, and dispensing of opioids. Beside this, poverty and illiteracy remain the most important factors of increased suffering.

Institutions:
(Saini S) Department of Anesthesiology, BLK Super Speciality Hospital, New Delhi, India.; (Bhatnagar S) Department of Oncoanaesthesia and Palliative Medicine, BRAIRCH, AIIMS, New Delhi, India.
(Saini S) Department of Anesthesiology, BLK Super Speciality Hospital, New Delhi, India.; (Bhatnagar S) Department of Oncoanaesthesia and Palliative Medicine, BRAIRCH, AIIMS, New Delhi, India.
Database: PubMed

94. [Patterns of prescription of opioid analgesics in Hôtel-Dieu de France of Beyrouth].

Author(s): Noufi P; Khoury E; Ayoub E; Naccache N; Richa S

Source: L'Encephale; Dec 2016; vol. 42 (no. 6); p. 511-516

Publication Date: Dec 2016

Publication Type(s): Journal Article

DOI: http://dx.doi.org/10.1016/j.encep.2016.05.010

ISSN: 0013-7006

Place of Publication: France

PubMedID: 27776797

Accession Number: 27776797

Keywords: Subject Terms: Addiction; Analgesics; Analgésiques; Chronic pain; Douleur chronique; Dépendance; Modalités de prescription; Opiacés; Opioids; Prescribing practices

Abstract:OBJECTIVES: Use of chronic opioid therapy has increased substantially over the past few years, even though opioid therapy is associated with potentially serious harms, including opioid-related adverse effects and outcomes. Prescription of opioids for chronic pain, particularly nonmalignant chronic pain, remains controversial. In the midst of this controversy, patterns of actual prescription and influences on these patterns are not well understood. This study aims to describe the frequency of prescription of opioid analgesics in a university hospital, the attitudes of doctors towards this category of drugs, and the follow-up modalities of patients taking these drugs. The study also explores the association between the practitioners' characteristics and the modalities of prescription.DESIGN AND METHODS: A survey was delivered to 112 doctors and surgeons in the hospital during the four months between August and December 2013 and it was returned by 55 (49.0%). The survey consists of three parts. The first part addresses the frequency and reluctance of doctors' prescription of opioids and other analgesics for acute and chronic pain. The second part studies the doctors' attitudes and concerns towards opioids. It explores the belief of the doctors in the efficacy of this category of drugs, their confidence in prescribing such medications and the eventual side effects they might worry about. The third part of the survey studies the modalities of evaluation prior to the prescription and the modalities of follow-up of the patients receiving a long-term opioid treatment.RESULTS: Overall, 76.4% of doctors reported they sometimes, frequently, or always, prescribe opioids, which, using the Wilcoxon test, proved to be a significantly lower frequency than for prescribing of minor analgesics or nonsteroidal anti-inflammatory drugs (NSAIDS). Similarly, 60.1% reported a reluctance to prescribe opioids for chronic nonmalignant pain, which was a significantly greater reluctance than for cancer pain. The age and sex of the participants were unrelated to prescribing, but those with specialty training and use of practice guidelines were more likely to prescribe opioids and were less reluctant to do so. A majority of practitioners felt that opioids are effective for the treatment of chronic nonmalignant pain and that they have the sufficient training to prescribe them adequately; however, they still worry about the long-term prescription of opioids, particularly fearing the psychological dependence this treatment might cause. Using a series of Spearman correlation tests, we found that practitioners who thought they were adequately trained and who believed in the efficacy of long-term opioid treatment were more likely to prescribe them but that the worries about side effects decreased the frequency of prescription. A significant proportion of practitioners do not evaluate addiction risk factors of patients before prescribing opioids. The results concerning the modalities of follow-up of prescription were very heterogeneous with 87% of practitioners not explaining and 65% not screening for adverse effects. We similarly found that the frequency of follow-up and the management of patients who were exhibiting signs of dependence were very

diverse.CONCLUSION: The results of this study were compatible with those of other recent studies about opioid prescription. The doctors practicing in the university hospital Hôtel-Dieu de France de Beyrouth present comparable prescription patterns, independent of their personal or professional characteristics, and they are more confident in their prescription when professionally trained for it. However, they exhibit a notable heterogeneity in their attitudes towards opioids and in their modalities of evaluating patients receiving long-term treatment. These results suggest a need for additional training in the management of this category of drugs.
Institutions:
(Noufi P) Hôtel-Dieu de France, faculté de médecine, université Saint-Joseph, BP 166830, Beyrouth, Liban.; (Khoury E) Hôtel-Dieu de France, faculté de médecine, université Saint-Joseph, BP 166830, Beyrouth, Liban.; (Ayoub E) Hôtel-Dieu de France, faculté de médecine, université Saint-Joseph, BP 166830, Beyrouth, Liban.; (Naccache N) Hôtel-Dieu de France, faculté de médecine, université Saint-Joseph, BP 166830, Beyrouth, Liban.; (Richa S) Hôtel-Dieu de France, faculté de médecine, université Saint-Joseph, BP 166830, Beyrouth, Liban. Electronic address: sami.richa@usj.edu.klb.
(Noufi P) Hôtel-Dieu de France, faculté de médecine, université Saint-Joseph, BP 166830, Beyrouth, Liban.; (Khoury E) Hôtel-Dieu de France, faculté de médecine, université Saint-Joseph, BP 166830, Beyrouth, Liban.; (Ayoub E) Hôtel-Dieu de France, faculté de médecine, université Saint-Joseph, BP 166830, Beyrouth, Liban.; (Naccache N) Hôtel-Dieu de France, faculté de médecine, université Saint-Joseph, BP 166830, Beyrouth, Liban.; (Richa S) Hôtel-Dieu de France, faculté de médecine, université Saint-Joseph, BP 166830, Beyrouth, Liban. Electronic address: sami.richa@usj.edu.klb.
(Noufi P) Hôtel-Dieu de France, faculté de médecine, université Saint-Joseph, BP 166830, Beyrouth, Liban.; (Khoury E) Hôtel-Dieu de France, faculté de médecine, université Saint-Joseph, BP 166830, Beyrouth, Liban.; (Ayoub E) Hôtel-Dieu de France, faculté de médecine, université Saint-Joseph, BP 166830, Beyrouth, Liban.; (Naccache N) Hôtel-Dieu de France, faculté de médecine, université Saint-Joseph, BP 166830, Beyrouth, Liban.; (Richa S) Hôtel-Dieu de France, faculté de médecine, université Saint-Joseph, BP 166830, Beyrouth, Liban. Electronic address: sami.richa@usj.edu.klb.
(Noufi P) Hôtel-Dieu de France, faculté de médecine, université Saint-Joseph, BP 166830, Beyrouth, Liban.; (Khoury E) Hôtel-Dieu de France, faculté de médecine, université Saint-Joseph, BP 166830, Beyrouth, Liban.; (Ayoub E) Hôtel-Dieu de France, faculté de médecine, université Saint-Joseph, BP 166830, Beyrouth, Liban.; (Naccache N) Hôtel-Dieu de France, faculté de médecine, université Saint-Joseph, BP 166830, Beyrouth, Liban.; (Richa S) Hôtel-Dieu de France, faculté de médecine, université Saint-Joseph, BP 166830, Beyrouth, Liban. Electronic address: sami.richa@usj.edu.klb.
(Noufi P) Hôtel-Dieu de France, faculté de médecine, université Saint-Joseph, BP 166830, Beyrouth, Liban.; (Khoury E) Hôtel-Dieu de France, faculté de médecine, université Saint-Joseph, BP 166830, Beyrouth, Liban.; (Ayoub E) Hôtel-Dieu de France, faculté de médecine, université Saint-Joseph, BP 166830, Beyrouth, Liban.; (Naccache N) Hôtel-Dieu de France, faculté de médecine, université Saint-Joseph, BP 166830, Beyrouth, Liban.; (Richa S) Hôtel-Dieu de France, faculté de médecine, université Saint-Joseph, BP 166830, Beyrouth, Liban. Electronic address: sami.richa@usj.edu.klb.
Database: PubMed

95. Managing Pain in Patients With Cancer: The Chinese Good Pain Management Experience.

Author(s): Yu SY; Wang JJ; Huang YG; Hu B; Wang K; Li PP; Wu YL; Zhang HL; Zhang L; Zhang QY; Qin SK

Source: Journal of global oncology; Oct 2017; vol. 3 (no. 5); p. 583-595

Publication Date: Oct 2017

Publication Type(s): Journal Article; Review

DOI: http://dx.doi.org/10.1200/JGO.2016.005686

ISSN: 2378-9506

Place of Publication: United States

PubMedID: 29094098

Accession Number: 29094098

Available at Journal of global oncology - from Unpaywall

Abstract: Purpose: The number of cancer cases in China has increased rapidly from 2.1 million in 2000 to 4.3 million in 2015. As a consequence, pain management as an integral part of cancer treatment became an important health care issue. In March 2011, the Good Pain Management (GPM) program was launched to standardize the treatment of cancer pain and improve the quality of life for patients with cancer. With this work, we will describe the GPM program, its implementation experience, and highlight key lessons that can improve pain management for patients with cancer.Methods: We describe procedures for the selection, implementation, and assessment procedures for model cancer wards. We analyzed published results in areas of staff training and patient education, pain management in practice, analgesic drugs administration, and patient follow-up and satisfaction.Results: Pain management training enabled medical staff to accurately assess the level of pain and to provide effective pain relief through timely dispensation of medication. Patients with good knowledge of treatment of pain were able to overcome their aversion to opioid drugs and cooperate with nursing staff on pain assessment to achieve effective drug dose titration. Consumption of strong opioid drugs increased significantly; however, there was no change for weaker opioids. Higher pain remission rates were achieved for patients with moderate-to-severe pain levels. Proper patient follow-up after discharge enabled improved outcomes to be maintained.Conclusion: The GPM program has instituted a consistent and high standard of care for pain management at cancer wards and improved the quality of life for patients with cancer.

Institutions:
(Yu SY) , Huazhong University of Science and Technology and Tongji Hospital, Wuhan; , Shanghai Changzheng Hospital, Shanghai; , Peking Union Medical College Hospital; , Beijing University Cancer Hospital, Beijing; , Anhui Provincial Hospital, Anhui; , Tianjin Medical University Cancer Institute and Hospital, National Clinical Research Center for Cancer, Tianjin; , Guangdong Lung Cancer Institute, Guangdong General Hospital, Guangdong; , Tangdu Hospital and Tangdu Comprehensive Cancer Center, Cancer Institute, 4th Military Medical University, Xi'an; , Sun Yat-Sen University Cancer Center, Guangzhou; , 3rd Affiliated Hospital of Harbin Medical University, Harbin, Heilongjiang; and , The People's Liberation Army

Cancer Centre, Nanjing Bayi Hospital, Nanjing, Jiangsu, People's Republic of China.; (Wang JJ) , Huazhong University of Science and Technology and Tongji Hospital, Wuhan; , Shanghai Changzheng Hospital, Shanghai; , Peking Union Medical College Hospital; , Beijing University Cancer Hospital, Beijing; , Anhui Provincial Hospital, Anhui; , Tianjin Medical University Cancer Institute and Hospital, National Clinical Research Center for Cancer, Tianjin; , Guangdong Lung Cancer Institute, Guangdong General Hospital, Guangdong; , Tangdu Hospital and Tangdu Comprehensive Cancer Center, Cancer Institute, 4th Military Medical University, Xi'an; , Sun Yat-Sen University Cancer Center, Guangzhou; , 3rd Affiliated Hospital of Harbin Medical University, Harbin, Heilongjiang; and , The People's Liberation Army Cancer Centre, Nanjing Bayi Hospital, Nanjing, Jiangsu, People's Republic of China.; (Huang YG) , Huazhong University of Science and Technology and Tongji Hospital, Wuhan; , Shanghai Changzheng Hospital, Shanghai; , Peking Union Medical College Hospital; , Beijing University Cancer Hospital, Beijing; , Anhui Provincial Hospital, Anhui; , Tianjin Medical University Cancer Institute and Hospital, National Clinical Research Center for Cancer, Tianjin; , Guangdong Lung Cancer Institute, Guangdong General Hospital, Guangdong; , Tangdu Hospital and Tangdu Comprehensive Cancer Center, Cancer Institute, 4th Military Medical University, Xi'an; , Sun Yat-Sen University Cancer Center, Guangzhou; , 3rd Affiliated Hospital of Harbin Medical University, Harbin, Heilongjiang; and , The People's Liberation Army Cancer Centre, Nanjing Bayi Hospital, Nanjing, Jiangsu, People's Republic of China.; (Hu B) , Huazhong University of Science and Technology and Tongji Hospital, Wuhan; , Shanghai Changzheng Hospital, Shanghai; , Peking Union Medical College Hospital; , Beijing University Cancer Hospital, Beijing; , Anhui Provincial Hospital, Anhui; , Tianjin Medical University Cancer Institute and Hospital, National Clinical Research Center for Cancer, Tianjin; , Guangdong Lung Cancer Institute, Guangdong General Hospital, Guangdong; , Tangdu Hospital and Tangdu Comprehensive Cancer Center, Cancer Institute, 4th Military Medical University, Xi'an; , Sun Yat-Sen University Cancer Center, Guangzhou; , 3rd Affiliated Hospital of Harbin Medical University, Harbin, Heilongjiang; and , The People's Liberation Army Cancer Centre, Nanjing Bayi Hospital, Nanjing, Jiangsu, People's Republic of China.; (Wang K) , Huazhong University of Science and Technology and Tongji Hospital, Wuhan; , Shanghai Changzheng Hospital, Shanghai; , Peking Union Medical College Hospital; , Beijing University Cancer Hospital, Beijing; , Anhui Provincial Hospital, Anhui; , Tianjin Medical University Cancer Institute and Hospital, National Clinical Research Center for Cancer, Tianjin; , Guangdong Lung Cancer Institute, Guangdong General Hospital, Guangdong; , Tangdu Hospital and Tangdu Comprehensive Cancer Center, Cancer Institute, 4th Military Medical University, Xi'an; , Sun Yat-Sen University Cancer Center, Guangzhou; , 3rd Affiliated Hospital of Harbin Medical University, Harbin, Heilongjiang; and , The People's Liberation Army Cancer Centre, Nanjing Bayi Hospital, Nanjing, Jiangsu, People's Republic of China.; (Li PP) , Huazhong University of Science and Technology and Tongji Hospital, Wuhan; , Shanghai Changzheng Hospital, Shanghai; , Peking Union Medical College Hospital; , Beijing University Cancer Hospital, Beijing; , Anhui Provincial Hospital, Anhui; , Tianjin Medical University Cancer Institute and Hospital, National Clinical Research Center for Cancer, Tianjin; , Guangdong Lung Cancer Institute, Guangdong General Hospital, Guangdong; , Tangdu Hospital and Tangdu Comprehensive Cancer Center, Cancer Institute, 4th Military Medical University, Xi'an; , Sun Yat-Sen University Cancer Center, Guangzhou; , 3rd Affiliated Hospital of Harbin Medical University, Harbin, Heilongjiang; and , The People's Liberation Army

Cancer Centre, Nanjing Bayi Hospital, Nanjing, Jiangsu, People's Republic of China.; (Wu YL) , Huazhong University of Science and Technology and Tongji Hospital, Wuhan; , Shanghai Changzheng Hospital, Shanghai; , Peking Union Medical College Hospital; , Beijing University Cancer Hospital, Beijing; , Anhui Provincial Hospital, Anhui; , Tianjin Medical University Cancer Institute and Hospital, National Clinical Research Center for Cancer, Tianjin; , Guangdong Lung Cancer Institute, Guangdong General Hospital, Guangdong; , Tangdu Hospital and Tangdu Comprehensive Cancer Center, Cancer Institute, 4th Military Medical University, Xi'an; , Sun Yat-Sen University Cancer Center, Guangzhou; , 3rd Affiliated Hospital of Harbin Medical University, Harbin, Heilongjiang; and , The People's Liberation Army Cancer Centre, Nanjing Bayi Hospital, Nanjing, Jiangsu, People's Republic of China.; (Zhang HL) , Huazhong University of Science and Technology and Tongji Hospital, Wuhan; , Shanghai Changzheng Hospital, Shanghai; , Peking Union Medical College Hospital; , Beijing University Cancer Hospital, Beijing; , Anhui Provincial Hospital, Anhui; , Tianjin Medical University Cancer Institute and Hospital, National Clinical Research Center for Cancer, Tianjin; , Guangdong Lung Cancer Institute, Guangdong General Hospital, Guangdong; , Tangdu Hospital and Tangdu Comprehensive Cancer Center, Cancer Institute, 4th Military Medical University, Xi'an; , Sun Yat-Sen University Cancer Center, Guangzhou; , 3rd Affiliated Hospital of Harbin Medical University, Harbin, Heilongjiang; and , The People's Liberation Army Cancer Centre, Nanjing Bayi Hospital, Nanjing, Jiangsu, People's Republic of China.; (Zhang L) , Huazhong University of Science and Technology and Tongji Hospital, Wuhan; , Shanghai Changzheng Hospital, Shanghai; , Peking Union Medical College Hospital; , Beijing University Cancer Hospital, Beijing; , Anhui Provincial Hospital, Anhui; , Tianjin Medical University Cancer Institute and Hospital, National Clinical Research Center for Cancer, Tianjin; , Guangdong Lung Cancer Institute, Guangdong General Hospital, Guangdong; , Tangdu Hospital and Tangdu Comprehensive Cancer Center, Cancer Institute, 4th Military Medical University, Xi'an; , Sun Yat-Sen University Cancer Center, Guangzhou; , 3rd Affiliated Hospital of Harbin Medical University, Harbin, Heilongjiang; and , The People's Liberation Army Cancer Centre, Nanjing Bayi Hospital, Nanjing, Jiangsu, People's Republic of China.; (Zhang QY) , Huazhong University of Science and Technology and Tongji Hospital, Wuhan; , Shanghai Changzheng Hospital, Shanghai; , Peking Union Medical College Hospital; , Beijing University Cancer Hospital, Beijing; , Anhui Provincial Hospital, Anhui; , Tianjin Medical University Cancer Institute and Hospital, National Clinical Research Center for Cancer, Tianjin; , Guangdong Lung Cancer Institute, Guangdong General Hospital, Guangdong; , Tangdu Hospital and Tangdu Comprehensive Cancer Center, Cancer Institute, 4th Military Medical University, Xi'an; , Sun Yat-Sen University Cancer Center, Guangzhou; , 3rd Affiliated Hospital of Harbin Medical University, Harbin, Heilongjiang; and , The People's Liberation Army Cancer Centre, Nanjing Bayi Hospital, Nanjing, Jiangsu, People's Republic of China.; (Qin SK) , Huazhong University of Science and Technology and Tongji Hospital, Wuhan; , Shanghai Changzheng Hospital, Shanghai; , Peking Union Medical College Hospital; , Beijing University Cancer Hospital, Beijing; , Anhui Provincial Hospital, Anhui; , Tianjin Medical University Cancer Institute and Hospital, National Clinical Research Center for Cancer, Tianjin; , Guangdong Lung Cancer Institute, Guangdong General Hospital, Guangdong; , Tangdu Hospital and Tangdu Comprehensive Cancer Center, Cancer Institute, 4th Military Medical University, Xi'an; , Sun Yat-Sen University Cancer Center, Guangzhou; , 3rd Affiliated Hospital of

Harbin Medical University, Harbin, Heilongjiang; and , The People's Liberation Army Cancer Centre, Nanjing Bayi Hospital, Nanjing, Jiangsu, People's Republic of China. (Yu SY) , Huazhong University of Science and Technology and Tongji Hospital, Wuhan; , Shanghai Changzheng Hospital, Shanghai; , Peking Union Medical College Hospital; , Beijing University Cancer Hospital, Beijing; , Anhui Provincial Hospital, Anhui; , Tianjin Medical University Cancer Institute and Hospital, National Clinical Research Center for Cancer, Tianjin; , Guangdong Lung Cancer Institute, Guangdong General Hospital, Guangdong; , Tangdu Hospital and Tangdu Comprehensive Cancer Center, Cancer Institute, 4th Military Medical University, Xi'an; , Sun Yat-Sen University Cancer Center, Guangzhou; , 3rd Affiliated Hospital of Harbin Medical University, Harbin, Heilongjiang; and , The People's Liberation Army Cancer Centre, Nanjing Bayi Hospital, Nanjing, Jiangsu, People's Republic of China.; (Wang JJ) , Huazhong University of Science and Technology and Tongji Hospital, Wuhan; , Shanghai Changzheng Hospital, Shanghai; , Peking Union Medical College Hospital; , Beijing University Cancer Hospital, Beijing; , Anhui Provincial Hospital, Anhui; , Tianjin Medical University Cancer Institute and Hospital, National Clinical Research Center for Cancer, Tianjin; , Guangdong Lung Cancer Institute, Guangdong General Hospital, Guangdong; , Tangdu Hospital and Tangdu Comprehensive Cancer Center, Cancer Institute, 4th Military Medical University, Xi'an; , Sun Yat-Sen University Cancer Center, Guangzhou; , 3rd Affiliated Hospital of Harbin Medical University, Harbin, Heilongjiang; and , The People's Liberation Army Cancer Centre, Nanjing Bayi Hospital, Nanjing, Jiangsu, People's Republic of China.; (Huang YG) , Huazhong University of Science and Technology and Tongji Hospital, Wuhan; , Shanghai Changzheng Hospital, Shanghai; , Peking Union Medical College Hospital; , Beijing University Cancer Hospital, Beijing; , Anhui Provincial Hospital, Anhui; , Tianjin Medical University Cancer Institute and Hospital, National Clinical Research Center for Cancer, Tianjin; , Guangdong Lung Cancer Institute, Guangdong General Hospital, Guangdong; , Tangdu Hospital and Tangdu Comprehensive Cancer Center, Cancer Institute, 4th Military Medical University, Xi'an; , Sun Yat-Sen University Cancer Center, Guangzhou; , 3rd Affiliated Hospital of Harbin Medical University, Harbin, Heilongjiang; and , The People's Liberation Army Cancer Centre, Nanjing Bayi Hospital, Nanjing, Jiangsu, People's Republic of China.; (Hu B) , Huazhong University of Science and Technology and Tongji Hospital, Wuhan; , Shanghai Changzheng Hospital, Shanghai; , Peking Union Medical College Hospital; , Beijing University Cancer Hospital, Beijing; , Anhui Provincial Hospital, Anhui; , Tianjin Medical University Cancer Institute and Hospital, National Clinical Research Center for Cancer, Tianjin; , Guangdong Lung Cancer Institute, Guangdong General Hospital, Guangdong; , Tangdu Hospital and Tangdu Comprehensive Cancer Center, Cancer Institute, 4th Military Medical University, Xi'an; , Sun Yat-Sen University Cancer Center, Guangzhou; , 3rd Affiliated Hospital of Harbin Medical University, Harbin, Heilongjiang; and , The People's Liberation Army Cancer Centre, Nanjing Bayi Hospital, Nanjing, Jiangsu, People's Republic of China.; (Wang K) , Huazhong University of Science and Technology and Tongji Hospital, Wuhan; , Shanghai Changzheng Hospital, Shanghai; , Peking Union Medical College Hospital; , Beijing University Cancer Hospital, Beijing; , Anhui Provincial Hospital, Anhui; , Tianjin Medical University Cancer Institute and Hospital, National Clinical Research Center for Cancer, Tianjin; , Guangdong Lung Cancer Institute, Guangdong General Hospital, Guangdong; , Tangdu Hospital and Tangdu Comprehensive Cancer Center, Cancer Institute, 4th Military Medical University, Xi'an; , Sun Yat-Sen University Cancer Center, Guangzhou; , 3rd Affiliated Hospital of

Harbin Medical University, Harbin, Heilongjiang; and , The People's Liberation Army Cancer Centre, Nanjing Bayi Hospital, Nanjing, Jiangsu, People's Republic of China.; (Li PP) , Huazhong University of Science and Technology and Tongji Hospital, Wuhan; , Shanghai Changzheng Hospital, Shanghai; , Peking Union Medical College Hospital; , Beijing University Cancer Hospital, Beijing; , Anhui Provincial Hospital, Anhui; , Tianjin Medical University Cancer Institute and Hospital, National Clinical Research Center for Cancer, Tianjin; , Guangdong Lung Cancer Institute, Guangdong General Hospital, Guangdong; , Tangdu Hospital and Tangdu Comprehensive Cancer Center, Cancer Institute, 4th Military Medical University, Xi'an; , Sun Yat-Sen University Cancer Center, Guangzhou; , 3rd Affiliated Hospital of Harbin Medical University, Harbin, Heilongjiang; and , The People's Liberation Army Cancer Centre, Nanjing Bayi Hospital, Nanjing, Jiangsu, People's Republic of China.; (Wu YL) , Huazhong University of Science and Technology and Tongji Hospital, Wuhan; , Shanghai Changzheng Hospital, Shanghai; , Peking Union Medical College Hospital; , Beijing University Cancer Hospital, Beijing; , Anhui Provincial Hospital, Anhui; , Tianjin Medical University Cancer Institute and Hospital, National Clinical Research Center for Cancer, Tianjin; , Guangdong Lung Cancer Institute, Guangdong General Hospital, Guangdong; , Tangdu Hospital and Tangdu Comprehensive Cancer Center, Cancer Institute, 4th Military Medical University, Xi'an; , Sun Yat-Sen University Cancer Center, Guangzhou; , 3rd Affiliated Hospital of Harbin Medical University, Harbin, Heilongjiang; and , The People's Liberation Army Cancer Centre, Nanjing Bayi Hospital, Nanjing, Jiangsu, People's Republic of China.; (Zhang HL) , Huazhong University of Science and Technology and Tongji Hospital, Wuhan; , Shanghai Changzheng Hospital, Shanghai; , Peking Union Medical College Hospital; , Beijing University Cancer Hospital, Beijing; , Anhui Provincial Hospital, Anhui; , Tianjin Medical University Cancer Institute and Hospital, National Clinical Research Center for Cancer, Tianjin; , Guangdong Lung Cancer Institute, Guangdong General Hospital, Guangdong; , Tangdu Hospital and Tangdu Comprehensive Cancer Center, Cancer Institute, 4th Military Medical University, Xi'an; , Sun Yat-Sen University Cancer Center, Guangzhou; , 3rd Affiliated Hospital of Harbin Medical University, Harbin, Heilongjiang; and , The People's Liberation Army Cancer Centre, Nanjing Bayi Hospital, Nanjing, Jiangsu, People's Republic of China.; (Zhang L) , Huazhong University of Science and Technology and Tongji Hospital, Wuhan; , Shanghai Changzheng Hospital, Shanghai; , Peking Union Medical College Hospital; , Beijing University Cancer Hospital, Beijing; , Anhui Provincial Hospital, Anhui; , Tianjin Medical University Cancer Institute and Hospital, National Clinical Research Center for Cancer, Tianjin; , Guangdong Lung Cancer Institute, Guangdong General Hospital, Guangdong; , Tangdu Hospital and Tangdu Comprehensive Cancer Center, Cancer Institute, 4th Military Medical University, Xi'an; , Sun Yat-Sen University Cancer Center, Guangzhou; , 3rd Affiliated Hospital of Harbin Medical University, Harbin, Heilongjiang; and , The People's Liberation Army Cancer Centre, Nanjing Bayi Hospital, Nanjing, Jiangsu, People's Republic of China.; (Zhang QY) , Huazhong University of Science and Technology and Tongji Hospital, Wuhan; , Shanghai Changzheng Hospital, Shanghai; , Peking Union Medical College Hospital; , Beijing University Cancer Hospital, Beijing; , Anhui Provincial Hospital, Anhui; , Tianjin Medical University Cancer Institute and Hospital, National Clinical Research Center for Cancer, Tianjin; , Guangdong Lung Cancer Institute, Guangdong General Hospital, Guangdong; , Tangdu Hospital and Tangdu Comprehensive Cancer Center, Cancer Institute, 4th Military Medical University, Xi'an; , Sun Yat-Sen University Cancer Center, Guangzhou; , 3rd Affiliated Hospital of

Harbin Medical University, Harbin, Heilongjiang; and , The People's Liberation Army Cancer Centre, Nanjing Bayi Hospital, Nanjing, Jiangsu, People's Republic of China.; (Qin SK) , Huazhong University of Science and Technology and Tongji Hospital, Wuhan; , Shanghai Changzheng Hospital, Shanghai; , Peking Union Medical College Hospital; , Beijing University Cancer Hospital, Beijing; , Anhui Provincial Hospital, Anhui; , Tianjin Medical University Cancer Institute and Hospital, National Clinical Research Center for Cancer, Tianjin; , Guangdong Lung Cancer Institute, Guangdong General Hospital, Guangdong; , Tangdu Hospital and Tangdu Comprehensive Cancer Center, Cancer Institute, 4th Military Medical University, Xi'an; , Sun Yat-Sen University Cancer Center, Guangzhou; , 3rd Affiliated Hospital of Harbin Medical University, Harbin, Heilongjiang; and , The People's Liberation Army Cancer Centre, Nanjing Bayi Hospital, Nanjing, Jiangsu, People's Republic of China. (Yu SY) , Huazhong University of Science and Technology and Tongji Hospital, Wuhan; , Shanghai Changzheng Hospital, Shanghai; , Peking Union Medical College Hospital; , Beijing University Cancer Hospital, Beijing; , Anhui Provincial Hospital, Anhui; , Tianjin Medical University Cancer Institute and Hospital, National Clinical Research Center for Cancer, Tianjin; , Guangdong Lung Cancer Institute, Guangdong General Hospital, Guangdong; , Tangdu Hospital and Tangdu Comprehensive Cancer Center, Cancer Institute, 4th Military Medical University, Xi'an; , Sun Yat-Sen University Cancer Center, Guangzhou; , 3rd Affiliated Hospital of Harbin Medical University, Harbin, Heilongjiang; and , The People's Liberation Army Cancer Centre, Nanjing Bayi Hospital, Nanjing, Jiangsu, People's Republic of China.; (Wang JJ) , Huazhong University of Science and Technology and Tongji Hospital, Wuhan; , Shanghai Changzheng Hospital, Shanghai; , Peking Union Medical College Hospital; , Beijing University Cancer Hospital, Beijing; , Anhui Provincial Hospital, Anhui; , Tianjin Medical University Cancer Institute and Hospital, National Clinical Research Center for Cancer, Tianjin; , Guangdong Lung Cancer Institute, Guangdong General Hospital, Guangdong; , Tangdu Hospital and Tangdu Comprehensive Cancer Center, Cancer Institute, 4th Military Medical University, Xi'an; , Sun Yat-Sen University Cancer Center, Guangzhou; , 3rd Affiliated Hospital of Harbin Medical University, Harbin, Heilongjiang; and , The People's Liberation Army Cancer Centre, Nanjing Bayi Hospital, Nanjing, Jiangsu, People's Republic of China.; (Huang YG) , Huazhong University of Science and Technology and Tongji Hospital, Wuhan; , Shanghai Changzheng Hospital, Shanghai; , Peking Union Medical College Hospital; , Beijing University Cancer Hospital, Beijing; , Anhui Provincial Hospital, Anhui; , Tianjin Medical University Cancer Institute and Hospital, National Clinical Research Center for Cancer, Tianjin; , Guangdong Lung Cancer Institute, Guangdong General Hospital, Guangdong; , Tangdu Hospital and Tangdu Comprehensive Cancer Center, Cancer Institute, 4th Military Medical University, Xi'an; , Sun Yat-Sen University Cancer Center, Guangzhou; , 3rd Affiliated Hospital of Harbin Medical University, Harbin, Heilongjiang; and , The People's Liberation Army Cancer Centre, Nanjing Bayi Hospital, Nanjing, Jiangsu, People's Republic of China.; (Hu B) , Huazhong University of Science and Technology and Tongji Hospital, Wuhan; , Shanghai Changzheng Hospital, Shanghai; , Peking Union Medical College Hospital; , Beijing University Cancer Hospital, Beijing; , Anhui Provincial Hospital, Anhui; , Tianjin Medical University Cancer Institute and Hospital, National Clinical Research Center for Cancer, Tianjin; , Guangdong Lung Cancer Institute, Guangdong General Hospital, Guangdong; , Tangdu Hospital and Tangdu Comprehensive Cancer Center, Cancer Institute, 4th Military Medical University, Xi'an; , Sun Yat-Sen University Cancer Center, Guangzhou; , 3rd Affiliated Hospital of

Harbin Medical University, Harbin, Heilongjiang; and , The People's Liberation Army Cancer Centre, Nanjing Bayi Hospital, Nanjing, Jiangsu, People's Republic of China.; (Wang K) , Huazhong University of Science and Technology and Tongji Hospital, Wuhan; , Shanghai Changzheng Hospital, Shanghai; , Peking Union Medical College Hospital; , Beijing University Cancer Hospital, Beijing; , Anhui Provincial Hospital, Anhui; , Tianjin Medical University Cancer Institute and Hospital, National Clinical Research Center for Cancer, Tianjin; , Guangdong Lung Cancer Institute, Guangdong General Hospital, Guangdong; , Tangdu Hospital and Tangdu Comprehensive Cancer Center, Cancer Institute, 4th Military Medical University, Xi'an; , Sun Yat-Sen University Cancer Center, Guangzhou; , 3rd Affiliated Hospital of Harbin Medical University, Harbin, Heilongjiang; and , The People's Liberation Army Cancer Centre, Nanjing Bayi Hospital, Nanjing, Jiangsu, People's Republic of China.; (Li PP) , Huazhong University of Science and Technology and Tongji Hospital, Wuhan; , Shanghai Changzheng Hospital, Shanghai; , Peking Union Medical College Hospital; , Beijing University Cancer Hospital, Beijing; , Anhui Provincial Hospital, Anhui; , Tianjin Medical University Cancer Institute and Hospital, National Clinical Research Center for Cancer, Tianjin; , Guangdong Lung Cancer Institute, Guangdong General Hospital, Guangdong; , Tangdu Hospital and Tangdu Comprehensive Cancer Center, Cancer Institute, 4th Military Medical University, Xi'an; , Sun Yat-Sen University Cancer Center, Guangzhou; , 3rd Affiliated Hospital of Harbin Medical University, Harbin, Heilongjiang; and , The People's Liberation Army Cancer Centre, Nanjing Bayi Hospital, Nanjing, Jiangsu, People's Republic of China.; (Wu YL) , Huazhong University of Science and Technology and Tongji Hospital, Wuhan; , Shanghai Changzheng Hospital, Shanghai; , Peking Union Medical College Hospital; , Beijing University Cancer Hospital, Beijing; , Anhui Provincial Hospital, Anhui; , Tianjin Medical University Cancer Institute and Hospital, National Clinical Research Center for Cancer, Tianjin; , Guangdong Lung Cancer Institute, Guangdong General Hospital, Guangdong; , Tangdu Hospital and Tangdu Comprehensive Cancer Center, Cancer Institute, 4th Military Medical University, Xi'an; , Sun Yat-Sen University Cancer Center, Guangzhou; , 3rd Affiliated Hospital of Harbin Medical University, Harbin, Heilongjiang; and , The People's Liberation Army Cancer Centre, Nanjing Bayi Hospital, Nanjing, Jiangsu, People's Republic of China.; (Zhang HL) , Huazhong University of Science and Technology and Tongji Hospital, Wuhan; , Shanghai Changzheng Hospital, Shanghai; , Peking Union Medical College Hospital; , Beijing University Cancer Hospital, Beijing; , Anhui Provincial Hospital, Anhui; , Tianjin Medical University Cancer Institute and Hospital, National Clinical Research Center for Cancer, Tianjin; , Guangdong Lung Cancer Institute, Guangdong General Hospital, Guangdong; , Tangdu Hospital and Tangdu Comprehensive Cancer Center, Cancer Institute, 4th Military Medical University, Xi'an; , Sun Yat-Sen University Cancer Center, Guangzhou; , 3rd Affiliated Hospital of Harbin Medical University, Harbin, Heilongjiang; and , The People's Liberation Army Cancer Centre, Nanjing Bayi Hospital, Nanjing, Jiangsu, People's Republic of China.; (Zhang L) , Huazhong University of Science and Technology and Tongji Hospital, Wuhan; , Shanghai Changzheng Hospital, Shanghai; , Peking Union Medical College Hospital; , Beijing University Cancer Hospital, Beijing; , Anhui Provincial Hospital, Anhui; , Tianjin Medical University Cancer Institute and Hospital, National Clinical Research Center for Cancer, Tianjin; , Guangdong Lung Cancer Institute, Guangdong General Hospital, Guangdong; , Tangdu Hospital and Tangdu Comprehensive Cancer Center, Cancer Institute, 4th Military Medical University, Xi'an; , Sun Yat-Sen University Cancer Center, Guangzhou; , 3rd Affiliated Hospital of

Harbin Medical University, Harbin, Heilongjiang; and , The People's Liberation Army Cancer Centre, Nanjing Bayi Hospital, Nanjing, Jiangsu, People's Republic of China.; (Zhang QY) , Huazhong University of Science and Technology and Tongji Hospital, Wuhan; , Shanghai Changzheng Hospital, Shanghai; , Peking Union Medical College Hospital; , Beijing University Cancer Hospital, Beijing; , Anhui Provincial Hospital, Anhui; , Tianjin Medical University Cancer Institute and Hospital, National Clinical Research Center for Cancer, Tianjin; , Guangdong Lung Cancer Institute, Guangdong General Hospital, Guangdong; , Tangdu Hospital and Tangdu Comprehensive Cancer Center, Cancer Institute, 4th Military Medical University, Xi'an; , Sun Yat-Sen University Cancer Center, Guangzhou; , 3rd Affiliated Hospital of Harbin Medical University, Harbin, Heilongjiang; and , The People's Liberation Army Cancer Centre, Nanjing Bayi Hospital, Nanjing, Jiangsu, People's Republic of China.; (Qin SK) , Huazhong University of Science and Technology and Tongji Hospital, Wuhan; , Shanghai Changzheng Hospital, Shanghai; , Peking Union Medical College Hospital; , Beijing University Cancer Hospital, Beijing; , Anhui Provincial Hospital, Anhui; , Tianjin Medical University Cancer Institute and Hospital, National Clinical Research Center for Cancer, Tianjin; , Guangdong Lung Cancer Institute, Guangdong General Hospital, Guangdong; , Tangdu Hospital and Tangdu Comprehensive Cancer Center, Cancer Institute, 4th Military Medical University, Xi'an; , Sun Yat-Sen University Cancer Center, Guangzhou; , 3rd Affiliated Hospital of Harbin Medical University, Harbin, Heilongjiang; and , The People's Liberation Army Cancer Centre, Nanjing Bayi Hospital, Nanjing, Jiangsu, People's Republic of China. (Yu SY) , Huazhong University of Science and Technology and Tongji Hospital, Wuhan; , Shanghai Changzheng Hospital, Shanghai; , Peking Union Medical College Hospital; , Beijing University Cancer Hospital, Beijing; , Anhui Provincial Hospital, Anhui; , Tianjin Medical University Cancer Institute and Hospital, National Clinical Research Center for Cancer, Tianjin; , Guangdong Lung Cancer Institute, Guangdong General Hospital, Guangdong; , Tangdu Hospital and Tangdu Comprehensive Cancer Center, Cancer Institute, 4th Military Medical University, Xi'an; , Sun Yat-Sen University Cancer Center, Guangzhou; , 3rd Affiliated Hospital of Harbin Medical University, Harbin, Heilongjiang; and , The People's Liberation Army Cancer Centre, Nanjing Bayi Hospital, Nanjing, Jiangsu, People's Republic of China.; (Wang JJ) , Huazhong University of Science and Technology and Tongji Hospital, Wuhan; , Shanghai Changzheng Hospital, Shanghai; , Peking Union Medical College Hospital; , Beijing University Cancer Hospital, Beijing; , Anhui Provincial Hospital, Anhui; , Tianjin Medical University Cancer Institute and Hospital, National Clinical Research Center for Cancer, Tianjin; , Guangdong Lung Cancer Institute, Guangdong General Hospital, Guangdong; , Tangdu Hospital and Tangdu Comprehensive Cancer Center, Cancer Institute, 4th Military Medical University, Xi'an; , Sun Yat-Sen University Cancer Center, Guangzhou; , 3rd Affiliated Hospital of Harbin Medical University, Harbin, Heilongjiang; and , The People's Liberation Army Cancer Centre, Nanjing Bayi Hospital, Nanjing, Jiangsu, People's Republic of China.; (Huang YG) , Huazhong University of Science and Technology and Tongji Hospital, Wuhan; , Shanghai Changzheng Hospital, Shanghai; , Peking Union Medical College Hospital; , Beijing University Cancer Hospital, Beijing; , Anhui Provincial Hospital, Anhui; , Tianjin Medical University Cancer Institute and Hospital, National Clinical Research Center for Cancer, Tianjin; , Guangdong Lung Cancer Institute, Guangdong General Hospital, Guangdong; , Tangdu Hospital and Tangdu Comprehensive Cancer Center, Cancer Institute, 4th Military Medical University, Xi'an; , Sun Yat-Sen University Cancer Center, Guangzhou; , 3rd Affiliated Hospital of

Harbin Medical University, Harbin, Heilongjiang; and , The People's Liberation Army Cancer Centre, Nanjing Bayi Hospital, Nanjing, Jiangsu, People's Republic of China.; (Hu B) , Huazhong University of Science and Technology and Tongji Hospital, Wuhan; , Shanghai Changzheng Hospital, Shanghai; , Peking Union Medical College Hospital; , Beijing University Cancer Hospital, Beijing; , Anhui Provincial Hospital, Anhui; , Tianjin Medical University Cancer Institute and Hospital, National Clinical Research Center for Cancer, Tianjin; , Guangdong Lung Cancer Institute, Guangdong General Hospital, Guangdong; , Tangdu Hospital and Tangdu Comprehensive Cancer Center, Cancer Institute, 4th Military Medical University, Xi'an; , Sun Yat-Sen University Cancer Center, Guangzhou; , 3rd Affiliated Hospital of Harbin Medical University, Harbin, Heilongjiang; and , The People's Liberation Army Cancer Centre, Nanjing Bayi Hospital, Nanjing, Jiangsu, People's Republic of China.; (Wang K) , Huazhong University of Science and Technology and Tongji Hospital, Wuhan; , Shanghai Changzheng Hospital, Shanghai; , Peking Union Medical College Hospital; , Beijing University Cancer Hospital, Beijing; , Anhui Provincial Hospital, Anhui; , Tianjin Medical University Cancer Institute and Hospital, National Clinical Research Center for Cancer, Tianjin; , Guangdong Lung Cancer Institute, Guangdong General Hospital, Guangdong; , Tangdu Hospital and Tangdu Comprehensive Cancer Center, Cancer Institute, 4th Military Medical University, Xi'an; , Sun Yat-Sen University Cancer Center, Guangzhou; , 3rd Affiliated Hospital of Harbin Medical University, Harbin, Heilongjiang; and , The People's Liberation Army Cancer Centre, Nanjing Bayi Hospital, Nanjing, Jiangsu, People's Republic of China.; (Li PP) , Huazhong University of Science and Technology and Tongji Hospital, Wuhan; , Shanghai Changzheng Hospital, Shanghai; , Peking Union Medical College Hospital; , Beijing University Cancer Hospital, Beijing; , Anhui Provincial Hospital, Anhui; , Tianjin Medical University Cancer Institute and Hospital, National Clinical Research Center for Cancer, Tianjin; , Guangdong Lung Cancer Institute, Guangdong General Hospital, Guangdong; , Tangdu Hospital and Tangdu Comprehensive Cancer Center, Cancer Institute, 4th Military Medical University, Xi'an; , Sun Yat-Sen University Cancer Center, Guangzhou; , 3rd Affiliated Hospital of Harbin Medical University, Harbin, Heilongjiang; and , The People's Liberation Army Cancer Centre, Nanjing Bayi Hospital, Nanjing, Jiangsu, People's Republic of China.; (Wu YL) , Huazhong University of Science and Technology and Tongji Hospital, Wuhan; , Shanghai Changzheng Hospital, Shanghai; , Peking Union Medical College Hospital; , Beijing University Cancer Hospital, Beijing; , Anhui Provincial Hospital, Anhui; , Tianjin Medical University Cancer Institute and Hospital, National Clinical Research Center for Cancer, Tianjin; , Guangdong Lung Cancer Institute, Guangdong General Hospital, Guangdong; , Tangdu Hospital and Tangdu Comprehensive Cancer Center, Cancer Institute, 4th Military Medical University, Xi'an; , Sun Yat-Sen University Cancer Center, Guangzhou; , 3rd Affiliated Hospital of Harbin Medical University, Harbin, Heilongjiang; and , The People's Liberation Army Cancer Centre, Nanjing Bayi Hospital, Nanjing, Jiangsu, People's Republic of China.; (Zhang HL) , Huazhong University of Science and Technology and Tongji Hospital, Wuhan; , Shanghai Changzheng Hospital, Shanghai; , Peking Union Medical College Hospital; , Beijing University Cancer Hospital, Beijing; , Anhui Provincial Hospital, Anhui; , Tianjin Medical University Cancer Institute and Hospital, National Clinical Research Center for Cancer, Tianjin; , Guangdong Lung Cancer Institute, Guangdong General Hospital, Guangdong; , Tangdu Hospital and Tangdu Comprehensive Cancer Center, Cancer Institute, 4th Military Medical University, Xi'an; , Sun Yat-Sen University Cancer Center, Guangzhou; , 3rd Affiliated Hospital of

Harbin Medical University, Harbin, Heilongjiang; and , The People's Liberation Army Cancer Centre, Nanjing Bayi Hospital, Nanjing, Jiangsu, People's Republic of China.; (Zhang L) , Huazhong University of Science and Technology and Tongji Hospital, Wuhan; , Shanghai Changzheng Hospital, Shanghai; , Peking Union Medical College Hospital; , Beijing University Cancer Hospital, Beijing; , Anhui Provincial Hospital, Anhui; , Tianjin Medical University Cancer Institute and Hospital, National Clinical Research Center for Cancer, Tianjin; , Guangdong Lung Cancer Institute, Guangdong General Hospital, Guangdong; , Tangdu Hospital and Tangdu Comprehensive Cancer Center, Cancer Institute, 4th Military Medical University, Xi'an; , Sun Yat-Sen University Cancer Center, Guangzhou; , 3rd Affiliated Hospital of Harbin Medical University, Harbin, Heilongjiang; and , The People's Liberation Army Cancer Centre, Nanjing Bayi Hospital, Nanjing, Jiangsu, People's Republic of China.; (Zhang QY) , Huazhong University of Science and Technology and Tongji Hospital, Wuhan; , Shanghai Changzheng Hospital, Shanghai; , Peking Union Medical College Hospital; , Beijing University Cancer Hospital, Beijing; , Anhui Provincial Hospital, Anhui; , Tianjin Medical University Cancer Institute and Hospital, National Clinical Research Center for Cancer, Tianjin; , Guangdong Lung Cancer Institute, Guangdong General Hospital, Guangdong; , Tangdu Hospital and Tangdu Comprehensive Cancer Center, Cancer Institute, 4th Military Medical University, Xi'an; , Sun Yat-Sen University Cancer Center, Guangzhou; , 3rd Affiliated Hospital of Harbin Medical University, Harbin, Heilongjiang; and , The People's Liberation Army Cancer Centre, Nanjing Bayi Hospital, Nanjing, Jiangsu, People's Republic of China.; (Qin SK) , Huazhong University of Science and Technology and Tongji Hospital, Wuhan; , Shanghai Changzheng Hospital, Shanghai; , Peking Union Medical College Hospital; , Beijing University Cancer Hospital, Beijing; , Anhui Provincial Hospital, Anhui; , Tianjin Medical University Cancer Institute and Hospital, National Clinical Research Center for Cancer, Tianjin; , Guangdong Lung Cancer Institute, Guangdong General Hospital, Guangdong; , Tangdu Hospital and Tangdu Comprehensive Cancer Center, Cancer Institute, 4th Military Medical University, Xi'an; , Sun Yat-Sen University Cancer Center, Guangzhou; , 3rd Affiliated Hospital of Harbin Medical University, Harbin, Heilongjiang; and , The People's Liberation Army Cancer Centre, Nanjing Bayi Hospital, Nanjing, Jiangsu, People's Republic of China. (Yu SY) , Huazhong University of Science and Technology and Tongji Hospital, Wuhan; , Shanghai Changzheng Hospital, Shanghai; , Peking Union Medical College Hospital; , Beijing University Cancer Hospital, Beijing; , Anhui Provincial Hospital, Anhui; , Tianjin Medical University Cancer Institute and Hospital, National Clinical Research Center for Cancer, Tianjin; , Guangdong Lung Cancer Institute, Guangdong General Hospital, Guangdong; , Tangdu Hospital and Tangdu Comprehensive Cancer Center, Cancer Institute, 4th Military Medical University, Xi'an; , Sun Yat-Sen University Cancer Center, Guangzhou; , 3rd Affiliated Hospital of Harbin Medical University, Harbin, Heilongjiang; and , The People's Liberation Army Cancer Centre, Nanjing Bayi Hospital, Nanjing, Jiangsu, People's Republic of China.; (Wang JJ) , Huazhong University of Science and Technology and Tongji Hospital, Wuhan; , Shanghai Changzheng Hospital, Shanghai; , Peking Union Medical College Hospital; , Beijing University Cancer Hospital, Beijing; , Anhui Provincial Hospital, Anhui; , Tianjin Medical University Cancer Institute and Hospital, National Clinical Research Center for Cancer, Tianjin; , Guangdong Lung Cancer Institute, Guangdong General Hospital, Guangdong; , Tangdu Hospital and Tangdu Comprehensive Cancer Center, Cancer Institute, 4th Military Medical University, Xi'an; , Sun Yat-Sen University Cancer Center, Guangzhou; , 3rd Affiliated Hospital of

Harbin Medical University, Harbin, Heilongjiang; and , The People's Liberation Army Cancer Centre, Nanjing Bayi Hospital, Nanjing, Jiangsu, People's Republic of China.; (Huang YG) , Huazhong University of Science and Technology and Tongji Hospital, Wuhan; , Shanghai Changzheng Hospital, Shanghai; , Peking Union Medical College Hospital; , Beijing University Cancer Hospital, Beijing; , Anhui Provincial Hospital, Anhui; , Tianjin Medical University Cancer Institute and Hospital, National Clinical Research Center for Cancer, Tianjin; , Guangdong Lung Cancer Institute, Guangdong General Hospital, Guangdong; , Tangdu Hospital and Tangdu Comprehensive Cancer Center, Cancer Institute, 4th Military Medical University, Xi'an; , Sun Yat-Sen University Cancer Center, Guangzhou; , 3rd Affiliated Hospital of Harbin Medical University, Harbin, Heilongjiang; and , The People's Liberation Army Cancer Centre, Nanjing Bayi Hospital, Nanjing, Jiangsu, People's Republic of China.; (Hu B) , Huazhong University of Science and Technology and Tongji Hospital, Wuhan; , Shanghai Changzheng Hospital, Shanghai; , Peking Union Medical College Hospital; , Beijing University Cancer Hospital, Beijing; , Anhui Provincial Hospital, Anhui; , Tianjin Medical University Cancer Institute and Hospital, National Clinical Research Center for Cancer, Tianjin; , Guangdong Lung Cancer Institute, Guangdong General Hospital, Guangdong; , Tangdu Hospital and Tangdu Comprehensive Cancer Center, Cancer Institute, 4th Military Medical University, Xi'an; , Sun Yat-Sen University Cancer Center, Guangzhou; , 3rd Affiliated Hospital of Harbin Medical University, Harbin, Heilongjiang; and , The People's Liberation Army Cancer Centre, Nanjing Bayi Hospital, Nanjing, Jiangsu, People's Republic of China.; (Wang K) , Huazhong University of Science and Technology and Tongji Hospital, Wuhan; , Shanghai Changzheng Hospital, Shanghai; , Peking Union Medical College Hospital; , Beijing University Cancer Hospital, Beijing; , Anhui Provincial Hospital, Anhui; , Tianjin Medical University Cancer Institute and Hospital, National Clinical Research Center for Cancer, Tianjin; , Guangdong Lung Cancer Institute, Guangdong General Hospital, Guangdong; , Tangdu Hospital and Tangdu Comprehensive Cancer Center, Cancer Institute, 4th Military Medical University, Xi'an; , Sun Yat-Sen University Cancer Center, Guangzhou; , 3rd Affiliated Hospital of Harbin Medical University, Harbin, Heilongjiang; and , The People's Liberation Army Cancer Centre, Nanjing Bayi Hospital, Nanjing, Jiangsu, People's Republic of China.; (Li PP) , Huazhong University of Science and Technology and Tongji Hospital, Wuhan; , Shanghai Changzheng Hospital, Shanghai; , Peking Union Medical College Hospital; , Beijing University Cancer Hospital, Beijing; , Anhui Provincial Hospital, Anhui; , Tianjin Medical University Cancer Institute and Hospital, National Clinical Research Center for Cancer, Tianjin; , Guangdong Lung Cancer Institute, Guangdong General Hospital, Guangdong; , Tangdu Hospital and Tangdu Comprehensive Cancer Center, Cancer Institute, 4th Military Medical University, Xi'an; , Sun Yat-Sen University Cancer Center, Guangzhou; , 3rd Affiliated Hospital of Harbin Medical University, Harbin, Heilongjiang; and , The People's Liberation Army Cancer Centre, Nanjing Bayi Hospital, Nanjing, Jiangsu, People's Republic of China.; (Wu YL) , Huazhong University of Science and Technology and Tongji Hospital, Wuhan; , Shanghai Changzheng Hospital, Shanghai; , Peking Union Medical College Hospital; , Beijing University Cancer Hospital, Beijing; , Anhui Provincial Hospital, Anhui; , Tianjin Medical University Cancer Institute and Hospital, National Clinical Research Center for Cancer, Tianjin; , Guangdong Lung Cancer Institute, Guangdong General Hospital, Guangdong; , Tangdu Hospital and Tangdu Comprehensive Cancer Center, Cancer Institute, 4th Military Medical University, Xi'an; , Sun Yat-Sen University Cancer Center, Guangzhou; , 3rd Affiliated Hospital of

Harbin Medical University, Harbin, Heilongjiang; and , The People's Liberation Army Cancer Centre, Nanjing Bayi Hospital, Nanjing, Jiangsu, People's Republic of China.; (Zhang HL) , Huazhong University of Science and Technology and Tongji Hospital, Wuhan; , Shanghai Changzheng Hospital, Shanghai; , Peking Union Medical College Hospital; , Beijing University Cancer Hospital, Beijing; , Anhui Provincial Hospital, Anhui; , Tianjin Medical University Cancer Institute and Hospital, National Clinical Research Center for Cancer, Tianjin; , Guangdong Lung Cancer Institute, Guangdong General Hospital, Guangdong; , Tangdu Hospital and Tangdu Comprehensive Cancer Center, Cancer Institute, 4th Military Medical University, Xi'an; , Sun Yat-Sen University Cancer Center, Guangzhou; , 3rd Affiliated Hospital of Harbin Medical University, Harbin, Heilongjiang; and , The People's Liberation Army Cancer Centre, Nanjing Bayi Hospital, Nanjing, Jiangsu, People's Republic of China.; (Zhang L) , Huazhong University of Science and Technology and Tongji Hospital, Wuhan; , Shanghai Changzheng Hospital, Shanghai; , Peking Union Medical College Hospital; , Beijing University Cancer Hospital, Beijing; , Anhui Provincial Hospital, Anhui; , Tianjin Medical University Cancer Institute and Hospital, National Clinical Research Center for Cancer, Tianjin; , Guangdong Lung Cancer Institute, Guangdong General Hospital, Guangdong; , Tangdu Hospital and Tangdu Comprehensive Cancer Center, Cancer Institute, 4th Military Medical University, Xi'an; , Sun Yat-Sen University Cancer Center, Guangzhou; , 3rd Affiliated Hospital of Harbin Medical University, Harbin, Heilongjiang; and , The People's Liberation Army Cancer Centre, Nanjing Bayi Hospital, Nanjing, Jiangsu, People's Republic of China.; (Zhang QY) , Huazhong University of Science and Technology and Tongji Hospital, Wuhan; , Shanghai Changzheng Hospital, Shanghai; , Peking Union Medical College Hospital; , Beijing University Cancer Hospital, Beijing; , Anhui Provincial Hospital, Anhui; , Tianjin Medical University Cancer Institute and Hospital, National Clinical Research Center for Cancer, Tianjin; , Guangdong Lung Cancer Institute, Guangdong General Hospital, Guangdong; , Tangdu Hospital and Tangdu Comprehensive Cancer Center, Cancer Institute, 4th Military Medical University, Xi'an; , Sun Yat-Sen University Cancer Center, Guangzhou; , 3rd Affiliated Hospital of Harbin Medical University, Harbin, Heilongjiang; and , The People's Liberation Army Cancer Centre, Nanjing Bayi Hospital, Nanjing, Jiangsu, People's Republic of China.; (Qin SK) , Huazhong University of Science and Technology and Tongji Hospital, Wuhan; , Shanghai Changzheng Hospital, Shanghai; , Peking Union Medical College Hospital; , Beijing University Cancer Hospital, Beijing; , Anhui Provincial Hospital, Anhui; , Tianjin Medical University Cancer Institute and Hospital, National Clinical Research Center for Cancer, Tianjin; , Guangdong Lung Cancer Institute, Guangdong General Hospital, Guangdong; , Tangdu Hospital and Tangdu Comprehensive Cancer Center, Cancer Institute, 4th Military Medical University, Xi'an; , Sun Yat-Sen University Cancer Center, Guangzhou; , 3rd Affiliated Hospital of Harbin Medical University, Harbin, Heilongjiang; and , The People's Liberation Army Cancer Centre, Nanjing Bayi Hospital, Nanjing, Jiangsu, People's Republic of China. (Yu SY) , Huazhong University of Science and Technology and Tongji Hospital, Wuhan; , Shanghai Changzheng Hospital, Shanghai; , Peking Union Medical College Hospital; , Beijing University Cancer Hospital, Beijing; , Anhui Provincial Hospital, Anhui; , Tianjin Medical University Cancer Institute and Hospital, National Clinical Research Center for Cancer, Tianjin; , Guangdong Lung Cancer Institute, Guangdong General Hospital, Guangdong; , Tangdu Hospital and Tangdu Comprehensive Cancer Center, Cancer Institute, 4th Military Medical University, Xi'an; , Sun Yat-Sen University Cancer Center, Guangzhou; , 3rd Affiliated Hospital of

Harbin Medical University, Harbin, Heilongjiang; and , The People's Liberation Army Cancer Centre, Nanjing Bayi Hospital, Nanjing, Jiangsu, People's Republic of China.; (Wang JJ) , Huazhong University of Science and Technology and Tongji Hospital, Wuhan; , Shanghai Changzheng Hospital, Shanghai; , Peking Union Medical College Hospital; , Beijing University Cancer Hospital, Beijing; , Anhui Provincial Hospital, Anhui; , Tianjin Medical University Cancer Institute and Hospital, National Clinical Research Center for Cancer, Tianjin; , Guangdong Lung Cancer Institute, Guangdong General Hospital, Guangdong; , Tangdu Hospital and Tangdu Comprehensive Cancer Center, Cancer Institute, 4th Military Medical University, Xi'an; , Sun Yat-Sen University Cancer Center, Guangzhou; , 3rd Affiliated Hospital of Harbin Medical University, Harbin, Heilongjiang; and , The People's Liberation Army Cancer Centre, Nanjing Bayi Hospital, Nanjing, Jiangsu, People's Republic of China.; (Huang YG) , Huazhong University of Science and Technology and Tongji Hospital, Wuhan; , Shanghai Changzheng Hospital, Shanghai; , Peking Union Medical College Hospital; , Beijing University Cancer Hospital, Beijing; , Anhui Provincial Hospital, Anhui; , Tianjin Medical University Cancer Institute and Hospital, National Clinical Research Center for Cancer, Tianjin; , Guangdong Lung Cancer Institute, Guangdong General Hospital, Guangdong; , Tangdu Hospital and Tangdu Comprehensive Cancer Center, Cancer Institute, 4th Military Medical University, Xi'an; , Sun Yat-Sen University Cancer Center, Guangzhou; , 3rd Affiliated Hospital of Harbin Medical University, Harbin, Heilongjiang; and , The People's Liberation Army Cancer Centre, Nanjing Bayi Hospital, Nanjing, Jiangsu, People's Republic of China.; (Hu B) , Huazhong University of Science and Technology and Tongji Hospital, Wuhan; , Shanghai Changzheng Hospital, Shanghai; , Peking Union Medical College Hospital; , Beijing University Cancer Hospital, Beijing; , Anhui Provincial Hospital, Anhui; , Tianjin Medical University Cancer Institute and Hospital, National Clinical Research Center for Cancer, Tianjin; , Guangdong Lung Cancer Institute, Guangdong General Hospital, Guangdong; , Tangdu Hospital and Tangdu Comprehensive Cancer Center, Cancer Institute, 4th Military Medical University, Xi'an; , Sun Yat-Sen University Cancer Center, Guangzhou; , 3rd Affiliated Hospital of Harbin Medical University, Harbin, Heilongjiang; and , The People's Liberation Army Cancer Centre, Nanjing Bayi Hospital, Nanjing, Jiangsu, People's Republic of China.; (Wang K) , Huazhong University of Science and Technology and Tongji Hospital, Wuhan; , Shanghai Changzheng Hospital, Shanghai; , Peking Union Medical College Hospital; , Beijing University Cancer Hospital, Beijing; , Anhui Provincial Hospital, Anhui; , Tianjin Medical University Cancer Institute and Hospital, National Clinical Research Center for Cancer, Tianjin; , Guangdong Lung Cancer Institute, Guangdong General Hospital, Guangdong; , Tangdu Hospital and Tangdu Comprehensive Cancer Center, Cancer Institute, 4th Military Medical University, Xi'an; , Sun Yat-Sen University Cancer Center, Guangzhou; , 3rd Affiliated Hospital of Harbin Medical University, Harbin, Heilongjiang; and , The People's Liberation Army Cancer Centre, Nanjing Bayi Hospital, Nanjing, Jiangsu, People's Republic of China.; (Li PP) , Huazhong University of Science and Technology and Tongji Hospital, Wuhan; , Shanghai Changzheng Hospital, Shanghai; , Peking Union Medical College Hospital; , Beijing University Cancer Hospital, Beijing; , Anhui Provincial Hospital, Anhui; , Tianjin Medical University Cancer Institute and Hospital, National Clinical Research Center for Cancer, Tianjin; , Guangdong Lung Cancer Institute, Guangdong General Hospital, Guangdong; , Tangdu Hospital and Tangdu Comprehensive Cancer Center, Cancer Institute, 4th Military Medical University, Xi'an; , Sun Yat-Sen University Cancer Center, Guangzhou; , 3rd Affiliated Hospital of

Harbin Medical University, Harbin, Heilongjiang; and , The People's Liberation Army Cancer Centre, Nanjing Bayi Hospital, Nanjing, Jiangsu, People's Republic of China.; (Wu YL) , Huazhong University of Science and Technology and Tongji Hospital, Wuhan; , Shanghai Changzheng Hospital, Shanghai; , Peking Union Medical College Hospital; , Beijing University Cancer Hospital, Beijing; , Anhui Provincial Hospital, Anhui; , Tianjin Medical University Cancer Institute and Hospital, National Clinical Research Center for Cancer, Tianjin; , Guangdong Lung Cancer Institute, Guangdong General Hospital, Guangdong; , Tangdu Hospital and Tangdu Comprehensive Cancer Center, Cancer Institute, 4th Military Medical University, Xi'an; , Sun Yat-Sen University Cancer Center, Guangzhou; , 3rd Affiliated Hospital of Harbin Medical University, Harbin, Heilongjiang; and , The People's Liberation Army Cancer Centre, Nanjing Bayi Hospital, Nanjing, Jiangsu, People's Republic of China.; (Zhang HL) , Huazhong University of Science and Technology and Tongji Hospital, Wuhan; , Shanghai Changzheng Hospital, Shanghai; , Peking Union Medical College Hospital; , Beijing University Cancer Hospital, Beijing; , Anhui Provincial Hospital, Anhui; , Tianjin Medical University Cancer Institute and Hospital, National Clinical Research Center for Cancer, Tianjin; , Guangdong Lung Cancer Institute, Guangdong General Hospital, Guangdong; , Tangdu Hospital and Tangdu Comprehensive Cancer Center, Cancer Institute, 4th Military Medical University, Xi'an; , Sun Yat-Sen University Cancer Center, Guangzhou; , 3rd Affiliated Hospital of Harbin Medical University, Harbin, Heilongjiang; and , The People's Liberation Army Cancer Centre, Nanjing Bayi Hospital, Nanjing, Jiangsu, People's Republic of China.; (Zhang L) , Huazhong University of Science and Technology and Tongji Hospital, Wuhan; , Shanghai Changzheng Hospital, Shanghai; , Peking Union Medical College Hospital; , Beijing University Cancer Hospital, Beijing; , Anhui Provincial Hospital, Anhui; , Tianjin Medical University Cancer Institute and Hospital, National Clinical Research Center for Cancer, Tianjin; , Guangdong Lung Cancer Institute, Guangdong General Hospital, Guangdong; , Tangdu Hospital and Tangdu Comprehensive Cancer Center, Cancer Institute, 4th Military Medical University, Xi'an; , Sun Yat-Sen University Cancer Center, Guangzhou; , 3rd Affiliated Hospital of Harbin Medical University, Harbin, Heilongjiang; and , The People's Liberation Army Cancer Centre, Nanjing Bayi Hospital, Nanjing, Jiangsu, People's Republic of China.; (Zhang QY) , Huazhong University of Science and Technology and Tongji Hospital, Wuhan; , Shanghai Changzheng Hospital, Shanghai; , Peking Union Medical College Hospital; , Beijing University Cancer Hospital, Beijing; , Anhui Provincial Hospital, Anhui; , Tianjin Medical University Cancer Institute and Hospital, National Clinical Research Center for Cancer, Tianjin; , Guangdong Lung Cancer Institute, Guangdong General Hospital, Guangdong; , Tangdu Hospital and Tangdu Comprehensive Cancer Center, Cancer Institute, 4th Military Medical University, Xi'an; , Sun Yat-Sen University Cancer Center, Guangzhou; , 3rd Affiliated Hospital of Harbin Medical University, Harbin, Heilongjiang; and , The People's Liberation Army Cancer Centre, Nanjing Bayi Hospital, Nanjing, Jiangsu, People's Republic of China.; (Qin SK) , Huazhong University of Science and Technology and Tongji Hospital, Wuhan; , Shanghai Changzheng Hospital, Shanghai; , Peking Union Medical College Hospital; , Beijing University Cancer Hospital, Beijing; , Anhui Provincial Hospital, Anhui; , Tianjin Medical University Cancer Institute and Hospital, National Clinical Research Center for Cancer, Tianjin; , Guangdong Lung Cancer Institute, Guangdong General Hospital, Guangdong; , Tangdu Hospital and Tangdu Comprehensive Cancer Center, Cancer Institute, 4th Military Medical University, Xi'an; , Sun Yat-Sen University Cancer Center, Guangzhou; , 3rd Affiliated Hospital of

Harbin Medical University, Harbin, Heilongjiang; and , The People's Liberation Army Cancer Centre, Nanjing Bayi Hospital, Nanjing, Jiangsu, People's Republic of China. (Yu SY) , Huazhong University of Science and Technology and Tongji Hospital, Wuhan; , Shanghai Changzheng Hospital, Shanghai; , Peking Union Medical College Hospital; , Beijing University Cancer Hospital, Beijing; , Anhui Provincial Hospital, Anhui; , Tianjin Medical University Cancer Institute and Hospital, National Clinical Research Center for Cancer, Tianjin; , Guangdong Lung Cancer Institute, Guangdong General Hospital, Guangdong; , Tangdu Hospital and Tangdu Comprehensive Cancer Center, Cancer Institute, 4th Military Medical University, Xi'an; , Sun Yat-Sen University Cancer Center, Guangzhou; , 3rd Affiliated Hospital of Harbin Medical University, Harbin, Heilongjiang; and , The People's Liberation Army Cancer Centre, Nanjing Bayi Hospital, Nanjing, Jiangsu, People's Republic of China.; (Wang JJ) , Huazhong University of Science and Technology and Tongji Hospital, Wuhan; , Shanghai Changzheng Hospital, Shanghai; , Peking Union Medical College Hospital; , Beijing University Cancer Hospital, Beijing; , Anhui Provincial Hospital, Anhui; , Tianjin Medical University Cancer Institute and Hospital, National Clinical Research Center for Cancer, Tianjin; , Guangdong Lung Cancer Institute, Guangdong General Hospital, Guangdong; , Tangdu Hospital and Tangdu Comprehensive Cancer Center, Cancer Institute, 4th Military Medical University, Xi'an; , Sun Yat-Sen University Cancer Center, Guangzhou; , 3rd Affiliated Hospital of Harbin Medical University, Harbin, Heilongjiang; and , The People's Liberation Army Cancer Centre, Nanjing Bayi Hospital, Nanjing, Jiangsu, People's Republic of China.; (Huang YG) , Huazhong University of Science and Technology and Tongji Hospital, Wuhan; , Shanghai Changzheng Hospital, Shanghai; , Peking Union Medical College Hospital; , Beijing University Cancer Hospital, Beijing; , Anhui Provincial Hospital, Anhui; , Tianjin Medical University Cancer Institute and Hospital, National Clinical Research Center for Cancer, Tianjin; , Guangdong Lung Cancer Institute, Guangdong General Hospital, Guangdong; , Tangdu Hospital and Tangdu Comprehensive Cancer Center, Cancer Institute, 4th Military Medical University, Xi'an; , Sun Yat-Sen University Cancer Center, Guangzhou; , 3rd Affiliated Hospital of Harbin Medical University, Harbin, Heilongjiang; and , The People's Liberation Army Cancer Centre, Nanjing Bayi Hospital, Nanjing, Jiangsu, People's Republic of China.; (Hu B) , Huazhong University of Science and Technology and Tongji Hospital, Wuhan; , Shanghai Changzheng Hospital, Shanghai; , Peking Union Medical College Hospital; , Beijing University Cancer Hospital, Beijing; , Anhui Provincial Hospital, Anhui; , Tianjin Medical University Cancer Institute and Hospital, National Clinical Research Center for Cancer, Tianjin; , Guangdong Lung Cancer Institute, Guangdong General Hospital, Guangdong; , Tangdu Hospital and Tangdu Comprehensive Cancer Center, Cancer Institute, 4th Military Medical University, Xi'an; , Sun Yat-Sen University Cancer Center, Guangzhou; , 3rd Affiliated Hospital of Harbin Medical University, Harbin, Heilongjiang; and , The People's Liberation Army Cancer Centre, Nanjing Bayi Hospital, Nanjing, Jiangsu, People's Republic of China.; (Wang K) , Huazhong University of Science and Technology and Tongji Hospital, Wuhan; , Shanghai Changzheng Hospital, Shanghai; , Peking Union Medical College Hospital; , Beijing University Cancer Hospital, Beijing; , Anhui Provincial Hospital, Anhui; , Tianjin Medical University Cancer Institute and Hospital, National Clinical Research Center for Cancer, Tianjin; , Guangdong Lung Cancer Institute, Guangdong General Hospital, Guangdong; , Tangdu Hospital and Tangdu Comprehensive Cancer Center, Cancer Institute, 4th Military Medical University, Xi'an; , Sun Yat-Sen University Cancer Center, Guangzhou; , 3rd Affiliated Hospital of

Harbin Medical University, Harbin, Heilongjiang; and , The People's Liberation Army Cancer Centre, Nanjing Bayi Hospital, Nanjing, Jiangsu, People's Republic of China.; (Li PP) , Huazhong University of Science and Technology and Tongji Hospital, Wuhan; , Shanghai Changzheng Hospital, Shanghai; , Peking Union Medical College Hospital; , Beijing University Cancer Hospital, Beijing; , Anhui Provincial Hospital, Anhui; , Tianjin Medical University Cancer Institute and Hospital, National Clinical Research Center for Cancer, Tianjin; , Guangdong Lung Cancer Institute, Guangdong General Hospital, Guangdong; , Tangdu Hospital and Tangdu Comprehensive Cancer Center, Cancer Institute, 4th Military Medical University, Xi'an; , Sun Yat-Sen University Cancer Center, Guangzhou; , 3rd Affiliated Hospital of Harbin Medical University, Harbin, Heilongjiang; and , The People's Liberation Army Cancer Centre, Nanjing Bayi Hospital, Nanjing, Jiangsu, People's Republic of China.; (Wu YL) , Huazhong University of Science and Technology and Tongji Hospital, Wuhan; , Shanghai Changzheng Hospital, Shanghai; , Peking Union Medical College Hospital; , Beijing University Cancer Hospital, Beijing; , Anhui Provincial Hospital, Anhui; , Tianjin Medical University Cancer Institute and Hospital, National Clinical Research Center for Cancer, Tianjin; , Guangdong Lung Cancer Institute, Guangdong General Hospital, Guangdong; , Tangdu Hospital and Tangdu Comprehensive Cancer Center, Cancer Institute, 4th Military Medical University, Xi'an; , Sun Yat-Sen University Cancer Center, Guangzhou; , 3rd Affiliated Hospital of Harbin Medical University, Harbin, Heilongjiang; and , The People's Liberation Army Cancer Centre, Nanjing Bayi Hospital, Nanjing, Jiangsu, People's Republic of China.; (Zhang HL) , Huazhong University of Science and Technology and Tongji Hospital, Wuhan; , Shanghai Changzheng Hospital, Shanghai; , Peking Union Medical College Hospital; , Beijing University Cancer Hospital, Beijing; , Anhui Provincial Hospital, Anhui; , Tianjin Medical University Cancer Institute and Hospital, National Clinical Research Center for Cancer, Tianjin; , Guangdong Lung Cancer Institute, Guangdong General Hospital, Guangdong; , Tangdu Hospital and Tangdu Comprehensive Cancer Center, Cancer Institute, 4th Military Medical University, Xi'an; , Sun Yat-Sen University Cancer Center, Guangzhou; , 3rd Affiliated Hospital of Harbin Medical University, Harbin, Heilongjiang; and , The People's Liberation Army Cancer Centre, Nanjing Bayi Hospital, Nanjing, Jiangsu, People's Republic of China.; (Zhang L) , Huazhong University of Science and Technology and Tongji Hospital, Wuhan; , Shanghai Changzheng Hospital, Shanghai; , Peking Union Medical College Hospital; , Beijing University Cancer Hospital, Beijing; , Anhui Provincial Hospital, Anhui; , Tianjin Medical University Cancer Institute and Hospital, National Clinical Research Center for Cancer, Tianjin; , Guangdong Lung Cancer Institute, Guangdong General Hospital, Guangdong; , Tangdu Hospital and Tangdu Comprehensive Cancer Center, Cancer Institute, 4th Military Medical University, Xi'an; , Sun Yat-Sen University Cancer Center, Guangzhou; , 3rd Affiliated Hospital of Harbin Medical University, Harbin, Heilongjiang; and , The People's Liberation Army Cancer Centre, Nanjing Bayi Hospital, Nanjing, Jiangsu, People's Republic of China.; (Zhang QY) , Huazhong University of Science and Technology and Tongji Hospital, Wuhan; , Shanghai Changzheng Hospital, Shanghai; , Peking Union Medical College Hospital; , Beijing University Cancer Hospital, Beijing; , Anhui Provincial Hospital, Anhui; , Tianjin Medical University Cancer Institute and Hospital, National Clinical Research Center for Cancer, Tianjin; , Guangdong Lung Cancer Institute, Guangdong General Hospital, Guangdong; , Tangdu Hospital and Tangdu Comprehensive Cancer Center, Cancer Institute, 4th Military Medical University, Xi'an; , Sun Yat-Sen University Cancer Center, Guangzhou; , 3rd Affiliated Hospital of

Harbin Medical University, Harbin, Heilongjiang; and , The People's Liberation Army Cancer Centre, Nanjing Bayi Hospital, Nanjing, Jiangsu, People's Republic of China.; (Qin SK) , Huazhong University of Science and Technology and Tongji Hospital, Wuhan; , Shanghai Changzheng Hospital, Shanghai; , Peking Union Medical College Hospital; , Beijing University Cancer Hospital, Beijing; , Anhui Provincial Hospital, Anhui; , Tianjin Medical University Cancer Institute and Hospital, National Clinical Research Center for Cancer, Tianjin; , Guangdong Lung Cancer Institute, Guangdong General Hospital, Guangdong; , Tangdu Hospital and Tangdu Comprehensive Cancer Center, Cancer Institute, 4th Military Medical University, Xi'an; , Sun Yat-Sen University Cancer Center, Guangzhou; , 3rd Affiliated Hospital of Harbin Medical University, Harbin, Heilongjiang; and , The People's Liberation Army Cancer Centre, Nanjing Bayi Hospital, Nanjing, Jiangsu, People's Republic of China. (Yu SY) , Huazhong University of Science and Technology and Tongji Hospital, Wuhan; , Shanghai Changzheng Hospital, Shanghai; , Peking Union Medical College Hospital; , Beijing University Cancer Hospital, Beijing; , Anhui Provincial Hospital, Anhui; , Tianjin Medical University Cancer Institute and Hospital, National Clinical Research Center for Cancer, Tianjin; , Guangdong Lung Cancer Institute, Guangdong General Hospital, Guangdong; , Tangdu Hospital and Tangdu Comprehensive Cancer Center, Cancer Institute, 4th Military Medical University, Xi'an; , Sun Yat-Sen University Cancer Center, Guangzhou; , 3rd Affiliated Hospital of Harbin Medical University, Harbin, Heilongjiang; and , The People's Liberation Army Cancer Centre, Nanjing Bayi Hospital, Nanjing, Jiangsu, People's Republic of China.; (Wang JJ) , Huazhong University of Science and Technology and Tongji Hospital, Wuhan; , Shanghai Changzheng Hospital, Shanghai; , Peking Union Medical College Hospital; , Beijing University Cancer Hospital, Beijing; , Anhui Provincial Hospital, Anhui; , Tianjin Medical University Cancer Institute and Hospital, National Clinical Research Center for Cancer, Tianjin; , Guangdong Lung Cancer Institute, Guangdong General Hospital, Guangdong; , Tangdu Hospital and Tangdu Comprehensive Cancer Center, Cancer Institute, 4th Military Medical University, Xi'an; , Sun Yat-Sen University Cancer Center, Guangzhou; , 3rd Affiliated Hospital of Harbin Medical University, Harbin, Heilongjiang; and , The People's Liberation Army Cancer Centre, Nanjing Bayi Hospital, Nanjing, Jiangsu, People's Republic of China.; (Huang YG) , Huazhong University of Science and Technology and Tongji Hospital, Wuhan; , Shanghai Changzheng Hospital, Shanghai; , Peking Union Medical College Hospital; , Beijing University Cancer Hospital, Beijing; , Anhui Provincial Hospital, Anhui; , Tianjin Medical University Cancer Institute and Hospital, National Clinical Research Center for Cancer, Tianjin; , Guangdong Lung Cancer Institute, Guangdong General Hospital, Guangdong; , Tangdu Hospital and Tangdu Comprehensive Cancer Center, Cancer Institute, 4th Military Medical University, Xi'an; , Sun Yat-Sen University Cancer Center, Guangzhou; , 3rd Affiliated Hospital of Harbin Medical University, Harbin, Heilongjiang; and , The People's Liberation Army Cancer Centre, Nanjing Bayi Hospital, Nanjing, Jiangsu, People's Republic of China.; (Hu B) , Huazhong University of Science and Technology and Tongji Hospital, Wuhan; , Shanghai Changzheng Hospital, Shanghai; , Peking Union Medical College Hospital; , Beijing University Cancer Hospital, Beijing; , Anhui Provincial Hospital, Anhui; , Tianjin Medical University Cancer Institute and Hospital, National Clinical Research Center for Cancer, Tianjin; , Guangdong Lung Cancer Institute, Guangdong General Hospital, Guangdong; , Tangdu Hospital and Tangdu Comprehensive Cancer Center, Cancer Institute, 4th Military Medical University, Xi'an; , Sun Yat-Sen University Cancer Center, Guangzhou; , 3rd Affiliated Hospital of

Harbin Medical University, Harbin, Heilongjiang; and , The People's Liberation Army Cancer Centre, Nanjing Bayi Hospital, Nanjing, Jiangsu, People's Republic of China.; (Wang K) , Huazhong University of Science and Technology and Tongji Hospital, Wuhan; , Shanghai Changzheng Hospital, Shanghai; , Peking Union Medical College Hospital; , Beijing University Cancer Hospital, Beijing; , Anhui Provincial Hospital, Anhui; , Tianjin Medical University Cancer Institute and Hospital, National Clinical Research Center for Cancer, Tianjin; , Guangdong Lung Cancer Institute, Guangdong General Hospital, Guangdong; , Tangdu Hospital and Tangdu Comprehensive Cancer Center, Cancer Institute, 4th Military Medical University, Xi'an; , Sun Yat-Sen University Cancer Center, Guangzhou; , 3rd Affiliated Hospital of Harbin Medical University, Harbin, Heilongjiang; and , The People's Liberation Army Cancer Centre, Nanjing Bayi Hospital, Nanjing, Jiangsu, People's Republic of China.; (Li PP) , Huazhong University of Science and Technology and Tongji Hospital, Wuhan; , Shanghai Changzheng Hospital, Shanghai; , Peking Union Medical College Hospital; , Beijing University Cancer Hospital, Beijing; , Anhui Provincial Hospital, Anhui; , Tianjin Medical University Cancer Institute and Hospital, National Clinical Research Center for Cancer, Tianjin; , Guangdong Lung Cancer Institute, Guangdong General Hospital, Guangdong; , Tangdu Hospital and Tangdu Comprehensive Cancer Center, Cancer Institute, 4th Military Medical University, Xi'an; , Sun Yat-Sen University Cancer Center, Guangzhou; , 3rd Affiliated Hospital of Harbin Medical University, Harbin, Heilongjiang; and , The People's Liberation Army Cancer Centre, Nanjing Bayi Hospital, Nanjing, Jiangsu, People's Republic of China.; (Wu YL) , Huazhong University of Science and Technology and Tongji Hospital, Wuhan; , Shanghai Changzheng Hospital, Shanghai; , Peking Union Medical College Hospital; , Beijing University Cancer Hospital, Beijing; , Anhui Provincial Hospital, Anhui; , Tianjin Medical University Cancer Institute and Hospital, National Clinical Research Center for Cancer, Tianjin; , Guangdong Lung Cancer Institute, Guangdong General Hospital, Guangdong; , Tangdu Hospital and Tangdu Comprehensive Cancer Center, Cancer Institute, 4th Military Medical University, Xi'an; , Sun Yat-Sen University Cancer Center, Guangzhou; , 3rd Affiliated Hospital of Harbin Medical University, Harbin, Heilongjiang; and , The People's Liberation Army Cancer Centre, Nanjing Bayi Hospital, Nanjing, Jiangsu, People's Republic of China.; (Zhang HL) , Huazhong University of Science and Technology and Tongji Hospital, Wuhan; , Shanghai Changzheng Hospital, Shanghai; , Peking Union Medical College Hospital; , Beijing University Cancer Hospital, Beijing; , Anhui Provincial Hospital, Anhui; , Tianjin Medical University Cancer Institute and Hospital, National Clinical Research Center for Cancer, Tianjin; , Guangdong Lung Cancer Institute, Guangdong General Hospital, Guangdong; , Tangdu Hospital and Tangdu Comprehensive Cancer Center, Cancer Institute, 4th Military Medical University, Xi'an; , Sun Yat-Sen University Cancer Center, Guangzhou; , 3rd Affiliated Hospital of Harbin Medical University, Harbin, Heilongjiang; and , The People's Liberation Army Cancer Centre, Nanjing Bayi Hospital, Nanjing, Jiangsu, People's Republic of China.; (Zhang L) , Huazhong University of Science and Technology and Tongji Hospital, Wuhan; , Shanghai Changzheng Hospital, Shanghai; , Peking Union Medical College Hospital; , Beijing University Cancer Hospital, Beijing; , Anhui Provincial Hospital, Anhui; , Tianjin Medical University Cancer Institute and Hospital, National Clinical Research Center for Cancer, Tianjin; , Guangdong Lung Cancer Institute, Guangdong General Hospital, Guangdong; , Tangdu Hospital and Tangdu Comprehensive Cancer Center, Cancer Institute, 4th Military Medical University, Xi'an; , Sun Yat-Sen University Cancer Center, Guangzhou; , 3rd Affiliated Hospital of

Harbin Medical University, Harbin, Heilongjiang; and , The People's Liberation Army Cancer Centre, Nanjing Bayi Hospital, Nanjing, Jiangsu, People's Republic of China.; (Zhang QY) , Huazhong University of Science and Technology and Tongji Hospital, Wuhan; , Shanghai Changzheng Hospital, Shanghai; , Peking Union Medical College Hospital; , Beijing University Cancer Hospital, Beijing; , Anhui Provincial Hospital, Anhui; , Tianjin Medical University Cancer Institute and Hospital, National Clinical Research Center for Cancer, Tianjin; , Guangdong Lung Cancer Institute, Guangdong General Hospital, Guangdong; , Tangdu Hospital and Tangdu Comprehensive Cancer Center, Cancer Institute, 4th Military Medical University, Xi'an; , Sun Yat-Sen University Cancer Center, Guangzhou; , 3rd Affiliated Hospital of Harbin Medical University, Harbin, Heilongjiang; and , The People's Liberation Army Cancer Centre, Nanjing Bayi Hospital, Nanjing, Jiangsu, People's Republic of China.; (Qin SK) , Huazhong University of Science and Technology and Tongji Hospital, Wuhan; , Shanghai Changzheng Hospital, Shanghai; , Peking Union Medical College Hospital; , Beijing University Cancer Hospital, Beijing; , Anhui Provincial Hospital, Anhui; , Tianjin Medical University Cancer Institute and Hospital, National Clinical Research Center for Cancer, Tianjin; , Guangdong Lung Cancer Institute, Guangdong General Hospital, Guangdong; , Tangdu Hospital and Tangdu Comprehensive Cancer Center, Cancer Institute, 4th Military Medical University, Xi'an; , Sun Yat-Sen University Cancer Center, Guangzhou; , 3rd Affiliated Hospital of Harbin Medical University, Harbin, Heilongjiang; and , The People's Liberation Army Cancer Centre, Nanjing Bayi Hospital, Nanjing, Jiangsu, People's Republic of China. (Yu SY) , Huazhong University of Science and Technology and Tongji Hospital, Wuhan; , Shanghai Changzheng Hospital, Shanghai; , Peking Union Medical College Hospital; , Beijing University Cancer Hospital, Beijing; , Anhui Provincial Hospital, Anhui; , Tianjin Medical University Cancer Institute and Hospital, National Clinical Research Center for Cancer, Tianjin; , Guangdong Lung Cancer Institute, Guangdong General Hospital, Guangdong; , Tangdu Hospital and Tangdu Comprehensive Cancer Center, Cancer Institute, 4th Military Medical University, Xi'an; , Sun Yat-Sen University Cancer Center, Guangzhou; , 3rd Affiliated Hospital of Harbin Medical University, Harbin, Heilongjiang; and , The People's Liberation Army Cancer Centre, Nanjing Bayi Hospital, Nanjing, Jiangsu, People's Republic of China.; (Wang JJ) , Huazhong University of Science and Technology and Tongji Hospital, Wuhan; , Shanghai Changzheng Hospital, Shanghai; , Peking Union Medical College Hospital; , Beijing University Cancer Hospital, Beijing; , Anhui Provincial Hospital, Anhui; , Tianjin Medical University Cancer Institute and Hospital, National Clinical Research Center for Cancer, Tianjin; , Guangdong Lung Cancer Institute, Guangdong General Hospital, Guangdong; , Tangdu Hospital and Tangdu Comprehensive Cancer Center, Cancer Institute, 4th Military Medical University, Xi'an; , Sun Yat-Sen University Cancer Center, Guangzhou; , 3rd Affiliated Hospital of Harbin Medical University, Harbin, Heilongjiang; and , The People's Liberation Army Cancer Centre, Nanjing Bayi Hospital, Nanjing, Jiangsu, People's Republic of China.; (Huang YG) , Huazhong University of Science and Technology and Tongji Hospital, Wuhan; , Shanghai Changzheng Hospital, Shanghai; , Peking Union Medical College Hospital; , Beijing University Cancer Hospital, Beijing; , Anhui Provincial Hospital, Anhui; , Tianjin Medical University Cancer Institute and Hospital, National Clinical Research Center for Cancer, Tianjin; , Guangdong Lung Cancer Institute, Guangdong General Hospital, Guangdong; , Tangdu Hospital and Tangdu Comprehensive Cancer Center, Cancer Institute, 4th Military Medical University, Xi'an; , Sun Yat-Sen University Cancer Center, Guangzhou; , 3rd Affiliated Hospital of

Harbin Medical University, Harbin, Heilongjiang; and , The People's Liberation Army Cancer Centre, Nanjing Bayi Hospital, Nanjing, Jiangsu, People's Republic of China.; (Hu B) , Huazhong University of Science and Technology and Tongji Hospital, Wuhan; , Shanghai Changzheng Hospital, Shanghai; , Peking Union Medical College Hospital; , Beijing University Cancer Hospital, Beijing; , Anhui Provincial Hospital, Anhui; , Tianjin Medical University Cancer Institute and Hospital, National Clinical Research Center for Cancer, Tianjin; , Guangdong Lung Cancer Institute, Guangdong General Hospital, Guangdong; , Tangdu Hospital and Tangdu Comprehensive Cancer Center, Cancer Institute, 4th Military Medical University, Xi'an; , Sun Yat-Sen University Cancer Center, Guangzhou; , 3rd Affiliated Hospital of Harbin Medical University, Harbin, Heilongjiang; and , The People's Liberation Army Cancer Centre, Nanjing Bayi Hospital, Nanjing, Jiangsu, People's Republic of China.; (Wang K) , Huazhong University of Science and Technology and Tongji Hospital, Wuhan; , Shanghai Changzheng Hospital, Shanghai; , Peking Union Medical College Hospital; , Beijing University Cancer Hospital, Beijing; , Anhui Provincial Hospital, Anhui; , Tianjin Medical University Cancer Institute and Hospital, National Clinical Research Center for Cancer, Tianjin; , Guangdong Lung Cancer Institute, Guangdong General Hospital, Guangdong; , Tangdu Hospital and Tangdu Comprehensive Cancer Center, Cancer Institute, 4th Military Medical University, Xi'an; , Sun Yat-Sen University Cancer Center, Guangzhou; , 3rd Affiliated Hospital of Harbin Medical University, Harbin, Heilongjiang; and , The People's Liberation Army Cancer Centre, Nanjing Bayi Hospital, Nanjing, Jiangsu, People's Republic of China.; (Li PP) , Huazhong University of Science and Technology and Tongji Hospital, Wuhan; , Shanghai Changzheng Hospital, Shanghai; , Peking Union Medical College Hospital; , Beijing University Cancer Hospital, Beijing; , Anhui Provincial Hospital, Anhui; , Tianjin Medical University Cancer Institute and Hospital, National Clinical Research Center for Cancer, Tianjin; , Guangdong Lung Cancer Institute, Guangdong General Hospital, Guangdong; , Tangdu Hospital and Tangdu Comprehensive Cancer Center, Cancer Institute, 4th Military Medical University, Xi'an; , Sun Yat-Sen University Cancer Center, Guangzhou; , 3rd Affiliated Hospital of Harbin Medical University, Harbin, Heilongjiang; and , The People's Liberation Army Cancer Centre, Nanjing Bayi Hospital, Nanjing, Jiangsu, People's Republic of China.; (Wu YL) , Huazhong University of Science and Technology and Tongji Hospital, Wuhan; , Shanghai Changzheng Hospital, Shanghai; , Peking Union Medical College Hospital; , Beijing University Cancer Hospital, Beijing; , Anhui Provincial Hospital, Anhui; , Tianjin Medical University Cancer Institute and Hospital, National Clinical Research Center for Cancer, Tianjin; , Guangdong Lung Cancer Institute, Guangdong General Hospital, Guangdong; , Tangdu Hospital and Tangdu Comprehensive Cancer Center, Cancer Institute, 4th Military Medical University, Xi'an; , Sun Yat-Sen University Cancer Center, Guangzhou; , 3rd Affiliated Hospital of Harbin Medical University, Harbin, Heilongjiang; and , The People's Liberation Army Cancer Centre, Nanjing Bayi Hospital, Nanjing, Jiangsu, People's Republic of China.; (Zhang HL) , Huazhong University of Science and Technology and Tongji Hospital, Wuhan; , Shanghai Changzheng Hospital, Shanghai; , Peking Union Medical College Hospital; , Beijing University Cancer Hospital, Beijing; , Anhui Provincial Hospital, Anhui; , Tianjin Medical University Cancer Institute and Hospital, National Clinical Research Center for Cancer, Tianjin; , Guangdong Lung Cancer Institute, Guangdong General Hospital, Guangdong; , Tangdu Hospital and Tangdu Comprehensive Cancer Center, Cancer Institute, 4th Military Medical University, Xi'an; , Sun Yat-Sen University Cancer Center, Guangzhou; , 3rd Affiliated Hospital of

Harbin Medical University, Harbin, Heilongjiang; and , The People's Liberation Army Cancer Centre, Nanjing Bayi Hospital, Nanjing, Jiangsu, People's Republic of China.; (Zhang L) , Huazhong University of Science and Technology and Tongji Hospital, Wuhan; , Shanghai Changzheng Hospital, Shanghai; , Peking Union Medical College Hospital; , Beijing University Cancer Hospital, Beijing; , Anhui Provincial Hospital, Anhui; , Tianjin Medical University Cancer Institute and Hospital, National Clinical Research Center for Cancer, Tianjin; , Guangdong Lung Cancer Institute, Guangdong General Hospital, Guangdong; , Tangdu Hospital and Tangdu Comprehensive Cancer Center, Cancer Institute, 4th Military Medical University, Xi'an; , Sun Yat-Sen University Cancer Center, Guangzhou; , 3rd Affiliated Hospital of Harbin Medical University, Harbin, Heilongjiang; and , The People's Liberation Army Cancer Centre, Nanjing Bayi Hospital, Nanjing, Jiangsu, People's Republic of China.; (Zhang QY) , Huazhong University of Science and Technology and Tongji Hospital, Wuhan; , Shanghai Changzheng Hospital, Shanghai; , Peking Union Medical College Hospital; , Beijing University Cancer Hospital, Beijing; , Anhui Provincial Hospital, Anhui; , Tianjin Medical University Cancer Institute and Hospital, National Clinical Research Center for Cancer, Tianjin; , Guangdong Lung Cancer Institute, Guangdong General Hospital, Guangdong; , Tangdu Hospital and Tangdu Comprehensive Cancer Center, Cancer Institute, 4th Military Medical University, Xi'an; , Sun Yat-Sen University Cancer Center, Guangzhou; , 3rd Affiliated Hospital of Harbin Medical University, Harbin, Heilongjiang; and , The People's Liberation Army Cancer Centre, Nanjing Bayi Hospital, Nanjing, Jiangsu, People's Republic of China.; (Qin SK) , Huazhong University of Science and Technology and Tongji Hospital, Wuhan; , Shanghai Changzheng Hospital, Shanghai; , Peking Union Medical College Hospital; , Beijing University Cancer Hospital, Beijing; , Anhui Provincial Hospital, Anhui; , Tianjin Medical University Cancer Institute and Hospital, National Clinical Research Center for Cancer, Tianjin; , Guangdong Lung Cancer Institute, Guangdong General Hospital, Guangdong; , Tangdu Hospital and Tangdu Comprehensive Cancer Center, Cancer Institute, 4th Military Medical University, Xi'an; , Sun Yat-Sen University Cancer Center, Guangzhou; , 3rd Affiliated Hospital of Harbin Medical University, Harbin, Heilongjiang; and , The People's Liberation Army Cancer Centre, Nanjing Bayi Hospital, Nanjing, Jiangsu, People's Republic of China. (Yu SY) , Huazhong University of Science and Technology and Tongji Hospital, Wuhan; , Shanghai Changzheng Hospital, Shanghai; , Peking Union Medical College Hospital; , Beijing University Cancer Hospital, Beijing; , Anhui Provincial Hospital, Anhui; , Tianjin Medical University Cancer Institute and Hospital, National Clinical Research Center for Cancer, Tianjin; , Guangdong Lung Cancer Institute, Guangdong General Hospital, Guangdong; , Tangdu Hospital and Tangdu Comprehensive Cancer Center, Cancer Institute, 4th Military Medical University, Xi'an; , Sun Yat-Sen University Cancer Center, Guangzhou; , 3rd Affiliated Hospital of Harbin Medical University, Harbin, Heilongjiang; and , The People's Liberation Army Cancer Centre, Nanjing Bayi Hospital, Nanjing, Jiangsu, People's Republic of China.; (Wang JJ) , Huazhong University of Science and Technology and Tongji Hospital, Wuhan; , Shanghai Changzheng Hospital, Shanghai; , Peking Union Medical College Hospital; , Beijing University Cancer Hospital, Beijing; , Anhui Provincial Hospital, Anhui; , Tianjin Medical University Cancer Institute and Hospital, National Clinical Research Center for Cancer, Tianjin; , Guangdong Lung Cancer Institute, Guangdong General Hospital, Guangdong; , Tangdu Hospital and Tangdu Comprehensive Cancer Center, Cancer Institute, 4th Military Medical University, Xi'an; , Sun Yat-Sen University Cancer Center, Guangzhou; , 3rd Affiliated Hospital of

Harbin Medical University, Harbin, Heilongjiang; and , The People's Liberation Army Cancer Centre, Nanjing Bayi Hospital, Nanjing, Jiangsu, People's Republic of China.; (Huang YG) , Huazhong University of Science and Technology and Tongji Hospital, Wuhan; , Shanghai Changzheng Hospital, Shanghai; , Peking Union Medical College Hospital; , Beijing University Cancer Hospital, Beijing; , Anhui Provincial Hospital, Anhui; , Tianjin Medical University Cancer Institute and Hospital, National Clinical Research Center for Cancer, Tianjin; , Guangdong Lung Cancer Institute, Guangdong General Hospital, Guangdong; , Tangdu Hospital and Tangdu Comprehensive Cancer Center, Cancer Institute, 4th Military Medical University, Xi'an; , Sun Yat-Sen University Cancer Center, Guangzhou; , 3rd Affiliated Hospital of Harbin Medical University, Harbin, Heilongjiang; and , The People's Liberation Army Cancer Centre, Nanjing Bayi Hospital, Nanjing, Jiangsu, People's Republic of China.; (Hu B) , Huazhong University of Science and Technology and Tongji Hospital, Wuhan; , Shanghai Changzheng Hospital, Shanghai; , Peking Union Medical College Hospital; , Beijing University Cancer Hospital, Beijing; , Anhui Provincial Hospital, Anhui; , Tianjin Medical University Cancer Institute and Hospital, National Clinical Research Center for Cancer, Tianjin; , Guangdong Lung Cancer Institute, Guangdong General Hospital, Guangdong; , Tangdu Hospital and Tangdu Comprehensive Cancer Center, Cancer Institute, 4th Military Medical University, Xi'an; , Sun Yat-Sen University Cancer Center, Guangzhou; , 3rd Affiliated Hospital of Harbin Medical University, Harbin, Heilongjiang; and , The People's Liberation Army Cancer Centre, Nanjing Bayi Hospital, Nanjing, Jiangsu, People's Republic of China.; (Wang K) , Huazhong University of Science and Technology and Tongji Hospital, Wuhan; , Shanghai Changzheng Hospital, Shanghai; , Peking Union Medical College Hospital; , Beijing University Cancer Hospital, Beijing; , Anhui Provincial Hospital, Anhui; , Tianjin Medical University Cancer Institute and Hospital, National Clinical Research Center for Cancer, Tianjin; , Guangdong Lung Cancer Institute, Guangdong General Hospital, Guangdong; , Tangdu Hospital and Tangdu Comprehensive Cancer Center, Cancer Institute, 4th Military Medical University, Xi'an; , Sun Yat-Sen University Cancer Center, Guangzhou; , 3rd Affiliated Hospital of Harbin Medical University, Harbin, Heilongjiang; and , The People's Liberation Army Cancer Centre, Nanjing Bayi Hospital, Nanjing, Jiangsu, People's Republic of China.; (Li PP) , Huazhong University of Science and Technology and Tongji Hospital, Wuhan; , Shanghai Changzheng Hospital, Shanghai; , Peking Union Medical College Hospital; , Beijing University Cancer Hospital, Beijing; , Anhui Provincial Hospital, Anhui; , Tianjin Medical University Cancer Institute and Hospital, National Clinical Research Center for Cancer, Tianjin; , Guangdong Lung Cancer Institute, Guangdong General Hospital, Guangdong; , Tangdu Hospital and Tangdu Comprehensive Cancer Center, Cancer Institute, 4th Military Medical University, Xi'an; , Sun Yat-Sen University Cancer Center, Guangzhou; , 3rd Affiliated Hospital of Harbin Medical University, Harbin, Heilongjiang; and , The People's Liberation Army Cancer Centre, Nanjing Bayi Hospital, Nanjing, Jiangsu, People's Republic of China.; (Wu YL) , Huazhong University of Science and Technology and Tongji Hospital, Wuhan; , Shanghai Changzheng Hospital, Shanghai; , Peking Union Medical College Hospital; , Beijing University Cancer Hospital, Beijing; , Anhui Provincial Hospital, Anhui; , Tianjin Medical University Cancer Institute and Hospital, National Clinical Research Center for Cancer, Tianjin; , Guangdong Lung Cancer Institute, Guangdong General Hospital, Guangdong; , Tangdu Hospital and Tangdu Comprehensive Cancer Center, Cancer Institute, 4th Military Medical University, Xi'an; , Sun Yat-Sen University Cancer Center, Guangzhou; , 3rd Affiliated Hospital of

Harbin Medical University, Harbin, Heilongjiang; and , The People's Liberation Army Cancer Centre, Nanjing Bayi Hospital, Nanjing, Jiangsu, People's Republic of China.; (Zhang HL) , Huazhong University of Science and Technology and Tongji Hospital, Wuhan; , Shanghai Changzheng Hospital, Shanghai; , Peking Union Medical College Hospital; , Beijing University Cancer Hospital, Beijing; , Anhui Provincial Hospital, Anhui; , Tianjin Medical University Cancer Institute and Hospital, National Clinical Research Center for Cancer, Tianjin; , Guangdong Lung Cancer Institute, Guangdong General Hospital, Guangdong; , Tangdu Hospital and Tangdu Comprehensive Cancer Center, Cancer Institute, 4th Military Medical University, Xi'an; , Sun Yat-Sen University Cancer Center, Guangzhou; , 3rd Affiliated Hospital of Harbin Medical University, Harbin, Heilongjiang; and , The People's Liberation Army Cancer Centre, Nanjing Bayi Hospital, Nanjing, Jiangsu, People's Republic of China.; (Zhang L) , Huazhong University of Science and Technology and Tongji Hospital, Wuhan; , Shanghai Changzheng Hospital, Shanghai; , Peking Union Medical College Hospital; , Beijing University Cancer Hospital, Beijing; , Anhui Provincial Hospital, Anhui; , Tianjin Medical University Cancer Institute and Hospital, National Clinical Research Center for Cancer, Tianjin; , Guangdong Lung Cancer Institute, Guangdong General Hospital, Guangdong; , Tangdu Hospital and Tangdu Comprehensive Cancer Center, Cancer Institute, 4th Military Medical University, Xi'an; , Sun Yat-Sen University Cancer Center, Guangzhou; , 3rd Affiliated Hospital of Harbin Medical University, Harbin, Heilongjiang; and , The People's Liberation Army Cancer Centre, Nanjing Bayi Hospital, Nanjing, Jiangsu, People's Republic of China.; (Zhang QY) , Huazhong University of Science and Technology and Tongji Hospital, Wuhan; , Shanghai Changzheng Hospital, Shanghai; , Peking Union Medical College Hospital; , Beijing University Cancer Hospital, Beijing; , Anhui Provincial Hospital, Anhui; , Tianjin Medical University Cancer Institute and Hospital, National Clinical Research Center for Cancer, Tianjin; , Guangdong Lung Cancer Institute, Guangdong General Hospital, Guangdong; , Tangdu Hospital and Tangdu Comprehensive Cancer Center, Cancer Institute, 4th Military Medical University, Xi'an; , Sun Yat-Sen University Cancer Center, Guangzhou; , 3rd Affiliated Hospital of Harbin Medical University, Harbin, Heilongjiang; and , The People's Liberation Army Cancer Centre, Nanjing Bayi Hospital, Nanjing, Jiangsu, People's Republic of China.; (Qin SK) , Huazhong University of Science and Technology and Tongji Hospital, Wuhan; , Shanghai Changzheng Hospital, Shanghai; , Peking Union Medical College Hospital; , Beijing University Cancer Hospital, Beijing; , Anhui Provincial Hospital, Anhui; , Tianjin Medical University Cancer Institute and Hospital, National Clinical Research Center for Cancer, Tianjin; , Guangdong Lung Cancer Institute, Guangdong General Hospital, Guangdong; , Tangdu Hospital and Tangdu Comprehensive Cancer Center, Cancer Institute, 4th Military Medical University, Xi'an; , Sun Yat-Sen University Cancer Center, Guangzhou; , 3rd Affiliated Hospital of Harbin Medical University, Harbin, Heilongjiang; and , The People's Liberation Army Cancer Centre, Nanjing Bayi Hospital, Nanjing, Jiangsu, People's Republic of China.
(Yu SY) , Huazhong University of Science and Technology and Tongji Hospital, Wuhan; , Shanghai Changzheng Hospital, Shanghai; , Peking Union Medical College Hospital; , Beijing University Cancer Hospital, Beijing; , Anhui Provincial Hospital, Anhui; , Tianjin Medical University Cancer Institute and Hospital, National Clinical Research Center for Cancer, Tianjin; , Guangdong Lung Cancer Institute, Guangdong General Hospital, Guangdong; , Tangdu Hospital and Tangdu Comprehensive Cancer Center, Cancer Institute, 4th Military Medical University, Xi'an; , Sun Yat-Sen University Cancer Center, Guangzhou; , 3rd Affiliated Hospital of

Harbin Medical University, Harbin, Heilongjiang; and , The People's Liberation Army Cancer Centre, Nanjing Bayi Hospital, Nanjing, Jiangsu, People's Republic of China.; (Wang JJ) , Huazhong University of Science and Technology and Tongji Hospital, Wuhan; , Shanghai Changzheng Hospital, Shanghai; , Peking Union Medical College Hospital; , Beijing University Cancer Hospital, Beijing; , Anhui Provincial Hospital, Anhui; , Tianjin Medical University Cancer Institute and Hospital, National Clinical Research Center for Cancer, Tianjin; , Guangdong Lung Cancer Institute, Guangdong General Hospital, Guangdong; , Tangdu Hospital and Tangdu Comprehensive Cancer Center, Cancer Institute, 4th Military Medical University, Xi'an; , Sun Yat-Sen University Cancer Center, Guangzhou; , 3rd Affiliated Hospital of Harbin Medical University, Harbin, Heilongjiang; and , The People's Liberation Army Cancer Centre, Nanjing Bayi Hospital, Nanjing, Jiangsu, People's Republic of China.; (Huang YG) , Huazhong University of Science and Technology and Tongji Hospital, Wuhan; , Shanghai Changzheng Hospital, Shanghai; , Peking Union Medical College Hospital; , Beijing University Cancer Hospital, Beijing; , Anhui Provincial Hospital, Anhui; , Tianjin Medical University Cancer Institute and Hospital, National Clinical Research Center for Cancer, Tianjin; , Guangdong Lung Cancer Institute, Guangdong General Hospital, Guangdong; , Tangdu Hospital and Tangdu Comprehensive Cancer Center, Cancer Institute, 4th Military Medical University, Xi'an; , Sun Yat-Sen University Cancer Center, Guangzhou; , 3rd Affiliated Hospital of Harbin Medical University, Harbin, Heilongjiang; and , The People's Liberation Army Cancer Centre, Nanjing Bayi Hospital, Nanjing, Jiangsu, People's Republic of China.; (Hu B) , Huazhong University of Science and Technology and Tongji Hospital, Wuhan; , Shanghai Changzheng Hospital, Shanghai; , Peking Union Medical College Hospital; , Beijing University Cancer Hospital, Beijing; , Anhui Provincial Hospital, Anhui; , Tianjin Medical University Cancer Institute and Hospital, National Clinical Research Center for Cancer, Tianjin; , Guangdong Lung Cancer Institute, Guangdong General Hospital, Guangdong; , Tangdu Hospital and Tangdu Comprehensive Cancer Center, Cancer Institute, 4th Military Medical University, Xi'an; , Sun Yat-Sen University Cancer Center, Guangzhou; , 3rd Affiliated Hospital of Harbin Medical University, Harbin, Heilongjiang; and , The People's Liberation Army Cancer Centre, Nanjing Bayi Hospital, Nanjing, Jiangsu, People's Republic of China.; (Wang K) , Huazhong University of Science and Technology and Tongji Hospital, Wuhan; , Shanghai Changzheng Hospital, Shanghai; , Peking Union Medical College Hospital; , Beijing University Cancer Hospital, Beijing; , Anhui Provincial Hospital, Anhui; , Tianjin Medical University Cancer Institute and Hospital, National Clinical Research Center for Cancer, Tianjin; , Guangdong Lung Cancer Institute, Guangdong General Hospital, Guangdong; , Tangdu Hospital and Tangdu Comprehensive Cancer Center, Cancer Institute, 4th Military Medical University, Xi'an; , Sun Yat-Sen University Cancer Center, Guangzhou; , 3rd Affiliated Hospital of Harbin Medical University, Harbin, Heilongjiang; and , The People's Liberation Army Cancer Centre, Nanjing Bayi Hospital, Nanjing, Jiangsu, People's Republic of China.; (Li PP) , Huazhong University of Science and Technology and Tongji Hospital, Wuhan; , Shanghai Changzheng Hospital, Shanghai; , Peking Union Medical College Hospital; , Beijing University Cancer Hospital, Beijing; , Anhui Provincial Hospital, Anhui; , Tianjin Medical University Cancer Institute and Hospital, National Clinical Research Center for Cancer, Tianjin; , Guangdong Lung Cancer Institute, Guangdong General Hospital, Guangdong; , Tangdu Hospital and Tangdu Comprehensive Cancer Center, Cancer Institute, 4th Military Medical University, Xi'an; , Sun Yat-Sen University Cancer Center, Guangzhou; , 3rd Affiliated Hospital of

Harbin Medical University, Harbin, Heilongjiang; and , The People's Liberation Army Cancer Centre, Nanjing Bayi Hospital, Nanjing, Jiangsu, People's Republic of China.; (Wu YL) , Huazhong University of Science and Technology and Tongji Hospital, Wuhan; , Shanghai Changzheng Hospital, Shanghai; , Peking Union Medical College Hospital; , Beijing University Cancer Hospital, Beijing; , Anhui Provincial Hospital, Anhui; , Tianjin Medical University Cancer Institute and Hospital, National Clinical Research Center for Cancer, Tianjin; , Guangdong Lung Cancer Institute, Guangdong General Hospital, Guangdong; , Tangdu Hospital and Tangdu Comprehensive Cancer Center, Cancer Institute, 4th Military Medical University, Xi'an; , Sun Yat-Sen University Cancer Center, Guangzhou; , 3rd Affiliated Hospital of Harbin Medical University, Harbin, Heilongjiang; and , The People's Liberation Army Cancer Centre, Nanjing Bayi Hospital, Nanjing, Jiangsu, People's Republic of China.; (Zhang HL) , Huazhong University of Science and Technology and Tongji Hospital, Wuhan; , Shanghai Changzheng Hospital, Shanghai; , Peking Union Medical College Hospital; , Beijing University Cancer Hospital, Beijing; , Anhui Provincial Hospital, Anhui; , Tianjin Medical University Cancer Institute and Hospital, National Clinical Research Center for Cancer, Tianjin; , Guangdong Lung Cancer Institute, Guangdong General Hospital, Guangdong; , Tangdu Hospital and Tangdu Comprehensive Cancer Center, Cancer Institute, 4th Military Medical University, Xi'an; , Sun Yat-Sen University Cancer Center, Guangzhou; , 3rd Affiliated Hospital of Harbin Medical University, Harbin, Heilongjiang; and , The People's Liberation Army Cancer Centre, Nanjing Bayi Hospital, Nanjing, Jiangsu, People's Republic of China.; (Zhang L) , Huazhong University of Science and Technology and Tongji Hospital, Wuhan; , Shanghai Changzheng Hospital, Shanghai; , Peking Union Medical College Hospital; , Beijing University Cancer Hospital, Beijing; , Anhui Provincial Hospital, Anhui; , Tianjin Medical University Cancer Institute and Hospital, National Clinical Research Center for Cancer, Tianjin; , Guangdong Lung Cancer Institute, Guangdong General Hospital, Guangdong; , Tangdu Hospital and Tangdu Comprehensive Cancer Center, Cancer Institute, 4th Military Medical University, Xi'an; , Sun Yat-Sen University Cancer Center, Guangzhou; , 3rd Affiliated Hospital of Harbin Medical University, Harbin, Heilongjiang; and , The People's Liberation Army Cancer Centre, Nanjing Bayi Hospital, Nanjing, Jiangsu, People's Republic of China.; (Zhang QY) , Huazhong University of Science and Technology and Tongji Hospital, Wuhan; , Shanghai Changzheng Hospital, Shanghai; , Peking Union Medical College Hospital; , Beijing University Cancer Hospital, Beijing; , Anhui Provincial Hospital, Anhui; , Tianjin Medical University Cancer Institute and Hospital, National Clinical Research Center for Cancer, Tianjin; , Guangdong Lung Cancer Institute, Guangdong General Hospital, Guangdong; , Tangdu Hospital and Tangdu Comprehensive Cancer Center, Cancer Institute, 4th Military Medical University, Xi'an; , Sun Yat-Sen University Cancer Center, Guangzhou; , 3rd Affiliated Hospital of Harbin Medical University, Harbin, Heilongjiang; and , The People's Liberation Army Cancer Centre, Nanjing Bayi Hospital, Nanjing, Jiangsu, People's Republic of China.; (Qin SK) , Huazhong University of Science and Technology and Tongji Hospital, Wuhan; , Shanghai Changzheng Hospital, Shanghai; , Peking Union Medical College Hospital; , Beijing University Cancer Hospital, Beijing; , Anhui Provincial Hospital, Anhui; , Tianjin Medical University Cancer Institute and Hospital, National Clinical Research Center for Cancer, Tianjin; , Guangdong Lung Cancer Institute, Guangdong General Hospital, Guangdong; , Tangdu Hospital and Tangdu Comprehensive Cancer Center, Cancer Institute, 4th Military Medical University, Xi'an; , Sun Yat-Sen University Cancer Center, Guangzhou; , 3rd Affiliated Hospital of

Harbin Medical University, Harbin, Heilongjiang; and , The People's Liberation Army Cancer Centre, Nanjing Bayi Hospital, Nanjing, Jiangsu, People's Republic of China.
Database: PubMed

96. Practice Patterns in Distinguishing Between Background Pain and Breakthrough Pain During Patient Education: a Korean Physician Survey.

Author(s): Shin J; Kim DY; Lee J; Choi YS; Hwang IG; Baek SK; Seo MS; Shim JY

Source: Journal of cancer education : the official journal of the American Association for Cancer Education; 2018; vol. 33 (no. 2); p. 284-292

Publication Date: 2018

Publication Type(s): Journal Article; Research Support, Non-U.S. Gov't

DOI: http://dx.doi.org/10.1007/s13187-016-1113-3

ISSN: 1543-0154

Place of Publication: England

PubMedID: 27623849

Accession Number: 27623849

Available at Journal of cancer education : the official journal of the American Association for Cancer Education - from EBSCO (MEDLINE Complete)

Available at Journal of cancer education : the official journal of the American Association for Cancer Education - from ProQuest (MEDLINE with Full Text) - NHS Version

Available at Journal of cancer education : the official journal of the American Association for Cancer Education - from ProQuest (Health Research Premium) - NHS Version

Keywords: Subject Terms: *Breakthrough pain; *Health knowledge; *Physician practice patterns

Abstract: This study sought to explore the association between physician practice patterns and patient education, with a focus on breakthrough cancer pain (BTcP). A nationwide online survey was conducted by 92 Korean physicians. Thirteen questions on Korean physician's assessment, prescription, patient education practices, and knowledge regarding BTcP were administered. Based on their responses, physicians were divided using two methods: (1) by their patient education practices, where the "education group" always explained the distinction between background pain and BTcP and the "less education group" which explained it less frequently; and (2) by their definition of BTcP, as occurring "after control of background pain" or "regardless of background pain." We compared practice patterns using Fisher's exact test or Student's t test and performed multiple logistic regression analysis. The "education group" (65 physicians, 70.7 %) was more likely than the "less education group" to assess BTcP meticulously (odds ratio [OR] 17.13, 95 % confidence interval [CI] 4.98-58.94), prepare rescue medications in advance (OR 3.67, 95 % CI 1.36-9.90), and give explicit instructions regarding medications (OR 36.68, 95 % CI 5.63-239.15). Physicians who defined BTcP as occurring "after control of background pain" were more likely to explain how to take rescue medication ($P < 0.05$) than physicians who defined BTcP as occurring "regardless of background pain." Korean physicians' BTcP practice patterns may be affected by

whether they consistently educate patients on the distinction between background pain and BTcP, regardless of their knowledge of the definition of BTcP.

Institutions:
(Shin J) Department of Family Medicine, Center for Cancer Supportive Care, Samsung Medical Center, Sungkyunkwan University School of Medicine, Seoul, South Korea.; (Kim DY) Department of Internal Medicine, Dongguk University Ilsan Hospital, Graduate School, Dongguk University-Seoul, Goyang, South Korea.; (Lee J) Department of Biostatistics, Korea University College of Medicine, Seoul, South Korea.; (Choi YS) Department of Family Medicine, Korea University Guro Hospital, Korea University College of Medicine, Seoul, South Korea.; (Hwang IG) Department of Internal Medicine, Chung-Ang University College of Medicine, Seoul, South Korea.; (Baek SK) Department of Internal Medicine, Kyung Hee University Hospital, Kyung Hee University School of Medicine, Seoul, South Korea.; (Seo MS) Department of Family Medicine, Incheon St. Mary's Hospital, Catholic University of Korea, Incheon, South Korea.; (Shim JY) Department of Family Medicine, Gangnam Severance Hospital, Yonsei University College of Medicine, 211 Eonju-ro, Gangnam-gu, Seoul, 06273, South Korea. hope@yuhs.ac.
(Shin J) Department of Family Medicine, Center for Cancer Supportive Care, Samsung Medical Center, Sungkyunkwan University School of Medicine, Seoul, South Korea.; (Kim DY) Department of Internal Medicine, Dongguk University Ilsan Hospital, Graduate School, Dongguk University-Seoul, Goyang, South Korea.; (Lee J) Department of Biostatistics, Korea University College of Medicine, Seoul, South Korea.; (Choi YS) Department of Family Medicine, Korea University Guro Hospital, Korea University College of Medicine, Seoul, South Korea.; (Hwang IG) Department of Internal Medicine, Chung-Ang University College of Medicine, Seoul, South Korea.; (Baek SK) Department of Internal Medicine, Kyung Hee University Hospital, Kyung Hee University School of Medicine, Seoul, South Korea.; (Seo MS) Department of Family Medicine, Incheon St. Mary's Hospital, Catholic University of Korea, Incheon, South Korea.; (Shim JY) Department of Family Medicine, Gangnam Severance Hospital, Yonsei University College of Medicine, 211 Eonju-ro, Gangnam-gu, Seoul, 06273, South Korea. hope@yuhs.ac.
(Shin J) Department of Family Medicine, Center for Cancer Supportive Care, Samsung Medical Center, Sungkyunkwan University School of Medicine, Seoul, South Korea.; (Kim DY) Department of Internal Medicine, Dongguk University Ilsan Hospital, Graduate School, Dongguk University-Seoul, Goyang, South Korea.; (Lee J) Department of Biostatistics, Korea University College of Medicine, Seoul, South Korea.; (Choi YS) Department of Family Medicine, Korea University Guro Hospital, Korea University College of Medicine, Seoul, South Korea.; (Hwang IG) Department of Internal Medicine, Chung-Ang University College of Medicine, Seoul, South Korea.; (Baek SK) Department of Internal Medicine, Kyung Hee University Hospital, Kyung Hee University School of Medicine, Seoul, South Korea.; (Seo MS) Department of Family Medicine, Incheon St. Mary's Hospital, Catholic University of Korea, Incheon, South Korea.; (Shim JY) Department of Family Medicine, Gangnam Severance Hospital, Yonsei University College of Medicine, 211 Eonju-ro, Gangnam-gu, Seoul, 06273, South Korea. hope@yuhs.ac.
(Shin J) Department of Family Medicine, Center for Cancer Supportive Care, Samsung Medical Center, Sungkyunkwan University School of Medicine, Seoul, South Korea.; (Kim DY) Department of Internal Medicine, Dongguk University Ilsan Hospital, Graduate School, Dongguk University-Seoul, Goyang, South Korea.; (Lee J) Department of Biostatistics, Korea University College of Medicine, Seoul, South

Korea.; (Choi YS) Department of Family Medicine, Korea University Guro Hospital, Korea University College of Medicine, Seoul, South Korea.; (Hwang IG) Department of Internal Medicine, Chung-Ang University College of Medicine, Seoul, South Korea.; (Baek SK) Department of Internal Medicine, Kyung Hee University Hospital, Kyung Hee University School of Medicine, Seoul, South Korea.; (Seo MS) Department of Family Medicine, Incheon St. Mary's Hospital, Catholic University of Korea, Incheon, South Korea.; (Shim JY) Department of Family Medicine, Gangnam Severance Hospital, Yonsei University College of Medicine, 211 Eonju-ro, Gangnam-gu, Seoul, 06273, South Korea. hope@yuhs.ac.
(Shin J) Department of Family Medicine, Center for Cancer Supportive Care, Samsung Medical Center, Sungkyunkwan University School of Medicine, Seoul, South Korea.; (Kim DY) Department of Internal Medicine, Dongguk University Ilsan Hospital, Graduate School, Dongguk University-Seoul, Goyang, South Korea.; (Lee J) Department of Biostatistics, Korea University College of Medicine, Seoul, South Korea.; (Choi YS) Department of Family Medicine, Korea University Guro Hospital, Korea University College of Medicine, Seoul, South Korea.; (Hwang IG) Department of Internal Medicine, Chung-Ang University College of Medicine, Seoul, South Korea.; (Baek SK) Department of Internal Medicine, Kyung Hee University Hospital, Kyung Hee University School of Medicine, Seoul, South Korea.; (Seo MS) Department of Family Medicine, Incheon St. Mary's Hospital, Catholic University of Korea, Incheon, South Korea.; (Shim JY) Department of Family Medicine, Gangnam Severance Hospital, Yonsei University College of Medicine, 211 Eonju-ro, Gangnam-gu, Seoul, 06273, South Korea. hope@yuhs.ac.
(Shin J) Department of Family Medicine, Center for Cancer Supportive Care, Samsung Medical Center, Sungkyunkwan University School of Medicine, Seoul, South Korea.; (Kim DY) Department of Internal Medicine, Dongguk University Ilsan Hospital, Graduate School, Dongguk University-Seoul, Goyang, South Korea.; (Lee J) Department of Biostatistics, Korea University College of Medicine, Seoul, South Korea.; (Choi YS) Department of Family Medicine, Korea University Guro Hospital, Korea University College of Medicine, Seoul, South Korea.; (Hwang IG) Department of Internal Medicine, Chung-Ang University College of Medicine, Seoul, South Korea.; (Baek SK) Department of Internal Medicine, Kyung Hee University Hospital, Kyung Hee University School of Medicine, Seoul, South Korea.; (Seo MS) Department of Family Medicine, Incheon St. Mary's Hospital, Catholic University of Korea, Incheon, South Korea.; (Shim JY) Department of Family Medicine, Gangnam Severance Hospital, Yonsei University College of Medicine, 211 Eonju-ro, Gangnam-gu, Seoul, 06273, South Korea. hope@yuhs.ac.
(Shin J) Department of Family Medicine, Center for Cancer Supportive Care, Samsung Medical Center, Sungkyunkwan University School of Medicine, Seoul, South Korea.; (Kim DY) Department of Internal Medicine, Dongguk University Ilsan Hospital, Graduate School, Dongguk University-Seoul, Goyang, South Korea.; (Lee J) Department of Biostatistics, Korea University College of Medicine, Seoul, South Korea.; (Choi YS) Department of Family Medicine, Korea University Guro Hospital, Korea University College of Medicine, Seoul, South Korea.; (Hwang IG) Department of Internal Medicine, Chung-Ang University College of Medicine, Seoul, South Korea.; (Baek SK) Department of Internal Medicine, Kyung Hee University Hospital, Kyung Hee University School of Medicine, Seoul, South Korea.; (Seo MS) Department of Family Medicine, Incheon St. Mary's Hospital, Catholic University of Korea, Incheon, South Korea.; (Shim JY) Department of Family Medicine, Gangnam

Severance Hospital, Yonsei University College of Medicine, 211 Eonju-ro, Gangnam-gu, Seoul, 06273, South Korea. hope@yuhs.ac.
(Shin J) Department of Family Medicine, Center for Cancer Supportive Care, Samsung Medical Center, Sungkyunkwan University School of Medicine, Seoul, South Korea.; (Kim DY) Department of Internal Medicine, Dongguk University Ilsan Hospital, Graduate School, Dongguk University-Seoul, Goyang, South Korea.; (Lee J) Department of Biostatistics, Korea University College of Medicine, Seoul, South Korea.; (Choi YS) Department of Family Medicine, Korea University Guro Hospital, Korea University College of Medicine, Seoul, South Korea.; (Hwang IG) Department of Internal Medicine, Chung-Ang University College of Medicine, Seoul, South Korea.; (Baek SK) Department of Internal Medicine, Kyung Hee University Hospital, Kyung Hee University School of Medicine, Seoul, South Korea.; (Seo MS) Department of Family Medicine, Incheon St. Mary's Hospital, Catholic University of Korea, Incheon, South Korea.; (Shim JY) Department of Family Medicine, Gangnam Severance Hospital, Yonsei University College of Medicine, 211 Eonju-ro, Gangnam-gu, Seoul, 06273, South Korea. hope@yuhs.ac.
Database: PubMed

97. Collaborative practice model for management of pain in patients with cancer.

Author(s): Hammer KJ; Segal EM; Alwan L; Li S; Patel AM; Tran M; Marshall HM

Source: American journal of health-system pharmacy : AJHP : official journal of the American Society of Health-System Pharmacists; Sep 2016; vol. 73 (no. 18); p. 1434-1441

Publication Date: Sep 2016

Publication Type(s): Journal Article

DOI: http://dx.doi.org/10.2146/ajhp150770

ISSN: 1535-2900

Place of Publication: England

PubMedID: 27605322

Accession Number: 27605322

Available at American Journal of Health-System Pharmacy - from EBSCO (MEDLINE Complete)

Available at American Journal of Health-System Pharmacy - from EBSCO (CINAHL Complete)

Abstract:PURPOSE: The use of a collaborative drug therapy agreement (CDTA) by oncology pharmacists in a comprehensive pain clinic is described.SUMMARY: Recognizing the complex clinical services required by patients with cancer, the Seattle Cancer Care Alliance began offering cancer pain management through a specialized pain service. Initially, the clinic was staffed by one attending physician; however, as the volume of patient referrals increased, the clinic expanded into an interprofessional team that includes physicians, advanced practice providers, nurses, and pharmacists. Through an extensive credentialing process and under the guidance of a CDTA, pharmacists in the pain clinic are able to evaluate patients, develop treatment plans, and prescribe pain medication therapies for oncology patients. By having pharmacists provide these services, the pain clinic can improve

medication dosing, ensure that medications are managed consistently, improve patients' quality of care, and save providers time by allowing tasks to be completed by appropriately trained ancillary staff. For cancer-related pain, the pharmacist, in conjunction with the attending provider, develops a pain medication plan following the principles of the World Health Organization's analgesic ladder. The pain clinic has implemented the routine use of several validated tools for screening and assessment of opioid risk as well as state guidelines for managing chronic opioid therapy. The pharmacists in the pain clinic also emphasize functional goals and improvement in functional status rather than complete relief of pain.CONCLUSION: As members of an interprofessional pain clinic team, oncology pharmacists use their specialized knowledge of cancer and pharmacotherapy to help manage and treat pain in complex cancer cases.

Institutions:

(Hammer KJ) University of Washington Medical Center/Seattle Cancer Care Alliance, Seattle, WA.; (Segal EM) University of Washington Medical Center/Seattle Cancer Care Alliance, Seattle, WA.; (Alwan L) University of Washington Medical Center/Seattle Cancer Care Alliance, Seattle, WA.; (Li S) University of Washington Medical Center/Seattle Cancer Care Alliance, Seattle, WA.; (Patel AM) University of Washington Medical Center/Seattle Cancer Care Alliance, Seattle, WA.; (Tran M) University of Washington Medical Center/Seattle Cancer Care Alliance, Seattle, WA.; (Marshall HM) University of Washington Medical Center/Seattle Cancer Care Alliance, Seattle, WA. helenm@seattlecca.org.
(Hammer KJ) University of Washington Medical Center/Seattle Cancer Care Alliance, Seattle, WA.; (Segal EM) University of Washington Medical Center/Seattle Cancer Care Alliance, Seattle, WA.; (Alwan L) University of Washington Medical Center/Seattle Cancer Care Alliance, Seattle, WA.; (Li S) University of Washington Medical Center/Seattle Cancer Care Alliance, Seattle, WA.; (Patel AM) University of Washington Medical Center/Seattle Cancer Care Alliance, Seattle, WA.; (Tran M) University of Washington Medical Center/Seattle Cancer Care Alliance, Seattle, WA.; (Marshall HM) University of Washington Medical Center/Seattle Cancer Care Alliance, Seattle, WA. helenm@seattlecca.org.
(Hammer KJ) University of Washington Medical Center/Seattle Cancer Care Alliance, Seattle, WA.; (Segal EM) University of Washington Medical Center/Seattle Cancer Care Alliance, Seattle, WA.; (Alwan L) University of Washington Medical Center/Seattle Cancer Care Alliance, Seattle, WA.; (Li S) University of Washington Medical Center/Seattle Cancer Care Alliance, Seattle, WA.; (Patel AM) University of Washington Medical Center/Seattle Cancer Care Alliance, Seattle, WA.; (Tran M) University of Washington Medical Center/Seattle Cancer Care Alliance, Seattle, WA.; (Marshall HM) University of Washington Medical Center/Seattle Cancer Care Alliance, Seattle, WA. helenm@seattlecca.org.
(Hammer KJ) University of Washington Medical Center/Seattle Cancer Care Alliance, Seattle, WA.; (Segal EM) University of Washington Medical Center/Seattle Cancer Care Alliance, Seattle, WA.; (Alwan L) University of Washington Medical Center/Seattle Cancer Care Alliance, Seattle, WA.; (Li S) University of Washington Medical Center/Seattle Cancer Care Alliance, Seattle, WA.; (Patel AM) University of Washington Medical Center/Seattle Cancer Care Alliance, Seattle, WA.; (Tran M) University of Washington Medical Center/Seattle Cancer Care Alliance, Seattle, WA.; (Marshall HM) University of Washington Medical Center/Seattle Cancer Care Alliance, Seattle, WA. helenm@seattlecca.org.

(Hammer KJ) University of Washington Medical Center/Seattle Cancer Care Alliance, Seattle, WA.; (Segal EM) University of Washington Medical Center/Seattle Cancer Care Alliance, Seattle, WA.; (Alwan L) University of Washington Medical Center/Seattle Cancer Care Alliance, Seattle, WA.; (Li S) University of Washington Medical Center/Seattle Cancer Care Alliance, Seattle, WA.; (Patel AM) University of Washington Medical Center/Seattle Cancer Care Alliance, Seattle, WA.; (Tran M) University of Washington Medical Center/Seattle Cancer Care Alliance, Seattle, WA.; (Marshall HM) University of Washington Medical Center/Seattle Cancer Care Alliance, Seattle, WA. helenm@seattlecca.org.
(Hammer KJ) University of Washington Medical Center/Seattle Cancer Care Alliance, Seattle, WA.; (Segal EM) University of Washington Medical Center/Seattle Cancer Care Alliance, Seattle, WA.; (Alwan L) University of Washington Medical Center/Seattle Cancer Care Alliance, Seattle, WA.; (Li S) University of Washington Medical Center/Seattle Cancer Care Alliance, Seattle, WA.; (Patel AM) University of Washington Medical Center/Seattle Cancer Care Alliance, Seattle, WA.; (Tran M) University of Washington Medical Center/Seattle Cancer Care Alliance, Seattle, WA.; (Marshall HM) University of Washington Medical Center/Seattle Cancer Care Alliance, Seattle, WA. helenm@seattlecca.org.
(Hammer KJ) University of Washington Medical Center/Seattle Cancer Care Alliance, Seattle, WA.; (Segal EM) University of Washington Medical Center/Seattle Cancer Care Alliance, Seattle, WA.; (Alwan L) University of Washington Medical Center/Seattle Cancer Care Alliance, Seattle, WA.; (Li S) University of Washington Medical Center/Seattle Cancer Care Alliance, Seattle, WA.; (Patel AM) University of Washington Medical Center/Seattle Cancer Care Alliance, Seattle, WA.; (Tran M) University of Washington Medical Center/Seattle Cancer Care Alliance, Seattle, WA.; (Marshall HM) University of Washington Medical Center/Seattle Cancer Care Alliance, Seattle, WA. helenm@seattlecca.org.
Database: PubMed

98. Integrated pain and palliative medicine model.

Author(s): Bhatnagar S; Gupta M

Source: Annals of palliative medicine; Jul 2016; vol. 5 (no. 3); p. 196-208

Publication Date: Jul 2016

Publication Type(s): Journal Article

DOI: http://dx.doi.org/10.21037/apm.2016.05.02

ISSN: 2224-5839

Place of Publication: China

PubMedID: 27334349

Accession Number: 27334349

Keywords: Subject Terms: Integration; cancer pain; intervention; medicine; model; pain; pain management; palliative care

Abstract: Pain is one of the most common, distressing and feared symptom among cancer and other patients in need of palliative care. An estimated 25% of cancer patients and 25 million people die in pain each year. Effective pain and symptom management are the core elements of palliative care which aims at reducing suffering and improving quality of life (QOL) throughout the course of illness starting

from diagnosis, in sync with curative treatments and at end of life. There is a prevailing shortage of manpower apt to deal with pain and providing cost-effective palliative care and with the rise of cancer, other chronic diseases and explosion of new life-prolonging therapeutic modalities, this 'Patient-pain and palliative physician' discrepancy is only going to increase, more so in developing countries. The need of the hour is to train all healthcare physicians and nurses especially those working in the field of chronic pain in principles of effective pain and symptom palliation, to integrate cancer pain and symptom management into existing pain management fellowships and to introduce a holistic pain and palliative care model at all levels of healthcare system. Simultaneously, of equal importance is to conduct research, evidence building and formulate policies and guidelines for meticulous symptom management among the diverse category of patients and diseases so as to have a personalized and individualistic approach to patient management. In this comprehensive review, we have pondered upon the need, advantages, barriers and recommendations to achieve ideal 'Integrated pain and palliative medicine' services, their equitable implementation and delivery to 'whomsoever in need of them'.

Institutions:
(Bhatnagar S) Institute Rotary Cancer Hospital, All India Institute of Medical Sciences, New Delhi, India. sushmabhatnagar1@gmail.com.; (Gupta M) Shri Guru Ram Rai Institute of Medical and Health Sciences, Shri Mahant Indiresh Hospital, Dehradun, Uttarakhand 248001, India.
(Bhatnagar S) Institute Rotary Cancer Hospital, All India Institute of Medical Sciences, New Delhi, India. sushmabhatnagar1@gmail.com.; (Gupta M) Shri Guru Ram Rai Institute of Medical and Health Sciences, Shri Mahant Indiresh Hospital, Dehradun, Uttarakhand 248001, India.
Database: PubMed

99. Opioid-induced Hallucinations: A Review of the Literature, Pathophysiology, Diagnosis, and Treatment.

Author(s): Sivanesan E; Gitlin MC; Candiotti KA

Source: Anesthesia and analgesia; 2016; vol. 123 (no. 4); p. 836-843

Publication Date: 2016

Publication Type(s): Journal Article; Review

DOI: http://dx.doi.org/10.1213/ANE.0000000000001417

ISSN: 1526-7598

Place of Publication: United States

PubMedID: 27258073

Accession Number: 27258073

Available at Anesthesia and analgesia - from Unpaywall

Abstract: Despite their association with multiple adverse effects, opioid prescription continues to increase. Opioid-induced hallucination is an uncommon yet significant adverse effect of opioid treatment. The practitioner may encounter patient reluctance to volunteer the occurrence of this phenomenon because of fears of being judged mentally unsound. The majority of the literature concerning opioid-induced hallucinations arises from treatment during end-of-life care and cancer pain. Because the rate of opioid prescriptions continues to increase in the population, the

rate of opioid-associated hallucinations may also conceivably increase. With a forecasted increase in the patient-to-physician ratio, opioid therapy is predicted to be provided by practitioners of varying backgrounds and medical specialties. Hence, knowledge of the pharmacology and potential adverse effects of these agents is required. This review seeks to increase awareness of this potential complication through a discussion of the literature, potential mechanisms of action, diagnosis, and treatment strategies.

Institutions:
(Sivanesan E) From the Department of Anesthesiology, Perioperative Medicine, and Pain Management, University of Miami Miller School of Medicine, Miami, Florida.
Database: PubMed

100. Nurse Attitude-Related Barriers to Effective Control of Cancer Pain among Iranian Nurses.

Author(s): Name N; Mohamadian R; Rahmani A; Fizollah-Zadeh H; Jabarzadeh F; Azadi A; Rostami H

Source: Asian Pacific journal of cancer prevention : APJCP; 2016; vol. 17 (no. 4); p. 2141-2144

Publication Date: 2016

Publication Type(s): Journal Article

DOI: http://dx.doi.org/10.7314/apjcp.2016.17.4.2141

ISSN: 2476-762X

Place of Publication: Thailand

PubMedID: 27221909

Accession Number: 27221909

Available at Asian Pacific journal of cancer prevention : APJCP - from Unpaywall

Abstract:BACKGROUND: Many cancer patients still experience pain worldwide. There are many barriers for effective control of cancer pain and many of these are related to health care providers. There is a need for further investigation of these barriers. The aim of this study was to investigate nurse-related barriers to control of cancer pain among Iranian nurses.MATERIALS AND METHODS: In this descriptive study 49 nurses from two hospitals affiliated to Tabriz and Ardebil Universities of Medical Sciences participated using a census sampling method. A demographic and profession related checklist and Barriers Questionnaire II (BQ-II) were used for data collection.RESULTS: The results showed negative attitudes of participants regarding control of cancer pain. Participants believed that cancer pain medications do not manage cancer pain at acceptable levels; patients may become addicted by using these drugs; cancer pain medications have many uncontrollable effects; and controlling cancer pain may distract the physicians from treating disease.CONCLUSIONS: Iranian nurses have negative attitudes toward pain control in cancer patients especially about effectiveness of pain medication and their side effects. Educational intervention to reduce these misconceptions is needed.

Institutions:
(Name N) Medical-Surgical Department, Nursing and Midwifery Faculty, Tabriz University of Medical Sciences, Tabriz, Iran E-mail : azad.rahmani@yahoo.com.

Database: PubMed

101. Cancer Pain Management Insights and Reality in Southeast Asia: Expert Perspectives From Six Countries.

Author(s): Javier FO; Irawan C; Mansor MB; Sriraj W; Tan KH; Thinh DHQ

Source: Journal of global oncology; Aug 2016; vol. 2 (no. 4); p. 235-243

Publication Date: Aug 2016

Publication Type(s): Journal Article

DOI: http://dx.doi.org/10.1200/JGO.2015.001859

ISSN: 2378-9506

Place of Publication: United States

PubMedID: 28717706

Accession Number: 28717706

Available at Journal of global oncology - from Unpaywall

Abstract: This expert opinion report examines the current realities of the cancer pain management landscape and the various factors that hinder optimal pain control in six countries in Southeast Asia, describes ongoing efforts to advance patient care, and discusses approaches for improving cancer pain management. Information was gathered from leading experts in the field of cancer pain management in each country through an initial meeting and subsequent e-mail discussions. Overall, there are vast disparities in cancer pain management practices and access to opioids in the Southeast Asian countries. The experts considered cancer pain as being generally undermanaged. Access to opioids is inadequate in most countries, and opioid use for analgesia remains inadequate in the region. Several system-, physician-, and patient-related barriers to adequate pain relief were identified, including widespread over-regulation of opioid use, shortage of trained health care workers, inadequacies in pain assessment and knowledge about managing pain, and widespread resistance among patients and physicians toward opioid treatment. According to the experts, many of the ongoing initiatives in the Southeast Asian countries are related to educating patients and physicians on cancer pain management and opioid use. Efforts to improve opioid availability and reduce regulatory barriers in the region are limited, and much work is still needed to improve the status of cancer pain management in the region. Enacting necessary change will require recognition of the unique needs and resources of each country and collaboration across interdisciplinary professional teams to improve cancer pain care in this region.

Institutions:
(Javier FO) , St Luke's Medical Center, Metro Manila, Philippines; , University of Indonesia, Jakarta, Indonesia; , University of Malaya, Kuala Lumpur, Malaysia; , Khon Kaen University, Khon Kaen, Thailand; , Singapore General Hospital, Singapore; and , Ho Chi Minh City Oncology Hospital, Ho Chi Minh City, Vietnam.; (Irawan C) , St Luke's Medical Center, Metro Manila, Philippines; , University of Indonesia, Jakarta, Indonesia; , University of Malaya, Kuala Lumpur, Malaysia; , Khon Kaen University, Khon Kaen, Thailand; , Singapore General Hospital, Singapore; and , Ho Chi Minh City Oncology Hospital, Ho Chi Minh City, Vietnam.; (Mansor MB) , St Luke's Medical Center, Metro Manila, Philippines; , University of

Indonesia, Jakarta, Indonesia; , University of Malaya, Kuala Lumpur, Malaysia; , Khon Kaen University, Khon Kaen, Thailand; , Singapore General Hospital, Singapore; and , Ho Chi Minh City Oncology Hospital, Ho Chi Minh City, Vietnam.; (Sriraj W) , St Luke's Medical Center, Metro Manila, Philippines; , University of Indonesia, Jakarta, Indonesia; , University of Malaya, Kuala Lumpur, Malaysia; , Khon Kaen University, Khon Kaen, Thailand; , Singapore General Hospital, Singapore; and , Ho Chi Minh City Oncology Hospital, Ho Chi Minh City, Vietnam.; (Tan KH) , St Luke's Medical Center, Metro Manila, Philippines; , University of Indonesia, Jakarta, Indonesia; , University of Malaya, Kuala Lumpur, Malaysia; , Khon Kaen University, Khon Kaen, Thailand; , Singapore General Hospital, Singapore; and , Ho Chi Minh City Oncology Hospital, Ho Chi Minh City, Vietnam.; (Thinh DHQ) , St Luke's Medical Center, Metro Manila, Philippines; , University of Indonesia, Jakarta, Indonesia; , University of Malaya, Kuala Lumpur, Malaysia; , Khon Kaen University, Khon Kaen, Thailand; , Singapore General Hospital, Singapore; and , Ho Chi Minh City Oncology Hospital, Ho Chi Minh City, Vietnam. (Javier FO) , St Luke's Medical Center, Metro Manila, Philippines; , University of Indonesia, Jakarta, Indonesia; , University of Malaya, Kuala Lumpur, Malaysia; , Khon Kaen University, Khon Kaen, Thailand; , Singapore General Hospital, Singapore; and , Ho Chi Minh City Oncology Hospital, Ho Chi Minh City, Vietnam.; (Irawan C) , St Luke's Medical Center, Metro Manila, Philippines; , University of Indonesia, Jakarta, Indonesia; , University of Malaya, Kuala Lumpur, Malaysia; , Khon Kaen University, Khon Kaen, Thailand; , Singapore General Hospital, Singapore; and , Ho Chi Minh City Oncology Hospital, Ho Chi Minh City, Vietnam.; (Mansor MB) , St Luke's Medical Center, Metro Manila, Philippines; , University of Indonesia, Jakarta, Indonesia; , University of Malaya, Kuala Lumpur, Malaysia; , Khon Kaen University, Khon Kaen, Thailand; , Singapore General Hospital, Singapore; and , Ho Chi Minh City Oncology Hospital, Ho Chi Minh City, Vietnam.; (Sriraj W) , St Luke's Medical Center, Metro Manila, Philippines; , University of Indonesia, Jakarta, Indonesia; , University of Malaya, Kuala Lumpur, Malaysia; , Khon Kaen University, Khon Kaen, Thailand; , Singapore General Hospital, Singapore; and , Ho Chi Minh City Oncology Hospital, Ho Chi Minh City, Vietnam.; (Tan KH) , St Luke's Medical Center, Metro Manila, Philippines; , University of Indonesia, Jakarta, Indonesia; , University of Malaya, Kuala Lumpur, Malaysia; , Khon Kaen University, Khon Kaen, Thailand; , Singapore General Hospital, Singapore; and , Ho Chi Minh City Oncology Hospital, Ho Chi Minh City, Vietnam.; (Thinh DHQ) , St Luke's Medical Center, Metro Manila, Philippines; , University of Indonesia, Jakarta, Indonesia; , University of Malaya, Kuala Lumpur, Malaysia; , Khon Kaen University, Khon Kaen, Thailand; , Singapore General Hospital, Singapore; and , Ho Chi Minh City Oncology Hospital, Ho Chi Minh City, Vietnam. (Javier FO) , St Luke's Medical Center, Metro Manila, Philippines; , University of Indonesia, Jakarta, Indonesia; , University of Malaya, Kuala Lumpur, Malaysia; , Khon Kaen University, Khon Kaen, Thailand; , Singapore General Hospital, Singapore; and , Ho Chi Minh City Oncology Hospital, Ho Chi Minh City, Vietnam.; (Irawan C) , St Luke's Medical Center, Metro Manila, Philippines; , University of Indonesia, Jakarta, Indonesia; , University of Malaya, Kuala Lumpur, Malaysia; , Khon Kaen University, Khon Kaen, Thailand; , Singapore General Hospital, Singapore; and , Ho Chi Minh City Oncology Hospital, Ho Chi Minh City, Vietnam.; (Mansor MB) , St Luke's Medical Center, Metro Manila, Philippines; , University of Indonesia, Jakarta, Indonesia; , University of Malaya, Kuala Lumpur, Malaysia; , Khon Kaen University, Khon Kaen, Thailand; , Singapore General Hospital,

Singapore; and , Ho Chi Minh City Oncology Hospital, Ho Chi Minh City, Vietnam.; (Sriraj W) , St Luke's Medical Center, Metro Manila, Philippines; , University of Indonesia, Jakarta, Indonesia; , University of Malaya, Kuala Lumpur, Malaysia; , Khon Kaen University, Khon Kaen, Thailand; , Singapore General Hospital, Singapore; and , Ho Chi Minh City Oncology Hospital, Ho Chi Minh City, Vietnam.; (Tan KH) , St Luke's Medical Center, Metro Manila, Philippines; , University of Indonesia, Jakarta, Indonesia; , University of Malaya, Kuala Lumpur, Malaysia; , Khon Kaen University, Khon Kaen, Thailand; , Singapore General Hospital, Singapore; and , Ho Chi Minh City Oncology Hospital, Ho Chi Minh City, Vietnam.; (Thinh DHQ) , St Luke's Medical Center, Metro Manila, Philippines; , University of Indonesia, Jakarta, Indonesia; , University of Malaya, Kuala Lumpur, Malaysia; , Khon Kaen University, Khon Kaen, Thailand; , Singapore General Hospital, Singapore; and , Ho Chi Minh City Oncology Hospital, Ho Chi Minh City, Vietnam. (Javier FO) , St Luke's Medical Center, Metro Manila, Philippines; , University of Indonesia, Jakarta, Indonesia; , University of Malaya, Kuala Lumpur, Malaysia; , Khon Kaen University, Khon Kaen, Thailand; , Singapore General Hospital, Singapore; and , Ho Chi Minh City Oncology Hospital, Ho Chi Minh City, Vietnam.; (Irawan C) , St Luke's Medical Center, Metro Manila, Philippines; , University of Indonesia, Jakarta, Indonesia; , University of Malaya, Kuala Lumpur, Malaysia; , Khon Kaen University, Khon Kaen, Thailand; , Singapore General Hospital, Singapore; and , Ho Chi Minh City Oncology Hospital, Ho Chi Minh City, Vietnam.; (Mansor MB) , St Luke's Medical Center, Metro Manila, Philippines; , University of Indonesia, Jakarta, Indonesia; , University of Malaya, Kuala Lumpur, Malaysia; , Khon Kaen University, Khon Kaen, Thailand; , Singapore General Hospital, Singapore; and , Ho Chi Minh City Oncology Hospital, Ho Chi Minh City, Vietnam.; (Sriraj W) , St Luke's Medical Center, Metro Manila, Philippines; , University of Indonesia, Jakarta, Indonesia; , University of Malaya, Kuala Lumpur, Malaysia; , Khon Kaen University, Khon Kaen, Thailand; , Singapore General Hospital, Singapore; and , Ho Chi Minh City Oncology Hospital, Ho Chi Minh City, Vietnam.; (Tan KH) , St Luke's Medical Center, Metro Manila, Philippines; , University of Indonesia, Jakarta, Indonesia; , University of Malaya, Kuala Lumpur, Malaysia; , Khon Kaen University, Khon Kaen, Thailand; , Singapore General Hospital, Singapore; and , Ho Chi Minh City Oncology Hospital, Ho Chi Minh City, Vietnam.; (Thinh DHQ) , St Luke's Medical Center, Metro Manila, Philippines; , University of Indonesia, Jakarta, Indonesia; , University of Malaya, Kuala Lumpur, Malaysia; , Khon Kaen University, Khon Kaen, Thailand; , Singapore General Hospital, Singapore; and , Ho Chi Minh City Oncology Hospital, Ho Chi Minh City, Vietnam. (Javier FO) , St Luke's Medical Center, Metro Manila, Philippines; , University of Indonesia, Jakarta, Indonesia; , University of Malaya, Kuala Lumpur, Malaysia; , Khon Kaen University, Khon Kaen, Thailand; , Singapore General Hospital, Singapore; and , Ho Chi Minh City Oncology Hospital, Ho Chi Minh City, Vietnam.; (Irawan C) , St Luke's Medical Center, Metro Manila, Philippines; , University of Indonesia, Jakarta, Indonesia; , University of Malaya, Kuala Lumpur, Malaysia; , Khon Kaen University, Khon Kaen, Thailand; , Singapore General Hospital, Singapore; and , Ho Chi Minh City Oncology Hospital, Ho Chi Minh City, Vietnam.; (Mansor MB) , St Luke's Medical Center, Metro Manila, Philippines; , University of Indonesia, Jakarta, Indonesia; , University of Malaya, Kuala Lumpur, Malaysia; , Khon Kaen University, Khon Kaen, Thailand; , Singapore General Hospital, Singapore; and , Ho Chi Minh City Oncology Hospital, Ho Chi Minh City, Vietnam.; (Sriraj W) , St Luke's Medical Center, Metro Manila, Philippines; , University of

Indonesia, Jakarta, Indonesia; , University of Malaya, Kuala Lumpur, Malaysia; , Khon Kaen University, Khon Kaen, Thailand; , Singapore General Hospital, Singapore; and , Ho Chi Minh City Oncology Hospital, Ho Chi Minh City, Vietnam.; (Tan KH) , St Luke's Medical Center, Metro Manila, Philippines; , University of Indonesia, Jakarta, Indonesia; , University of Malaya, Kuala Lumpur, Malaysia; , Khon Kaen University, Khon Kaen, Thailand; , Singapore General Hospital, Singapore; and , Ho Chi Minh City Oncology Hospital, Ho Chi Minh City, Vietnam.; (Thinh DHQ) , St Luke's Medical Center, Metro Manila, Philippines; , University of Indonesia, Jakarta, Indonesia; , University of Malaya, Kuala Lumpur, Malaysia; , Khon Kaen University, Khon Kaen, Thailand; , Singapore General Hospital, Singapore; and , Ho Chi Minh City Oncology Hospital, Ho Chi Minh City, Vietnam. (Javier FO) , St Luke's Medical Center, Metro Manila, Philippines; , University of Indonesia, Jakarta, Indonesia; , University of Malaya, Kuala Lumpur, Malaysia; , Khon Kaen University, Khon Kaen, Thailand; , Singapore General Hospital, Singapore; and , Ho Chi Minh City Oncology Hospital, Ho Chi Minh City, Vietnam.; (Irawan C) , St Luke's Medical Center, Metro Manila, Philippines; , University of Indonesia, Jakarta, Indonesia; , University of Malaya, Kuala Lumpur, Malaysia; , Khon Kaen University, Khon Kaen, Thailand; , Singapore General Hospital, Singapore; and , Ho Chi Minh City Oncology Hospital, Ho Chi Minh City, Vietnam.; (Mansor MB) , St Luke's Medical Center, Metro Manila, Philippines; , University of Indonesia, Jakarta, Indonesia; , University of Malaya, Kuala Lumpur, Malaysia; , Khon Kaen University, Khon Kaen, Thailand; , Singapore General Hospital, Singapore; and , Ho Chi Minh City Oncology Hospital, Ho Chi Minh City, Vietnam.; (Sriraj W) , St Luke's Medical Center, Metro Manila, Philippines; , University of Indonesia, Jakarta, Indonesia; , University of Malaya, Kuala Lumpur, Malaysia; , Khon Kaen University, Khon Kaen, Thailand; , Singapore General Hospital, Singapore; and , Ho Chi Minh City Oncology Hospital, Ho Chi Minh City, Vietnam.; (Tan KH) , St Luke's Medical Center, Metro Manila, Philippines; , University of Indonesia, Jakarta, Indonesia; , University of Malaya, Kuala Lumpur, Malaysia; , Khon Kaen University, Khon Kaen, Thailand; , Singapore General Hospital, Singapore; and , Ho Chi Minh City Oncology Hospital, Ho Chi Minh City, Vietnam.; (Thinh DHQ) , St Luke's Medical Center, Metro Manila, Philippines; , University of Indonesia, Jakarta, Indonesia; , University of Malaya, Kuala Lumpur, Malaysia; , Khon Kaen University, Khon Kaen, Thailand; , Singapore General Hospital, Singapore; and , Ho Chi Minh City Oncology Hospital, Ho Chi Minh City, Vietnam.
Database: PubMed

102. Performance and quality indicators for the management of non-cancer chronic pain: a scoping review protocol.

Author(s): Zidarov D; Visca R; Gogovor A; Ahmed S

Source: BMJ open; Feb 2016; vol. 6 (no. 2); p. e010487

Publication Date: Feb 2016

Publication Type(s): Journal Article; Research Support, Non-U.S. Gov't; Review

DOI: http://dx.doi.org/10.1136/bmjopen-2015-010487

ISSN: 2044-6055

Place of Publication: England

PubMedID: 26895987

Accession Number: 26895987

Available at BMJ open - from Europe PubMed Central - Open Access

Available at BMJ open - from HighWire - Free Full Text

Available at BMJ open - from ProQuest (Health Research Premium) - NHS Version

Available at BMJ open - from Unpaywall

Keywords: Subject Terms: Non-cancer chronic pain; indicators; performance; quality of care; scoping review

Abstract:INTRODUCTION: Chronic pain is a public health problem of epidemic proportion in most countries with important physical, psychological, social and economic consequences. The management of chronic pain is complex and requires an integrated network approach between all levels of the healthcare system and the involvement of several health professionals from different disciplines. Measuring the performance of organisations that provide care to individuals with chronic pain is essential to improve quality of care and requires the use of relevant performance and quality indicators. A scoping review methodology will be used to synthesise the evidence on performance and quality indicators developed for non-cancer chronic pain management across the continuum of care.METHODS AND ANALYSIS: The following electronic databases will be searched from 2000 onwards: Cochrane Effective Practice and Organisation of Care (EPOC) Review Group Specialised Register; Cochrane Library; EMBASE; PubMed; CINAHL; PsycINFO; ProQuest Dissertations and Theses. All types of studies will be included if these are concerned with performance or quality indicators in adults with chronic non-cancer pain. In addition, searches will be conducted on provincial, national and international health organisations as well as health professional and scientific associations' websites. A qualitative descriptive approach will be used to describe characteristics of each indicator. All identified indicators will be classified according to dimensions covered by Donabedian and the Triple Aim frameworks.ETHICS AND DISSEMINATION: The scoping review findings will inform the development of a performance measurement system comprising a list of performance indicators with their level of evidence which can be used by stakeholders to evaluate the quality of care for individuals with chronic non-cancer pain at the patient, institutional and system level. The results will be disseminated via several knowledge translation strategies, including 2 stakeholder meetings, publication and presentation at conferences.

Institutions:
(Zidarov D) School of Public Health, University of Montreal, Montreal, Quebec, Canada Centre de Recherche Interdisciplinaire en Réadaptation du Montréal Métropolitain, Montreal, Quebec, Canada.; (Visca R) Centre of Expertise in Chronic Pain, Réseau Universitaire Intégré de Santé (RUIS) McGill, Montreal, Quebec, Canada.; (Gogovor A) Faculty of Medicine, School of Physical and Occupational Therapy, Epidemiology, Biostatistics, and Occupational Health, McGill University, Montreal, Quebec, Canada.; (Ahmed S) Centre de Recherche Interdisciplinaire en Réadaptation du Montréal Métropolitain, Montreal, Quebec, Canada Centre of Expertise in Chronic Pain, Réseau Universitaire Intégré de Santé (RUIS) McGill, Montreal, Quebec, Canada Faculty of Medicine, School of Physical and Occupational Therapy, Epidemiology, Biostatistics, and Occupational Health, McGill University, Montreal, Quebec, Canada Department of Clinical Epidemiology, McGill University Health Center, Montreal, Quebec, Canada.

(Zidarov D) School of Public Health, University of Montreal, Montreal, Quebec, Canada Centre de Recherche Interdisciplinaire en Réadaptation du Montréal Métropolitain, Montreal, Quebec, Canada.; (Visca R) Centre of Expertise in Chronic Pain, Réseau Universitaire Intégré de Santé (RUIS) McGill, Montreal, Quebec, Canada.; (Gogovor A) Faculty of Medicine, School of Physical and Occupational Therapy, Epidemiology, Biostatistics, and Occupational Health, McGill University, Montreal, Quebec, Canada.; (Ahmed S) Centre de Recherche Interdisciplinaire en Réadaptation du Montréal Métropolitain, Montreal, Quebec, Canada Centre of Expertise in Chronic Pain, Réseau Universitaire Intégré de Santé (RUIS) McGill, Montreal, Quebec, Canada Faculty of Medicine, School of Physical and Occupational Therapy, Epidemiology, Biostatistics, and Occupational Health, McGill University, Montreal, Quebec, Canada Department of Clinical Epidemiology, McGill University Health Center, Montreal, Quebec, Canada.
(Zidarov D) School of Public Health, University of Montreal, Montreal, Quebec, Canada Centre de Recherche Interdisciplinaire en Réadaptation du Montréal Métropolitain, Montreal, Quebec, Canada.; (Visca R) Centre of Expertise in Chronic Pain, Réseau Universitaire Intégré de Santé (RUIS) McGill, Montreal, Quebec, Canada.; (Gogovor A) Faculty of Medicine, School of Physical and Occupational Therapy, Epidemiology, Biostatistics, and Occupational Health, McGill University, Montreal, Quebec, Canada.; (Ahmed S) Centre de Recherche Interdisciplinaire en Réadaptation du Montréal Métropolitain, Montreal, Quebec, Canada Centre of Expertise in Chronic Pain, Réseau Universitaire Intégré de Santé (RUIS) McGill, Montreal, Quebec, Canada Faculty of Medicine, School of Physical and Occupational Therapy, Epidemiology, Biostatistics, and Occupational Health, McGill University, Montreal, Quebec, Canada Department of Clinical Epidemiology, McGill University Health Center, Montreal, Quebec, Canada.
(Zidarov D) School of Public Health, University of Montreal, Montreal, Quebec, Canada Centre de Recherche Interdisciplinaire en Réadaptation du Montréal Métropolitain, Montreal, Quebec, Canada.; (Visca R) Centre of Expertise in Chronic Pain, Réseau Universitaire Intégré de Santé (RUIS) McGill, Montreal, Quebec, Canada.; (Gogovor A) Faculty of Medicine, School of Physical and Occupational Therapy, Epidemiology, Biostatistics, and Occupational Health, McGill University, Montreal, Quebec, Canada.; (Ahmed S) Centre de Recherche Interdisciplinaire en Réadaptation du Montréal Métropolitain, Montreal, Quebec, Canada Centre of Expertise in Chronic Pain, Réseau Universitaire Intégré de Santé (RUIS) McGill, Montreal, Quebec, Canada Faculty of Medicine, School of Physical and Occupational Therapy, Epidemiology, Biostatistics, and Occupational Health, McGill University, Montreal, Quebec, Canada Department of Clinical Epidemiology, McGill University Health Center, Montreal, Quebec, Canada.
Database: PubMed

103. Medical use of cannabis products: Lessons to be learned from Israel and Canada.

Author(s): Ablin J; Ste-Marie PA; Schäfer M; Häuser W; Fitzcharles MA

Source: Schmerz (Berlin, Germany); Feb 2016; vol. 30 (no. 1); p. 3-13

Publication Date: Feb 2016

Publication Type(s): Comparative Study; Journal Article

DOI: http://dx.doi.org/10.1007/s00482-015-0083-4

ISSN: 1432-2129

Place of Publication: Germany

PubMedID: 26767992

Accession Number: 26767992

Available at Schmerz (Berlin, Germany) - from EBSCO (MEDLINE Complete)

Keywords: Subject Terms: *Cannabinoids; *Herbal cannabis; *Israel–Canada; *Medical use; *Regulatory framework

Abstract:INTRODUCTION: The German government intends to reduce the barriers for the medical use of cannabis products. A discussion on the indications and contraindications of the medical use of cannabis and on the changes of the regulatory framework has already begun in Germany. It is useful to draw from the experiences of other countries with a more liberal medical use of cannabis.METHODS: The Israeli and Canadian experience is outlined by physicians who have been charged with expertise on the medical use of cannabis by their jurisdiction.RESULTS: In Israel, only the plant-based cannabinoid nabiximol (mixture of tetrahydrocannabinol/cannabidiol) can be prescribed for spasticity/chronic pain in multiple sclerosis and for cancer pain. The costs of nabiximole are reimbursed by some, but not by all health maintenance organizations. The medical use of marijuana is permitted; however, it is strictly regulated by the government. Selected companies are allowed to produce marijuana for medical use, and only certain physicians are licensed to prescribe marijuana as a therapeutic drug for specific indications such as chronic neuropathic, and cancer pain, inflammatory bowel diseases, or posttraumatic stress disorder if conventional treatments have failed. The costs of marijuana are not reimbursed by health insurance companies. In Canada, synthetic cannabinoids and the plant-based (nabiximol) are licensed for neuropathic and cancer pain, HIV-related anorexia and chemotherapy-associate nausea. The costs of these synthetic cannabinoids are covered by health insurance companies. The medical use of marijuana as a treatment option is allowed for individual patients suffering from any medical condition when authorized by a medical practitioner or nurse. Licensed producers are the only source for patients to newly access medical cannabis, although those with previous permission to grow may continue cultivation at the present time. The costs of marijuana are not reimbursed by health insurance companies. There are multiple contraindications for the medical use of cannabis products in both countries.CONCLUSIONS: The use of standardized, synthetic, and plant-based cannabis products should be allowed in Germany for defined medical conditions when high-level evidence of efficacy and safety exists. The costs should be reimbursed by the health insurance companies. Contraindications for the medical use of cannabis should be defined. Growing marijuana by patients for their medical use should not be allowed.

Institutions:
(Ablin J) Institute of Rheumatology, Tel Aviv Sourasky Medical Center and Sackler School of Medicine, Tel Aviv University, Tel Aviv, Israel.; (Ste-Marie PA) Division of Rheumatology, McGill University, Montreal, Quebec, Canada.; (Schäfer M) Department of Anesthesiology and Intensive Care Medicine, Charité University, Berlin Campus Virchow Klinikum, Berlin, Germany.; (Häuser W) Klinikum Saarbrücken gGmbH, Innere Medizin 1, Winterberg 1, 66119, Saarbrücken, Germany. whaeuser@klinikum-saarbruecken.de.; (Fitzcharles MA) Division of Rheumatology, McGill University, Montreal, Quebec, Canada.

(Ablin J) Institute of Rheumatology, Tel Aviv Sourasky Medical Center and Sackler School of Medicine, Tel Aviv University, Tel Aviv, Israel.; (Ste-Marie PA) Division of Rheumatology, McGill University, Montreal, Quebec, Canada.; (Schäfer M) Department of Anesthesiology and Intensive Care Medicine, Charité University, Berlin Campus Virchow Klinikum, Berlin, Germany.; (Häuser W) Klinikum Saarbrücken gGmbH, Innere Medizin 1, Winterberg 1, 66119, Saarbrücken, Germany. whaeuser@klinikum-saarbruecken.de.; (Fitzcharles MA) Division of Rheumatology, McGill University, Montreal, Quebec, Canada.
(Ablin J) Institute of Rheumatology, Tel Aviv Sourasky Medical Center and Sackler School of Medicine, Tel Aviv University, Tel Aviv, Israel.; (Ste-Marie PA) Division of Rheumatology, McGill University, Montreal, Quebec, Canada.; (Schäfer M) Department of Anesthesiology and Intensive Care Medicine, Charité University, Berlin Campus Virchow Klinikum, Berlin, Germany.; (Häuser W) Klinikum Saarbrücken gGmbH, Innere Medizin 1, Winterberg 1, 66119, Saarbrücken, Germany. whaeuser@klinikum-saarbruecken.de.; (Fitzcharles MA) Division of Rheumatology, McGill University, Montreal, Quebec, Canada.
(Ablin J) Institute of Rheumatology, Tel Aviv Sourasky Medical Center and Sackler School of Medicine, Tel Aviv University, Tel Aviv, Israel.; (Ste-Marie PA) Division of Rheumatology, McGill University, Montreal, Quebec, Canada.; (Schäfer M) Department of Anesthesiology and Intensive Care Medicine, Charité University, Berlin Campus Virchow Klinikum, Berlin, Germany.; (Häuser W) Klinikum Saarbrücken gGmbH, Innere Medizin 1, Winterberg 1, 66119, Saarbrücken, Germany. whaeuser@klinikum-saarbruecken.de.; (Fitzcharles MA) Division of Rheumatology, McGill University, Montreal, Quebec, Canada.
(Ablin J) Institute of Rheumatology, Tel Aviv Sourasky Medical Center and Sackler School of Medicine, Tel Aviv University, Tel Aviv, Israel.; (Ste-Marie PA) Division of Rheumatology, McGill University, Montreal, Quebec, Canada.; (Schäfer M) Department of Anesthesiology and Intensive Care Medicine, Charité University, Berlin Campus Virchow Klinikum, Berlin, Germany.; (Häuser W) Klinikum Saarbrücken gGmbH, Innere Medizin 1, Winterberg 1, 66119, Saarbrücken, Germany. whaeuser@klinikum-saarbruecken.de.; (Fitzcharles MA) Division of Rheumatology, McGill University, Montreal, Quebec, Canada.
Database: PubMed

104. What to Do, and What Not to Do, When Diagnosing and Treating Breakthrough Cancer Pain (BTcP): Expert Opinion.

Author(s): Working Group Nientemale DEI; Vellucci R; Fanelli G; Pannuti R; Peruselli C; Adamo S; Alongi G; Amato F; Consoletti L; Lamarca L; Liguori S; Lo Presti C; Maione A; Mameli S; Marinangeli F; Marulli S; Minotti V; Miotti D; Montanari L; Moruzzi G; Palermo S; Parolini M; Poli P; Tirelli W; Valle A; Romualdi P

Source: Drugs; Mar 2016; vol. 76 (no. 3); p. 315-330

Publication Date: Mar 2016

Publication Type(s): Journal Article; Review

DOI: http://dx.doi.org/10.1007/s40265-015-0519-2

ISSN: 1179-1950

Place of Publication: New Zealand

PubMedID: 26755179

Accession Number: 26755179

Available at Drugs - from ProQuest (Health Research Premium) - NHS Version

Available at Drugs - from ProQuest (MEDLINE with Full Text) - NHS Version

Available at Drugs - from Unpaywall

Abstract: Clinical management of breakthrough cancer pain (BTcP) is still not satisfactory despite the availability of effective pharmacological agents. This is in part linked to the lack of clarity regarding certain essential aspects of BTcP, including terminology, definition, epidemiology and assessment. Other barriers to effective management include a widespread prejudice among doctors and patients concerning the use of opioids, and inadequate assessment of pain severity, resulting in the prescription of ineffective drugs or doses. This review presents an overview of the appropriate and inappropriate actions to take in the diagnosis and treatment of BTcP, as determined by a panel of experts in the field. The ultimate aim is to provide a practical contribution to the unresolved issues in the management of BTcP. Five 'things to do' and five 'things not to do' in the diagnosis and treatment of BTcP are proposed, and evidence supporting said recommendations are described. It is the duty of all healthcare workers involved in managing cancer patients to be mindful of the possibility of BTcP occurrence and not to underestimate its severity. It is vital that all the necessary steps are carried out to establish an accurate and timely diagnosis, principally by establishing effective communication with the patient, the main information source. It is crucial that BTcP is treated with an effective pharmacological regimen and drug(s), dose and administration route prescribed are designed to suit the particular type of pain and importantly the individual needs of the patient.

Institutions:
(Working Group Nientemale DEI) Working Group Nientemale DEI; (Vellucci R) SOD Cure Palliative e Terapia del Dolore, Ospedale Universitario Careggi, Florence, Italy. renato.vellucci@gmail.com.; (Fanelli G) SC Anestesia, Rianimazione e Terapia Antalgica, Azienda Ospedaliero-Universitaria di Parma, Parma, Italy.; (Pannuti R) Fondazione ANT Italia Onlus, Andria, Italy.; (Peruselli C) SC Cure Palliative, Ospedale di Biella, Ponderano, BI, Italy.; (Adamo S) UO Terapia del Dolore, ARNAS Civico di Palermo, Palermo, Italy.; (Alongi G) Hospice e Cure Palliative, ASP 1di Agrigento, Agrigento, Italy.; (Amato F) UOC Terapia del Dolore e Cure Palliative, Azienda ospedaliera di Cosenza, Cosenza, Italy.; (Consoletti L) Struttura di Medicina del Dolore, Ospedale Universitario "Ospedali Riuniti", Foggia, Italy.; (Lamarca L) UOS Cure Palliative e Terapia Antalgica, Azienda ULSS N. 10 "Veneto Orientale", San Donà di Piave, VE, Italy.; (Liguori S) USC Cure Palliative Terapia del Dolore, Azienda Ospedaliera Papa Giovanni XXIII, Bergamo, Italy.; (Lo Presti C) UOD Terapia del Dolore e Cure Palliative, ACO San Filippo Neri, ASLRME, Rome, Italy.; (Maione A) Terapia antalgica e Cure Palliative, Presidio Ospedaliero "S. Maria della Pietà", Nola, NA, Italy.; (Mameli S) SC Terapia del Dolore, Presidio Ospedaliero "A. Businco", Cagliari, Italy.; (Marinangeli F) Scuola di Specializzazione di Anestesia, Rianimazione e Terapia Intensiva, Università dell'Aquila, L'Aquila, Italy.; (Marulli S) OC Anestesia, Rianimazione e Terapia Iperbarica, UOS-I Gruppo Operatorio, Ospedale "Vito Fazzi", Lecce, Italy.; (Minotti V) SC Oncologia Medica, Azienda Ospedaliera "S.M. della Misericordia", Perugia, Italy.; (Miotti D) UO Cure Palliative e Terapia del Dolore, Fondazione Salvatore Maugeri-IRCCS, Pavia, Italy.; (Montanari L) UO Semplice Cure Palliative, Ravenna, Italy.; (Moruzzi G) UOS Hospice, Azienda Sanitaria Provinciale di Siracusa, Siracuse, Italy.; (Palermo S) UOC Terapia Antalgica, IRCCS San Martino-IST, Genoa, Italy.; (Parolini M) UOC Anestesia e

Rianimazione B, Azienda Universitaria integrata di Verona, Verona, Italy.; (Poli P) UO Terapia del Dolore, Azienda Ospedaliero-Universitaria Pisana, Pisa, Italy.; (Tirelli W) Centro di Terapia del Dolore, Hospice "Fondazione Roma Sanità", Rome, Italy.; (Valle A) Fondazione FARO, Turin, Italy.; (Romualdi P) Dipartimento di Farmacia e Biotecnologie, Alma mater studiorum, Università di Bologna, Bologna, Italy. (Working Group Nientemale DEI) Working Group Nientemale DEI; (Vellucci R) SOD Cure Palliative e Terapia del Dolore, Ospedale Universitario Careggi, Florence, Italy. renato.vellucci@gmail.com.; (Fanelli G) SC Anestesia, Rianimazione e Terapia Antalgica, Azienda Ospedaliero-Universitaria di Parma, Parma, Italy.; (Pannuti R) Fondazione ANT Italia Onlus, Andria, Italy.; (Peruselli C) SC Cure Palliative, Ospedale di Biella, Ponderano, BI, Italy.; (Adamo S) UO Terapia del Dolore, ARNAS Civico di Palermo, Palermo, Italy.; (Alongi G) Hospice e Cure Palliative, ASP 1di Agrigento, Agrigento, Italy.; (Amato F) UOC Terapia del Dolore e Cure Palliative, Azienda ospedaliera di Cosenza, Cosenza, Italy.; (Consoletti L) Struttura di Medicina del Dolore, Ospedale Universitario "Ospedali Riuniti", Foggia, Italy.; (Lamarca L) UOS Cure Palliative e Terapia Antalgica, Azienda ULSS N. 10 "Veneto Orientale", San Donà di Piave, VE, Italy.; (Liguori S) USC Cure Palliative Terapia del Dolore, Azienda Ospedaliera Papa Giovanni XXIII, Bergamo, Italy.; (Lo Presti C) UOD Terapia del Dolore e Cure Palliative, ACO San Filippo Neri, ASLRME, Rome, Italy.; (Maione A) Terapia antalgica e Cure Palliative, Presidio Ospedaliero "S. Maria della Pietà", Nola, NA, Italy.; (Mameli S) SC Terapia del Dolore, Presidio Ospedaliero "A. Businco", Cagliari, Italy.; (Marinangeli F) Scuola di Specializzazione di Anestesia, Rianimazione e Terapia Intensiva, Università dell'Aquila, L'Aquila, Italy.; (Marulli S) OC Anestesia, Rianimazione e Terapia Iperbarica, UOS-I Gruppo Operatorio, Ospedale "Vito Fazzi", Lecce, Italy.; (Minotti V) SC Oncologia Medica, Azienda Ospedaliera "S.M. della Misericordia", Perugia, Italy.; (Miotti D) UO Cure Palliative e Terapia del Dolore, Fondazione Salvatore Maugeri-IRCCS, Pavia, Italy.; (Montanari L) UO Semplice Cure Palliative, Ravenna, Italy.; (Moruzzi G) UOS Hospice, Azienda Sanitaria Provinciale di Siracusa, Siracuse, Italy.; (Palermo S) UOC Terapia Antalgica, IRCCS San Martino-IST, Genoa, Italy.; (Parolini M) UOC Anestesia e
Rianimazione B, Azienda Universitaria integrata di Verona, Verona, Italy.; (Poli P) UO Terapia del Dolore, Azienda Ospedaliero-Universitaria Pisana, Pisa, Italy.; (Tirelli W) Centro di Terapia del Dolore, Hospice "Fondazione Roma Sanità", Rome, Italy.; (Valle A) Fondazione FARO, Turin, Italy.; (Romualdi P) Dipartimento di Farmacia e Biotecnologie, Alma mater studiorum, Università di Bologna, Bologna, Italy. (Working Group Nientemale DEI) Working Group Nientemale DEI; (Vellucci R) SOD Cure Palliative e Terapia del Dolore, Ospedale Universitario Careggi, Florence, Italy. renato.vellucci@gmail.com.; (Fanelli G) SC Anestesia, Rianimazione e Terapia Antalgica, Azienda Ospedaliero-Universitaria di Parma, Parma, Italy.; (Pannuti R) Fondazione ANT Italia Onlus, Andria, Italy.; (Peruselli C) SC Cure Palliative, Ospedale di Biella, Ponderano, BI, Italy.; (Adamo S) UO Terapia del Dolore, ARNAS Civico di Palermo, Palermo, Italy.; (Alongi G) Hospice e Cure Palliative, ASP 1di Agrigento, Agrigento, Italy.; (Amato F) UOC Terapia del Dolore e Cure Palliative, Azienda ospedaliera di Cosenza, Cosenza, Italy.; (Consoletti L) Struttura di Medicina del Dolore, Ospedale Universitario "Ospedali Riuniti", Foggia, Italy.; (Lamarca L) UOS Cure Palliative e Terapia Antalgica, Azienda ULSS N. 10 "Veneto Orientale", San Donà di Piave, VE, Italy.; (Liguori S) USC Cure Palliative Terapia del Dolore, Azienda Ospedaliera Papa Giovanni XXIII, Bergamo, Italy.; (Lo Presti C) UOD Terapia del Dolore e Cure Palliative, ACO San Filippo Neri, ASLRME, Rome, Italy.; (Maione A) Terapia antalgica e Cure Palliative, Presidio Ospedaliero "S. Maria della

Pietà", Nola, NA, Italy.; (Mameli S) SC Terapia del Dolore, Presidio Ospedaliero "A. Businco", Cagliari, Italy.; (Marinangeli F) Scuola di Specializzazione di Anestesia, Rianimazione e Terapia Intensiva, Università dell'Aquila, L'Aquila, Italy.; (Marulli S) OC Anestesia, Rianimazione e Terapia Iperbarica, UOS-I Gruppo Operatorio, Ospedale "Vito Fazzi", Lecce, Italy.; (Minotti V) SC Oncologia Medica, Azienda Ospedaliera "S.M. della Misericordia", Perugia, Italy.; (Miotti D) UO Cure Palliative e Terapia del Dolore, Fondazione Salvatore Maugeri-IRCCS, Pavia, Italy.; (Montanari L) UO Semplice Cure Palliative, Ravenna, Italy.; (Moruzzi G) UOS Hospice, Azienda Sanitaria Provinciale di Siracusa, Siracuse, Italy.; (Palermo S) UOC Terapia Antalgica, IRCCS San Martino-IST, Genoa, Italy.; (Parolini M) UOC Anestesia e Rianimazione B, Azienda Universitaria integrata di Verona, Verona, Italy.; (Poli P) UO Terapia del Dolore, Azienda Ospedaliero-Universitaria Pisana, Pisa, Italy.; (Tirelli W) Centro di Terapia del Dolore, Hospice "Fondazione Roma Sanità", Rome, Italy.; (Valle A) Fondazione FARO, Turin, Italy.; (Romualdi P) Dipartimento di Farmacia e Biotecnologie, Alma mater studiorum, Università di Bologna, Bologna, Italy. (Working Group Nientemale DEI) Working Group Nientemale DEI; (Vellucci R) SOD Cure Palliative e Terapia del Dolore, Ospedale Universitario Careggi, Florence, Italy. renato.vellucci@gmail.com.; (Fanelli G) SC Anestesia, Rianimazione e Terapia Antalgica, Azienda Ospedaliero-Universitaria di Parma, Parma, Italy.; (Pannuti R) Fondazione ANT Italia Onlus, Andria, Italy.; (Peruselli C) SC Cure Palliative, Ospedale di Biella, Ponderano, BI, Italy.; (Adamo S) UO Terapia del Dolore, ARNAS Civico di Palermo, Palermo, Italy.; (Alongi G) Hospice e Cure Palliative, ASP 1di Agrigento, Agrigento, Italy.; (Amato F) UOC Terapia del Dolore e Cure Palliative, Azienda ospedaliera di Cosenza, Cosenza, Italy.; (Consoletti L) Struttura di Medicina del Dolore, Ospedale Universitario "Ospedali Riuniti", Foggia, Italy.; (Lamarca L) UOS Cure Palliative e Terapia Antalgica, Azienda ULSS N. 10 "Veneto Orientale", San Donà di Piave, VE, Italy.; (Liguori S) USC Cure Palliative Terapia del Dolore, Azienda Ospedaliera Papa Giovanni XXIII, Bergamo, Italy.; (Lo Presti C) UOD Terapia del Dolore e Cure Palliative, ACO San Filippo Neri, ASLRME, Rome, Italy.; (Maione A) Terapia antalgica e Cure Palliative, Presidio Ospedaliero "S. Maria della
Pietà", Nola, NA, Italy.; (Mameli S) SC Terapia del Dolore, Presidio Ospedaliero "A. Businco", Cagliari, Italy.; (Marinangeli F) Scuola di Specializzazione di Anestesia, Rianimazione e Terapia Intensiva, Università dell'Aquila, L'Aquila, Italy.; (Marulli S) OC Anestesia, Rianimazione e Terapia Iperbarica, UOS-I Gruppo Operatorio, Ospedale "Vito Fazzi", Lecce, Italy.; (Minotti V) SC Oncologia Medica, Azienda Ospedaliera "S.M. della Misericordia", Perugia, Italy.; (Miotti D) UO Cure Palliative e Terapia del Dolore, Fondazione Salvatore Maugeri-IRCCS, Pavia, Italy.; (Montanari L) UO Semplice Cure Palliative, Ravenna, Italy.; (Moruzzi G) UOS Hospice, Azienda Sanitaria Provinciale di Siracusa, Siracuse, Italy.; (Palermo S) UOC Terapia Antalgica, IRCCS San Martino-IST, Genoa, Italy.; (Parolini M) UOC Anestesia e Rianimazione B, Azienda Universitaria integrata di Verona, Verona, Italy.; (Poli P) UO Terapia del Dolore, Azienda Ospedaliero-Universitaria Pisana, Pisa, Italy.; (Tirelli W) Centro di Terapia del Dolore, Hospice "Fondazione Roma Sanità", Rome, Italy.; (Valle A) Fondazione FARO, Turin, Italy.; (Romualdi P) Dipartimento di Farmacia e Biotecnologie, Alma mater studiorum, Università di Bologna, Bologna, Italy. (Working Group Nientemale DEI) Working Group Nientemale DEI; (Vellucci R) SOD Cure Palliative e Terapia del Dolore, Ospedale Universitario Careggi, Florence, Italy. renato.vellucci@gmail.com.; (Fanelli G) SC Anestesia, Rianimazione e Terapia Antalgica, Azienda Ospedaliero-Universitaria di Parma, Parma, Italy.; (Pannuti R) Fondazione ANT Italia Onlus, Andria, Italy.; (Peruselli C) SC Cure Palliative,

Ospedale di Biella, Ponderano, BI, Italy.; (Adamo S) UO Terapia del Dolore, ARNAS Civico di Palermo, Palermo, Italy.; (Alongi G) Hospice e Cure Palliative, ASP 1di Agrigento, Agrigento, Italy.; (Amato F) UOC Terapia del Dolore e Cure Palliative, Azienda ospedaliera di Cosenza, Cosenza, Italy.; (Consoletti L) Struttura di Medicina del Dolore, Ospedale Universitario "Ospedali Riuniti", Foggia, Italy.; (Lamarca L) UOS Cure Palliative e Terapia Antalgica, Azienda ULSS N. 10 "Veneto Orientale", San Donà di Piave, VE, Italy.; (Liguori S) USC Cure Palliative Terapia del Dolore, Azienda Ospedaliera Papa Giovanni XXIII, Bergamo, Italy.; (Lo Presti C) UOD Terapia del Dolore e Cure Palliative, ACO San Filippo Neri, ASLRME, Rome, Italy.; (Maione A) Terapia antalgica e Cure Palliative, Presidio Ospedaliero "S. Maria della Pietà", Nola, NA, Italy.; (Mameli S) SC Terapia del Dolore, Presidio Ospedaliero "A. Businco", Cagliari, Italy.; (Marinangeli F) Scuola di Specializzazione di Anestesia, Rianimazione e Terapia Intensiva, Università dell'Aquila, L'Aquila, Italy.; (Marulli S) OC Anestesia, Rianimazione e Terapia Iperbarica, UOS-I Gruppo Operatorio, Ospedale "Vito Fazzi", Lecce, Italy.; (Minotti V) SC Oncologia Medica, Azienda Ospedaliera "S.M. della Misericordia", Perugia, Italy.; (Miotti D) UO Cure Palliative e Terapia del Dolore, Fondazione Salvatore Maugeri-IRCCS, Pavia, Italy.; (Montanari L) UO Semplice Cure Palliative, Ravenna, Italy.; (Moruzzi G) UOS Hospice, Azienda Sanitaria Provinciale di Siracusa, Siracuse, Italy.; (Palermo S) UOC Terapia Antalgica, IRCCS San Martino-IST, Genoa, Italy.; (Parolini M) UOC Anestesia e Rianimazione B, Azienda Universitaria integrata di Verona, Verona, Italy.; (Poli P) UO Terapia del Dolore, Azienda Ospedaliero-Universitaria Pisana, Pisa, Italy.; (Tirelli W) Centro di Terapia del Dolore, Hospice "Fondazione Roma Sanità", Rome, Italy.; (Valle A) Fondazione FARO, Turin, Italy.; (Romualdi P) Dipartimento di Farmacia e Biotecnologie, Alma mater studiorum, Università di Bologna, Bologna, Italy.
(Working Group Nientemale DEI) Working Group Nientemale DEI; (Vellucci R) SOD Cure Palliative e Terapia del Dolore, Ospedale Universitario Careggi, Florence, Italy. renato.vellucci@gmail.com.; (Fanelli G) SC Anestesia, Rianimazione e Terapia Antalgica, Azienda Ospedaliero-Universitaria di Parma, Parma, Italy.; (Pannuti R) Fondazione ANT Italia Onlus, Andria, Italy.; (Peruselli C) SC Cure Palliative,
Ospedale di Biella, Ponderano, BI, Italy.; (Adamo S) UO Terapia del Dolore, ARNAS Civico di Palermo, Palermo, Italy.; (Alongi G) Hospice e Cure Palliative, ASP 1di Agrigento, Agrigento, Italy.; (Amato F) UOC Terapia del Dolore e Cure Palliative, Azienda ospedaliera di Cosenza, Cosenza, Italy.; (Consoletti L) Struttura di Medicina del Dolore, Ospedale Universitario "Ospedali Riuniti", Foggia, Italy.; (Lamarca L) UOS Cure Palliative e Terapia Antalgica, Azienda ULSS N. 10 "Veneto Orientale", San Donà di Piave, VE, Italy.; (Liguori S) USC Cure Palliative Terapia del Dolore, Azienda Ospedaliera Papa Giovanni XXIII, Bergamo, Italy.; (Lo Presti C) UOD Terapia del Dolore e Cure Palliative, ACO San Filippo Neri, ASLRME, Rome, Italy.; (Maione A) Terapia antalgica e Cure Palliative, Presidio Ospedaliero "S. Maria della Pietà", Nola, NA, Italy.; (Mameli S) SC Terapia del Dolore, Presidio Ospedaliero "A. Businco", Cagliari, Italy.; (Marinangeli F) Scuola di Specializzazione di Anestesia, Rianimazione e Terapia Intensiva, Università dell'Aquila, L'Aquila, Italy.; (Marulli S) OC Anestesia, Rianimazione e Terapia Iperbarica, UOS-I Gruppo Operatorio, Ospedale "Vito Fazzi", Lecce, Italy.; (Minotti V) SC Oncologia Medica, Azienda Ospedaliera "S.M. della Misericordia", Perugia, Italy.; (Miotti D) UO Cure Palliative e Terapia del Dolore, Fondazione Salvatore Maugeri-IRCCS, Pavia, Italy.; (Montanari L) UO Semplice Cure Palliative, Ravenna, Italy.; (Moruzzi G) UOS Hospice, Azienda Sanitaria Provinciale di Siracusa, Siracuse, Italy.; (Palermo S) UOC Terapia Antalgica, IRCCS San Martino-IST, Genoa, Italy.; (Parolini M) UOC Anestesia e

Rianimazione B, Azienda Universitaria integrata di Verona, Verona, Italy.; (Poli P) UO Terapia del Dolore, Azienda Ospedaliero-Universitaria Pisana, Pisa, Italy.; (Tirelli W) Centro di Terapia del Dolore, Hospice "Fondazione Roma Sanità", Rome, Italy.; (Valle A) Fondazione FARO, Turin, Italy.; (Romualdi P) Dipartimento di Farmacia e Biotecnologie, Alma mater studiorum, Università di Bologna, Bologna, Italy. (Working Group Nientemale DEI) Working Group Nientemale DEI; (Vellucci R) SOD Cure Palliative e Terapia del Dolore, Ospedale Universitario Careggi, Florence, Italy. renato.vellucci@gmail.com.; (Fanelli G) SC Anestesia, Rianimazione e Terapia Antalgica, Azienda Ospedaliero-Universitaria di Parma, Parma, Italy.; (Pannuti R) Fondazione ANT Italia Onlus, Andria, Italy.; (Peruselli C) SC Cure Palliative, Ospedale di Biella, Ponderano, BI, Italy.; (Adamo S) UO Terapia del Dolore, ARNAS Civico di Palermo, Palermo, Italy.; (Alongi G) Hospice e Cure Palliative, ASP 1di Agrigento, Agrigento, Italy.; (Amato F) UOC Terapia del Dolore e Cure Palliative, Azienda ospedaliera di Cosenza, Cosenza, Italy.; (Consoletti L) Struttura di Medicina del Dolore, Ospedale Universitario "Ospedali Riuniti", Foggia, Italy.; (Lamarca L) UOS Cure Palliative e Terapia Antalgica, Azienda ULSS N. 10 "Veneto Orientale", San Donà di Piave, VE, Italy.; (Liguori S) USC Cure Palliative Terapia del Dolore, Azienda Ospedaliera Papa Giovanni XXIII, Bergamo, Italy.; (Lo Presti C) UOD Terapia del Dolore e Cure Palliative, ACO San Filippo Neri, ASLRME, Rome, Italy.; (Maione A) Terapia antalgica e Cure Palliative, Presidio Ospedaliero "S. Maria della Pietà", Nola, NA, Italy.; (Mameli S) SC Terapia del Dolore, Presidio Ospedaliero "A. Businco", Cagliari, Italy.; (Marinangeli F) Scuola di Specializzazione di Anestesia, Rianimazione e Terapia Intensiva, Università dell'Aquila, L'Aquila, Italy.; (Marulli S) OC Anestesia, Rianimazione e Terapia Iperbarica, UOS-I Gruppo Operatorio, Ospedale "Vito Fazzi", Lecce, Italy.; (Minotti V) SC Oncologia Medica, Azienda Ospedaliera "S.M. della Misericordia", Perugia, Italy.; (Miotti D) UO Cure Palliative e Terapia del Dolore, Fondazione Salvatore Maugeri-IRCCS, Pavia, Italy.; (Montanari L) UO Semplice Cure Palliative, Ravenna, Italy.; (Moruzzi G) UOS Hospice, Azienda Sanitaria Provinciale di Siracusa, Siracuse, Italy.; (Palermo S) UOC Terapia Antalgica, IRCCS San Martino-IST, Genoa, Italy.; (Parolini M) UOC Anestesia e
Rianimazione B, Azienda Universitaria integrata di Verona, Verona, Italy.; (Poli P) UO Terapia del Dolore, Azienda Ospedaliero-Universitaria Pisana, Pisa, Italy.; (Tirelli W) Centro di Terapia del Dolore, Hospice "Fondazione Roma Sanità", Rome, Italy.; (Valle A) Fondazione FARO, Turin, Italy.; (Romualdi P) Dipartimento di Farmacia e Biotecnologie, Alma mater studiorum, Università di Bologna, Bologna, Italy. (Working Group Nientemale DEI) Working Group Nientemale DEI; (Vellucci R) SOD Cure Palliative e Terapia del Dolore, Ospedale Universitario Careggi, Florence, Italy. renato.vellucci@gmail.com.; (Fanelli G) SC Anestesia, Rianimazione e Terapia Antalgica, Azienda Ospedaliero-Universitaria di Parma, Parma, Italy.; (Pannuti R) Fondazione ANT Italia Onlus, Andria, Italy.; (Peruselli C) SC Cure Palliative, Ospedale di Biella, Ponderano, BI, Italy.; (Adamo S) UO Terapia del Dolore, ARNAS Civico di Palermo, Palermo, Italy.; (Alongi G) Hospice e Cure Palliative, ASP 1di Agrigento, Agrigento, Italy.; (Amato F) UOC Terapia del Dolore e Cure Palliative, Azienda ospedaliera di Cosenza, Cosenza, Italy.; (Consoletti L) Struttura di Medicina del Dolore, Ospedale Universitario "Ospedali Riuniti", Foggia, Italy.; (Lamarca L) UOS Cure Palliative e Terapia Antalgica, Azienda ULSS N. 10 "Veneto Orientale", San Donà di Piave, VE, Italy.; (Liguori S) USC Cure Palliative Terapia del Dolore, Azienda Ospedaliera Papa Giovanni XXIII, Bergamo, Italy.; (Lo Presti C) UOD Terapia del Dolore e Cure Palliative, ACO San Filippo Neri, ASLRME, Rome, Italy.; (Maione A) Terapia antalgica e Cure Palliative, Presidio Ospedaliero "S. Maria della

Pietà", Nola, NA, Italy.; (Mameli S) SC Terapia del Dolore, Presidio Ospedaliero "A. Businco", Cagliari, Italy.; (Marinangeli F) Scuola di Specializzazione di Anestesia, Rianimazione e Terapia Intensiva, Università dell'Aquila, L'Aquila, Italy.; (Marulli S) OC Anestesia, Rianimazione e Terapia Iperbarica, UOS-I Gruppo Operatorio, Ospedale "Vito Fazzi", Lecce, Italy.; (Minotti V) SC Oncologia Medica, Azienda Ospedaliera "S.M. della Misericordia", Perugia, Italy.; (Miotti D) UO Cure Palliative e Terapia del Dolore, Fondazione Salvatore Maugeri-IRCCS, Pavia, Italy.; (Montanari L) UO Semplice Cure Palliative, Ravenna, Italy.; (Moruzzi G) UOS Hospice, Azienda Sanitaria Provinciale di Siracusa, Siracuse, Italy.; (Palermo S) UOC Terapia Antalgica, IRCCS San Martino-IST, Genoa, Italy.; (Parolini M) UOC Anestesia e Rianimazione B, Azienda Universitaria integrata di Verona, Verona, Italy.; (Poli P) UO Terapia del Dolore, Azienda Ospedaliero-Universitaria Pisana, Pisa, Italy.; (Tirelli W) Centro di Terapia del Dolore, Hospice "Fondazione Roma Sanità", Rome, Italy.; (Valle A) Fondazione FARO, Turin, Italy.; (Romualdi P) Dipartimento di Farmacia e Biotecnologie, Alma mater studiorum, Università di Bologna, Bologna, Italy.
(Working Group Nientemale DEI) Working Group Nientemale DEI; (Vellucci R) SOD Cure Palliative e Terapia del Dolore, Ospedale Universitario Careggi, Florence, Italy. renato.vellucci@gmail.com.; (Fanelli G) SC Anestesia, Rianimazione e Terapia Antalgica, Azienda Ospedaliero-Universitaria di Parma, Parma, Italy.; (Pannuti R) Fondazione ANT Italia Onlus, Andria, Italy.; (Peruselli C) SC Cure Palliative, Ospedale di Biella, Ponderano, BI, Italy.; (Adamo S) UO Terapia del Dolore, ARNAS Civico di Palermo, Palermo, Italy.; (Alongi G) Hospice e Cure Palliative, ASP 1di Agrigento, Agrigento, Italy.; (Amato F) UOC Terapia del Dolore e Cure Palliative, Azienda ospedaliera di Cosenza, Cosenza, Italy.; (Consoletti L) Struttura di Medicina del Dolore, Ospedale Universitario "Ospedali Riuniti", Foggia, Italy.; (Lamarca L) UOS Cure Palliative e Terapia Antalgica, Azienda ULSS N. 10 "Veneto Orientale", San Donà di Piave, VE, Italy.; (Liguori S) USC Cure Palliative Terapia del Dolore, Azienda Ospedaliera Papa Giovanni XXIII, Bergamo, Italy.; (Lo Presti C) UOD Terapia del Dolore e Cure Palliative, ACO San Filippo Neri, ASLRME, Rome, Italy.; (Maione A) Terapia antalgica e Cure Palliative, Presidio Ospedaliero "S. Maria della
Pietà", Nola, NA, Italy.; (Mameli S) SC Terapia del Dolore, Presidio Ospedaliero "A. Businco", Cagliari, Italy.; (Marinangeli F) Scuola di Specializzazione di Anestesia, Rianimazione e Terapia Intensiva, Università dell'Aquila, L'Aquila, Italy.; (Marulli S) OC Anestesia, Rianimazione e Terapia Iperbarica, UOS-I Gruppo Operatorio, Ospedale "Vito Fazzi", Lecce, Italy.; (Minotti V) SC Oncologia Medica, Azienda Ospedaliera "S.M. della Misericordia", Perugia, Italy.; (Miotti D) UO Cure Palliative e Terapia del Dolore, Fondazione Salvatore Maugeri-IRCCS, Pavia, Italy.; (Montanari L) UO Semplice Cure Palliative, Ravenna, Italy.; (Moruzzi G) UOS Hospice, Azienda Sanitaria Provinciale di Siracusa, Siracuse, Italy.; (Palermo S) UOC Terapia Antalgica, IRCCS San Martino-IST, Genoa, Italy.; (Parolini M) UOC Anestesia e Rianimazione B, Azienda Universitaria integrata di Verona, Verona, Italy.; (Poli P) UO Terapia del Dolore, Azienda Ospedaliero-Universitaria Pisana, Pisa, Italy.; (Tirelli W) Centro di Terapia del Dolore, Hospice "Fondazione Roma Sanità", Rome, Italy.; (Valle A) Fondazione FARO, Turin, Italy.; (Romualdi P) Dipartimento di Farmacia e Biotecnologie, Alma mater studiorum, Università di Bologna, Bologna, Italy.
(Working Group Nientemale DEI) Working Group Nientemale DEI; (Vellucci R) SOD Cure Palliative e Terapia del Dolore, Ospedale Universitario Careggi, Florence, Italy. renato.vellucci@gmail.com.; (Fanelli G) SC Anestesia, Rianimazione e Terapia Antalgica, Azienda Ospedaliero-Universitaria di Parma, Parma, Italy.; (Pannuti R) Fondazione ANT Italia Onlus, Andria, Italy.; (Peruselli C) SC Cure Palliative,

Ospedale di Biella, Ponderano, BI, Italy.; (Adamo S) UO Terapia del Dolore, ARNAS Civico di Palermo, Palermo, Italy.; (Alongi G) Hospice e Cure Palliative, ASP 1di Agrigento, Agrigento, Italy.; (Amato F) UOC Terapia del Dolore e Cure Palliative, Azienda ospedaliera di Cosenza, Cosenza, Italy.; (Consoletti L) Struttura di Medicina del Dolore, Ospedale Universitario "Ospedali Riuniti", Foggia, Italy.; (Lamarca L) UOS Cure Palliative e Terapia Antalgica, Azienda ULSS N. 10 "Veneto Orientale", San Donà di Piave, VE, Italy.; (Liguori S) USC Cure Palliative Terapia del Dolore, Azienda Ospedaliera Papa Giovanni XXIII, Bergamo, Italy.; (Lo Presti C) UOD Terapia del Dolore e Cure Palliative, ACO San Filippo Neri, ASLRME, Rome, Italy.; (Maione A) Terapia antalgica e Cure Palliative, Presidio Ospedaliero "S. Maria della Pietà", Nola, NA, Italy.; (Mameli S) SC Terapia del Dolore, Presidio Ospedaliero "A. Businco", Cagliari, Italy.; (Marinangeli F) Scuola di Specializzazione di Anestesia, Rianimazione e Terapia Intensiva, Università dell'Aquila, L'Aquila, Italy.; (Marulli S) OC Anestesia, Rianimazione e Terapia Iperbarica, UOS-I Gruppo Operatorio, Ospedale "Vito Fazzi", Lecce, Italy.; (Minotti V) SC Oncologia Medica, Azienda Ospedaliera "S.M. della Misericordia", Perugia, Italy.; (Miotti D) UO Cure Palliative e Terapia del Dolore, Fondazione Salvatore Maugeri-IRCCS, Pavia, Italy.; (Montanari L) UO Semplice Cure Palliative, Ravenna, Italy.; (Moruzzi G) UOS Hospice, Azienda Sanitaria Provinciale di Siracusa, Siracuse, Italy.; (Palermo S) UOC Terapia Antalgica, IRCCS San Martino-IST, Genoa, Italy.; (Parolini M) UOC Anestesia e Rianimazione B, Azienda Universitaria integrata di Verona, Verona, Italy.; (Poli P) UO Terapia del Dolore, Azienda Ospedaliero-Universitaria Pisana, Pisa, Italy.; (Tirelli W) Centro di Terapia del Dolore, Hospice "Fondazione Roma Sanità", Rome, Italy.; (Valle A) Fondazione FARO, Turin, Italy.; (Romualdi P) Dipartimento di Farmacia e Biotecnologie, Alma mater studiorum, Università di Bologna, Bologna, Italy. (Working Group Nientemale DEI) Working Group Nientemale DEI; (Vellucci R) SOD Cure Palliative e Terapia del Dolore, Ospedale Universitario Careggi, Florence, Italy. renato.vellucci@gmail.com.; (Fanelli G) SC Anestesia, Rianimazione e Terapia Antalgica, Azienda Ospedaliero-Universitaria di Parma, Parma, Italy.; (Pannuti R) Fondazione ANT Italia Onlus, Andria, Italy.; (Peruselli C) SC Cure Palliative,
Ospedale di Biella, Ponderano, BI, Italy.; (Adamo S) UO Terapia del Dolore, ARNAS Civico di Palermo, Palermo, Italy.; (Alongi G) Hospice e Cure Palliative, ASP 1di Agrigento, Agrigento, Italy.; (Amato F) UOC Terapia del Dolore e Cure Palliative, Azienda ospedaliera di Cosenza, Cosenza, Italy.; (Consoletti L) Struttura di Medicina del Dolore, Ospedale Universitario "Ospedali Riuniti", Foggia, Italy.; (Lamarca L) UOS Cure Palliative e Terapia Antalgica, Azienda ULSS N. 10 "Veneto Orientale", San Donà di Piave, VE, Italy.; (Liguori S) USC Cure Palliative Terapia del Dolore, Azienda Ospedaliera Papa Giovanni XXIII, Bergamo, Italy.; (Lo Presti C) UOD Terapia del Dolore e Cure Palliative, ACO San Filippo Neri, ASLRME, Rome, Italy.; (Maione A) Terapia antalgica e Cure Palliative, Presidio Ospedaliero "S. Maria della Pietà", Nola, NA, Italy.; (Mameli S) SC Terapia del Dolore, Presidio Ospedaliero "A. Businco", Cagliari, Italy.; (Marinangeli F) Scuola di Specializzazione di Anestesia, Rianimazione e Terapia Intensiva, Università dell'Aquila, L'Aquila, Italy.; (Marulli S) OC Anestesia, Rianimazione e Terapia Iperbarica, UOS-I Gruppo Operatorio, Ospedale "Vito Fazzi", Lecce, Italy.; (Minotti V) SC Oncologia Medica, Azienda Ospedaliera "S.M. della Misericordia", Perugia, Italy.; (Miotti D) UO Cure Palliative e Terapia del Dolore, Fondazione Salvatore Maugeri-IRCCS, Pavia, Italy.; (Montanari L) UO Semplice Cure Palliative, Ravenna, Italy.; (Moruzzi G) UOS Hospice, Azienda Sanitaria Provinciale di Siracusa, Siracuse, Italy.; (Palermo S) UOC Terapia Antalgica, IRCCS San Martino-IST, Genoa, Italy.; (Parolini M) UOC Anestesia e

Rianimazione B, Azienda Universitaria integrata di Verona, Verona, Italy.; (Poli P) UO Terapia del Dolore, Azienda Ospedaliero-Universitaria Pisana, Pisa, Italy.; (Tirelli W) Centro di Terapia del Dolore, Hospice "Fondazione Roma Sanità", Rome, Italy.; (Valle A) Fondazione FARO, Turin, Italy.; (Romualdi P) Dipartimento di Farmacia e Biotecnologie, Alma mater studiorum, Università di Bologna, Bologna, Italy. (Working Group Nientemale DEI) Working Group Nientemale DEI; (Vellucci R) SOD Cure Palliative e Terapia del Dolore, Ospedale Universitario Careggi, Florence, Italy. renato.vellucci@gmail.com.; (Fanelli G) SC Anestesia, Rianimazione e Terapia Antalgica, Azienda Ospedaliero-Universitaria di Parma, Parma, Italy.; (Pannuti R) Fondazione ANT Italia Onlus, Andria, Italy.; (Peruselli C) SC Cure Palliative, Ospedale di Biella, Ponderano, BI, Italy.; (Adamo S) UO Terapia del Dolore, ARNAS Civico di Palermo, Palermo, Italy.; (Alongi G) Hospice e Cure Palliative, ASP 1di Agrigento, Agrigento, Italy.; (Amato F) UOC Terapia del Dolore e Cure Palliative, Azienda ospedaliera di Cosenza, Cosenza, Italy.; (Consoletti L) Struttura di Medicina del Dolore, Ospedale Universitario "Ospedali Riuniti", Foggia, Italy.; (Lamarca L) UOS Cure Palliative e Terapia Antalgica, Azienda ULSS N. 10 "Veneto Orientale", San Donà di Piave, VE, Italy.; (Liguori S) USC Cure Palliative Terapia del Dolore, Azienda Ospedaliera Papa Giovanni XXIII, Bergamo, Italy.; (Lo Presti C) UOD Terapia del Dolore e Cure Palliative, ACO San Filippo Neri, ASLRME, Rome, Italy.; (Maione A) Terapia antalgica e Cure Palliative, Presidio Ospedaliero "S. Maria della Pietà", Nola, NA, Italy.; (Mameli S) SC Terapia del Dolore, Presidio Ospedaliero "A. Businco", Cagliari, Italy.; (Marinangeli F) Scuola di Specializzazione di Anestesia, Rianimazione e Terapia Intensiva, Università dell'Aquila, L'Aquila, Italy.; (Marulli S) OC Anestesia, Rianimazione e Terapia Iperbarica, UOS-I Gruppo Operatorio, Ospedale "Vito Fazzi", Lecce, Italy.; (Minotti V) SC Oncologia Medica, Azienda Ospedaliera "S.M. della Misericordia", Perugia, Italy.; (Miotti D) UO Cure Palliative e Terapia del Dolore, Fondazione Salvatore Maugeri-IRCCS, Pavia, Italy.; (Montanari L) UO Semplice Cure Palliative, Ravenna, Italy.; (Moruzzi G) UOS Hospice, Azienda Sanitaria Provinciale di Siracusa, Siracuse, Italy.; (Palermo S) UOC Terapia Antalgica, IRCCS San Martino-IST, Genoa, Italy.; (Parolini M) UOC Anestesia e
Rianimazione B, Azienda Universitaria integrata di Verona, Verona, Italy.; (Poli P) UO Terapia del Dolore, Azienda Ospedaliero-Universitaria Pisana, Pisa, Italy.; (Tirelli W) Centro di Terapia del Dolore, Hospice "Fondazione Roma Sanità", Rome, Italy.; (Valle A) Fondazione FARO, Turin, Italy.; (Romualdi P) Dipartimento di Farmacia e Biotecnologie, Alma mater studiorum, Università di Bologna, Bologna, Italy. (Working Group Nientemale DEI) Working Group Nientemale DEI; (Vellucci R) SOD Cure Palliative e Terapia del Dolore, Ospedale Universitario Careggi, Florence, Italy. renato.vellucci@gmail.com.; (Fanelli G) SC Anestesia, Rianimazione e Terapia Antalgica, Azienda Ospedaliero-Universitaria di Parma, Parma, Italy.; (Pannuti R) Fondazione ANT Italia Onlus, Andria, Italy.; (Peruselli C) SC Cure Palliative, Ospedale di Biella, Ponderano, BI, Italy.; (Adamo S) UO Terapia del Dolore, ARNAS Civico di Palermo, Palermo, Italy.; (Alongi G) Hospice e Cure Palliative, ASP 1di Agrigento, Agrigento, Italy.; (Amato F) UOC Terapia del Dolore e Cure Palliative, Azienda ospedaliera di Cosenza, Cosenza, Italy.; (Consoletti L) Struttura di Medicina del Dolore, Ospedale Universitario "Ospedali Riuniti", Foggia, Italy.; (Lamarca L) UOS Cure Palliative e Terapia Antalgica, Azienda ULSS N. 10 "Veneto Orientale", San Donà di Piave, VE, Italy.; (Liguori S) USC Cure Palliative Terapia del Dolore, Azienda Ospedaliera Papa Giovanni XXIII, Bergamo, Italy.; (Lo Presti C) UOD Terapia del Dolore e Cure Palliative, ACO San Filippo Neri, ASLRME, Rome, Italy.; (Maione A) Terapia antalgica e Cure Palliative, Presidio Ospedaliero "S. Maria della

Pietà", Nola, NA, Italy.; (Mameli S) SC Terapia del Dolore, Presidio Ospedaliero "A. Businco", Cagliari, Italy.; (Marinangeli F) Scuola di Specializzazione di Anestesia, Rianimazione e Terapia Intensiva, Università dell'Aquila, L'Aquila, Italy.; (Marulli S) OC Anestesia, Rianimazione e Terapia Iperbarica, UOS-I Gruppo Operatorio, Ospedale "Vito Fazzi", Lecce, Italy.; (Minotti V) SC Oncologia Medica, Azienda Ospedaliera "S.M. della Misericordia", Perugia, Italy.; (Miotti D) UO Cure Palliative e Terapia del Dolore, Fondazione Salvatore Maugeri-IRCCS, Pavia, Italy.; (Montanari L) UO Semplice Cure Palliative, Ravenna, Italy.; (Moruzzi G) UOS Hospice, Azienda Sanitaria Provinciale di Siracusa, Siracuse, Italy.; (Palermo S) UOC Terapia Antalgica, IRCCS San Martino-IST, Genoa, Italy.; (Parolini M) UOC Anestesia e Rianimazione B, Azienda Universitaria integrata di Verona, Verona, Italy.; (Poli P) UO Terapia del Dolore, Azienda Ospedaliero-Universitaria Pisana, Pisa, Italy.; (Tirelli W) Centro di Terapia del Dolore, Hospice "Fondazione Roma Sanità", Rome, Italy.; (Valle A) Fondazione FARO, Turin, Italy.; (Romualdi P) Dipartimento di Farmacia e Biotecnologie, Alma mater studiorum, Università di Bologna, Bologna, Italy. (Working Group Nientemale DEI) Working Group Nientemale DEI; (Vellucci R) SOD Cure Palliative e Terapia del Dolore, Ospedale Universitario Careggi, Florence, Italy. renato.vellucci@gmail.com.; (Fanelli G) SC Anestesia, Rianimazione e Terapia Antalgica, Azienda Ospedaliero-Universitaria di Parma, Parma, Italy.; (Pannuti R) Fondazione ANT Italia Onlus, Andria, Italy.; (Peruselli C) SC Cure Palliative, Ospedale di Biella, Ponderano, BI, Italy.; (Adamo S) UO Terapia del Dolore, ARNAS Civico di Palermo, Palermo, Italy.; (Alongi G) Hospice e Cure Palliative, ASP 1di Agrigento, Agrigento, Italy.; (Amato F) UOC Terapia del Dolore e Cure Palliative, Azienda ospedaliera di Cosenza, Cosenza, Italy.; (Consoletti L) Struttura di Medicina del Dolore, Ospedale Universitario "Ospedali Riuniti", Foggia, Italy.; (Lamarca L) UOS Cure Palliative e Terapia Antalgica, Azienda ULSS N. 10 "Veneto Orientale", San Donà di Piave, VE, Italy.; (Liguori S) USC Cure Palliative Terapia del Dolore, Azienda Ospedaliera Papa Giovanni XXIII, Bergamo, Italy.; (Lo Presti C) UOD Terapia del Dolore e Cure Palliative, ACO San Filippo Neri, ASLRME, Rome, Italy.; (Maione A) Terapia antalgica e Cure Palliative, Presidio Ospedaliero "S. Maria della Pietà", Nola, NA, Italy.; (Mameli S) SC Terapia del Dolore, Presidio Ospedaliero "A. Businco", Cagliari, Italy.; (Marinangeli F) Scuola di Specializzazione di Anestesia, Rianimazione e Terapia Intensiva, Università dell'Aquila, L'Aquila, Italy.; (Marulli S) OC Anestesia, Rianimazione e Terapia Iperbarica, UOS-I Gruppo Operatorio, Ospedale "Vito Fazzi", Lecce, Italy.; (Minotti V) SC Oncologia Medica, Azienda Ospedaliera "S.M. della Misericordia", Perugia, Italy.; (Miotti D) UO Cure Palliative e Terapia del Dolore, Fondazione Salvatore Maugeri-IRCCS, Pavia, Italy.; (Montanari L) UO Semplice Cure Palliative, Ravenna, Italy.; (Moruzzi G) UOS Hospice, Azienda Sanitaria Provinciale di Siracusa, Siracuse, Italy.; (Palermo S) UOC Terapia Antalgica, IRCCS San Martino-IST, Genoa, Italy.; (Parolini M) UOC Anestesia e Rianimazione B, Azienda Universitaria integrata di Verona, Verona, Italy.; (Poli P) UO Terapia del Dolore, Azienda Ospedaliero-Universitaria Pisana, Pisa, Italy.; (Tirelli W) Centro di Terapia del Dolore, Hospice "Fondazione Roma Sanità", Rome, Italy.; (Valle A) Fondazione FARO, Turin, Italy.; (Romualdi P) Dipartimento di Farmacia e Biotecnologie, Alma mater studiorum, Università di Bologna, Bologna, Italy. (Working Group Nientemale DEI) Working Group Nientemale DEI; (Vellucci R) SOD Cure Palliative e Terapia del Dolore, Ospedale Universitario Careggi, Florence, Italy. renato.vellucci@gmail.com.; (Fanelli G) SC Anestesia, Rianimazione e Terapia Antalgica, Azienda Ospedaliero-Universitaria di Parma, Parma, Italy.; (Pannuti R) Fondazione ANT Italia Onlus, Andria, Italy.; (Peruselli C) SC Cure Palliative,

Ospedale di Biella, Ponderano, BI, Italy.; (Adamo S) UO Terapia del Dolore, ARNAS Civico di Palermo, Palermo, Italy.; (Alongi G) Hospice e Cure Palliative, ASP 1di Agrigento, Agrigento, Italy.; (Amato F) UOC Terapia del Dolore e Cure Palliative, Azienda ospedaliera di Cosenza, Cosenza, Italy.; (Consoletti L) Struttura di Medicina del Dolore, Ospedale Universitario "Ospedali Riuniti", Foggia, Italy.; (Lamarca L) UOS Cure Palliative e Terapia Antalgica, Azienda ULSS N. 10 "Veneto Orientale", San Donà di Piave, VE, Italy.; (Liguori S) USC Cure Palliative Terapia del Dolore, Azienda Ospedaliera Papa Giovanni XXIII, Bergamo, Italy.; (Lo Presti C) UOD Terapia del Dolore e Cure Palliative, ACO San Filippo Neri, ASLRME, Rome, Italy.; (Maione A) Terapia antalgica e Cure Palliative, Presidio Ospedaliero "S. Maria della Pietà", Nola, NA, Italy.; (Mameli S) SC Terapia del Dolore, Presidio Ospedaliero "A. Businco", Cagliari, Italy.; (Marinangeli F) Scuola di Specializzazione di Anestesia, Rianimazione e Terapia Intensiva, Università dell'Aquila, L'Aquila, Italy.; (Marulli S) OC Anestesia, Rianimazione e Terapia Iperbarica, UOS-I Gruppo Operatorio, Ospedale "Vito Fazzi", Lecce, Italy.; (Minotti V) SC Oncologia Medica, Azienda Ospedaliera "S.M. della Misericordia", Perugia, Italy.; (Miotti D) UO Cure Palliative e Terapia del Dolore, Fondazione Salvatore Maugeri-IRCCS, Pavia, Italy.; (Montanari L) UO Semplice Cure Palliative, Ravenna, Italy.; (Moruzzi G) UOS Hospice, Azienda Sanitaria Provinciale di Siracusa, Siracuse, Italy.; (Palermo S) UOC Terapia Antalgica, IRCCS San Martino-IST, Genoa, Italy.; (Parolini M) UOC Anestesia e Rianimazione B, Azienda Universitaria integrata di Verona, Verona, Italy.; (Poli P) UO Terapia del Dolore, Azienda Ospedaliero-Universitaria Pisana, Pisa, Italy.; (Tirelli W) Centro di Terapia del Dolore, Hospice "Fondazione Roma Sanità", Rome, Italy.; (Valle A) Fondazione FARO, Turin, Italy.; (Romualdi P) Dipartimento di Farmacia e Biotecnologie, Alma mater studiorum, Università di Bologna, Bologna, Italy. (Working Group Nientemale DEI) Working Group Nientemale DEI; (Vellucci R) SOD Cure Palliative e Terapia del Dolore, Ospedale Universitario Careggi, Florence, Italy. renato.vellucci@gmail.com.; (Fanelli G) SC Anestesia, Rianimazione e Terapia Antalgica, Azienda Ospedaliero-Universitaria di Parma, Parma, Italy.; (Pannuti R) Fondazione ANT Italia Onlus, Andria, Italy.; (Peruselli C) SC Cure Palliative,
Ospedale di Biella, Ponderano, BI, Italy.; (Adamo S) UO Terapia del Dolore, ARNAS Civico di Palermo, Palermo, Italy.; (Alongi G) Hospice e Cure Palliative, ASP 1di Agrigento, Agrigento, Italy.; (Amato F) UOC Terapia del Dolore e Cure Palliative, Azienda ospedaliera di Cosenza, Cosenza, Italy.; (Consoletti L) Struttura di Medicina del Dolore, Ospedale Universitario "Ospedali Riuniti", Foggia, Italy.; (Lamarca L) UOS Cure Palliative e Terapia Antalgica, Azienda ULSS N. 10 "Veneto Orientale", San Donà di Piave, VE, Italy.; (Liguori S) USC Cure Palliative Terapia del Dolore, Azienda Ospedaliera Papa Giovanni XXIII, Bergamo, Italy.; (Lo Presti C) UOD Terapia del Dolore e Cure Palliative, ACO San Filippo Neri, ASLRME, Rome, Italy.; (Maione A) Terapia antalgica e Cure Palliative, Presidio Ospedaliero "S. Maria della Pietà", Nola, NA, Italy.; (Mameli S) SC Terapia del Dolore, Presidio Ospedaliero "A. Businco", Cagliari, Italy.; (Marinangeli F) Scuola di Specializzazione di Anestesia, Rianimazione e Terapia Intensiva, Università dell'Aquila, L'Aquila, Italy.; (Marulli S) OC Anestesia, Rianimazione e Terapia Iperbarica, UOS-I Gruppo Operatorio, Ospedale "Vito Fazzi", Lecce, Italy.; (Minotti V) SC Oncologia Medica, Azienda Ospedaliera "S.M. della Misericordia", Perugia, Italy.; (Miotti D) UO Cure Palliative e Terapia del Dolore, Fondazione Salvatore Maugeri-IRCCS, Pavia, Italy.; (Montanari L) UO Semplice Cure Palliative, Ravenna, Italy.; (Moruzzi G) UOS Hospice, Azienda Sanitaria Provinciale di Siracusa, Siracuse, Italy.; (Palermo S) UOC Terapia Antalgica, IRCCS San Martino-IST, Genoa, Italy.; (Parolini M) UOC Anestesia e

Rianimazione B, Azienda Universitaria integrata di Verona, Verona, Italy.; (Poli P) UO Terapia del Dolore, Azienda Ospedaliero-Universitaria Pisana, Pisa, Italy.; (Tirelli W) Centro di Terapia del Dolore, Hospice "Fondazione Roma Sanità", Rome, Italy.; (Valle A) Fondazione FARO, Turin, Italy.; (Romualdi P) Dipartimento di Farmacia e Biotecnologie, Alma mater studiorum, Università di Bologna, Bologna, Italy. (Working Group Nientemale DEI) Working Group Nientemale DEI; (Vellucci R) SOD Cure Palliative e Terapia del Dolore, Ospedale Universitario Careggi, Florence, Italy. renato.vellucci@gmail.com.; (Fanelli G) SC Anestesia, Rianimazione e Terapia Antalgica, Azienda Ospedaliero-Universitaria di Parma, Parma, Italy.; (Pannuti R) Fondazione ANT Italia Onlus, Andria, Italy.; (Peruselli C) SC Cure Palliative, Ospedale di Biella, Ponderano, BI, Italy.; (Adamo S) UO Terapia del Dolore, ARNAS Civico di Palermo, Palermo, Italy.; (Alongi G) Hospice e Cure Palliative, ASP 1di Agrigento, Agrigento, Italy.; (Amato F) UOC Terapia del Dolore e Cure Palliative, Azienda ospedaliera di Cosenza, Cosenza, Italy.; (Consoletti L) Struttura di Medicina del Dolore, Ospedale Universitario "Ospedali Riuniti", Foggia, Italy.; (Lamarca L) UOS Cure Palliative e Terapia Antalgica, Azienda ULSS N. 10 "Veneto Orientale", San Donà di Piave, VE, Italy.; (Liguori S) USC Cure Palliative Terapia del Dolore, Azienda Ospedaliera Papa Giovanni XXIII, Bergamo, Italy.; (Lo Presti C) UOD Terapia del Dolore e Cure Palliative, ACO San Filippo Neri, ASLRME, Rome, Italy.; (Maione A) Terapia antalgica e Cure Palliative, Presidio Ospedaliero "S. Maria della Pietà", Nola, NA, Italy.; (Mameli S) SC Terapia del Dolore, Presidio Ospedaliero "A. Businco", Cagliari, Italy.; (Marinangeli F) Scuola di Specializzazione di Anestesia, Rianimazione e Terapia Intensiva, Università dell'Aquila, L'Aquila, Italy.; (Marulli S) OC Anestesia, Rianimazione e Terapia Iperbarica, UOS-I Gruppo Operatorio, Ospedale "Vito Fazzi", Lecce, Italy.; (Minotti V) SC Oncologia Medica, Azienda Ospedaliera "S.M. della Misericordia", Perugia, Italy.; (Miotti D) UO Cure Palliative e Terapia del Dolore, Fondazione Salvatore Maugeri-IRCCS, Pavia, Italy.; (Montanari L) UO Semplice Cure Palliative, Ravenna, Italy.; (Moruzzi G) UOS Hospice, Azienda Sanitaria Provinciale di Siracusa, Siracuse, Italy.; (Palermo S) UOC Terapia Antalgica, IRCCS San Martino-IST, Genoa, Italy.; (Parolini M) UOC Anestesia e
Rianimazione B, Azienda Universitaria integrata di Verona, Verona, Italy.; (Poli P) UO Terapia del Dolore, Azienda Ospedaliero-Universitaria Pisana, Pisa, Italy.; (Tirelli W) Centro di Terapia del Dolore, Hospice "Fondazione Roma Sanità", Rome, Italy.; (Valle A) Fondazione FARO, Turin, Italy.; (Romualdi P) Dipartimento di Farmacia e Biotecnologie, Alma mater studiorum, Università di Bologna, Bologna, Italy. (Working Group Nientemale DEI) Working Group Nientemale DEI; (Vellucci R) SOD Cure Palliative e Terapia del Dolore, Ospedale Universitario Careggi, Florence, Italy. renato.vellucci@gmail.com.; (Fanelli G) SC Anestesia, Rianimazione e Terapia Antalgica, Azienda Ospedaliero-Universitaria di Parma, Parma, Italy.; (Pannuti R) Fondazione ANT Italia Onlus, Andria, Italy.; (Peruselli C) SC Cure Palliative, Ospedale di Biella, Ponderano, BI, Italy.; (Adamo S) UO Terapia del Dolore, ARNAS Civico di Palermo, Palermo, Italy.; (Alongi G) Hospice e Cure Palliative, ASP 1di Agrigento, Agrigento, Italy.; (Amato F) UOC Terapia del Dolore e Cure Palliative, Azienda ospedaliera di Cosenza, Cosenza, Italy.; (Consoletti L) Struttura di Medicina del Dolore, Ospedale Universitario "Ospedali Riuniti", Foggia, Italy.; (Lamarca L) UOS Cure Palliative e Terapia Antalgica, Azienda ULSS N. 10 "Veneto Orientale", San Donà di Piave, VE, Italy.; (Liguori S) USC Cure Palliative Terapia del Dolore, Azienda Ospedaliera Papa Giovanni XXIII, Bergamo, Italy.; (Lo Presti C) UOD Terapia del Dolore e Cure Palliative, ACO San Filippo Neri, ASLRME, Rome, Italy.; (Maione A) Terapia antalgica e Cure Palliative, Presidio Ospedaliero "S. Maria della

Pietà", Nola, NA, Italy.; (Mameli S) SC Terapia del Dolore, Presidio Ospedaliero "A. Businco", Cagliari, Italy.; (Marinangeli F) Scuola di Specializzazione di Anestesia, Rianimazione e Terapia Intensiva, Università dell'Aquila, L'Aquila, Italy.; (Marulli S) OC Anestesia, Rianimazione e Terapia Iperbarica, UOS-I Gruppo Operatorio, Ospedale "Vito Fazzi", Lecce, Italy.; (Minotti V) SC Oncologia Medica, Azienda Ospedaliera "S.M. della Misericordia", Perugia, Italy.; (Miotti D) UO Cure Palliative e Terapia del Dolore, Fondazione Salvatore Maugeri-IRCCS, Pavia, Italy.; (Montanari L) UO Semplice Cure Palliative, Ravenna, Italy.; (Moruzzi G) UOS Hospice, Azienda Sanitaria Provinciale di Siracusa, Siracuse, Italy.; (Palermo S) UOC Terapia Antalgica, IRCCS San Martino-IST, Genoa, Italy.; (Parolini M) UOC Anestesia e Rianimazione B, Azienda Universitaria integrata di Verona, Verona, Italy.; (Poli P) UO Terapia del Dolore, Azienda Ospedaliero-Universitaria Pisana, Pisa, Italy.; (Tirelli W) Centro di Terapia del Dolore, Hospice "Fondazione Roma Sanità", Rome, Italy.; (Valle A) Fondazione FARO, Turin, Italy.; (Romualdi P) Dipartimento di Farmacia e Biotecnologie, Alma mater studiorum, Università di Bologna, Bologna, Italy. (Working Group Nientemale DEI) Working Group Nientemale DEI; (Vellucci R) SOD Cure Palliative e Terapia del Dolore, Ospedale Universitario Careggi, Florence, Italy. renato.vellucci@gmail.com.; (Fanelli G) SC Anestesia, Rianimazione e Terapia Antalgica, Azienda Ospedaliero-Universitaria di Parma, Parma, Italy.; (Pannuti R) Fondazione ANT Italia Onlus, Andria, Italy.; (Peruselli C) SC Cure Palliative, Ospedale di Biella, Ponderano, BI, Italy.; (Adamo S) UO Terapia del Dolore, ARNAS Civico di Palermo, Palermo, Italy.; (Alongi G) Hospice e Cure Palliative, ASP 1di Agrigento, Agrigento, Italy.; (Amato F) UOC Terapia del Dolore e Cure Palliative, Azienda ospedaliera di Cosenza, Cosenza, Italy.; (Consoletti L) Struttura di Medicina del Dolore, Ospedale Universitario "Ospedali Riuniti", Foggia, Italy.; (Lamarca L) UOS Cure Palliative e Terapia Antalgica, Azienda ULSS N. 10 "Veneto Orientale", San Donà di Piave, VE, Italy.; (Liguori S) USC Cure Palliative Terapia del Dolore, Azienda Ospedaliera Papa Giovanni XXIII, Bergamo, Italy.; (Lo Presti C) UOD Terapia del Dolore e Cure Palliative, ACO San Filippo Neri, ASLRME, Rome, Italy.; (Maione A) Terapia antalgica e Cure Palliative, Presidio Ospedaliero "S. Maria della
Pietà", Nola, NA, Italy.; (Mameli S) SC Terapia del Dolore, Presidio Ospedaliero "A. Businco", Cagliari, Italy.; (Marinangeli F) Scuola di Specializzazione di Anestesia, Rianimazione e Terapia Intensiva, Università dell'Aquila, L'Aquila, Italy.; (Marulli S) OC Anestesia, Rianimazione e Terapia Iperbarica, UOS-I Gruppo Operatorio, Ospedale "Vito Fazzi", Lecce, Italy.; (Minotti V) SC Oncologia Medica, Azienda Ospedaliera "S.M. della Misericordia", Perugia, Italy.; (Miotti D) UO Cure Palliative e Terapia del Dolore, Fondazione Salvatore Maugeri-IRCCS, Pavia, Italy.; (Montanari L) UO Semplice Cure Palliative, Ravenna, Italy.; (Moruzzi G) UOS Hospice, Azienda Sanitaria Provinciale di Siracusa, Siracuse, Italy.; (Palermo S) UOC Terapia Antalgica, IRCCS San Martino-IST, Genoa, Italy.; (Parolini M) UOC Anestesia e Rianimazione B, Azienda Universitaria integrata di Verona, Verona, Italy.; (Poli P) UO Terapia del Dolore, Azienda Ospedaliero-Universitaria Pisana, Pisa, Italy.; (Tirelli W) Centro di Terapia del Dolore, Hospice "Fondazione Roma Sanità", Rome, Italy.; (Valle A) Fondazione FARO, Turin, Italy.; (Romualdi P) Dipartimento di Farmacia e Biotecnologie, Alma mater studiorum, Università di Bologna, Bologna, Italy. (Working Group Nientemale DEI) Working Group Nientemale DEI; (Vellucci R) SOD Cure Palliative e Terapia del Dolore, Ospedale Universitario Careggi, Florence, Italy. renato.vellucci@gmail.com.; (Fanelli G) SC Anestesia, Rianimazione e Terapia Antalgica, Azienda Ospedaliero-Universitaria di Parma, Parma, Italy.; (Pannuti R) Fondazione ANT Italia Onlus, Andria, Italy.; (Peruselli C) SC Cure Palliative,

Ospedale di Biella, Ponderano, BI, Italy.; (Adamo S) UO Terapia del Dolore, ARNAS Civico di Palermo, Palermo, Italy.; (Alongi G) Hospice e Cure Palliative, ASP 1di Agrigento, Agrigento, Italy.; (Amato F) UOC Terapia del Dolore e Cure Palliative, Azienda ospedaliera di Cosenza, Cosenza, Italy.; (Consoletti L) Struttura di Medicina del Dolore, Ospedale Universitario "Ospedali Riuniti", Foggia, Italy.; (Lamarca L) UOS Cure Palliative e Terapia Antalgica, Azienda ULSS N. 10 "Veneto Orientale", San Donà di Piave, VE, Italy.; (Liguori S) USC Cure Palliative Terapia del Dolore, Azienda Ospedaliera Papa Giovanni XXIII, Bergamo, Italy.; (Lo Presti C) UOD Terapia del Dolore e Cure Palliative, ACO San Filippo Neri, ASLRME, Rome, Italy.; (Maione A) Terapia antalgica e Cure Palliative, Presidio Ospedaliero "S. Maria della Pietà", Nola, NA, Italy.; (Mameli S) SC Terapia del Dolore, Presidio Ospedaliero "A. Businco", Cagliari, Italy.; (Marinangeli F) Scuola di Specializzazione di Anestesia, Rianimazione e Terapia Intensiva, Università dell'Aquila, L'Aquila, Italy.; (Marulli S) OC Anestesia, Rianimazione e Terapia Iperbarica, UOS-I Gruppo Operatorio, Ospedale "Vito Fazzi", Lecce, Italy.; (Minotti V) SC Oncologia Medica, Azienda Ospedaliera "S.M. della Misericordia", Perugia, Italy.; (Miotti D) UO Cure Palliative e Terapia del Dolore, Fondazione Salvatore Maugeri-IRCCS, Pavia, Italy.; (Montanari L) UO Semplice Cure Palliative, Ravenna, Italy.; (Moruzzi G) UOS Hospice, Azienda Sanitaria Provinciale di Siracusa, Siracuse, Italy.; (Palermo S) UOC Terapia Antalgica, IRCCS San Martino-IST, Genoa, Italy.; (Parolini M) UOC Anestesia e Rianimazione B, Azienda Universitaria integrata di Verona, Verona, Italy.; (Poli P) UO Terapia del Dolore, Azienda Ospedaliero-Universitaria Pisana, Pisa, Italy.; (Tirelli W) Centro di Terapia del Dolore, Hospice "Fondazione Roma Sanità", Rome, Italy.; (Valle A) Fondazione FARO, Turin, Italy.; (Romualdi P) Dipartimento di Farmacia e Biotecnologie, Alma mater studiorum, Università di Bologna, Bologna, Italy. (Working Group Nientemale DEI) Working Group Nientemale DEI; (Vellucci R) SOD Cure Palliative e Terapia del Dolore, Ospedale Universitario Careggi, Florence, Italy. renato.vellucci@gmail.com.; (Fanelli G) SC Anestesia, Rianimazione e Terapia Antalgica, Azienda Ospedaliero-Universitaria di Parma, Parma, Italy.; (Pannuti R) Fondazione ANT Italia Onlus, Andria, Italy.; (Peruselli C) SC Cure Palliative,
Ospedale di Biella, Ponderano, BI, Italy.; (Adamo S) UO Terapia del Dolore, ARNAS Civico di Palermo, Palermo, Italy.; (Alongi G) Hospice e Cure Palliative, ASP 1di Agrigento, Agrigento, Italy.; (Amato F) UOC Terapia del Dolore e Cure Palliative, Azienda ospedaliera di Cosenza, Cosenza, Italy.; (Consoletti L) Struttura di Medicina del Dolore, Ospedale Universitario "Ospedali Riuniti", Foggia, Italy.; (Lamarca L) UOS Cure Palliative e Terapia Antalgica, Azienda ULSS N. 10 "Veneto Orientale", San Donà di Piave, VE, Italy.; (Liguori S) USC Cure Palliative Terapia del Dolore, Azienda Ospedaliera Papa Giovanni XXIII, Bergamo, Italy.; (Lo Presti C) UOD Terapia del Dolore e Cure Palliative, ACO San Filippo Neri, ASLRME, Rome, Italy.; (Maione A) Terapia antalgica e Cure Palliative, Presidio Ospedaliero "S. Maria della Pietà", Nola, NA, Italy.; (Mameli S) SC Terapia del Dolore, Presidio Ospedaliero "A. Businco", Cagliari, Italy.; (Marinangeli F) Scuola di Specializzazione di Anestesia, Rianimazione e Terapia Intensiva, Università dell'Aquila, L'Aquila, Italy.; (Marulli S) OC Anestesia, Rianimazione e Terapia Iperbarica, UOS-I Gruppo Operatorio, Ospedale "Vito Fazzi", Lecce, Italy.; (Minotti V) SC Oncologia Medica, Azienda Ospedaliera "S.M. della Misericordia", Perugia, Italy.; (Miotti D) UO Cure Palliative e Terapia del Dolore, Fondazione Salvatore Maugeri-IRCCS, Pavia, Italy.; (Montanari L) UO Semplice Cure Palliative, Ravenna, Italy.; (Moruzzi G) UOS Hospice, Azienda Sanitaria Provinciale di Siracusa, Siracuse, Italy.; (Palermo S) UOC Terapia Antalgica, IRCCS San Martino-IST, Genoa, Italy.; (Parolini M) UOC Anestesia e

Rianimazione B, Azienda Universitaria integrata di Verona, Verona, Italy.; (Poli P) UO Terapia del Dolore, Azienda Ospedaliero-Universitaria Pisana, Pisa, Italy.; (Tirelli W) Centro di Terapia del Dolore, Hospice "Fondazione Roma Sanità", Rome, Italy.; (Valle A) Fondazione FARO, Turin, Italy.; (Romualdi P) Dipartimento di Farmacia e Biotecnologie, Alma mater studiorum, Università di Bologna, Bologna, Italy. (Working Group Nientemale DEI) Working Group Nientemale DEI; (Vellucci R) SOD Cure Palliative e Terapia del Dolore, Ospedale Universitario Careggi, Florence, Italy. renato.vellucci@gmail.com.; (Fanelli G) SC Anestesia, Rianimazione e Terapia Antalgica, Azienda Ospedaliero-Universitaria di Parma, Parma, Italy.; (Pannuti R) Fondazione ANT Italia Onlus, Andria, Italy.; (Peruselli C) SC Cure Palliative, Ospedale di Biella, Ponderano, BI, Italy.; (Adamo S) UO Terapia del Dolore, ARNAS Civico di Palermo, Palermo, Italy.; (Alongi G) Hospice e Cure Palliative, ASP 1di Agrigento, Agrigento, Italy.; (Amato F) UOC Terapia del Dolore e Cure Palliative, Azienda ospedaliera di Cosenza, Cosenza, Italy.; (Consoletti L) Struttura di Medicina del Dolore, Ospedale Universitario "Ospedali Riuniti", Foggia, Italy.; (Lamarca L) UOS Cure Palliative e Terapia Antalgica, Azienda ULSS N. 10 "Veneto Orientale", San Donà di Piave, VE, Italy.; (Liguori S) USC Cure Palliative Terapia del Dolore, Azienda Ospedaliera Papa Giovanni XXIII, Bergamo, Italy.; (Lo Presti C) UOD Terapia del Dolore e Cure Palliative, ACO San Filippo Neri, ASLRME, Rome, Italy.; (Maione A) Terapia antalgica e Cure Palliative, Presidio Ospedaliero "S. Maria della Pietà", Nola, NA, Italy.; (Mameli S) SC Terapia del Dolore, Presidio Ospedaliero "A. Businco", Cagliari, Italy.; (Marinangeli F) Scuola di Specializzazione di Anestesia, Rianimazione e Terapia Intensiva, Università dell'Aquila, L'Aquila, Italy.; (Marulli S) OC Anestesia, Rianimazione e Terapia Iperbarica, UOS-I Gruppo Operatorio, Ospedale "Vito Fazzi", Lecce, Italy.; (Minotti V) SC Oncologia Medica, Azienda Ospedaliera "S.M. della Misericordia", Perugia, Italy.; (Miotti D) UO Cure Palliative e Terapia del Dolore, Fondazione Salvatore Maugeri-IRCCS, Pavia, Italy.; (Montanari L) UO Semplice Cure Palliative, Ravenna, Italy.; (Moruzzi G) UOS Hospice, Azienda Sanitaria Provinciale di Siracusa, Siracuse, Italy.; (Palermo S) UOC Terapia Antalgica, IRCCS San Martino-IST, Genoa, Italy.; (Parolini M) UOC Anestesia e
Rianimazione B, Azienda Universitaria integrata di Verona, Verona, Italy.; (Poli P) UO Terapia del Dolore, Azienda Ospedaliero-Universitaria Pisana, Pisa, Italy.; (Tirelli W) Centro di Terapia del Dolore, Hospice "Fondazione Roma Sanità", Rome, Italy.; (Valle A) Fondazione FARO, Turin, Italy.; (Romualdi P) Dipartimento di Farmacia e Biotecnologie, Alma mater studiorum, Università di Bologna, Bologna, Italy. (Working Group Nientemale DEI) Working Group Nientemale DEI; (Vellucci R) SOD Cure Palliative e Terapia del Dolore, Ospedale Universitario Careggi, Florence, Italy. renato.vellucci@gmail.com.; (Fanelli G) SC Anestesia, Rianimazione e Terapia Antalgica, Azienda Ospedaliero-Universitaria di Parma, Parma, Italy.; (Pannuti R) Fondazione ANT Italia Onlus, Andria, Italy.; (Peruselli C) SC Cure Palliative, Ospedale di Biella, Ponderano, BI, Italy.; (Adamo S) UO Terapia del Dolore, ARNAS Civico di Palermo, Palermo, Italy.; (Alongi G) Hospice e Cure Palliative, ASP 1di Agrigento, Agrigento, Italy.; (Amato F) UOC Terapia del Dolore e Cure Palliative, Azienda ospedaliera di Cosenza, Cosenza, Italy.; (Consoletti L) Struttura di Medicina del Dolore, Ospedale Universitario "Ospedali Riuniti", Foggia, Italy.; (Lamarca L) UOS Cure Palliative e Terapia Antalgica, Azienda ULSS N. 10 "Veneto Orientale", San Donà di Piave, VE, Italy.; (Liguori S) USC Cure Palliative Terapia del Dolore, Azienda Ospedaliera Papa Giovanni XXIII, Bergamo, Italy.; (Lo Presti C) UOD Terapia del Dolore e Cure Palliative, ACO San Filippo Neri, ASLRME, Rome, Italy.; (Maione A) Terapia antalgica e Cure Palliative, Presidio Ospedaliero "S. Maria della

Pietà", Nola, NA, Italy.; (Mameli S) SC Terapia del Dolore, Presidio Ospedaliero "A. Businco", Cagliari, Italy.; (Marinangeli F) Scuola di Specializzazione di Anestesia, Rianimazione e Terapia Intensiva, Università dell'Aquila, L'Aquila, Italy.; (Marulli S) OC Anestesia, Rianimazione e Terapia Iperbarica, UOS-I Gruppo Operatorio, Ospedale "Vito Fazzi", Lecce, Italy.; (Minotti V) SC Oncologia Medica, Azienda Ospedaliera "S.M. della Misericordia", Perugia, Italy.; (Miotti D) UO Cure Palliative e Terapia del Dolore, Fondazione Salvatore Maugeri-IRCCS, Pavia, Italy.; (Montanari L) UO Semplice Cure Palliative, Ravenna, Italy.; (Moruzzi G) UOS Hospice, Azienda Sanitaria Provinciale di Siracusa, Siracuse, Italy.; (Palermo S) UOC Terapia Antalgica, IRCCS San Martino-IST, Genoa, Italy.; (Parolini M) UOC Anestesia e Rianimazione B, Azienda Universitaria integrata di Verona, Verona, Italy.; (Poli P) UO Terapia del Dolore, Azienda Ospedaliero-Universitaria Pisana, Pisa, Italy.; (Tirelli W) Centro di Terapia del Dolore, Hospice "Fondazione Roma Sanità", Rome, Italy.; (Valle A) Fondazione FARO, Turin, Italy.; (Romualdi P) Dipartimento di Farmacia e Biotecnologie, Alma mater studiorum, Università di Bologna, Bologna, Italy.
(Working Group Nientemale DEI) Working Group Nientemale DEI; (Vellucci R) SOD Cure Palliative e Terapia del Dolore, Ospedale Universitario Careggi, Florence, Italy. renato.vellucci@gmail.com.; (Fanelli G) SC Anestesia, Rianimazione e Terapia Antalgica, Azienda Ospedaliero-Universitaria di Parma, Parma, Italy.; (Pannuti R) Fondazione ANT Italia Onlus, Andria, Italy.; (Peruselli C) SC Cure Palliative, Ospedale di Biella, Ponderano, BI, Italy.; (Adamo S) UO Terapia del Dolore, ARNAS Civico di Palermo, Palermo, Italy.; (Alongi G) Hospice e Cure Palliative, ASP 1di Agrigento, Agrigento, Italy.; (Amato F) UOC Terapia del Dolore e Cure Palliative, Azienda ospedaliera di Cosenza, Cosenza, Italy.; (Consoletti L) Struttura di Medicina del Dolore, Ospedale Universitario "Ospedali Riuniti", Foggia, Italy.; (Lamarca L) UOS Cure Palliative e Terapia Antalgica, Azienda ULSS N. 10 "Veneto Orientale", San Donà di Piave, VE, Italy.; (Liguori S) USC Cure Palliative Terapia del Dolore, Azienda Ospedaliera Papa Giovanni XXIII, Bergamo, Italy.; (Lo Presti C) UOD Terapia del Dolore e Cure Palliative, ACO San Filippo Neri, ASLRME, Rome, Italy.; (Maione A) Terapia antalgica e Cure Palliative, Presidio Ospedaliero "S. Maria della Pietà", Nola, NA, Italy.; (Mameli S) SC Terapia del Dolore, Presidio Ospedaliero "A. Businco", Cagliari, Italy.; (Marinangeli F) Scuola di Specializzazione di Anestesia, Rianimazione e Terapia Intensiva, Università dell'Aquila, L'Aquila, Italy.; (Marulli S) OC Anestesia, Rianimazione e Terapia Iperbarica, UOS-I Gruppo Operatorio, Ospedale "Vito Fazzi", Lecce, Italy.; (Minotti V) SC Oncologia Medica, Azienda Ospedaliera "S.M. della Misericordia", Perugia, Italy.; (Miotti D) UO Cure Palliative e Terapia del Dolore, Fondazione Salvatore Maugeri-IRCCS, Pavia, Italy.; (Montanari L) UO Semplice Cure Palliative, Ravenna, Italy.; (Moruzzi G) UOS Hospice, Azienda Sanitaria Provinciale di Siracusa, Siracuse, Italy.; (Palermo S) UOC Terapia Antalgica, IRCCS San Martino-IST, Genoa, Italy.; (Parolini M) UOC Anestesia e Rianimazione B, Azienda Universitaria integrata di Verona, Verona, Italy.; (Poli P) UO Terapia del Dolore, Azienda Ospedaliero-Universitaria Pisana, Pisa, Italy.; (Tirelli W) Centro di Terapia del Dolore, Hospice "Fondazione Roma Sanità", Rome, Italy.; (Valle A) Fondazione FARO, Turin, Italy.; (Romualdi P) Dipartimento di Farmacia e Biotecnologie, Alma mater studiorum, Università di Bologna, Bologna, Italy.
(Working Group Nientemale DEI) Working Group Nientemale DEI; (Vellucci R) SOD Cure Palliative e Terapia del Dolore, Ospedale Universitario Careggi, Florence, Italy. renato.vellucci@gmail.com.; (Fanelli G) SC Anestesia, Rianimazione e Terapia Antalgica, Azienda Ospedaliero-Universitaria di Parma, Parma, Italy.; (Pannuti R) Fondazione ANT Italia Onlus, Andria, Italy.; (Peruselli C) SC Cure Palliative,

Ospedale di Biella, Ponderano, BI, Italy.; (Adamo S) UO Terapia del Dolore, ARNAS Civico di Palermo, Palermo, Italy.; (Alongi G) Hospice e Cure Palliative, ASP 1di Agrigento, Agrigento, Italy.; (Amato F) UOC Terapia del Dolore e Cure Palliative, Azienda ospedaliera di Cosenza, Cosenza, Italy.; (Consoletti L) Struttura di Medicina del Dolore, Ospedale Universitario "Ospedali Riuniti", Foggia, Italy.; (Lamarca L) UOS Cure Palliative e Terapia Antalgica, Azienda ULSS N. 10 "Veneto Orientale", San Donà di Piave, VE, Italy.; (Liguori S) USC Cure Palliative Terapia del Dolore, Azienda Ospedaliera Papa Giovanni XXIII, Bergamo, Italy.; (Lo Presti C) UOD Terapia del Dolore e Cure Palliative, ACO San Filippo Neri, ASLRME, Rome, Italy.; (Maione A) Terapia antalgica e Cure Palliative, Presidio Ospedaliero "S. Maria della Pietà", Nola, NA, Italy.; (Mameli S) SC Terapia del Dolore, Presidio Ospedaliero "A. Businco", Cagliari, Italy.; (Marinangeli F) Scuola di Specializzazione di Anestesia, Rianimazione e Terapia Intensiva, Università dell'Aquila, L'Aquila, Italy.; (Marulli S) OC Anestesia, Rianimazione e Terapia Iperbarica, UOS-I Gruppo Operatorio, Ospedale "Vito Fazzi", Lecce, Italy.; (Minotti V) SC Oncologia Medica, Azienda Ospedaliera "S.M. della Misericordia", Perugia, Italy.; (Miotti D) UO Cure Palliative e Terapia del Dolore, Fondazione Salvatore Maugeri-IRCCS, Pavia, Italy.; (Montanari L) UO Semplice Cure Palliative, Ravenna, Italy.; (Moruzzi G) UOS Hospice, Azienda Sanitaria Provinciale di Siracusa, Siracuse, Italy.; (Palermo S) UOC Terapia Antalgica, IRCCS San Martino-IST, Genoa, Italy.; (Parolini M) UOC Anestesia e Rianimazione B, Azienda Universitaria integrata di Verona, Verona, Italy.; (Poli P) UO Terapia del Dolore, Azienda Ospedaliero-Universitaria Pisana, Pisa, Italy.; (Tirelli W) Centro di Terapia del Dolore, Hospice "Fondazione Roma Sanità", Rome, Italy.; (Valle A) Fondazione FARO, Turin, Italy.; (Romualdi P) Dipartimento di Farmacia e Biotecnologie, Alma mater studiorum, Università di Bologna, Bologna, Italy. (Working Group Nientemale DEI) Working Group Nientemale DEI; (Vellucci R) SOD Cure Palliative e Terapia del Dolore, Ospedale Universitario Careggi, Florence, Italy. renato.vellucci@gmail.com.; (Fanelli G) SC Anestesia, Rianimazione e Terapia Antalgica, Azienda Ospedaliero-Universitaria di Parma, Parma, Italy.; (Pannuti R) Fondazione ANT Italia Onlus, Andria, Italy.; (Peruselli C) SC Cure Palliative,
Ospedale di Biella, Ponderano, BI, Italy.; (Adamo S) UO Terapia del Dolore, ARNAS Civico di Palermo, Palermo, Italy.; (Alongi G) Hospice e Cure Palliative, ASP 1di Agrigento, Agrigento, Italy.; (Amato F) UOC Terapia del Dolore e Cure Palliative, Azienda ospedaliera di Cosenza, Cosenza, Italy.; (Consoletti L) Struttura di Medicina del Dolore, Ospedale Universitario "Ospedali Riuniti", Foggia, Italy.; (Lamarca L) UOS Cure Palliative e Terapia Antalgica, Azienda ULSS N. 10 "Veneto Orientale", San Donà di Piave, VE, Italy.; (Liguori S) USC Cure Palliative Terapia del Dolore, Azienda Ospedaliera Papa Giovanni XXIII, Bergamo, Italy.; (Lo Presti C) UOD Terapia del Dolore e Cure Palliative, ACO San Filippo Neri, ASLRME, Rome, Italy.; (Maione A) Terapia antalgica e Cure Palliative, Presidio Ospedaliero "S. Maria della Pietà", Nola, NA, Italy.; (Mameli S) SC Terapia del Dolore, Presidio Ospedaliero "A. Businco", Cagliari, Italy.; (Marinangeli F) Scuola di Specializzazione di Anestesia, Rianimazione e Terapia Intensiva, Università dell'Aquila, L'Aquila, Italy.; (Marulli S) OC Anestesia, Rianimazione e Terapia Iperbarica, UOS-I Gruppo Operatorio, Ospedale "Vito Fazzi", Lecce, Italy.; (Minotti V) SC Oncologia Medica, Azienda Ospedaliera "S.M. della Misericordia", Perugia, Italy.; (Miotti D) UO Cure Palliative e Terapia del Dolore, Fondazione Salvatore Maugeri-IRCCS, Pavia, Italy.; (Montanari L) UO Semplice Cure Palliative, Ravenna, Italy.; (Moruzzi G) UOS Hospice, Azienda Sanitaria Provinciale di Siracusa, Siracuse, Italy.; (Palermo S) UOC Terapia Antalgica, IRCCS San Martino-IST, Genoa, Italy.; (Parolini M) UOC Anestesia e

Rianimazione B, Azienda Universitaria integrata di Verona, Verona, Italy.; (Poli P) UO Terapia del Dolore, Azienda Ospedaliero-Universitaria Pisana, Pisa, Italy.; (Tirelli W) Centro di Terapia del Dolore, Hospice "Fondazione Roma Sanità", Rome, Italy.; (Valle A) Fondazione FARO, Turin, Italy.; (Romualdi P) Dipartimento di Farmacia e Biotecnologie, Alma mater studiorum, Università di Bologna, Bologna, Italy.
Database: PubMed

105. Knowledge and Beliefs about Chronic Non Cancer Pain Management for Family Medicine Group Nurses.

Author(s): Bergeron DA; Bourgault P; Gallagher F

Source: Pain management nursing : official journal of the American Society of Pain Management Nurses; Dec 2015; vol. 16 (no. 6); p. 951-958

Publication Date: Dec 2015

Publication Type(s): Journal Article

DOI: http://dx.doi.org/10.1016/j.pmn.2015.09.001

ISSN: 1532-8635

Place of Publication: United States

PubMedID: 26697819

Accession Number: 26697819

Available at Pain management nursing : official journal of the American Society of Pain Management Nurses - from Unpaywall

Abstract: To provide effective care for chronic pain sufferers, nurses must have a knowledge of chronic pain management. In Quebec, nurses working in Family Medicine Groups (FMGs) could play a major role in helping patients with chronic noncancer pain (CNCP); however, the extent of their knowledge about CNCP management is unknown. The primary goal of this study was to explore the knowledge and beliefs of FMG nurses about CNCP management. The secondary goal was to explore the obstacles seen by these nurses as preventing them from performing CNCP management. We used a mixed-methods design with quantitative preponderance. Fifty-three FMG nurses answered a self-administered mail-in questionnaire. A rigorous data collection method was used. FMG nurses have suboptimal knowledge about CNCP management. They identify their lack of training and lack of knowledge as major obstacles to conducting pain management interventions. There is a need for pain management training specifically designed around the realities of FMG nursing.

Institutions:
(Bergeron DA) Department of Nursing, Université du Québec à Rimouski, Rimouski, Quebec, Canada. Electronic address: dave.a.bergeron@usherbrooke.ca.; (Bourgault P) School of Nursing, Université de Sherbrooke, Sherbrooke, Quebec, Canada.; (Gallagher F) School of Nursing, Université de Sherbrooke, Sherbrooke, Quebec, Canada.
(Bergeron DA) Department of Nursing, Université du Québec à Rimouski, Rimouski, Quebec, Canada. Electronic address: dave.a.bergeron@usherbrooke.ca.; (Bourgault P) School of Nursing, Université de Sherbrooke, Sherbrooke, Quebec, Canada.; (Gallagher F) School of Nursing, Université de Sherbrooke, Sherbrooke, Quebec, Canada.

(Bergeron DA) Department of Nursing, Université du Québec à Rimouski, Rimouski, Quebec, Canada. Electronic address: dave.a.bergeron@usherbrooke.ca.; (Bourgault P) School of Nursing, Université de Sherbrooke, Sherbrooke, Quebec, Canada.; (Gallagher F) School of Nursing, Université de Sherbrooke, Sherbrooke, Quebec, Canada.
Database: PubMed

106. Diagnosis and management of breakthrough cancer pain: Have all the questions been resolved? A Delphi-based consensus assessment (DOIRON).

Author(s): Porta-Sales J; Pérez C; Escobar Y; Martínez V

Source: Clinical & translational oncology : official publication of the Federation of Spanish Oncology Societies and of the National Cancer Institute of Mexico; Sep 2016; vol. 18 (no. 9); p. 945-954

Publication Date: Sep 2016

Publication Type(s): Journal Article

DOI: http://dx.doi.org/10.1007/s12094-015-1468-7

ISSN: 1699-3055

Place of Publication: Italy

PubMedID: 26693731

Accession Number: 26693731

Keywords: Subject Terms: Breakthrough cancer pain; Cancer pain; Consensus; Delphi method; Pain

Abstract:OBJECTIVE: To ascertain the level of agreement and achieve a consensus among cancer pain specialists in Spain with regard to the optimal definition, diagnosis, and management of breakthrough cancer pain (BTcP).DESIGN: Two-round Delphi methodology survey (February-May 2013) using seven-point Likert scales (ranging from 1 "strongly disagree" to 7 "strongly agree") was carried out. Mean scores >5 or <3 indicated, respectively, agreement or disagreement. Scores from 3 to 5 indicated no consensus.RESULTS: A total of 126 experienced specialists were surveyed. Response rates were 68 % in round 1 and 90 % in round 2. Agreement (mean Likert score) was strongest for the proposed BTcP definition (6.6), the use of oral (6.1), and intranasal (6.0) transmucosal fentanyl, the need for early assessment after BTcP treatment initiation, and the need to improve staff knowledge of BTcP. Broad agreement was also reached regarding the need to systematically screen all cancer patients for BTcP (5.9). Most respondents (82 %) considered strong opioids to be appropriate treatment. In contrast, no consensus was reached regarding strong opioid treatment for baseline pain as a prerequisite for BTcP diagnosis.CONCLUSIONS: Consensus was strong for most treatment, and diagnostic aspects were evaluated in the study. However, several important issues remain unresolved, particularly whether the diagnostic criteria must include strong opioids for background pain. Nurses' awareness and understanding of BTcP was considered insufficient, and more training is needed in this area. Overall, agreement among specialists was good, but more work is needed to better define the optimal diagnostic features and treatments for this condition.

Institutions:

(Porta-Sales J) Palliative Care Service, Institut Català d'Oncologia (ICO), Bellvitge Biomedical Research Institute (IDIBELL), WeCare Chair: End of Life Care, Institut Català d'Oncologia, Barcelona, Spain.; (Pérez C) Pain Clinic, Hospital Universitario de la Princesa, C/Diego de León 62, 28002, Madrid, Spain.; (Escobar Y) Department of Medical Oncology, Hospital General Universitario Gregorio Marañón, Madrid, Spain.; (Martínez V) Medical Manager Takeda, Madrid, Spain. Vicente.martinez@takeda.com.
(Porta-Sales J) Palliative Care Service, Institut Català d'Oncologia (ICO), Bellvitge Biomedical Research Institute (IDIBELL), WeCare Chair: End of Life Care, Institut Català d'Oncologia, Barcelona, Spain.; (Pérez C) Pain Clinic, Hospital Universitario de la Princesa, C/Diego de León 62, 28002, Madrid, Spain.; (Escobar Y) Department of Medical Oncology, Hospital General Universitario Gregorio Marañón, Madrid, Spain.; (Martínez V) Medical Manager Takeda, Madrid, Spain. Vicente.martinez@takeda.com.
(Porta-Sales J) Palliative Care Service, Institut Català d'Oncologia (ICO), Bellvitge Biomedical Research Institute (IDIBELL), WeCare Chair: End of Life Care, Institut Català d'Oncologia, Barcelona, Spain.; (Pérez C) Pain Clinic, Hospital Universitario de la Princesa, C/Diego de León 62, 28002, Madrid, Spain.; (Escobar Y) Department of Medical Oncology, Hospital General Universitario Gregorio Marañón, Madrid, Spain.; (Martínez V) Medical Manager Takeda, Madrid, Spain. Vicente.martinez@takeda.com.
(Porta-Sales J) Palliative Care Service, Institut Català d'Oncologia (ICO), Bellvitge Biomedical Research Institute (IDIBELL), WeCare Chair: End of Life Care, Institut Català d'Oncologia, Barcelona, Spain.; (Pérez C) Pain Clinic, Hospital Universitario de la Princesa, C/Diego de León 62, 28002, Madrid, Spain.; (Escobar Y) Department of Medical Oncology, Hospital General Universitario Gregorio Marañón, Madrid, Spain.; (Martínez V) Medical Manager Takeda, Madrid, Spain. Vicente.martinez@takeda.com.
Database: PubMed

107. Oncology Nursing Certification: Relation to Nurses' Knowledge and Attitudes About Pain, Patient-Reported Pain Care Quality, and Pain Outcomes.

Author(s): Beck SL; Brant JM; Donohue R; Smith EM; Towsley G; Berry PH; Guo JW; Al-Qaaydeh S; Pett MA; Donaldson G

Source: Oncology nursing forum; Jan 2016; vol. 43 (no. 1); p. 67-76

Publication Date: Jan 2016

Publication Type(s): Comparative Study; Journal Article; Research Support, Non-U.S. Gov't

DOI: http://dx.doi.org/10.1188/16.ONF.67-76

ISSN: 1538-0688

Place of Publication: United States

PubMedID: 26679446

Accession Number: 26679446

Available at Oncology Nursing Forum - from Ovid (Journals @ Ovid)

Available at Oncology Nursing Forum - from EBSCO (CINAHL Complete)

Available at Oncology Nursing Forum - from ProQuest (MEDLINE with Full Text) - NHS Version

Available at Oncology Nursing Forum - from ProQuest (Health Research Premium) - NHS Version

Available at Oncology Nursing Forum - from David Adams Library Journals Collection Local Print Collection [location] : David Adams Library.

Keywords: Subject Terms: nurse certification; oncology; oncology nurses; outcomes; pain; pain attitudes and knowledge; pain care quality

Abstract:PURPOSE/OBJECTIVES: To (a) compare pain knowledge and attitudes between nurses with oncology certified nurse (OCN®) status, non-OCN®-certified nurses, and nurses ineligible for certification and (b) examine the relationships among OCN® status, nurses' knowledge and attitudes about pain, patient-reported quality of nursing pain care, and pain outcomes. .DESIGN: Prospective, correlational survey design. Patients were nested within nurses. .SETTING: Six inpatient oncology units in three hospitals.SAMPLE: 91 nurses in three states (28 OCN®-certified nurses, 37 noncertified nurses, and 26 not eligible for certification). Certification status was validated for 105 nurses who were matched with a sample of 320 patients. .METHODS: Nurses completed a survey, and matched adult patients who were experiencing pain rated their pain care quality and pain experience during the past shift. .MAIN RESEARCH VARIABLES: Demographic characteristics, certification status, and responses to the Nurse Knowledge and Attitudes Survey Regarding Pain (NKASRP), Pain Care Quality Survey-Nursing, and modified Brief Pain Inventory (Short Form). .FINDINGS: OCN®-certified nurses scored significantly higher on the NKASRP (82% correct) compared to non-OCN® eligible nurses (76%) and non-OCN® ineligible nurses (74%) (p 0.05). .CONCLUSIONS: OCN®-certified nurses' knowledge and attitudes related to pain management were superior to noncertified nurses. Neither knowledge and attitudes nor OCN® status were associated with pain care quality or pain outcomes. .IMPLICATIONS FOR NURSING: Knowledge is necessary but insufficient to improve patient outcomes; providing optimal pain care requires action. Sustained efforts to improve cancer pain management are indicated.

Institutions:
(Beck SL) University of Utah, Salt Lake City.; (Brant JM) Cancer Center of Acadiana.; (Donohue R) Dartmouth-Hitchcock Medical Center, University of Utah, Salt Lake City.; (Smith EM) University of Utah.; (Towsley G) Oregon Health and Science University.; (Berry PH) University of Utah.; (Guo JW) University of Utah.; (Al-Qaaydeh S) College of Nursing, University of Utah in Salt Lake City.; (Pett MA) University of Utah.
(Beck SL) University of Utah, Salt Lake City.; (Brant JM) Cancer Center of Acadiana.; (Donohue R) Dartmouth-Hitchcock Medical Center, University of Utah, Salt Lake City.; (Smith EM) University of Utah.; (Towsley G) Oregon Health and Science University.; (Berry PH) University of Utah.; (Guo JW) University of Utah.; (Al-Qaaydeh S) College of Nursing, University of Utah in Salt Lake City.; (Pett MA) University of Utah.
(Beck SL) University of Utah, Salt Lake City.; (Brant JM) Cancer Center of Acadiana.; (Donohue R) Dartmouth-Hitchcock Medical Center, University of Utah, Salt Lake City.; (Smith EM) University of Utah.; (Towsley G) Oregon Health and Science University.; (Berry PH) University of Utah.; (Guo JW) University of Utah.; (Al-

Qaaydeh S) College of Nursing, University of Utah in Salt Lake City.; (Pett MA) University of Utah.
(Beck SL) University of Utah, Salt Lake City.; (Brant JM) Cancer Center of Acadiana.; (Donohue R) Dartmouth-Hitchcock Medical Center, University of Utah, Salt Lake City.; (Smith EM) University of Utah.; (Towsley G) Oregon Health and Science University.; (Berry PH) University of Utah.; (Guo JW) University of Utah.; (Al-Qaaydeh S) College of Nursing, University of Utah in Salt Lake City.; (Pett MA) University of Utah.
(Beck SL) University of Utah, Salt Lake City.; (Brant JM) Cancer Center of Acadiana.; (Donohue R) Dartmouth-Hitchcock Medical Center, University of Utah, Salt Lake City.; (Smith EM) University of Utah.; (Towsley G) Oregon Health and Science University.; (Berry PH) University of Utah.; (Guo JW) University of Utah.; (Al-Qaaydeh S) College of Nursing, University of Utah in Salt Lake City.; (Pett MA) University of Utah.
(Beck SL) University of Utah, Salt Lake City.; (Brant JM) Cancer Center of Acadiana.; (Donohue R) Dartmouth-Hitchcock Medical Center, University of Utah, Salt Lake City.; (Smith EM) University of Utah.; (Towsley G) Oregon Health and Science University.; (Berry PH) University of Utah.; (Guo JW) University of Utah.; (Al-Qaaydeh S) College of Nursing, University of Utah in Salt Lake City.; (Pett MA) University of Utah.
(Beck SL) University of Utah, Salt Lake City.; (Brant JM) Cancer Center of Acadiana.; (Donohue R) Dartmouth-Hitchcock Medical Center, University of Utah, Salt Lake City.; (Smith EM) University of Utah.; (Towsley G) Oregon Health and Science University.; (Berry PH) University of Utah.; (Guo JW) University of Utah.; (Al-Qaaydeh S) College of Nursing, University of Utah in Salt Lake City.; (Pett MA) University of Utah.
(Beck SL) University of Utah, Salt Lake City.; (Brant JM) Cancer Center of Acadiana.; (Donohue R) Dartmouth-Hitchcock Medical Center, University of Utah, Salt Lake City.; (Smith EM) University of Utah.; (Towsley G) Oregon Health and Science University.; (Berry PH) University of Utah.; (Guo JW) University of Utah.; (Al-Qaaydeh S) College of Nursing, University of Utah in Salt Lake City.; (Pett MA) University of Utah.
(Beck SL) University of Utah, Salt Lake City.; (Brant JM) Cancer Center of Acadiana.; (Donohue R) Dartmouth-Hitchcock Medical Center, University of Utah, Salt Lake City.; (Smith EM) University of Utah.; (Towsley G) Oregon Health and Science University.; (Berry PH) University of Utah.; (Guo JW) University of Utah.; (Al-Qaaydeh S) College of Nursing, University of Utah in Salt Lake City.; (Pett MA) University of Utah.
Database: PubMed

108. Comparison of Oncology Patients' and Their Family Caregivers' Attitudes and Concerns Toward Pain and Pain Management.

Author(s): Valeberg BT; Miaskowski C; Paul SM; Rustøen T

Source: Cancer nursing; 2016; vol. 39 (no. 4); p. 328-334

Publication Date: 2016

Publication Type(s): Journal Article

DOI: http://dx.doi.org/10.1097/NCC.0000000000000319

ISSN: 1538-9804
Place of Publication: United States
PubMedID: 26632879
Accession Number: 26632879
Available at Cancer nursing - from Ovid (Journals @ Ovid)
Available at Cancer nursing - from Unpaywall
Abstract:BACKGROUND: Both cancer patients and their family caregivers (FCs) report concerns about pain and pain management. When dyads share appraisal of the illness context, they may experience better dyadic adjustment.OBJECTIVE: The aim of this study was to compare oncology outpatients' and their FCs' attitudes and concerns toward pain and pain management.METHODS: In a cross-sectional study, outpatients with pain (n = 71) and their FCs completed the Barriers Questionnaire II, the Brief Pain Inventory, and information about demographic characteristics. Correlations and paired-samples t tests were calculated to evaluate agreement and differences in barrier scores between the patients and their FCs.RESULTS: Congruence was found in patients' and FCs' beliefs about pain and the use of analgesics on 4 of 7 subscales (ie, tolerance, immune system, side effects, distract the medical doctor) and on the Barriers Questionnaire II total score. Both patients and their FCs were most concerned about addiction.CONCLUSIONS: The concerns that both patients and their FCs have about pain and the use of analgesics may act as barriers to effective pain management. Fear of addiction may be an important barrier to cancer pain management. Only small differences were found in concerns between the patients and their FCs.IMPLICATIONS FOR PRACTICE: Patients and FCs need education about perceived barriers to effective pain management. They should be coached together to maintain or increase the congruence between them.
Institutions:
(Valeberg BT) Author Affiliations: Faculty of Nursing, College of Applied Sciences, Oslo and Akershus University, Norway (Dr Valeberg); Department of Physiological Nursing, University of California, San Francisco (Drs Miaskowski and Paul); Division of Emergencies and Critical Care, Department of Research and Development, Oslo University Hospital, and Institute of Health and Society, University of Oslo, Norway (Dr Rustøen).
Database: PubMed

109. Oncology Nurses Knowledge and Attitudes Regarding Cancer Pain Management.

Author(s): Shahriary S; Shiryazdi SM; Shiryazdi SA; Arjomandi A; Haghighi F; Vakili FM; Mostafaie N
Source: Asian Pacific journal of cancer prevention : APJCP; 2015; vol. 16 (no. 17); p. 7501-7506
Publication Date: 2015
Publication Type(s): Journal Article
DOI: http://dx.doi.org/10.7314/apjcp.2015.16.17.7501
ISSN: 2476-762X
Place of Publication: Thailand

PubMedID: 26625752

Accession Number: 26625752

Available at Asian Pacific journal of cancer prevention : APJCP - from Unpaywall

Abstract:BACKGROUND: Oncology nurses play a crucial role in cancer pain management and must be highly informed to ensure their effective practice in the cancer setting. The aim of this study was to determine the baseline level of knowledge and attitudes of oncology nurses regarding cancer pain management.MATERIALS AND METHODS: A cross-sectional survey research design was employed. The sample comprised 58 cancer nurses working in Shahid Sadoughi hospital, Yazd, Iran. The "Nurses Knowledge and Attitudes Survey Regarding Pain" (NKAS) tool and a demographic form were utilized to ascertain the knowledge and attitudes of oncology nurses working in oncology settings.RESULTS: The average correct response rate for oncology nurses was 66.6%, ranging from 12.1% to 94.8%. The nurses mean score on the knowledge and attitudes survey regarding pain management was 28.5%. Results revealed that the mean percentage score overall was 65.7%. Only 8.6% of nurse participants obtained a passing score of 75% or greater. Widespread knowledge deficits and poor attitudes were noted in this study, particularly regard pharmacological management of pain.CONCLUSIONS: The present study provides important information about knowledge deficits in pain management among oncology nurses and limited training regarding pain management. Our results support the universal concern of inadequate knowledge and attitudes of nurses regarding cancer pain. It is suggested educational and quality improvement initiatives in pain management could enhance nurses knowledge in the area of pain and possibly improve practice.

Institutions:
(Shahriary S) Baghaie-pour Clinic, Shahid Sadoughi University of Medical Sciences and Health Services, Yazd, Iran E-mail : seyedmostafashiryazdi@gmail.com, seyedmostafashiryazdi@gmail.com.
Database: PubMed

110. Interventions for Nurse-Related Barriers in Cancer Pain Management.

Author(s): Bartoszczyk DA; Gilbertson-White S

Source: Oncology nursing forum; 2015; vol. 42 (no. 6); p. 634-641

Publication Date: 2015

Publication Type(s): Journal Article; Research Support, N.I.H., Extramural; Review

DOI: http://dx.doi.org/10.1188/15.ONF.634-641

ISSN: 1538-0688

Place of Publication: United States

PubMedID: 26488832

Accession Number: 26488832

Available at Oncology nursing forum - from Ovid (Journals @ Ovid)

Available at Oncology nursing forum - from EBSCO (CINAHL Complete)

Available at Oncology nursing forum - from ProQuest (MEDLINE with Full Text) - NHS Version

Available at Oncology nursing forum - from ProQuest (Health Research Premium) - NHS Version

Available at Oncology nursing forum - from David Adams Library Journals Collection Local Print Collection [location] : David Adams Library.

Available at Oncology nursing forum - from Unpaywall

Keywords: Subject Terms: *cancer; *interventions; *literature review; *nurses; *pain

Abstract:PURPOSE/OBJECTIVES: To describe the findings and critique the studies of interventions for nursing staff to improve pain management in adults with cancer.DATA SOURCES: Publications were identified through database searches. Studies that describe interventions to overcome nurse-related barriers in cancer pain management practices were included in this review.DATA SYNTHESIS: Nine studies were found that met the inclusion criteria. All studies were experimental and conducted from 1993-2013.CONCLUSIONS: Increase in knowledge, change of attitudes and behaviors, and good relationships with specialists were found to be influential in overcoming existing nursing barriers to pain management in cancer survivors. Educational interventions are more effective in increasing knowledge than in improving attitudes. Specialists were acknowledged as important resources and role models for nurses, particularly when trust was established between the two.IMPLICATIONS FOR NURSING: A number of interventions have been developed to address healthcare provider barriers. However, scarce literature exists on whether interventions that aim to overcome nurse-related barriers have been successful. This literature review provides critical insights on the effectiveness of interventions aimed to overcome barriers to effective pain management by nurses for adults with cancer .

Institutions:
(Bartoszczyk DA) University of Iowa Hospitals and Clinics.; (Gilbertson-White S) University of Iowa.
(Bartoszczyk DA) University of Iowa Hospitals and Clinics.; (Gilbertson-White S) University of Iowa.
Database: PubMed

111. Australian survey of current practice and guideline use in adult cancer pain assessment and management: The community nurse perspective.

Author(s): Phillips JL; Lovell M; Luckett T; Agar M; Green A; Davidson P

Source: Collegian (Royal College of Nursing, Australia); 2015; vol. 22 (no. 1); p. 33-41

Publication Date: 2015

Publication Type(s): Journal Article; Research Support, Non-U.S. Gov't

DOI: http://dx.doi.org/10.1016/j.colegn.2013.11.002

ISSN: 1322-7696

Place of Publication: Australia

PubMedID: 26285407

Accession Number: 26285407

Abstract:BACKGROUND: Cancer pain remains a major public health concern. Despite effective treatments being available to manage the majority of cancer pain,

this debilitating symptom is frequently under treated. As cancer has becomes a chronic disease a range of health professionals, including community nurses in Australia are increasingly caring for people living with cancer related pain. Yet, little is known about community nurses capacity to assess and manage cancer pain in accordance with best available evidence.OBJECTIVES: This study aimed to: identify the barriers and facilitators to adult cancer pain assessment and management as perceived by Australian health professionals; identify if cancer pain guidelines are currently used; identify barriers and facilitators to guideline use; and establish the need for Australian cancer pain guidelines. This article reports on community nurses' perceptions of managing cancer pain in the community setting.METHODS: A cross-sectional survey was administered online. Invitations were circulated via peak bodies and clinical leaders seeking the views and experiences of health professionals involved in caring for people living with cancer pain. Descriptive statistics were used to summarise the quantitative data, and thematic content analysis were used to describe the qualitative data.RESULTS: Sixty-two community nurses responded to the survey, representing 29% of the total sample. These participants reported high levels of adherence to accepted cancer pain management practices in their workplace, with 71% nominating the Palliative Care Therapeutic Guideline V.3 as being most frequently used to manage community patients' cancer related pain. Key barriers to effective cancer pain management in the community were: difficulties accessing non-pharmacological interventions (89%), lack of coordination by multiple providers (89%), and impact of distance on ability to access pain-related services for patients (86%).CONCLUSION: A range of system, health professional and consumer barriers limit access to best available treatment in the community setting for people with cancer pain. A clinical pathway that gives step-by-step guidance on evidence-based practice along with an evaluation framework may be the best way of enabling community nurses to ensure their patients with cancer related pain have access to best available care.

Database: PubMed

112. A holistic approach to chronic pain management that involves all stakeholders: change is needed.

Author(s): Kress HG; Aldington D; Alon E; Coaccioli S; Collett B; Coluzzi F; Huygen F; Jaksch W; Kalso E; Kocot-Kępska M; Mangas AC; Ferri CM; Mavrocordatos P; Morlion B; Müller-Schwefe G; Nicolaou A; Hernández CP; Sichère P

Source: Current medical research and opinion; 2015; vol. 31 (no. 9); p. 1743-1754

Publication Date: 2015

Publication Type(s): Journal Article; Research Support, Non-U.S. Gov't; Review

DOI: http://dx.doi.org/10.1185/03007995.2015.1072088

ISSN: 1473-4877

Place of Publication: England

PubMedID: 26172982

Accession Number: 26172982

Available at Current medical research and opinion - from Unpaywall

Keywords: Subject Terms: Biopsychosocial model; Chronic pain; Improved training; Multidisciplinary management; Patient-centered approach; Political will; Standardized pain assessment tools

Abstract: Chronic pain affects a large proportion of the population, imposing significant individual distress and a considerable burden on society, yet treatment is not always instituted and/or adequate. Comprehensive multidisciplinary management based on the biopsychosocial model of pain has been shown to be clinically effective and cost-efficient, but is not widely available. A literature review of stakeholder groups revealed many reasons for this, including: i) many patients believe healthcare professionals lack relevant knowledge, and consultations are rushed, ii) general practitioners consider that pain management has a low priority and is under-resourced, iii) pain specialists cite non-adherence to evidence-based treatment, sub-optimal prescribing, and chronic pain not being regarded as a disease in its own right, iv) nurses', pharmacists' and physiotherapists' skills are not fully utilized, and v) psychological therapy is employed infrequently and often too late. Many of the issues relating to physicians could be addressed by improving medical training, both at undergraduate and postgraduate levels - for example, by making pain medicine a compulsory core subject of the undergraduate medical curriculum. This would improve physician/patient communication, increase the use of standardized pain assessment tools, and allow more patients to participate in treatment decisions. Patient care would also benefit from improved training for other multidisciplinary team members; for example, nurses could provide counseling and follow-up support, psychologists offer coping skills training, and physiotherapists have a greater role in rehabilitation. Equally important measures include the widespread adoption of a patient-centered approach, chronic pain being recognized as a disease in its own right, and the development of universal guidelines for managing chronic non-cancer pain. Perhaps the greatest barrier to improvement is lack of political will at both national and international level. Some powerful initiatives and collaborations are currently lobbying policy-making bodies to raise standards and reduce unnecessary pain - it is vital they continue.

Institutions:
(Kress HG) a a Department of Special Anaesthesia and Pain Therapy , Medizinische Universität/AKH Wien , Vienna , Austria.; (Aldington D) b b Hampshire Hospitals NHS Trust , Winchester , Hants , UK.; (Alon E) c c Universitätsspital Zurich , Zurich , Switzerland.; (Coaccioli S) d d Santa Maria General Hospital , Terni , Italy.; (Collett B) e e University Hospitals of Leicester NHS Trust , Leicester , UK.; (Coluzzi F) f f Department of Medical and Surgical Sciences and Biotechnologies , Sapienza University of Rome , Italy.; (Huygen F) g g University Hospital , Rotterdam , The Netherlands.; (Jaksch W) h h Wilhelminenspital der Stadt Wien , Austria.; (Kalso E) i i Pain Clinic, Department of Anaesthesiology, Intensive Care and Pain Medicine, University of Helsinki, and Helsinki University Hospital , Finland.; (Kocot-Kępska M) j j Department of Pain Research and Treatment , Collegium Medicum Jagiellonian University , Kraków , Poland.; (Mangas AC) k k UNIDOR, Centro Hospitalar de Leiria , Leiria , Portugal.; (Ferri CM) l l Hospital General Universitario de Alicante , Spain.; (Mavrocordatos P) m m Clinique Cecil , Lausanne , Switzerland.; (Morlion B) n n University Hospitals Leuven , Belgium.; (Müller-Schwefe G) o o Schmerz- und Palliativzentrum , Göppingen , Germany.; (Nicolaou A) p p St. Georges Hospital , London , UK.; (Hernández CP) q q Hospital Universitario de la Princesa , Madrid , Spain.; (Sichère P) r r Hôpitaux de Saint-Denis , Paris , France.

(Kress HG) a a Department of Special Anaesthesia and Pain Therapy , Medizinische Universität/AKH Wien , Vienna , Austria.; (Aldington D) b b Hampshire Hospitals NHS Trust , Winchester , Hants , UK.; (Alon E) c c Universitätsspital Zurich , Zurich , Switzerland.; (Coaccioli S) d d Santa Maria General Hospital , Terni , Italy.; (Collett B) e e University Hospitals of Leicester NHS Trust , Leicester , UK.; (Coluzzi F) f f Department of Medical and Surgical Sciences and Biotechnologies , Sapienza University of Rome , Italy.; (Huygen F) g g University Hospital , Rotterdam , The Netherlands.; (Jaksch W) h h Wilhelminenspital der Stadt Wien , Austria.; (Kalso E) i i Pain Clinic, Department of Anaesthesiology, Intensive Care and Pain Medicine, University of Helsinki, and Helsinki University Hospital , Finland.; (Kocot-Kępska M) j j Department of Pain Research and Treatment , Collegium Medicum Jagiellonian University , Kraków , Poland.; (Mangas AC) k k UNIDOR, Centro Hospitalar de Leiria , Leiria , Portugal.; (Ferri CM) l l Hospital General Universitario de Alicante , Spain.; (Mavrocordatos P) m m Clinique Cecil , Lausanne , Switzerland.; (Morlion B) n n University Hospitals Leuven , Belgium.; (Müller-Schwefe G) o o Schmerz- und Palliativzentrum , Göppingen , Germany.; (Nicolaou A) p p St. Georges Hospital , London , UK.; (Hernández CP) q q Hospital Universitario de la Princesa , Madrid , Spain.; (Sichère P) r r Hôpitaux de Saint-Denis , Paris , France.
(Kress HG) a a Department of Special Anaesthesia and Pain Therapy , Medizinische Universität/AKH Wien , Vienna , Austria.; (Aldington D) b b Hampshire Hospitals NHS Trust , Winchester , Hants , UK.; (Alon E) c c Universitätsspital Zurich , Zurich , Switzerland.; (Coaccioli S) d d Santa Maria General Hospital , Terni , Italy.; (Collett B) e e University Hospitals of Leicester NHS Trust , Leicester , UK.; (Coluzzi F) f f Department of Medical and Surgical Sciences and Biotechnologies , Sapienza University of Rome , Italy.; (Huygen F) g g University Hospital , Rotterdam , The Netherlands.; (Jaksch W) h h Wilhelminenspital der Stadt Wien , Austria.; (Kalso E) i i Pain Clinic, Department of Anaesthesiology, Intensive Care and Pain Medicine, University of Helsinki, and Helsinki University Hospital , Finland.; (Kocot-Kępska M) j j Department of Pain Research and Treatment , Collegium Medicum Jagiellonian University , Kraków , Poland.; (Mangas AC) k k UNIDOR, Centro Hospitalar de Leiria , Leiria , Portugal.; (Ferri CM) l l Hospital General Universitario de Alicante , Spain.; (Mavrocordatos P) m m Clinique Cecil , Lausanne , Switzerland.; (Morlion B) n n University Hospitals Leuven , Belgium.; (Müller-Schwefe G) o o Schmerz- und Palliativzentrum , Göppingen , Germany.; (Nicolaou A) p p St. Georges Hospital , London , UK.; (Hernández CP) q q Hospital Universitario de la Princesa , Madrid , Spain.; (Sichère P) r r Hôpitaux de Saint-Denis , Paris , France.
(Kress HG) a a Department of Special Anaesthesia and Pain Therapy , Medizinische Universität/AKH Wien , Vienna , Austria.; (Aldington D) b b Hampshire Hospitals NHS Trust , Winchester , Hants , UK.; (Alon E) c c Universitätsspital Zurich , Zurich , Switzerland.; (Coaccioli S) d d Santa Maria General Hospital , Terni , Italy.; (Collett B) e e University Hospitals of Leicester NHS Trust , Leicester , UK.; (Coluzzi F) f f Department of Medical and Surgical Sciences and Biotechnologies , Sapienza University of Rome , Italy.; (Huygen F) g g University Hospital , Rotterdam , The Netherlands.; (Jaksch W) h h Wilhelminenspital der Stadt Wien , Austria.; (Kalso E) i i Pain Clinic, Department of Anaesthesiology, Intensive Care and Pain Medicine, University of Helsinki, and Helsinki University Hospital , Finland.; (Kocot-Kępska M) j j Department of Pain Research and Treatment , Collegium Medicum Jagiellonian University , Kraków , Poland.; (Mangas AC) k k UNIDOR, Centro Hospitalar de Leiria , Leiria , Portugal.; (Ferri CM) l l Hospital General Universitario de Alicante , Spain.; (Mavrocordatos P) m m Clinique Cecil , Lausanne , Switzerland.; (Morlion B) n n

University Hospitals Leuven , Belgium.; (Müller-Schwefe G) o o Schmerz- und Palliativzentrum , Göppingen , Germany.; (Nicolaou A) p p St. Georges Hospital , London , UK.; (Hernández CP) q q Hospital Universitario de la Princesa , Madrid , Spain.; (Sichère P) r r Hôpitaux de Saint-Denis , Paris , France.

(Kress HG) a a Department of Special Anaesthesia and Pain Therapy , Medizinische Universität/AKH Wien , Vienna , Austria.; (Aldington D) b b Hampshire Hospitals NHS Trust , Winchester , Hants , UK.; (Alon E) c c Universitätsspital Zurich , Zurich , Switzerland.; (Coaccioli S) d d Santa Maria General Hospital , Terni , Italy.; (Collett B) e e University Hospitals of Leicester NHS Trust , Leicester , UK.; (Coluzzi F) f f Department of Medical and Surgical Sciences and Biotechnologies , Sapienza University of Rome , Italy.; (Huygen F) g g University Hospital , Rotterdam , The Netherlands.; (Jaksch W) h h Wilhelminenspital der Stadt Wien , Austria.; (Kalso E) i i Pain Clinic, Department of Anaesthesiology, Intensive Care and Pain Medicine, University of Helsinki, and Helsinki University Hospital , Finland.; (Kocot-Kępska M) j j Department of Pain Research and Treatment , Collegium Medicum Jagiellonian University , Kraków , Poland.; (Mangas AC) k k UNIDOR, Centro Hospitalar de Leiria , Leiria , Portugal.; (Ferri CM) l l Hospital General Universitario de Alicante , Spain.; (Mavrocordatos P) m m Clinique Cecil , Lausanne , Switzerland.; (Morlion B) n n University Hospitals Leuven , Belgium.; (Müller-Schwefe G) o o Schmerz- und Palliativzentrum , Göppingen , Germany.; (Nicolaou A) p p St. Georges Hospital , London , UK.; (Hernández CP) q q Hospital Universitario de la Princesa , Madrid , Spain.; (Sichère P) r r Hôpitaux de Saint-Denis , Paris , France.

(Kress HG) a a Department of Special Anaesthesia and Pain Therapy , Medizinische Universität/AKH Wien , Vienna , Austria.; (Aldington D) b b Hampshire Hospitals NHS Trust , Winchester , Hants , UK.; (Alon E) c c Universitätsspital Zurich , Zurich , Switzerland.; (Coaccioli S) d d Santa Maria General Hospital , Terni , Italy.; (Collett B) e e University Hospitals of Leicester NHS Trust , Leicester , UK.; (Coluzzi F) f f Department of Medical and Surgical Sciences and Biotechnologies , Sapienza University of Rome , Italy.; (Huygen F) g g University Hospital , Rotterdam , The Netherlands.; (Jaksch W) h h Wilhelminenspital der Stadt Wien , Austria.; (Kalso E) i i Pain Clinic, Department of Anaesthesiology, Intensive Care and Pain Medicine, University of Helsinki, and Helsinki University Hospital , Finland.; (Kocot-Kępska M) j j Department of Pain Research and Treatment , Collegium Medicum Jagiellonian University , Kraków , Poland.; (Mangas AC) k k UNIDOR, Centro Hospitalar de Leiria , Leiria , Portugal.; (Ferri CM) l l Hospital General Universitario de Alicante , Spain.; (Mavrocordatos P) m m Clinique Cecil , Lausanne , Switzerland.; (Morlion B) n n University Hospitals Leuven , Belgium.; (Müller-Schwefe G) o o Schmerz- und Palliativzentrum , Göppingen , Germany.; (Nicolaou A) p p St. Georges Hospital , London , UK.; (Hernández CP) q q Hospital Universitario de la Princesa , Madrid , Spain.; (Sichère P) r r Hôpitaux de Saint-Denis , Paris , France.

(Kress HG) a a Department of Special Anaesthesia and Pain Therapy , Medizinische Universität/AKH Wien , Vienna , Austria.; (Aldington D) b b Hampshire Hospitals NHS Trust , Winchester , Hants , UK.; (Alon E) c c Universitätsspital Zurich , Zurich , Switzerland.; (Coaccioli S) d d Santa Maria General Hospital , Terni , Italy.; (Collett B) e e University Hospitals of Leicester NHS Trust , Leicester , UK.; (Coluzzi F) f f Department of Medical and Surgical Sciences and Biotechnologies , Sapienza University of Rome , Italy.; (Huygen F) g g University Hospital , Rotterdam , The Netherlands.; (Jaksch W) h h Wilhelminenspital der Stadt Wien , Austria.; (Kalso E) i i Pain Clinic, Department of Anaesthesiology, Intensive Care and Pain Medicine, University of Helsinki, and Helsinki University Hospital , Finland.; (Kocot-Kępska M) j

j Department of Pain Research and Treatment , Collegium Medicum Jagiellonian University , Kraków , Poland.; (Mangas AC) k k UNIDOR, Centro Hospitalar de Leiria , Leiria , Portugal.; (Ferri CM) l l Hospital General Universitario de Alicante , Spain.; (Mavrocordatos P) m m Clinique Cecil , Lausanne , Switzerland.; (Morlion B) n n University Hospitals Leuven , Belgium.; (Müller-Schwefe G) o o Schmerz- und Palliativzentrum , Göppingen , Germany.; (Nicolaou A) p p St. Georges Hospital , London , UK.; (Hernández CP) q q Hospital Universitario de la Princesa , Madrid , Spain.; (Sichère P) r r Hôpitaux de Saint-Denis , Paris , France.

(Kress HG) a a Department of Special Anaesthesia and Pain Therapy , Medizinische Universität/AKH Wien , Vienna , Austria.; (Aldington D) b b Hampshire Hospitals NHS Trust , Winchester , Hants , UK.; (Alon E) c c Universitätsspital Zurich , Zurich , Switzerland.; (Coaccioli S) d d Santa Maria General Hospital , Terni , Italy.; (Collett B) e e University Hospitals of Leicester NHS Trust , Leicester , UK.; (Coluzzi F) f f Department of Medical and Surgical Sciences and Biotechnologies , Sapienza University of Rome , Italy.; (Huygen F) g g University Hospital , Rotterdam , The Netherlands.; (Jaksch W) h h Wilhelminenspital der Stadt Wien , Austria.; (Kalso E) i i Pain Clinic, Department of Anaesthesiology, Intensive Care and Pain Medicine, University of Helsinki, and Helsinki University Hospital , Finland.; (Kocot-Kępska M) j j Department of Pain Research and Treatment , Collegium Medicum Jagiellonian University , Kraków , Poland.; (Mangas AC) k k UNIDOR, Centro Hospitalar de Leiria , Leiria , Portugal.; (Ferri CM) l l Hospital General Universitario de Alicante , Spain.; (Mavrocordatos P) m m Clinique Cecil , Lausanne , Switzerland.; (Morlion B) n n University Hospitals Leuven , Belgium.; (Müller-Schwefe G) o o Schmerz- und Palliativzentrum , Göppingen , Germany.; (Nicolaou A) p p St. Georges Hospital , London , UK.; (Hernández CP) q q Hospital Universitario de la Princesa , Madrid , Spain.; (Sichère P) r r Hôpitaux de Saint-Denis , Paris , France.

(Kress HG) a a Department of Special Anaesthesia and Pain Therapy , Medizinische Universität/AKH Wien , Vienna , Austria.; (Aldington D) b b Hampshire Hospitals NHS Trust , Winchester , Hants , UK.; (Alon E) c c Universitätsspital Zurich , Zurich , Switzerland.; (Coaccioli S) d d Santa Maria General Hospital , Terni , Italy.; (Collett B) e e University Hospitals of Leicester NHS Trust , Leicester , UK.; (Coluzzi F) f f Department of Medical and Surgical Sciences and Biotechnologies , Sapienza University of Rome , Italy.; (Huygen F) g g University Hospital , Rotterdam , The Netherlands.; (Jaksch W) h h Wilhelminenspital der Stadt Wien , Austria.; (Kalso E) i i Pain Clinic, Department of Anaesthesiology, Intensive Care and Pain Medicine, University of Helsinki, and Helsinki University Hospital , Finland.; (Kocot-Kępska M) j j Department of Pain Research and Treatment , Collegium Medicum Jagiellonian University , Kraków , Poland.; (Mangas AC) k k UNIDOR, Centro Hospitalar de Leiria , Leiria , Portugal.; (Ferri CM) l l Hospital General Universitario de Alicante , Spain.; (Mavrocordatos P) m m Clinique Cecil , Lausanne , Switzerland.; (Morlion B) n n University Hospitals Leuven , Belgium.; (Müller-Schwefe G) o o Schmerz- und Palliativzentrum , Göppingen , Germany.; (Nicolaou A) p p St. Georges Hospital , London , UK.; (Hernández CP) q q Hospital Universitario de la Princesa , Madrid , Spain.; (Sichère P) r r Hôpitaux de Saint-Denis , Paris , France.

(Kress HG) a a Department of Special Anaesthesia and Pain Therapy , Medizinische Universität/AKH Wien , Vienna , Austria.; (Aldington D) b b Hampshire Hospitals NHS Trust , Winchester , Hants , UK.; (Alon E) c c Universitätsspital Zurich , Zurich , Switzerland.; (Coaccioli S) d d Santa Maria General Hospital , Terni , Italy.; (Collett B) e e University Hospitals of Leicester NHS Trust , Leicester , UK.; (Coluzzi F) f f Department of Medical and Surgical Sciences and Biotechnologies , Sapienza

University of Rome , Italy.; (Huygen F) g g University Hospital , Rotterdam , The Netherlands.; (Jaksch W) h h Wilhelminenspital der Stadt Wien , Austria.; (Kalso E) i i Pain Clinic, Department of Anaesthesiology, Intensive Care and Pain Medicine, University of Helsinki, and Helsinki University Hospital , Finland.; (Kocot-Kępska M) j j Department of Pain Research and Treatment , Collegium Medicum Jagiellonian University , Kraków , Poland.; (Mangas AC) k k UNIDOR, Centro Hospitalar de Leiria , Leiria , Portugal.; (Ferri CM) l l Hospital General Universitario de Alicante , Spain.; (Mavrocordatos P) m m Clinique Cecil , Lausanne , Switzerland.; (Morlion B) n n University Hospitals Leuven , Belgium.; (Müller-Schwefe G) o o Schmerz- und Palliativzentrum , Göppingen , Germany.; (Nicolaou A) p p St. Georges Hospital , London , UK.; (Hernández CP) q q Hospital Universitario de la Princesa , Madrid , Spain.; (Sichère P) r r Hôpitaux de Saint-Denis , Paris , France.
(Kress HG) a a Department of Special Anaesthesia and Pain Therapy , Medizinische Universität/AKH Wien , Vienna , Austria.; (Aldington D) b b Hampshire Hospitals NHS Trust , Winchester , Hants , UK.; (Alon E) c c Universitätsspital Zurich , Zurich , Switzerland.; (Coaccioli S) d d Santa Maria General Hospital , Terni , Italy.; (Collett B) e e University Hospitals of Leicester NHS Trust , Leicester , UK.; (Coluzzi F) f f Department of Medical and Surgical Sciences and Biotechnologies , Sapienza University of Rome , Italy.; (Huygen F) g g University Hospital , Rotterdam , The Netherlands.; (Jaksch W) h h Wilhelminenspital der Stadt Wien , Austria.; (Kalso E) i i Pain Clinic, Department of Anaesthesiology, Intensive Care and Pain Medicine, University of Helsinki, and Helsinki University Hospital , Finland.; (Kocot-Kępska M) j j Department of Pain Research and Treatment , Collegium Medicum Jagiellonian University , Kraków , Poland.; (Mangas AC) k k UNIDOR, Centro Hospitalar de Leiria , Leiria , Portugal.; (Ferri CM) l l Hospital General Universitario de Alicante , Spain.; (Mavrocordatos P) m m Clinique Cecil , Lausanne , Switzerland.; (Morlion B) n n University Hospitals Leuven , Belgium.; (Müller-Schwefe G) o o Schmerz- und Palliativzentrum , Göppingen , Germany.; (Nicolaou A) p p St. Georges Hospital , London , UK.; (Hernández CP) q q Hospital Universitario de la Princesa , Madrid , Spain.; (Sichère P) r r Hôpitaux de Saint-Denis , Paris , France.
(Kress HG) a a Department of Special Anaesthesia and Pain Therapy , Medizinische Universität/AKH Wien , Vienna , Austria.; (Aldington D) b b Hampshire Hospitals NHS Trust , Winchester , Hants , UK.; (Alon E) c c Universitätsspital Zurich , Zurich , Switzerland.; (Coaccioli S) d d Santa Maria General Hospital , Terni , Italy.; (Collett B) e e University Hospitals of Leicester NHS Trust , Leicester , UK.; (Coluzzi F) f f Department of Medical and Surgical Sciences and Biotechnologies , Sapienza University of Rome , Italy.; (Huygen F) g g University Hospital , Rotterdam , The Netherlands.; (Jaksch W) h h Wilhelminenspital der Stadt Wien , Austria.; (Kalso E) i i Pain Clinic, Department of Anaesthesiology, Intensive Care and Pain Medicine, University of Helsinki, and Helsinki University Hospital , Finland.; (Kocot-Kępska M) j j Department of Pain Research and Treatment , Collegium Medicum Jagiellonian University , Kraków , Poland.; (Mangas AC) k k UNIDOR, Centro Hospitalar de Leiria , Leiria , Portugal.; (Ferri CM) l l Hospital General Universitario de Alicante , Spain.; (Mavrocordatos P) m m Clinique Cecil , Lausanne , Switzerland.; (Morlion B) n n University Hospitals Leuven , Belgium.; (Müller-Schwefe G) o o Schmerz- und Palliativzentrum , Göppingen , Germany.; (Nicolaou A) p p St. Georges Hospital , London , UK.; (Hernández CP) q q Hospital Universitario de la Princesa , Madrid , Spain.; (Sichère P) r r Hôpitaux de Saint-Denis , Paris , France.
(Kress HG) a a Department of Special Anaesthesia and Pain Therapy , Medizinische Universität/AKH Wien , Vienna , Austria.; (Aldington D) b b Hampshire Hospitals

NHS Trust , Winchester , Hants , UK.; (Alon E) c c Universitätsspital Zurich , Zurich , Switzerland.; (Coaccioli S) d d Santa Maria General Hospital , Terni , Italy.; (Collett B) e e University Hospitals of Leicester NHS Trust , Leicester , UK.; (Coluzzi F) f f Department of Medical and Surgical Sciences and Biotechnologies , Sapienza University of Rome , Italy.; (Huygen F) g g University Hospital , Rotterdam , The Netherlands.; (Jaksch W) h h Wilhelminenspital der Stadt Wien , Austria.; (Kalso E) i i Pain Clinic, Department of Anaesthesiology, Intensive Care and Pain Medicine, University of Helsinki, and Helsinki University Hospital , Finland.; (Kocot-Kępska M) j j Department of Pain Research and Treatment , Collegium Medicum Jagiellonian University , Kraków , Poland.; (Mangas AC) k k UNIDOR, Centro Hospitalar de Leiria , Leiria , Portugal.; (Ferri CM) l l Hospital General Universitario de Alicante , Spain.; (Mavrocordatos P) m m Clinique Cecil , Lausanne , Switzerland.; (Morlion B) n n University Hospitals Leuven , Belgium.; (Müller-Schwefe G) o o Schmerz- und Palliativzentrum , Göppingen , Germany.; (Nicolaou A) p p St. Georges Hospital , London , UK.; (Hernández CP) q q Hospital Universitario de la Princesa , Madrid , Spain.; (Sichère P) r r Hôpitaux de Saint-Denis , Paris , France.
(Kress HG) a a Department of Special Anaesthesia and Pain Therapy , Medizinische Universität/AKH Wien , Vienna , Austria.; (Aldington D) b b Hampshire Hospitals NHS Trust , Winchester , Hants , UK.; (Alon E) c c Universitätsspital Zurich , Zurich , Switzerland.; (Coaccioli S) d d Santa Maria General Hospital , Terni , Italy.; (Collett B) e e University Hospitals of Leicester NHS Trust , Leicester , UK.; (Coluzzi F) f f Department of Medical and Surgical Sciences and Biotechnologies , Sapienza University of Rome , Italy.; (Huygen F) g g University Hospital , Rotterdam , The Netherlands.; (Jaksch W) h h Wilhelminenspital der Stadt Wien , Austria.; (Kalso E) i i Pain Clinic, Department of Anaesthesiology, Intensive Care and Pain Medicine, University of Helsinki, and Helsinki University Hospital , Finland.; (Kocot-Kępska M) j j Department of Pain Research and Treatment , Collegium Medicum Jagiellonian University , Kraków , Poland.; (Mangas AC) k k UNIDOR, Centro Hospitalar de Leiria , Leiria , Portugal.; (Ferri CM) l l Hospital General Universitario de Alicante , Spain.; (Mavrocordatos P) m m Clinique Cecil , Lausanne , Switzerland.; (Morlion B) n n University Hospitals Leuven , Belgium.; (Müller-Schwefe G) o o Schmerz- und Palliativzentrum , Göppingen , Germany.; (Nicolaou A) p p St. Georges Hospital , London , UK.; (Hernández CP) q q Hospital Universitario de la Princesa , Madrid , Spain.; (Sichère P) r r Hôpitaux de Saint-Denis , Paris , France.
(Kress HG) a a Department of Special Anaesthesia and Pain Therapy , Medizinische Universität/AKH Wien , Vienna , Austria.; (Aldington D) b b Hampshire Hospitals NHS Trust , Winchester , Hants , UK.; (Alon E) c c Universitätsspital Zurich , Zurich , Switzerland.; (Coaccioli S) d d Santa Maria General Hospital , Terni , Italy.; (Collett B) e e University Hospitals of Leicester NHS Trust , Leicester , UK.; (Coluzzi F) f f Department of Medical and Surgical Sciences and Biotechnologies , Sapienza University of Rome , Italy.; (Huygen F) g g University Hospital , Rotterdam , The Netherlands.; (Jaksch W) h h Wilhelminenspital der Stadt Wien , Austria.; (Kalso E) i i Pain Clinic, Department of Anaesthesiology, Intensive Care and Pain Medicine, University of Helsinki, and Helsinki University Hospital , Finland.; (Kocot-Kępska M) j j Department of Pain Research and Treatment , Collegium Medicum Jagiellonian University , Kraków , Poland.; (Mangas AC) k k UNIDOR, Centro Hospitalar de Leiria , Leiria , Portugal.; (Ferri CM) l l Hospital General Universitario de Alicante , Spain.; (Mavrocordatos P) m m Clinique Cecil , Lausanne , Switzerland.; (Morlion B) n n University Hospitals Leuven , Belgium.; (Müller-Schwefe G) o o Schmerz- und Palliativzentrum , Göppingen , Germany.; (Nicolaou A) p p St. Georges Hospital ,

London , UK.; (Hernández CP) q q Hospital Universitario de la Princesa , Madrid , Spain.; (Sichère P) r r Hôpitaux de Saint-Denis , Paris , France.
(Kress HG) a a Department of Special Anaesthesia and Pain Therapy , Medizinische Universität/AKH Wien , Vienna , Austria.; (Aldington D) b b Hampshire Hospitals NHS Trust , Winchester , Hants , UK.; (Alon E) c c Universitätsspital Zurich , Zurich , Switzerland.; (Coaccioli S) d d Santa Maria General Hospital , Terni , Italy.; (Collett B) e e University Hospitals of Leicester NHS Trust , Leicester , UK.; (Coluzzi F) f f Department of Medical and Surgical Sciences and Biotechnologies , Sapienza University of Rome , Italy.; (Huygen F) g g University Hospital , Rotterdam , The Netherlands.; (Jaksch W) h h Wilhelminenspital der Stadt Wien , Austria.; (Kalso E) i i Pain Clinic, Department of Anaesthesiology, Intensive Care and Pain Medicine, University of Helsinki, and Helsinki University Hospital , Finland.; (Kocot-Kępska M) j j Department of Pain Research and Treatment , Collegium Medicum Jagiellonian University , Kraków , Poland.; (Mangas AC) k k UNIDOR, Centro Hospitalar de Leiria , Leiria , Portugal.; (Ferri CM) l l Hospital General Universitario de Alicante , Spain.; (Mavrocordatos P) m m Clinique Cecil , Lausanne , Switzerland.; (Morlion B) n n University Hospitals Leuven , Belgium.; (Müller-Schwefe G) o o Schmerz- und Palliativzentrum , Göppingen , Germany.; (Nicolaou A) p p St. Georges Hospital , London , UK.; (Hernández CP) q q Hospital Universitario de la Princesa , Madrid , Spain.; (Sichère P) r r Hôpitaux de Saint-Denis , Paris , France.
(Kress HG) a a Department of Special Anaesthesia and Pain Therapy , Medizinische Universität/AKH Wien , Vienna , Austria.; (Aldington D) b b Hampshire Hospitals NHS Trust , Winchester , Hants , UK.; (Alon E) c c Universitätsspital Zurich , Zurich , Switzerland.; (Coaccioli S) d d Santa Maria General Hospital , Terni , Italy.; (Collett B) e e University Hospitals of Leicester NHS Trust , Leicester , UK.; (Coluzzi F) f f Department of Medical and Surgical Sciences and Biotechnologies , Sapienza University of Rome , Italy.; (Huygen F) g g University Hospital , Rotterdam , The Netherlands.; (Jaksch W) h h Wilhelminenspital der Stadt Wien , Austria.; (Kalso E) i i Pain Clinic, Department of Anaesthesiology, Intensive Care and Pain Medicine, University of Helsinki, and Helsinki University Hospital , Finland.; (Kocot-Kępska M) j j Department of Pain Research and Treatment , Collegium Medicum Jagiellonian University , Kraków , Poland.; (Mangas AC) k k UNIDOR, Centro Hospitalar de Leiria , Leiria , Portugal.; (Ferri CM) l l Hospital General Universitario de Alicante , Spain.; (Mavrocordatos P) m m Clinique Cecil , Lausanne , Switzerland.; (Morlion B) n n University Hospitals Leuven , Belgium.; (Müller-Schwefe G) o o Schmerz- und Palliativzentrum , Göppingen , Germany.; (Nicolaou A) p p St. Georges Hospital , London , UK.; (Hernández CP) q q Hospital Universitario de la Princesa , Madrid , Spain.; (Sichère P) r r Hôpitaux de Saint-Denis , Paris , France.
(Kress HG) a a Department of Special Anaesthesia and Pain Therapy , Medizinische Universität/AKH Wien , Vienna , Austria.; (Aldington D) b b Hampshire Hospitals NHS Trust , Winchester , Hants , UK.; (Alon E) c c Universitätsspital Zurich , Zurich , Switzerland.; (Coaccioli S) d d Santa Maria General Hospital , Terni , Italy.; (Collett B) e e University Hospitals of Leicester NHS Trust , Leicester , UK.; (Coluzzi F) f f Department of Medical and Surgical Sciences and Biotechnologies , Sapienza University of Rome , Italy.; (Huygen F) g g University Hospital , Rotterdam , The Netherlands.; (Jaksch W) h h Wilhelminenspital der Stadt Wien , Austria.; (Kalso E) i i Pain Clinic, Department of Anaesthesiology, Intensive Care and Pain Medicine, University of Helsinki, and Helsinki University Hospital , Finland.; (Kocot-Kępska M) j j Department of Pain Research and Treatment , Collegium Medicum Jagiellonian University , Kraków , Poland.; (Mangas AC) k k UNIDOR, Centro Hospitalar de Leiria

, Leiria , Portugal.; (Ferri CM) l l Hospital General Universitario de Alicante , Spain.; (Mavrocordatos P) m m Clinique Cecil , Lausanne , Switzerland.; (Morlion B) n n University Hospitals Leuven , Belgium.; (Müller-Schwefe G) o o Schmerz- und Palliativzentrum , Göppingen , Germany.; (Nicolaou A) p p St. Georges Hospital , London , UK.; (Hernández CP) q q Hospital Universitario de la Princesa , Madrid , Spain.; (Sichère P) r r Hôpitaux de Saint-Denis , Paris , France.
Database: PubMed

113. Priority interventions to improve the management of chronic non-cancer pain in primary care: a participatory research of the ACCORD program.

Author(s): Lalonde L; Choinière M; Martin E; Lévesque L; Hudon E; Bélanger D; Perreault S; Lacasse A; Laliberté MC

Source: Journal of pain research; 2015; vol. 8 ; p. 203-215

Publication Date: 2015

Publication Type(s): Journal Article

DOI: http://dx.doi.org/10.2147/JPR.S78177

ISSN: 1178-7090

Place of Publication: New Zealand

PubMedID: 25995648

Accession Number: 25995648

Available at Journal of pain research - from Europe PubMed Central - Open Access

Available at Journal of pain research - from Unpaywall

Keywords: Subject Terms: chronic pain; community-based participatory research; health service accessibility; patient-centered care; primary health care

Abstract:PURPOSE: There is evidence that the management of chronic non-cancer pain (CNCP) in primary care is far from being optimal. A 1-day workshop was held to explore the perceptions of key actors regarding the challenges and priority interventions to improve CNCP management in primary care.METHODS: Using the Chronic Care Model as a conceptual framework, physicians (n=6), pharmacists (n=6), nurses (n=6), physiotherapists (n=6), psychologists (n=6), pain specialists (n=6), patients (n=3), family members (n=3), decision makers and managers (n=4), and pain researchers (n=7) took part in seven focus groups and five nominal groups.RESULTS: Challenges identified in focus group discussions were related to five dimensions: knowledge gap, "work in silos", lack of awareness that CNCP represents an important clinical problem, difficulties in access to health professionals and services, and patient empowerment needs. Based on the nominal group discussions, the following priority interventions were identified: interdisciplinary continuing education, interdisciplinary treatment approach, regional expert leadership, creation and definition of care paths, and patient education programs.CONCLUSION: Barriers to optimal management of CNCP in primary care are numerous. Improving its management cannot be envisioned without considering multifaceted interventions targeting several dimensions of the Chronic Care Model and focusing on both clinicians and patients.

Institutions:

(Lalonde L) Faculty of Pharmacy, Université de Montréal, Montreal, QC, Canada ; Équipe de recherche en soins de première ligne, Centre de santé et de services sociaux de Laval, Laval, QC, Canada ; Centre de recherche, Centre hospitalier de l'Université de Montréal (CRCHUM), Montreal, QC, Canada ; Sanofi Aventis Endowment Chair in Ambulatory Pharmaceutical Care, Faculty of Pharmacy Université de Montréal and Centre de santé et de services sociaux de Laval, QC, Canada.; (Choinière M) Centre de recherche, Centre hospitalier de l'Université de Montréal (CRCHUM), Montreal, QC, Canada ; Department of Anesthesiology Faculty of Medicine, Université de Montréal, Montreal, QC, Canada.; (Martin E) Centre de recherche, Centre hospitalier de l'Université de Montréal (CRCHUM), Montreal, QC, Canada.; (Lévesque L) Centre de recherche, Centre hospitalier de l'Université de Montréal (CRCHUM), Montreal, QC, Canada.; (Hudon E) Équipe de recherche en soins de première ligne, Centre de santé et de services sociaux de Laval, Laval, QC, Canada ; Centre de recherche, Centre hospitalier de l'Université de Montréal (CRCHUM), Montreal, QC, Canada ; Department of Family Medicine and Emergency, Faculty of Medicine, Université de Montréal, Montreal, QC, Canada.; (Bélanger D) Équipe de recherche en soins de première ligne, Centre de santé et de services sociaux de Laval, Laval, QC, Canada.; (Perreault S) Faculty of Pharmacy, Université de Montréal, Montreal, QC, Canada ; Sanofi Aventis Endowment Chair in Drug Utilization, Faculty of Pharmacy, Université de Montréal, Montreal, QC, Canada.; (Lacasse A) Département des sciences de la santé, Université du Québec en Abitibi-Témiscamingue, Rouyn-Noranda, QC, Canada.; (Laliberté MC) Faculty of Pharmacy, Université de Montréal, Montreal, QC, Canada ; AbbVie Corporation, St-Laurent, QC, Canada.
(Lalonde L) Faculty of Pharmacy, Université de Montréal, Montreal, QC, Canada ; Équipe de recherche en soins de première ligne, Centre de santé et de services sociaux de Laval, Laval, QC, Canada ; Centre de recherche, Centre hospitalier de l'Université de Montréal (CRCHUM), Montreal, QC, Canada ; Sanofi Aventis Endowment Chair in Ambulatory Pharmaceutical Care, Faculty of Pharmacy Université de Montréal and Centre de santé et de services sociaux de Laval, QC, Canada.; (Choinière M) Centre de recherche, Centre hospitalier de l'Université de Montréal (CRCHUM), Montreal, QC, Canada ; Department of Anesthesiology Faculty of Medicine, Université de Montréal, Montreal, QC, Canada.; (Martin E) Centre de recherche, Centre hospitalier de l'Université de Montréal (CRCHUM), Montreal, QC, Canada.; (Lévesque L) Centre de recherche, Centre hospitalier de l'Université de Montréal (CRCHUM), Montreal, QC, Canada.; (Hudon E) Équipe de recherche en soins de première ligne, Centre de santé et de services sociaux de Laval, Laval, QC, Canada ; Centre de recherche, Centre hospitalier de l'Université de Montréal (CRCHUM), Montreal, QC, Canada ; Department of Family Medicine and Emergency, Faculty of Medicine, Université de Montréal, Montreal, QC, Canada.; (Bélanger D) Équipe de recherche en soins de première ligne, Centre de santé et de services sociaux de Laval, Laval, QC, Canada.; (Perreault S) Faculty of Pharmacy, Université de Montréal, Montreal, QC, Canada ; Sanofi Aventis Endowment Chair in Drug Utilization, Faculty of Pharmacy, Université de Montréal, Montreal, QC, Canada.; (Lacasse A) Département des sciences de la santé, Université du Québec en Abitibi-Témiscamingue, Rouyn-Noranda, QC, Canada.; (Laliberté MC) Faculty of Pharmacy, Université de Montréal, Montreal, QC, Canada ; AbbVie Corporation, St-Laurent, QC, Canada.
(Lalonde L) Faculty of Pharmacy, Université de Montréal, Montreal, QC, Canada ; Équipe de recherche en soins de première ligne, Centre de santé et de services

sociaux de Laval, Laval, QC, Canada ; Centre de recherche, Centre hospitalier de l'Université de Montréal (CRCHUM), Montreal, QC, Canada ; Sanofi Aventis Endowment Chair in Ambulatory Pharmaceutical Care, Faculty of Pharmacy Université de Montréal and Centre de santé et de services sociaux de Laval, QC, Canada.; (Choinière M) Centre de recherche, Centre hospitalier de l'Université de Montréal (CRCHUM), Montreal, QC, Canada ; Department of Anesthesiology Faculty of Medicine, Université de Montréal, Montreal, QC, Canada.; (Martin E) Centre de recherche, Centre hospitalier de l'Université de Montréal (CRCHUM), Montreal, QC, Canada.; (Lévesque L) Centre de recherche, Centre hospitalier de l'Université de Montréal (CRCHUM), Montreal, QC, Canada.; (Hudon E) Équipe de recherche en soins de première ligne, Centre de santé et de services sociaux de Laval, Laval, QC, Canada ; Centre de recherche, Centre hospitalier de l'Université de Montréal (CRCHUM), Montreal, QC, Canada ; Department of Family Medicine and Emergency, Faculty of Medicine, Université de Montréal, Montreal, QC, Canada.; (Bélanger D) Équipe de recherche en soins de première ligne, Centre de santé et de services sociaux de Laval, Laval, QC, Canada.; (Perreault S) Faculty of Pharmacy, Université de Montréal, Montreal, QC, Canada ; Sanofi Aventis Endowment Chair in Drug Utilization, Faculty of Pharmacy, Université de Montréal, Montreal, QC, Canada.; (Lacasse A) Département des sciences de la santé, Université du Québec en Abitibi-Témiscamingue, Rouyn-Noranda, QC, Canada.; (Laliberté MC) Faculty of Pharmacy, Université de Montréal, Montreal, QC, Canada ; AbbVie Corporation, St-Laurent, QC, Canada.
(Lalonde L) Faculty of Pharmacy, Université de Montréal, Montreal, QC, Canada ; Équipe de recherche en soins de première ligne, Centre de santé et de services sociaux de Laval, Laval, QC, Canada ; Centre de recherche, Centre hospitalier de l'Université de Montréal (CRCHUM), Montreal, QC, Canada ; Sanofi Aventis Endowment Chair in Ambulatory Pharmaceutical Care, Faculty of Pharmacy Université de Montréal and Centre de santé et de services sociaux de Laval, QC, Canada.; (Choinière M) Centre de recherche, Centre hospitalier de l'Université de Montréal (CRCHUM), Montreal, QC, Canada ; Department of Anesthesiology Faculty of Medicine, Université de Montréal, Montreal, QC, Canada.; (Martin E) Centre de recherche, Centre hospitalier de l'Université de Montréal (CRCHUM), Montreal, QC, Canada.; (Lévesque L) Centre de recherche, Centre hospitalier de l'Université de Montréal (CRCHUM), Montreal, QC, Canada.; (Hudon E) Équipe de recherche en soins de première ligne, Centre de santé et de services sociaux de Laval, Laval, QC, Canada ; Centre de recherche, Centre hospitalier de l'Université de Montréal (CRCHUM), Montreal, QC, Canada ; Department of Family Medicine and Emergency, Faculty of Medicine, Université de Montréal, Montreal, QC, Canada.; (Bélanger D) Équipe de recherche en soins de première ligne, Centre de santé et de services sociaux de Laval, Laval, QC, Canada.; (Perreault S) Faculty of Pharmacy, Université de Montréal, Montreal, QC, Canada ; Sanofi Aventis Endowment Chair in Drug Utilization, Faculty of Pharmacy, Université de Montréal, Montreal, QC, Canada.; (Lacasse A) Département des sciences de la santé, Université du Québec en Abitibi-Témiscamingue, Rouyn-Noranda, QC, Canada.; (Laliberté MC) Faculty of Pharmacy, Université de Montréal, Montreal, QC, Canada ; AbbVie Corporation, St-Laurent, QC, Canada.
(Lalonde L) Faculty of Pharmacy, Université de Montréal, Montreal, QC, Canada ; Équipe de recherche en soins de première ligne, Centre de santé et de services sociaux de Laval, Laval, QC, Canada ; Centre de recherche, Centre hospitalier de l'Université de Montréal (CRCHUM), Montreal, QC, Canada ; Sanofi Aventis

Endowment Chair in Ambulatory Pharmaceutical Care, Faculty of Pharmacy Université de Montréal and Centre de santé et de services sociaux de Laval, QC, Canada.; (Choinière M) Centre de recherche, Centre hospitalier de l'Université de Montréal (CRCHUM), Montreal, QC, Canada ; Department of Anesthesiology Faculty of Medicine, Université de Montréal, Montreal, QC, Canada.; (Martin E) Centre de recherche, Centre hospitalier de l'Université de Montréal (CRCHUM), Montreal, QC, Canada.; (Lévesque L) Centre de recherche, Centre hospitalier de l'Université de Montréal (CRCHUM), Montreal, QC, Canada.; (Hudon E) Équipe de recherche en soins de première ligne, Centre de santé et de services sociaux de Laval, Laval, QC, Canada ; Centre de recherche, Centre hospitalier de l'Université de Montréal (CRCHUM), Montreal, QC, Canada ; Department of Family Medicine and Emergency, Faculty of Medicine, Université de Montréal, Montreal, QC, Canada.; (Bélanger D) Équipe de recherche en soins de première ligne, Centre de santé et de services sociaux de Laval, Laval, QC, Canada.; (Perreault S) Faculty of Pharmacy, Université de Montréal, Montreal, QC, Canada ; Sanofi Aventis Endowment Chair in Drug Utilization, Faculty of Pharmacy, Université de Montréal, Montreal, QC, Canada.; (Lacasse A) Département des sciences de la santé, Université du Québec en Abitibi-Témiscamingue, Rouyn-Noranda, QC, Canada.; (Laliberté MC) Faculty of Pharmacy, Université de Montréal, Montreal, QC, Canada ; AbbVie Corporation, St-Laurent, QC, Canada.

(Lalonde L) Faculty of Pharmacy, Université de Montréal, Montreal, QC, Canada ; Équipe de recherche en soins de première ligne, Centre de santé et de services sociaux de Laval, Laval, QC, Canada ; Centre de recherche, Centre hospitalier de l'Université de Montréal (CRCHUM), Montreal, QC, Canada ; Sanofi Aventis Endowment Chair in Ambulatory Pharmaceutical Care, Faculty of Pharmacy Université de Montréal and Centre de santé et de services sociaux de Laval, QC, Canada.; (Choinière M) Centre de recherche, Centre hospitalier de l'Université de Montréal (CRCHUM), Montreal, QC, Canada ; Department of Anesthesiology Faculty of Medicine, Université de Montréal, Montreal, QC, Canada.; (Martin E) Centre de recherche, Centre hospitalier de l'Université de Montréal (CRCHUM), Montreal, QC, Canada.; (Lévesque L) Centre de recherche, Centre hospitalier de l'Université de Montréal (CRCHUM), Montreal, QC, Canada.; (Hudon E) Équipe de recherche en soins de première ligne, Centre de santé et de services sociaux de Laval, Laval, QC, Canada ; Centre de recherche, Centre hospitalier de l'Université de Montréal (CRCHUM), Montreal, QC, Canada ; Department of Family Medicine and Emergency, Faculty of Medicine, Université de Montréal, Montreal, QC, Canada.; (Bélanger D) Équipe de recherche en soins de première ligne, Centre de santé et de services sociaux de Laval, Laval, QC, Canada.; (Perreault S) Faculty of Pharmacy, Université de Montréal, Montreal, QC, Canada ; Sanofi Aventis Endowment Chair in Drug Utilization, Faculty of Pharmacy, Université de Montréal, Montreal, QC, Canada.; (Lacasse A) Département des sciences de la santé, Université du Québec en Abitibi-Témiscamingue, Rouyn-Noranda, QC, Canada.; (Laliberté MC) Faculty of Pharmacy, Université de Montréal, Montreal, QC, Canada ; AbbVie Corporation, St-Laurent, QC, Canada.

(Lalonde L) Faculty of Pharmacy, Université de Montréal, Montreal, QC, Canada ; Équipe de recherche en soins de première ligne, Centre de santé et de services sociaux de Laval, Laval, QC, Canada ; Centre de recherche, Centre hospitalier de l'Université de Montréal (CRCHUM), Montreal, QC, Canada ; Sanofi Aventis Endowment Chair in Ambulatory Pharmaceutical Care, Faculty of Pharmacy Université de Montréal and Centre de santé et de services sociaux de Laval, QC,

Canada.; (Choinière M) Centre de recherche, Centre hospitalier de l'Université de Montréal (CRCHUM), Montreal, QC, Canada ; Department of Anesthesiology Faculty of Medicine, Université de Montréal, Montreal, QC, Canada.; (Martin E) Centre de recherche, Centre hospitalier de l'Université de Montréal (CRCHUM), Montreal, QC, Canada.; (Lévesque L) Centre de recherche, Centre hospitalier de l'Université de Montréal (CRCHUM), Montreal, QC, Canada.; (Hudon E) Équipe de recherche en soins de première ligne, Centre de santé et de services sociaux de Laval, Laval, QC, Canada ; Centre de recherche, Centre hospitalier de l'Université de Montréal (CRCHUM), Montreal, QC, Canada ; Department of Family Medicine and Emergency, Faculty of Medicine, Université de Montréal, Montreal, QC, Canada.; (Bélanger D) Équipe de recherche en soins de première ligne, Centre de santé et de services sociaux de Laval, Laval, QC, Canada.; (Perreault S) Faculty of Pharmacy, Université de Montréal, Montreal, QC, Canada ; Sanofi Aventis Endowment Chair in Drug Utilization, Faculty of Pharmacy, Université de Montréal, Montreal, QC, Canada.; (Lacasse A) Département des sciences de la santé, Université du Québec en Abitibi-Témiscamingue, Rouyn-Noranda, QC, Canada.; (Laliberté MC) Faculty of Pharmacy, Université de Montréal, Montreal, QC, Canada ; AbbVie Corporation, St-Laurent, QC, Canada.
(Lalonde L) Faculty of Pharmacy, Université de Montréal, Montreal, QC, Canada ; Équipe de recherche en soins de première ligne, Centre de santé et de services sociaux de Laval, Laval, QC, Canada ; Centre de recherche, Centre hospitalier de l'Université de Montréal (CRCHUM), Montreal, QC, Canada ; Sanofi Aventis Endowment Chair in Ambulatory Pharmaceutical Care, Faculty of Pharmacy Université de Montréal and Centre de santé et de services sociaux de Laval, QC, Canada.; (Choinière M) Centre de recherche, Centre hospitalier de l'Université de Montréal (CRCHUM), Montreal, QC, Canada ; Department of Anesthesiology Faculty of Medicine, Université de Montréal, Montreal, QC, Canada.; (Martin E) Centre de recherche, Centre hospitalier de l'Université de Montréal (CRCHUM), Montreal, QC, Canada.; (Lévesque L) Centre de recherche, Centre hospitalier de l'Université de Montréal (CRCHUM), Montreal, QC, Canada.; (Hudon E) Équipe de recherche en soins de première ligne, Centre de santé et de services sociaux de Laval, Laval, QC, Canada ; Centre de recherche, Centre hospitalier de l'Université de Montréal (CRCHUM), Montreal, QC, Canada ; Department of Family Medicine and Emergency, Faculty of Medicine, Université de Montréal, Montreal, QC, Canada.; (Bélanger D) Équipe de recherche en soins de première ligne, Centre de santé et de services sociaux de Laval, Laval, QC, Canada.; (Perreault S) Faculty of Pharmacy, Université de Montréal, Montreal, QC, Canada ; Sanofi Aventis Endowment Chair in Drug Utilization, Faculty of Pharmacy, Université de Montréal, Montreal, QC, Canada.; (Lacasse A) Département des sciences de la santé, Université du Québec en Abitibi-Témiscamingue, Rouyn-Noranda, QC, Canada.; (Laliberté MC) Faculty of Pharmacy, Université de Montréal, Montreal, QC, Canada ; AbbVie Corporation, St-Laurent, QC, Canada.
(Lalonde L) Faculty of Pharmacy, Université de Montréal, Montreal, QC, Canada ; Équipe de recherche en soins de première ligne, Centre de santé et de services sociaux de Laval, Laval, QC, Canada ; Centre de recherche, Centre hospitalier de l'Université de Montréal (CRCHUM), Montreal, QC, Canada ; Sanofi Aventis Endowment Chair in Ambulatory Pharmaceutical Care, Faculty of Pharmacy Université de Montréal and Centre de santé et de services sociaux de Laval, QC, Canada.; (Choinière M) Centre de recherche, Centre hospitalier de l'Université de Montréal (CRCHUM), Montreal, QC, Canada ; Department of Anesthesiology Faculty

of Medicine, Université de Montréal, Montreal, QC, Canada.; (Martin E) Centre de recherche, Centre hospitalier de l'Université de Montréal (CRCHUM), Montreal, QC, Canada.; (Lévesque L) Centre de recherche, Centre hospitalier de l'Université de Montréal (CRCHUM), Montreal, QC, Canada.; (Hudon E) Équipe de recherche en soins de première ligne, Centre de santé et de services sociaux de Laval, Laval, QC, Canada ; Centre de recherche, Centre hospitalier de l'Université de Montréal (CRCHUM), Montreal, QC, Canada ; Department of Family Medicine and Emergency, Faculty of Medicine, Université de Montréal, Montreal, QC, Canada.; (Bélanger D) Équipe de recherche en soins de première ligne, Centre de santé et de services sociaux de Laval, Laval, QC, Canada.; (Perreault S) Faculty of Pharmacy, Université de Montréal, Montreal, QC, Canada ; Sanofi Aventis Endowment Chair in Drug Utilization, Faculty of Pharmacy, Université de Montréal, Montreal, QC, Canada.; (Lacasse A) Département des sciences de la santé, Université du Québec en Abitibi-Témiscamingue, Rouyn-Noranda, QC, Canada.; (Laliberté MC) Faculty of Pharmacy, Université de Montréal, Montreal, QC, Canada ; AbbVie Corporation, St-Laurent, QC, Canada.
Database: PubMed

114. Self-management support intervention to control cancer pain in the outpatient setting: a randomized controlled trial study protocol.

Author(s): Hochstenbach LM; Courtens AM; Zwakhalen SM; van Kleef M; de Witte LP

Source: BMC cancer; May 2015; vol. 15 ; p. 416

Publication Date: May 2015

Publication Type(s): Comparative Study; Journal Article; Multicenter Study; Randomized Controlled Trial; Research Support, Non-U.S. Gov't

DOI: http://dx.doi.org/10.1186/s12885-015-1428-1

ISSN: 1471-2407

Place of Publication: England

PubMedID: 25986294

Accession Number: 25986294

Available at BMC cancer - from BioMed Central

Available at BMC cancer - from Europe PubMed Central - Open Access

Available at BMC cancer - from DOAJ - Directory of Open Access Journals

Available at BMC cancer - from ProQuest (Health Research Premium) - NHS Version

Available at BMC cancer - from EBSCO (MEDLINE Complete)

Available at BMC cancer - from Unpaywall

Abstract:BACKGROUND: Pain is a prevalent and distressing symptom in patients with cancer, having an enormous impact on functioning and quality of life. Fragmentation of care, inadequate pain communication, and reluctance towards pain medication contribute to difficulties in optimizing outcomes. Integration of patient self-management and professional care by means of healthcare technology provides new opportunities in the outpatient setting.METHODS/DESIGN: This study protocol

outlines a two-armed multicenter randomized controlled trial that compares a technology based multicomponent self-management support intervention with care as usual and includes an effect, economic and process evaluation. Patients will be recruited consecutively via the outpatient oncology clinics and inpatient oncology wards of one academic hospital and one regional hospital in the south of the Netherlands. Irrespective of the stage of disease, patients are eligible when they are diagnosed with cancer and have uncontrolled moderate to severe cancer (treatment) related pain defined as NRS≥4 for more than two weeks. Randomization (1:1) will assign patients to either the intervention or control group; patients in the intervention group receive self-management support and patients in the control group receive care as usual. The intervention will be delivered by registered nurses specialized in pain and palliative care. Important components include monitoring of pain, adverse effects and medication as well as graphical feedback, education, and nurse support. Effect measurements for both groups will be carried out with questionnaires at baseline (T0), after 4 weeks (T1) and after 12 weeks (T2). Pain intensity and quality of life are the primary outcomes. Secondary outcomes include self-efficacy, knowledge, anxiety, depression and pain medication use. The final questionnaire contains also questions for the economic evaluation that includes both cost-effectiveness and cost-utility analysis. Data for the process evaluation will be gathered continuously over the study period and focus on recruitment, reach, dose delivered and dose received.DISCUSSION: The proposed study will provide insight into the effectiveness of the self-management support intervention delivered by nurses to outpatients with uncontrolled cancer pain. Study findings will be used to empower patients and health professionals to improve cancer pain control.TRIAL REGISTRATION: NCT02333968 December 29, 2014.

Institutions:

(Hochstenbach LM) School for Public Health and Primary Care (CAPHRI), Department of Health Services Research, Maastricht University, P.O. Box 616, 6200, Maastricht, MD, The Netherlands. l.hochstenbach@maastrichtuniversity.nl.; (Courtens AM) Department of Patient and Care, Maastricht University Medical Center, P.O. Box 5800, 6202, Maastricht, AZ, The Netherlands. a.courtens@mumc.nl.; (Zwakhalen SM) School for Public Health and Primary Care (CAPHRI), Department of Health Services Research, Maastricht University, P.O. Box 616, 6200, Maastricht, MD, The Netherlands. s.zwakhalen@maastrichtuniversity.nl.; (van Kleef M) Department of Anesthesiology, School for Mental Health and Neuroscience (MHeNS), Maastricht University Medical Center, P.O. Box 5800, 6202, Maastricht, AZ, The Netherlands. maarten.van.kleef@mumc.nl.; (de Witte LP) School for Public Health and Primary Care (CAPHRI), Department of Health Services Research, Maastricht University, P.O. Box 616, 6200, Maastricht, MD, The Netherlands. l.dewitte@maastrichtuniversity.nl.
(Hochstenbach LM) School for Public Health and Primary Care (CAPHRI), Department of Health Services Research, Maastricht University, P.O. Box 616, 6200, Maastricht, MD, The Netherlands. l.hochstenbach@maastrichtuniversity.nl.; (Courtens AM) Department of Patient and Care, Maastricht University Medical Center, P.O. Box 5800, 6202, Maastricht, AZ, The Netherlands. a.courtens@mumc.nl.; (Zwakhalen SM) School for Public Health and Primary Care (CAPHRI), Department of Health Services Research, Maastricht University, P.O. Box 616, 6200, Maastricht, MD, The Netherlands. s.zwakhalen@maastrichtuniversity.nl.; (van Kleef M) Department of Anesthesiology, School for Mental Health and Neuroscience (MHeNS), Maastricht University Medical Center, P.O. Box 5800, 6202,

Maastricht, AZ, The Netherlands. maarten.van.kleef@mumc.nl.; (de Witte LP) School for Public Health and Primary Care (CAPHRI), Department of Health Services Research, Maastricht University, P.O. Box 616, 6200, Maastricht, MD, The Netherlands. l.dewitte@maastrichtuniversity.nl.
(Hochstenbach LM) School for Public Health and Primary Care (CAPHRI), Department of Health Services Research, Maastricht University, P.O. Box 616, 6200, Maastricht, MD, The Netherlands. l.hochstenbach@maastrichtuniversity.nl.; (Courtens AM) Department of Patient and Care, Maastricht University Medical Center, P.O. Box 5800, 6202, Maastricht, AZ, The Netherlands. a.courtens@mumc.nl.; (Zwakhalen SM) School for Public Health and Primary Care (CAPHRI), Department of Health Services Research, Maastricht University, P.O. Box 616, 6200, Maastricht, MD, The Netherlands. s.zwakhalen@maastrichtuniversity.nl.; (van Kleef M) Department of Anesthesiology, School for Mental Health and Neuroscience (MHeNS), Maastricht University Medical Center, P.O. Box 5800, 6202, Maastricht, AZ, The Netherlands. maarten.van.kleef@mumc.nl.; (de Witte LP) School for Public Health and Primary Care (CAPHRI), Department of Health Services Research, Maastricht University, P.O. Box 616, 6200, Maastricht, MD, The Netherlands. l.dewitte@maastrichtuniversity.nl.
(Hochstenbach LM) School for Public Health and Primary Care (CAPHRI), Department of Health Services Research, Maastricht University, P.O. Box 616, 6200, Maastricht, MD, The Netherlands. l.hochstenbach@maastrichtuniversity.nl.; (Courtens AM) Department of Patient and Care, Maastricht University Medical Center, P.O. Box 5800, 6202, Maastricht, AZ, The Netherlands. a.courtens@mumc.nl.; (Zwakhalen SM) School for Public Health and Primary Care (CAPHRI), Department of Health Services Research, Maastricht University, P.O. Box 616, 6200, Maastricht, MD, The Netherlands. s.zwakhalen@maastrichtuniversity.nl.; (van Kleef M) Department of Anesthesiology, School for Mental Health and Neuroscience (MHeNS), Maastricht University Medical Center, P.O. Box 5800, 6202, Maastricht, AZ, The Netherlands. maarten.van.kleef@mumc.nl.; (de Witte LP) School for Public Health and Primary Care (CAPHRI), Department of Health Services Research, Maastricht University, P.O. Box 616, 6200, Maastricht, MD, The Netherlands. l.dewitte@maastrichtuniversity.nl.
Database: PubMed

115. Current practices in cancer pain management in Asia: a survey of patients and physicians across 10 countries.

Author(s): ACHEON Working Group; Kim YC; Ahn JS; Calimag MM; Chao TC; Ho KY; Tho LM; Xia ZJ; Ward L; Moon H; Bhagat A

Source: Cancer medicine; Aug 2015; vol. 4 (no. 8); p. 1196-1204

Publication Date: Aug 2015

Publication Type(s): Journal Article; Multicenter Study; Research Support, Non-U.S. Gov't

DOI: http://dx.doi.org/10.1002/cam4.471

ISSN: 2045-7634

Place of Publication: United States

PubMedID: 25914253

Accession Number: 25914253

Available at Cancer medicine - from Europe PubMed Central - Open Access

Available at Cancer medicine - from Wiley Online Library Free Content - NHS

Available at Cancer medicine - from DOAJ - Directory of Open Access Journals

Available at Cancer medicine - from ProQuest (Health Research Premium) - NHS Version

Keywords: Subject Terms: Cancer pain; pain management survey

Abstract: In order to implement more effective policies for cancer pain management, a better understanding of current practices is needed. Physicians managing cancer pain and patients experiencing cancer pain were randomly surveyed across 10 Asian countries to assess attitudes and perceptions toward cancer pain management. A total of 463 physicians (77.3% oncologists) with a median experience of 13 years were included. Medical school training on opioid use was considered inadequate by 30.5% of physicians and 55.9% indicated ≤ 10 h of continuing medical education (CME). Of the 1190 patients included, 1026 reported moderate-to-severe pain (median duration, 12 months). Discordance was observed between physician and patient outcomes on pain assessment with 88.3% of physicians reporting pain quantification, while 49.5% of patients claimed that no scale was used. Inadequate assessment of pain was recognized as a barrier to therapy optimization by 49.7% of physicians. Additional barriers identified were patients' reluctance owing to fear of addiction (67.2%) and adverse events (65.0%), patients' reluctance to report pain (52.5%), excessive regulations (48.0%) and reluctance to prescribe opioids (42.8%). Opioid use was confirmed only in 53.2% (286/538) of patients remembering their medication. Pain affected the activities of daily living for 81.3% of patients. These findings highlight the need for better training and CME opportunities for cancer pain management in Asia. Collaborative efforts between physicians, patients, policy makers, and related parties may assist in overcoming the barriers identified. Addressing the opioid stigma and enhancing awareness is vital to improving current standards of patient care.

Institutions:
(ACHEON Working Group) ACHEON Working Group; (Kim YC) Department of Anesthesiology and Pain Medicine, Seoul National University School of Medicine, Seoul, Korea.; (Ahn JS) Department of Medicine, Samsung Medical Center,

Sungkyunkwan University School of Medicine, Seoul, Korea.; (Calimag MM) Departments of Pharmacology, Clinical Epidemiology and Anesthesiology, University of Santo Tomas Faculty of Medicine and Surgery and the UST Hospital, Manila, Philippines.; (Chao TC) Institute of Clinical Medicine, School of Medicine, National Yang-Ming University, Taipei, Taiwan, China.; (Ho KY) Pain Management Service, Raffles Hospital, Singapore, Singapore.; (Tho LM) Department of Clinical Oncology, Beacon International Specialist Centre, Selangor, Malaysia.; (Xia ZJ) Sun Yat-Sen University Cancer Center, Guangzhou, China.; (Ward L) Mundipharma Research Ltd, Cambridge, United Kingdom.; (Moon H) Mundipharma Pte Ltd, Singapore, Singapore.; (Bhagat A) Mundipharma Pte Ltd, Singapore, Singapore.
(ACHEON Working Group) ACHEON Working Group; (Kim YC) Department of Anesthesiology and Pain Medicine, Seoul National University School of Medicine, Seoul, Korea.; (Ahn JS) Department of Medicine, Samsung Medical Center, Sungkyunkwan University School of Medicine, Seoul, Korea.; (Calimag MM) Departments of Pharmacology, Clinical Epidemiology and Anesthesiology, University of Santo Tomas Faculty of Medicine and Surgery and the UST Hospital, Manila, Philippines.; (Chao TC) Institute of Clinical Medicine, School of Medicine, National Yang-Ming University, Taipei, Taiwan, China.; (Ho KY) Pain Management Service, Raffles Hospital, Singapore, Singapore.; (Tho LM) Department of Clinical Oncology, Beacon International Specialist Centre, Selangor, Malaysia.; (Xia ZJ) Sun Yat-Sen University Cancer Center, Guangzhou, China.; (Ward L) Mundipharma Research Ltd, Cambridge, United Kingdom.; (Moon H) Mundipharma Pte Ltd, Singapore, Singapore.; (Bhagat A) Mundipharma Pte Ltd, Singapore, Singapore.
(ACHEON Working Group) ACHEON Working Group; (Kim YC) Department of Anesthesiology and Pain Medicine, Seoul National University School of Medicine, Seoul, Korea.; (Ahn JS) Department of Medicine, Samsung Medical Center, Sungkyunkwan University School of Medicine, Seoul, Korea.; (Calimag MM) Departments of Pharmacology, Clinical Epidemiology and Anesthesiology, University of Santo Tomas Faculty of Medicine and Surgery and the UST Hospital, Manila, Philippines.; (Chao TC) Institute of Clinical Medicine, School of Medicine, National Yang-Ming University, Taipei, Taiwan, China.; (Ho KY) Pain Management Service, Raffles Hospital, Singapore, Singapore.; (Tho LM) Department of Clinical Oncology, Beacon International Specialist Centre, Selangor, Malaysia.; (Xia ZJ) Sun Yat-Sen University Cancer Center, Guangzhou, China.; (Ward L) Mundipharma Research Ltd, Cambridge, United Kingdom.; (Moon H) Mundipharma Pte Ltd, Singapore, Singapore.; (Bhagat A) Mundipharma Pte Ltd, Singapore, Singapore.
(ACHEON Working Group) ACHEON Working Group; (Kim YC) Department of Anesthesiology and Pain Medicine, Seoul National University School of Medicine, Seoul, Korea.; (Ahn JS) Department of Medicine, Samsung Medical Center, Sungkyunkwan University School of Medicine, Seoul, Korea.; (Calimag MM) Departments of Pharmacology, Clinical Epidemiology and Anesthesiology, University of Santo Tomas Faculty of Medicine and Surgery and the UST Hospital, Manila, Philippines.; (Chao TC) Institute of Clinical Medicine, School of Medicine, National Yang-Ming University, Taipei, Taiwan, China.; (Ho KY) Pain Management Service, Raffles Hospital, Singapore, Singapore.; (Tho LM) Department of Clinical Oncology, Beacon International Specialist Centre, Selangor, Malaysia.; (Xia ZJ) Sun Yat-Sen University Cancer Center, Guangzhou, China.; (Ward L) Mundipharma Research Ltd, Cambridge, United Kingdom.; (Moon H) Mundipharma Pte Ltd, Singapore, Singapore.; (Bhagat A) Mundipharma Pte Ltd, Singapore, Singapore.

(ACHEON Working Group) ACHEON Working Group; (Kim YC) Department of Anesthesiology and Pain Medicine, Seoul National University School of Medicine, Seoul, Korea.; (Ahn JS) Department of Medicine, Samsung Medical Center, Sungkyunkwan University School of Medicine, Seoul, Korea.; (Calimag MM) Departments of Pharmacology, Clinical Epidemiology and Anesthesiology, University of Santo Tomas Faculty of Medicine and Surgery and the UST Hospital, Manila, Philippines.; (Chao TC) Institute of Clinical Medicine, School of Medicine, National Yang-Ming University, Taipei, Taiwan, China.; (Ho KY) Pain Management Service, Raffles Hospital, Singapore, Singapore.; (Tho LM) Department of Clinical Oncology, Beacon International Specialist Centre, Selangor, Malaysia.; (Xia ZJ) Sun Yat-Sen University Cancer Center, Guangzhou, China.; (Ward L) Mundipharma Research Ltd, Cambridge, United Kingdom.; (Moon H) Mundipharma Pte Ltd, Singapore, Singapore.; (Bhagat A) Mundipharma Pte Ltd, Singapore, Singapore.
(ACHEON Working Group) ACHEON Working Group; (Kim YC) Department of Anesthesiology and Pain Medicine, Seoul National University School of Medicine, Seoul, Korea.; (Ahn JS) Department of Medicine, Samsung Medical Center, Sungkyunkwan University School of Medicine, Seoul, Korea.; (Calimag MM) Departments of Pharmacology, Clinical Epidemiology and Anesthesiology, University of Santo Tomas Faculty of Medicine and Surgery and the UST Hospital, Manila, Philippines.; (Chao TC) Institute of Clinical Medicine, School of Medicine, National Yang-Ming University, Taipei, Taiwan, China.; (Ho KY) Pain Management Service, Raffles Hospital, Singapore, Singapore.; (Tho LM) Department of Clinical Oncology, Beacon International Specialist Centre, Selangor, Malaysia.; (Xia ZJ) Sun Yat-Sen University Cancer Center, Guangzhou, China.; (Ward L) Mundipharma Research Ltd, Cambridge, United Kingdom.; (Moon H) Mundipharma Pte Ltd, Singapore, Singapore.; (Bhagat A) Mundipharma Pte Ltd, Singapore, Singapore.
(ACHEON Working Group) ACHEON Working Group; (Kim YC) Department of Anesthesiology and Pain Medicine, Seoul National University School of Medicine, Seoul, Korea.; (Ahn JS) Department of Medicine, Samsung Medical Center, Sungkyunkwan University School of Medicine, Seoul, Korea.; (Calimag MM) Departments of Pharmacology, Clinical Epidemiology and Anesthesiology, University of Santo Tomas Faculty of Medicine and Surgery and the UST Hospital, Manila, Philippines.; (Chao TC) Institute of Clinical Medicine, School of Medicine, National Yang-Ming University, Taipei, Taiwan, China.; (Ho KY) Pain Management Service, Raffles Hospital, Singapore, Singapore.; (Tho LM) Department of Clinical Oncology, Beacon International Specialist Centre, Selangor, Malaysia.; (Xia ZJ) Sun Yat-Sen University Cancer Center, Guangzhou, China.; (Ward L) Mundipharma Research Ltd, Cambridge, United Kingdom.; (Moon H) Mundipharma Pte Ltd, Singapore, Singapore.; (Bhagat A) Mundipharma Pte Ltd, Singapore, Singapore.
(ACHEON Working Group) ACHEON Working Group; (Kim YC) Department of Anesthesiology and Pain Medicine, Seoul National University School of Medicine, Seoul, Korea.; (Ahn JS) Department of Medicine, Samsung Medical Center, Sungkyunkwan University School of Medicine, Seoul, Korea.; (Calimag MM) Departments of Pharmacology, Clinical Epidemiology and Anesthesiology, University of Santo Tomas Faculty of Medicine and Surgery and the UST Hospital, Manila, Philippines.; (Chao TC) Institute of Clinical Medicine, School of Medicine, National Yang-Ming University, Taipei, Taiwan, China.; (Ho KY) Pain Management Service, Raffles Hospital, Singapore, Singapore.; (Tho LM) Department of Clinical Oncology, Beacon International Specialist Centre, Selangor, Malaysia.; (Xia ZJ) Sun Yat-Sen University Cancer Center, Guangzhou, China.; (Ward L) Mundipharma Research Ltd,

Cambridge, United Kingdom.; (Moon H) Mundipharma Pte Ltd, Singapore, Singapore.; (Bhagat A) Mundipharma Pte Ltd, Singapore, Singapore.
(ACHEON Working Group) ACHEON Working Group; (Kim YC) Department of Anesthesiology and Pain Medicine, Seoul National University School of Medicine, Seoul, Korea.; (Ahn JS) Department of Medicine, Samsung Medical Center, Sungkyunkwan University School of Medicine, Seoul, Korea.; (Calimag MM) Departments of Pharmacology, Clinical Epidemiology and Anesthesiology, University of Santo Tomas Faculty of Medicine and Surgery and the UST Hospital, Manila, Philippines.; (Chao TC) Institute of Clinical Medicine, School of Medicine, National Yang-Ming University, Taipei, Taiwan, China.; (Ho KY) Pain Management Service, Raffles Hospital, Singapore, Singapore.; (Tho LM) Department of Clinical Oncology, Beacon International Specialist Centre, Selangor, Malaysia.; (Xia ZJ) Sun Yat-Sen University Cancer Center, Guangzhou, China.; (Ward L) Mundipharma Research Ltd, Cambridge, United Kingdom.; (Moon H) Mundipharma Pte Ltd, Singapore, Singapore.; (Bhagat A) Mundipharma Pte Ltd, Singapore, Singapore.
(ACHEON Working Group) ACHEON Working Group; (Kim YC) Department of Anesthesiology and Pain Medicine, Seoul National University School of Medicine, Seoul, Korea.; (Ahn JS) Department of Medicine, Samsung Medical Center, Sungkyunkwan University School of Medicine, Seoul, Korea.; (Calimag MM) Departments of Pharmacology, Clinical Epidemiology and Anesthesiology, University of Santo Tomas Faculty of Medicine and Surgery and the UST Hospital, Manila, Philippines.; (Chao TC) Institute of Clinical Medicine, School of Medicine, National Yang-Ming University, Taipei, Taiwan, China.; (Ho KY) Pain Management Service, Raffles Hospital, Singapore, Singapore.; (Tho LM) Department of Clinical Oncology, Beacon International Specialist Centre, Selangor, Malaysia.; (Xia ZJ) Sun Yat-Sen University Cancer Center, Guangzhou, China.; (Ward L) Mundipharma Research Ltd, Cambridge, United Kingdom.; (Moon H) Mundipharma Pte Ltd, Singapore, Singapore.; (Bhagat A) Mundipharma Pte Ltd, Singapore, Singapore.
Database: PubMed

116. Evaluating resident physicians' knowledge, attitude, and practice regarding the pain control in cancer patients.

Author(s): Hashemi M; Akbari ME; Razavi SS; Saadat-Niaki A; Hoseini Khameneh SM

Source: Iranian journal of cancer prevention; 2015; vol. 8 (no. 1); p. 1-10

Publication Date: 2015
Publication Type(s): Journal Article
ISSN: 2008-2398
Place of Publication: Iran
PubMedID: 25821565
Accession Number: 25821565
Available at Iranian journal of cancer prevention - from EBSCO (CINAHL Complete)
Available at Iranian journal of cancer prevention - from PubMed Central
Available at Iranian journal of cancer prevention - from PubMed
Keywords: Subject Terms: Attitudes; Cancer pain control; Practice; knowledge
Abstract:BACKGROUND: Pain has been one of the most debilitating symptoms of cancer. The aim of this study was to evaluate residents' knowledge, attitude, and practice regarding pain control in cancer patients.METHODS: In a descriptive study, 69 randomly selected third-year various residents practicing in teaching hospitals of Shahid Beheshti School of medicine participated in this study. They have provided their demographic characteristics and completed a questionnaire, based on their "knowledge", "attitude" and "practice" regarding cancer pain and its management. Data analysis has performed using SPSS v.19. A p value of less than 0.05 has considered as significant.RESULTS: Obtained Data from 69 participants including 32 anesthesiology residents has included to our study. The average scores were 35.8±6.1 (ranging from 20 to 49) for the residents' attitude, 25.1±9.1 (ranging from 0 to 53) for their knowledge and 11.2±4.1 (ranging from 0 to 17) for their practice. The overall scores of the questions have related to attitude and knowledge were higher for residents of anesthesiology but the difference was not statistically significant (A: 37.1±4.9 vs. 34.7±6.8, p=0.106, K: 27.2±11.8 vs. 23.3±5.6, p=0.076). The average score for questions on physician' practice was significantly higher in residents of anesthesiology (P: 12.8±3.2 vs. 9.7±4.2, p=0.001).CONCLUSION: In order to provide patients with adequate pain relief, it has seemed advisable for medical schools to focus on improving the educational curriculum and integrating it into clinical practice.
Institutions:
(Hashemi M) Dept. of Anesthesiology, Shahid Beheshti University of Medical Sciences, Tehran, Iran.; (Akbari ME) Cancer Research Center, Shahid Beheshti University of Medical Sciences, Tehran, Iran.; (Razavi SS) Dept. of Anesthesiology, Shahid Beheshti University of Medical Sciences, Tehran, Iran.; (Saadat-Niaki A) Dept. of Anesthesiology, Shahid Beheshti University of Medical Sciences, Tehran, Iran.; (Hoseini Khameneh SM) Dept. of Orthopedi, Shahid Beheshti University of Medical Sciences, Tehran, Iran.
(Hashemi M) Dept. of Anesthesiology, Shahid Beheshti University of Medical Sciences, Tehran, Iran.; (Akbari ME) Cancer Research Center, Shahid Beheshti University of Medical Sciences, Tehran, Iran.; (Razavi SS) Dept. of Anesthesiology, Shahid Beheshti University of Medical Sciences, Tehran, Iran.; (Saadat-Niaki A) Dept. of Anesthesiology, Shahid Beheshti University of Medical Sciences, Tehran, Iran.; (Hoseini Khameneh SM) Dept. of Orthopedi, Shahid Beheshti University of Medical Sciences, Tehran, Iran.
(Hashemi M) Dept. of Anesthesiology, Shahid Beheshti University of Medical Sciences, Tehran, Iran.; (Akbari ME) Cancer Research Center, Shahid Beheshti

University of Medical Sciences, Tehran, Iran.; (Razavi SS) Dept. of Anesthesiology, Shahid Beheshti University of Medical Sciences, Tehran, Iran.; (Saadat-Niaki A) Dept. of Anesthesiology, Shahid Beheshti University of Medical Sciences, Tehran, Iran.; (Hoseini Khameneh SM) Dept. of Orthopedi, Shahid Beheshti University of Medical Sciences, Tehran, Iran.
(Hashemi M) Dept. of Anesthesiology, Shahid Beheshti University of Medical Sciences, Tehran, Iran.; (Akbari ME) Cancer Research Center, Shahid Beheshti University of Medical Sciences, Tehran, Iran.; (Razavi SS) Dept. of Anesthesiology, Shahid Beheshti University of Medical Sciences, Tehran, Iran.; (Saadat-Niaki A) Dept. of Anesthesiology, Shahid Beheshti University of Medical Sciences, Tehran, Iran.; (Hoseini Khameneh SM) Dept. of Orthopedi, Shahid Beheshti University of Medical Sciences, Tehran, Iran.
(Hashemi M) Dept. of Anesthesiology, Shahid Beheshti University of Medical Sciences, Tehran, Iran.; (Akbari ME) Cancer Research Center, Shahid Beheshti University of Medical Sciences, Tehran, Iran.; (Razavi SS) Dept. of Anesthesiology, Shahid Beheshti University of Medical Sciences, Tehran, Iran.; (Saadat-Niaki A) Dept. of Anesthesiology, Shahid Beheshti University of Medical Sciences, Tehran, Iran.; (Hoseini Khameneh SM) Dept. of Orthopedi, Shahid Beheshti University of Medical Sciences, Tehran, Iran.
Database: PubMed

117. Nurse-led educational interventions on cancer pain outcomes for oncology outpatients: a systematic review.

Author(s): Zhou L; Liu XL; Tan JY; Yu HP; Pratt J; Peng YQ

Source: International nursing review; Jun 2015; vol. 62 (no. 2); p. 218-230

Publication Date: Jun 2015

Publication Type(s): Journal Article; Research Support, Non-U.S. Gov't; Review; Systematic Review

DOI: http://dx.doi.org/10.1111/inr.12172

ISSN: 1466-7657

Place of Publication: England

PubMedID: 25711778

Accession Number: 25711778

Available at International nursing review - from Wiley Online Library Medicine and Nursing Collection 2020

Keywords: Subject Terms: Cancer Care; Nurse-Patient; Pain; Pain Management; Patient Teaching; Systematic Review

Abstract:BACKGROUND: Cancer pain management is still unsatisfactory, although some effective guidelines exist. Educational interventions are reported to be useful in pain relief for oncology outpatients.AIM: The aims of this systematic review were to evaluate the effects of nurse-led educational interventions on improving cancer pain outcomes for oncology patients, and to establish an effective cancer pain protocol for clinical nursing practice in China.METHODS: A three-step search strategy was utilized. Eight databases were searched using the standards provided by the Joanna Briggs Institute that guided article selection, critical appraisal, data collection and data synthesis.RESULTS: A total of 1093 studies were identified through a literature

search. Only six studies complied with the inclusion criteria and were found to be methodologically sound. In general, the included studies indicated positive results pertaining to patient's knowledge and attitudes towards analgesics and cancer pain management and decreased pain intensity. Studies reported minimal effects of intervention on anxiety, depression, satisfaction regarding cancer pain management and patient's quality of life.CONCLUSIONS: Educational interventions were reported as effective methods to improve cancer pain outcomes. Analysis of the six included studies demonstrated the overall positive effects of nurse-led educational interventions for improving cancer pain management.IMPLICATIONS FOR NURSING AND HEALTH POLICY: The results suggest that an effective cancer pain protocol for improving cancer pain management can be established in China.

Institutions:
(Zhou L) VIP Department, East Hospital, Shanghai, China.; (Liu XL) Tenth People's Hospital, Tongji University, Shanghai, China.; (Tan JY) School of Nursing, Fujian University of Traditional Chinese Medicine, Fuzhou, China.; (Yu HP) VIP Department, East Hospital, Shanghai, China.; (Pratt J) School of Nursing, Midwifery and Paramedicine, Australian Catholic University, Brisbane, Qld, Australia.; (Peng YQ) Department of Nursing, East Hospital, Shanghai, China.
(Zhou L) VIP Department, East Hospital, Shanghai, China.; (Liu XL) Tenth People's Hospital, Tongji University, Shanghai, China.; (Tan JY) School of Nursing, Fujian University of Traditional Chinese Medicine, Fuzhou, China.; (Yu HP) VIP Department, East Hospital, Shanghai, China.; (Pratt J) School of Nursing, Midwifery and Paramedicine, Australian Catholic University, Brisbane, Qld, Australia.; (Peng YQ) Department of Nursing, East Hospital, Shanghai, China.
(Zhou L) VIP Department, East Hospital, Shanghai, China.; (Liu XL) Tenth People's Hospital, Tongji University, Shanghai, China.; (Tan JY) School of Nursing, Fujian University of Traditional Chinese Medicine, Fuzhou, China.; (Yu HP) VIP Department, East Hospital, Shanghai, China.; (Pratt J) School of Nursing, Midwifery and Paramedicine, Australian Catholic University, Brisbane, Qld, Australia.; (Peng YQ) Department of Nursing, East Hospital, Shanghai, China.
(Zhou L) VIP Department, East Hospital, Shanghai, China.; (Liu XL) Tenth People's Hospital, Tongji University, Shanghai, China.; (Tan JY) School of Nursing, Fujian University of Traditional Chinese Medicine, Fuzhou, China.; (Yu HP) VIP Department, East Hospital, Shanghai, China.; (Pratt J) School of Nursing, Midwifery and Paramedicine, Australian Catholic University, Brisbane, Qld, Australia.; (Peng YQ) Department of Nursing, East Hospital, Shanghai, China.
(Zhou L) VIP Department, East Hospital, Shanghai, China.; (Liu XL) Tenth People's Hospital, Tongji University, Shanghai, China.; (Tan JY) School of Nursing, Fujian University of Traditional Chinese Medicine, Fuzhou, China.; (Yu HP) VIP Department, East Hospital, Shanghai, China.; (Pratt J) School of Nursing, Midwifery and Paramedicine, Australian Catholic University, Brisbane, Qld, Australia.; (Peng YQ) Department of Nursing, East Hospital, Shanghai, China.
(Zhou L) VIP Department, East Hospital, Shanghai, China.; (Liu XL) Tenth People's Hospital, Tongji University, Shanghai, China.; (Tan JY) School of Nursing, Fujian University of Traditional Chinese Medicine, Fuzhou, China.; (Yu HP) VIP Department, East Hospital, Shanghai, China.; (Pratt J) School of Nursing, Midwifery and Paramedicine, Australian Catholic University, Brisbane, Qld, Australia.; (Peng YQ) Department of Nursing, East Hospital, Shanghai, China.
Database: PubMed

118. A satisfaction survey on cancer pain management using a self-reporting pain assessment tool.

Author(s): Lim SN; Han HS; Lee KH; Lee SC; Kim J; Yun J; Park S; Park M; Choe Y; Ryoo HM; Lee K; Cho D; Zang DY; Choi J

Source: Journal of palliative medicine; Mar 2015; vol. 18 (no. 3); p. 225-231

Publication Date: Mar 2015

Publication Type(s): Journal Article; Research Support, Non-U.S. Gov't

DOI: http://dx.doi.org/10.1089/jpm.2014.0021

ISSN: 1557-7740

Place of Publication: United States

PubMedID: 25650504

Accession Number: 25650504

Abstract:BACKGROUND: Pain is one of the most common and distressing symptoms in patients with cancer, with a high prevalence of 90%. Appropriate pain assessment is very important in managing cancer pain.OBJECTIVE: The aims of this study were to (1) evaluate patient satisfaction with pain control therapy using a self-reporting pain assessment tool, (2) explore the usefulness of a self-reporting assessment tool for patients and physicians, and (3) evaluate patient perception of pain management and opioid analgesics.METHODS: We enrolled a total of 587 South Korean adult cancer patients hospitalized for five days or more. Pain assessment using a self-reporting pain assessment tool was performed by patients themselves from Day 1 to Day 5. The average pain intensity on a numeric rating scale (NRS) and the frequency of breakthrough pain between Day 1 and Day 5 were recorded with a self-reporting pain assessment tool. We evaluated patient satisfaction with pain control and the usefulness of a self-reporting pain assessment tool for patients and physicians on Day 5.RESULTS: Among the 587 enrolled patients, 551, excluding 36 patients who violated inclusion criteria, were analyzed. The pain satisfaction rate was 79.5%, and only 6.2% of assessed patients had a negative pain management index (PMI). However, symmetry analysis for pain intensity between patient and physician showed low agreement (kappa=0.21). The patients with dissatisfaction for cancer pain control expressed negative attitudes toward using opioid analgesics and misconceptions regarding pain management. The satisfaction for using a self-reporting pain assessment tool was 79.2% in patients and 86.4% in physicians, respectively.CONCLUSION: The use of a self-reporting pain assessment tool as a communication instrument provides an effective foundation for evaluating pain intensity in cancer pain management. A more individualized approach to patient education about pain management may improve patient outcome.

Institutions:
(Lim SN) 1 Department of Internal Medicine, Haeundae-Paik Hospital, College of Medicine Inje University , Busan, South Korea .

Database: PubMed

119. Intravenous lidocaine for cancer pain without electrocardiographic monitoring: a retrospective review.

Author(s): Peixoto RD; Hawley P

Source: Journal of palliative medicine; Apr 2015; vol. 18 (no. 4); p. 373-377

Publication Date: Apr 2015

Publication Type(s): Journal Article

DOI: http://dx.doi.org/10.1089/jpm.2014.0279

ISSN: 1557-7740

Place of Publication: United States

PubMedID: 25469808

Accession Number: 25469808

Abstract:BACKGROUND: Intravenous lidocaine infusion has been clearly demonstrated as effective for pain in randomized controlled trials, but the belief that cardiac monitoring is required for safe administration is a barrier to access in the palliative care setting. There are also multiple infusion protocols reported in the literature. We have been administering lidocaine infusions for severe cancer pain at the BC Cancer Agency (BCCA) since 2003, without electrocardiographic (ECG) monitoring. Our simple protocol is for 5 mg/kg to be infused over 1 hour, with the option for subsequent doses to be increased if necessary, up to a maximum of 10 mg/kg. Our aim with this study is to share 11 years of our experience with this protocol.METHODS: This is a retrospective case series. Records of patients who received at least one lidocaine infusion for pain between 2003 and 2013 at the BCCA were reviewed. The primary end points were the documentation of clinical benefit and adverse effects.RESULTS: A total of 122 lidocaine infusions were administered in 51 individual patients. Twenty-five (49%) had a major response, 12 (23.5%) had a minor response, and 14 (27.5%) were considered nonresponders. Twenty-two (43.1%) patients were noted to have some adverse effect during at least one of the infusions, but only 1 (1.9%) patient had the infusion permanently discontinued. The most common side effects were drowsiness (30.7%), perioral numbness (13.4%), nausea (5.7%), and minor fluctuations of blood pressure (3.8%).CONCLUSIONS: This case series demonstrates that our protocol of infusional lidocaine can be beneficial to patients with cancer with severe opioid-refractory pain, and can safely be administered with close observation and vital sign monitoring, without ECG monitoring. Lidocaine infusion is a useful option to consider when other pain treatments have not been successful. Although only approximately half of patients will respond well, there is little harm to be expected from a trial of lidocaine infusion and responders can be repeatedly treated. This treatment could be delivered in palliative care units, hospices, or even patients' homes, providing suitable nursing supervision can be provided.

Institutions:
(Peixoto RD) 1 Department of Medical Oncology, BC Cancer Agency, Vancouver Cancer Centre , Vancouver, British Columbia, Canada .

Database: PubMed

120. Educational gaps among healthcare providers: an institution needs assessment to improve pain management for postsurgical patients.

Author(s): González-Fernández M; Aboumatar H; Conti D; Patel AM; Purvin MA; Hanna M

Source: Journal of opioid management; 2014; vol. 10 (no. 5); p. 345-351

Publication Date: 2014

Publication Type(s): Journal Article

DOI: http://dx.doi.org/10.5055/jom.2014.0224

ISSN: 1551-7489

Place of Publication: United States

PubMedID: 25350476

Accession Number: 25350476

Abstract:OBJECTIVE: Nurses should be educated in pain management because they are more likely than other healthcare professionals to educate patients about their pain. The authors sought to identify the knowledge gaps in postoperative pain management among postsurgical nursing staff and the existence of institutional policies and educational programs to support them in delivering optimal pain management services.SETTING: Academic hospital.PARTICIPANTS: Two hundred seventy-seven registered nurses, nurse practitioners, nurse managers, physician assistants, and other health professionals.INTERVENTIONS: Nurses participated in an online, anonymous survey that consisted of 43 questions in two broad categories: (1) knowledge and attitudes about pain management and (2) institutional pain management and assessment.MAIN OUTCOME MEASURE: Knowledge base of surgical nursing staff regarding postoperative pain management.RESULTS: The overall mean knowledge score was 44.84 percent, with the highest percent of correct answers in the cancer pain category (54.03 percent). Recognition of signs and symptoms of pain had the lowest correct response rate (40.91 percent). Forty-nine percent of respondents reported that pain management protocols tailored to the specific population treated were available, 42 percent reported that patient pain education was always performed, 29 percent reported that they received regular training about pain management, and 17 percent had access to national pain management guidelines.CONCLUSION: The survey results demonstrate general gaps in pain management knowledge among nurses, particularly in recognizing signs and symptoms of pain. This work may guide the development of programs that improve postoperative pain management by increasing the frequency of nurses' pain education and improving the availability of pain-related policies and protocols.

Institutions:
(González-Fernández M) Department of Physical Medicine and Rehabilitation, Johns Hopkins University School of Medicine, Baltimore, Maryland.; (Aboumatar H) Department of Medicine, Armstrong Institute for Safety and Quality, Johns Hopkins University, Baltimore, Maryland.; (Conti D) Department of Nursing, Johns Hopkins University School of Medicine, Baltimore, Maryland.; (Patel AM) Department of Anesthesiology and Critical Care Medicine, Johns Hopkins University School of Medicine, Baltimore, Maryland.; (Purvin MA) Department of Anesthesiology and Critical Care Medicine, Johns Hopkins University School of Medicine, Baltimore, Maryland.; (Hanna M) Department of Anesthesiology and Critical Care Medicine, Johns Hopkins University School of Medicine, Baltimore, Maryland.
(González-Fernández M) Department of Physical Medicine and Rehabilitation, Johns Hopkins University School of Medicine, Baltimore, Maryland.; (Aboumatar H)

Department of Medicine, Armstrong Institute for Safety and Quality, Johns Hopkins University, Baltimore, Maryland.; (Conti D) Department of Nursing, Johns Hopkins University School of Medicine, Baltimore, Maryland.; (Patel AM) Department of Anesthesiology and Critical Care Medicine, Johns Hopkins University School of Medicine, Baltimore, Maryland.; (Purvin MA) Department of Anesthesiology and Critical Care Medicine, Johns Hopkins University School of Medicine, Baltimore, Maryland.; (Hanna M) Department of Anesthesiology and Critical Care Medicine, Johns Hopkins University School of Medicine, Baltimore, Maryland.
(González-Fernández M) Department of Physical Medicine and Rehabilitation, Johns Hopkins University School of Medicine, Baltimore, Maryland.; (Aboumatar H) Department of Medicine, Armstrong Institute for Safety and Quality, Johns Hopkins University, Baltimore, Maryland.; (Conti D) Department of Nursing, Johns Hopkins University School of Medicine, Baltimore, Maryland.; (Patel AM) Department of Anesthesiology and Critical Care Medicine, Johns Hopkins University School of Medicine, Baltimore, Maryland.; (Purvin MA) Department of Anesthesiology and Critical Care Medicine, Johns Hopkins University School of Medicine, Baltimore, Maryland.; (Hanna M) Department of Anesthesiology and Critical Care Medicine, Johns Hopkins University School of Medicine, Baltimore, Maryland.
(González-Fernández M) Department of Physical Medicine and Rehabilitation, Johns Hopkins University School of Medicine, Baltimore, Maryland.; (Aboumatar H) Department of Medicine, Armstrong Institute for Safety and Quality, Johns Hopkins University, Baltimore, Maryland.; (Conti D) Department of Nursing, Johns Hopkins University School of Medicine, Baltimore, Maryland.; (Patel AM) Department of Anesthesiology and Critical Care Medicine, Johns Hopkins University School of Medicine, Baltimore, Maryland.; (Purvin MA) Department of Anesthesiology and Critical Care Medicine, Johns Hopkins University School of Medicine, Baltimore, Maryland.; (Hanna M) Department of Anesthesiology and Critical Care Medicine, Johns Hopkins University School of Medicine, Baltimore, Maryland.
(González-Fernández M) Department of Physical Medicine and Rehabilitation, Johns Hopkins University School of Medicine, Baltimore, Maryland.; (Aboumatar H) Department of Medicine, Armstrong Institute for Safety and Quality, Johns Hopkins University, Baltimore, Maryland.; (Conti D) Department of Nursing, Johns Hopkins University School of Medicine, Baltimore, Maryland.; (Patel AM) Department of Anesthesiology and Critical Care Medicine, Johns Hopkins University School of Medicine, Baltimore, Maryland.; (Purvin MA) Department of Anesthesiology and Critical Care Medicine, Johns Hopkins University School of Medicine, Baltimore, Maryland.; (Hanna M) Department of Anesthesiology and Critical Care Medicine, Johns Hopkins University School of Medicine, Baltimore, Maryland.
Database: PubMed

121. Improving cancer pain control with NCCN guideline-based analgesic administration: a patient-centered outcome.

Author(s): Janjan N

Source: Journal of the National Comprehensive Cancer Network : JNCCN; Sep 2014; vol. 12 (no. 9); p. 1243-1249

Publication Date: Sep 2014

Publication Type(s): Letter

DOI: http://dx.doi.org/10.6004/jnccn.2014.0122

ISSN: 1540-1413

Place of Publication: United States

PubMedID: 25190693

Accession Number: 25190693

Abstract: Improving the control of cancer-related pain (CRP) is a clinical and ethical imperative. Clinical research has documented improved treatment tolerance and survival rates among patients with cancer who have effective pain control. Barriers to CRP control include inadequate patient and physician education. Meta-analyses of patient education studies correlate improvements in CRP control with improved communications with health care providers and the implementation of strategies that assist with adherence to medication schedules. These strategies build patient confidence, allowing better self-management of pain and reduced psychological consequences. For physicians, ample educational resources exist in CRP management. However, in both the inpatient and outpatient settings, compliance with NCCN Clinical Practice Guidelines in Oncology for Adult Cancer Pain continues to be less than 70%, and more than one-third of patients continue to receive inadequate doses of analgesics. Patient-centered outcomes have become an integral end point in health policy, and the nation's medical training, research, and delivery systems are transforming to a value-based accreditation and reimbursement system. Pain control is a significant patient-centered outcome in cancer care, because pain adversely impacts function and affects all domains of quality of life. Agreement is clear on the value of health care interventions that relieve suffering from cancer pain and restore personal dignity.

Institutions:
(Janjan N) From the National Center for Policy Analysis, Dallas, Texas.
Database: PubMed

122. Management of persistent pain in the older patient: a clinical review.

Author(s): Makris UE; Abrams RC; Gurland B; Reid MC

Source: JAMA; Aug 2014; vol. 312 (no. 8); p. 825-836

Publication Date: Aug 2014

Publication Type(s): Case Reports; Journal Article; Research Support, N.I.H., Extramural; Research Support, Non-U.S. Gov't; Research Support, U.S. Gov't, P.H.S.; Review

DOI: http://dx.doi.org/10.1001/jama.2014.9405

ISSN: 1538-3598

Place of Publication: United States

PubMedID: 25157726

Accession Number: 25157726

Available at JAMA - from EBSCO (MEDLINE Complete)

Available at JAMA - from Unpaywall

Abstract:IMPORTANCE: Persistent pain is highly prevalent, costly, and frequently disabling in later life.OBJECTIVE: To describe barriers to the management of persistent pain among older adults, summarize current management approaches, including pharmacologic and nonpharmacologic modalities; present rehabilitative approaches; and highlight aspects of the patient-physician relationship that can help to improve treatment outcomes. This review is relevant for physicians who seek an age-appropriate approach to delivering pain care for the older adult.EVIDENCE ACQUISITION: Search of MEDLINE and the Cochrane database from January 1990 through May 2014, using the search terms older adults, senior, ages 65 and above, elderly, and aged along with non-cancer pain, chronic pain, persistent pain, pain management, intractable pain, and refractory pain to identify English-language peer-reviewed systematic reviews, meta-analyses, Cochrane reviews, consensus statements, and guidelines relevant to the management of persistent pain in older adults.FINDINGS: Of the 92 identified studies, 35 evaluated pharmacologic interventions, whereas 57 examined nonpharmacologic modalities; the majority (n = 50) focused on older adults with osteoarthritis. This evidence base supports a stepwise approach with acetaminophen as first-line therapy. If treatment goals are not met, a trial of a topical nonsteroidal anti-inflammatory drug, tramadol, or both is recommended. Oral nonsteroidal anti-inflammatory drugs are not recommended for long-term use. Careful surveillance to monitor for toxicity and efficacy is critical, given that advancing age increases risk for adverse effects. A multimodal approach is strongly recommended-emphasizing a combination of both pharmacologic and nonpharmacologic treatments to include physical and occupational rehabilitation, as well as cognitive-behavioral and movement-based interventions. An integrated pain management approach is ideally achieved by cultivating a strong therapeutic alliance between the older patient and the physician.CONCLUSIONS AND RELEVANCE: Treatment planning for persistent pain in later life requires a clear understanding of the patient's treatment goals and expectations, comorbidities, and cognitive and functional status, as well as coordinating community resources and family support when available. A combination of pharmacologic, nonpharmacologic, and rehabilitative approaches in addition to a strong therapeutic alliance between the patient and physician is essential in setting, adjusting, and achieving realistic goals of therapy.

Institutions:
(Makris UE) Department of Internal Medicine, Division of Rheumatic Diseases, UT Southwestern Medical Center, Dallas, Texas2Department of Medicine, Division of Rheumatology, Veterans Administration Medical Center, Dallas, Texas.; (Abrams RC) Department of Psychiatry, Weill Cornell Medical College, New York, New York4Division of Geriatrics and Palliative Medicine, Weill Cornell Medical College, New York, New York.; (Gurland B) Stroud Center, Columbia University, New York, New York.; (Reid MC) Division of Geriatrics and Palliative Medicine, Weill Cornell Medical College, New York, New York.

(Makris UE) Department of Internal Medicine, Division of Rheumatic Diseases, UT Southwestern Medical Center, Dallas, Texas2Department of Medicine, Division of Rheumatology, Veterans Administration Medical Center, Dallas, Texas.; (Abrams RC) Department of Psychiatry, Weill Cornell Medical College, New York, New York4Division of Geriatrics and Palliative Medicine, Weill Cornell Medical College, New York, New York.; (Gurland B) Stroud Center, Columbia University, New York, New York.; (Reid MC) Division of Geriatrics and Palliative Medicine, Weill Cornell Medical College, New York, New York.
(Makris UE) Department of Internal Medicine, Division of Rheumatic Diseases, UT Southwestern Medical Center, Dallas, Texas2Department of Medicine, Division of Rheumatology, Veterans Administration Medical Center, Dallas, Texas.; (Abrams RC) Department of Psychiatry, Weill Cornell Medical College, New York, New York4Division of Geriatrics and Palliative Medicine, Weill Cornell Medical College, New York, New York.; (Gurland B) Stroud Center, Columbia University, New York, New York.; (Reid MC) Division of Geriatrics and Palliative Medicine, Weill Cornell Medical College, New York, New York.
(Makris UE) Department of Internal Medicine, Division of Rheumatic Diseases, UT Southwestern Medical Center, Dallas, Texas2Department of Medicine, Division of Rheumatology, Veterans Administration Medical Center, Dallas, Texas.; (Abrams RC) Department of Psychiatry, Weill Cornell Medical College, New York, New York4Division of Geriatrics and Palliative Medicine, Weill Cornell Medical College, New York, New York.; (Gurland B) Stroud Center, Columbia University, New York, New York.; (Reid MC) Division of Geriatrics and Palliative Medicine, Weill Cornell Medical College, New York, New York.
Database: PubMed

123. Knowledge, practices, and perceived barriers regarding cancer pain management among physicians and nurses in Korea: a nationwide multicenter survey.

Author(s): Jho HJ; Kim Y; Kong KA; Kim DH; Choi JY; Nam EJ; Koh S; Hwang KO; Baek SK; Park EJ

Source: PloS one; 2014; vol. 9 (no. 8); p. e105900

Publication Date: 2014

Publication Type(s): Clinical Trial; Journal Article; Multicenter Study; Research Support, Non-U.S. Gov't

DOI: http://dx.doi.org/10.1371/journal.pone.0105900

ISSN: 1932-6203

Place of Publication: United States

PubMedID: 25144641

Accession Number: 25144641

Available at PloS one - from Europe PubMed Central - Open Access

Available at PloS one - from Public Library of Science (PLoS)

Available at PloS one - from DOAJ - Directory of Open Access Journals

Available at PloS one - from ProQuest (MEDLINE with Full Text) - NHS Version

Available at PloS one - from EBSCO (MEDLINE Complete)

Available at PloS one - from ProQuest (Health Research Premium) - NHS Version

Available at PloS one - from Unpaywall

Abstract:PURPOSE: Medical professionals' practices and knowledge regarding cancer pain management have often been cited as inadequate. This study aimed to evaluate knowledge, practices and perceived barriers regarding cancer pain management among physicians and nurses in Korea.METHODS: A nationwide questionnaire survey was administered to physicians and nurses involved in the care of cancer patients. Questionnaire items covered pain assessment and documentation practices, knowledge regarding cancer pain management, the perceived barriers to cancer pain control, and processes perceived as the major causes of delay in opioid administration.RESULTS: A total of 333 questionnaires (149 physicians and 284 nurses) were analyzed. Nurses performed pain assessment and documentation more regularly than physicians did. Although physicians had better knowledge of pain management than did nurses, both groups lacked knowledge regarding the side effects and pharmacology of opioids. Physicians working in the palliative care ward and nurses who had received pain management education obtained higher scores on knowledge. Physicians perceived patients' reluctance to take opioids as a barrier to pain control, more so than did nurses, while nurses perceived patients' tendency to under-report of pain as a barrier, more so than did physicians. Physicians and nurses held different perceptions regarding major cause of delay during opioid administration.CONCLUSIONS: There were differences between physicians and nurses in knowledge and practices for cancer pain management. An effective educational strategy for cancer pain management is needed in order to improve medical professionals' knowledge and clinical practices.

Institutions:
(Jho HJ) Hospice & Palliative Care Branch, National Cancer Control Institute, National Cancer Center, Goyang, Republic of Korea.; (Kim Y) Division of Cancer Management & Policy, National Cancer Control Institute, National Cancer Center, Goyang, Republic of Korea.; (Kong KA) Clinical Trial Center, Ewha Womans University Medical Center, Seoul, Republic of Korea.; (Kim DH) Department of Pain and Anesthesiology, Hospital, National Cancer Center, Goyang, Republic of Korea.; (Choi JY) Hospice & Palliative Care Branch, National Cancer Control Institute, National Cancer Center, Goyang, Republic of Korea.; (Nam EJ) Department of Nursing, Hospital, National Cancer Center, Goyang, Republic of Korea.; (Choi JY) Department of Nursing, Hospital, National Cancer Center, Goyang, Republic of Korea.; (Koh S) Department of Hematology and Oncology, Ulsan University Hospital, Ulsan, Republic of Korea.; (Hwang KO) Korean Hospice & Palliative Nurse Association, Daejeon, Republic of Korea.; (Baek SK) Department of Medical Oncology and Hematology, Kyung Hee University Medical Center, Seoul, Republic of Korea.; (Park EJ) Hospice & Palliative Care Branch, National Cancer Control Institute, National Cancer Center, Goyang, Republic of Korea.
(Jho HJ) Hospice & Palliative Care Branch, National Cancer Control Institute, National Cancer Center, Goyang, Republic of Korea.; (Kim Y) Division of Cancer Management & Policy, National Cancer Control Institute, National Cancer Center, Goyang, Republic of Korea.; (Kong KA) Clinical Trial Center, Ewha Womans University Medical Center, Seoul, Republic of Korea.; (Kim DH) Department of Pain and Anesthesiology, Hospital, National Cancer Center, Goyang, Republic of Korea.; (Choi JY) Hospice & Palliative Care Branch, National Cancer Control Institute, National Cancer Center, Goyang, Republic of Korea.; (Nam EJ) Department of

Nursing, Hospital, National Cancer Center, Goyang, Republic of Korea.; (Choi JY) Department of Nursing, Hospital, National Cancer Center, Goyang, Republic of Korea.; (Koh S) Department of Hematology and Oncology, Ulsan University Hospital, Ulsan, Republic of Korea.; (Hwang KO) Korean Hospice & Palliative Nurse Association, Daejeon, Republic of Korea.; (Baek SK) Department of Medical Oncology and Hematology, Kyung Hee University Medical Center, Seoul, Republic of Korea.; (Park EJ) Hospice & Palliative Care Branch, National Cancer Control Institute, National Cancer Center, Goyang, Republic of Korea.

(Jho HJ) Hospice & Palliative Care Branch, National Cancer Control Institute, National Cancer Center, Goyang, Republic of Korea.; (Kim Y) Division of Cancer Management & Policy, National Cancer Control Institute, National Cancer Center, Goyang, Republic of Korea.; (Kong KA) Clinical Trial Center, Ewha Womans University Medical Center, Seoul, Republic of Korea.; (Kim DH) Department of Pain and Anesthesiology, Hospital, National Cancer Center, Goyang, Republic of Korea.; (Choi JY) Hospice & Palliative Care Branch, National Cancer Control Institute, National Cancer Center, Goyang, Republic of Korea.; (Nam EJ) Department of Nursing, Hospital, National Cancer Center, Goyang, Republic of Korea.; (Choi JY) Department of Nursing, Hospital, National Cancer Center, Goyang, Republic of Korea.; (Koh S) Department of Hematology and Oncology, Ulsan University Hospital, Ulsan, Republic of Korea.; (Hwang KO) Korean Hospice & Palliative Nurse Association, Daejeon, Republic of Korea.; (Baek SK) Department of Medical Oncology and Hematology, Kyung Hee University Medical Center, Seoul, Republic of Korea.; (Park EJ) Hospice & Palliative Care Branch, National Cancer Control Institute, National Cancer Center, Goyang, Republic of Korea.

(Jho HJ) Hospice & Palliative Care Branch, National Cancer Control Institute, National Cancer Center, Goyang, Republic of Korea.; (Kim Y) Division of Cancer Management & Policy, National Cancer Control Institute, National Cancer Center, Goyang, Republic of Korea.; (Kong KA) Clinical Trial Center, Ewha Womans University Medical Center, Seoul, Republic of Korea.; (Kim DH) Department of Pain and Anesthesiology, Hospital, National Cancer Center, Goyang, Republic of Korea.; (Choi JY) Hospice & Palliative Care Branch, National Cancer Control Institute, National Cancer Center, Goyang, Republic of Korea.; (Nam EJ) Department of Nursing, Hospital, National Cancer Center, Goyang, Republic of Korea.; (Choi JY) Department of Nursing, Hospital, National Cancer Center, Goyang, Republic of Korea.; (Koh S) Department of Hematology and Oncology, Ulsan University Hospital, Ulsan, Republic of Korea.; (Hwang KO) Korean Hospice & Palliative Nurse Association, Daejeon, Republic of Korea.; (Baek SK) Department of Medical Oncology and Hematology, Kyung Hee University Medical Center, Seoul, Republic of Korea.; (Park EJ) Hospice & Palliative Care Branch, National Cancer Control Institute, National Cancer Center, Goyang, Republic of Korea.

(Jho HJ) Hospice & Palliative Care Branch, National Cancer Control Institute, National Cancer Center, Goyang, Republic of Korea.; (Kim Y) Division of Cancer Management & Policy, National Cancer Control Institute, National Cancer Center, Goyang, Republic of Korea.; (Kong KA) Clinical Trial Center, Ewha Womans University Medical Center, Seoul, Republic of Korea.; (Kim DH) Department of Pain and Anesthesiology, Hospital, National Cancer Center, Goyang, Republic of Korea.; (Choi JY) Hospice & Palliative Care Branch, National Cancer Control Institute, National Cancer Center, Goyang, Republic of Korea.; (Nam EJ) Department of Nursing, Hospital, National Cancer Center, Goyang, Republic of Korea.; (Choi JY) Department of Nursing, Hospital, National Cancer Center, Goyang, Republic of

Korea.; (Koh S) Department of Hematology and Oncology, Ulsan University Hospital, Ulsan, Republic of Korea.; (Hwang KO) Korean Hospice & Palliative Nurse Association, Daejeon, Republic of Korea.; (Baek SK) Department of Medical Oncology and Hematology, Kyung Hee University Medical Center, Seoul, Republic of Korea.; (Park EJ) Hospice & Palliative Care Branch, National Cancer Control Institute, National Cancer Center, Goyang, Republic of Korea.
(Jho HJ) Hospice & Palliative Care Branch, National Cancer Control Institute, National Cancer Center, Goyang, Republic of Korea.; (Kim Y) Division of Cancer Management & Policy, National Cancer Control Institute, National Cancer Center, Goyang, Republic of Korea.; (Kong KA) Clinical Trial Center, Ewha Womans University Medical Center, Seoul, Republic of Korea.; (Kim DH) Department of Pain and Anesthesiology, Hospital, National Cancer Center, Goyang, Republic of Korea.; (Choi JY) Hospice & Palliative Care Branch, National Cancer Control Institute, National Cancer Center, Goyang, Republic of Korea.; (Nam EJ) Department of Nursing, Hospital, National Cancer Center, Goyang, Republic of Korea.; (Choi JY) Department of Nursing, Hospital, National Cancer Center, Goyang, Republic of Korea.; (Koh S) Department of Hematology and Oncology, Ulsan University Hospital, Ulsan, Republic of Korea.; (Hwang KO) Korean Hospice & Palliative Nurse Association, Daejeon, Republic of Korea.; (Baek SK) Department of Medical Oncology and Hematology, Kyung Hee University Medical Center, Seoul, Republic of Korea.; (Park EJ) Hospice & Palliative Care Branch, National Cancer Control Institute, National Cancer Center, Goyang, Republic of Korea.
(Jho HJ) Hospice & Palliative Care Branch, National Cancer Control Institute, National Cancer Center, Goyang, Republic of Korea.; (Kim Y) Division of Cancer Management & Policy, National Cancer Control Institute, National Cancer Center, Goyang, Republic of Korea.; (Kong KA) Clinical Trial Center, Ewha Womans University Medical Center, Seoul, Republic of Korea.; (Kim DH) Department of Pain and Anesthesiology, Hospital, National Cancer Center, Goyang, Republic of Korea.; (Choi JY) Hospice & Palliative Care Branch, National Cancer Control Institute, National Cancer Center, Goyang, Republic of Korea.; (Nam EJ) Department of Nursing, Hospital, National Cancer Center, Goyang, Republic of Korea.; (Choi JY) Department of Nursing, Hospital, National Cancer Center, Goyang, Republic of Korea.; (Koh S) Department of Hematology and Oncology, Ulsan University Hospital, Ulsan, Republic of Korea.; (Hwang KO) Korean Hospice & Palliative Nurse Association, Daejeon, Republic of Korea.; (Baek SK) Department of Medical Oncology and Hematology, Kyung Hee University Medical Center, Seoul, Republic of Korea.; (Park EJ) Hospice & Palliative Care Branch, National Cancer Control Institute, National Cancer Center, Goyang, Republic of Korea.
(Jho HJ) Hospice & Palliative Care Branch, National Cancer Control Institute, National Cancer Center, Goyang, Republic of Korea.; (Kim Y) Division of Cancer Management & Policy, National Cancer Control Institute, National Cancer Center, Goyang, Republic of Korea.; (Kong KA) Clinical Trial Center, Ewha Womans University Medical Center, Seoul, Republic of Korea.; (Kim DH) Department of Pain and Anesthesiology, Hospital, National Cancer Center, Goyang, Republic of Korea.; (Choi JY) Hospice & Palliative Care Branch, National Cancer Control Institute, National Cancer Center, Goyang, Republic of Korea.; (Nam EJ) Department of Nursing, Hospital, National Cancer Center, Goyang, Republic of Korea.; (Choi JY) Department of Nursing, Hospital, National Cancer Center, Goyang, Republic of Korea.; (Koh S) Department of Hematology and Oncology, Ulsan University Hospital, Ulsan, Republic of Korea.; (Hwang KO) Korean Hospice & Palliative Nurse

Association, Daejeon, Republic of Korea.; (Baek SK) Department of Medical Oncology and Hematology, Kyung Hee University Medical Center, Seoul, Republic of Korea.; (Park EJ) Hospice & Palliative Care Branch, National Cancer Control Institute, National Cancer Center, Goyang, Republic of Korea.
(Jho HJ) Hospice & Palliative Care Branch, National Cancer Control Institute, National Cancer Center, Goyang, Republic of Korea.; (Kim Y) Division of Cancer Management & Policy, National Cancer Control Institute, National Cancer Center, Goyang, Republic of Korea.; (Kong KA) Clinical Trial Center, Ewha Womans University Medical Center, Seoul, Republic of Korea.; (Kim DH) Department of Pain and Anesthesiology, Hospital, National Cancer Center, Goyang, Republic of Korea.; (Choi JY) Hospice & Palliative Care Branch, National Cancer Control Institute, National Cancer Center, Goyang, Republic of Korea.; (Nam EJ) Department of Nursing, Hospital, National Cancer Center, Goyang, Republic of Korea.; (Choi JY) Department of Nursing, Hospital, National Cancer Center, Goyang, Republic of Korea.; (Koh S) Department of Hematology and Oncology, Ulsan University Hospital, Ulsan, Republic of Korea.; (Hwang KO) Korean Hospice & Palliative Nurse Association, Daejeon, Republic of Korea.; (Baek SK) Department of Medical Oncology and Hematology, Kyung Hee University Medical Center, Seoul, Republic of Korea.; (Park EJ) Hospice & Palliative Care Branch, National Cancer Control Institute, National Cancer Center, Goyang, Republic of Korea.
(Jho HJ) Hospice & Palliative Care Branch, National Cancer Control Institute, National Cancer Center, Goyang, Republic of Korea.; (Kim Y) Division of Cancer Management & Policy, National Cancer Control Institute, National Cancer Center, Goyang, Republic of Korea.; (Kong KA) Clinical Trial Center, Ewha Womans University Medical Center, Seoul, Republic of Korea.; (Kim DH) Department of Pain and Anesthesiology, Hospital, National Cancer Center, Goyang, Republic of Korea.; (Choi JY) Hospice & Palliative Care Branch, National Cancer Control Institute, National Cancer Center, Goyang, Republic of Korea.; (Nam EJ) Department of Nursing, Hospital, National Cancer Center, Goyang, Republic of Korea.; (Choi JY) Department of Nursing, Hospital, National Cancer Center, Goyang, Republic of Korea.; (Koh S) Department of Hematology and Oncology, Ulsan University Hospital, Ulsan, Republic of Korea.; (Hwang KO) Korean Hospice & Palliative Nurse Association, Daejeon, Republic of Korea.; (Baek SK) Department of Medical Oncology and Hematology, Kyung Hee University Medical Center, Seoul, Republic of Korea.; (Park EJ) Hospice & Palliative Care Branch, National Cancer Control Institute, National Cancer Center, Goyang, Republic of Korea.
(Jho HJ) Hospice & Palliative Care Branch, National Cancer Control Institute, National Cancer Center, Goyang, Republic of Korea.; (Kim Y) Division of Cancer Management & Policy, National Cancer Control Institute, National Cancer Center, Goyang, Republic of Korea.; (Kong KA) Clinical Trial Center, Ewha Womans University Medical Center, Seoul, Republic of Korea.; (Kim DH) Department of Pain and Anesthesiology, Hospital, National Cancer Center, Goyang, Republic of Korea.; (Choi JY) Hospice & Palliative Care Branch, National Cancer Control Institute, National Cancer Center, Goyang, Republic of Korea.; (Nam EJ) Department of Nursing, Hospital, National Cancer Center, Goyang, Republic of Korea.; (Choi JY) Department of Nursing, Hospital, National Cancer Center, Goyang, Republic of Korea.; (Koh S) Department of Hematology and Oncology, Ulsan University Hospital, Ulsan, Republic of Korea.; (Hwang KO) Korean Hospice & Palliative Nurse Association, Daejeon, Republic of Korea.; (Baek SK) Department of Medical Oncology and Hematology, Kyung Hee University Medical Center, Seoul, Republic of

Korea.; (Park EJ) Hospice & Palliative Care Branch, National Cancer Control Institute, National Cancer Center, Goyang, Republic of Korea.
Database: PubMed

124. Use of Opioids and Sedatives at End-of-Life.

Author(s): Sim SW; Ho S; Kumar RK

Source: Indian journal of palliative care; May 2014; vol. 20 (no. 2); p. 160-165

Publication Date: May 2014

Publication Type(s): Journal Article

DOI: http://dx.doi.org/10.4103/0973-1075.132654

ISSN: 0973-1075

Place of Publication: United States

PubMedID: 25125876

Accession Number: 25125876

Available at Indian journal of palliative care - from Europe PubMed Central - Open Access

Available at Indian journal of palliative care - from EBSCO (CINAHL Complete)

Available at Indian journal of palliative care - from ProQuest (Health Research Premium) - NHS Version

Available at Indian journal of palliative care - from Unpaywall

Keywords: Subject Terms: Asia; End-of-life care; Opioids; Sedatives; Singapore

Abstract: Despite their proven efficacy and safety, opioid and sedative use for palliation in patients afflicted with cancer in Singapore have been shown to be a fraction of that in other countries. This paper explores the various psychosocial and system-related factors that appear to propagate this conservative approach to care in what is largely a western-influenced care practice. A search for publications relating to sedative and opioid usage in Asia was performed on PubMed, Google, Google Scholar, World Health Organization, and Singapore's government agency websites using search terms such as "opioids," "sedatives," "palliation," "end-of-life-care," "pain management," "palliative care," "cancer pain," "Asia," "Singapore," and "morphine." Findings were classified into three broad groups - system-related, physician-related, and patient-related factors. A cautious medico-legal climate, shortage of physicians trained in palliative care, and lack of instruments for symptom assessment of patients at the end of life contribute to system-related barriers. Physician-related barriers include delayed access to palliative care due to late referrals, knowledge deficits in non-palliative medicine physicians, and sub-optimal care provided by palliative physicians. Patients' under-reporting of symptoms and fear of addiction, tolerance, and side effects of opioids and sedatives may lead to conservative opioid use in palliative care as well. System-related, physician-related, and patient-related factors play crucial roles in steering the management of palliative patients. Addressing and increasing the awareness of these factors may help ensure patients receive adequate relief and control of distressing symptoms.

Institutions:

(Sim SW) Department of Palliative Medicine, National Cancer Center, Singapore 11 Hospital Drive, Singapore.; (Ho S) Department of Palliative Medicine, National Cancer Center, Singapore 11 Hospital Drive, Singapore.; (Kumar RK) Department of Palliative Medicine, National Cancer Center, Singapore 11 Hospital Drive, Singapore.
(Sim SW) Department of Palliative Medicine, National Cancer Center, Singapore 11 Hospital Drive, Singapore.; (Ho S) Department of Palliative Medicine, National Cancer Center, Singapore 11 Hospital Drive, Singapore.; (Kumar RK) Department of Palliative Medicine, National Cancer Center, Singapore 11 Hospital Drive, Singapore.
(Sim SW) Department of Palliative Medicine, National Cancer Center, Singapore 11 Hospital Drive, Singapore.; (Ho S) Department of Palliative Medicine, National Cancer Center, Singapore 11 Hospital Drive, Singapore.; (Kumar RK) Department of Palliative Medicine, National Cancer Center, Singapore 11 Hospital Drive, Singapore.
Database: PubMed

125. Breakthrough cancer pain (BTcP): a synthesis of taxonomy, pathogenesis, therapy, and good clinical practice in adult patients in Italy.

Author(s): Zucco F; Bonezzi C; Fornasari D

Source: Advances in therapy; Jul 2014; vol. 31 (no. 7); p. 657-682

Publication Date: Jul 2014

Publication Type(s): Journal Article; Review

DOI: http://dx.doi.org/10.1007/s12325-014-0130-z

ISSN: 1865-8652

Place of Publication: United States

PubMedID: 25005168

Accession Number: 25005168

Available at Advances in therapy - from EBSCO (MEDLINE Complete)

Available at Advances in therapy - from Unpaywall

Abstract: Pain presents in 80% of patients with advanced cancer, and 30% have periods of increased pain due to fluctuating intensity, known as breakthrough cancer pain (BTcP). BTcP is high-intensity, short-duration pain occurring in several episodes per day and is non-responsive to treatment. The clinical approach to BTcP is variable. A review of the literature was performed to provide clinicians and practitioners with a rational synthesis of the ongoing scientific debate on BTcP and to provide a basis for optimal clinical approach to BTcP in adult Italian patients. Data show that circadian exacerbations of pain should be carefully monitored, differentiating, if possible, between fluctuations of background pain (BP), end-of-dose effect, and BTcP. BTcP should be monitored in all care contexts in clinical practice and each care facility must have all the medications and products approved for use in BTcP at their disposal. Data show that knowledge about medications for BTcP is lacking: medications for BTcP treatment are not interchangeable, although containing the same active substance; each physician must know the specific characteristics of each medication, its pharmacological properties, limitations in clinical practice, specifics relating to titration and repeatability of administration, and technical specifics relating to the accessibility and delivery. Importantly, before choosing a rapid-onset opioid (ROO), it is essential to deeply understand the status of patient and the characteristics of their family unit/caregivers, taking into account

the patient's progressive loss of autonomy and/or cognitive-relational functionality. When BTcP therapy is initiated or changed, special attention must be paid to training the patient and family members/caregivers, providing clear instructions regarding the timing of drug administration. The patient must already be treated effectively with opioids before introducing ROOs for control of BTcP.

Institutions:
(Zucco F) "Presenza Amica" Association, Piazza Piemonte 4, 20145, Milan, Italy, fzucco_1951@libero.it.
Database: PubMed

126. Healthcare providers' perspectives of the supportive care needs of men with advanced prostate cancer.

Author(s): Carter N; Miller PA; Murphy BR; Payne VJ; Bryant-Lukosius D

Source: Oncology nursing forum; Jul 2014; vol. 41 (no. 4); p. 421-430

Publication Date: Jul 2014

Publication Type(s): Journal Article

DOI: http://dx.doi.org/10.1188/14.ONF.421-430

ISSN: 1538-0688

Place of Publication: United States

PubMedID: 24969251

Accession Number: 24969251

Available at Oncology Nursing Forum - from Ovid (Journals @ Ovid)

Available at Oncology Nursing Forum - from EBSCO (CINAHL Complete)

Available at Oncology Nursing Forum - from ProQuest (MEDLINE with Full Text) - NHS Version

Available at Oncology Nursing Forum - from ProQuest (Health Research Premium) - NHS Version

Available at Oncology Nursing Forum - from David Adams Library Journals Collection Local Print Collection [location] : David Adams Library.

Keywords: Subject Terms: nursing research; professional issues; prostate cancer; qualitative

Abstract:PURPOSE/OBJECTIVES: To examine healthcare providers' (HCPs') perceptions of the supportive care needs of men with advanced prostate cancer (APC).RESEARCH APPROACH: A qualitative, descriptive study.SETTING: Healthcare facilities caring for men with APC in a south-central region of Ontario.PARTICIPANTS: 19 nurses, physicians, and allied health providers who cared for men with APC in outpatient settings.METHODOLOGIC APPROACH: Interviews and focus groups.FINDINGS: HCPs identified four themes related to men's supportive care needs: pain and symptom management, informational needs, emotional needs, and the need for practical assistance. HCPs emphasized issues related to pain, urinary incontinence, and fatigue. They also reported that men continually ask for more information related to treatment, side effects, and prognosis. PARTICIPANTS identified a variety of barriers in meeting supportive care needs, including lack of management strategies, poor knowledge retention, and the "stoic

and old-school" nature of men in this population.CONCLUSIONS: Supportive care for this population can be improved through more focused implementation of interprofessional care, with clearly defined professional roles and additional specialized roles to address prostate cancer pain, urinary incontinence, and fatigue.INTERPRETATION: Specialized roles in the management of pain, urinary incontinence, and fatigue affecting men with APC could be integrated into interprofessional care to meet supportive care needs.

Institutions:
(Carter N) School of Nursing, McMaster University in Hamilton, Ontario, Canada.; (Miller PA) School of Rehabilitation Science, McMaster University in Hamilton, Ontario, Canada.; (Murphy BR) School of Rehabilitation Science, McMaster University in Hamilton, Ontario, Canada.; (Payne VJ) School of Rehabilitation Science, McMaster University in Hamilton, Ontario, Canada.; (Bryant-Lukosius D) School of Nursing, McMaster University in Hamilton, Ontario, Canada.
(Carter N) School of Nursing, McMaster University in Hamilton, Ontario, Canada.; (Miller PA) School of Rehabilitation Science, McMaster University in Hamilton, Ontario, Canada.; (Murphy BR) School of Rehabilitation Science, McMaster University in Hamilton, Ontario, Canada.; (Payne VJ) School of Rehabilitation Science, McMaster University in Hamilton, Ontario, Canada.; (Bryant-Lukosius D) School of Nursing, McMaster University in Hamilton, Ontario, Canada.
(Carter N) School of Nursing, McMaster University in Hamilton, Ontario, Canada.; (Miller PA) School of Rehabilitation Science, McMaster University in Hamilton, Ontario, Canada.; (Murphy BR) School of Rehabilitation Science, McMaster University in Hamilton, Ontario, Canada.; (Payne VJ) School of Rehabilitation Science, McMaster University in Hamilton, Ontario, Canada.; (Bryant-Lukosius D) School of Nursing, McMaster University in Hamilton, Ontario, Canada.
(Carter N) School of Nursing, McMaster University in Hamilton, Ontario, Canada.; (Miller PA) School of Rehabilitation Science, McMaster University in Hamilton, Ontario, Canada.; (Murphy BR) School of Rehabilitation Science, McMaster University in Hamilton, Ontario, Canada.; (Payne VJ) School of Rehabilitation Science, McMaster University in Hamilton, Ontario, Canada.; (Bryant-Lukosius D) School of Nursing, McMaster University in Hamilton, Ontario, Canada.
(Carter N) School of Nursing, McMaster University in Hamilton, Ontario, Canada.; (Miller PA) School of Rehabilitation Science, McMaster University in Hamilton, Ontario, Canada.; (Murphy BR) School of Rehabilitation Science, McMaster University in Hamilton, Ontario, Canada.; (Payne VJ) School of Rehabilitation Science, McMaster University in Hamilton, Ontario, Canada.; (Bryant-Lukosius D) School of Nursing, McMaster University in Hamilton, Ontario, Canada.
Database: PubMed

127. A nationwide survey of knowledge of and compliance with cancer pain management guidelines by korean physicians.

Author(s): Kim DY; Ahn JS; Lee KH; Kim YC; Lee J; Kim SY

Source: Cancer research and treatment; Apr 2014; vol. 46 (no. 2); p. 131-140

Publication Date: Apr 2014

Publication Type(s): Journal Article

DOI: http://dx.doi.org/10.4143/crt.2014.46.2.131

ISSN: 1598-2998

Place of Publication: Korea (South)

PubMedID: 24851104

Accession Number: 24851104

Available at Cancer research and treatment : official journal of Korean Cancer Association - from Europe PubMed Central - Open Access

Available at Cancer research and treatment : official journal of Korean Cancer Association - from Unpaywall

Keywords: Subject Terms: Analgesics; Compliance; Guideline; Knowledge; Neoplasms; Pain

Abstract:PURPOSE: Although cancer pain is prevalent, under-treatment still remains a problem. Knowledge of and compliance with guidelines for management of cancer pain were analyzed for exploration of physician-related barriers to cancer pain management. In addition, physicians' knowledge and its correlation with cancer pain control were audited.MATERIALS AND METHODS: From July 8 to December 2, 2010, a nationwide survey of house staff enquired about their knowledge of cancer pain control guidelines, and the medical records of patients under their care were analyzed.RESULTS: In total, 180 physicians participated in the study. Their average score for knowledge was 14.6 (range, 7 to 19; maximum possible, 20). When the knowledge score was divided into low, medium, and high scores, patients receiving care from physicians with high levels of knowledge tended to have better cancer pain control (p<0.001). Of the total patients with severe pain, 19.5% were not prescribed strong opioids, and 40% were not prescribed any medication for breakthrough pain.CONCLUSION: Physicians' knowledge of guidelines for control of cancer pain showed an association with improvement of pain management. Overall adherence to the guidelines was lacking. Continuous interventions such as education and audits regarding cancer pain control guidelines for physician are needed.

Institutions:
(Kim DY) Department of Internal Medicine, Dongguk University Ilsan Hospital, Dongguk University College of Medicine, Goyang, Korea.; (Ahn JS) Department of Medicine, Samsung Medical Center, Sungkyunkwan University School of Medicine, Seoul, Korea.; (Lee KH) Department of Internal Medicine, Yeungnam University College of Medicine, Daegu, Korea.; (Kim YC) Department of Internal Medicine, Chonnam National University Hwasun Hospital, Chonnam National University Medical School, Hwasun, Korea.; (Lee J) Department of Biostatistics, Korea University College of Medicine, Seoul, Korea.; (Kim SY) Department of Internal Medicine, Kyung Hee University Hospital, Kyung Hee University School of Medicine, Seoul, Korea.
(Kim DY) Department of Internal Medicine, Dongguk University Ilsan Hospital, Dongguk University College of Medicine, Goyang, Korea.; (Ahn JS) Department of Medicine, Samsung Medical Center, Sungkyunkwan University School of Medicine, Seoul, Korea.; (Lee KH) Department of Internal Medicine, Yeungnam University College of Medicine, Daegu, Korea.; (Kim YC) Department of Internal Medicine, Chonnam National University Hwasun Hospital, Chonnam National University Medical School, Hwasun, Korea.; (Lee J) Department of Biostatistics, Korea University College of Medicine, Seoul, Korea.; (Kim SY) Department of Internal Medicine, Kyung Hee University Hospital, Kyung Hee University School of Medicine, Seoul, Korea.

(Kim DY) Department of Internal Medicine, Dongguk University Ilsan Hospital, Dongguk University College of Medicine, Goyang, Korea.; (Ahn JS) Department of Medicine, Samsung Medical Center, Sungkyunkwan University School of Medicine, Seoul, Korea.; (Lee KH) Department of Internal Medicine, Yeungnam University College of Medicine, Daegu, Korea.; (Kim YC) Department of Internal Medicine, Chonnam National University Hwasun Hospital, Chonnam National University Medical School, Hwasun, Korea.; (Lee J) Department of Biostatistics, Korea University College of Medicine, Seoul, Korea.; (Kim SY) Department of Internal Medicine, Kyung Hee University Hospital, Kyung Hee University School of Medicine, Seoul, Korea.
(Kim DY) Department of Internal Medicine, Dongguk University Ilsan Hospital, Dongguk University College of Medicine, Goyang, Korea.; (Ahn JS) Department of Medicine, Samsung Medical Center, Sungkyunkwan University School of Medicine, Seoul, Korea.; (Lee KH) Department of Internal Medicine, Yeungnam University College of Medicine, Daegu, Korea.; (Kim YC) Department of Internal Medicine, Chonnam National University Hwasun Hospital, Chonnam National University Medical School, Hwasun, Korea.; (Lee J) Department of Biostatistics, Korea University College of Medicine, Seoul, Korea.; (Kim SY) Department of Internal Medicine, Kyung Hee University Hospital, Kyung Hee University School of Medicine, Seoul, Korea.
(Kim DY) Department of Internal Medicine, Dongguk University Ilsan Hospital, Dongguk University College of Medicine, Goyang, Korea.; (Ahn JS) Department of Medicine, Samsung Medical Center, Sungkyunkwan University School of Medicine, Seoul, Korea.; (Lee KH) Department of Internal Medicine, Yeungnam University College of Medicine, Daegu, Korea.; (Kim YC) Department of Internal Medicine, Chonnam National University Hwasun Hospital, Chonnam National University Medical School, Hwasun, Korea.; (Lee J) Department of Biostatistics, Korea University College of Medicine, Seoul, Korea.; (Kim SY) Department of Internal Medicine, Kyung Hee University Hospital, Kyung Hee University School of Medicine, Seoul, Korea.
(Kim DY) Department of Internal Medicine, Dongguk University Ilsan Hospital, Dongguk University College of Medicine, Goyang, Korea.; (Ahn JS) Department of Medicine, Samsung Medical Center, Sungkyunkwan University School of Medicine, Seoul, Korea.; (Lee KH) Department of Internal Medicine, Yeungnam University College of Medicine, Daegu, Korea.; (Kim YC) Department of Internal Medicine, Chonnam National University Hwasun Hospital, Chonnam National University Medical School, Hwasun, Korea.; (Lee J) Department of Biostatistics, Korea University College of Medicine, Seoul, Korea.; (Kim SY) Department of Internal Medicine, Kyung Hee University Hospital, Kyung Hee University School of Medicine, Seoul, Korea.
Database: PubMed

128. Pain in the cancer patient: different pain characteristics CHANGE pharmacological treatment requirements.

Author(s): Müller-Schwefe G; Ahlbeck K; Aldington D; Alon E; Coaccioli S; Coluzzi F; Huygen F; Jaksch W; Kalso E; Kocot-Kępska M; Kress HG; Mangas AC; Ferri CM; Morlion B; Nicolaou A; Hernández CP; Pergolizzi J; Schäfer M; Sichère P

Source: Current medical research and opinion; Sep 2014; vol. 30 (no. 9); p. 1895-1908
Publication Date: Sep 2014
Publication Type(s): Journal Article; Research Support, Non-U.S. Gov't
DOI: http://dx.doi.org/10.1185/03007995.2014.925439
ISSN: 1473-4877
Place of Publication: England
PubMedID: 24841174
Accession Number: 24841174
Available at Current medical research and opinion - from Unpaywall
Keywords: Subject Terms: Breakthrough pain; Causative mechanisms; Chemotherapy-induced neuropathic pain; Fast-acting fentanyl formulations; Multidisciplinary approach; Opioids; Under-treatment
Abstract: Twenty years ago, the main barriers to successful cancer pain management were poor assessment by physicians, and patients' reluctance to report pain and take opioids. Those barriers are almost exactly the same today. Cancer pain remains under-treated; in Europe, almost three-quarters of cancer patients experience pain, and almost a quarter of those with moderate to severe pain do not receive any analgesic medication. Yet it has been suggested that pain management could be improved simply by ensuring that every consultation includes the patient's rating of pain, that the physician pays attention to this rating, and a plan is agreed to increase analgesia when it is inadequate. After outlining current concepts of carcinogenesis in some detail, this paper describes different methods of classifying and diagnosing cancer pain and the extent of current under-treatment. Key points are made regarding cancer pain management. Firstly, the pain may be caused by multiple different mechanisms and therapy should reflect those underlying mechanisms - rather than being simply based on pain intensity as recommended by the WHO three-step ladder. Secondly, a multidisciplinary approach is required which combines both pharmacological and non-pharmacological treatment, such as psychotherapy, exercise therapy and electrostimulation. The choice of analgesic agent and its route of administration are considered, along with various interventional procedures and the requirements of palliative care. Special attention is paid to the treatment of breakthrough pain (particularly with fast-acting fentanyl formulations, which have pharmacokinetic profiles that closely match those of breakthrough pain episodes) and chemotherapy-induced neuropathic pain, which affects around one third of patients who receive chemotherapy. Finally, the point is made that medical education should place a greater emphasis on pain therapy, both at undergraduate and postgraduate level.
Institutions:
(Müller-Schwefe G) Schmerz- und Palliativzentrum , Göppingen , Germany.
Database: PubMed

129. Patient education, coaching, and self-management for cancer pain.

Author(s): Lovell MR; Luckett T; Boyle FM; Phillips J; Agar M; Davidson PM

Source: Journal of clinical oncology : official journal of the American Society of Clinical Oncology; Jun 2014; vol. 32 (no. 16); p. 1712-1720

Publication Date: Jun 2014

Publication Type(s): Journal Article; Review

DOI: http://dx.doi.org/10.1200/JCO.2013.52.4850

ISSN: 1527-7755

Place of Publication: United States

PubMedID: 24799486

Accession Number: 24799486

Available at Journal of clinical oncology : official journal of the American Society of Clinical Oncology - from Unpaywall

Abstract:PURPOSE: Multiple systematic reviews and meta-analyses have identified the effectiveness of patient education in improving cancer pain management. However, the mechanisms by which patient education improves pain outcomes are uncertain, as are the optimal delivery, content, timing, frequency, and duration. This review provides best-bet recommendations based on available evidence to guide service managers and clinicians in developing a patient education program.METHODS: We used patient-centered care, self-management, coaching, and a behavior change wheel as lenses through which to consider the evidence for elements of patient education most likely to be effective within the context of other strategies for overcoming barriers to cancer pain assessment and management.RESULTS: The evidence suggests that optimal strategies include those that are patient-centered and tailored to individual needs, are embedded within health professional-patient communication and therapeutic relationships, empower patients to self-manage and coordinate their care, and are routinely integrated into standard cancer care. An approach that integrates patient education with processes and systems to ensure implementation of key standards for pain assessment and management and education of health professionals has been shown to be most effective.CONCLUSION: Patient education is effective in reducing cancer pain and should be standard practice in all settings. For optimal results, patient education should be integrated with other strategies for implementing evidence-based, person-centered care and overcoming barriers at the levels of patient, provider, and health system.

Institutions:
(Lovell MR) Melanie R. Lovell and Meera Agar, HammondCare; Melanie R. Lovell and Frances M. Boyle, University of Sydney; Melanie R. Lovell, Tim Luckett, Jane Phillips, Meera Agar, and Patricia M. Davidson, ImPaCCT (New South Wales Palliative Care Trials Group); Tim Luckett and Patricia M. Davidson, University of Technology Sydney; Jane Phillips, University of Notre Dame; and Meera Agar, University of New South Wales, Sydney, New South Wales, Australia. mlovell@hammond.com.au.; (Luckett T) Melanie R. Lovell and Meera Agar, HammondCare; Melanie R. Lovell and Frances M. Boyle, University of Sydney; Melanie R. Lovell, Tim Luckett, Jane Phillips, Meera Agar, and Patricia M. Davidson, ImPaCCT (New South Wales Palliative Care Trials Group); Tim Luckett and Patricia M. Davidson, University of Technology Sydney; Jane Phillips, University of Notre Dame; and Meera Agar, University of New South Wales, Sydney, New South Wales, Australia.; (Boyle FM) Melanie R. Lovell and Meera Agar, HammondCare; Melanie R.

Lovell and Frances M. Boyle, University of Sydney; Melanie R. Lovell, Tim Luckett, Jane Phillips, Meera Agar, and Patricia M. Davidson, ImPaCCT (New South Wales Palliative Care Trials Group); Tim Luckett and Patricia M. Davidson, University of Technology Sydney; Jane Phillips, University of Notre Dame; and Meera Agar, University of New South Wales, Sydney, New South Wales, Australia.; (Phillips J) Melanie R. Lovell and Meera Agar, HammondCare; Melanie R. Lovell and Frances M. Boyle, University of Sydney; Melanie R. Lovell, Tim Luckett, Jane Phillips, Meera Agar, and Patricia M. Davidson, ImPaCCT (New South Wales Palliative Care Trials Group); Tim Luckett and Patricia M. Davidson, University of Technology Sydney; Jane Phillips, University of Notre Dame; and Meera Agar, University of New South Wales, Sydney, New South Wales, Australia.; (Agar M) Melanie R. Lovell and Meera Agar, HammondCare; Melanie R. Lovell and Frances M. Boyle, University of Sydney; Melanie R. Lovell, Tim Luckett, Jane Phillips, Meera Agar, and Patricia M. Davidson, ImPaCCT (New South Wales Palliative Care Trials Group); Tim Luckett and Patricia M. Davidson, University of Technology Sydney; Jane Phillips, University of Notre Dame; and Meera Agar, University of New South Wales, Sydney, New South Wales, Australia.; (Davidson PM) Melanie R. Lovell and Meera Agar, HammondCare; Melanie R. Lovell and Frances M. Boyle, University of Sydney; Melanie R. Lovell, Tim Luckett, Jane Phillips, Meera Agar, and Patricia M. Davidson, ImPaCCT (New South Wales Palliative Care Trials Group); Tim Luckett and Patricia M. Davidson, University of Technology Sydney; Jane Phillips, University of Notre Dame; and Meera Agar, University of New South Wales, Sydney, New South Wales, Australia. (Lovell MR) Melanie R. Lovell and Meera Agar, HammondCare; Melanie R. Lovell and Frances M. Boyle, University of Sydney; Melanie R. Lovell, Tim Luckett, Jane Phillips, Meera Agar, and Patricia M. Davidson, ImPaCCT (New South Wales Palliative Care Trials Group); Tim Luckett and Patricia M. Davidson, University of Technology Sydney; Jane Phillips, University of Notre Dame; and Meera Agar, University of New South Wales, Sydney, New South Wales, Australia. mlovell@hammond.com.au.; (Luckett T) Melanie R. Lovell and Meera Agar, HammondCare; Melanie R. Lovell and Frances M. Boyle, University of Sydney; Melanie R. Lovell, Tim Luckett, Jane Phillips, Meera Agar, and Patricia M. Davidson, ImPaCCT (New South Wales Palliative Care Trials Group); Tim Luckett and Patricia M. Davidson, University of Technology Sydney; Jane Phillips, University of Notre Dame; and Meera Agar, University of New South Wales, Sydney, New South Wales, Australia.; (Boyle FM) Melanie R. Lovell and Meera Agar, HammondCare; Melanie R. Lovell and Frances M. Boyle, University of Sydney; Melanie R. Lovell, Tim Luckett, Jane Phillips, Meera Agar, and Patricia M. Davidson, ImPaCCT (New South Wales Palliative Care Trials Group); Tim Luckett and Patricia M. Davidson, University of Technology Sydney; Jane Phillips, University of Notre Dame; and Meera Agar, University of New South Wales, Sydney, New South Wales, Australia.; (Phillips J) Melanie R. Lovell and Meera Agar, HammondCare; Melanie R. Lovell and Frances M. Boyle, University of Sydney; Melanie R. Lovell, Tim Luckett, Jane Phillips, Meera Agar, and Patricia M. Davidson, ImPaCCT (New South Wales Palliative Care Trials Group); Tim Luckett and Patricia M. Davidson, University of Technology Sydney; Jane Phillips, University of Notre Dame; and Meera Agar, University of New South Wales, Sydney, New South Wales, Australia.; (Agar M) Melanie R. Lovell and Meera Agar, HammondCare; Melanie R. Lovell and Frances M. Boyle, University of Sydney; Melanie R. Lovell, Tim Luckett, Jane Phillips, Meera Agar, and Patricia M. Davidson, ImPaCCT (New South Wales Palliative Care Trials Group); Tim Luckett and Patricia M. Davidson, University of Technology Sydney; Jane Phillips, University of Notre

Dame; and Meera Agar, University of New South Wales, Sydney, New South Wales, Australia.; (Davidson PM) Melanie R. Lovell and Meera Agar, HammondCare; Melanie R. Lovell and Frances M. Boyle, University of Sydney; Melanie R. Lovell, Tim Luckett, Jane Phillips, Meera Agar, and Patricia M. Davidson, ImPaCCT (New South Wales Palliative Care Trials Group); Tim Luckett and Patricia M. Davidson, University of Technology Sydney; Jane Phillips, University of Notre Dame; and Meera Agar, University of New South Wales, Sydney, New South Wales, Australia. (Lovell MR) Melanie R. Lovell and Meera Agar, HammondCare; Melanie R. Lovell and Frances M. Boyle, University of Sydney; Melanie R. Lovell, Tim Luckett, Jane Phillips, Meera Agar, and Patricia M. Davidson, ImPaCCT (New South Wales Palliative Care Trials Group); Tim Luckett and Patricia M. Davidson, University of Technology Sydney; Jane Phillips, University of Notre Dame; and Meera Agar, University of New South Wales, Sydney, New South Wales, Australia. mlovell@hammond.com.au.; (Luckett T) Melanie R. Lovell and Meera Agar, HammondCare; Melanie R. Lovell and Frances M. Boyle, University of Sydney; Melanie R. Lovell, Tim Luckett, Jane Phillips, Meera Agar, and Patricia M. Davidson, ImPaCCT (New South Wales Palliative Care Trials Group); Tim Luckett and Patricia M. Davidson, University of Technology Sydney; Jane Phillips, University of Notre Dame; and Meera Agar, University of New South Wales, Sydney, New South Wales, Australia.; (Boyle FM) Melanie R. Lovell and Meera Agar, HammondCare; Melanie R. Lovell and Frances M. Boyle, University of Sydney; Melanie R. Lovell, Tim Luckett, Jane Phillips, Meera Agar, and Patricia M. Davidson, ImPaCCT (New South Wales Palliative Care Trials Group); Tim Luckett and Patricia M. Davidson, University of Technology Sydney; Jane Phillips, University of Notre Dame; and Meera Agar, University of New South Wales, Sydney, New South Wales, Australia.; (Phillips J) Melanie R. Lovell and Meera Agar, HammondCare; Melanie R. Lovell and Frances M. Boyle, University of Sydney; Melanie R. Lovell, Tim Luckett, Jane Phillips, Meera Agar, and Patricia M. Davidson, ImPaCCT (New South Wales Palliative Care Trials Group); Tim Luckett and Patricia M. Davidson, University of Technology Sydney; Jane Phillips, University of Notre Dame; and Meera Agar, University of New South Wales, Sydney, New South Wales, Australia.; (Agar M) Melanie R. Lovell and Meera Agar, HammondCare; Melanie R. Lovell and Frances M. Boyle, University of Sydney; Melanie R. Lovell, Tim Luckett, Jane Phillips, Meera Agar, and Patricia M. Davidson, ImPaCCT (New South Wales Palliative Care Trials Group); Tim Luckett and Patricia M. Davidson, University of Technology Sydney; Jane Phillips, University of Notre Dame; and Meera Agar, University of New South Wales, Sydney, New South Wales, Australia.; (Davidson PM) Melanie R. Lovell and Meera Agar, HammondCare; Melanie R. Lovell and Frances M. Boyle, University of Sydney; Melanie R. Lovell, Tim Luckett, Jane Phillips, Meera Agar, and Patricia M. Davidson, ImPaCCT (New South Wales Palliative Care Trials Group); Tim Luckett and Patricia M. Davidson, University of Technology Sydney; Jane Phillips, University of Notre Dame; and Meera Agar, University of New South Wales, Sydney, New South Wales, Australia. (Lovell MR) Melanie R. Lovell and Meera Agar, HammondCare; Melanie R. Lovell and Frances M. Boyle, University of Sydney; Melanie R. Lovell, Tim Luckett, Jane Phillips, Meera Agar, and Patricia M. Davidson, ImPaCCT (New South Wales Palliative Care Trials Group); Tim Luckett and Patricia M. Davidson, University of Technology Sydney; Jane Phillips, University of Notre Dame; and Meera Agar, University of New South Wales, Sydney, New South Wales, Australia. mlovell@hammond.com.au.; (Luckett T) Melanie R. Lovell and Meera Agar, HammondCare; Melanie R. Lovell and Frances M. Boyle, University of Sydney;

Melanie R. Lovell, Tim Luckett, Jane Phillips, Meera Agar, and Patricia M. Davidson, ImPaCCT (New South Wales Palliative Care Trials Group); Tim Luckett and Patricia M. Davidson, University of Technology Sydney; Jane Phillips, University of Notre Dame; and Meera Agar, University of New South Wales, Sydney, New South Wales, Australia.; (Boyle FM) Melanie R. Lovell and Meera Agar, HammondCare; Melanie R. Lovell and Frances M. Boyle, University of Sydney; Melanie R. Lovell, Tim Luckett, Jane Phillips, Meera Agar, and Patricia M. Davidson, ImPaCCT (New South Wales Palliative Care Trials Group); Tim Luckett and Patricia M. Davidson, University of Technology Sydney; Jane Phillips, University of Notre Dame; and Meera Agar, University of New South Wales, Sydney, New South Wales, Australia.; (Phillips J) Melanie R. Lovell and Meera Agar, HammondCare; Melanie R. Lovell and Frances M. Boyle, University of Sydney; Melanie R. Lovell, Tim Luckett, Jane Phillips, Meera Agar, and Patricia M. Davidson, ImPaCCT (New South Wales Palliative Care Trials Group); Tim Luckett and Patricia M. Davidson, University of Technology Sydney; Jane Phillips, University of Notre Dame; and Meera Agar, University of New South Wales, Sydney, New South Wales, Australia.; (Agar M) Melanie R. Lovell and Meera Agar, HammondCare; Melanie R. Lovell and Frances M. Boyle, University of Sydney; Melanie R. Lovell, Tim Luckett, Jane Phillips, Meera Agar, and Patricia M. Davidson, ImPaCCT (New South Wales Palliative Care Trials Group); Tim Luckett and Patricia M. Davidson, University of Technology Sydney; Jane Phillips, University of Notre Dame; and Meera Agar, University of New South Wales, Sydney, New South Wales, Australia.; (Davidson PM) Melanie R. Lovell and Meera Agar, HammondCare; Melanie R. Lovell and Frances M. Boyle, University of Sydney; Melanie R. Lovell, Tim Luckett, Jane Phillips, Meera Agar, and Patricia M. Davidson, ImPaCCT (New South Wales Palliative Care Trials Group); Tim Luckett and Patricia M. Davidson, University of Technology Sydney; Jane Phillips, University of Notre Dame; and Meera Agar, University of New South Wales, Sydney, New South Wales, Australia. (Lovell MR) Melanie R. Lovell and Meera Agar, HammondCare; Melanie R. Lovell and Frances M. Boyle, University of Sydney; Melanie R. Lovell, Tim Luckett, Jane Phillips, Meera Agar, and Patricia M. Davidson, ImPaCCT (New South Wales Palliative Care Trials Group); Tim Luckett and Patricia M. Davidson, University of Technology Sydney; Jane Phillips, University of Notre Dame; and Meera Agar, University of New South Wales, Sydney, New South Wales, Australia. mlovell@hammond.com.au.; (Luckett T) Melanie R. Lovell and Meera Agar, HammondCare; Melanie R. Lovell and Frances M. Boyle, University of Sydney; Melanie R. Lovell, Tim Luckett, Jane Phillips, Meera Agar, and Patricia M. Davidson, ImPaCCT (New South Wales Palliative Care Trials Group); Tim Luckett and Patricia M. Davidson, University of Technology Sydney; Jane Phillips, University of Notre Dame; and Meera Agar, University of New South Wales, Sydney, New South Wales, Australia.; (Boyle FM) Melanie R. Lovell and Meera Agar, HammondCare; Melanie R. Lovell and Frances M. Boyle, University of Sydney; Melanie R. Lovell, Tim Luckett, Jane Phillips, Meera Agar, and Patricia M. Davidson, ImPaCCT (New South Wales Palliative Care Trials Group); Tim Luckett and Patricia M. Davidson, University of Technology Sydney; Jane Phillips, University of Notre Dame; and Meera Agar, University of New South Wales, Sydney, New South Wales, Australia.; (Phillips J) Melanie R. Lovell and Meera Agar, HammondCare; Melanie R. Lovell and Frances M. Boyle, University of Sydney; Melanie R. Lovell, Tim Luckett, Jane Phillips, Meera Agar, and Patricia M. Davidson, ImPaCCT (New South Wales Palliative Care Trials Group); Tim Luckett and Patricia M. Davidson, University of Technology Sydney; Jane Phillips, University of Notre Dame; and Meera Agar, University of New South

Wales, Sydney, New South Wales, Australia.; (Agar M) Melanie R. Lovell and Meera Agar, HammondCare; Melanie R. Lovell and Frances M. Boyle, University of Sydney; Melanie R. Lovell, Tim Luckett, Jane Phillips, Meera Agar, and Patricia M. Davidson, ImPaCCT (New South Wales Palliative Care Trials Group); Tim Luckett and Patricia M. Davidson, University of Technology Sydney; Jane Phillips, University of Notre Dame; and Meera Agar, University of New South Wales, Sydney, New South Wales, Australia.; (Davidson PM) Melanie R. Lovell and Meera Agar, HammondCare; Melanie R. Lovell and Frances M. Boyle, University of Sydney; Melanie R. Lovell, Tim Luckett, Jane Phillips, Meera Agar, and Patricia M. Davidson, ImPaCCT (New South Wales Palliative Care Trials Group); Tim Luckett and Patricia M. Davidson, University of Technology Sydney; Jane Phillips, University of Notre Dame; and Meera Agar, University of New South Wales, Sydney, New South Wales, Australia. (Lovell MR) Melanie R. Lovell and Meera Agar, HammondCare; Melanie R. Lovell and Frances M. Boyle, University of Sydney; Melanie R. Lovell, Tim Luckett, Jane Phillips, Meera Agar, and Patricia M. Davidson, ImPaCCT (New South Wales Palliative Care Trials Group); Tim Luckett and Patricia M. Davidson, University of Technology Sydney; Jane Phillips, University of Notre Dame; and Meera Agar, University of New South Wales, Sydney, New South Wales, Australia. mlovell@hammond.com.au.; (Luckett T) Melanie R. Lovell and Meera Agar, HammondCare; Melanie R. Lovell and Frances M. Boyle, University of Sydney; Melanie R. Lovell, Tim Luckett, Jane Phillips, Meera Agar, and Patricia M. Davidson, ImPaCCT (New South Wales Palliative Care Trials Group); Tim Luckett and Patricia M. Davidson, University of Technology Sydney; Jane Phillips, University of Notre Dame; and Meera Agar, University of New South Wales, Sydney, New South Wales, Australia.; (Boyle FM) Melanie R. Lovell and Meera Agar, HammondCare; Melanie R. Lovell and Frances M. Boyle, University of Sydney; Melanie R. Lovell, Tim Luckett, Jane Phillips, Meera Agar, and Patricia M. Davidson, ImPaCCT (New South Wales Palliative Care Trials Group); Tim Luckett and Patricia M. Davidson, University of Technology Sydney; Jane Phillips, University of Notre Dame; and Meera Agar, University of New South Wales, Sydney, New South Wales, Australia.; (Phillips J) Melanie R. Lovell and Meera Agar, HammondCare; Melanie R. Lovell and Frances M. Boyle, University of Sydney; Melanie R. Lovell, Tim Luckett, Jane Phillips, Meera Agar, and Patricia M. Davidson, ImPaCCT (New South Wales Palliative Care Trials Group); Tim Luckett and Patricia M. Davidson, University of Technology Sydney; Jane Phillips, University of Notre Dame; and Meera Agar, University of New South Wales, Sydney, New South Wales, Australia.; (Agar M) Melanie R. Lovell and Meera Agar, HammondCare; Melanie R. Lovell and Frances M. Boyle, University of Sydney; Melanie R. Lovell, Tim Luckett, Jane Phillips, Meera Agar, and Patricia M. Davidson, ImPaCCT (New South Wales Palliative Care Trials Group); Tim Luckett and Patricia M. Davidson, University of Technology Sydney; Jane Phillips, University of Notre Dame; and Meera Agar, University of New South Wales, Sydney, New South Wales, Australia.; (Davidson PM) Melanie R. Lovell and Meera Agar, HammondCare; Melanie R. Lovell and Frances M. Boyle, University of Sydney; Melanie R. Lovell, Tim Luckett, Jane Phillips, Meera Agar, and Patricia M. Davidson, ImPaCCT (New South Wales Palliative Care Trials Group); Tim Luckett and Patricia M. Davidson, University of Technology Sydney; Jane Phillips, University of Notre Dame; and Meera Agar, University of New South Wales, Sydney, New South Wales, Australia.
Database: PubMed

130. A survey on doctors' knowledge and attitude of treating chronic pain in three tertiary hospitals in Nigeria.

Author(s): Sanya EO; Kolo PM; Makusidi MA

Source: Nigerian medical journal : journal of the Nigeria Medical Association; Mar 2014; vol. 55 (no. 2); p. 106-110

Publication Date: Mar 2014

Publication Type(s): Journal Article

DOI: http://dx.doi.org/10.4103/0300-1652.129635

ISSN: 0300-1652

Place of Publication: Nigeria

PubMedID: 24791041

Accession Number: 24791041

Available at Nigerian medical journal : journal of the Nigeria Medical Association - from Europe PubMed Central - Open Access

Available at Nigerian medical journal : journal of the Nigeria Medical Association - from Unpaywall

Keywords: Subject Terms: Attitude; chronic pain; doctors; knowledge; treatment

Abstract:BACKGROUND: Chronic non-cancer pain (CP) is one of the most common complaints that bring patients to the hospital. When pain persists, people move from doctor-to-doctor seeking for help, thus the burden of CP is huge. This study, therefore was aimed at assessing attitude and knowledge of doctors in three teaching hospitals in Nigeria to CP.MATERIALS AND METHODS: Structured questionnaire was administered to doctors practicing at the University of Ilorin Teaching Hospital, Usmanu Danfodio University Teaching Hospital and University of Maiduguri Teaching Hospital. Responses were graded on maximum scale of five.RESULTS: Of the 410 doctors who participated in study, 79.7% were men. Their years of practice varied from 1 year to 20 years (mean SD = 4.5 ± 1.7 years). Close to 58% of participants were resident doctors, 36.4% medical officers and 8.6% consultants. Only 23.3% of participants had basic medical or postgraduate training on pain management. The physicians' mean goal of treating CP in patients was 3.7 ± 1.1, compared to 4.0 ± 1.1 in close relative and 4.1 ± 0.9 for doctors'-self pain. Only 9.5% of doctors use opioids for CP compared to 73% who use Nonsteroidal anti-inflammatory drugs (NSAIDs). Few doctors (23%) use ≥2 drugs to treat CP. Doctors were indifferent on the appropriateness of patients with CP to request for additional analgesics (mean score = 3.1 + 1.4). Doctors' self-rated knowledge of CP was 1.8 ± 0.7 compared to 4.1 ± 0.9 for acute and 0.8 ± 0.3 for cancer pains (P = 0. 003).CONCLUSION: Incorporation of pain management into continuing medical education could help improve observed deficiency in doctors' knowledge of pain treatment which resulted from lack of basic medical education on pain.

Institutions:
(Sanya EO) Department of Medicine, University of Ilorin Teaching Hospital, Ilorin, Kwara State, Nigeria.; (Kolo PM) Department of Medicine, University of Ilorin Teaching Hospital, Ilorin, Kwara State, Nigeria.; (Makusidi MA) Department of Medicine, Usmanu Dan Fodio University Teaching Hospital, Sokoto, Sokoto State, Nigeria.

(Sanya EO) Department of Medicine, University of Ilorin Teaching Hospital, Ilorin, Kwara State, Nigeria.; (Kolo PM) Department of Medicine, University of Ilorin Teaching Hospital, Ilorin, Kwara State, Nigeria.; (Makusidi MA) Department of Medicine, Usmanu Dan Fodio University Teaching Hospital, Sokoto, Sokoto State, Nigeria.
(Sanya EO) Department of Medicine, University of Ilorin Teaching Hospital, Ilorin, Kwara State, Nigeria.; (Kolo PM) Department of Medicine, University of Ilorin Teaching Hospital, Ilorin, Kwara State, Nigeria.; (Makusidi MA) Department of Medicine, Usmanu Dan Fodio University Teaching Hospital, Sokoto, Sokoto State, Nigeria.
Database: PubMed

131. An ethnographic study of barriers to cancer pain management and opioid availability in India.

Author(s): Lebaron V; Beck SL; Maurer M; Black F; Palat G

Source: The oncologist; May 2014; vol. 19 (no. 5); p. 515-522

Publication Date: May 2014

Publication Type(s): Journal Article

DOI: http://dx.doi.org/10.1634/theoncologist.2013-0435

ISSN: 1549-490X

Place of Publication: United States

PubMedID: 24755460

Accession Number: 24755460

Available at The oncologist - from Europe PubMed Central - Open Access

Available at The oncologist - from HighWire - Free Full Text

Available at The oncologist - from EBSCO (MEDLINE Complete)

Available at The oncologist - from Unpaywall

Keywords: Subject Terms: Cancer; Ethnography; India; Morphine; Nursing; Opioids; Pain management; Palliative care; World health

Abstract: The world's global cancer burden disproportionally affects lower income countries, where 80% of patients present with late-stage disease and have limited access to palliative care and effective pain-relieving medications, such as morphine. Consequently, millions die each year with unrelieved pain. Objective. The objective of this study was to examine barriers to opioid availability and cancer pain management in India, with an emphasis on the experiences of nurses, who are often the front-line providers of palliative care. Methods. Fifty-nine participants were recruited using a purposive, snowball sampling strategy. Ethnographic data collection included in-depth, semistructured interviews (n = 54), 400+ hours of participant observation, and review of documents over 9 months at a government cancer hospital in South India. Systematic qualitative analysis led to identification of key barriers that are exemplified by representative quotes. Results. Morphine is more available at this study site than in most of India, but access is limited to patients seen by the palliative care service, and significant gaps in supply still occur. Systems to measure and improve pain outcomes are largely absent. Key barriers related to pain management include the role of nursing, opioid misperceptions,

bureaucratic hurdles, and sociocultural/infrastructure challenges. Implications. Interventions must streamline process details of morphine procurement, work within the existing sociocultural infrastructure to ensure opioids reach patients most in need, target unexpected audiences for symptom management education, and account for role expectations of health care providers. Conclusion. Macro- and micro-level policy and practice changes are needed to improve opioid availability and cancer pain management in India.

Institutions:
(Lebaron V) Dana-Farber Cancer Institute/Harvard Global Equity Initiative/University of Massachusetts, Boston, Massachusetts, USA; University of Utah College of Nursing, Salt Lake City, Utah, USA; Pain and Policy Studies Group, University of Wisconsin Carbone Cancer Center, Madison, Wisconsin, USA; Victoria Hospice, Victoria, British Columbia, Canada; Two Worlds Cancer Collaboration Foundation, Canada, International Network for Cancer Treatment and Research, India.
Database: PubMed

132. A systematic review: non-pharmacological interventions in treating pain in patients with advanced cancer.

Author(s): Hökkä M; Kaakinen P; Pölkki T

Source: Journal of advanced nursing; Sep 2014; vol. 70 (no. 9); p. 1954-1969

Publication Date: Sep 2014

Publication Type(s): Journal Article; Review; Systematic Review

DOI: http://dx.doi.org/10.1111/jan.12424

ISSN: 1365-2648

Place of Publication: England

PubMedID: 24730753

Accession Number: 24730753

Available at Journal of advanced nursing - from Wiley Online Library Medicine and Nursing Collection 2020

Keywords: Subject Terms: adult care; cancer; complementary therapy; holistic care; literature review; non-pharmacological intervention; nursing; pain; palliative care

Abstract:AIMS: To assess and synthesize the evidence of the effects and safety of non-pharmacological interventions in treating pain in patients with advanced cancer.BACKGROUND: Pain is a common symptom experienced by patients with advanced cancer; the treatment of such pain is often suboptimal. To manage it, non-pharmacological interventions are recommended after pharmacological treatments have been re-evaluated and modified. However, there remains a lack of knowledge about the effects and safety of such interventions.DESIGN: A systematic review was conducted based on the procedure of the Centre of Reviews and Dissemination.DATA SOURCES: Research papers published between 2000-2013 were identified from the following databases: CINAHL, MEDIC, MEDLINE (Ovid) and PsycINFO. The references in the selected studies were searched manually.REVIEW METHODS: The studies selected were reviewed for quality, using Cochrane Effective Practice and Organisation of Care Review Group risk of bias assessment

criteria.RESULTS: There was limited evidence that some of the non-pharmacological interventions were promising with respect to reducing cancer pain. Relatively, few adverse events were reported as a result of using such interventions.CONCLUSION: It was not possible to draw conclusions about the effects and safety of the non-pharmacological interventions in reducing cancer pain. Some interventions showed promising short-term effects, but there is a need for more rigorous trials. Qualitative studies are required to collect information about patients' perceptions. There are several research gaps: we found no studies about music, spiritual care, hypnosis, active coping training, cold or ultrasonic stimulation.

Institutions:
(Hökkä M) Institution of Health Sciences, University of Oulu, Finland.; (Kaakinen P) Institution of Health Sciences, University of Oulu, University Hospital of Oulu, Finland.; (Pölkki T) Institution of Health Sciences, University of Oulu, Finland.
(Hökkä M) Institution of Health Sciences, University of Oulu, Finland.; (Kaakinen P) Institution of Health Sciences, University of Oulu, University Hospital of Oulu, Finland.; (Pölkki T) Institution of Health Sciences, University of Oulu, Finland.
(Hökkä M) Institution of Health Sciences, University of Oulu, Finland.; (Kaakinen P) Institution of Health Sciences, University of Oulu, University Hospital of Oulu, Finland.; (Pölkki T) Institution of Health Sciences, University of Oulu, Finland.
Database: PubMed

133. Pain medication management processes used by oncology outpatients and family caregivers part I: health systems contexts.

Author(s): Schumacher KL; Plano Clark VL; West CM; Dodd MJ; Rabow MW; Miaskowski C

Source: Journal of pain and symptom management; Nov 2014; vol. 48 (no. 5); p. 770-783

Publication Date: Nov 2014

Publication Type(s): Journal Article; Randomized Controlled Trial; Research Support, N.I.H., Extramural

DOI: http://dx.doi.org/10.1016/j.jpainsymman.2013.12.242

ISSN: 1873-6513

Place of Publication: United States

PubMedID: 24704800

Accession Number: 24704800

Available at Journal of pain and symptom management - from ScienceDirect

Available at Journal of pain and symptom management - from David Adams Library Journals Collection Local Print Collection [location] : David Adams Library.

Available at Journal of pain and symptom management - from Unpaywall

Keywords: Subject Terms: Cancer pain management; analgesics; family caregivers; health systems; medication management; medication safety; qualitative research; randomized clinical trial; self-care; self-management

Abstract:CONTEXT: Oncology patients with persistent pain treated in outpatient settings and their family caregivers have significant responsibility for managing pain medications. However, little is known about their practical day-to-day experiences

with pain medication management.OBJECTIVES: The aim was to describe day-to-day pain medication management from the perspectives of oncology outpatients and their family caregivers who participated in a randomized clinical trial of a psychoeducational intervention called the Pro-Self(©) Plus Pain Control Program. In this article, we focus on pain medication management by patients and family caregivers in the context of multiple complex health systems.METHODS: We qualitatively analyzed audio-recorded intervention sessions that included extensive dialogue between patients, family caregivers, and nurses about pain medication management during the 10-week intervention.RESULTS: The health systems context for pain medication management included multiple complex systems for clinical care, reimbursement, and regulation of analgesic prescriptions. Pain medication management processes particularly relevant to this context were getting prescriptions and obtaining medications. Responsibilities that fell primarily to patients and family caregivers included facilitating communication and coordination among multiple clinicians, overcoming barriers to access, and serving as a final safety checkpoint. Significant effort was required of patients and family caregivers to insure safe and effective pain medication management.CONCLUSION: Health systems issues related to access to needed analgesics, medication safety in outpatient settings, and the effort expended by oncology patients and their family caregivers require more attention in future research and health-care reform initiatives.

Institutions:

(Schumacher KL) College of Nursing, University of Nebraska Medical Center, Omaha, Nebraska, USA. Electronic address: kschumacher@unmc.edu.; (Plano Clark VL) University of Cincinnati, Cincinnati, Ohio, USA.; (West CM) University of California, San Francisco, San Francisco, California, USA.; (Dodd MJ) University of California, San Francisco, San Francisco, California, USA.; (Rabow MW) University of California, San Francisco, San Francisco, California, USA.; (Miaskowski C) University of California, San Francisco, San Francisco, California, USA.
(Schumacher KL) College of Nursing, University of Nebraska Medical Center, Omaha, Nebraska, USA. Electronic address: kschumacher@unmc.edu.; (Plano Clark VL) University of Cincinnati, Cincinnati, Ohio, USA.; (West CM) University of California, San Francisco, San Francisco, California, USA.; (Dodd MJ) University of California, San Francisco, San Francisco, California, USA.; (Rabow MW) University of California, San Francisco, San Francisco, California, USA.; (Miaskowski C) University of California, San Francisco, San Francisco, California, USA.
(Schumacher KL) College of Nursing, University of Nebraska Medical Center, Omaha, Nebraska, USA. Electronic address: kschumacher@unmc.edu.; (Plano Clark VL) University of Cincinnati, Cincinnati, Ohio, USA.; (West CM) University of California, San Francisco, San Francisco, California, USA.; (Dodd MJ) University of California, San Francisco, San Francisco, California, USA.; (Rabow MW) University of California, San Francisco, San Francisco, California, USA.; (Miaskowski C) University of California, San Francisco, San Francisco, California, USA.
(Schumacher KL) College of Nursing, University of Nebraska Medical Center, Omaha, Nebraska, USA. Electronic address: kschumacher@unmc.edu.; (Plano Clark VL) University of Cincinnati, Cincinnati, Ohio, USA.; (West CM) University of California, San Francisco, San Francisco, California, USA.; (Dodd MJ) University of California, San Francisco, San Francisco, California, USA.; (Rabow MW) University of California, San Francisco, San Francisco, California, USA.; (Miaskowski C) University of California, San Francisco, San Francisco, California, USA.

(Schumacher KL) College of Nursing, University of Nebraska Medical Center, Omaha, Nebraska, USA. Electronic address: kschumacher@unmc.edu.; (Plano Clark VL) University of Cincinnati, Cincinnati, Ohio, USA.; (West CM) University of California, San Francisco, San Francisco, California, USA.; (Dodd MJ) University of California, San Francisco, San Francisco, California, USA.; (Rabow MW) University of California, San Francisco, San Francisco, California, USA.; (Miaskowski C) University of California, San Francisco, San Francisco, California, USA.
(Schumacher KL) College of Nursing, University of Nebraska Medical Center, Omaha, Nebraska, USA. Electronic address: kschumacher@unmc.edu.; (Plano Clark VL) University of Cincinnati, Cincinnati, Ohio, USA.; (West CM) University of California, San Francisco, San Francisco, California, USA.; (Dodd MJ) University of California, San Francisco, San Francisco, California, USA.; (Rabow MW) University of California, San Francisco, San Francisco, California, USA.; (Miaskowski C) University of California, San Francisco, San Francisco, California, USA.
Database: PubMed

134. "We all talk about it as though we're thinking about the same thing." Healthcare professionals' goals in the management of pain due to advanced cancer: a qualitative study.

Author(s): Bhatia R; Rebecca B; Gibbins J; Jane G; Forbes K; Karen F; Reid C; Colette R

Source: Supportive care in cancer : official journal of the Multinational Association of Supportive Care in Cancer; Aug 2014; vol. 22 (no. 8); p. 2067-2073

Publication Date: Aug 2014

Publication Type(s): Journal Article; Research Support, Non-U.S. Gov't

DOI: http://dx.doi.org/10.1007/s00520-014-2191-6

ISSN: 1433-7339

Place of Publication: Germany

PubMedID: 24633591

Accession Number: 24633591

Available at Supportive Care in Cancer - from SpringerLink

Available at Supportive Care in Cancer - from EBSCO (MEDLINE Complete)

Available at Supportive Care in Cancer - from EBSCO (CINAHL Complete)

Available at Supportive Care in Cancer - from ProQuest (MEDLINE with Full Text) - NHS Version

Available at Supportive Care in Cancer - from ProQuest (Health Research Premium) - NHS Version

Available at Supportive Care in Cancer - from Unpaywall

Abstract:PURPOSE: Unfortunately, several barriers impede successful management of cancer pain including those relating to the assessment and measurement of pain. There is currently no consensus as to what constitutes good pain control or what healthcare professionals are aiming to achieve in the management of pain for patients with advanced cancer. This study aimed to explore healthcare professionals' views and experiences to elicit what they are aiming to

achieve in managing pain for patients with advanced cancer.METHODS: Healthcare professionals involved in the management of cancer pain were sampled purposively and interviewed using a semi-structured interview technique until saturation of data. Data were analysed using the constant comparison approach.RESULTS: Sixteen interviews took place and four main themes emerged: aims of pain management, assessing response to pain management, managing expectations, and building relationships. Healthcare professionals found assessing patients' pain challenging and reported that patients had difficulty using numerical rating scales. Healthcare professionals used different terms when talking about managing pain, such as 'pain control' but found it difficult to define these terms. Maintaining patients' function and managing their expectations were described as important. However, it was not always clear whether the patient goals mentioned were voiced explicitly by the patient or assumed by the healthcare professional.CONCLUSION: Healthcare professionals described what they deemed important in the management of pain. The goals they mentioned almost exclusively related to function as opposed to pain scores, but patients' goals and expectations were often not elicited specifically.

Institutions:
(Bhatia R) Department of Palliative Medicine, Elgar House, Southmead Hospital, Bristol, BS10 5NB, UK, rebeccabhatia@hotmail.com.

Database: PubMed

135. Improvement of pain-related self-management for cancer patients through a modular transitional nursing intervention: a cluster-randomized multicenter trial.

Author(s): Jahn P; Kuss O; Schmidt H; Bauer A; Kitzmantel M; Jordan K; Krasemann S; Landenberger M

Source: Pain; Apr 2014; vol. 155 (no. 4); p. 746-754

Publication Date: Apr 2014

Publication Type(s): Journal Article; Multicenter Study; Randomized Controlled Trial; Research Support, Non-U.S. Gov't

DOI: http://dx.doi.org/10.1016/j.pain.2014.01.006

ISSN: 1872-6623

Place of Publication: United States

PubMedID: 24434732

Accession Number: 24434732

Available at Pain - from Ovid (Journals @ Ovid)

Available at Pain - from Ovid (Journals @ Ovid) - London Health Libraries

Keywords: Subject Terms: Cancer pain; Care transition; Patient education; Patient-related barriers; Self-management

Abstract: Patients' self-management skills are affected by their knowledge, activities, and attitudes toward pain management. This trial aimed to test the Self Care Improvement through Oncology Nursing (SCION)-PAIN program, a multimodular structured intervention to reduce patients' barriers to self-management of cancer pain. Two hundred sixty-three patients with diagnosed malignancy, pain>3 days, and average pain > or = 3/10 participated in a cluster-randomized trial on 18 wards in 2

German university hospitals. Patients on the intervention wards received, in addition to standard pain treatment, the SCION-PAIN program consisting of 3 modules: pharmacologic, nonpharmacologic pain management, and discharge management. The intervention was conducted by specially trained cancer nurses and included components of patient education, skills training, and counseling. Starting with admission, patients received booster sessions every third day and one follow-up telephone counseling session within 2 to 3 days after discharge. Patients in the control group received standard care. Primary end point was the group difference in patient-related barriers to self-management of cancer pain (Barriers Questionnaire-BQ II) 7 days after discharge. The SCION-PAIN program resulted in a significant reduction of patient-related barriers to pain management 1 week after discharge from the hospital: mean difference on BQ II was -0.49 points (95% confidence interval -0.87 points to -0.12 points; P=0.02). Furthermore, patients showed improved adherence to pain medication; odds ratio 8.58 (95% confidence interval 1.66-44.40; P=0.02). A post hoc analysis indicated reduced average and worst pain intensity as well as improved quality of life. This trial reveals the positive impact of a nursing intervention to improve patients' self-management of cancer pain.

Institutions:
(Jahn P) University Hospital Halle, Martin-Luther-University Halle-Wittenberg, Halle, Germany Institute for Biometry and Epidemiology, German Diabetes Center, Leibniz Institute for Diabetes Research at Heinrich Heine University Düsseldorf, Düsseldorf, Germany Institute for Health and Nursing Science, Medical Faculty, Martin-Luther-University Halle-Wittenberg, Halle, Germany University Hospital rechts der Isar, Technical University Munich, Munich, Germany.
Database: PubMed

136. Regional medical professionals' confidence in providing palliative care, associated difficulties and availability of specialized palliative care services in Japan.

Author(s): Hirooka K; Miyashita M; Morita T; Ichikawa T; Yoshida S; Akizuki N; Akiyama M; Shirahige Y; Eguchi K

Source: Japanese journal of clinical oncology; Mar 2014; vol. 44 (no. 3); p. 249-256

Publication Date: Mar 2014

Publication Type(s): Journal Article; Research Support, Non-U.S. Gov't

DOI: http://dx.doi.org/10.1093/jjco/hyt204

ISSN: 1465-3621

Place of Publication: England

PubMedID: 24407834

Accession Number: 24407834

Available at Japanese journal of clinical oncology - from HighWire - Free Full Text

Available at Japanese journal of clinical oncology - from Unpaywall

Keywords: Subject Terms: confidence; difficulties; medical professionals; palliative care

Abstract:BACKGROUND: Although confidence in providing palliative care services is an essential component of providing such care, factors relating to this have not

been investigated in Japan.OBJECTIVE: This study aimed to explore confidence in the ability to provide palliative care and associated difficulties and to explore correlations between these variables. Design A cross-sectional mail survey of medical doctors and registered nurses in Japan was performed as part of a regional intervention trial: the Outreach Palliative Care Trial of Integrated Regional Model study. Subjects Questionnaires were sent to 7905 medical professionals, and 409 hospital doctors, 235 general practitioners, 2160 hospital nurses and 115 home visiting nurses completed them.RESULTS: Confidence in providing palliative care was low and difficulties frequent for all types of medical professionals assessed. In particular, only 8-24% of them, depending on category, agreed to 'having adequate knowledge and skills regarding cancer pain management'. In particular, 55-80% of medical professionals acknowledged difficulty with 'alleviation of cancer pain'. Multiple regression analysis revealed that confidence was positively correlated with the amount of relevant experience and, for medical doctors, with 'prescriptions of opioids (per year)'. Moreover, difficulties were negatively correlated with the amount of relevant clinical experience.CONCLUSIONS: Effective strategies for developing regional palliative care programs include basic education of medical professionals on management of cancer-related pain (especially regarding opioids) and other symptoms.

Institutions:
(Hirooka K) *Department of System Management in Nursing, Graduate School of Heath Care Sciences, Tokyo Medical and Dental University, 1-5-45 Yushima, Bunkyo-ku, Tokyo 113-0034, Japan. hirkanr@tmd.ac.jp.
Database: PubMed

137. European Oncology Nursing Society breakthrough cancer pain guidelines.

Author(s): Wengström Y; Geerling J; Rustøen T

Source: European journal of oncology nursing : the official journal of European Oncology Nursing Society; Apr 2014; vol. 18 (no. 2); p. 127-131

Publication Date: Apr 2014

Publication Type(s): Journal Article; Review

DOI: http://dx.doi.org/10.1016/j.ejon.2013.11.009

ISSN: 1532-2122

Place of Publication: Scotland

PubMedID: 24369817

Accession Number: 24369817

Available at European journal of oncology nursing : the official journal of European Oncology Nursing Society - from ScienceDirect

Keywords: Subject Terms: Assessment; Breakthrough cancer pain; Evidence-based; Guideline; Management

Abstract:PURPOSE: The overall aim of the project was to update and inform nurses of current best practice based on previously published literature to enable nurses to assess and manage breakthrough cancer pain (BTCP) and thereby to provide optimal management of BTCP.METHODS: The EONS started a project in 2010 by

recruiting a working group and a multidisciplinary advisory board to develop guidelines with the purpose of helping oncology nurses understand and recognise BTCP.RESULTS: This paper presents and overview of the guideline. Key recommendations include; using an algorithm for assessment of BTCP, individualise treatment interventions, optimization of analgesia and reassessment of outcomes of interventions.CONCLUSIONS: By implementing the EONS guidelines nurses will utilise the latest available knowledge in clinical practice and the understanding and management of BTCP will improve assessment and overall management of breakthrough pain in cancer patients.

Institutions:
(Wengström Y) Karolinska Institutet, Department of Neurobiology, Care Science and Society, Division of Nursing, Huddinge, Sweden. Electronic address: Yvonne.wengstrom@ki.se.; (Geerling J) University Medical Centre Groningen, The Netherlands.; (Rustøen T) Division of Emergencies and Critical Care, Department of Research and Development, Oslo University Hospital, Ullevål, Norway; Lovisenberg Diaconal University College, Oslo, Norway.
(Wengström Y) Karolinska Institutet, Department of Neurobiology, Care Science and Society, Division of Nursing, Huddinge, Sweden. Electronic address: Yvonne.wengstrom@ki.se.; (Geerling J) University Medical Centre Groningen, The Netherlands.; (Rustøen T) Division of Emergencies and Critical Care, Department of Research and Development, Oslo University Hospital, Ullevål, Norway; Lovisenberg Diaconal University College, Oslo, Norway.
(Wengström Y) Karolinska Institutet, Department of Neurobiology, Care Science and Society, Division of Nursing, Huddinge, Sweden. Electronic address: Yvonne.wengstrom@ki.se.; (Geerling J) University Medical Centre Groningen, The Netherlands.; (Rustøen T) Division of Emergencies and Critical Care, Department of Research and Development, Oslo University Hospital, Ullevål, Norway; Lovisenberg Diaconal University College, Oslo, Norway.
Database: PubMed

138. Challenges of conducting experimental studies within a clinical nursing context.

Author(s): Gustafsson M; Bohman DM; Borglin G

Source: Applied nursing research : ANR; May 2014; vol. 27 (no. 2); p. 133-136

Publication Date: May 2014

Publication Type(s): Journal Article

DOI: http://dx.doi.org/10.1016/j.apnr.2013.11.013

ISSN: 1532-8201

Place of Publication: United States

PubMedID: 24355415

Accession Number: 24355415

Available at Applied nursing research : ANR - from EBSCO (CINAHL Complete)

Keywords: Subject Terms: Evidence-based nursing; Intervention studies; Nursing research; Quasi-experimental design

Abstract: In recent years, several distinguished scholars have advocated for nursing research that may carry strong evidence for practice. Their advocacy have highlighted that nursing science has reached a point where as nurse researchers we need to develop the questions we ask and design studies that have the power to produce solid, translational, evidence-based knowledge. To do so, we need to carry out experimental tests on complex, everyday nursing interventions and activities. We also need to create public space to present accounts of our endeavours pursuing this type of design in clinical practice. This paper will discuss some of the most important insights gained from conducting a quasi-experimental study in which the aim was to investigate the effect of a theory-based intervention, targeting knowledge and attitudes among registered nurses regarding cancer pain management. The importance of careful practical and methodological planning is emphasised, and the need for participation-friendly interventions is discussed.

Institutions:
(Gustafsson M) Department of Health Science, Blekinge Institute of Technology, SE-379 71 Blekinge, Sweden. Electronic address: markus.gustafsson@bth.se.; (Bohman DM) Department of Health Science, Blekinge Institute of Technology, SE-379 71 Blekinge, Sweden.; (Borglin G) Department of Health Science, Blekinge Institute of Technology, SE-379 71 Blekinge, Sweden.
(Gustafsson M) Department of Health Science, Blekinge Institute of Technology, SE-379 71 Blekinge, Sweden. Electronic address: markus.gustafsson@bth.se.; (Bohman DM) Department of Health Science, Blekinge Institute of Technology, SE-379 71 Blekinge, Sweden.; (Borglin G) Department of Health Science, Blekinge Institute of Technology, SE-379 71 Blekinge, Sweden.
(Gustafsson M) Department of Health Science, Blekinge Institute of Technology, SE-379 71 Blekinge, Sweden. Electronic address: markus.gustafsson@bth.se.; (Bohman DM) Department of Health Science, Blekinge Institute of Technology, SE-379 71 Blekinge, Sweden.; (Borglin G) Department of Health Science, Blekinge Institute of Technology, SE-379 71 Blekinge, Sweden.
Database: PubMed

139. GPs prescribing of strong opioid drugs for patients with chronic non-cancer pain: a qualitative study.

Author(s): Seamark D; Seamark C; Greaves C; Blake S

Source: The British journal of general practice : the journal of the Royal College of General Practitioners; Dec 2013; vol. 63 (no. 617); p. e821

Publication Date: Dec 2013

Publication Type(s): Journal Article; Research Support, Non-U.S. Gov't

DOI: http://dx.doi.org/10.3399/bjgp13X675403

ISSN: 1478-5242

Place of Publication: England

PubMedID: 24351498

Accession Number: 24351498

Available at The British journal of general practice : the journal of the Royal College of General Practitioners - from Europe PubMed Central - Open Access

Available at The British journal of general practice : the journal of the Royal College of General Practitioners - from HighWire - Free Full Text

Available at The British journal of general practice : the journal of the Royal College of General Practitioners - from EBSCO (MEDLINE Complete)

Available at The British journal of general practice : the journal of the Royal College of General Practitioners - from Unpaywall

Abstract:BACKGROUND: Chronic non-cancer pain (CNCP) is common in the UK. GPs manage most patients with such pain. Previous research has suggested that prescribing is influenced by patient and doctor factors, but less is known about the decision- making process involved in prescribing opioid drugs for CNCP.AIM: To describe the factors influencing GPs' prescribing of strong opioid drugs for CNCP. Design and setting Semi-structured interviews and a focus group of a purposive sample of GPs from a range of practice settings including male and female GPs with experience of prescribing strong opioids.METHOD: Transcripts of interviews and a focus group were analysed using qualitative research methodology (thematic analysis).RESULTS: GPs described prescribing opioid drugs for patients with CNCP as being different from treating cancer related pain. GPs followed accepted stepwise approaches in their prescribing for CNCP. They reported difficulty in assessing the level of pain and concern over duration of use of strong opioids and their possible side effects, tolerance, and addiction. Variation in reported practice was observed, which may be linked to experience and significant events.CONCLUSION: GPs in this study demonstrated a thoughtful attitude towards prescribing strong opioids for CNCP. They were aware of the difficulties of long-term strong opioid prescription. Only a few GPs had had specific training in chronic pain management and this may explain some of the variation in practice reported. GPs may benefit from training in pain assessment and long-term management of patients with CNCP.

Institutions:
(Seamark D) Honiton Research Practice, Honiton, Devon, UK.; (Seamark C) Honiton Research Practice, Honiton, Devon, UK.; (Greaves C) University of Exeter medical School (Primary Care), Exeter, UK.; (Blake S) Honiton Research Practice, Honiton, Devon, UK.
(Seamark D) Honiton Research Practice, Honiton, Devon, UK.; (Seamark C) Honiton Research Practice, Honiton, Devon, UK.; (Greaves C) University of Exeter medical School (Primary Care), Exeter, UK.; (Blake S) Honiton Research Practice, Honiton, Devon, UK.
(Seamark D) Honiton Research Practice, Honiton, Devon, UK.; (Seamark C) Honiton Research Practice, Honiton, Devon, UK.; (Greaves C) University of Exeter medical School (Primary Care), Exeter, UK.; (Blake S) Honiton Research Practice, Honiton, Devon, UK.
(Seamark D) Honiton Research Practice, Honiton, Devon, UK.; (Seamark C) Honiton Research Practice, Honiton, Devon, UK.; (Greaves C) University of Exeter medical School (Primary Care), Exeter, UK.; (Blake S) Honiton Research Practice, Honiton, Devon, UK.

Database: PubMed

140. [Cancer pain management: good clinical practices, use of strong opioids].

Author(s): Rostaing-Rigattieri S; Guerin J

Source: Presse medicale (Paris, France : 1983); Mar 2014; vol. 43 (no. 3); p. 252-262

Publication Date: Mar 2014

Publication Type(s): English Abstract; Journal Article; Review

DOI: http://dx.doi.org/10.1016/j.lpm.2013.05.005

ISSN: 2213-0276

Place of Publication: France

PubMedID: 24268466

Accession Number: 24268466

Abstract: Pain prevalence increases during cancer course disease. Cancer incidence is increasing in France, but thanks to therapeutic advances in the specific treatment, cancer has become a chronic disease, often associated with persistent pain. Cancer pain evaluation should be multidimensional. Pain mechanism (nociceptive, neuropathic or mixed pain) should be recognised in order to prescribe appropriate analgesic treatment. Cancer pain characteristics should be defined (baseline and breakthrough pain). Today, a wide range of pain medications, including strong opioids, are available. The use of analgesic combinations is strongly recommended for cancer pain management. Medication characteristics knowledge (pharmacodynamics and pharmacokinetics) is essential and must be associated with a good doctor-patient relationship, to ensure optimal treatment of the painful patient at all stages of the disease.

Institutions:
(Rostaing-Rigattieri S) Hôpital Saint-Antoine, centre d'évaluation et traitement de la douleur (CETD), 75012 Paris, France. Electronic address: sylvie.rostaing@sat.aphp.fr.; (Guerin J) Hôpital Saint-Antoine, centre d'évaluation et traitement de la douleur (CETD), 75012 Paris, France.
(Rostaing-Rigattieri S) Hôpital Saint-Antoine, centre d'évaluation et traitement de la douleur (CETD), 75012 Paris, France. Electronic address: sylvie.rostaing@sat.aphp.fr.; (Guerin J) Hôpital Saint-Antoine, centre d'évaluation et traitement de la douleur (CETD), 75012 Paris, France.
Database: PubMed

141. Can a theory-based educational intervention change nurses' knowledge and attitudes concerning cancer pain management? A quasi-experimental design.

Author(s): Gustafsson M; Borglin G

Source: BMC health services research; Aug 2013; vol. 13 ; p. 328

Publication Date: Aug 2013

Publication Type(s): Clinical Trial; Journal Article; Research Support, Non-U.S. Gov't

DOI: http://dx.doi.org/10.1186/1472-6963-13-328

ISSN: 1472-6963

Place of Publication: England

PubMedID: 23958335

Accession Number: 23958335

Available at BMC Health Services Research - from BioMed Central

Available at BMC Health Services Research - from Europe PubMed Central - Open Access

Available at BMC Health Services Research - from ProQuest (Health Research Premium) - NHS Version

Available at BMC Health Services Research - from EBSCO (MEDLINE Complete)

Available at BMC Health Services Research - from Unpaywall

Abstract:BACKGROUND: Registered Nurses (RNs) play an important role in caring for patients suffering from cancer pain. A lack of knowledge regarding pain management and the RNs' own perception of cancer pain could act as barriers to effective pain management. Educational interventions that target RNs' knowledge and attitudes have proved promising. However, an intervention consisting of evidence-based practice is a multifaceted process and demands behavioural and cognitive changes to sustain the effects of the intervention. Therefore, our study aimed to investigate if a theory-based educational intervention could change RNs' knowledge and attitudes to cancer pain and pain management, both four and 12 weeks after the start of the intervention.METHODS: A quasi-experimental design with non-equivalent control groups was used. The primary outcome was measured using a modified version of the instrument Nurses' Knowledge and Attitudes Survey Regarding Pain (NKAS) at baseline, four weeks and 12 weeks after the start of the intervention to evaluate its persistence. The intervention's educational curriculum was based on the principles of Ajzen's Theory of Planned Behaviour and consisted of interactive learning activities conducted in workshops founded on evidence-based knowledge. The RN's own experiences from cancer pain management were used in the learning process.RESULTS: The theory-based educational intervention aimed at changing RNs knowledge and attitudes regarding cancer pain management measured by primary outcome NKAS resulted in a statistical significant ($p<0.05$) improvement of total mean score from baseline to four weeks at the intervention ward.CONCLUSIONS: The findings of this study, suggest that a theory-based educational intervention focused at RNs can be effective in changing RN's knowledge and attitudes regarding cancer pain management. However, the high number of dropouts between baseline and four weeks needs to be taken into account when evaluating our findings. Finally, this kind of theory-based educational intervention with interactive learning activities has been sparsely researched and needs to be evaluated further in larger projects.TRIAL REGISTRATION: Clinical Trials. Gov: NCT01313234.

Institutions:
(Gustafsson M) School of Health Science, Blekinge Institute of Technology, Blekinge, SE-379 71, Sweden. markus.gustafsson@bth.se.
Database: PubMed

142. [Attitudes of Hungarian adults toward use of opioids in pain management].

Author(s): Biró E

Source: Orvosi hetilap; Mar 2013; vol. 154 (no. 12); p. 455-463

Publication Date: Mar 2013
Publication Type(s): English Abstract; Journal Article
DOI: http://dx.doi.org/10.1556/OH.2013.29569
ISSN: 0030-6002
Place of Publication: Hungary
PubMedID: 23506802
Accession Number: 23506802
Abstract:INTRODUCTION: Though the most effective pain medication is already available in Western countries, cancer pain is often undertreated. The primary causes of this phenomenon are the fears of opioids, which haven't been explored systematically in Hungary.AIMS: Exploration of the attitudes toward opioids among healthy Hungarian adults.METHODS: The participants (88/143 male/female, 29.47 ± 11.05 years) were asked to complete story of a patient who was suggested to accept opioid therapy. The stories were rated by independent raters on Likert-scales to access the strength of attitudes toward pain medication [1].RESULTS: Only an average 29.6% of the stories expressed positive attitudes, but varied fears (of addiction: 20.9%, nearing death: 10.5%, the inattention of the doctor: 4.7%), and hopes connected to alternative therapies (4.7%) emerged, too.CONCLUSIONS: The compliance of the patient and his/her family members is a very important factor of effective medical treatment, therefore individual attitudes must be considered.
Institutions:
(Biró E) ELTE Pszichológiai Intézet Affektív Pszichológia Tanszék Budapest Izabella u. 46. 1064 Magyar Hospice Alapítvány Budapest. eszter.biro@hospicehaz.hu
Database: PubMed

143. Barriers to cancer pain management: Jordanian nurses' perspectives.

Author(s): Al Khalaileh M; Al Qadire M
Source: International journal of palliative nursing; Nov 2012; vol. 18 (no. 11); p. 535
Publication Date: Nov 2012
Publication Type(s): Journal Article
DOI: http://dx.doi.org/10.12968/ijpn.2012.18.11.535
ISSN: 1357-6321
Place of Publication: England
PubMedID: 23413501
Accession Number: 23413501
Available at International journal of palliative nursing - from MAG Online Library
Available at International journal of palliative nursing - from EBSCO (CINAHL Complete)
Abstract:BACKGROUND: Adequate management of cancer pain is a human right. However, cancer pain is still not well medicated, and some of the barriers to achieving relief are related to nursing. Identifying these barriers would help the development of interventions to improve pain management.AIM: To explore barriers to cancer pain management among Jordanian nurses.METHOD: A convenience

sample of 96 nurses from 3 hospitals participated in a cross-sectional survey using an Arabic translation of Ward and colleagues' barriers questionnaire II.RESULTS: The nurses expressed high levels of barriers on the questionnaire, with a mean score of 2.5 for the questionnaire as a whole (standard deviation (SD) 0.8).The harmful and physiological effects of medications subscales received the highest mean scores: 2.7 (SD 1.1) and 2.6 (SD 0.9) respectively.CONCLUSIONS: Many nursing-related barriers to cancer pain management were found.These barriers need to be addressed and eliminated, for example through education and training.

Institutions:
(Al Khalaileh M) School of Nursing, Al Al-Bayait University, Mafraq, Jordan. m_khalailah@aabu.edu.jo

Database: PubMed

144. The appropriate treatment of chronic pain.

Author(s): Sarzi-Puttini P; Vellucci R; Zuccaro SM; Cherubino P; Labianca R; Fornasari D

Source: Clinical drug investigation; Feb 2012 ; p. 21-33

Publication Date: Feb 2012

Publication Type(s): Journal Article; Research Support, Non-U.S. Gov't; Review

DOI: http://dx.doi.org/10.2165/11630050-000000000-00000

ISSN: 1179-1918

Place of Publication: New Zealand

PubMedID: 23389873

Accession Number: 23389873

Abstract: Chronic pain is a common healthcare problem worldwide that ranks as a predominant reason for consulting a physician, yet effective management of chronic pain remains suboptimal, often resulting in unnecessary suffering and decreased quality of life, lost productivity and excessive healthcare costs. To overcome the challenges associated with the management of chronic pain, increased awareness and both patient and physician education are required. Improving physician knowledge of pain assessment and management guided by recommendations for a comprehensive, multifactorial, personalised treatment approach involving pharmacological and non-pharmacological approaches is key to achieving effective pain relief. Guidelines for the management of non-cancer and cancer pain recommend thorough patient assessment before individualized therapy based on the type and intensity of pain. The availability of mechanism-specific analgesics has facilitated improvements in the treatment of chronic non-cancer pain, which may be of neuropathic, muscle, inflammatory, mechanical/compressive or mixed origin. Stepwise escalation of analgesic therapy (paracetamol, non-steroidal anti-inflammatory drugs, mild to strong opioids) according to the World Health Organization's three-step pain ladder remains the standard approach for the selection of treatment for chronic cancer pain, although there is now a greater awareness of the requirements for effective administration of opioids including dose titration, use of short versus long-acting opioids, opioid rotation, management of adverse effects, and ongoing monitoring. Selection of an effective, appropriate,

personalized analgesic regimen for patients with chronic pain is achievable and is expected to enhance compliance, overall functioning and quality of life.

Institutions:
(Sarzi-Puttini P) Rheumatology Unit, L. Sacco University Hospital, Milan, Italy. sarzi.piercarlo@hsacco.it

Database: PubMed

145. How nurses assess breakthrough cancer pain, and the impact of this pain on patients' daily lives--results of a European survey.

Author(s): Rustøen T; Geerling JI; Pappa T; Rundström C; Weisse I; Williams SC; Zavratnik B; Wengström Y

Source: European journal of oncology nursing : the official journal of European Oncology Nursing Society; Aug 2013; vol. 17 (no. 4); p. 402-407

Publication Date: Aug 2013

Publication Type(s): Journal Article; Research Support, Non-U.S. Gov't

DOI: http://dx.doi.org/10.1016/j.ejon.2012.12.002

ISSN: 1532-2122

Place of Publication: Scotland

PubMedID: 23276599

Accession Number: 23276599

Available at European Journal of Oncology Nursing - from ScienceDirect

Available at European Journal of Oncology Nursing - from David Adams Library Journals Collection Local Print Collection [location] : David Adams Library.

Abstract:PURPOSE: To increase our knowledge of how nurses assess breakthrough cancer pain (BTCP); and whether they find it difficult to distinguish BTCP from background pain; how they estimate the impact of BTCP on patients' daily lives, and the factors that nurses consider to induce BTCP. Variations in their use of assessment tools and their ability to distinguish between different types of pain were also examined in terms of the number of years of oncology nursing experience and the practice in different countries.METHODS: In total, 1241 nurses (90% female) who care for patients with cancer, from 12 European countries, completed a survey questionnaire.KEY RESULTS: Half the sample had >9 years of experience in oncology nursing. Although 39% had no pain assessment tool to help them distinguish between types of pain, 95% of those who used a tool found it useful. Furthermore, 37% reported that they had problems distinguishing background pain from BTCP. Movement was identified as the factor that most commonly exacerbated BTCP across all countries. The nurses reported that BTCP greatly interfered with patients' everyday activities, and they rated the patients' enjoyment of life as most strongly affected. The use of tools and the ability to distinguish between different pains varied between European countries and with years of experience in oncology nursing.CONCLUSIONS: The nurses reported that BTCP greatly interfered with patients' lives, and many nurses had problems distinguishing between background pain and BTCP. Nurses require more knowledge about BTCP management, and guidelines should be developed for clinical use.

Institutions:

(Rustøen T) Division of Emergencies and Critical Care, Department of Research and Development, Ullevål, Oslo University Hospital, Postbox 4956, Nydalen, 0424 Oslo, Norway. tone.rustoen@rr-research.no
Database: PubMed

146. The PRO-SELF pain control program improves family caregivers' knowledge of cancer pain management.

Author(s): Valeberg BT; Kolstad E; Småstuen MC; Miaskowski C; Rustøen T

Source: Cancer nursing; 2013; vol. 36 (no. 6); p. 429-435

Publication Date: 2013

Publication Type(s): Journal Article; Randomized Controlled Trial; Research Support, Non-U.S. Gov't

DOI: http://dx.doi.org/10.1097/NCC.0b013e3182747bcf

ISSN: 1538-9804

Place of Publication: United States

PubMedID: 23154516

Accession Number: 23154516

Available at Cancer Nursing - from Ovid (Journals @ Ovid)

Available at Cancer Nursing - from Ovid (Journals @ Ovid) - London Health Libraries

Available at Cancer Nursing - from David Adams Library Journals Collection Local Print Collection [location] : David Adams Library.

Available at Cancer Nursing - from Unpaywall

Abstract:BACKGROUND: The majority of cancer treatment is provided in outpatient settings. Family caregivers' (FCs') knowledge and beliefs about pain and its management are critical components of effective care.OBJECTIVE: This study's aim was to evaluate the efficacy of a psychoeducational intervention, compared with control, to increase FCs' knowledge of cancer pain management.INTERVENTION/METHODS: Family caregivers of oncology outpatients were randomized together with the patients into the PRO-SELF Pain Control Program (n = 58) or a control group (n = 54). Family caregivers completed a demographic questionnaire and the Family Pain Questionnaire (FPQ) at the beginning and end of the study to assess their knowledge about pain and its management. The intervention consisted of nurse coaching, home visits, and phone calls that occurred over 6 weeks.RESULTS: One hundred twelve FCs (60% female) with a mean age of 63 (SD, 10.7) years participated. Compared with FCs in the control group, FCs in the PRO-SELF group had significantly higher knowledge scores on all of the single items on the FPQ, except for the item "cancer pain can be relieved," as well as for the total FPQ score.CONCLUSION: The use of a knowledge and attitude survey like the FPQ, as part of a psychoeducational intervention provides an effective foundation for FC education about cancer pain management.IMPLICATIONS FOR PRACTICE: Oncology nurses can use FCs' responses to the FPQ to individualize teaching and spend more time on identified knowledge deficits. This individualized approach to FC education may save staff time and improve patient outcomes.

Institutions:
(Valeberg BT) Author Affiliations: Faculty of Nursing, Oslo and Akershus University College (Drs Valeberg and Småstuen), and Cancer Clinic, Oslo University Hospital, Ullevål (Ms Kolstad), Norway; Department of Physiological Nursing, University of California, San Francisco (Dr Miaskowski); and Emergency Care Clinic, Oslo University Hospital, Ullevål (Dr Rustøen), and Lovisenberg Diaconal College, Oslo (Dr Rustøen), Norway.
Database: PubMed

147. Interventional pain management in the palliative care patient.

Author(s): McHugh ME; Miller-Saultz D; Wuhrman E; Kosharskyy B

Source: International journal of palliative nursing; Sep 2012; vol. 18 (no. 9); p. 426

Publication Date: Sep 2012

Publication Type(s): Journal Article

DOI: http://dx.doi.org/10.12968/ijpn.2012.18.9.426

ISSN: 1357-6321

Place of Publication: England

PubMedID: 23124052

Accession Number: 23124052

Available at International journal of palliative nursing - from MAG Online Library

Available at International journal of palliative nursing - from EBSCO (CINAHL Complete)

Abstract: For the majority of patients, cancer pain can be treated using the World Health Organization cancer pain guidelines; however, for 10-20% of patients with advanced cancer, adequate pain control cannot be achieved using these methods owing to disease pathophysiology preventing administration/absorption of pain medications or intolerance due to opioid toxicities. The need to expand analgesic treatment when oral, transdermal, and intravenous therapies fail requires exploration of interventional pain management techniques such as neuraxial (e.g. epidural and intrathecal) infusion therapies and neurolytic interventions. Nurses caring for patients with cancer pain should develop their knowledge of these multimodal approaches to cancer pain management.

Institutions:
(McHugh ME) Palliative Care Service, Montefiore Medical Center. mm234@columbia.edu
Database: PubMed

148. Results of a randomized controlled pilot study of a self-management intervention for cancer pain.

Author(s): Koller A; Miaskowski C; De Geest S; Opitz O; Spichiger E

Source: European journal of oncology nursing : the official journal of European Oncology Nursing Society; Jun 2013; vol. 17 (no. 3); p. 284-291

Publication Date: Jun 2013

Publication Type(s): Comparative Study; Journal Article; Randomized Controlled Trial; Research Support, Non-U.S. Gov't

DOI: http://dx.doi.org/10.1016/j.ejon.2012.08.002

ISSN: 1532-2122

Place of Publication: Scotland

PubMedID: 22959603

Accession Number: 22959603

Available at European journal of oncology nursing : the official journal of European Oncology Nursing Society - from ScienceDirect

Available at European journal of oncology nursing : the official journal of European Oncology Nursing Society - from David Adams Library Journals Collection Local Print Collection [location] : David Adams Library.

Abstract:PURPOSE OF THE RESEARCH: This paper reports findings from a randomized controlled pilot study evaluating the PRO-SELF Plus Pain Control Program, a U.S.-developed cancer pain self-management intervention, regarding feasibility and effect sizes in a German patient sample.METHODS AND SAMPLE: Thirty-nine German oncology outpatients were randomized to intervention (n = 19) and control (n = 20) groups. The intervention group received the PRO-SELF Plus Pain Control Program in 6 visits and 4 phone calls a 10-week period. The control group received standard education and care. The intervention employed three key strategies: information provision, skills building, and nurse coaching. Primary outcomes were changes in average and worst pain intensity. Secondary outcomes included changes in pain-related knowledge, opioid intake, and self-efficacy. Data were collected at enrollment, then at 6, 10, 14, and 22 weeks.KEY RESULTS: The group-by-time effect showed a statistically significant increase in knowledge (week 10: $p = 0.04$; week 22: $p < 0.01$). Despite slight reductions in average and worst pain, no statistically significant changes were found for pain, opioid intake, or self-efficacy.CONCLUSIONS: This study is the first to evaluate and demonstrate the feasibility of a U.S.-developed cancer pain self-management intervention in a German patient population. Pain self-management related knowledge improved significantly and effect sizes for pain reduction were determined. Findings from this pilot RCT provide the basis for planning a larger RCT.CLINICAL TRIAL REGISTRATION NUMBER: NCT00920504.

Institutions:
(Koller A) Institute of Nursing Science, Faculty of Medicine, University of Basel, Switzerland.
Database: PubMed

149. Cancer pain part 2: assessment and management.

Author(s): Chapman S

Source: Nursing standard (Royal College of Nursing (Great Britain) : 1987); 2012; vol. 26 (no. 48); p. 44-49

Publication Date: 2012

Publication Type(s): Journal Article

DOI: http://dx.doi.org/10.7748/ns2012.08.26.48.44.c9229

ISSN: 0029-6570

Place of Publication: England

PubMedID: 22916657

Accession Number: 22916657

Available at Nursing standard (Royal College of Nursing (Great Britain) : 1987) - from ProQuest (MEDLINE with Full Text) - NHS Version

Available at Nursing standard (Royal College of Nursing (Great Britain) : 1987) - from ProQuest (Health Research Premium) - NHS Version

Abstract: Pain is common in patients with cancer and may be caused by the disease itself or treatments. Part 1 of this article identified the causes and types of cancer pain to inform assessment and management of pain, which will be discussed in this article. Barriers to pain management and the non-medical prescribing role of the advanced practice nurse in treating patients with cancer pain will be explored.

Institutions:
(Chapman S) Clinical Services Division, The Royal Marsden NHS Foundation Trust, London. suzanne.chapman@rmh.nhs.UK
Database: PubMed

150. Opioid epidemic in the United States.

Author(s): Manchikanti L; Helm S; Fellows B; Janata JW; Pampati V; Grider JS; Boswell MV

Source: Pain physician; Jul 2012; vol. 15 (no. 3 Suppl); p. ES9

Publication Date: Jul 2012

Publication Type(s): Journal Article; Review

ISSN: 2150-1149

Place of Publication: United States

PubMedID: 22786464

Accession Number: 22786464

Available at Pain physician - from EBSCO (MEDLINE Complete)

Abstract: Over the past two decades, as the prevalence of chronic pain and health care costs have exploded, an opioid epidemic with adverse consequences has escalated. Efforts to increase opioid use and a campaign touting the alleged undertreatment of pain continue to be significant factors in the escalation. Many arguments in favor of opioids are based solely on traditions, expert opinion, practical experience and uncontrolled anecdotal observations. Over the past 20 years, the liberalization of laws governing the prescribing of opioids for the treatment of chronic non-cancer pain by the state medical boards has led to dramatic increases in opioid use. This has evolved into the present stage, with the introduction of new pain management standards by the Joint Commission on the Accreditation of Healthcare Organizations (JCAHO) in 2000, an increased awareness of the right to pain relief, the support of various organizations supporting the use of opioids in large doses, and finally, aggressive marketing by the pharmaceutical industry. These positions are based on unsound science and blatant misinformation, and accompanied by the dangerous assumptions that opioids are highly effective and safe, and devoid of

adverse events when prescribed by physicians. Results of the 2010 National Survey on Drug Use and Health (NSDUH) showed that an estimated 22.6 million, or 8.9% of Americans, aged 12 or older, were current or past month illicit drug users, The survey showed that just behind the 7 million people who had used marijuana, 5.1 million had used pain relievers. It has also been shown that only one in 6 or 17.3% of users of non-therapeutic opioids indicated that they received the drugs through a prescription from one doctor. The escalating use of therapeutic opioids shows hydrocodone topping all prescriptions with 136.7 million prescriptions in 2011, with all narcotic analgesics exceeding 238 million prescriptions. It has also been illustrated that opioid analgesics are now responsible for more deaths than the number of deaths from both suicide and motor vehicle crashes, or deaths from cocaine and heroin combined. A significant relationship exists between sales of opioid pain relievers and deaths. The majority of deaths (60%) occur in patients when they are given prescriptions based on prescribing guidelines by medical boards, with 20% of deaths in low dose opioid therapy of 100 mg of morphine equivalent dose or less per day and 40% in those receiving morphine of over 100 mg per day. In comparison, 40% of deaths occur in individuals abusing the drugs obtained through multiple prescriptions, doctor shopping, and drug diversion. The purpose of this comprehensive review is to describe various aspects of crisis of opioid use in the United States. The obstacles that must be surmounted are primarily inappropriate prescribing patterns, which are largely based on a lack of knowledge, perceived safety, and inaccurate belief of undertreatment of pain.

Institutions:
(Manchikanti L) Pain Management Center of Paducah, Paducah, KY, USA. drlm@thepainmd.com

Database: PubMed

151. The PRO-SELF(©) Pain Control Program improves patients' knowledge of cancer pain management.

Author(s): Rustøen T; Valeberg BT; Kolstad E; Wist E; Paul S; Miaskowski C

Source: Journal of pain and symptom management; Sep 2012; vol. 44 (no. 3); p. 321-330

Publication Date: Sep 2012

Publication Type(s): Journal Article; Randomized Controlled Trial; Research Support, Non-U.S. Gov't

DOI: http://dx.doi.org/10.1016/j.jpainsymman.2011.09.015

ISSN: 1873-6513

Place of Publication: United States

PubMedID: 22704056

Accession Number: 22704056

Available at Journal of pain and symptom management - from ScienceDirect

Available at Journal of pain and symptom management - from David Adams Library Journals Collection Local Print Collection [location] : David Adams Library.

Available at Journal of pain and symptom management - from Unpaywall

Abstract:CONTEXT: Inadequate knowledge is one barrier to effective cancer pain management.OBJECTIVES: This study's aim was to evaluate the effects of a psychoeducational intervention (the Norwegian version of the PRO-SELF(©) Pain Control Program) compared with a control group in increasing patients' knowledge of cancer pain management.METHODS: Adult oncology outpatients with pain from bone metastasis of 2.5 or greater on a 0 to 10 numeric rating scale were randomized into the PRO-SELF (n=87) or control (n=92) groups. Patients completed a demographic questionnaire and the Pain Experience Scale (PES) at the beginning and end of the study to assess their knowledge and attitudes. The six-week intervention consisted of education, skills building, and nurse coaching. Mixed-model analyses with tests of a group×time interaction were done for each of the individual items and total PES scores to evaluate between-group differences in changes in knowledge over time.RESULTS: Except for functional status, no differences were found between the PRO-SELF and control groups on any baseline demographic, clinical, or pain characteristics. Significant group×time interactions were found for all the single item and total PES scores. Compared with the control group, patients in the PRO-SELF group had significant increases in knowledge scores.CONCLUSION: The use of a knowledge and attitude survey, like the PES, as part of a psychoeducational intervention provides an effective foundation for patient education in cancer pain management. This individualized approach to education about pain management may save staff time and improve patient outcomes.

Institutions:
(Rustøen T) Department of Research and Development, Oslo University Hospital, Ullevål, Oslo, Norway. tone.rustoen@rr-research.no
Database: PubMed

152. The appropriate treatment of chronic pain.

Author(s): Sarzi-Puttini P; Vellucci R; Zuccaro SM; Cherubino P; Labianca R; Fornasari D

Source: Clinical drug investigation; Feb 2012 ; p. 21-33

Publication Date: Feb 2012

Publication Type(s): Journal Article; Review

DOI: http://dx.doi.org/10.2165/11630050-000000000-00000

ISSN: 1179-1918

Place of Publication: New Zealand

PubMedID: 22356221

Accession Number: 22356221

Abstract: Chronic pain is a common healthcare problem worldwide that ranks as a predominant reason for consulting a physician, yet effective management of chronic pain remains suboptimal, often resulting in unnecessary suffering and decreased quality of life, lost productivity and excessive healthcare costs. To overcome the challenges associated with the management of chronic pain, increased awareness and both patient and physician education are required. Improving physician knowledge of pain assessment and management guided by recommendations for a comprehensive, multifactorial, personalised treatment approach involving pharmacological and non-pharmacological approaches is key to achieving effective

pain relief. Guidelines for the management of non-cancer and cancer pain recommend thorough patient assessment before individualized therapy based on the type and intensity of pain. The availability of mechanism-specific analgesics has facilitated improvements in the treatment of chronic non-cancer pain, which may be of neuropathic, muscle, inflammatory, mechanical/compressive or mixed origin. Stepwise escalation of analgesic therapy (paracetamol, non-steroidal anti-inflammatory drugs, mild to strong opioids) according to the World Health Organization's three-step pain ladder remains the standard approach for the selection of treatment for chronic cancer pain, although there is now a greater awareness of the requirements for effective administration of opioids including dose titration, use of short versus long-acting opioids, opioid rotation, management of adverse effects, and ongoing monitoring. Selection of an effective, appropriate, personalized analgesic regimen for patients with chronic pain is achievable and is expected to enhance compliance, overall functioning and quality of life.

Institutions:
(Sarzi-Puttini P) Rheumatology Unit, L Sacco University Hospital, Milan, Italy. sarzi.piercarlo@hsacco.it
Database: PubMed

153. Pain in clinical oncology: patient satisfaction with management of cancer pain.

Author(s): Antón A; Montalar J; Carulla J; Jara C; Batista N; Camps C; Cassinello J; Sanz-Ortiz J; Díaz-Rubio E; Martínez C; Ledesma F; Zubillaga E; ALGOS Group; DOME III Study Group

Source: European journal of pain (London, England); Mar 2012; vol. 16 (no. 3); p. 381-389

Publication Date: Mar 2012

Publication Type(s): Journal Article; Multicenter Study; Research Support, Non-U.S. Gov't

DOI: http://dx.doi.org/10.1002/j.1532-2149.2011.00036.x

ISSN: 1532-2149

Place of Publication: England

PubMedID: 22337158

Accession Number: 22337158

Available at European journal of pain (London, England) - from Wiley Online Library Medicine and Nursing Collection 2020

Available at European journal of pain (London, England) - from EBSCO (MEDLINE Complete)

Available at European journal of pain (London, England) - from Unpaywall

Abstract:BACKGROUND: Despite effective analgesic therapy, inadequate pain control is frequently perceived by patients and caregivers.AIMS: To assess satisfaction with management of pain in cancer patients.METHODS: Between January and May 2007, a cross-sectional multicentre study was conducted in 64 Medical Oncology Departments throughout Spain. A total of 525 outpatients with oncological diseases completed a questionnaire with demographic data,

characteristics and intensity of pain, and perceptions and attitudes towards pain management at the time of a routine clinical visit. Physicians also completed a questionnaire with tumour-related and treatment-related data. Cluster analysis was used to classify patients into three groups (satisfied, neither satisfied nor dissatisfied or neutral, dissatisfied) according to pain intensity and satisfaction with treatment.RESULTS: Patients satisfied with their analgesic treatment (33%) had lower pain intensities and, when regularly asked about their pain, considered their physicians to be more involved in their treatment. Neither satisfied nor dissatisfied patients (neutral) (44%) had higher mean pain intensities. Two-thirds of them achieved marked relief of their pain and also thought that physicians were aware of their situation. Dissatisfied patients (23%) had moderate to severe pain intensities, and said that they were asked less frequently about their pain, and thought that their physicians were less involved in their analgesic treatment.CONCLUSION: Physician-patient communication and information provided to patients are essential aspects of patient perceptions and attitudes towards control of cancer-related pain. Pain is seen as a condition that may be controlled but affects the capacity to lead a normal life.

Institutions:
(Antón A) Service of Medical Oncology, Hospital Universitario Miguel Servet, Zaragoza, Spain.; (Montalar J) ALGOS Group; (Carulla J) DOME III Study Group
(Antón A) Service of Medical Oncology, Hospital Universitario Miguel Servet, Zaragoza, Spain.; (Montalar J) ALGOS Group; (Carulla J) DOME III Study Group
(Antón A) Service of Medical Oncology, Hospital Universitario Miguel Servet, Zaragoza, Spain.; (Montalar J) ALGOS Group; (Carulla J) DOME III Study Group
Database: PubMed

154. [Cancer patients adherence and symptom management: the influence of the patient-physician relationship].

Author(s): Chou PL; Lin CC

Source: Hu li za zhi The journal of nursing; Feb 2012; vol. 59 (no. 1); p. 11-15

Publication Date: Feb 2012

Publication Type(s): English Abstract; Journal Article; Review

ISSN: 0047-262X

Place of Publication: China (Republic : 1949-)

PubMedID: 22314645

Accession Number: 22314645

Available at Hu li za zhi The journal of nursing - from EBSCO (CINAHL Complete)

Available at Hu li za zhi The journal of nursing - from ProQuest (MEDLINE with Full Text) - NHS Version

Abstract: Appropriate management of cancer symptoms positively influences quality of life. Inappropriate symptom management, disease recurrence, co-morbidity and death result from lack of medication adherence. Factors influencing adherence are multidimensional and include personal attributes, knowledge and beliefs regarding the disease and medication, self-efficacy and social support. The patient-physician relationship is a significant factor of influence in patient medication adherence and ultimate positive outcomes. If patients are satisfied with the relationship, they are highly likely to comply with health provider advice. Improving patient-physician

relationships can effectively promote patient medication adherence. In cancer pain management and adjuvant hormone therapy for breast cancer, regular follow-up examinations have a positive impact. The physician-patient relationship with regard to cancer patient satisfaction is a relatively new topic of discussion, and many implications are scarcely understood. However, the physician-patient relationship should not be ignored as a factor in cancer symptoms management, especially with regard to promoting compliance behavior.

Institutions:
(Chou PL) Department of Nursing, Chung Hwa University of Medical Technology.
Database: PubMed

155. The survey of nurse's knowledge and attitude toward cancer pain management: Application of Health Belief Model.

Author(s): Shahnazi H; Saryazdi H; Sharifirad G; Hasanzadeh A; Charkazi A; Moodi M

Source: Journal of education and health promotion; 2012; vol. 1 ; p. 15

Publication Date: 2012

Publication Type(s): Journal Article

DOI: http://dx.doi.org/10.4103/2277-9531.98573

ISSN: 2277-9531

Place of Publication: India

PubMedID: 23555118

Accession Number: 23555118

Available at Journal of education and health promotion - from Europe PubMed Central - Open Access

Available at Journal of education and health promotion - from ProQuest (Health Research Premium) - NHS Version

Available at Journal of education and health promotion - from Unpaywall

Keywords: Subject Terms: Attitude; Health Belief Model; cancer; knowledge; nurse; pain management

Abstract:BACKGROUND: Effective cancer pain management requires accurate knowledge, attitudes, and assessment skills. The purpose of this study was to obtain information about the knowledge and attitudes of nurses concerning cancer pain management with the use Health Belief Model (HBM) as conceptual framework.MATERIALS AND METHODS: The study was a descriptive survey and included 98 randomly selected nurses from Alzahra hospital, Isfahan, Iran. A self-administered questionnaire which was designed on the basis of HBM was used to collect the data. Knowledge, attitudes, and HBM constructs regarding cancer pain were the main research variables. The obtained data were analyzed by SPSS (version11.5) using descriptive statistics, independent t-test, and Pearson correlation at the significant level of $\alpha=0.05$.RESULTS: Ninety-eight nurses aged 38.7 ± 7.04 years were studied in this survey. From the 10 pain knowledge questions assessed, the mean number of correctly answered question was 61.2 (SD=16.5), with a range of 30-100. There was a direct correlation between knowledge and attitude of nurses with HBM constructs except for perceived barriers and perceived threat. Among the

HBM constructs, the highest score was related to self-efficacy with mean score of 87.2 (SD=16.4).CONCLUSIONS: The findings support the concern of inadequate knowledge and attitudes in relation to cancer pain management. We believe that basic and continuing education programs may improve the knowledge level of nursing about pain management.

Institutions:
(Shahnazi H) Department of Health Education and Promotion, Isfahan University of Medical Sciences, Isfahan, Iran.
Database: PubMed

156. A randomized, clinical trial of education or motivational-interviewing-based coaching compared to usual care to improve cancer pain management.

Author(s): Thomas ML; Elliott JE; Rao SM; Fahey KF; Paul SM; Miaskowski C

Source: Oncology nursing forum; Jan 2012; vol. 39 (no. 1); p. 39-49

Publication Date: Jan 2012

Publication Type(s): Journal Article; Multicenter Study; Randomized Controlled Trial; Research Support, U.S. Gov't, Non-P.H.S.

DOI: http://dx.doi.org/10.1188/12.ONF.39-49

ISSN: 1538-0688

Place of Publication: United States

PubMedID: 22201654

Accession Number: 22201654

Available at Oncology Nursing Forum - from Ovid (Journals @ Ovid)

Available at Oncology Nursing Forum - from EBSCO (CINAHL Complete)

Available at Oncology Nursing Forum - from ProQuest (MEDLINE with Full Text) - NHS Version

Available at Oncology Nursing Forum - from ProQuest (Health Research Premium) - NHS Version

Available at Oncology Nursing Forum - from David Adams Library Journals Collection Local Print Collection [location] : David Adams Library.

Abstract:PURPOSE/OBJECTIVES: To test the effectiveness of two interventions compared to usual care in decreasing attitudinal barriers to cancer pain management, decreasing pain intensity, and improving functional status and quality of life (QOL).DESIGN: Randomized clinical trial.SETTING: Six outpatient oncology clinics (three Veterans Affairs [VA] facilities, one county hospital, and one community-based practice in California, and one VA clinic in New Jersey)Sample: 318 adults with various types of cancer-related pain.METHODS: Patients were randomly assigned to one of three groups: control, standardized education, or coaching. Patients in the education and coaching groups viewed a video and received a pamphlet on managing cancer pain. In addition, patients in the coaching group participated in four telephone sessions with an advanced practice nurse interventionist using motivational interviewing techniques to decrease attitudinal barriers to cancer pain management. Questionnaires were completed at baseline and six weeks after the final telephone calls. Analysis of covariance was used to

evaluate for differences in study outcomes among the three groups.MAIN RESEARCH VARIABLES: Pain intensity, pain relief, pain interference, attitudinal barriers, functional status, and QOL.FINDINGS: Attitudinal barrier scores did not change over time among groups. Patients randomized to the coaching group reported significant improvement in their ratings of pain-related interference with function, as well as general health, vitality, and mental health.CONCLUSIONS: Although additional evaluation is needed, coaching may be a useful strategy to help patients decrease attitudinal barriers toward cancer pain management and to better manage their cancer pain.IMPLICATIONS FOR NURSING: By using motivational interviewing techniques, advanced practice oncology nurses can help patients develop an appropriate plan of care to decrease pain and other symptoms.

Institutions:
(Thomas ML) Veterans Administration Palo Alto Healthcare System, California, USA. mary.thomas4@va.gov

Database: PubMed

157. [Physicians' knowledge on cancer pain therapy : Comparison of palliative care and prehospital emergency physicians in training].

Author(s): Wiese CH; Lassen CL; Vormelker J; Meyer N; Popov AF; Graf BM; Hanekop GG; Wirz S

Source: Schmerz (Berlin, Germany); Dec 2011; vol. 25 (no. 6); p. 654-662

Publication Date: Dec 2011

Publication Type(s): Comparative Study; English Abstract; Journal Article

DOI: http://dx.doi.org/10.1007/s00482-011-1110-8

ISSN: 1432-2129

Place of Publication: Germany

PubMedID: 22120919

Accession Number: 22120919

Available at Schmerz (Berlin, Germany) - from EBSCO (MEDLINE Complete)

Abstract:BACKGROUND: Palliative care needs a high level of expertise. In particular, there are some potential difficulties in the treatment of patients with the symptom cancer pain (for example lack of education). In Germany, various physicians are involved in cancer pain treatment but in general palliative care patients are treated by a physician who is educated in palliative medicine. In special circumstances prehospital emergency physicians and other physicians are involved in therapy decisions in palliative care patients as well. The authors surveyed different groups of physicians in Germany about their specific knowledge of cancer pain management.MATERIAL AND METHODS: A self-designed, standardized questionnaire (50 items) was given to palliative physicians in training (PP). The survey asked prospectively for knowledge on the World Health Organization (WHO) step ladder of cancer pain therapy. The results were retrolectively compared with an earlier investigation with the same background (emergency physicians in training EP).RESULTS: There was a 99.5% response rate with a total of 654 respondents (PP 185, EP 469) and 461 (70.5%) of the respondents had knowledge of the WHO step ladder for the treatment of cancer pain [PP 164/185 (88.6%), EP 297/469 (63.3%), PP versus EP $p < 0.001$)]. The correct numbers of therapeutic levels were

known by 361/461 participants [PP 151/164 (92.1%), EP 210/297 (70.7%), $p < 0.001$].The EPs with a professional experience less than 5 years answered statistically significantly more questions correctly ($p = 0.004$). Concerning the defined parameters knowledge and professional experience, there was no statistically significant difference in the group of PP.CONCLUSIONS: The results of this study verified that the highest knowledge scores were achieved by PPs and overall, the knowledge scores showed an improvement in comparison to previous investigations. In recent years there seems to have been an improvement in education on pain treatment,for example during medical school. Whether this also leads to an improvement of patient care and the relevance of these data for the clinical practice needs to be investigated in further studies.

Institutions:
(Wiese CH) Klinik für Anästhesiologie, Universitätsklinikum Regensburg, Franz-Josef-Strauß-Allee 11, 93053, Regensburg, Deutschland.
christoph.wiese@klinik.uni-regensburg.de
Database: PubMed

158. Medical oncologists' attitudes and practice in cancer pain management: a national survey.

Author(s): Breuer B; Fleishman SB; Cruciani RA; Portenoy RK

Source: Journal of clinical oncology : official journal of the American Society of Clinical Oncology; Dec 2011; vol. 29 (no. 36); p. 4769-4775

Publication Date: Dec 2011

Publication Type(s): Journal Article; Research Support, Non-U.S. Gov't

DOI: http://dx.doi.org/10.1200/JCO.2011.35.0561

ISSN: 1527-7755

Place of Publication: United States

PubMedID: 22084372

Accession Number: 22084372

Abstract:PURPOSE: To evaluate the attitudes, knowledge, and practices of US medical oncologists that are related to management of cancer pain.METHODS: An anonymous survey was mailed to a geographically representative sample of medical oncologists randomly selected from the American Medical Association's Physician Master File.RESULTS: From a total of 2,000 oncologists, 354 responded to the original questionnaire and 256 responded to one of two subsequent shortened versions (overall response rate, 32%). Responders were demographically similar to all US medical oncologists. Using numeric rating scales of 0 to 10, oncologists rated their specialty highly for the ability to manage cancer pain (median, 7; interquartile range [IQR], 6 to 8) but rated their peers as more conservative prescribers than themselves (median, 3; IQR, 2 to 5). The quality of pain management training during medical school and residency was rated as 3 (IQR, 1 to 5) and 5 (IQR, 3 to 7), respectively. The most important barriers to pain management were poor assessment (median, 6; IQR, 4 to 7) and patient reluctance to take opioids (median, 6; IQR, 5 to 7) or report pain (median, 6; IQR, 4 to 7). Other barriers included physician reluctance to prescribe opioids (median, 5; IQR, 3 to 7) and perceived excessive regulation (median, 4; IQR, 2 to 7). In response to two vignettes

describing challenging clinical scenarios, 60% and 87%, respectively, endorsed treatment decisions that would be considered unacceptable by pain specialists. Frequent referrals to pain or palliative care specialists were reported by only 14% and 16%, respectively.CONCLUSION: These data suggest that, for more than 20 years, a focus on cancer pain has not adequately addressed the perception of treatment barriers or limitations in pain-related knowledge and practice within the oncology community. Additional efforts are needed to achieve meaningful progress.

Institutions:
(Breuer B) Beth Israel Medical Center, New York, NY 10003, USA. bbreuer@chpnet.org

Database: PubMed

159. Attitudes, beliefs, and practices of Sri Lankan nurses toward cancer pain management: an ethnographic study.

Author(s): De Silva BS; Rolls C

Source: Nursing & health sciences; Dec 2011; vol. 13 (no. 4); p. 419-424

Publication Date: Dec 2011

Publication Type(s): Journal Article

DOI: http://dx.doi.org/10.1111/j.1442-2018.2011.00635.x

ISSN: 1442-2018

Place of Publication: Australia

PubMedID: 21902777

Accession Number: 21902777

Available at Nursing & health sciences - from Wiley Online Library Medicine and Nursing Collection 2020

Abstract: Cancer pain is a serious problem that requires specialized nursing knowledge. In the present ethnographic study, we sought to explore the experiences and cancer pain management practices of nurses working at a government hospital in Sri Lanka. Data were collected from October 2007 to January 2008, and were obtained by observing the nurses in a cancer ward, conducting semistructured interviews with 10 participants, and maintaining a research diary. To analyze the data, the data were coded, and an integrative process was implemented to develop categories. The results suggested that Sri Lankan nurses perform poor cancer pain management practices due to a lack of resources, a shortage of nurses, and poor workload allocation within the hospital. Additionally, the nurses are not autonomous, and are required to refer to medical staff for cancer pain management strategies. The nurses work in a task-oriented system that rarely acknowledges cancer patients' pain management needs. This study might improve nursing pain management practices for cancer patients and lead to changes in the curriculum of nursing courses in Sri Lanka.

Institutions:
(De Silva BS) Department of Health Sciences, The Open University of Sri Lanka, Nawala, Nugegoda, Sri Lanka. bssil@ou.ac.lk

Database: PubMed

160. Primary care providers' perspective on prescribing opioids to older adults with chronic non-cancer pain: a qualitative study.

Author(s): Spitz A; Moore AA; Papaleontiou M; Granieri E; Turner BJ; Reid MC

Source: BMC geriatrics; Jul 2011; vol. 11 ; p. 35

Publication Date: Jul 2011

Publication Type(s): Comparative Study; Evaluation Study; Journal Article; Research Support, N.I.H., Extramural; Research Support, Non-U.S. Gov't

DOI: http://dx.doi.org/10.1186/1471-2318-11-35

ISSN: 1471-2318

Place of Publication: England

PubMedID: 21752299

Accession Number: 21752299

Available at BMC Geriatrics - from BioMed Central

Available at BMC Geriatrics - from Europe PubMed Central - Open Access

Available at BMC Geriatrics - from ProQuest (Health Research Premium) - NHS Version

Available at BMC Geriatrics - from EBSCO (MEDLINE Complete)

Available at BMC Geriatrics - from Unpaywall

Abstract:BACKGROUND: The use of opioid medications as treatment for chronic non-cancer pain remains controversial. Little information is currently available regarding healthcare providers' attitudes and beliefs about this practice among older adults. This study aimed to describe primary care providers' experiences and attitudes towards, as well as perceived barriers and facilitators to prescribing opioids as a treatment for chronic pain among older adults.METHODS: Six focus groups were conducted with a total of 23 physicians and three nurse practitioners from two academically affiliated primary care practices and three community health centers located in New York City. Focus groups were audiotape recorded and transcribed. The data were analyzed using directed content analysis; NVivo software was used to assist in the quantification of identified themes.RESULTS: Most participants (96%) employed opioids as therapy for some of their older patients with chronic pain, although not as first-line therapy. Providers cited multiple barriers, including fear of causing harm, the subjectivity of pain, lack of education, problems converting between opioids, and stigma. New barriers included patient/family member reluctance to try an opioid and concerns about opioid abuse by family members/caregivers. Studies confirming treatment benefit, validated tools for assessing risk and/or dosing for comorbidities, improved conversion methods, patient education, and peer support could facilitate opioid prescribing. Participants voiced greater comfort using opioids in the setting of delivering palliative or hospice care versus care of patients with chronic pain, and expressed substantial frustration managing chronic pain.CONCLUSIONS: Providers perceive multiple barriers to prescribing opioids to older adults with chronic pain, and use these medications cautiously. Establishing the long-term safety and efficacy of these medications, generating improved prescribing methods, and implementing provider and patient educational interventions could help to improve the management of chronic pain in later life.

Institutions:
(Spitz A) Department of Internal Medicine, Virginia Mason Medical Center, Seattle, WA, USA.
Database: PubMed

161. Medical students' knowledge and attitude toward cancer pain management in Saudi Arabia.

Author(s): Kaki AM

Source: Saudi medical journal; Jun 2011; vol. 32 (no. 6); p. 628-632

Publication Date: Jun 2011

Publication Type(s): Journal Article

ISSN: 0379-5284

Place of Publication: Saudi Arabia

PubMedID: 21666947

Accession Number: 21666947

Abstract:OBJECTIVE: To assess the final year medical students' knowledge, beliefs, and attitude toward cancer pain, and the need for a formal pain curriculum in medical schools.METHODS: An epidemiological study was conducted from May 2008 to October 2009 at King Abdulaziz University Hospital, Jeddah, Kingdom of Saudi Arabia to assess the students' knowledge and attitude toward cancer pain management. A survey in the form of self-conducted questionnaire was distributed among them.RESULTS: Response rate was 55% (N=325). Fifty-four percent of the respondents believed that <40% of cancer patients suffered from pain. Forty-six percent of them considered cancer pain as untreatable, while 41.6% considered pain as a minor problem, and 58.6% considered the risk of addiction is high with legitimate opioids' prescription. There are 23.1% of students believed that patients are poor judges of their pain, 68% of them limited opioids prescription to patients with poor prognosis, and 77.1% believed that drug tolerance or psychological dependence, rather than advanced stages' cancer is the cause of increasing analgesic doses. The students' knowledge on the causes of cancer pain, pain clinic rule, and pain inclusion in the medical curriculum was poor. The correlation between personal life experience and respondents' attitude toward cancer pain management did not reveal any statistical significant.CONCLUSION: The study revealed poor knowledge and negative attitude of medical students' toward cancer pain. A structured teaching pain program is needed to improve the knowledge and attitude of future doctors toward pain.

Institutions:
(Kaki AM) Department of Anesthesia and Critical Care, Faculty of Medicine, King Abdulaziz University Hospital, Jeddah, Kingdom of Saudi Arabia.
amkaki@yahoo.com
Database: PubMed

162. A pain education programme to improve patient satisfaction with cancer pain management: a randomised control trial.

Author(s): Chou PL; Lin CC

Source: Journal of clinical nursing; Jul 2011; vol. 20 (no. 13-14); p. 1858-1869
Publication Date: Jul 2011
Publication Type(s): Journal Article; Randomized Controlled Trial; Research Support, Non-U.S. Gov't
DOI: http://dx.doi.org/10.1111/j.1365-2702.2011.03740.x
ISSN: 1365-2702
Place of Publication: England
PubMedID: 21615576
Accession Number: 21615576
Available at Journal of Clinical Nursing - from Wiley Online Library Medicine and Nursing Collection 2020
Abstract:AIM: The purpose of this study was (1) to evaluate the effectiveness of a pain education programme to increase the satisfaction of patients with cancer with regard to pain management and (2) to examine how patient satisfaction with pain management mediates the barriers to using analgesics and analgesic adherence.BACKGROUND: The patients' satisfaction with pain management is not merely an indicator, it is actually a contributor to medication adherence. However, very few studies investigate methods for improving patient satisfaction with pain management.DESIGN: This study used an experimental and longitudinal design.METHODS: A total of 61 patient-family pairs (n = 122) were randomly assigned to either experimental or control groups. The instruments included the American Pain Society outcome questionnaire, the Barriers Questionnaire-Taiwan form, self-reporting evaluations of analgesic adherence and the Pain Education Booklet. The experimental group (n = 31) participated in a pain education programme, while those in the control group (n = 30) did not. The two groups were compared using generalised estimation equations after the second and fourth weeks. A Sobel test was used to examine the mediating relationships among patient satisfaction with pain management, barriers to using analgesics and analgesic adherence.RESULTS: The experimental group showed a significant improvement in the level of satisfaction they felt for physicians and nurses regarding pain management. For those in the experimental group, satisfaction with pain management was a significant mediator between barriers to using analgesics and analgesic adherence.CONCLUSIONS: This research provides evidence supporting the effectiveness of a pain education programme for patients and their family members in increasing patient satisfaction with regard to the management of cancer pain.RELEVANCE TO CLINICAL PRACTICE: It is important for health providers to consider patient satisfaction when attempting to improve adherence to pain management regimes in a clinical setting.
Institutions:
(Chou PL) Graduate Institute of Nursing, College of Nursing, Taipei Medical University, Taipei, Taiwan.
Database: PubMed

163. Nursing's role in cancer pain management.

Author(s): Vallerand AH; Musto S; Polomano RC

Source: Current pain and headache reports; Aug 2011; vol. 15 (no. 4); p. 250-262

Publication Date: Aug 2011
Publication Type(s): Journal Article; Review
DOI: http://dx.doi.org/10.1007/s11916-011-0203-5
ISSN: 1534-3081
Place of Publication: United States
PubMedID: 21538044
Accession Number: 21538044

Available at Current Pain & Headache Reports - from EBSCO (MEDLINE Complete)

Available at Current Pain & Headache Reports - from ProQuest (Health Research Premium) - NHS Version

Abstract: Nurses have advanced practice, research, and education in the field of cancer pain management. This paper highlights the contributions nurses have made to pain science and practice through literature published in the past 3 years. Work accomplished by nurses is examined in the areas of pain assessment, pain management, intervention-based research, evidence-based practice, patient education, and palliative care. Nurses serve as advocates for empowering patients to engage in self-management of their pain, and offer education and support to patients and families at their most vulnerable times. Nurse researchers have been at the forefront of work to develop and test new instruments and approaches to measure pain, elucidate pain experiences through quantitative and qualitative methodologies, and gauge the quality of pain care for patients and its impact on their caregivers. This research has uncovered many patient, health care professional, and systemic barriers to effective pain control, and has offered feasible solutions to overcoming these barriers.

Institutions:
(Vallerand AH) Wayne State University College of Nursing, Detroit, MI 48202, USA. April.Vallerand@wayne.edu
Database: PubMed

164. Attitude and knowledge of physicians about cancer pain management: young doctors of South Korea in their early career.

Author(s): Kim MH; Park H; Park EC; Park K

Source: Japanese journal of clinical oncology; Jun 2011; vol. 41 (no. 6); p. 783-791
Publication Date: Jun 2011
Publication Type(s): Journal Article; Research Support, Non-U.S. Gov't
DOI: http://dx.doi.org/10.1093/jjco/hyr043
ISSN: 1465-3621
Place of Publication: England
PubMedID: 21502282
Accession Number: 21502282

Available at Japanese journal of clinical oncology - from HighWire - Free Full Text

Available at Japanese journal of clinical oncology - from Unpaywall

Abstract:OBJECTIVE: This study is aimed at evaluating the attitude and knowledge about the optimal use of opioids and finding out the barriers to cancer pain management especially for young doctors in South Korea.METHODS: A survey through questionnaire form was conducted on 1204 physicians. Physicians were grouped by their medical specialties and personal characteristics. Specialties were grouped into internal medicine and family medicine doctors, surgeons, anesthesiologists, pediatricians, other board holders and general physicians. Personal characteristics were grouped by their past experiences and current surroundings.RESULTS: Though many doctors thought that they were fairly well educated for pain management strategy, a large population of physicians showed a negative attitude and inadequate knowledge status about cancer pain management. The degree of attitude and knowledge status was different as their specialties and personal experiences. The factors that affected doctors' attitude and knowledge were: (i) medical specialty, (ii) past history of using practical pain assessment tool, (iii) self-perception of knowledge status about pain management, (iv) experience of prescribing opioids, (v) experience of education for cancer pain management. Although many physicians had a passive attitude in prescribing opioid analgesics, they are willingly open to use opioids for cancer pain management in the future. The most important perceived barriers to optimal cancer pain management were the fear for risk of tolerance, drug addiction, side effects of opioid analgesics and knowledge deficit about opioid analgesics.CONCLUSIONS: From this study, we found that further education and practical training will be needed for adequate cancer pain management for young physicians in their early career.

Institutions:
(Kim MH) Cancer Information and Education Branch, National Cancer Center, National Cancer Control Research Institute, 323 Ilsan ro, Ilsandong-gu, Goyang-Si, Gyeonggi-do 410-769, South Korea.
Database: PubMed

165. Meta-analysis of cultural differences in Western and Asian patient-perceived barriers to managing cancer pain.

Author(s): Chen CH; Tang ST

Source: Palliative medicine; Apr 2012; vol. 26 (no. 3); p. 206-221

Publication Date: Apr 2012

Publication Type(s): Journal Article; Meta-Analysis; Research Support, Non-U.S. Gov't

DOI: http://dx.doi.org/10.1177/0269216311402711

ISSN: 1477-030X

Place of Publication: England

PubMedID: 21474622

Accession Number: 21474622

Available at Palliative medicine - from ProQuest (MEDLINE with Full Text) - NHS Version

Available at Palliative medicine - from ProQuest (Health Research Premium) - NHS Version

Abstract:PURPOSE: barriers to managing cancer pain contribute to cancer patients' reluctance to report pain and use prescribed analgesics, resulting in inadequate pain control. Patients' perceived barriers to managing cancer pain may be influenced by culture. This meta-analysis compared differences in Western and Asian patient-perceived barriers to managing cancer pain.METHODS: the literature was systematically reviewed to compare pain barriers in Western and Asian cancer patients in 22 studies that used Ward's Barrier Questionnaire. Differences in weighted barrier scores were compared by meta-regression analysis.RESULTS: Asian cancer patients had higher barrier scores than Western patients, except for barriers of 'good patient', 'side effects', 'distract physician', 'fear of injections', and 'addiction'. Meta-regression analysis indicated that Asian patients' perceived pain barriers differed significantly from those of Western patients for disease progression (weighted mean difference [WMD] = 1.32; 95% confidence interval [CI] 0.80, 1.84, $p < 0.0001$), tolerance (WMD = 1.63; 95% CI 0.91, 2.36, $p < 0.0001$), fatalism (WMD = 0.89; 95% CI 0.28, 1.52, $p = 0.004$), and total score (WMD = 0.82; 95% CI 0.36, 1.28, $p < 0.0001$).CONCLUSION: Asian patients' perceived barriers to managing cancer pain were significantly higher than those for Western patients (especially for concerns about disease progression, tolerance, and fatalism). Asian cancer patients need to be assessed and carefully treated for perceived barriers to optimize cancer pain management.

Institutions:
(Chen CH) Chang Gung University, Graduate School of Nursing, Tao-Yuan, Taiwan, ROC.

Database: PubMed

166. Opioids for cancer pain in the Middle Eastern countries: a physician point of view.

Author(s): Daher M

Source: Journal of pediatric hematology/oncology; Apr 2011 ; p. S23

Publication Date: Apr 2011

Publication Type(s): Journal Article

DOI: http://dx.doi.org/10.1097/MPH.0b013e3182121a0f

ISSN: 1536-3678

Place of Publication: United States

PubMedID: 21448030

Accession Number: 21448030

Abstract: Cancer is an increasing problem in the Middle Eastern (ME) countries. It is the fourth leading cause of death in this region. At present, resources for cancer control in the ME countries as a whole are not only inadequate but directed almost exclusively to treatment. In the majority of countries of this region, cancer is generally diagnosed when it is at a relatively advanced stage. Pain is prevalent among people who have cancer, and is one of the most feared and burdensome symptoms. Pain negatively affects the quality of life of patients with cancer. Inadequate and inappropriate pain management of patients who experienced cancer pain has been documented in several studies and this is possibly due to insufficient understanding of pain assessment and management. Middle Eastern countries

include a wide range of economically diverse countries, from technically advanced countries with high level cancer care to countries with little or no cancer treatment capabilities. There are large differences in population size, wealth and health expenditure. Palliative care (PC) is an urgent humanitarian need worldwide for people with cancer and other chronic fatal diseases; relieving pain and suffering is an essential part of PC. The need for improved palliative care in ME countries is great. Of 58 million people who die every year, 45 million die in developing countries. An estimated 60% (27 million) of these people in developing countries would benefit from palliative care, and this number is growing as chronic diseases such as cancer rise rapidly. From the situation analysis of palliative care in the ME countries, suggesting that pain relief is insufficient, improvements in palliative care delivery are a high priority.We reviewed the situation of pain management and pain control in Lebanon and the ME countries, the barriers that are present, and we propose the priorities and a reform for an integrated approach to address the problem of under-treated pain at all levels:

Institutions:
(Daher M) MedicalEthics and Bioethics Teaching Program, University of Balamand Saint Georges Hospital, Beirut, Lebanon. mndaher@inco.com.lb
Database: PubMed

167. Urine drug testing in chronic pain.

Author(s): Christo PJ; Manchikanti L; Ruan X; Bottros M; Hansen H; Solanki DR; Jordan AE; Colson J

Source: Pain physician; 2011; vol. 14 (no. 2); p. 123-143

Publication Date: 2011

Publication Type(s): Journal Article; Review

ISSN: 2150-1149

Place of Publication: United States

PubMedID: 21412368

Accession Number: 21412368

Abstract: Therapeutic use, overuse, abuse, and diversion of controlled substances in managing chronic non-cancer pain continue to be an issue for physicians and patients. The challenge is to eliminate or significantly curtail abuse of controlled prescription drugs while still assuring the proper treatment of those patients. Some physicians are apprehensive regarding the use of chronic opioid therapy in chronic non-cancer pain due to a perceived lack of proven evidence, the misuse of opioids, tolerance, dependence, and hyperalgesia. However, others have criticized the underuse of opioids, resulting in the undertreatment of pain. It has been the convention that federal, state, and local governments; professional associations; as well as pharmaceutical companies, physicians, accrediting bodies, medical licensure boards, and the public all share responsibility for preventing abuse of controlled prescription drugs. To overcome the critical challenge of eliminating or significantly curtailing abuse of controlled prescription drugs and at the same time assuring the appropriate treatment for those patients who can be helped by these medications, it is crucial to practice adherence or compliance monitoring of opioid therapy. Compliance monitoring has been shown to be crucial in delivering proper opioid

therapy and preserving this therapy for the future. Urine drug testing (UDT) is considered one of the mainstays of adherence monitoring in conjunction with prescription monitoring programs and other screening tools, however, UDT is associated with multiple limitations secondary to potential pitfalls related to drug metabolism, reliability of the tests, and the knowledge of the pain physician. UDT is a widely available and familiar method for monitoring opioid use in chronic pain patients. UDT can provide tools for tracking patient compliance and expose possible drug misuse and abuse. UDT is one of the major tools of adherence monitoring in the assessment of the patient's predisposition to, and patterns of, drug misuse/abuse--a vital first step towards establishing and maintaining the safe and effective use of opioid analgesics in the treatment of chronic pain. This comprehensive review provides the role of UDT in monitoring chronic opioid therapy along with reliability and accuracy, appropriate use, overuse, misuse, and abuse.

Institutions:
(Christo PJ) Department of Anesthesiology and Critical Care Medicine Division of Pain Medicine, Johns Hopkins University School of Medicine, Baltimore, MD 21205, USA. pchristo@jhmi.edu
Database: PubMed

168. Using latent transition analysis in nursing research to explore change over time.

Author(s): Roberts TJ; Ward SE

Source: Nursing research; 2011; vol. 60 (no. 1); p. 73-79

Publication Date: 2011

Publication Type(s): Journal Article; Research Support, N.I.H., Extramural

DOI: http://dx.doi.org/10.1097/NNR.0b013e3182001c63

ISSN: 1538-9847

Place of Publication: United States

PubMedID: 21127448

Accession Number: 21127448

Available at Nursing research - from Unpaywall

Abstract:BACKGROUND: Latent transition analysis is a method of modeling change over time in categorical variables. It has been used in the social sciences for many years, but not in nursing research.OBJECTIVE: The purposes of this study were to illustrate the utility of latent transition analysis for nursing research by presenting a case example (a secondary analysis of data from a previously conducted randomized control trial testing the effectiveness of a tailored psychoeducational intervention to decrease patient-related attitudinal barriers to cancer pain management) and to understand for whom and in what direction the tailored intervention resulted in change with respect to attitudinal barriers and pain symptoms.METHODS: The model was developed by (a) defining a class structure on the basis of individuals' barrier patterns, (b) adding demographic predictors and distal pain outcomes, and (c) modeling and testing transitions across classes.RESULTS: There were two classes of individuals: Low Barriers and High Barriers. Older, less educated individuals were more likely to be in the High Barriers class at Time 1. Individuals in either class did not have different pain outcomes at the

end of the study. Of those individuals that transitioned across classes, those who received the intervention were statistically more likely to move in a favorable direction (to the Low Barriers class). Furthermore, there is evidence that some individuals in the control group had unfavorable outcomes.DISCUSSION: The results from the example provide useful information about for whom and in what direction the intervention resulted in change. Latent transition analysis is a valuable procedure for nurse researchers because it collapses large arrays of categorical data into meaningful patterns. It is a flexible modeling procedure with extensions allowing further understanding of a change process.

Institutions:
(Roberts TJ) Center for Patient-Centered Interventions, School of Nursing, University of Wisconsin-Madison, USA. tjbeal@wisc.edu
Database: PubMed

169. Cancer pain management in ambulatory care: can we link assessment and action to outcomes?

Author(s): Wells N; McDowell MR; Hendricks P; Dietrich MS; Murphy B

Source: Supportive care in cancer : official journal of the Multinational Association of Supportive Care in Cancer; Nov 2011; vol. 19 (no. 11); p. 1865-1871

Publication Date: Nov 2011

Publication Type(s): Journal Article; Multicenter Study; Research Support, N.I.H., Extramural; Research Support, Non-U.S. Gov't

DOI: http://dx.doi.org/10.1007/s00520-010-1030-7

ISSN: 1433-7339

Place of Publication: Germany

PubMedID: 21052733

Accession Number: 21052733

Available at Supportive Care in Cancer - from SpringerLink

Available at Supportive Care in Cancer - from EBSCO (MEDLINE Complete)

Available at Supportive Care in Cancer - from EBSCO (CINAHL Complete)

Available at Supportive Care in Cancer - from ProQuest (MEDLINE with Full Text) - NHS Version

Available at Supportive Care in Cancer - from ProQuest (Health Research Premium) - NHS Version

Available at Supportive Care in Cancer - from David Adams Library Journals Collection Local Print Collection [location] : David Adams Library.

Available at Supportive Care in Cancer - from Unpaywall

Abstract:PURPOSE: Good cancer pain control requires appropriate assessment and treatment. The purpose of this study was to examine the relationships among physician, nurse practitioner, and nurse knowledge, documentation of assessment, treatment, and pain reduction in cancer patients seen in ambulatory settings.METHOD: The study method included an assessment of pain knowledge of providers (physicians, nurse practitioners, and nurses) who worked in cancer clinics and a retrospective review of patients' records treated for cancer-related pain in their

clinics. Fifty-eight providers from eight cancer clinics completed the knowledge questionnaire; 56 patient records were reviewed for assessment, treatment, and outcome data. Pain relief, the outcome, was obtained from documentation at the next clinic visit.RESULTS: Of the 54 patient records that documented pain relief at the next clinic visit, 61.9% reported no relief. Chi square analysis revealed clinics with a higher level of pain knowledge documented a greater number of elements of an ideal pain assessment (p = 0.03) but was unrelated to treatment and pain relief reported. Assessment and treatment were unrelated to reported pain relief at the next clinic visit.CONCLUSION: These data suggest that providers' pain knowledge is related to pain assessment but not treatment or outcome. In addition, these data showed no relationship between assessment, treatment prescribed, and pain relief in these ambulatory settings.
Institutions:
(Wells N) Department of Nursing, Vanderbilt University Medical Center, Nashville, TN, USA. nancy.wells@vanderbilt.edu
Database: PubMed

170. The DSCP-CA: a decision support computer program--cancer pain management.

Author(s): Im EO; Chee W

Source: Computers, informatics, nursing : CIN; May 2011; vol. 29 (no. 5); p. 289-296

Publication Date: May 2011

Publication Type(s): Journal Article; Research Support, N.I.H., Extramural

DOI: http://dx.doi.org/10.1097/NCN.0b013e3181f9dd23

ISSN: 1538-9774

Place of Publication: United States

PubMedID: 20975538

Accession Number: 20975538

Abstract: The purpose of this study was to develop a decision support system using fuzzy logic that would support nurses' decisions about cancer pain management, especially for ethnic minority cancer patients. The study had two phases: (1) data collection and (2) development of the decision support computer program. In the data collection phase, an Internet survey of 428 cancer patients and four ethnic-specific online forums (about 30 participants per forum) were conducted to gather data on the cancer pain experience of the four major ethnic groups in the United States. The development phase included two components: (1) development of three modules, including a knowledge base module, a decision module, and a self-adaptation module; and (2) a 3-month evaluation of the decision support computer program by oncology nurses and subsequent incorporation of their feedback into the program. The Internet survey and online forum data were processed into fuzzy and crisp data sets, and ethnic-specific algorithms for the decision module were developed. Using the self-adaptation module, the decision support computer program was further refined as additional data were processed. Then, the decision support computer program was further developed by adding additional components suggested by the oncology nurses.

Institutions:

(Im EO) School of Nursing, The University of Texas at Austin, 1700 Red River, Austin, TX 78701, USA. eim@mail.nur.utexas.edu
Database: PubMed

171. Effect of certification in oncology nursing on nursing-sensitive outcomes.

Author(s): Coleman EA; Coon SK; Lockhart K; Kennedy RL; Montgomery R; Copeland N; McNatt P; Savell S; Stewart C

Source: The Journal of nursing administration; Oct 2010; vol. 40 (no. 10 Suppl); p. S35

Publication Date: Oct 2010

Publication Type(s): Journal Article

DOI: http://dx.doi.org/10.1097/NNA.0b013e3181f37f9f

ISSN: 1539-0721

Place of Publication: United States

PubMedID: 20859100

Accession Number: 20859100

Abstract: The study compared certified nurses with noncertified nurses for symptom management of nausea, vomiting, and pain; patient satisfaction; and nurse satisfaction to determine the effect of certification in oncology nursing on those nursing-sensitive outcomes. A total of 93 nurses-35 (38%) of them certified in oncology nursing-and 270 patients completed surveys. Chart audits provided additional data on symptom management. Certified nurses scored higher than noncertified nurses on the Nurses' Knowledge and Attitudes Survey Regarding Pain as well as the Nausea Management: Nurses' Knowledge and Attitudes Survey. The chart audits showed that certified nurses followed National Comprehensive Cancer Network guidelines for chemotherapy-induced nausea and vomiting (CINV) management more often than noncertified nurses. The study demonstrated that job satisfaction is fairly high for oncology nurses and patient satisfaction is high. In general, cancer pain and CINV were managed well but improvements can be made. Nurses and physicians continuously should be educated on evidence-based guidelines for symptom management of cancer pain and CINV, and a CINV knowledge and attitude assessment tool should be developed.

Institutions:
(Coleman EA) College of Nursing at the University of Arkansas for Medical Sciences in Little Rock, USA. colemanann@uams.edu
Database: PubMed

172. Rational use and effectiveness of morphine in the palliative care of cancer patients at the Ocean Road Cancer Institute in Dar es Salaam, Tanzania.

Author(s): Kamuhabwa A; Ezekiel D

Source: Tanzania journal of health research; Oct 2009; vol. 11 (no. 4); p. 170-174

Publication Date: Oct 2009

Publication Type(s): Journal Article

DOI: http://dx.doi.org/10.4314/thrb.v11i4.50147

ISSN: 1821-6404

Place of Publication: Tanzania

PubMedID: 20734695

Accession Number: 20734695

Available at Tanzania journal of health research - from EBSCO (MEDLINE Complete)

Available at Tanzania journal of health research - from Unpaywall

Abstract: Morphine and other opioids is the mainstay of cancer pain management. However, considerable fears surrounding their use present barriers to pain control. The aim of this study was to assess the rational use and effectiveness of morphine for management of pain in the palliative care of cancer patients at Ocean Road Cancer Institute (ORCI) in Tanzania. A total of 100 cancer patients who were receiving morphine therapy at the ORCI were interviewed to get information on morphine use. In addition, information on the prescribed doses of morphine was obtained from medical records of 200 patients who have used morphine from September 2005 to April 2006. Both outpatients and inpatients with advanced cancer who were receiving morphine for palliative care were involved. Seven (7) palliative caregivers, including two doctors, two nurses, a pharmacist, a pharmaceutical technician and a social worker were also interviewed. Of the 100 interviewees, 37% were aware of morphine. The level of education and duration of therapy had an impact on the awareness. The results also showed that oral morphine solution was the most common route (96%) of administration. Fifty-seven percent of the patients described the doses of morphine given to be effective in relieving their pain. Although most patients (79%) experienced morphine-induced side effects, the majority (93%) were continuing with the therapy. There were no indication of irrational use of morphine and morphine-induced side effects were well managed. The majority of patients and caregivers had positive attitude towards the use of morphine. In conclusion, the study revealed that the use of morphine is acceptable among a large proportion of patients receiving palliative care and that the majority of them find the doses given effective to relieve their pain.

Institutions:
(Kamuhabwa A) Unit of Pharmacology and Therapeutics, School of Pharmacy, Muhimbili University of Health and Allied Sciences, P.O. Box 65013, Dar es Salaam, Tanzania. akamuhabwa@muhas.ac.tz

Database: PubMed

173. Types and epidemiology of cancer-related neuropathic pain: the intersection of cancer pain and neuropathic pain.

Author(s): Lema MJ; Foley KM; Hausheer FH

Source: The oncologist; 2010 ; p. 3-8

Publication Date: 2010

Publication Type(s): Journal Article; Review

DOI: http://dx.doi.org/10.1634/theoncologist.2009-S505

ISSN: 1549-490X

Place of Publication: United States

PubMedID: 20489190

Accession Number: 20489190

Available at The oncologist - from Europe PubMed Central - Open Access

Available at The oncologist - from HighWire - Free Full Text

Available at The oncologist - from EBSCO (MEDLINE Complete)

Abstract: Neuropathic pain--pain resulting from a lesion, damage, or dysfunction of the somatosensory nervous system--can arise through several distinct etiologies ranging from toxicity, surgery, radiation, and trauma to congenital disorders. Neuropathic pain is widely recognized as a common consequence of cancer and results from administration of several common oncology drugs. It not only impacts quality of life, but it also impacts patient outcomes because of resulting treatment delays, dose reductions, and discontinuations. We estimate that the cost of the problem in the U.S. alone is approximately $2.3 billion. Despite its widely recognized importance, there is a paucity of reliable information available regarding the incidence, prevalence of patient-and physician-reported severity, and time course of cancer-related neuropathic pain. To address this severe knowledge gap, we need new, high-quality, population-based studies of individual cancer pain syndromes and conditions. However, in order to gather this information, we also need substantial improvements in the specific classification of cancer-related neuropathic syndromes and better validated diagnostic tools that can help to elucidate the incidence, prevalence, severity, and potential economic impact of cancer-associated neuropathies.

Institutions:
(Lema MJ) Roswell Park Cancer Institute, Buffalo, New York, USA.
Database: PubMed

174. Psychological and behavioural predictors of pain management outcomes in patients with cancer.

Author(s): Jacobsen R; Møldrup C; Christrup L; Sjøgren P; Hansen OB

Source: Scandinavian journal of caring sciences; Dec 2010; vol. 24 (no. 4); p. 781-790

Publication Date: Dec 2010

Publication Type(s): Journal Article; Research Support, Non-U.S. Gov't

DOI: http://dx.doi.org/10.1111/j.1471-6712.2010.00776.x

ISSN: 1471-6712

Place of Publication: Sweden

PubMedID: 20487402

Accession Number: 20487402

Available at Scandinavian Journal of Caring Sciences - from Wiley Online Library Medicine and Nursing Collection 2020

Available at Scandinavian Journal of Caring Sciences - from EBSCO (MEDLINE Complete)

Available at Scandinavian Journal of Caring Sciences - from EBSCO (CINAHL Complete)

Available at Scandinavian Journal of Caring Sciences - from EBSCO (Psychology and Behavioral Sciences Collection)

Abstract: To better understand the phenomenon of patient-related barriers to cancer pain management and address them more effectively in interventional studies, a theoretical model related to psychological aspects of pain experience and pain-related behaviours was elaborated. The aim of the study was to analyse the impact of patient-related barriers on cancer pain management outcomes following this model. Thirty-three patients responded to the Brief Pain Inventory Pain scale, the Danish Barriers Questionnaire II (DBQ-II), the Hospital Anxiety and Depression scale (HADS), the Danish version of Patient Perceived Involvement in Care Scale measuring the quality of patient-physician pain communication, and the Danish version of Medication Adherence Report Scale (DMARS-4). Statistical analysis was performed with SPSS 16.00. The results of the multivariable linear regression analyses showed that pain intensity was explained by patients' emotional distress (symptoms of anxiety and depression) and that pain relief was explained by cognitive barriers. In conclusion, interventions in emotional distress and patients' concerns may supposedly result in better cancer pain management outcomes.

Institutions:
(Jacobsen R) Department of Pharmacology and Pharmacotherapy, The Faculty of Pharmaceutical Sciences, University of Copenhagen, Universitetsparken, Copenhagen, Denmark. raj@farma.ku.dk
Database: PubMed

175. Review of the effect of opioid-related side effects on the undertreatment of moderate to severe chronic non-cancer pain: tapentadol, a step toward a solution?

Author(s): Candiotti KA; Gitlin MC

Source: Current medical research and opinion; Jul 2010; vol. 26 (no. 7); p. 1677-1684

Publication Date: Jul 2010

Publication Type(s): Evaluation Study; Journal Article; Research Support, Non-U.S. Gov't; Review

DOI: http://dx.doi.org/10.1185/03007995.2010.483941

ISSN: 1473-4877

Place of Publication: England

PubMedID: 20465361

Accession Number: 20465361

Abstract:OBJECTIVE: Opioids are among the most effective and potent analgesics currently available. Their utility in the management of pain associated with cancer, acute injury, or surgery is well recognized. However, extending the application of opioids to the management of chronic non-cancer pain has met with considerable resistance. This resistance is due in part to concerns related to gastrointestinal and central nervous system-related adverse events as well as issues pertaining to

regulatory affairs, the development of tolerance, incorrect drug usage, and addiction. This review focuses on the incidence of opioid-related side effects and the patient and physician barriers to opioid therapy for chronic non-cancer pain. Tapentadol, a centrally acting analgesic with two mechanisms of action, micro-opioid agonism and norepinephrine reuptake inhibition, may be considered to be a partial solution to some of these issues.METHODS: MEDLINE was searched for English-language articles from 1950 to February 2010 using the terms chronic non-cancer pain and opioids together and in combination with undertreatment, adherence, and compliance.RESULTS: The majority of patients treated with traditional opioids experience gastrointestinal- or central nervous system-related adverse events, most commonly constipation, nausea, and somnolence. These side effects often lead to discontinuation of opioid therapy. Concerns about side effects, analgesic tolerance, dependence, and addiction limit the use of opioids for the management of chronic pain. Treatment with tapentadol appears to provide several advantages of an analgesic with a more favorable side-effect profile than the classic micro-opioid receptor agonist oxycodone (especially related to gastrointestinal tolerability).CONCLUSIONS: The pervasiveness of opioid-associated side effects and concerns related to tolerance, dependence, and addiction present potential barriers to the approval and use of opioids for the management of chronic non-cancer pain. The lower incidence of opioid-associated adverse events and possibly fewer withdrawal symptoms, combined with a satisfactory analgesic profile associated with tapentadol, suggest its potential utility for the management of chronic non-cancer pain. This review will focus on the incidence of opioid-related side effects and barriers to opioid therapy that are available as English-language articles in the MEDLINE index, and as such, it is a representative but not an exhaustive review of the current literature.

Institutions:
(Candiotti KA) Department of Anesthesiology, Perioperative Medicine and Pain Management, University of Miami Miller School of Medicine, Miami, FL 33101-6370, USA. kcandiotti@miami.edu
Database: PubMed

176. Using leadership and advocacy to improve cancer pain management--based on a presentation at the cancer pain, suffering and spirituality course.

Author(s): Nevidjon B

Source: Asian Pacific journal of cancer prevention : APJCP; 2010 ; p. 13-16

Publication Date: 2010

Publication Type(s): Journal Article; Review

ISSN: 2476-762X

Place of Publication: Thailand

PubMedID: 20590342

Accession Number: 20590342

Abstract: Being a leader is not dependent on a title and, in fact, every oncology nurse is a clinical leader. Building on skills in caring for patients, oncology clinicians and nurses use their knowledge and skills to: advocate for patients; initiate performance improvement projects; develop new services for patients and families;

ensure quality and safety of care; influence health policy. Because of oncology clinicians and nurses closeness to patients and families in all settings, they know where the barriers to excellence are in organizations. This session will provide an overview of leadership skills and how nurses in particular can use their expertise as clinicians to improve the care delivery in their organizations and communities.

Institutions:
(Nevidjon B) Oncology Nursing Society, Pittsburgh, PA, USA.
brenda.nevidjon@duke.edu
Database: PubMed

177. Improvement of pain related self management for oncologic patients through a trans institutional modular nursing intervention: protocol of a cluster randomized multicenter trial.

Author(s): Jahn P; Kitzmantel M; Renz P; Kukk E; Kuss O; Thoke-Colberg A; Horn I; Landenberger M

Source: Trials; Mar 2010; vol. 11 ; p. 29

Publication Date: Mar 2010

Publication Type(s): Journal Article; Multicenter Study; Randomized Controlled Trial; Research Support, Non-U.S. Gov't

DOI: http://dx.doi.org/10.1186/1745-6215-11-29

ISSN: 1745-6215

Place of Publication: England

PubMedID: 20307262

Accession Number: 20307262

Available at Trials - from BioMed Central

Available at Trials - from Europe PubMed Central - Open Access

Available at Trials - from EBSCO (MEDLINE Complete)

Available at Trials - from Unpaywall

Abstract:BACKGROUND: Pain is one of the most frequent and distressing symptoms in cancer patients. For the majority of the patients, sufficient pain relief can be obtained if adequate treatment is provided. However, pain remains often undertreated due to institutional, health care professional and patient related barriers. Patients self management skills are affected by the patients' knowledge, activities and attitude to pain management. This trial protocol is aimed to test the SCION-PAIN program, a multi modular structured intervention to improve self management in cancer patients with pain.METHODS: 240 patients with diagnosed malignancy and pain > 3 days and average pain >or= 3/10 will participate in a cluster randomized trial on 18 wards in 2 German university hospitals. Patients from the intervention wards will receive, additionally to standard pain treatment, the SCION-PAIN program consisting of 3 modules: pharmacologic pain management, nonpharmacologic pain management and discharge management. The intervention will be conducted by specially trained oncology nurses and includes components of patient education, skills training and counseling to improve self care regarding pain management beginning with admission followed by booster session every 3rd day and one follow up telephone counseling within 2 to 3 days after discharge. Patients

in the control group will receive standard care. Primary endpoint is the group difference in patient related barriers to management of cancer pain (BQII), 7 days after discharge. Secondary endpoints are: pain intensity & interference, adherence, coping and HRQoL.DISCUSSION: The study will determine if the acquired self management skills of the patients continue to be used after discharge from hospital. It is hypothesized that patients who receive the multi modular structured intervention will have less patient related barriers and a better self management of cancer pain.TRIAL REGISTRATION: ClinicalTrials NCT00779597.

Institutions:
(Jahn P) Institute for Health and Nursing Science, Medical Faculty, Martin-Luther-University Halle-Wittenberg, Germany. patrick.jahn@medizin.uni-halle.de

Database: PubMed

178. A survey on physician knowledge and attitudes towards clinical use of morphine for cancer pain treatment in China.

Author(s): Yanjun S; Changli W; Ling W; Woo JC; Sabrina K; Chang L; Lei Z

Source: Supportive care in cancer : official journal of the Multinational Association of Supportive Care in Cancer; Nov 2010; vol. 18 (no. 11); p. 1455-1460

Publication Date: Nov 2010

Publication Type(s): Journal Article

DOI: http://dx.doi.org/10.1007/s00520-009-0768-2

ISSN: 1433-7339

Place of Publication: Germany

PubMedID: 19902274

Accession Number: 19902274

Available at Supportive care in cancer : official journal of the Multinational Association of Supportive Care in Cancer - from SpringerLink

Available at Supportive care in cancer : official journal of the Multinational Association of Supportive Care in Cancer - from EBSCO (MEDLINE Complete)

Available at Supportive care in cancer : official journal of the Multinational Association of Supportive Care in Cancer - from EBSCO (CINAHL Complete)

Available at Supportive care in cancer : official journal of the Multinational Association of Supportive Care in Cancer - from ProQuest (MEDLINE with Full Text) - NHS Version

Available at Supportive care in cancer : official journal of the Multinational Association of Supportive Care in Cancer - from ProQuest (Health Research Premium) - NHS Version

Available at Supportive care in cancer : official journal of the Multinational Association of Supportive Care in Cancer - from David Adams Library Journals Collection Local Print Collection [location] : David Adams Library.

Abstract:BACKGROUND: The WHO's three-step guideline for cancer pain management has been introduced in China; however, there remain large differences in the standards of cancer pain management between China and other developed countries. This survey was carried out to determine the degree of physician

knowledge on morphine use and the factors that impede morphine use in clinical practice in China.METHODS: A self-reported questionnaire was designed and administered to randomly selected physicians in four tertiary hospitals in the cities of Changchun and Changsha in China. Statistical analyses were conducted using SPSS statistical software.RESULTS: Two hundred and one clinical physicians participated in the survey. Physicians who reported having received training in cancer pain management and drug use demonstrated a significantly higher mean score of basic knowledge compared to physicians who reported not having received training (9.31 ± 2.88:8.23 ± 2.70, u = 2.74, p < 0.001). The top three cited impediments to widespread clinical use of morphine for cancer pain were: (1) lack of professional knowledge and training; (2) fear of opioid addiction; and (3) physicians' personal preferences to select other drugs.CONCLUSIONS: Medical staffs lack the basic knowledge and harbor misconceptions about the clinical use of morphine for cancer pain treatment. Creating training opportunities for medical staffs is necessary to increase their awareness and knowledge of effective cancer pain management.

Institutions:
(Yanjun S) Department of Thoracic Surgery, Tianjin Lung Cancer Center, Tianjin Medical University Cancer Institute and Hospital, Tianjin 300060, People's Republic of China.

Database: PubMed

179. Guilty until proven innocent: a qualitative study of the management of chronic non-cancer pain among patients with a history of substance abuse.

Author(s): Baldacchino A; Gilchrist G; Fleming R; Bannister J

Source: Addictive behaviors; Mar 2010; vol. 35 (no. 3); p. 270-272

Publication Date: Mar 2010

Publication Type(s): Journal Article; Research Support, Non-U.S. Gov't

DOI: http://dx.doi.org/10.1016/j.addbeh.2009.10.008

ISSN: 1873-6327

Place of Publication: England

PubMedID: 19897313

Accession Number: 19897313

Abstract:INTRODUCTION: Physicians are often reluctant to prescribe strong opioids for chronic non cancer pain (CNCP). No study has qualitatively examined physicians' beliefs about prescribing opioids for CNCP to patients with a history of substance abuse (PWHSA).AIMS: To describe physicians' attitudes and experience of prescribing opioids for CNCP to PWHSA.DESIGN, SETTING AND PARTICIPANTS: Nineteen individual interviews and two focus groups were conducted with GPs, Addiction Specialists, Pain Specialists and Rheumatologists.RESULTS: Physicians were "reluctant" to prescribe opioids to PWHSA experiencing CNCP for fear of addiction, misuse or diversion of medications. Many exhibited "distrust" that such patients were experiencing "genuine pain", resulting in patients often being considered guilty until proven innocent. Such negative regard towards these patients was based on previous manipulative "drug seeking" encounters and often resulted in the under treatment of pain. Potential "flags" were identified that alerted physicians to the potential for abuse or diversion

of their prescription including: doctor shopping, loosing prescriptions, frequent attendance and early requests for repeat prescriptions. Physicians reported different management approaches and stricter prescribing regimes for PWHSA to limit the potential of addiction, misuse and diversion. Examples of poor pain management were described where drug users had been under treated as a result of negative attitudes or inexperience of staff.DISCUSSION: Applying the chronic disease model to comorbid addiction and CNCP would ensure a health and social care system that makes it difficult to stigmatise patients experiencing these conditions and would facilitate the prescribing of opioid pain medication to patients who could benefit.

Institutions:
(Baldacchino A) Centre for Addiction Research and Education Scotland (CARES), The University of Dundee, Ninewells Hospital & Medical School, Dundee, DD1 9SY, Scotland.

Database: PubMed

180. [Attitude survey of medical staff on the participation of community pharmacists in palliative home care].

Author(s): Akai N; Fujita-Hamabe W; Tokuyama S

Source: Yakugaku zasshi : Journal of the Pharmaceutical Society of Japan; Nov 2009; vol. 129 (no. 11); p. 1393-1401

Publication Date: Nov 2009

Publication Type(s): English Abstract; Journal Article

DOI: http://dx.doi.org/10.1248/yakushi.129.1393

ISSN: 0031-6903

Place of Publication: Japan

PubMedID: 19881212

Accession Number: 19881212

Available at Yakugaku zasshi : Journal of the Pharmaceutical Society of Japan - from Unpaywall

Abstract: The treatment of cancer pain requires an individually-targeted multidimensional team approach. Further, the basic act for the Anti-Cancer Measures describes that medical staff including pharmacists should participate in the palliative care. Thus it is obvious that community pharmacists should also participate in palliative home care. In addition, a misunderstanding about opioids remains strong in Japan, which could be one of the barriers to palliative home care. In our previous report, we clarified for local residents the importance of educational activity using opioids as a new role for community pharmacists, and it was recognized of great significance by these pharmacists. In this study, we conducted a questionnaire survey among medical doctors, nurses, care managers and home helpers about the need and meaning of the educational activity performed by pharmacists in palliative home care. 86.4% of respondents felt pharmacists' participation in home care was required. Furthermore, most respondents thought that misunderstanding remained about opioids in palliative home care, and believed that pharmacists could play an important role in educational activity about opioids as experts in medicine. This study clarified that other medical team members need the participation of community

pharmacists in palliative home care. Therefore, it seems important for these pharmacists to be proactive in participating in such care in the future.

Institutions:
(Akai N) Department of Clinical Pharmacy, Faculty of Pharmaceutical Sciences, Kobe Gakuin University, Chuo-ku, Kobe, Japan.
Database: PubMed

181. Barriers to cancer pain management: a review of empirical research.

Author(s): Jacobsen R; Liubarskiene Z; Møldrup C; Christrup L; Sjøgren P; Samsanaviciene J

Source: Medicina (Kaunas, Lithuania); 2009; vol. 45 (no. 6); p. 427-433

Publication Date: 2009

Publication Type(s): Journal Article; Review

ISSN: 1648-9144

Place of Publication: Switzerland

PubMedID: 19605961

Accession Number: 19605961

Available at Medicina (Kaunas, Lithuania) - from Unpaywall

Abstract: Patient-, physician-, and health care system-related barriers of cancer pain management in patients with malignant diseases are a recognized and widely investigated issue. The purpose of this review is to summarize the main findings of empirical research on these barriers in the literature. The most significant patient-related barriers were patient reluctance to report pain and adhere to treatment recommendations. Besides that, cognitive, affective, and sensory patient-related barriers to cancer pain management with opioid analgesics have been studied using quantitative and qualitative research methods. The Barriers Questionnaire and its shortened and modified versions were the most commonly used instruments in the context of research on patient-related barriers to cancer pain management. The most prominent physician-related barriers were insufficient physicians' knowledge about cancer pain management, inadequate patterns of pain assessment, and inadequate opioid prescription. The methodologies used to conduct the majority of the studies on physician-related barriers were weak. Nevertheless, physician knowledge of pain management guidelines, the quality of pain assessment and opioid prescription have been shown to be obviously better in a few Western countries. Institutional and health care system-related barriers were relevant only in countries with restrictive opioid prescription regulations. The evaluation of the influence of cultural-social-economical background on cancer pain management could probably help to obtain better insight into the problems of unrelieved cancer pain.

Institutions:
(Jacobsen R) Department of Pharmacology and Pharmacotherapy, Faculty of Pharmaceutical Sciences, University of Copenhagen, Denmark.
Database: PubMed

182. The Danish Barriers Questionnaire-II: preliminary validation in cancer pain patients.

Author(s): Jacobsen R; Møldrup C; Christrup L; Sjøgren P; Hansen OB

Source: Pain practice : the official journal of World Institute of Pain; 2009; vol. 9 (no. 4); p. 266-274

Publication Date: 2009

Publication Type(s): Journal Article; Multicenter Study; Research Support, Non-U.S. Gov't; Validation Study

DOI: http://dx.doi.org/10.1111/j.1533-2500.2009.00296.x

ISSN: 1533-2500

Place of Publication: United States

PubMedID: 19549059

Accession Number: 19549059

Available at Pain practice : the official journal of World Institute of Pain - from Wiley Online Library Medicine and Nursing Collection 2020

Available at Pain practice : the official journal of World Institute of Pain - from EBSCO (CINAHL Complete)

Available at Pain practice : the official journal of World Institute of Pain - from EBSCO (Psychology and Behavioral Sciences Collection)

Abstract:OBJECTIVE: The objective of this study was to examine the psychometric properties of the Danish version of the Barriers Questionnaire-II (DBQ-II).METHODS: The validated Norwegian version of the DBQ-II was translated into Danish. Cancer patients for the study were recruited from specialized pain management facilities. Thirty-three patients responded to the DBQ-II, Hospital Anxiety and Depression Scale, and Brief Pain Inventory pain severity scale.RESULTS: A factor analysis of the DBQ-II resulted in six scales. Scale one, Fatalism, consisted of three items addressing fatalistic beliefs regarding cancer pain management. Scale two, Immune System, consisted of three items addressing the belief that pain medications harm the immune system. Scale three, Monitor, consisted of three items addressing the fear that pain medicine masks changes in one's body. Scale four, Communication, consisted of five items addressing the concern that reports of pain distract the physician from treating the cancer, and the belief that "good" patients do not complain. Scale five, Addiction, consisted of two items addressing the fear of becoming addicted to pain medication. Finally, scale six, Tolerance, consisted of three items addressing the fear of getting tolerant to analgesic effect of pain medicine. Items related to medication side effects were analyzed as separate units. The DBQ-II total had an internal consistency of 0.87. The DBQ-II total score was related to measures of pain relief and anxiety.CONCLUSIONS: The DBQ-II seems to be a reliable and valid measure of the barriers to pain management among Danish cancer patients.

Institutions:
(Jacobsen R) Section for Social Pharmacy, Department of Pharmacology and Pharmacotherapy, Faculty of Pharmaceutical Sciences, University of Copenhagen, Copenhagen, Denmark. raj@farma.ku.dk

Database: PubMed

183. Effect of certification in oncology nursing on nursing-sensitive outcomes.

Author(s): Coleman EA; Coon SK; Lockhart K; Kennedy RL; Montgomery R; Copeland N; McNatt P; Savell S; Stewart C

Source: Clinical journal of oncology nursing; Apr 2009; vol. 13 (no. 2); p. 165-172

Publication Date: Apr 2009

Publication Type(s): Journal Article

DOI: http://dx.doi.org/10.1188/09.CJON.165-172

ISSN: 1538-067X

Place of Publication: United States

PubMedID: 19349263

Accession Number: 19349263

Available at Clinical journal of oncology nursing - from Ovid (Journals @ Ovid)

Available at Clinical journal of oncology nursing - from EBSCO (CINAHL Complete)

Available at Clinical journal of oncology nursing - from ProQuest (Health Research Premium) - NHS Version

Available at Clinical journal of oncology nursing - from ProQuest (MEDLINE with Full Text) - NHS Version

Available at Clinical journal of oncology nursing - from David Adams Library Journals Collection Local Print Collection [location] : David Adams Library.

Abstract: The study compared certified nurses with noncertified nurses for symptom management of nausea, vomiting, and pain; patient satisfaction; and nurse satisfaction to determine the effect of certification in oncology nursing on those nursing-sensitive outcomes. A total of 93 nurses--35 (38%) of them certified in oncology nursing--and 270 patients completed surveys. Chart audits provided additional data on symptom management. Certified nurses scored higher than noncertified nurses on the Nurses' Knowledge and Attitudes Survey Regarding Pain as well as the Nausea Management: Nurses' Knowledge and Attitudes Survey. The chart audits showed that certified nurses followed National Comprehensive Cancer Network guidelines for chemotherapy-induced nausea and vomiting (CINV) management more often than noncertified nurses. The study demonstrated that job satisfaction is fairly high for oncology nurses and patient satisfaction is high. In general, cancer pain and CINV were managed well but improvements can be made. Nurses and physicians continuously should be educated on evidence-based guidelines for symptom management of cancer pain and CINV, and a CINV knowledge and attitude assessment tool should be developed.

Institutions:
(Coleman EA) The College of Nursing, The University of Arkansas for Medical Sciences, Little Rock, Arkansas, USA. colemanann@uams.edu
Database: PubMed

184. Pediatric palliative care: use of opioids for the management of pain.

Author(s): Zernikow B; Michel E; Craig F; Anderson BJ

Source: Paediatric drugs; 2009; vol. 11 (no. 2); p. 129-151

Publication Date: 2009

Publication Type(s): Journal Article; Review
DOI: http://dx.doi.org/10.2165/00148581-200911020-00004
ISSN: 1174-5878
Place of Publication: Switzerland
PubMedID: 19301934
Accession Number: 19301934
Available at Paediatric drugs - from EBSCO (MEDLINE Complete)
Available at Paediatric drugs - from ProQuest (MEDLINE with Full Text) - NHS Version
Available at Paediatric drugs - from ProQuest (Health Research Premium) - NHS Version

Abstract: Pediatric palliative care (PPC) is provided to children experiencing life-limiting diseases (LLD) or life-threatening diseases (LTD). Sixty to 90% of children with LLD/LTD undergoing PPC receive opioids at the end of life. Analgesia is often insufficient. Reasons include a lack of knowledge concerning opioid prescribing and adjustment of opioid dose to changing requirements. The choice of first-line opioid is based on scientific evidence, pain pathophysiology, and available administration modes. Doses are calculated on a bodyweight basis up to a maximum absolute starting dose. Morphine remains the gold standard starting opioid in PPC. Long-term opioid choice and dose administration is determined by the pathology, analgesic effectiveness, and adverse effect profile. Slow-release oral morphine remains the dominant formulation for long-term use in PPC with hydromorphone slow-release preparations being the first rotation opioid when morphine shows severe adverse effects. The recently introduced fentanyl transdermal therapeutic system with a drug-release rate of 12.5 microg/hour matches the lower dose requirements of pediatric cancer pain control. Its use may be associated with less constipation compared with morphine use. Though oral transmucosal fentanyl citrate has reduced bioavailability (25%), it inherits potential for breakthrough pain management. However, the gold standard breakthrough opioid remains immediate-release morphine. Buprenorphine is of special clinical interest as a result of its different administration routes, long duration of action, and metabolism largely independent of renal function. Antihyperalgesic effects, induced through antagonism at the kappa-receptor, may contribute to its effectiveness in neuropathic pain. Methadone also has a long elimination half-life (19 [SD 14] hours) and NMDA receptor activity although dose administration is complicated by highly variable morphine equianalgesic equivalence (1 : 2.5-20). Opioid rotation to methadone requires special protocols that take this into account. Strategies to minimize adverse effects of long-term opioid treatment include dose reduction, symptomatic therapy, opioid rotation, and administration route change. Patient- or nurse-controlled analgesia devices are useful when pain is rapidly changing, or in terminal care where analgesic requirements may escalate. In this article, we present detailed pediatric pharmacokinetic and pharmacodynamic data for opioids, their indications and contraindications, as well as dose-administration regimens that include practical strategies for opioid switching and dose reduction. Additionally, we discuss the problem of hyperalgesia and the use of adjuvant drugs to support opioid therapy.

Institutions:

(Zernikow B) Children's Hospital, Witten/Herdecke University, Vodafone Foundation Institute for Children's Pain Therapy and Paediatric Palliative Care, Datteln, Germany. B.Zernikow@Kinderklinik-Datteln.de
Database: PubMed

185. Nursing pain management--a qualitative interview study of patients with pain, hospitalized for cancer treatment.

Author(s): Rustøen T; Gaardsrud T; Leegaard M; Wahl AK

Source: Pain management nursing : official journal of the American Society of Pain Management Nurses; Mar 2009; vol. 10 (no. 1); p. 48-55

Publication Date: Mar 2009

Publication Type(s): Journal Article

DOI: http://dx.doi.org/10.1016/j.pmn.2008.09.003

ISSN: 1532-8635

Place of Publication: United States

PubMedID: 19264283

Accession Number: 19264283

Abstract: Pain is a significant symptom in cancer patients. Understanding of patients' experiences in relation to pain management is important in evidence-based nursing in the field of pain. The aim of this study was to explore cancer patients' experiences of nursing pain management during hospitalization for cancer treatment. Eighteen cancer patients participated in the study, all with advanced cancer, including skeleton metastases. The female participants all had breast cancer, and the male participants all had prostate cancer. Data were collected by in-depth interviews, and qualitative description was used to entail low-inference interpretation to reach an understanding of the essence of pain and nursing pain management. Patients found it somewhat difficult to express their expectations of nursing pain management and competencies. However, 1) being present and supportive; 2) giving information and sharing knowledge; 3) taking care of medication; and 4) recognizing the pain emerged as themes in nursing pain management. Although patients believed that nurses were caring persons, they perceived differences between nurses in the ways they handled pain management. Furthermore, some patients experienced a lack of information from nurses in relation to pain management. Although cancer patients' experiences showed the importance of nurses in pain management, it seems that nurses should have a clearer role in cancer pain management in relation to counseling and patient education. The results from this study can increase nurses' awareness of their role in pain management as a first step in improving pain management for patients.

Institutions:
(Rustøen T) Center for Shared Decision Making and Nursing Research, Rikshospitalet University Hospital, Oslo, Norway. tone.rustoen@rr-research.no
Database: PubMed

186. Cancer-related pain: a pan-European survey of prevalence, treatment, and patient attitudes.

Author(s): Breivik H; Cherny N; Collett B; de Conno F; Filbet M; Foubert AJ; Cohen R; Dow L

Source: Annals of oncology : official journal of the European Society for Medical Oncology; Aug 2009; vol. 20 (no. 8); p. 1420-1433

Publication Date: Aug 2009

Publication Type(s): Journal Article; Research Support, Non-U.S. Gov't

DOI: http://dx.doi.org/10.1093/annonc/mdp001

ISSN: 1569-8041

Place of Publication: England

PubMedID: 19244085

Accession Number: 19244085

Available at Annals of oncology : official journal of the European Society for Medical Oncology - from HighWire - Free Full Text

Abstract:BACKGROUND: The European Pain in Cancer survey sought to increase understanding of cancer-related pain and treatment across Europe.PATIENTS AND METHODS: Patients with all stages of cancer participated in a two-phase telephone survey conducted in 11 European countries and Israel in 2006-2007. The survey screened for patients experiencing pain at least weekly, then randomly selected adult patients with pain of at least moderate intensity occurring several times per week for the last month completed a detailed attitudinal questionnaire.RESULTS: Of 5084 adult patients contacted, 56% suffered moderate-to-severe pain at least monthly. Of 573 patients randomly selected for the second survey phase, 77% were receiving prescription-only analgesics, with 41% taking strong opioids either alone or with other drugs for cancer-related pain. Of those prescribed analgesics, 63% experienced breakthrough pain. In all, 69% reported pain-related difficulties with everyday activities; however, 50% believed that their quality of life was not considered a priority in their overall care by their health care professional.CONCLUSIONS: Across Europe and Israel, treatment of cancer pain is suboptimal. Pain and pain relief should be considered integral to the diagnosis and treatment of cancer; management guidelines should be revised to improve pain control in patients with cancer.

Institutions:
(Breivik H) Faculty of Medicine, University of Oslo and Department of Anaesthesiology, Rikshospitalet University Hospital, Oslo, Norway. harald.breivik@medisin.uio.no

Database: PubMed

187. Health care providers' assessments of the quality of advanced-cancer care in Latin American medical institutions: a comparison of predictors in five countries: Argentina, Brazil, Cuba, Mexico, and Peru.

Author(s): Torres-Vigil I; Aday LA; Reyes-Gibby C; De Lima L; Herrera AP; Mendoza T; Cleeland CS

Source: Journal of pain & palliative care pharmacotherapy; 2008; vol. 22 (no. 1); p. 7-20

Publication Date: 2008

Publication Type(s): Comparative Study; Journal Article
DOI: http://dx.doi.org/10.1080/15360280801989195
ISSN: 1536-0539
Place of Publication: England
PubMedID: 19042817
Accession Number: 19042817

Available at Journal of pain & palliative care pharmacotherapy - from EBSCO (MEDLINE Complete)

Abstract: This paper describes an innovative Pan-American survey on advanced-cancer care and examines the quality-of-care provided by Latin American institutions. A convenience sample of 777 physicians and nurses who treat cancer patients in Argentina, Brazil, Cuba, Mexico, and Peru were surveyed. Providers were identified through mass mailings, distribution at professional meetings and conferences, collaboration with regional institutions, professional organizations, and PAHO and online posting. Multiple linear regression analyses were conducted to identify predictors of quality-of-care assessments in each country. The five predictive models were subsequently compared descriptively. Higher access to care ratings and greater availability of end-of-life services corresponded with improved institutional quality-of-care ratings for all five countries. Barring respondents from Cuba, providers from the other four nations who practice in public institutions rated the quality of advanced-cancer care in their own institutions lower than those practicing in private hospitals or specialized cancer centers. Other institutional quality-of-care predictors included type of city, affordability-of-care ratings, availability of opioid analgesics, where patients die, barriers to cancer pain management, and the provider's specialty and gender. These findings highlight the need for providing accessible care and services to improve the quality of advanced-cancer care in Latin American institutions. Efforts should be aimed at improving the care offered in public institutions and addressing other types of disparities that may exist within countries by creating supportive and palliative cancer care programs that are accessible and affordable to those most in need.

Institutions:
(Torres-Vigil I) Department of Health Disparities Research, Center for Research on Minority Health at The University of Texas M. D. Anderson Cancer Center, Houston, TX 77021, USA. istorres@mdanderson.org
Database: PubMed

188. Doctors' opinions, knowledge and attitudes towards cancer pain management in a university hospital.

Author(s): Peker L; Celebi N; Canbay O; Sahin A; Cakir B; Uzun S; Aypar U

Source: Agri : Agri (Algoloji) Dernegi'nin Yayin organidir = The journal of the Turkish Society of Algology; Apr 2008; vol. 20 (no. 2); p. 20-30
Publication Date: Apr 2008
Publication Type(s): Journal Article
ISSN: 1300-0012
Place of Publication: Turkey

PubMedID: 19021007

Accession Number: 19021007

Abstract: Cancer pain management is still reported to be inadequate despite of recent developments in medicine, resulting in serious outcomes. This study is to evaluate opinions, knowledge and attitudes of doctors working and/or being trainedg in surgical and medical departments in our university hospital, towards cancer pain management via a questionnaire. Of all doctors approached, eighty percent could be reached and 83% of them completed the questionnaire. In this group of doctors, reportedly 60% evaluating cancer patients with pain at least once in a week, most had not have any formal education about cancer pain management during their medical school or residency training and the ones reporting "any" education, described this as "limited in quality and as hours of lessons" and were not satisfied. The results of this survey suggest specific targets for the strategic and educational projects to overcome some of the barriers against the optimal cancer pain management. Most of the doctors believe that barriers originating from health professionals and systems are more important than the ones resulting from patients and give high priority to treatment of cancer pain relative to the treatment of cancer; but still half of them report that legal regulations have some influence on opioid prescription; and almost three quarters of them believe that opioid use may cause high rates of psychological addiction or abuse. Two thirds of the doctors feel themselves "insufficient" in cancer pain management, being more prominent in tasks requiring knowledge, skill, education and experience about opioid use.

Institutions:
(Peker L) Hacettepe University Faculty Of Medicine Department Of Anesthesiology, 06100 Ankara, Turkey. nalanmd@hotmail.com

Database: PubMed

189. The Danish version of the Medication Adherence Report Scale: preliminary validation in cancer pain patients.

Author(s): Jacobsen R; Møldrup C; Christrup L; Sjøgren P; Hansen OB

Source: Pain practice : the official journal of World Institute of Pain; 2009; vol. 9 (no. 1); p. 1-7

Publication Date: 2009

Publication Type(s): Journal Article; Research Support, Non-U.S. Gov't; Validation Study

DOI: http://dx.doi.org/10.1111/j.1533-2500.2008.00245.x

ISSN: 1533-2500

Place of Publication: United States

PubMedID: 19019056

Accession Number: 19019056

Available at Pain practice : the official journal of World Institute of Pain - from Wiley Online Library Medicine and Nursing Collection 2020

Available at Pain practice : the official journal of World Institute of Pain - from EBSCO (CINAHL Complete)

Available at Pain practice : the official journal of World Institute of Pain - from EBSCO (Psychology and Behavioral Sciences Collection)

Abstract:OBJECTIVE: To examine the psychometric properties of the Danish version of the Medication Adherence Report Scale (DMARS-4) adapted to measure adherence to analgesic regimen among cancer patients.METHODS: The validated English version of the Medication Adherence Report Scale was translated into Danish following the repeated back-translation procedure. Cancer patients for the study were recruited from specialized pain management facilities. Thirty-three patients responded to the DMARS-4, the Danish Barriers Questionnaire II, The Danish version of Patient Perceived Involvement in Care Scale measuring the quality of patient-physician pain communication, and the Danish Brief Pain Inventory pain severity scale.RESULTS: A factor analysis of the DMARS-4 resulted in one factor. Mean (SD) score on the cumulative scale ranging from 4 to 20, with higher scores indicating better medication adherence, was 17.8 (0.42). The DMARS-4 scores were related to the measures of patients' concerns about pain management and patients' pain communication. The internal consistency of the DMARS-4 was 0.70.CONCLUSIONS: The DMARS-4 seems to be a valid and reliable measure of self-reported adherence to analgesic regimen in the context of cancer pain.

Institutions:
(Jacobsen R) Department of Pharmacology and Pharmacotherapy, Faculty of Pharmaceutical Sciences, University of Copenhagen, Copenhagen, Denmark. raj@farma.ku.dk
Database: PubMed

190. Opioids and cancer survivors: issues in side-effect management.

Author(s): Palos GR

Source: Oncology nursing forum; Nov 2008 ; p. 13-19

Publication Date: Nov 2008

Publication Type(s): Journal Article; Review

DOI: http://dx.doi.org/10.1188/08.ONF.S1.13-19

ISSN: 1538-0688

Place of Publication: United States

PubMedID: 18980935

Accession Number: 18980935

Available at Oncology nursing forum - from Ovid (Journals @ Ovid)

Available at Oncology nursing forum - from EBSCO (CINAHL Complete)

Available at Oncology nursing forum - from ProQuest (MEDLINE with Full Text) - NHS Version

Available at Oncology nursing forum - from ProQuest (Health Research Premium) - NHS Version

Available at Oncology nursing forum - from David Adams Library Journals Collection Local Print Collection [location] : David Adams Library.

Abstract:PURPOSE/OBJECTIVES: To describe the most common side effects associated with the use of opioid treatment in patients with moderate to severe

cancer pain; to discuss research findings specific to the use of opioids for cancer pain in long-term cancer survivors.DATA SOURCES: Published research, articles from a literature review, and U. S. statistics.DATA SYNTHESIS: Side effects associated with opioid use are a major contributor to patient reluctance to follow treatment plans for cancer pain. Clinicians must follow the critical steps necessary to build comprehensive treatment plans that include a preventive approach to side effects and opioid rotation when side effects do not resolve.CONCLUSIONS: Side effects associated with long-term use of opioids by cancer survivors are a major contributor to patient reluctance to follow a cancer pain treatment plan. Patient education efforts must promote open and clear communication between survivors and their providers about side effects and other important issues related to long-term use of opioids in managing pain related to cancer and its treatment.IMPLICATIONS FOR NURSING: Oncology nurses recognize that patients often require the long-term use of opioids when they experience chronic pain as a result of their disease or its treatment. The long-term physical and cognitive effects of such opioid use are not well known, despite the advances that have been made in cancer pain control and research. Survivors should communicate their concerns about side effects to the treatment team. In addition, patients and family members must be encouraged to inform their providers about personal attitudes, beliefs, and practices that may affect decisions about taking their analgesics as prescribed. Most importantly, oncology nurses must teach patients and their families to self-advocate for optimal pain relief with minimal side effects.

Institutions:
(Palos GR) Division of Internal Medicine, Department of Symptom Research, The University of Texas, M.D. Anderson Cancer Center, Houston, TX, USA. gpalos@mdanderson.org

Database: PubMed

191. Cancer pain: perspectives of a medical oncologist.

Author(s): Eaton KD; Frieze DA

Source: Current pain and headache reports; Aug 2008; vol. 12 (no. 4); p. 270-276

Publication Date: Aug 2008

Publication Type(s): Journal Article; Review

DOI: http://dx.doi.org/10.1007/s11916-008-0046-x

ISSN: 1534-3081

Place of Publication: United States

PubMedID: 18625104

Accession Number: 18625104

Available at Current pain and headache reports - from EBSCO (MEDLINE Complete)

Available at Current pain and headache reports - from ProQuest (Health Research Premium) - NHS Version

Abstract: Pain is highly prevalent in cancer patients and primarily managed by medical oncologists. This article reviews cancer pain syndromes related to cancer and sequelae of treatment. We discuss the assessment and treatment of cancer pain

with pharmacotherapy and chemotherapy, and the role of pain specialists. There are numerous barriers to care, which arise from both the physician and patient. We review approaches that diminish these barriers to improve treatment of cancer pain.
Institutions:
(Eaton KD) University of Washington, Seattle Cancer Care Alliance, 825 Eastlake Avenue East, Seattle, WA 98109, USA. kdeaton@u.washington.edu
Database: PubMed

192. Pain centers professionals' beliefs on non-cancer chronic pain.

Author(s): Garcia DM; Mattos-Pimenta CA

Source: Arquivos de neuro-psiquiatria; Jun 2008; vol. 66 (no. 2A); p. 221-228

Publication Date: Jun 2008

Publication Type(s): Journal Article; Multicenter Study

DOI: http://dx.doi.org/10.1590/s0004-282x2008000200016

ISSN: 0004-282X

Place of Publication: Brazil

PubMedID: 18545787

Accession Number: 18545787

Available at Arquivos de neuro-psiquiatria - from Unpaywall

Abstract: The beliefs and attitudes of health professionals affect the care ultimately provided to patients. The objective of this study was to analyze health professionals' beliefs toward chronic no cancer pain in nine (82%) pain centers in the city of S.Paulo. The Survey of Chronic Pain Attitudes-Professionals was employed to evaluate pain professionals' beliefs toward emotions, control, disability, solicitude, cure and harm. A total of 75 health professionals (59%), most of whom were doctors (44), followed by physical therapist (11) and dentists (8), were interviewed. The professionals professed a belief in a medical cure for chronic pain, that solicitous displays were desirable behaviors in treating pain, that chronic pain is related to injury and that it is the cause of disability, all of which are erroneous beliefs. Contrary to the expected result, the health professionals with more experience and education did not express more appropriate beliefs. These beliefs may compromise the treatment of patients with chronic pain and should therefore be reviewed.

Institutions:
(Garcia DM) Grupo de Dor, Hospital Sírio-Libanês, São Paulo, Brazil.
Database: PubMed

193. [Variable prescription of opioids to cancer patients in Norway].

Author(s): Olsen AS; Ottesen S

Source: Tidsskrift for den Norske laegeforening : tidsskrift for praktisk medicin, ny raekke; May 2008; vol. 128 (no. 11); p. 1271-1274

Publication Date: May 2008

Publication Type(s): Journal Article

ISSN: 0807-7096

Place of Publication: Norway

PubMedID: 18511968

Accession Number: 18511968

Abstract:BACKGROUND: We examined prescription of the opioids used most often in cancer pain relief in the Norwegian counties, doctors' use of the law section concerning reimbursement of medicine to incurable far advanced cancer patients, and the prescription of analgesics with codein to this patient group.MATERIAL AND METHOD: Data were retrieved from The Norwegian prescription database for the period 01.01.04 to 31.08.05 (NorPD) on every prescription of slow and instant release morphine and oxycodone for oral administration and of transdermal fentanyl patches. SPSS was used for analyses.RESULTS: 5,675,038 Defined Daily Doses (DDD) of strong opioids were prescribed to 20,797 patients (46% of these to cancer patients) from 01.01.04 to 31.08.05. The county that prescribed most had prescribed 2.83 times more opioids than the county that had prescribed the least. Use of the law section concerning prescription of opioids indicates that these are prescribed as much for non-malignant diseases as for cancer. "Codein analgesics" are also commonly prescribed to cancer patients.INTERPRETATION: Prescription of strong opioids varies substantially in Norway's counties. Doctors' knowledge, attitude, geographical and cultural differences may be some reasons for this. Knowledge about and use of the law section concerning reimbursement is probably not good enough. Increased awareness of how to treat cancer pain is important go give a good treatment offer to all in Norway.

Institutions:
(Olsen AS) Det medisinske fakultet, Universitetet i Tromsø, 9037 Tromsø.
astorol@mailbox.uit.no
Database: PubMed

194. Concepts within the Chinese culture that influence the cancer pain experience.

Author(s): Chen LM; Miaskowski C; Dodd M; Pantilat S

Source: Cancer nursing; 2008; vol. 31 (no. 2); p. 103-108

Publication Date: 2008

Publication Type(s): Journal Article; Review

DOI: http://dx.doi.org/10.1097/01.NCC.0000305702.07035.4d

ISSN: 1538-9804

Place of Publication: United States

PubMedID: 18490884

Accession Number: 18490884

Available at Cancer nursing - from Ovid (Journals @ Ovid)

Available at Cancer nursing - from Ovid (Journals @ Ovid) - London Health Libraries

Available at Cancer nursing - from David Adams Library Journals Collection Local Print Collection [location] : David Adams Library.

Abstract: The purpose of this article is to describe some of the concepts within the Chinese culture that influence the sociocultural dimension of the cancer pain experience. The major concepts that influence Chinese patients' perspectives on cancer pain and its management include Taoism/energy, Buddhism, and Confucianism. Within the beliefs of Taoism/energy, pain occurs if Qi, or blood circulation, is blocked. To relieve pain, the blockage of Qi/blood must be removed and the person needs to maintain harmony with the universe. Within the beliefs of Buddhism, pain/suffering is a power, unwanted but existent, that comes from a barrier in the last life; from the objective world; from a person's own sensation; or from other people, animals, and materials. Only by following the 8 right ways (ie, right view, right intention, right speech, right action, right livelihood, right effort, right mindfulness, and right concentration) can an individual end the path of pain/suffering. A Confucian believes that pain is an essential element of life, a "trial" or a "sacrifice." Therefore, when a person suffers with pain, he or she would rather endure the pain and not report it to a clinician until the pain becomes unbearable. Oncology nurses who care for Chinese patients need to understand the fundamental beliefs that influence the sociocultural dimension of the pain experience for these patients. This information will assist the oncology nurse in developing a more effective pain management plan.

Institutions:
(Chen LM) School of Nursing, Kaohsiung Medical University, Kaohsiung City, Taiwan. Lih-Mih.Chen@ucsf.edu

Database: PubMed

195. Prevalence and treatment of cancer pain in Italian oncological wards centres: a cross-sectional survey.

Author(s): Mercadante S; Roila F; Berretto O; Labianca R; Casilini S; DOMAIN-AIOM study group

Source: Supportive care in cancer : official journal of the Multinational Association of Supportive Care in Cancer; Nov 2008; vol. 16 (no. 11); p. 1203-1211

Publication Date: Nov 2008

Publication Type(s): Journal Article

DOI: http://dx.doi.org/10.1007/s00520-008-0456-7

ISSN: 0941-4355

Place of Publication: Germany

PubMedID: 18478276

Accession Number: 18478276

Available at Supportive care in cancer : official journal of the Multinational Association of Supportive Care in Cancer - from SpringerLink

Available at Supportive care in cancer : official journal of the Multinational Association of Supportive Care in Cancer - from EBSCO (MEDLINE Complete)

Available at Supportive care in cancer : official journal of the Multinational Association of Supportive Care in Cancer - from EBSCO (CINAHL Complete)

Available at Supportive care in cancer : official journal of the Multinational Association of Supportive Care in Cancer - from ProQuest (MEDLINE with Full Text) - NHS Version

Available at Supportive care in cancer : official journal of the Multinational Association of Supportive Care in Cancer - from ProQuest (Health Research Premium) - NHS Version

Available at Supportive care in cancer : official journal of the Multinational Association of Supportive Care in Cancer - from David Adams Library Journals Collection Local Print Collection [location] : David Adams Library.

Abstract:OBJECTIVE: The aim of this national cross-sectional survey was to draw information on pain prevalence and intensity from a large sample of patients who were admitted to oncologic centres for different reasons and to evaluate the pain treatment and possible influencing factors.MATERIALS AND METHODS: A total of 2,655 patients completed the study. Nine hundred and one patients (34%) reported pain.RESULTS AND DISCUSSIONS: Higher pain levels were observed in inpatients, in the presence of bone metastases, and with low levels of Eastern Cooperative Oncology Group status. The number of patients receiving strong opioids increased with the highest levels of pain. However, a significant part of patients with moderate-severe pain were not receiving appropriate medication, patients being predominantly administered non-opioid drugs. General practitioners' attitudes did not negatively influence the opioid prescription.CONCLUSION: The results of this survey indicate a need for continuing educational and informative program in pain management for oncologists and more generally for any physician dealing with cancer patients.

Institutions:
(Mercadante S) La Maddalena Cancer Center, Palermo, Italy. terapiadeldolore@lamaddalenanet.it; (Roila F) DOMAIN-AIOM study group
(Mercadante S) La Maddalena Cancer Center, Palermo, Italy. terapiadeldolore@lamaddalenanet.it; (Roila F) DOMAIN-AIOM study group
Database: PubMed

196. [Nursing certification system in cancer nursing].

Author(s): Hasegawa K

Source: Gan to kagaku ryoho. Cancer & chemotherapy; Apr 2008; vol. 35 (no. 4); p. 572-577

Publication Date: Apr 2008

Publication Type(s): English Abstract; Journal Article

ISSN: 0385-0684

Place of Publication: Japan

PubMedID: 18408425

Accession Number: 18408425

Abstract: The Japanese Nursing Association (JNA) started an advanced nursing certification system and gave the first certification to certified nurse specialist (CNS) in cancer nursing in 1996. CNS is recognized for excellent nursing practice in a specific area, and for demonstrating six roles: excellent nursing practice, consultation, ethical coordination, education, coordination, and research activities. To

receive CNS certification need nurse (or public health nurse or nurse midwife) qualification, master's degree, at least five-years clinical experience, and after that to pass a JNA certification examination. Certified nurse (CN) demonstrates three roles in high level of nursing practice by using matured nursing skills and knowledge, leadership, and consultation in a specific nursing area. CN need nurse qualification, at least fiveyears clinical experience, completing educational program at least 6 months, and passing a JNA certification examination. Both of CNS and CN requier certification renewal every five years. The number of people who get certification of CNS's in cancer nursing become 104, and of CN in cancer nursing area become 942 (chemotherapy 204, palliative care 420, cancer pain 267, breast cancer 51) in 2007. CNS's in cancer nursing activities are not understood broaden, because they have various work positions and activities, and are very few in Japan. But they are considered to be a change agent in cancer health care system. Also they will expand their activity setting. There is a prospect that CNS in cancer nursing will increase in number with nursing graduate school increasing. It shows that we will face some problems, for example educational contents or methods, certification system and so on.

Institutions:
(Hasegawa K) Toranomon Hospital, Minato-ku, Tokyo, Japan.
Database: PubMed

197. Nurse coaching to explore and modify patient attitudinal barriers interfering with effective cancer pain management.

Author(s): Fahey KF; Rao SM; Douglas MK; Thomas ML; Elliott JE; Miaskowski C

Source: Oncology nursing forum; Mar 2008; vol. 35 (no. 2); p. 233-240

Publication Date: Mar 2008

Publication Type(s): Journal Article

DOI: http://dx.doi.org/10.1188/08.ONF.233-240

ISSN: 1538-0688

Place of Publication: United States

PubMedID: 18321835

Accession Number: 18321835

Available at Oncology nursing forum - from Ovid (Journals @ Ovid)

Available at Oncology nursing forum - from EBSCO (CINAHL Complete)

Available at Oncology nursing forum - from ProQuest (MEDLINE with Full Text) - NHS Version

Available at Oncology nursing forum - from ProQuest (Health Research Premium) - NHS Version

Available at Oncology nursing forum - from David Adams Library Journals Collection Local Print Collection [location] : David Adams Library.

Abstract:PURPOSE/OBJECTIVES: To describe a complex coaching intervention to help patients with cancer pain explore beliefs and attitudinal barriers interfering with pain management. Patients were coached to explore beliefs about pain, communications about pain management, and the use of analgesics and

nonpharmacologic interventions.DATA SOURCES: Published journal articles, abstracts, and psychology textbooks.DATA SYNTHESIS: Personal beliefs, related attitudinal barriers, and associated behaviors impede patient adherence to and success with pain management treatments. Interventions targeting beliefs help patients overcome attitudinal barriers, improve treatment adherence, and obtain better pain relief.CONCLUSIONS: Coaching patients to explore beliefs reduces ineffective behaviors and improves pain treatment adherence.IMPLICATIONS FOR NURSING: A coaching intervention incorporating assessment of patient beliefs promotes self-management, self-efficacy, and adherence to pain management treatment plans. Advanced practice nurses should consider incorporating this intervention into their communications with patients experiencing cancer pain.

Institutions:
(Fahey KF) Palliative Care, El Camino Hospital in Mountain View, CA, USA. kathleen_fahey@sbcglobal.net
Database: PubMed

198. Knowledge and attitudes of Turkish oncology nurses about cancer pain management.

Author(s): Yildirim YK; Cicek F; Uyar M

Source: Pain management nursing : official journal of the American Society of Pain Management Nurses; Mar 2008; vol. 9 (no. 1); p. 17-25

Publication Date: Mar 2008

Publication Type(s): Journal Article

DOI: http://dx.doi.org/10.1016/j.pmn.2007.09.002

ISSN: 1532-8635

Place of Publication: United States

PubMedID: 18313586

Accession Number: 18313586

Abstract: Effective pain management requires accurate knowledge, attitudes, and assessment skills. The purpose of the present study was to examine information about the knowledge and attitudes of Turkish oncology nurses regarding cancer pain management. The sample consisted of 68 oncology nurses employed in oncology and hematology units in two university hospitals located in Izmir, Turkey. The Nurses' Knowledge and Attitudes Survey Regarding Pain was used to measure the nurses' pain management knowledge and attitudes. Data were analyzed by using descriptive statistics, t test, Kruskal-Wallis analysis, and Pearson correlation test. Of the nurses that participated in the study, 57.4% were between the ages of 21 and 30 years, 58.8% were unmarried, and 55.9% had an associate degree. The average correct response rate was 35.41%, with rates ranging from 5.13% to 56.41% for each survey question. Among the 39 pain knowledge questions assessed, the mean number of correctly answered questions was 13.81 +/- 5.02, with a range of 2 to 22 items correctly answered. When the knowledge scores were further analyzed by nurses' background characteristics, the nurses' pain knowledge was only positively correlated to length of working experience in oncology units ($r = 0.263$; $p < .05$). The findings support the concern of inadequate knowledge and attitudes in relation to

cancer pain management. We believe that basic and continuing education programs may improve knowledge level of nursing about pain management.

Institutions:
(Yildirim YK) Department of Internal Medicine Nursing, Ege University Nursing School, Bornova, Izmir, Turkey. yasemin.kyildirim@ege.edu.tr
Database: PubMed

199. Action research: developing a pediatric cancer pain program in jordan.

Author(s): Finley GA; Forgeron P; Arnaout M

Source: Journal of pain and symptom management; Apr 2008; vol. 35 (no. 4); p. 447-454

Publication Date: Apr 2008

Publication Type(s): Journal Article; Research Support, Non-U.S. Gov't

DOI: http://dx.doi.org/10.1016/j.jpainsymman.2007.05.006

ISSN: 0885-3924

Place of Publication: United States

PubMedID: 18258410

Accession Number: 18258410

Available at Journal of pain and symptom management - from David Adams Library Journals Collection Local Print Collection [location] : David Adams Library.

Available at Journal of pain and symptom management - from Unpaywall

Abstract: Children's pain is undertreated worldwide. Using a model of pediatric cancer pain management in Amman, Jordan, the authors demonstrated that an action research approach to pain service development resulted in a sustainable program of pain control. Barriers to care were due more often to health professionals' misconceptions concerning pain and opioid use than to concerns related to cultural, religious, or societal beliefs. Successful implementation of a pain management program requires education, policy development, and support from several levels of hospital administration. Role-modeling and mentorship are important factors. Established knowledge translation theories explained some but not all of the findings. Outcomes included consistent pain assessment and documentation by nursing staff, increased consultation for pain management, and increased use of intravenous opioids.

Institutions:
(Finley GA) Dalhousie University, Halifax, Nova Scotia, Canada. allen.finley@dal.ca <allen.finley@dal.ca>
Database: PubMed

200. Controlling cancer pain with pharmacotherapy.

Author(s): Pharo GH; Zhou L

Source: The Journal of the American Osteopathic Association; Dec 2007; vol. 107 (no. 12 Suppl 7); p. ES22

Publication Date: Dec 2007

Publication Type(s): Journal Article; Review

ISSN: 1945-1997

Place of Publication: United States

PubMedID: 18165374

Accession Number: 18165374

Abstract: Cancer remains the second most common cause of death in the United States despite advances in prevention, early detection, and newer treatment protocols. Pain continues to be the most feared complication of this diagnosis. Numerous studies have shown that when the World Health Organization treatment guidelines are followed, 90% of patients are pain-free. Although clinical evidence is convincing that opioids are effective in treating patients for cancer pain, physician reluctance to prescribe them and patient unwillingness to take such medication continue. Barriers to opioid use are multifactorial, but with education of healthcare providers and patients, pharmacotherapy for pain management will be more effective.

Institutions:
(Pharo GH) Professional Pain Management Associates, Suite 308, 829 Spruce St, Philadelphia, PA 19107, USA. gregory.pharo@ppmapc.com

Database: PubMed

201. Patient training in cancer pain management using integrated print and video materials: a multisite randomized controlled trial.

Author(s): Syrjala KL; Abrams JR; Polissar NL; Hansberry J; Robison J; DuPen S; Stillman M; Fredrickson M; Rivkin S; Feldman E; Gralow J; Rieke JW; Raish RJ; Lee DJ; Cleeland CS; DuPen A

Source: Pain; Mar 2008; vol. 135 (no. 1-2); p. 175-186

Publication Date: Mar 2008

Publication Type(s): Journal Article; Multicenter Study; Randomized Controlled Trial; Research Support, N.I.H., Extramural

DOI: http://dx.doi.org/10.1016/j.pain.2007.10.026

ISSN: 1872-6623

Place of Publication: United States

PubMedID: 18093738

Accession Number: 18093738

Available at Pain - from Unpaywall

Abstract: Standard guidelines for cancer pain treatment routinely recommend training patients to reduce barriers to pain relief, use medications appropriately, and communicate their pain-related needs. Methods are needed to reduce professional time required while achieving sustained intervention effectiveness. In a multisite, randomized controlled trial, this study tested a pain training method versus a nutrition control. At six oncology clinics, physicians (N=22) and nurses (N=23) enrolled patients (N=93) who were over 18 years of age, with cancer diagnoses, pain, and a life expectancy of at least 6 months. Pain training and control interventions were matched for materials and method. Patients watched a video followed by about 20

min of manual-standardized training with an oncology nurse focused on reviewing the printed material and adapted to individual concerns of patients. A follow-up phone call after 72 h addressed individualized treatment content and pain communication. Assessments at baseline, one, three, and 6 months included barriers, the Brief Pain Inventory, opioid use, and physician and nurse ratings of their patients' pain. Trained versus control patients reported reduced barriers to pain relief (P<.001), lower usual pain (P=.03), and greater opioid use (P6 on a 0-10 scale) at 1-month outcomes (P=.03). Physician and nurse ratings were closer to patients' ratings of pain for trained versus nutrition groups (P=.04 and <.001, respectively). Training efficacy was not modified by patient characteristics. Using video and print materials, with brief individualized training, effectively improved pain management over time for cancer patients of varying diagnostic and demographic groups.

Institutions:
(Syrjala KL) Biobehavioral Sciences, Clinical Research Division, Fred Hutchinson Cancer Research Center, D5-220, 1100 Fairview Avenue N, Seattle, WA 98109, USA. ksyrjala@fhcrc.org
Database: PubMed

202. [An attitude survey on the medical use of narcotics for cancer pain relief].

Author(s): Sato Y

Source: Gan to kagaku ryoho. Cancer & chemotherapy; Dec 2007; vol. 34 (no. 13); p. 2267-2270

Publication Date: Dec 2007

Publication Type(s): English Abstract; Journal Article

ISSN: 0385-0684

Place of Publication: Japan

PubMedID: 18079628

Accession Number: 18079628

Abstract: Narcotic medications are the major drug therapy for cancer pain relief. A clinical use of fentanyl patches and oxycodone extended-release tablets has recently become available, which led to more choices of narcotics to be used medically. On the other hand, palliative care systems have not yet been fully established by medical institutions. The management of symptoms including pain, therefore, actually has to be performed by "care doctors" involved in cancer care. We conducted a survey on the medical use of narcotics for 500 people, including cancer patients and their family members. The result showed that the recognition rate of morphine used for cancer pain relief was 88%, while the recognition rate of narcotics for medical use other than morphine available for cancer pain relief was 20%. The most acceptable dosage form of narcotics was a skin patch, followed by an oral preparation. A response percentage that the dosage form of medical narcotics should be selectable by users was 93%.

Institutions:
(Sato Y) Dept. of Surgery, Saiseikai Wakakusa Hospital.
Database: PubMed

203. Audit and feedback as a clinical practice guideline implementation strategy: a model for acute care nurse practitioners.

Author(s): Dulko D

Source: Worldviews on evidence-based nursing; 2007; vol. 4 (no. 4); p. 200-209

Publication Date: 2007

Publication Type(s): Journal Article; Review; Systematic Review

DOI: http://dx.doi.org/10.1111/j.1741-6787.2007.00098.x

ISSN: 1545-102X

Place of Publication: United States

PubMedID: 18076463

Accession Number: 18076463

Available at Worldviews on evidence-based nursing - from Wiley Online Library Medicine and Nursing Collection 2020

Available at Worldviews on evidence-based nursing - from EBSCO (CINAHL Complete)

Abstract:BACKGROUND: The transfer of research evidence into practice and changing provider behavior is challenging, even when the advantages are strong. Despite the availability of supportive care clinical practice guidelines (CPG), consistent integration of these principles into practice has not been achieved. The failure of dissemination strategies has been identified as a key barrier to successful implementation. A potentially effective approach to facilitating the transfer of research evidence into practice is audit and feedback. Audit and feedback is a summary of provider performance over a specified period of time, with or without recommendations to improve practice.RATIONALE: Cancer pain is an optimal symptom to examine when studying the effect of an audit and feedback intervention. It is a common condition with important consequences, established CPG are available, measurable outcomes are defined, and there is potential for improvement in current practice. Acute care nurse practitioners (NPs) are often responsible for overseeing and directly managing symptoms such as pain and are well positioned to implement CPG and study the effects of adherence to guidelines on patients' pain outcomes.METHODOLOGY: A systematic review of published articles, MEDLINE, the Cumulative Index to Nursing and Allied Health Literature, and the Cochrane Library computerized databases was performed to evaluate the state of the science on audit and feedback as a professional practice change strategy. A behavior change model is proposed for its application to advanced practice nursing.IMPLICATIONS FOR PRACTICE: Recognized in medicine as a valuable intervention to improve healthcare quality, audit and feedback is a strategy that has not been widely studied in nursing. Although cancer pain cannot always be entirely eliminated, appropriate use of available therapies can effectively relieve pain in a majority of patients. This article is a review of the literature on audit and feedback as a professional practice change strategy and indicates a model for operationalizing the intervention.

Institutions:
(Dulko D) Memorial Sloan-Kettering Cancer Center, New York, NY 10021, USA. dulko2@mskcc.org

Database: PubMed

204. Implementing the Fatigue Guidelines at one NCCN member institution: process and outcomes.

Author(s): Borneman T; Piper BF; Sun VC; Koczywas M; Uman G; Ferrell B

Source: Journal of the National Comprehensive Cancer Network : JNCCN; Nov 2007; vol. 5 (no. 10); p. 1092-1101

Publication Date: Nov 2007

Publication Type(s): Journal Article; Practice Guideline; Review

DOI: http://dx.doi.org/10.6004/jnccn.2007.0090

ISSN: 1540-1405

Place of Publication: United States

PubMedID: 18053431

Accession Number: 18053431

Available at Journal of the National Comprehensive Cancer Network : JNCCN - from Unpaywall

Abstract: Fatigue, despite being the most common and distressing symptom in cancer, is often unrelieved because of numerous patient, provider, and system barriers. The overall purpose of this 5-year prospective clinical trial is to translate the NCCN Cancer-Related Fatigue Clinical Practice Guidelines in Oncology and NCCN Adult Cancer Pain Clinical Practice Guidelines in Oncology into practice and develop a translational interventional model that can be replicated across settings. This article focuses on one NCCN member institution's experience related to the first phase of the NCCN Cancer-Related Fatigue Guidelines implementation, describing usual care compared with evidence-based guidelines. Phase 1 of this 3-phased clinical trial compared the usual care of fatigue with that administered according to the NCCN guidelines. Eligibility criteria included age 18 years or older; English-speaking; diagnosed with breast, lung, colon, or prostate cancer; and fatigue and/or pain ratings of 4 or more on a 0 to 10 screening scale. Research nurses screened all available subjects in a cancer center medical oncology clinic to identify those meeting these criteria. Instruments included the Piper Fatigue Scale, a Fatigue Barriers Scale, a Fatigue Knowledge Scale, and a Fatigue Chart Audit Tool. Descriptive and inferential statistics were used in data analysis. At baseline, 45 patients had fatigue only (> or = 4) and 24 had both fatigue and pain (> or = 4). This combined sample (N = 69) was predominantly Caucasian (65%), female (63%), an average of 60 years old, diagnosed with stage 3 or 4 breast cancer, and undergoing treatment (82%). The most common barriers noted were patients' belief that physicians would introduce the subject of fatigue if it was important (patient barrier); lack of fatigue documentation (professional barrier); and lack of supportive care referrals (system barrier). Findings showed several patient, professional, and system barriers that distinguish usual care from that recommended by the NCCN Cancer-Related Fatigue Guidelines. Phase 2, the intervention model, is designed to decrease these barriers and improve patient outcomes over time, and is in progress.

Institutions:
(Borneman T) Department of Nursing Research & Education, Division of Population Sciences, Beckman Research Institute, City of Hope National Medical Center, 1500 E. Duarte Road, Duarte, CA 91010, USA. tborneman@coh.org

Database: PubMed

205. Physician-related barriers to cancer pain management with opioid analgesics: a systematic review.

Author(s): Jacobsen R; Sjøgren P; Møldrup C; Christrup L

Source: Journal of opioid management; 2007; vol. 3 (no. 4); p. 207-214

Publication Date: 2007

Publication Type(s): Journal Article; Research Support, Non-U.S. Gov't; Review; Systematic Review

DOI: http://dx.doi.org/10.5055/jom.2007.0006

ISSN: 1551-7489

Place of Publication: United States

PubMedID: 17957980

Accession Number: 17957980

Abstract:OBJECTIVE: The purpose of this review is to summarize the results of studies on physician-related barriers to cancer pain management with opioid analgesics.METHODS: A literature search was conducted in PUBMED, using a combined text word and MeSH heading search strategy. Those articles whose full texts were not available in PUBMED were retrieved from the electronic databases of specific journals.RESULTS: Sixty-five relevant articles, published in the period from 1986 to 2006, were identified. Physicians' barriers to cancer pain management were studied in questionnaire surveys and in the reviews of drug prescribing documents. The results of the articles found were analyzed with respect to (a) knowledge, beliefs, concerns, problems endorsed or acknowledged by physicians treating cancer pain, (b) physicians' skills in pain assessment, and (c) adequacy of opioid prescription.CONCLUSIONS: This review revealed mostly general and common physician-related barriers to cancer pain management: concerns about side effects to opioids, prescription of not efficient doses of opioids, and very poor prescription for the treatment of side effects from opioids. In the future, the evaluation of the influence of cultural-social-economical background, as well as the differences between the various specialists involved in the care of patients with cancer, should be explored to better understand physicians' barriers and more effectively address them in interventional and educational programs.

Institutions:
(Jacobsen R) Department of Pharmacology and Pharmacotherapy, Faculty of Pharmaceutical Sciences, University of Copenhagen, Denmark.
Database: PubMed

206. Evaluation of "The Many Faces of Pain": a chronic cancer pain management education program.

Author(s): Wells J; Turner B; Coombs E

Source: Canadian oncology nursing journal = Revue canadienne de nursing oncologique; 2007; vol. 17 (no. 2); p. 91-101

Publication Date: 2007

Publication Type(s): Clinical Trial; Journal Article

DOI: http://dx.doi.org/10.5737/1181912x1729195

ISSN: 1181-912X

Place of Publication: Canada

PubMedID: 17847984

Accession Number: 17847984

Available at Canadian oncology nursing journal = Revue canadienne de nursing oncologique - from David Adams Library Journals Collection Local Print Collection [location] : David Adams Library.

Available at Canadian oncology nursing journal = Revue canadienne de nursing oncologique - from Unpaywall

Abstract:A descriptive, correlational design was used to evaluate the effectiveness of a chronic cancer pain management education program. The Nurses' Knowledge and Attitude Survey Regarding Pain (NKAS) was used to evaluate the program. A convenience sample of 27 registered nurses was recruited to participate in the study. Analysis of the data revealed a significant difference between pre-test and post-test scores on the NKAS. There were no significant correlations observed between any study variables. The small convenience sample prevents the ability to generalize the findings. It is concluded that the education program was effective in improving knowledge and attitudes related to chronic cancer pain management. Implications for nursing practice, research, education, and administration are suggested.

Institutions:
(Wells J) Western Regional School of Nursing, Western Regional Integrated Health Authority, P.O. Box 2005, Corner Brook, NL A2H 6J7. jwells@swgc.mun.ca
Database: PubMed

207. Management of cancer pain with complementary therapies.

Author(s):

Source: Oncology (Williston Park, N.Y.); Apr 2007; vol. 21 (no. 4 Suppl); p. 10-22; discussion 22

Publication Date: Apr 2007

Publication Type(s): Journal Article; Review

ISSN: 0890-9091

Place of Publication: United States

PubMedID: 17508495

Accession Number: 17508495

Available at Oncology (Williston Park, N.Y.) - from ProQuest (MEDLINE with Full Text) - NHS Version

Available at Oncology (Williston Park, N.Y.) - from ProQuest (Health Research Premium) - NHS Version

Abstract: Pain is one of the most feared consequences of cancer. Pain is a major symptom in 75% of hospitalized cancer patients. Poorly relieved pain contributes to the suffering of the patient and family, which may motivate them to seek additional complementary and alternative therapies. Evidence-based complementary therapies are being used for symptom control and to improve quality of life. There is recent

research on several complementary therapies-acupuncture, mind-body therapies, massage, reflexology, and Reiki--that provides evidence for pain management. These therapies are not well utilized due to a lack of information on benefits, risks, and resources. There is a call for education to alert patients, families, nurses, and physicians to the benefits of evidence-based complementary therapies and to the dangers of "unproven" cancer therapies. Oncology nurses are ideally positioned to assess patients' pain, to educate patients, to determine with the patient and physician the most appropriate and safe complementary therapy for pain, to refer patients to appropriate resources, and in some cases to provide the therapy itself. This article will discuss specific complementary therapies for pain control and will arm nurses with the confidence to intervene with knowledge, referrals, and ideas for hands-on implementation.

Database: PubMed

208. Educating for tomorrow: enhancing nurses' pain management knowledge.

Author(s): Linkewich B; Sevean P; Habjan S; Poling M; Bailey S; Kortes-Miller K

Source: The Canadian nurse; Apr 2007; vol. 103 (no. 4); p. 24-28

Publication Date: Apr 2007

Publication Type(s): Evaluation Study; Journal Article

ISSN: 0008-4581

Place of Publication: Canada

PubMedID: 17494475

Accession Number: 17494475

Available at The Canadian nurse - from EBSCO (MEDLINE Complete)

Available at The Canadian nurse - from EBSCO (CINAHL Complete)

Available at The Canadian nurse - from ProQuest (Health Research Premium) - NHS Version

Available at The Canadian nurse - from ProQuest (MEDLINE with Full Text) - NHS Version

Available at The Canadian nurse - from ProQuest (Health Research Premium) - NHS Version

Abstract: The Centre for Education and Research on Aging and Health at Lakehead University, Thunder Bay, Ontario, was the lead agency in developing a pain management continuing education program for front-line nurses in a variety of settings in northwestern Ontario. A committee of experts from the centre as well as from the Thunder Bay Regional Health Sciences Centre; Regional Cancer Care; the Pain and Symptom Management Team, North West Community Care Access Centre; the Victorian Order of Nurses and Lakehead University school of nursing developed the program. The program included a pre-test of knowledge and attitudes; four two-hour educational sessions focusing on total pain, acute pain, chronic pain and cancer pain; and a post self-test at the end of each session. The educational sessions were evaluated through a survey to participants. Overall, the nurses expressed high satisfaction with the workshops, and the post self-tests indicated a better understanding of patients' pain management experiences. As a result of the

evaluation, the education planning committee refined the program, which is currently being delivered to nurses in rural and remote communities via telehealth.

Institutions:
(Linkewich B) Health Services, Meno Ya Win Health Centre, Sioux Lookout, Ontario, Canada.

Database: PubMed

209. Pain management in hospitalized cancer patients: a systematic review.

Author(s): Goldberg GR; Morrison RS

Source: Journal of clinical oncology : official journal of the American Society of Clinical Oncology; May 2007; vol. 25 (no. 13); p. 1792-1801

Publication Date: May 2007

Publication Type(s): Journal Article; Research Support, N.I.H., Extramural; Research Support, Non-U.S. Gov't; Review; Systematic Review

DOI: http://dx.doi.org/10.1200/JCO.2006.07.9038

ISSN: 1527-7755

Place of Publication: United States

PubMedID: 17470871

Accession Number: 17470871

Abstract:PURPOSE: To assist cancer centers in improving pain management, we conducted a systematic review of institutional interventions designed to improve the assessment and treatment of pain in hospitalized cancer patients.METHODS: We performed a MEDLINE search for all English-language articles published from January 1966 through February 2006 using the medical subject headings terms of pain or pain measurement and outcome assessment (health care) or quality assurance (health care). Selected bibliographies were also searched. Studies were reviewed if they included clinical interventions directed at improving the treatment of cancer pain across an institution or nursing unit. Meta-analyses and randomized controlled trials or other controlled studies were included where possible. If no such trials were identified, then the best evidence available from studies with other designs was included.RESULTS: Five interventions were identified. These interventions included professional and patient education, instituting regular pain assessment (pain as a vital sign), audit of pain results and feedback to clinical staff, computerized decisional support systems, and specialist-level pain consultation services. Most studies were small in size and used quasiexperimental pre-post test designs. Successes were reported in increasing patient satisfaction, increasing documentation of pain intensity, and improving nurses' knowledge and attitudes. No study reported successful interventions that consistently improved patients' pain severity.CONCLUSION: Although professional knowledge and attitudes about pain and nursing pain assessment rates have been shown to be improvable, no systematic, hospital-wide intervention has yet to be associated with improvement in pain severity. Future research on the development of new interventions, perhaps targeted specifically at physicians, is urgently needed.

Institutions:
(Goldberg GR) Division of Hematology and Medical Oncology, Samuel Bronfman Department of Medicine, Mount Sinai School of Medicine, New York 10029, USA.

Database: PubMed

210. Knowledge and attitudes about cancer pain management: a national survey of Italian hospice nurses.

Author(s): Bernardi M; Catania G; Tridello G

Source: Cancer nursing; 2007; vol. 30 (no. 2); p. E20

Publication Date: 2007

Publication Type(s): Journal Article

DOI: http://dx.doi.org/10.1097/01.NCC.0000265299.25017.24

ISSN: 1538-9804

Place of Publication: United States

PubMedID: 17413771

Accession Number: 17413771

Available at Cancer nursing - from Ovid (Journals @ Ovid)

Available at Cancer nursing - from Ovid (Journals @ Ovid) - London Health Libraries

Available at Cancer nursing - from David Adams Library Journals Collection Local Print Collection [location] : David Adams Library.

Abstract: To obtain information about the knowledge and attitudes of Italian hospice nurses concerning cancer pain management and to determine the predictor of nurses' pain management knowledge. Nationwide descriptive study. Hospice nurses in Italy from 9 hospice units distributed in the north, center, and south of Italy. Sixty-six nurses completed the questionnaire, indicating a 66.6% response rate. The Nurses' Knowledge and Attitudes Survey (Italian version) and a background information form were used to collect the data. Knowledge and attitudes regarding cancer pain. Among the 39 pain knowledge questions assessed, the mean number of correctly answered question was 24.4 (SD = 4.2), with a range of 15 to 35 items correctly answered. The correct answer rate for the entire scale, on average, was 62.7% (SD = 28%). Further analysis of items showed that more than 30% of hospice nurses underestimated the patients' pain and they did not treat the pain in the correct way; they had an incorrect self-evaluation about their pain management knowledge. Results from stepwise regression showed that nurses with higher mean correct answer scores had attended more courses on pain education. From these results, we conclude that there are still significant knowledge deficits and erroneous beliefs that may hamper treatment of hospice patients in pain. The results of this study could be useful to institutions involved in the education and application of patient pain management.

Institutions:
(Bernardi M) Department of Pediatrics, Clinic of Pediatric Hematology Oncology, University of Padova, Padova, Italy. Matteoberna@alice.it
Database: PubMed

211. The pain of residents with terminal cancer in USA nursing homes: family members' perspectives.

Author(s): Berry PH

Source: International journal of palliative nursing; Jan 2007; vol. 13 (no. 1); p. 20-27

Publication Date: Jan 2007

Publication Type(s): Journal Article; Research Support, N.I.H., Extramural

DOI: http://dx.doi.org/10.12968/ijpn.2007.13.1.22777

ISSN: 1357-6321

Place of Publication: England

PubMedID: 17353847

Accession Number: 17353847

Available at International journal of palliative nursing - from MAG Online Library

Available at International journal of palliative nursing - from EBSCO (CINAHL Complete)

Abstract: Cancer pain continues to be poorly treated despite efforts aimed at improvement. This causes considerable distress to both patients and their families. The purpose of this research is to explore the perspectives of family members of nursing home residents with terminal cancer, about pain and pain management. Participants who believed their pain could be better managed viewed their family member's pain and illness differently than those who believed the pain could not be managed better. The family members who believed better pain management was possible often took on the role of advocate and saw to it that the pain was addressed. Those who believed that their relative did not have adequate pain relief, but felt better management was not possible, expressed no concerns about this aspect of their relatives' care; they could not separate their relatives' pain from their illness. The interpretation and presence of adequate pain management is critical for family members to construct meaning around their relative's pain. When family members do not believe that the pain can be managed any better, this acts as a barrier to the resident's access to adequate pain management.

Institutions:
(Berry PH) University of Utah College of Nursing, 10 South 2000 East, Salt Lake City, Utah, USA. patricia.berry@nurs.utah.edu

Database: PubMed

212. [Reading nursing Literature in English: new inputs for practicing nurses].

Author(s): von Klitzing W; Stoll H; Trachsel E; Aldorf K; Bernhard A; Eze G; Spirig R

Source: Pflege; Feb 2007; vol. 20 (no. 1); p. 41-47

Publication Date: Feb 2007

Publication Type(s): English Abstract; Journal Article

DOI: http://dx.doi.org/10.1024/1012-5302.20.1.41

ISSN: 1012-5302

Place of Publication: Switzerland

PubMedID: 17294375

Accession Number: 17294375

Abstract:A prerequisite to providing evidence-based care is the ability to comprehend the nursing research literature, most of which is published in English. To facilitate this understanding, a course on "reading the research literature for evidence-based practice in English" was developed by an interdisciplinary team for staff nurses at the University Hospital Basel. The pilot course was offered to nurses who specialized in cancer care. It was led by the oncology Advanced Practice Nurse (APN) from the Department of Medicine. Research articles focusing on the management of chronic illness and cancer pain management were assigned and read. The course consisted of ten 90 minute lessons. The evaluation was designed to address the following questions: 1. Did participation in the course improve the oncology related knowledge of the nurses? 2. Did participation in the course improve the nurses' English language skills? 3. At what level of difficulty did the nurse participants perceive the course to be? 4. Were course participants able to use their newly acquired knowledge to teach their nursing colleagues on the ward? The course evaluation demonstrated that the 15 participants significantly improved their oncology knowledge through this process but that their English skills did not improve. The participants were able to present lectures on their wards based on the course literature, which were positively evaluated by their colleagues and the APN course leader. The participants perceived the course as being sophisticated but also effective at demonstrating the use of English-language research literature for one's own nursing practice.

Institutions:
(von Klitzing W) Institut für Pflegewissenschaft, Universität Basel, and Bereich Medizin, Universitätsspital Basel, Germany.

Database: PubMed

213. Can patient coaching reduce racial/ethnic disparities in cancer pain control? Secondary analysis of a randomized controlled trial.

Author(s): Kalauokalani D; Franks P; Oliver JW; Meyers FJ; Kravitz RL

Source: Pain medicine (Malden, Mass.); 2007; vol. 8 (no. 1); p. 17-24

Publication Date: 2007

Publication Type(s): Journal Article; Randomized Controlled Trial; Research Support, Non-U.S. Gov't

DOI: http://dx.doi.org/10.1111/j.1526-4637.2007.00170.x

ISSN: 1526-2375

Place of Publication: England

PubMedID: 17244100

Accession Number: 17244100

Available at Pain medicine (Malden, Mass.) - from EBSCO (MEDLINE Complete)

Available at Pain medicine (Malden, Mass.) - from Unpaywall

Abstract:PURPOSE: Minority patients with cancer experience worse control of their pain than do their white counterparts. This disparity may, in part, reflect more miscommunication between minority patients and their physicians. Therefore, we examined whether patient coaching could reduce disparities in pain control in a secondary analysis of a randomized controlled trial.METHODS: Sixty-seven English-

speaking adult cancer outpatients, including 15 minorities, with moderate pain over the prior 2 weeks were randomly assigned to the experimental (N = 34) or control group (N = 33). Experimental patients received a 20-minute individualized education and coaching session to increase knowledge of pain self-management, to redress personal misconceptions about pain treatment, and to rehearse an individually scripted patient-physician dialog about pain control. The control group received standardized information on controlling pain. Data on average pain (0-10 scale) were collected at enrollment and 2-week follow-up.RESULTS: At enrollment, minority patients had significantly more pain than their white counterparts (6.0 vs 5.0, P = 0.05). At follow-up, minorities in the control group continued to have more pain (6.4 vs 4.7, P = 0.01), whereas in the experimental group, disparities were eliminated (4.0 vs 4.3, P = 0.71). The effect of the intervention on reducing disparities was significant (P = 0.04).CONCLUSIONS: Patient coaching offers promise as a means of reducing racial/ethnic disparities in pain control. Larger studies are needed to validate these findings and to explore possible mechanisms.

Institutions:
(Kalauokalani D) Department of Anesthesiology and Pain Medicine, Division of Pain Medicine, University of California, Davis, California, USA. dkalauokalani@ucdavis.edu
Database: PubMed

214. Pain issues from the palliative perspective: a survey among doctors in Hospital Melaka.

Author(s): Taye GA

Source: The Medical journal of Malaysia; Oct 2006; vol. 61 (no. 4); p. 405-409

Publication Date: Oct 2006

Publication Type(s): Journal Article

ISSN: 0300-5283

Place of Publication: Malaysia

PubMedID: 17243516

Accession Number: 17243516

Abstract: This survey was intended to gauge the management of pain in palliative cancer patients by the doctors in Melaka Hospital. It also sought to identify possible barriers to adequate pain management among doctors and gauge their response to the adequacy of medical school teaching on cancer pain issues. A 39 item survey was used to cover the issues involved. Overall, the doctors displayed a lack of systematic approach to cancer pain management with inadequate knowledge of analgesia handling. Medical school exposure to cancer pain issues was lacking. Formulation of accepted clinical practice guidelines and new education strategies can improve cancer pain management.

Institutions:
(Taye GA) Palliative Care Unit and Department of Medicine, Hospital Melaka, Jalan Mufti Haji Khalil, 75499, Melaka.
Database: PubMed

215. Perioperative and intraoperative pain and anesthetic care of the chronic pain and cancer pain patient receiving chronic opioid therapy.

Author(s): Rozen D; Grass GW

Source: Pain practice : the official journal of World Institute of Pain; Mar 2005; vol. 5 (no. 1); p. 18-32

Publication Date: Mar 2005

Publication Type(s): Journal Article

DOI: http://dx.doi.org/10.1111/j.1533-2500.2005.05104.x

ISSN: 1533-2500

Place of Publication: United States

PubMedID: 17156114

Accession Number: 17156114

Available at Pain practice : the official journal of World Institute of Pain - from Wiley Online Library Medicine and Nursing Collection 2020

Available at Pain practice : the official journal of World Institute of Pain - from EBSCO (Psychology and Behavioral Sciences Collection)

Abstract: The expanding role of the anesthesiologist as a "perioperative physician" places ever-increasing demands upon his or her clinical skills and knowledge. One area of growing concern for the anesthesiologist involves the perioperative assessment and management of the opioid-tolerant chronic pain patient. Opioids occupy a position of unsurpassed clinical utility for the treatment of many types of painful conditions. Coupled with noticeable shifts in physician attitudes that have occurred in recent years regarding the use of opioids for the treatment of benign and malignancy-related pain, many more patients are presenting for surgical procedures who are opioid tolerant. It is important therefore that the practicing anesthesiologist become familiar with the currently available opioid formulations, including drug interactions and side effects, in order to better plan the patient's perioperative anesthetic needs and management. Unfortunately, there is a lack of scientifically rigorous studies in this important area, and most of the information must be derived from anecdotal reports and personal experience of anesthesiologists working in this field. In this review, we shall discuss some aspects of current chronic pain management, the newer forms of opioid administration which may be unfamiliar to the anesthesiologist, as well as clinical aspects of opioid use and tolerance including the impact it may have on perioperative anesthetic management.

Institutions:
(Rozen D) Department of Anesthesiology and Pain Medicine, Mount Sinai Medical Center, New York 10029-6574, USA. dimarozen@hotmail.com
Database: PubMed

216. Clinicians' practice and attitudes toward cancer pain management in Korea.

Author(s): Jeon YS; Kim HK; Cleeland CS; Wang XS

Source: Supportive care in cancer : official journal of the Multinational Association of Supportive Care in Cancer; May 2007; vol. 15 (no. 5); p. 463-469

Publication Date: May 2007

Publication Type(s): Journal Article; Multicenter Study; Research Support, Non-U.S. Gov't

DOI: http://dx.doi.org/10.1007/s00520-006-0183-x

ISSN: 0941-4355

Place of Publication: Germany

PubMedID: 17115200

Accession Number: 17115200

Available at Supportive care in cancer : official journal of the Multinational Association of Supportive Care in Cancer - from SpringerLink

Available at Supportive care in cancer : official journal of the Multinational Association of Supportive Care in Cancer - from EBSCO (MEDLINE Complete)

Available at Supportive care in cancer : official journal of the Multinational Association of Supportive Care in Cancer - from ProQuest (MEDLINE with Full Text) - NHS Version

Available at Supportive care in cancer : official journal of the Multinational Association of Supportive Care in Cancer - from ProQuest (Health Research Premium) - NHS Version

Available at Supportive care in cancer : official journal of the Multinational Association of Supportive Care in Cancer - from David Adams Library Journals Collection Local Print Collection [location] : David Adams Library.

Abstract:OBJECTIVES: The purposes of this study were to assess clinicians' (n = 250) current practices and attitudes about cancer pain management and to identify perceived concerns about and barriers to pain control in urban cancer-treatment settings in Korea.MATERIALS AND METHODS: Survey data (20 items) were collected either by mail or interview. Approximately 40% of the sample were nurses. More medical clinicians than surgical clinicians had more than 5 years of experience with cancer care (43 vs 31%) and committed more than 30% of their working hours to palliation (61 vs 19%). Significantly more medical clinicians claimed to be somewhat more or much more liberal than their professional peers in using analgesics compared with surgical clinicians (54 vs 35%). The liberal sample was more likely to be male (OR = 3.3, p < 0.001) and to be more experienced with cancer care (OR = 1.2, p < 0.001). Medical clinicians also reported more adequate pain-management training and a more proactive approach to assessing and treating pain.RESULTS: Overall, the greatest concerns regarding opioid use were safety, side effects, and fear of addiction. Inadequate pain assessment and lack of staff knowledge and time were identified as barriers to pain management. Unrealistic expectations and denial from both patient and family were the most troublesome issues for delivery of care to dying patients. This study suggests a more conservative attitude toward cancer pain management in Korea than in other countries surveyed in a similar manner.CONCLUSION: A combination of routine professional education and dissemination of guidelines is needed to bring about significant improvement in cancer pain control in Korea.

Institutions:

(Jeon YS) Department of Anesthesiology and Pain Medicine, Saint Vincent's Hospital, The Catholic University of Korea, 93-6 Chi-Dong, Paldal-Gu, Suwon, South Korea.
Database: PubMed

217. Knowledge and attitudes about cancer pain management: a national survey of Italian oncology nurses.

Author(s): Bernardi M; Catania G; Lambert A; Tridello G; Luzzani M

Source: European journal of oncology nursing : the official journal of European Oncology Nursing Society; Jul 2007; vol. 11 (no. 3); p. 272-279

Publication Date: Jul 2007

Publication Type(s): Journal Article; Research Support, Non-U.S. Gov't

DOI: http://dx.doi.org/10.1016/j.ejon.2006.09.003

ISSN: 1462-3889

Place of Publication: Scotland

PubMedID: 17112781

Accession Number: 17112781

Available at European journal of oncology nursing : the official journal of European Oncology Nursing Society - from David Adams Library Journals Collection Local Print Collection [location] : David Adams Library.

Abstract: The purpose of this study was to obtain information about the knowledge and attitudes of Italian oncology nurses concerning cancer pain management and to determine the predictors of nurses' pain management knowledge. The study was a nationwide descriptive survey and included 287 nurses in Italy from 21 oncology wards in the north, center and south of Italy. The Nurses' Knowledge and Attitudes Survey (Italian version) and a background information form were used to collect the data. Knowledge and attitudes regarding cancer pain were the main research variables. Among the 39 pain knowledge questions assessed, the mean number of correctly answered question was 21.4 (SD=5.5), with a range of 6-35. The correct answer rate for the entire scale, on average, was 55% (SD=25.9). Further analysis of items showed that more than 50% of oncology nurses underestimated the patients' pain and they did not treat it in the correct way; they also had an incorrect self-evaluation about their pain management knowledge. Results from stepwise regression showed that nurses with higher mean correct answer scores had attended more courses about pain education. There are still significant knowledge deficits and erroneous beliefs that may hamper treatment of oncology patients in pain. The results of this study could be useful to institutions involved in patient care and teaching of pain management.

Institutions:
(Bernardi M) Dipartimento di Pediatria, Oncoematologia Pediatrica, AO e Università Padova, Italy. matteoberna@alice.it
Database: PubMed

218. Structural visualization of expert nursing: Cancer pain management.

Author(s): Watanabe C; Uchiyama M; Takahashi M; Sato E; Tsuru S; Dannoue H

Source: Studies in health technology and informatics; 2006; vol. 122 ; p. 935

Publication Date: 2006

Publication Type(s): Journal Article

ISSN: 0926-9630

Place of Publication: Netherlands

PubMedID: 17102475

Accession Number: 17102475

Available at Studies in health technology and informatics - from EBSCO (MEDLINE Complete)

Abstract: Nurses' knowledge and making decisions in Cancer Pain Management contribute much to the improvement of cancer patients' QOL. Based on the practice of a expert nurse involved in Cancer Pain Management, we have developed the algorithm, which clarified that nurses implement much of observations or complicated decision-makings in Cancer Pain Manage Hereafter it is a significant challenge to examine its consistency and validity to develop its system.

Institutions:
(Watanabe C) Section of Nurse Administration, St. Luke's International Hospital, Tokyo, Japan. chiwa@luke.or.jp
Database: PubMed

219. The measurement of pain from metastatic bone disease: capturing the patient's experience.

Author(s): Cleeland CS

Source: Clinical cancer research : an official journal of the American Association for Cancer Research; Oct 2006; vol. 12 (no. 20 Pt 2); p. 6236s-6242s

Publication Date: Oct 2006

Publication Type(s): Journal Article; Research Support, N.I.H., Extramural; Research Support, Non-U.S. Gov't; Review

DOI: http://dx.doi.org/10.1158/1078-0432.CCR-06-0988

ISSN: 1078-0432

Place of Publication: United States

PubMedID: 17062707

Accession Number: 17062707

Available at Clinical cancer research : an official journal of the American Association for Cancer Research - from HighWire - Free Full Text

Available at Clinical cancer research : an official journal of the American Association for Cancer Research - from Unpaywall

Abstract: Pain is prevalent for large numbers of patients with metastatic cancer, and this pain is often due to bone metastases. Despite the availability of effective pain treatments and various pain management guidelines, multiple studies document the undertreatment of pain in patients with cancer. The most frequently identified barriers to appropriate pain management are physician underestimation of the patient's pain,

inadequate pain assessment, and patient reluctance to report pain. A first step toward reducing inadequate pain control in clinical practice is adequate assessment of cancer pain using validated pain measurement instruments. Using treatment for bone pain as an example, we discuss how patient satisfaction measures and self-report measures of pain and other symptoms, symptom interference with patient function, and related adverse effects can be combined with appropriate trial design to provide the information needed to choose the best possible treatment for bone pain. Symptom assessment needs to be done via standardized questionnaires, administered at appropriate times during the trial. The Brief Pain Inventory uses 11-point numerical rating scales to measure both pain severity and the resulting functional interference caused by pain. These severity and interference ratings can be further categorized as mild, moderate, or severe, as required to implement most pain management guidelines and define outcome expectations for clinical trials. Consistent pain measurement and standardized recording of analgesic use across clinical trials would enhance comparability and increase the chances that treatment decisions for management of bone pain could be evidence based. These and other considerations in the design of clinical trials are discussed.

Institutions:
(Cleeland CS) Department of Symptom Research, The University of Texas M.D. Anderson Cancer Center, Houston, Texas 77030, USA. ccleeland@mdanderson.org
Database: PubMed

220. What doctors know about cancer pain management: an exploratory study in Sarawak, Malaysia.

Author(s): Devi BC; Tang TS; Corbex M

Source: Journal of pain & palliative care pharmacotherapy; 2006; vol. 20 (no. 2); p. 15-22

Publication Date: 2006

Publication Type(s): Journal Article

ISSN: 1536-0288

Place of Publication: England

PubMedID: 16702132

Accession Number: 16702132

Available at Journal of pain & palliative care pharmacotherapy - from EBSCO (MEDLINE Complete)

Abstract: Effective cancer pain management is influenced by the attitudes and knowledge of treating physicians. A survey was conducted among the total population of government hospital doctors of Sarawak to study the barriers to cancer pain management. Two hundred and fifty-three respondents (83%) completed the survey. The study results highlight that knowledge about cancer pain management was low and barriers to morphine prescription were high. A majority of doctors were deterred from using morphine because of fear of addiction (36.5%) and respiratory depression (53.1%). Only 16.2% of the doctors chose the oral mode of administration to treat pain, furthermore 25% prescribed morphine on "PRN" basis. Doctors with undergraduate study in oncology consistently answered better suggesting that the situation can be improved by education. This study showed that

barriers to morphine prescription and knowledge deficit amongst government doctors in Sarawak are strong but similar to those reported in western countries few years ago.

Institutions:
(Devi BC) Department of Radiotherapy & Oncology, Sarawak General Hsopital, 93586 Kuching, Sarawak, Malaysia. devina@pc.jaring.my
Database: PubMed

221. [Essentials for transition of palliative care patients to palliative home care and for management of their cancer pain].

Author(s): Koshikawa T; Shimoyama N

Source: Gan to kagaku ryoho. Cancer & chemotherapy; May 2006; vol. 33 (no. 5); p. 611-615

Publication Date: May 2006

Publication Type(s): English Abstract; Journal Article

ISSN: 0385-0684

Place of Publication: Japan

PubMedID: 16685158

Accession Number: 16685158

Abstract: Multi-disciplinary team work among visiting doctors, nurses, care managers and pharmacists located close to the patient's home is essential for smooth transition of a palliative care patient from hospital care to palliative home care and should be set up prior to the patient's discharge from the hospital. Palliative home care physicians should have knowledge of the fundamental support by the government to spare excessive cost to the patients. As for cancer pain management, opioid-centered analgesic therapies have lead to better quality home care for patients. In Japan, although oxycodone SRs and fentanyl patches are available besides morphine, there is no rescue opioid other than morphine. On the other hand, some cancer pain refractory to opioids such as neuropathic cancer pain should be carefully treated by adjuvant analgesics in conjunction with non-pharmacological treatments.

Institutions:
(Koshikawa T) Koshikawa Hospital.
Database: PubMed

222. The prescription of opioid analgesics to terminal cancer patients: impact of physicians' general attitudes and contextual factors.

Author(s): Peretti-Watel P; Bendiane MK; Obadia Y; Favre R; Lapiana JM; Moatti JP; South-Eastern France Palliative Care Group

Source: Palliative & supportive care; Dec 2003; vol. 1 (no. 4); p. 345-352

Publication Date: Dec 2003

Publication Type(s): Journal Article; Research Support, Non-U.S. Gov't

ISSN: 1478-9515

Place of Publication: England

PubMedID: 16594224

Accession Number: 16594224

Available at Palliative & supportive care - from Cambridge Journals Online

Available at Palliative & supportive care - from ProQuest (MEDLINE with Full Text) - NHS Version

Available at Palliative & supportive care - from ProQuest (Health Research Premium) - NHS Version

Available at Palliative & supportive care - from David Adams Library Journals Collection Local Print Collection [location] : David Adams Library.

Abstract:OBJECTIVE: This study aimed to examine factors associated with the prescription of opioid analgesics to terminal cancer patients, including physicians' general attitudes toward morphine and contextual factors.METHODS: A survey was conducted among a sample of French general practitioners (GPs) and oncologists. Respondents were asked to describe the last three terminally ill patients they had followed up to death.RESULTS: Overall, 526 GPs and oncologists (global response rate: 57%) described 1,082 cancer patients, among whom 85.4% received opioid analgesics. Among other significant predictors (patient age, cancer type, family assistance), this prescription was less frequent for female patients followed by male physicians (OR = 0.53), and more frequent for patients followed by physicians trained in palliative care (OR = 2.70). On the other hand, physicians' attitudes toward morphine were not associated with prescription of morphine and other opioid analgesics.SIGNIFICANCE OF RESULTS: Although nonprescription of opioid analgesics is only a crude proxy measure for undertreatment of cancer pain, our findings suggest the need to develop training in palliative care in order to standardize practices among GPs and specialists. Our results also highlight the necessity to study pain assessment as an interaction between the physician and the patient, and to consider patients' and physicians' respective genders as a key variable within this interaction.

Institutions:
(Peretti-Watel P) Regional Centre for Disease Control of South-Eastern France, Marseilles. peretti@marseille.inserm.fr; (Bendiane MK) South-Eastern France Palliative Care Group
(Peretti-Watel P) Regional Centre for Disease Control of South-Eastern France, Marseilles. peretti@marseille.inserm.fr; (Bendiane MK) South-Eastern France Palliative Care Group

Database: PubMed

223. A randomized controlled trial of an educational intervention on Hellenic nursing staff's knowledge and attitudes on cancer pain management.

Author(s): Patiraki EI; Papathanassoglou ED; Tafas C; Akarepi V; Katsaragakis SG; Kampitsi A; Lemonidou C

Source: European journal of oncology nursing : the official journal of European Oncology Nursing Society; Dec 2006; vol. 10 (no. 5); p. 337-352

Publication Date: Dec 2006

Publication Type(s): Journal Article; Randomized Controlled Trial; Research Support, Non-U.S. Gov't
DOI: http://dx.doi.org/10.1016/j.ejon.2005.07.006
ISSN: 1462-3889
Place of Publication: Scotland
PubMedID: 16246621
Accession Number: 16246621

Available at European journal of oncology nursing : the official journal of European Oncology Nursing Society - from David Adams Library Journals Collection Local Print Collection [location] : David Adams Library.

Abstract: The purpose of this randomized controlled study was to explore the effectiveness of an educational intervention on nurses' attitudes and knowledge regarding pain management and to explore associations with nurses' characteristics. A four Solomon group experimental design was employed to assess the effect of the intervention and potential effects of pre-intervention testing. One hundred and twelve nurses were randomized to two intervention and two control groups. The intervention was based on viewing a series of educational videotapes and case scenarios. The Validated Hellenic version of the Nurses Knowledge and Attitudes Survey Regarding Pain (GV-NKASRP) was used. Pre-intervention scores revealed various limitations in regard to pain assessment and management. At the pre-test, the average number of correct answers was 17.58+/-7.58 (45.1%+/-19.3% of total questions). Pre-intervention scores differed significantly among participants with different educational backgrounds (P < 0.0001). A significant effect of pain education on total knowledge scores as well as regarding specific questions was detected. Intervention group participants provided 6.11+/-5.55 additional correct answers (15.66%+/-14.23% improvement, P < 0.0001), and they exhibited significantly improved post-test scores compared to controls (26.49+/-5.24 vs. 18.75+/-4.48; P < 0.0001). A potential negative effect of pre-test on knowledge gain for specific items and for total scores was detected. These findings suggest low pre-test knowledge scores among Hellenic oncology nurses and a significant effect of the intervention.

Institutions:
(Patiraki EI) University of Athens School of Nursing, Athens, Hellas, Greece. epatiraki@nurs.uoa.gr
Database: PubMed

224. Home care nurses' perceptions of control over cancer pain.

Author(s): Vallerand AH; Anthony M; Saunders MM

Source: Home healthcare nurse; Oct 2005; vol. 23 (no. 10); p. 647-652
Publication Date: Oct 2005
Publication Type(s): Journal Article; Multicenter Study; Research Support, N.I.H., Extramural
DOI: http://dx.doi.org/10.1097/00004045-200510000-00009
ISSN: 0884-741X
Place of Publication: United States

PubMedID: 16217214

Accession Number: 16217214

Abstract: This qualitative study examined home care nurses' perceptions of control over cancer pain. Four major themes emerged: Being heard, feeling invisible in the pain management process; not knowing, a need for pain education; control through advocacy; and patient-related barriers to optimal pain management. This study documents the need for continued education in pain management and communication skills for home care nurses.

Institutions:
(Vallerand AH) College of Nursing, Wayne State University, Detroit, Michigan 48202, USA. April.Vallerand@wayne.edu
Database: PubMed

225. Cancer pain in palliative care: why is management so difficult?

Author(s): Hemming L; Maher D

Source: British journal of community nursing; Aug 2005; vol. 10 (no. 8); p. 362-367

Publication Date: Aug 2005

Publication Type(s): Journal Article; Review

DOI: http://dx.doi.org/10.12968/bjcn.2005.10.8.18574

ISSN: 1462-4753

Place of Publication: England

PubMedID: 16116395

Accession Number: 16116395

Available at British journal of community nursing - from MAG Online Library

Available at British journal of community nursing - from EBSCO (CINAHL Complete)

Abstract: Pain is the major source of anxiety and distress at the end of life, particularly in cases of end-stage cancer. However, pain management is not always effective or effectively implemented. This article identifies several barriers to effective pain relief in terminal cancer--the complexity of pain; difficulties in physical, emotional and spiritual assessment; difficulties in the delivery of medication--that challenge the skills of all professionals involved in palliative care. There are no simple answers, but awareness of the breadth of the issues may help focus nurses' minds on the patient in every encounter.

Institutions:
(Hemming L) Department of Nursing and Midwifery, University of Hertfordshire. l.hemming@herts.ac.uk
Database: PubMed

226. Alternatives in cancer pain treatment: the application of chiropractic care.

Author(s): Evans RC; Rosner AL

Source: Seminars in oncology nursing; Aug 2005; vol. 21 (no. 3); p. 184-189

Publication Date: Aug 2005

Publication Type(s): Journal Article; Review
DOI: http://dx.doi.org/10.1016/j.soncn.2005.04.007
ISSN: 0749-2081
Place of Publication: United States
PubMedID: 16092806
Accession Number: 16092806

Available at Seminars in oncology nursing - from David Adams Library Journals Collection Local Print Collection [location] : David Adams Library.

Abstract:OBJECTIVES: To review written resources disclosing reliable facts and knowledge in chiropractic services in cancer pain management.DATA SOURCES: Conventional and biomedical and complementary and alternative medicine journals, electronic media, full text databases, electronic resources, books in print, and newsletters.CONCLUSION: The judicial use of chiropractic services in cancer patients appears to offer many economical and effective strategies for reducing the pain and suffering of cancer patients, as well as providing the potential to improve patient health overall.IMPLICATIONS FOR NURSING PRACTICE: Clinicians should assess and support the use of chiropractic services in cancer patients. Chiropractic is one of the leading alternatives to standard medical treatment in cancer pain management.

Institutions:
(Evans RC) Foundation for Chiropractic Education and Research, Norwalk, IA, USA. evansceo@icpc.net
Database: PubMed

227. Palliative care. Some organisational considerations.

Author(s): Welshman A

Source: Minerva anestesiologica; 2005; vol. 71 (no. 7-8); p. 439-443
Publication Date: 2005
Publication Type(s): Journal Article; Review
ISSN: 0375-9393
Place of Publication: Italy
PubMedID: 16012417
Accession Number: 16012417

Abstract: Managing pain effectively is one of the biggest challenges in medicine, let alone when dealing with the dying patient and his family. For palliative care specialists this is a daily challenge. However, "To cure when possible, to give comfort always" is an empty credo if physicians don't use every weapon in the medical arsenal to relieve the suffering caused by chronic pain. It's of course the opioids: morphine, heroin, their synthetic derivatives and other narcotics, a class of medications that conjure up visions of drug addiction and narcotic squads. To say that opioids are stigmatised by such allusions is putting it mildly. An unhealthy proportion of doctors and patients alike are afraid to have anything to do with them, even in when facing their final stages of life. This is particularly so in the Mediterranean society. It is here in Italy that an effort must be made to educate both

physicians and the general public, an arduous task to change a long standing belief which requires a quick cultural turn around. Those who refuse opioids because they are afraid of addiction, and the doctors who refuse to prescribe them out of fear or pure unwillingness to address an apprehensive attitude on behalf of his patient, need to be better informed. Most misconceptions about opioids have to do with terminology, because words like "morphine, addiction, dependency" and "tolerance" mean entirely different things in popular and medical parlance. Add to this the perceptions and attitudes the patient can have with this terminology which then can have a profound effect on the success or failure of a pain control programme. In fact, most people think that medication such as morphine are only for people who are dying and as a consequence is synonymous with death itself. Is this why Italian physicians are not prescribing morphine even though great efforts have been made recently by the Health Ministry to facilitate prescribing laws and costs? It is worthy of serious consideration. Another important issue faced daily by palliative care physicians is the broad number of chronic conditions which could make use of opioids. Severe cancer pain is the most obvious example of an appropriate use of opioids, but hardly the only one. The North American Chronic Pain Association of Canada (NACPAC) advocates the use of opioids for a wide range of conditions causing severe chronic pain, including lower back pain, inflammatory bowel disease, migraines, AIDS, multiple sclerosis and arthritis. Concerns regarding under treatment of chronic pain have captured the attention of patient advocacy groups, policy makers and scientific organisations. Misconceptions of opioid laws, negative social stigma and lack of valid prescribing alternatives to overcome this, together with paucity of formal provider education confound the issue. Much education needs to be done before opioids will be seen as a safe and reasonable treatment for chronic pain here in Italy.

Institutions:
(Welshman A) Sue Ryder Foundation, Rome, Italy. posta@sueryder.it
Database: PubMed

228. Evaluation of education in palliative care: determining the effects on nurses' knowledge and attitudes.

Author(s): McClement SE; Care D; Dean R; Cheang M

Source: Journal of palliative care; 2005; vol. 21 (no. 1); p. 44-48

Publication Date: 2005

Publication Type(s): Journal Article

ISSN: 0825-8597

Place of Publication: United States

PubMedID: 15895549

Accession Number: 15895549

Available at Journal of palliative care - from ProQuest (MEDLINE with Full Text) - NHS Version

Available at Journal of palliative care - from ProQuest (Health Research Premium) - NHS Version

Available at Journal of palliative care - from David Adams Library Journals Collection Local Print Collection [location] : David Adams Library.

Abstract: Our study was an evaluation of a pilot course in palliative nursing care designed to improve practising nurses' knowledge regarding cancer pain management and attitudes toward care of the dying patient. The course involved 12 weeks (36 hours) of systematic classroom instruction for registered nurses, and a one-day observational experience on a designated palliative care unit. A total of 16 nurses participated in the study. A repeated measures design was used to evaluate the effectiveness of the course. Attitudes toward care of the dying patient and his/her family were also evaluated. Results showed that nurses' attitudes improved significantly from Time 1 (first day of course) to Time 2 (last day of course) (p=0.0007), and that this improvement was maintained at Time 3 (three months later) (p=0.064). The observational experience on a palliative care unit held in conjunction with the course was reported to be helpful to students in consolidating theoretical instruction.

Institutions:
(McClement SE) Faculty of Nursing, University of Manitoba, Winnipeg, Canada.
Database: PubMed

229. Nurses' willingness to maximize opioid analgesia for severe cancer pain, and its predictor.

Author(s): Chang YJ; Yun YH; Park SM; Lee SW; Park HA; Ro YJ; Huh BY

Source: Supportive care in cancer : official journal of the Multinational Association of Supportive Care in Cancer; Sep 2005; vol. 13 (no. 9); p. 743-751

Publication Date: Sep 2005

Publication Type(s): Comparative Study; Journal Article; Research Support, Non-U.S. Gov't

DOI: http://dx.doi.org/10.1007/s00520-005-0791-x

ISSN: 0941-4355

Place of Publication: Germany

PubMedID: 15827729

Accession Number: 15827729

Available at Supportive care in cancer : official journal of the Multinational Association of Supportive Care in Cancer - from SpringerLink

Available at Supportive care in cancer : official journal of the Multinational Association of Supportive Care in Cancer - from ProQuest (MEDLINE with Full Text) - NHS Version

Available at Supportive care in cancer : official journal of the Multinational Association of Supportive Care in Cancer - from ProQuest (Health Research Premium) - NHS Version

Available at Supportive care in cancer : official journal of the Multinational Association of Supportive Care in Cancer - from David Adams Library Journals Collection Local Print Collection [location] : David Adams Library.

Abstract:GOALS OF WORK: The effectiveness of cancer pain management (CPM) is influenced by nurses' willingness to maximize opioid analgesia for severe cancer pain. The purposes of this study were to identify the willingness of nurses to provide maximum-dose opioids whenever needed for CPM and to determine its associated

predictors.METHODS: This multicenter study was conducted among the entire total of registered nurses in seven large hospitals in Korea. Its overall response rate was 41.6%, and the data from 930 who responded (40.1%) were analyzed. We utilized a three-step, multidimensional, multiple logistic regression to identify the predictors of nurses' willingness.MAIN RESULTS: Only 255 nurses (27.4%) indicated that they recommended the maximum dose of opioids whenever it was needed. The respondents who were more likely to recommend morphine showed the following characteristics: older nurses (odds ratio, OR, 1.57; confidence interval, CI, 1.13-2.19); they knew the effectiveness of opioids for CPM (OR 1.53; CI 1.06-2.20); rarely concerned about a patient's addiction to opioids (OR 2.16; CI 1.48-3.15), or to a family member's addiction (OR 1.81; CI 1.20-2.73); prior experience with pain assessment tools (OR 1.62; CI 1.11-2.37); practical experience caring for cancer patients with pain over 51% (OR 1.55; CI 1.09-2.19).CONCLUSIONS: Our multicenter study suggested that in order to improve nurses' willingness to recommend opioids liberally in CPM: (1) attitudes about fear of opioid addiction must be changed; (2) the efficiency of opioids in CPM must be taught; and (3) implementation of pain assessment tools must be undertaken.

Institutions:
(Chang YJ) Cancer Information Branch, Research Institute, National Cancer Center, Goyang, South Korea.

Database: PubMed

230. Physicians' knowledge of transdermal fentanyl.

Author(s): Welsh J; Reid A; Graham J; Curto J; MacLeod K; O'Neill C

Source: Palliative medicine; Jan 2005; vol. 19 (no. 1); p. 9-16

Publication Date: Jan 2005

Publication Type(s): Journal Article; Research Support, Non-U.S. Gov't

DOI: http://dx.doi.org/10.1191/0269216305pm971oa

ISSN: 0269-2163

Place of Publication: England

PubMedID: 15690863

Accession Number: 15690863

Available at Palliative medicine - from ProQuest (MEDLINE with Full Text) - NHS Version

Available at Palliative medicine - from ProQuest (Health Research Premium) - NHS Version

Abstract:BACKGROUND: Different opioids for use in the control of moderate to severe cancer pain have become widely available. More recently, sophisticated formulations such as transdermal patches have been developed. One example, transdermal fentanyl (TF), has characteristic pharmacokinetics and along with the other opioids, equivalency conversions are often made when changing from or to other opioids.AIM OF STUDY: To explore the knowledge of general practitioners (GPs), hospital consultants and oncologists about the pharmacology and use of TF in the management of moderate/severe cancer pain.METHOD: During 2001 and 2002 a questionnaire survey was carried out. A randomized selection of GPs,

hospital consultants and oncologists (n=1167) from the UK and Ireland were sent a questionnaire and 576 (49%) were returned and evaluated.RESULTS: The results show doctors who had previously prescribed TF are more confident and knowledgeable about the indications and pharmacology of TF than doctors who had never prescribed TF. Overall knowledge and confidence in using TF was poor.

Institutions:
(Welsh J) University of Glasgow and Beatson Oncology Centre, Western Infirmary, Glasgow.

Database: PubMed

231. A survey of cancer pain management knowledge and attitudes of British Columbian physicians.

Author(s): Gallagher R; Hawley P; Yeomans W

Source: Pain research & management; 2004; vol. 9 (no. 4); p. 188-194

Publication Date: 2004

Publication Type(s): Journal Article; Research Support, Non-U.S. Gov't

DOI: http://dx.doi.org/10.1155/2004/748685

ISSN: 1203-6765

Place of Publication: United States

PubMedID: 15605132

Accession Number: 15605132

Available at Pain research & management - from Hindawi Open Access Journals

Abstract:INTRODUCTION: There are many potential barriers to adequate cancer pain management, including lack of physician education and prescription monitoring programs. The authors surveyed physicians about their specific knowledge of pain management and the effects of the regulation of opioids on their prescribing practices.METHODS: A questionnaire was mailed out to British Columbia physicians who were likely to encounter cancer patients. The survey asked for physicians' opinions about College of Physicians and Surgeons of British Columbia regulation and other issues related to their prescribing practices, and assessed basic knowledge of cancer pain management.RESULTS: There was a 69% return rate with a total of 4618 evaluable responses. There was a significant difference among medical disciplines, years in practice, number of chronic pain patients seen and size of community of practice. The highest knowledge scores were achieved by oncologists and the lowest scores were from surgeons. Those who practiced in smaller communities had a higher average knowledge score. Those who felt their knowledge about cancer pain was inadequate scored lower than those who felt their knowledge was adequate. The questions most frequently answered incorrectly (or by "don't know") were those about equianalgesic dosing (68%) and adequate breakthrough dosing (45%), revealing knowledge deficiencies that would significantly impair a physician's ability to manage cancer pain.CONCLUSIONS: The details of opioid prescribing are crucial areas to target education for cancer pain management. The surveyed physicians accepted the need for regulation of opioid prescribing with very few being fearful of scrutiny from the College of Physicians and Surgeons of British Columbia. However, the inconvenience of the triplicate prescription pad was

more of a barrier to prescribing, it being of concern to 20% of respondents, particularly surgeons and medical specialists.

Institutions:
(Gallagher R) Division of Palliative Care, University of British Columbia, Vancouver. romayne@interchange.ubc.ca
Database: PubMed

232. The PRO-SELF pain control program improves patients' knowledge of cancer pain management.

Author(s): Kim JE; Dodd M; West C; Paul S; Facione N; Schumacher K; Tripathy D; Koo P; Miaskowski C

Source: Oncology nursing forum; Nov 2004; vol. 31 (no. 6); p. 1137-1143

Publication Date: Nov 2004

Publication Type(s): Clinical Trial; Journal Article; Multicenter Study; Randomized Controlled Trial; Research Support, N.I.H., Extramural; Research Support, Non-U.S. Gov't; Research Support, U.S. Gov't, P.H.S.

DOI: http://dx.doi.org/10.1188/04.ONF.1137-1143

ISSN: 1538-0688

Place of Publication: United States

PubMedID: 15547636

Accession Number: 15547636

Available at Oncology nursing forum - from Ovid (Journals @ Ovid)

Available at Oncology nursing forum - from EBSCO (CINAHL Complete)

Available at Oncology nursing forum - from David Adams Library Journals Collection Local Print Collection [location] : David Adams Library.

Abstract:PURPOSE/OBJECTIVES: To evaluate the effectiveness of a psychoeducational program (i.e., PRO-SELF Pain Control Program) compared to standard care in increasing patients' knowledge regarding cancer pain management.DESIGN: Randomized clinical trial.SETTING: Seven outpatient settings in northern California.SAMPLE: 174 outpatients with cancer and pain from bone metastasis.METHODS: Following randomization into either the PRO-SELF or standard care group, patients completed the Pain Experience Scale (PES) prior to and at the completion of the intervention.MAIN RESEARCH VARIABLES: Total and individual item scores on the PES.FINDINGS: Total PES knowledge scores increased significantly in the PRO-SELF group (21%) compared to the standard care group (0.5%). Significant improvements in knowledge scores for patients in the PRO-SELF group were found on five of the nine PES items when compared to baseline scores.CONCLUSIONS: The PRO-SELF Pain Control Program was an effective approach to increase patients' knowledge of cancer pain management.IMPLICATIONS FOR NURSING: The use of a structured paper-and-pencil questionnaire, such as the PES, as part of a psychoeducational intervention provides an effective foundation for patient education in cancer pain management. Oncology nurses can use patients' responses to this type of questionnaire to individualize the teaching and to spend more time on the identified knowledge

deficits. This individualized approach to education about pain management may save staff time and improve patient outcomes.

Institutions:
(Kim JE) University of California Medical Center, San Francisco, CA, USA. chris.miaskowski@nursing.ucsf.edu
Database: PubMed

233. National Institutes of Health State-of-the-Science Conference Statement: Symptom management in cancer: pain, depression, and fatigue, July 15-17, 2002.

Author(s): Patrick DL; Ferketich SL; Frame PS; Harris JJ; Hendricks CB; Levin B; Link MP; Lustig C; McLaughlin J; Reid LD; Turrisi AT; Unützer J; Vernon SW; National Institutes of Health State-of-the-Science Panel

Source: Journal of the National Cancer Institute. Monographs; 2004 (no. 32); p. 9-16

Publication Date: 2004

Publication Type(s): Consensus Development Conference; Consensus Development Conference, NIH; Journal Article; Review

DOI: http://dx.doi.org/10.1093/jncimonographs/djg014

ISSN: 1052-6773

Place of Publication: United States

PubMedID: 15263035

Accession Number: 15263035

Available at Journal of the National Cancer Institute. Monographs - from Unpaywall

Abstract:BACKGROUND: Despite advances in early detection and effective treatment, cancer remains one of the most feared diseases. Among the most common side effects of cancer and treatments for cancer are pain, depression, and fatigue. Although research is producing increasingly hopeful insights into the causes and cures for cancer, efforts to manage the side effects of the disease and its treatments have not kept pace. The challenge that faces us is how to increase awareness of the importance of recognizing and actively addressing cancer-related distress. The National Institutes of Health (NIH) convened a State-of-the-Science Conference on Symptom Management in Cancer: Pain, Depression, and Fatigue to examine the current state of knowledge regarding the management of pain, depression, and fatigue in individuals with cancer and to identify directions for future research. Specifically, the conference examined how to identify individuals who are at risk for cancer-related pain, depression, and/or fatigue; what treatments work best to address these symptoms when they occur; and what is the best way to deliver interventions across the continuum of care. STATE-OF-THE-SCIENCE PROCESS: A non-advocate, non-Federal, 14-member panel of experts representing the fields of oncology, radiology, psychology, nursing, public health, social work, and epidemiology prepared the statement. In addition, 24 experts in medical oncology, geriatrics, pharmacology, psychology, and neurology presented data to the panel and to the conference audience during the first 1.5 days of the conference. The panel then prepared its statement, addressing the five predetermined questions and drawing on submitted literature, the speakers' presentations, and discussions held at the conference. The statement was presented to the conference audience, followed

by a press conference to allow the panel to respond to questions from the media. After its release at the conference, the draft statement was made available on the Internet. The panel's final statement is available at http://consensus.nih.gov.CONCLUSIONS: The panel concluded that the available evidence supports a variety of interventions for treating cancer patients' pain, depression, and fatigue. Clinicians should routinely use brief assessment tools to ask patients about pain, depression, and fatigue and to initiate evidence-based treatments. Assessment should include discussion about common symptoms experienced by cancer patients, and these discussions should continue over the duration of the illness. Impediments to effective symptom management in cancer patients can arise from different sources and interactions among providers, patients and their families, and the health care system. Numerous factors could interfere with adequate symptom management. Among these factors are incomplete effectiveness of some treatments, a lack of sufficient knowledge regarding effective treatment strategies, patient reluctance to report symptoms to caregivers, a belief that such symptoms are simply a part of the cancer experience that must be tolerated, and inadequate coverage and reimbursement for some treatments. Additional research is needed on the definition, occurrence, the treatment of pain, depression, and fatigue, alone and in combination, in adequately funded prospective studies. The panel also concluded that the state of the science in cancer symptom management should be reassessed periodically.

Institutions:
(Patrick DL) National Institutes of Health State-of-the-Science Panel
Database: PubMed

234. Improving cancer pain management by homecare nurses.

Author(s): Vallerand AH; Riley-Doucet C; Hasenau SM; Templin T

Source: Oncology nursing forum; Jul 2004; vol. 31 (no. 4); p. 809-816

Publication Date: Jul 2004

Publication Type(s): Clinical Trial; Comparative Study; Journal Article; Multicenter Study; Randomized Controlled Trial; Research Support, U.S. Gov't, P.H.S.

DOI: http://dx.doi.org/10.1188/04.ONF.809-816

ISSN: 1538-0688

Place of Publication: United States

PubMedID: 15252435

Accession Number: 15252435

Available at Oncology nursing forum - from Ovid (Journals @ Ovid)

Available at Oncology nursing forum - from EBSCO (CINAHL Complete)

Available at Oncology nursing forum - from David Adams Library Journals Collection Local Print Collection [location] : David Adams Library.

Abstract:PURPOSE/OBJECTIVES: To demonstrate the effects of a program, directed at homecare nurses, of structured educational interventions on the management of pain and opioid-related side effects in homecare patients with cancer.DESIGN: A longitudinal multilevel, randomized, controlled clinical trial.SETTING: Midwestern region in the United States.SAMPLE: 202 nurses caring

for patients with cancer recruited from homecare agencies.METHODS: The two-tiered educational program focused on basic and advanced pain management strategies, particularly in the area of pharmacologic options and assertive communication skills. Instruments used were the Nurses' Knowledge and Attitudes Survey Regarding Pain, the barriers questionnaire, perception of control over pain, and a demographic questionnaire.MAIN RESEARCH VARIABLES: Knowledge and attitudes about pain management, barriers to pain management, and perception of control over pain.FINDINGS: Nurses in the intervention group had a significant increase in their knowledge, a more positive attitude about pain management, fewer perceived barriers to pain management, and an increase in perceived control over pain compared to the nurses who did not receive the intervention.CONCLUSIONS: The educational program Power Over Pain has beneficial effects for homecare nurses caring for patients with cancer pain.IMPLICATIONS FOR NURSING: A need exists for homecare nurses to gain more insight into pain management strategies and enhance their advocacy skills to improve pain management for patients with cancer treated in the home.

Institutions:
(Vallerand AH) College of Nursing, Wayne State University, Detroit, MI, USA. april.vallerand@wayne.edu
Database: PubMed

235. A randomized controlled trial of a nurse-administered educational intervention for improving cancer pain management in ambulatory settings.

Author(s): Yates P; Edwards H; Nash R; Aranda S; Purdie D; Najman J; Skerman H; Walsh A

Source: Patient education and counseling; May 2004; vol. 53 (no. 2); p. 227-237

Publication Date: May 2004

Publication Type(s): Clinical Trial; Journal Article; Randomized Controlled Trial; Research Support, Non-U.S. Gov't

DOI: http://dx.doi.org/10.1016/S0738-3991(03)00165-4

ISSN: 0738-3991

Place of Publication: Ireland

PubMedID: 15140463

Accession Number: 15140463

Available at Patient education and counseling - from Unpaywall

Abstract: The persistence of negative attitudes towards cancer pain and its treatment suggests there is scope for identifying more effective pain education strategies. This randomized controlled trial involving 189 ambulatory cancer patients evaluated an educational intervention that aimed to optimize patients' ability to manage pain. One week post-intervention, patients receiving the pain management intervention (PMI) had a significantly greater increase in self-reported pain knowledge, perceived control over pain, and number of pain treatments recommended. Intervention group patients also demonstrated a greater reduction in willingness to tolerate pain, concerns about addiction and side effects, being a "good" patient, and tolerance to pain relieving medication. The results suggest that targeted educational interventions that utilize individualized instructional techniques

may alter cancer patient attitudes, which can potentially act as barriers to effective pain management.

Institutions:
(Yates P) Center for Health Research, Queensland University of Technology, Kelvin Grove Campus, Victoria Park Road, Kelvin Grove, Brisbane 4059, Australia. p.yates@qut.edu.au
Database: PubMed

236. Managing children's cancer pain in Morocco.

Author(s): McCarthy P; Chammas G; Wilimas J; Alaoui FM; Harif M

Source: Journal of nursing scholarship : an official publication of Sigma Theta Tau International Honor Society of Nursing; 2004; vol. 36 (no. 1); p. 11-15

Publication Date: 2004

Publication Type(s): Journal Article; Research Support, Non-U.S. Gov't

DOI: http://dx.doi.org/10.1111/j.1547-5069.2004.04005.x

ISSN: 1527-6546

Place of Publication: United States

PubMedID: 15098413

Accession Number: 15098413

Available at Journal of nursing scholarship : an official publication of Sigma Theta Tau International Honor Society of Nursing - from Wiley Online Library Medicine and Nursing Collection 2020

Available at Journal of nursing scholarship : an official publication of Sigma Theta Tau International Honor Society of Nursing - from ProQuest (MEDLINE with Full Text) - NHS Version

Available at Journal of nursing scholarship : an official publication of Sigma Theta Tau International Honor Society of Nursing - from ProQuest (Health Research Premium) - NHS Version

Abstract:PURPOSE: To identify issues in managing pain of children with cancer in the two pediatric oncology centers in Morocco.METHODS: Focus groups were conducted with pediatric oncology nurses and physicians.FINDINGS: Four themes were identified: (a) children's cancer pain is an overwhelming concern to the Moroccan nurses and physicians who participated in this study; (b) training and resources for children's cancer pain management are lacking in Morocco; (c) some impediments to pain relief were verbalized, such as a stoic approach to suffering and limited use of some drugs; and (d) a critical need exists for a comprehensive pain management approach for children with cancer in Morocco.CONCLUSIONS: This study elucidated issues in managing children's cancer pain in Morocco and increased knowledge of current practice issues. A program of policy research has been initiated with the aim of establishing guidelines for practice policies for managing children's cancer pain in Morocco.

Institutions:
(McCarthy P) Oncology Department, Children's Hospital of Eastern Ontario, Ottawa, Ontario, Canada. mccarthy-p@cheo.on.ca
Database: PubMed

237. Epidemiology of cancer pain and factors influencing poor pain control.

Author(s): Davis MP; Walsh D

Source: The American journal of hospice & palliative care; 2004; vol. 21 (no. 2); p. 137-142

Publication Date: 2004

Publication Type(s): Journal Article; Review

DOI: http://dx.doi.org/10.1177/104990910402100213

ISSN: 1049-9091

Place of Publication: United States

PubMedID: 15055515

Accession Number: 15055515

Abstract: Pain is one of the most commonly experienced and feared symptoms of advanced cancer. Most cancer patients experience pain, usually of moderate to severe intensity, and most also have a number of distinct pains. The most common type of pain is related to bone metastases. Neuropathic pain occurs in one-third of patients, alone, or as a mix of nociceptive and neuropathic pain. The failure to manage pain properly is due to several factors. In developing countries, it is likely to be related to geography and limited resources. Legal restrictions also present barriers. In developed countries, failure to manage pain properly is usually related to a "disease" rather than a "symptom" model of care, which minimizes symptom management. Other factors include lack of physician education and failure to follow existing guidelines. Patients fear addiction, drug tolerance, and side effects. Despite adequate resources, pain is still undertreated.

Institutions:
(Davis MP) The Harry R. Horvitz Center for Palliative Medicine, Cleveland Clinic Foundation, Cleveland, Ohio, USA.
Database: PubMed

238. Use of strong opioids for non-cancer pain in the community: a case study.

Author(s): Cowan DT; While A; Griffiths P

Source: British journal of community nursing; Feb 2004; vol. 9 (no. 2); p. 53-58

Publication Date: Feb 2004

Publication Type(s): Case Reports; Journal Article

DOI: http://dx.doi.org/10.12968/bjcn.2004.9.2.12418

ISSN: 1462-4753

Place of Publication: England

PubMedID: 15007281

Accession Number: 15007281

Available at British journal of community nursing - from MAG Online Library

Available at British journal of community nursing - from EBSCO (CINAHL Complete)

Abstract: The continued extension of prescribing rights among nurses may necessitate that effective pain management will require more involvement of nurses in the prescription of controlled drugs. The prescription of strong opioid analgesic drugs for chronic non-cancer pain (CNCP) is viewed as controversial. Misconceptions about opioid drugs fuel this controversy. This case study highlights the knowledge gap that exists between pain and addiction medicine and highlights the problems that CNCP patients treated in the community with opioid therapy may encounter. Community nurses are in an ideal position to be instrumental in identifying such vulnerable patients and ensuring that appropriate interventions are available.

Institutions:
(Cowan DT) Florence Nightingale School of Nursing and Midwifery, King's College London. david.t.cowan@kcl.ac.uk
Database: PubMed

239. Attitudes toward opioid use for chronic pain: a Canadian physician survey.

Author(s): Morley-Forster PK; Clark AJ; Speechley M; Moulin DE

Source: Pain research & management; 2003; vol. 8 (no. 4); p. 189-194

Publication Date: 2003

Publication Type(s): Comparative Study; Journal Article; Research Support, Non-U.S. Gov't

DOI: http://dx.doi.org/10.1155/2003/184247

ISSN: 1203-6765

Place of Publication: United States

PubMedID: 14679412

Accession Number: 14679412

Available at Pain research & management - from Hindawi Open Access Journals

Available at Pain research & management - from Unpaywall

Abstract:OBJECTIVES: To measure chronic pain patient volumes seen in primary care practice; to determine what medications physicians choose for the treatment of moderate to severe chronic pain; to identify barriers to the use of opioids in the treatment of chronic pain; and to assess physicians' attitudes toward the current management of chronic pain in Canada.DESIGN: A computer-assisted telephone survey of 100 regionally representative Canadian physicians with a defined interest in palliative care (PC, n=30) or noncancer pain (GP, n=70).SETTING: A survey was conducted by Ipsos-Reid in June 2001. Only physicians who met the eligibility criteria of having written 20 or more prescriptions for moderate to severe pain in the preceding four weeks or having devoted 20% of time to palliative care were eligible to participate.RESULTS: In one month, the average number of patients with moderate to severe chronic pain seen by PCs was 94.2; the average seen by GPs was 44.7. The pain experienced by 83.3% of GP patients was noncancer related. For chronic cancer pain, an opioid analgesic was the treatment of choice of 79% of physicians (48% preferred morphine, 21% codeine, 10% other). For moderate to severe chronic noncancer pain, opioids were the first-line treatment of only 32% of

physicians (16% preferred codeine, 16% major opioids) because a significant number preferred either non-steroidal anti-inflammatory drugs (29%) or acetaminophen (16%). Thirty-five per cent of GPs and 23% of PCs would never use opioids for noncancer pain, even when described as severe. Chronic pain was deemed by 68% of physicians to be inadequately managed. Almost 60% thought that pain management could be enhanced by improved physician education. Identified barriers to opioid use included addiction potential (37%) and side effects (25%). Seventeen per cent of GPs and 10% of PCs thought that regulatory sanctions limited opioid prescribing.CONCLUSIONS: Even among physicians experienced in chronic pain treatment, there is a reluctance to use opioids for severe nonmalignant pain. One-half of the survey participants believed that there was a need for improved physician education in pain management, including the use of opioids.

Institutions:
(Morley-Forster PK) University of Western Ontario Interdisciplinary Pain Program, St. Joseph's Health Care, London, Ontario, Canada. pat.morley-forster@sjhc.london.on.ca

Database: PubMed

240. Clinical decision making in pain management: Contributions of physician and patient characteristics to variations in practice.

Author(s): Green CR; Wheeler JR; LaPorte F

Source: The journal of pain; Feb 2003; vol. 4 (no. 1); p. 29-39

Publication Date: Feb 2003

Publication Type(s): Journal Article

DOI: http://dx.doi.org/10.1054/jpai.2003.5

ISSN: 1526-5900

Place of Publication: United States

PubMedID: 14622725

Accession Number: 14622725

Abstract: Differences in the quality of pain management may very well be due to physician characteristics and their treatment goals based on the type of pain or patient demographics. This study was done to (1) determine the role of physician characteristics in their goals and treatment of acute, cancer, and chronic pain and (2) provide an evaluation of the differences in physician pain management decision making due to patient characteristics and the type of pain being treated. A prospective cohort study of 368 Michigan physicians was done to determine their pain management knowledge, attitudes, and prescribing habits via study-specific multi-item mail survey. Nine clinical vignettes were used to examine potential differences in the physician's pain management based on the type of pain and patient demographic characteristics. The responses of the study group varied on the basis of the type of pain and gender of the patient. They were more likely to provide optimal treatment for men with acute postoperative or cancer pain. The physicians also reported lesser goals for relief of chronic pain when compared to acute and cancer pain. Lower goals for chronic pain relief may lead to the undertreatment of chronic pain. This study demonstrates that the provision of adequate pain management may be influenced by patient characteristics and physician variability.

Institutions:
(Green CR) University of Michigan Health System, University of Michigan, Ann Arbor, MI 48109, USA.
Database: PubMed

241. Evaluation of a cancer pain education module.

Author(s): Wilkes G; Lasch KE; Lee JC; Greenhill A; Chiri G

Source: Oncology nursing forum; 2003; vol. 30 (no. 6); p. 1037-1043

Publication Date: 2003

Publication Type(s): Evaluation Study; Journal Article; Research Support, U.S. Gov't, P.H.S.

DOI: http://dx.doi.org/10.1188/03.ONF.1037-1043

ISSN: 1538-0688

Place of Publication: United States

PubMedID: 14603361

Accession Number: 14603361

Available at Oncology nursing forum - from Ovid (Journals @ Ovid)

Available at Oncology nursing forum - from EBSCO (CINAHL Complete)

Available at Oncology nursing forum - from David Adams Library Journals Collection Local Print Collection [location] : David Adams Library.

Abstract:PURPOSE/OBJECTIVES: To assess whether a case-based cancer pain education module would lead to acquisition and retention of knowledge and attitudes at the graduate nursing student level.DESIGN: Quasi-experimental pretest, post-test, and follow-up.SETTING: Three nursing schools in the New England area.SAMPLE: 92 graduate nursing students.METHODS: An oncology nurse specialist delivered seven two- to four-hour seminars integrated in existing pharmacology, primary care, or adult health courses. Participants' cancer pain knowledge was assessed at four time points with a paper-and-pencil test: before the seminar, immediately after, and approximately 6 and 24 months after the seminar.MAIN RESEARCH VARIABLE: Cancer pain knowledge.FINDINGS: The intervention was effective in improving students' knowledge of cancer pain management and assessment ($p = 0.0001$), and the effect was retained at 6 and 24 months ($p = 0.0001$ and $p = 0.0024$, respectively).CONCLUSIONS: Policymakers, clinicians, and professional organizations have recommended providing cancer pain education during professional training to overcome the continuing problem of the undertreatment of cancer pain. The education module used was effective in changing students' knowledge of cancer pain management, and the results suggest that this knowledge is lasting.IMPLICATIONS FOR NURSING: Early cancer pain education for nurses may play an important role in improving pain control for patients with cancer. Although this study did not evaluate the application of cancer pain knowledge to clinical practice, the results support the notion that advanced practice nurses can improve their cancer pain management knowledge and attitudes while in training. One implication is that this shift in attitudes and knowledge will translate to effective management of pain in varied healthcare settings.

Institutions:

(Wilkes G) Boston Medical Center, Boston, MA, USA.
Database: PubMed

242. Are nurses prepared to manage cancer pain? A national survey of nurses' knowledge about pain control in Taiwan.

Author(s): Lai YH; Chen ML; Tsai LY; Lo LH; Wei LL; Hong MY; Hsiu LN; Hsiao-Sheen ST; Chen SC; Kao CC; Huang TW; Chang SC; Chen L; Guo SL

Source: Journal of pain and symptom management; Nov 2003; vol. 26 (no. 5); p. 1016-1025

Publication Date: Nov 2003

Publication Type(s): Journal Article; Research Support, Non-U.S. Gov't

DOI: http://dx.doi.org/10.1016/s0885-3924(03)00330-0

ISSN: 0885-3924

Place of Publication: United States

PubMedID: 14585553

Accession Number: 14585553

Available at Journal of pain and symptom management - from David Adams Library Journals Collection Local Print Collection [location] : David Adams Library.

Available at Journal of pain and symptom management - from Unpaywall

Abstract: Nurses play a crucial role in cancer pain control, but little is known about how well-prepared nurses are to manage cancer pain in Taiwan. The purpose of this study was to examine the level of knowledge about pain management among Taiwanese nurses with different background characteristics and to determine the predictor(s) of nurses' pain management knowledge. Nurse subjects were recruited by a cross-sectional nationwide survey with stratified sampling from nine hospitals distributed in the four major geographic regions of Taiwan. The Nurses' Knowledge and Attitudes Survey-Taiwanese version (NKAS-T) and a background information form were used to collect the data. Of 1900 surveys distributed, 1797 valid questionnaires (94.5%) were analyzed. The average correct response rate was 50.5%, with rates ranging from 7-86% for each survey question. Results from stepwise regression showed that nurses with higher mean correct answer scores had BS or higher degrees, had received pain education at professional conferences, had more prior hours of pain education, had longer clinical care experiences, and always worked with cancer patients. Nurses who worked in intensive care units, however, had significantly lower mean correct scores. The results strongly suggest an urgent need to strengthen pain education in Taiwan. The results also provide the direction for developing pain education.

Institutions:
(Lai YH) College of Nursing, Taipei Medical University, Taipei, Taiwan.
Database: PubMed

243. My love is hurting: the meaning spouses attribute to their loved ones' pain during palliative care.

Author(s): Mehta A; Ezer H

Source: Journal of palliative care; 2003; vol. 19 (no. 2); p. 87-94
Publication Date: 2003
Publication Type(s): Journal Article
ISSN: 0825-8597
Place of Publication: United States
PubMedID: 12955924
Accession Number: 12955924

Available at Journal of palliative care - from ProQuest (MEDLINE with Full Text) - NHS Version

Available at Journal of palliative care - from ProQuest (Health Research Premium) - NHS Version

Available at Journal of palliative care - from David Adams Library Journals Collection Local Print Collection [location] : David Adams Library.

Abstract: The purpose of this qualitative study was to develop our knowledge of the pain experiences of family members by addressing the meaning of cancer pain to the spouse of a patient receiving palliative care. In particular, this study explored factors associated with the meanings the spouses ascribe to the experience of pain during palliative care and whether the meanings the patients attributed to pain were similar to the meanings held by the spouse. Two different states emerged, the "in-pain state" and the "out of pain state". The spouses described feelings of helplessness, fear, and unfairness when witnessing their loved one in pain. Once the pain had been controlled, spouses described feelings of peace and relaxation, and felt this meant that the couple could return to their old routines because their spouse was still alive. It was discovered that the meanings placed on the cancer pain differed for the spouse and the patient, with the spouse focusing on future consequences. Implications and suggestions for nursing practice and future research are proposed.

Institutions:
(Mehta A) Sir Mortimer B. Davis-Jewish General Hospital, School of Nursing, McGill University, Montreal, Quebec, Canada.
Database: PubMed

244. Knowledge and attitudes about cancer pain management: a comparison of oncology and nononcology nurses.

Author(s): Rushton P; Eggett D; Sutherland CW

Source: Oncology nursing forum; 2003; vol. 30 (no. 5); p. 849-855
Publication Date: 2003
Publication Type(s): Comparative Study; Journal Article; Research Support, Non-U.S. Gov't
DOI: http://dx.doi.org/10.1188/03.ONF.849-855
ISSN: 1538-0688
Place of Publication: United States
PubMedID: 12949598

Accession Number: 12949598

Available at Oncology nursing forum - from Ovid (Journals @ Ovid)

Available at Oncology nursing forum - from EBSCO (CINAHL Complete)

Available at Oncology nursing forum - from David Adams Library Journals Collection Local Print Collection [location] : David Adams Library.

Abstract:PURPOSE/OBJECTIVES: To obtain information about the knowledge and attitudes of Utah nurses concerning cancer pain management.DESIGN: Descriptive study.SETTING: Nurses in Utah.SAMPLE: 44 oncology nurses and 303 nononcology nurses completed the study.METHODS: Ferrell's Nurses' Knowledge and Attitudes Survey Regarding Pain was given to oncology and nononcology nurses to compare knowledge and attitudes about treating cancer pain.MAIN RESEARCH VARIABLES: Knowledge and attitudes regarding cancer pain.FINDINGS: Attitudes of oncology nurses were more in line with recommended practices (principles) of cancer pain management than those of nononcology nurses. Oncology nurses had a better understanding of recommended practices (principles) of cancer pain management than nononcology nurses but still struggled with understanding the pharmacology of medications used to manage cancer pain.CONCLUSIONS: Nurses do not use evidence-based practice in pain management consistently. Continuing education regarding cancer pain management remains important for oncology and nononcology nurses.IMPLICATIONS FOR NURSING: Adoption of evidence-based practice requires ongoing education of nurses and support from nursing colleagues, nursing administration, and associated healthcare providers. Data from this study can be used to design a curriculum involving content about cancer pain management. All members of the healthcare team should be supported in practicing the correct principles of cancer pain management in actual practice.

Institutions:
(Rushton P) College of Nursing, Brigham Young University, Salt Lake City, UT, USA. patricia_rushton@byu.edu

Database: PubMed

245. National Institutes of Health State-of-the-Science Conference Statement: Symptom Management in Cancer: Pain, Depression, and Fatigue, July 15-17, 2002.

Author(s): Patrick DL; Ferketich SL; Frame PS; Harris JJ; Hendricks CB; Levin B; Link MP; Lustig C; McLaughlin J; Ried LD; Turrisi AT; Unützer J; Vernon SW; National Institutes of Health State-of-the-Science Panel

Source: Journal of the National Cancer Institute; Aug 2003; vol. 95 (no. 15); p. 1110-1117

Publication Date: Aug 2003

Publication Type(s): Consensus Development Conference; Consensus Development Conference, NIH; Journal Article; Research Support, U.S. Gov't, P.H.S.; Review

DOI: http://dx.doi.org/10.1093/jnci/djg014

ISSN: 1460-2105

Place of Publication: United States

PubMedID: 12902440

Accession Number: 12902440

Available at Journal of the National Cancer Institute - from HighWire - Free Full Text

Available at Journal of the National Cancer Institute - from Unpaywall

Abstract:BACKGROUND: Despite advances in early detection and effective treatment, cancer remains one of the most feared diseases. Among the most common side effects of cancer and treatments for cancer are pain, depression, and fatigue. Although research is producing increasingly hopeful insights into the causes and cures for cancer, efforts to manage the side effects of the disease and its treatments have not kept pace. The challenge that faces us is how to increase awareness of the importance of recognizing and actively addressing cancer-related distress. The National Institutes of Health (NIH) convened a State-of-the-Science Conference on Symptom Management in Cancer: Pain, Depression, and Fatigue to examine the current state of knowledge regarding the management of pain, depression, and fatigue in individuals with cancer and to identify directions for future research. Specifically, the conference examined how to identify individuals who are at risk for cancer-related pain, depression, and/or fatigue; what treatments work best to address these symptoms when they occur; and what is the best way to deliver interventions across the continuum of care. State-of-the-Science Process: A non-advocate, non-Federal, 14-member panel of experts representing the fields of oncology, radiology, psychology, nursing, public health, social work, and epidemiology prepared the statement. In addition, 24 experts in medical oncology, geriatrics, pharmacology, psychology, and neurology presented data to the panel and to the conference audience during the first 1.5 days of the conference. The panel then prepared its statement, addressing the five predetermined questions and drawing on submitted literature, the speakers' presentations, and discussions held at the conference. The statement was presented to the conference audience, followed by a press conference to allow the panel to respond to questions from the media. After its release at the conference, the draft statement was made available on the Internet. The panel's final statement is available at http://consensus.nih.gov.CONCLUSIONS: The panel concluded that the available evidence supports a variety of interventions for treating cancer patients' pain, depression, and fatigue. Clinicians should routinely use brief assessment tools to ask patients about pain, depression, and fatigue and to initiate evidence-based treatments. Assessment should include discussion about common symptoms experienced by cancer patients, and these discussions should continue over the duration of the illness. Impediments to effective symptom management in cancer patients can arise from different sources and interactions among providers, patients and their families, and the health care system. Numerous factors could interfere with adequate symptom management. Among these factors are incomplete effectiveness of some treatments, a lack of sufficient knowledge regarding effective treatment strategies, patient reluctance to report symptoms to caregivers, a belief that such symptoms are simply a part of the cancer experience that must be tolerated, and inadequate coverage and reimbursement for some treatments. Additional research is needed on the definition, occurrence, the treatment of pain, depression, and fatigue, alone and in combination, in adequately funded prospective studies. The panel also concluded that the state of the science in cancer symptom management should be reassessed periodically.

Institutions:

(Patrick DL) Department of Health Services, University of Washington, Seattle, WA, USA.; (Ferketich SL) National Institutes of Health State-of-the-Science Panel
(Patrick DL) Department of Health Services, University of Washington, Seattle, WA, USA.; (Ferketich SL) National Institutes of Health State-of-the-Science Panel
Database: PubMed

246. Clinicians communicating with patients experiencing cancer pain.

Author(s): Berry DL; Wilkie DJ; Thomas CR; Fortner P

Source: Cancer investigation; Jun 2003; vol. 21 (no. 3); p. 374-381

Publication Date: Jun 2003

Publication Type(s): Journal Article; Research Support, Non-U.S. Gov't; Review

DOI: http://dx.doi.org/10.1081/cnv-120018228

ISSN: 0735-7907

Place of Publication: England

PubMedID: 12901283

Accession Number: 12901283

Available at Cancer investigation - from EBSCO (MEDLINE Complete)

Abstract:PURPOSE: Provider-patient communication deficits are often implicated as barriers to adequate cancer pain relief. The purpose of this study was to describe verbal communication behaviors and interactions between providers and patients reporting cancer pain.METHODS: As part of a multisite clinical trial, we enrolled 17 oncology physician specialists and 84 patient participants who had reported cancer pain or treatment-related pain in the previous week associated with prostate or head and neck cancer. The study baseline clinic visits (N = 84) were audiotaped, transcribed, and entered into non-numerical unstructured data indexing searching and theorizing (NUD.IST) for content analysis. Each text unit in each transcript was coded as to conversation context: pain, additional symptom/side effects, tumor treatment, and/or personal remarks. Clinician questions were coded as either open-ended or closed-ended, clinician interruptions and subject changes were counted, and a measure of verbal dominance was calculated.RESULTS: The clinicians spent over half the conversations doing the talking. We calculated a close-ended to open-ended question ratio of 5.8:1. In 55% of the visits, the patient with cancer was interrupted by the clinician when the patient attempted to provide information or ask a question. Symptoms/side effects were addressed in practically all visits, whereas sensory pain was addressed in 90% of the visits.CONCLUSIONS: The clinicians were attentive to daily problems relevant to treatment side effects; however, the results also indicate a pattern of communication during the clinic visit that is typically clinician oriented. The nature of such communication may prevent the patient from sharing significant facts and experiences relevant to cancer pain and thus compromise the quality of pain management.

Institutions:

(Berry DL) Biobehavioral Nursing and Health Systems, University of Washington, Box 357266, Seattle, WA 98195-7266, USA. donnalb@u.washington.edu

Database: PubMed

247. Barriers to cancer pain management: home-health and hospice nurses and patients.

Author(s): Randall-David E; Wright J; Porterfield DS; Lesser G

Source: Supportive care in cancer : official journal of the Multinational Association of Supportive Care in Cancer; Oct 2003; vol. 11 (no. 10); p. 660-665

Publication Date: Oct 2003

Publication Type(s): Journal Article; Research Support, Non-U.S. Gov't

DOI: http://dx.doi.org/10.1007/s00520-003-0497-x

ISSN: 0941-4355

Place of Publication: Germany

PubMedID: 12898368

Accession Number: 12898368

Available at Supportive care in cancer : official journal of the Multinational Association of Supportive Care in Cancer - from SpringerLink

Available at Supportive care in cancer : official journal of the Multinational Association of Supportive Care in Cancer - from ProQuest (MEDLINE with Full Text) - NHS Version

Available at Supportive care in cancer : official journal of the Multinational Association of Supportive Care in Cancer - from ProQuest (Health Research Premium) - NHS Version

Available at Supportive care in cancer : official journal of the Multinational Association of Supportive Care in Cancer - from David Adams Library Journals Collection Local Print Collection [location] : David Adams Library.

Abstract:GOALS: Undertreatment of cancer pain remains a major health-care problem. We utilized focus groups of hospice and home-health nurses and patients to elucidate factors contributing to inadequate pain management and to generate solutions for closing the gap between the current reality and optimal pain management.PATIENTS AND METHODS: Focus groups were conducted among hospice and home-health-care nurses (two groups; n=22) and patients (six groups; n=54) using a standardized question guide. Audiotapes were transcribed and analyzed using NUD*IST software. Themes discovered among patients and nurses were analyzed for similarities and differences.MAIN RESULTS: Of 22 participants in the two home-health and hospice nurses focus groups, all were white women, the average age was 43 (range 29-64) years, and the average number of years in nursing was 21 (range 8-47) years. Of 54 participants in the six cancer patient focus groups, 80% were women, the average age was 54 (range 25-76) years, and 76% were white. Fifty-four percent of patients reported a history of pain associated with their cancer, and almost 30% had pain that they rated as 8 or higher on the pain scale. Barriers to adequate pain management fell into four categories: fears; attitudes, beliefs, and values; patient and provider behaviors; and structural barriers. Patients and nurses reported similar barriers to pain management; however, patients identified more barriers related to provider behavior and structure of the health care system.CONCLUSIONS: This study identified several barriers to cancer pain control not previously identified in the literature. Strategies to improve cancer pain control are suggested.

Institutions:

(Randall-David E) Cancer Control Branch, North Carolina Department of Health and Human Services, Mail Center 1915, Raleigh, NC 27699,USA.
Database: PubMed

248. Physician variability in the management of acute postoperative and cancer pain: a quantitative analysis of the Michigan experience.

Author(s): Green CR; Wheeler JR

Source: Pain medicine (Malden, Mass.); Mar 2003; vol. 4 (no. 1); p. 8-20

Publication Date: Mar 2003

Publication Type(s): Journal Article; Research Support, Non-U.S. Gov't

DOI: http://dx.doi.org/10.1046/j.1526-4637.2003.03006.x

ISSN: 1526-2375

Place of Publication: England

PubMedID: 12873274

Accession Number: 12873274

Available at Pain medicine (Malden, Mass.) - from EBSCO (MEDLINE Complete)

Available at Pain medicine (Malden, Mass.) - from Unpaywall

Abstract:BACKGROUND: Little is known about physician attitudes, goals, or satisfaction regarding acute postoperative and cancer pain management.OBJECTIVES: To provide quantitative data regarding the status of acute postoperative and cancer pain management by Michigan physicians. To measure physician confidence, preference, and satisfaction as well as identify their pain care goals for acute postoperative and cancer pain management. To evaluate variability in acute postoperative and cancer pain decision making based upon physician demographic characteristics, knowledge, and attitudes.RESEARCH DESIGN: A cross-sectional survey, which included two cancer and three acute postoperative pain vignettes.SUBJECTS: A randomly-selected sample of three hundred sixty-eight licensed Michigan physicians who provide clinical care for acute postoperative and cancer pain patients.RESULTS: The majority of respondents (>50%) reported providing acute postoperative pain care frequently, while a minority (75%) reported goals of at least adequate pain relief without distress for both acute postoperative and cancer pain. Physicians more frequently chose the optimal pain management response for men following prostatectomy (56.2%) than for women following myomectomy (42%). They also chose the optimal response for metastatic prostate cancer more frequently (16.3%) than for metastatic breast cancer pain management (10.7%).CONCLUSION: These data highlight physician variability in acute postoperative and cancer pain management decision making. Further study of the physician variable is necessary to improve the management of acute postoperative and cancer pain.

Institutions:

(Green CR) Department of Anesthesiology, University of Michigan Health System, Ann Arbor 48109, USA. carmeng@umich.edu
Database: PubMed

249. Developing a computerized data collection and decision support system for cancer pain management.

Author(s): Huang HY; Wilkie DJ; Zong SP; Berry D; Hairabedian D; Judge MK; Farber S; Chabal C

Source: Computers, informatics, nursing : CIN; 2003; vol. 21 (no. 4); p. 206-217

Publication Date: 2003

Publication Type(s): Journal Article; Research Support, Non-U.S. Gov't; Research Support, U.S. Gov't, P.H.S.

DOI: http://dx.doi.org/10.1097/00024665-200307000-00011

ISSN: 1538-2931

Place of Publication: United States

PubMedID: 12869874

Accession Number: 12869874

Available at Computers, informatics, nursing : CIN - from Ovid (Journals @ Ovid) - London Health Libraries

Abstract: Contemporary nursing practice needs reengineering to deliver its service effectively and efficiently. Using computer technology to support clinicians' decision making may be a parsimonious way to provide high-quality, patient-centered, efficient care. The process of developing the PAINReportIt and PAINConsultN system is described, and the results of two pilot studies in which the system was tested are summarized. The feasibility of using the system to assess pain and provide decision support for clinicians is demonstrated. The findings show PAINReportIt to be promising as an effective, efficient way for patients to report their pain. Whether PAINConsultN is an effective answer to cancer pain management barriers warrants further evaluation with larger samples. The advantages of using the system, as compared with use of the traditional pain management process, are discussed.

Institutions:
(Huang HY) Cancer Pain and Symptom Management Research Group, Department of Biobehavioral Nursing and Health Systems, School of Medicine, Box 357266, University of Washington, Seattle, WA 98195-7266, USA.
hyhuang@u.washington.edu
Database: PubMed

250. [A survey of physicians' knowledge about pain therapy with strong opioid analgesics].

Author(s): Rothstein D; Strumpf M; Dertwinkel R; Donner B; Zenz M

Source: Schmerz (Berlin, Germany); Apr 1998; vol. 12 (no. 2); p. 125-129

Publication Date: Apr 1998

Publication Type(s): English Abstract; Journal Article

DOI: http://dx.doi.org/10.1007/s004829800051

ISSN: 0932-433X

Place of Publication: Germany

PubMedID: 12799980

Accession Number: 12799980

Available at Schmerz (Berlin, Germany) - from EBSCO (MEDLINE Complete)

Abstract:INTRODUCTION: The WHO analgesic ladder, including the use of strong opioid analgesics for the treatment of cancer pain, is widely accepted. However, the use of opioids for the treatment of non-cancer pain is still controversial. This study investigates doctors' medical knowledge about basic aspects of pain management. Additionally, we determined whether the deficiencies in the treatment of patients suffering from pain are based on the rigorous national narcotic control system in Germany.METHODS: We investigated the juridical and technical knowledge of physicians specializing in pain therapy by a questionnaire. During a postgraduate course the knowledge about pain therapy according to the WHO analgesic ladder and the beliefs concerning the narcotic regulations in Germany were evaluated. The survey participants were asked to rate their attitudes on a 10-point analogue scale (1=disagreement, 10=full agreement). The participants were also asked to indicate occupational criteria such as specialty, clinical practice area, and postgraduate years of practice. Descriptive statistics for the mean values were used.RESULTS: One hundred and forty-three questionnaires were completed. The majority of participants worked at departments of anaesthesiology. Some 51.1% of the participants had no specific multiple-copy prescriptions for opioid analgesics. Only 72% of the physicians knew from which governmental institution they could order multiple-copy prescriptions. In general, more doctors would prescribe opioids by the use of normal forms. The controlled substance laws were seen as an impediment by the majority of participants, without relevant differences as to their years of practice. The regulations were regarded as ineffective protection against illegal use of opioids. Treatment of pain with strong opioid analgesics was seen as beneficial for the patients. The use of strong opioids for long-term treatment was recommended, and psychological addiction was regarded as non-existent.CONCLUSION: Therapy with strong opioids is accepted practice, but significant deficits of legal and technical knowledge uphold the undertreatment of patients suffering from cancer and non-cancer pain. Patients with a legitimate need for pain relief by strong opioids are the unintended victims of tight narcotic regulations and deficits in medical education. An ease of regulatory conditions is mandatory to reduce the reluctance for prescribing opioids. On the other hand intensified continuous medical education is mandatory to reduce the undertreatment of patients with severe pain conditions.

Institutions:
(Rothstein D) Klinik für Anästhesiologie Intensiv- und Schmerztherapie, Berufsgenossenschaftliche Kliniken Bergmannsheil, Bochum.
Database: PubMed

251. Multicenter study of pain and its management in patients with advanced cancer in Korea.

Author(s): Yun YH; Heo DS; Lee IG; Jeong HS; Kim HJ; Kim SY; Kim YH; Ro YJ; Yoon SS; Lee KH; Huh BY

Source: Journal of pain and symptom management; May 2003; vol. 25 (no. 5); p. 430-437

Publication Date: May 2003

Publication Type(s): Journal Article; Multicenter Study; Research Support, Non-U.S. Gov't
DOI: http://dx.doi.org/10.1016/s0885-3924(03)00103-9
ISSN: 0885-3924
Place of Publication: United States
PubMedID: 12727040
Accession Number: 12727040

Available at Journal of pain and symptom management - from David Adams Library Journals Collection Local Print Collection [location] : David Adams Library.

Available at Journal of pain and symptom management - from Unpaywall

Abstract: The aim of this study was to evaluate the prevalence, severity, and management of pain in Korean patients with advanced cancer, and to identify the predictors of inadequate management of cancer pain in Korea. From 8 university hospitals, 655 patients with advanced cancer were surveyed. Information concerning analgesics prescribed was acquired from the medical records by the investigator. Physicians, nurses and caregivers were asked to estimate patients' pain. The Korean Brief Pain Inventory and the Barrier Questionnaire were completed by the patients. The Pain Management Index was estimated. Among all patients, 70.8% (464 of 655) reported pain. Among those who had pain, 63.6% (295 of 464) reported pain rated 5 or higher on a 0-10 scale. Thirty-nine percent of the patients had not received any analgesics and 53.2% were not receiving optimal pain management. Although there was a correlation between patients' pain ratings and those of doctors, nurses, and caregivers, there was no significant correlation between patients' ratings and health care providers' ratings at pain levels above moderate intensity. Cancer pain was more poorly managed in advanced cancer than terminal cancer patients (OR:3.20, 95%C.I, 1.83-5.60), in patients with better performance(OR:3.17, 95%C.I, 1.64-6.11), and in those patients whose pain was underestimated by the doctor (OR:2.58, 95%C.I. 1.42-4.69). Despite the high prevalence and severity of pain in cancer patients, the assessment and management of cancer pain were found to be inadequate in Korea.

Institutions:
(Yun YH) Quality of Cancer Care Branch, Research Institute, National Cancer Center, Seoul, South Korea
Database: PubMed

252. The Zero Acceptance of Pain (ZAP) Quality Improvement Project: evaluation of pain severity, pain interference, global quality of life, and pain-related costs.

Author(s): Fortner BV; Okon TA; Ashley J; Kepler G; Chavez J; Tauer K; Clements-Thompson M; Schwartzberg L; Demarco G; Houts AC

Source: Journal of pain and symptom management; Apr 2003; vol. 25 (no. 4); p. 334-343

Publication Date: Apr 2003

Publication Type(s): Evaluation Study; Journal Article; Research Support, Non-U.S. Gov't

DOI: http://dx.doi.org/10.1016/s0885-3924(02)00679-6
ISSN: 0885-3924
Place of Publication: United States
PubMedID: 12691685
Accession Number: 12691685

Available at Journal of pain and symptom management - from David Adams Library Journals Collection Local Print Collection [location] : David Adams Library.

Available at Journal of pain and symptom management - from Unpaywall

Abstract: The Zero Acceptance of Pain (ZAP) Quality Improvement Project was a multi-site effort to improve the lives of outpatients with cancer pain by enhancing the clinical practice of pain assessment and management. Independent samples of patients completed self-report measures of severity of pain, pain interference, global quality of life, pain treatment satisfaction, general medical treatment satisfaction, pain attitudes, and pain-related medical costs before and after the implementation of ZAP. Results suggested that ZAP decreased the severity of recent pain, decreased interference of pain on daily functioning, and improved satisfaction with pain treatment and attitudes about addiction to opioid medication. Direct medical costs consisting of pain-related hospitalizations, emergency department visits, and physician office visits were greatly reduced. In summary, the findings of this study support the idea that clinic-based efforts to improve the practice of pain management are effective in improving the lives of cancer patients who are experiencing pain.

Institutions:
(Fortner BV) Psychology and Cancer Symptom Research, West Clinic, 100 N. Humphreys Boulevard, Memphis, TN 38117, USA.
Database: PubMed

253. Decision support computer program for cancer pain management.

Author(s): Im EO; Chee W

Source: Computers, informatics, nursing : CIN; 2003; vol. 21 (no. 1); p. 12-21
Publication Date: 2003
Publication Type(s): Journal Article; Research Support, Non-U.S. Gov't
DOI: http://dx.doi.org/10.1097/00024665-200301000-00008
ISSN: 1538-2931
Place of Publication: United States
PubMedID: 12544150
Accession Number: 12544150

Available at Computers, informatics, nursing : CIN - from Ovid (Journals @ Ovid) - London Health Libraries

Abstract: The purpose of the study was to develop an initial version of computer software that could assist nurses' decision making about cancer pain reported by women from diverse cultural groups. This cross-sectional study included two phases: (1) data collection and (2) development of computer software. Data were collected using an Internet survey and e-mail group discussions of 19 faculty members from 10 countries who were self-identified experts in oncology nursing. The data were

analyzed using descriptive statistics and content analysis. The findings indicated ethnic, gender, geographic, and age differences in cancer pain descriptions. Based on the collected data, a decision support computer program for cancer pain management, including (1) a knowledge base generation module, (2) a decision-making module, and (3) a self-adaptation module, was developed. Based on the study findings, suggestions for future research and practice related to cancer pain and expert systems were proposed.

Institutions:
(Im EO) School of Nursing University of Texas at Austin, 78701, USA. nur@mail.nur.utexas.edu

Database: PubMed

254. The PRO-SELF(c): Pain Control Program--an effective approach for cancer pain management.

Author(s): West CM; Dodd MJ; Paul SM; Schumacher K; Tripathy D; Koo P; Miaskowski C

Source: Oncology nursing forum; 2003; vol. 30 (no. 1); p. 65-73

Publication Date: 2003

Publication Type(s): Journal Article; Research Support, Non-U.S. Gov't; Research Support, U.S. Gov't, P.H.S.; Review

DOI: http://dx.doi.org/10.1188/03.ONF.65-73

ISSN: 1538-0688

Place of Publication: United States

PubMedID: 12515985

Accession Number: 12515985

Available at Oncology nursing forum - from Ovid (Journals @ Ovid)

Available at Oncology nursing forum - from EBSCO (CINAHL Complete)

Available at Oncology nursing forum - from David Adams Library Journals Collection Local Print Collection [location] : David Adams Library.

Abstract:PURPOSE/OBJECTIVES: To describe the PRO-SELF(c): Pain Control Program, an educational approach that provides patients and family caregivers with the knowledge, skills, and nursing support needed to improve pain relief.DATA SOURCES: Published research studies, articles, and conference abstracts.DATA SYNTHESIS: Patients with cancer and family caregivers lack knowledge about pain management and side effects. Engaging in self-care behaviors improves patients' health outcomes.CONCLUSIONS: The PRO-SELF: Pain Control Program is an effective approach that can be used to help patients with cancer and their family caregivers obtain the knowledge and skills that are needed to manage pain. Three key strategies for delivering the PRO-SELF program are (a) provision of information using academic detailing, (b) skill building with ongoing nurse coaching, and (c) interactive nursing support.IMPLICATIONS FOR NURSING: Adequate pain relief is vital to decreasing cancer morbidity and improving patients' quality of life. The PRO-SELF: Pain Control Program should be implemented in all settings where cancer care takes place.

Institutions:

(West CM) School of Nursing, University of California, San Francisco, CA, USA.
Database: PubMed

255. Patient-related barriers to pain management: the Barriers Questionnaire II (BQ-II).

Author(s): Gunnarsdottir S; Donovan HS; Serlin RC; Voge C; Ward S

Source: Pain; Oct 2002; vol. 99 (no. 3); p. 385-396

Publication Date: Oct 2002

Publication Type(s): Comparative Study; Journal Article; Research Support, Non-U.S. Gov't; Research Support, U.S. Gov't, P.H.S.

DOI: http://dx.doi.org/10.1016/S0304-3959(02)00243-9

ISSN: 0304-3959

Place of Publication: United States

PubMedID: 12406513

Accession Number: 12406513

Abstract: Patients' beliefs can act as barriers to optimal management of cancer pain. The Barriers Questionnaire (BQ) is a tool used to evaluate such barriers. Here, the BQ has been revised to reflect changes in pain management practices, resulting in the Barriers Questionnaire-II (BQ-II), a 27-item, self report instrument. This paper presents the results from two studies where the psychometric properties of the BQ-II were evaluated. In the first study, the responses of 27 nurses trained in pain management were compared to responses of a convenience sample of 12 patients with cancer. The results indicated that patients with cancer had higher mean scores on the BQ-II than did nurses trained in pain management. In the second study, a convenience sample of 172 patients with cancer responded to the BQ-II and a set of pain and quality of life (QOL) measures. A factor analysis supported four factors. Factor one, physiological effects, consists of 12 items addressing the beliefs that side effects of analgesics are inevitable and unmanageable, concerns about tolerance, and concerns about not being able to monitor changes in one's body when taking strong pain medications. Factor two, Fatalism, consists of three items addressing fatalistic beliefs about cancer pain and its management. Factor three, Communication, consists of six items addressing the concern that reports of pain distract the physician from treating the underlying disease, and the belief that 'good' patients do not complain of pain. The fourth and final factor, harmful effects, consists of six items addressing fear of becoming addicted to pain medication and the belief that pain medications harm the immune system. The BQ-II total had an internal consistency of 0.89, and alpha for the subscales ranged from 0.75 to 0.85. Mean (SD) scores on the total scale was 1.52 (0.73). BQ-II scores were related to measures of pain intensity and duration, mood, and QOL. Patients who used adequate analgesics for their levels of pain had lower scores on the BQ-II than did patients who used inadequate analgesics. The BQ-II is a reliable and valid measure of patient-related barriers to cancer pain management.

Institutions:
(Gunnarsdottir S) School of Nursing, University of Wisconsin-Madison, K6/333, 600 Highland Avenue, Madison, WI 53792-2455, USA Department of Educational

Psychology, University of Wisconsin-Madison, Madison, WI, USA University of Wisconsin Madison Hospital and Clinics, Madison, WI, USA.
Database: PubMed

256. [Indicators of structural quality in palliative care for cancer pain patients in Lower-Saxony].

Author(s): Ensink FB; Bautz MT; Voss MC; Görlitz A; Hanekop GG

Source: Schmerz (Berlin, Germany); Aug 2002; vol. 16 (no. 4); p. 255-262

Publication Date: Aug 2002

Publication Type(s): English Abstract; Journal Article

DOI: http://dx.doi.org/10.1007/s00482-002-0163-0

ISSN: 0932-433X

Place of Publication: Germany

PubMedID: 12192434

Accession Number: 12192434

Available at Schmerz (Berlin, Germany) - from EBSCO (MEDLINE Complete)

Abstract:INTRODUCTION: Palliative care in Germany fails to reach established standards. To improve this situation the Chamber of Physicians of Lower-Saxony initiated SUPPORT in 1995. Prior to interventions structural quality of care was evaluated, specifically the rate of availability of opioid-prescription-forms and the ability to treat chronic pain (defined as a construct of knowledge, attitudes and skills) were examined.METHODS: The survey was carried out using a standardized questionnaire mailed to a representative stratified sample of 1200 physicians.RESULTS: Out of 865 answering physicians (response rate 72.1%) only 36.9% had their own opioid-prescription-forms. Differentiations regarding to specialty, working place (clinic vs. private practice) and treatment of cancer pain patients during the last three months shows a better result for GPs (84.6%), internists (48.6%), gynecologists (51%) and pain specialists (66.7%). Only 33.1% of respondents claimed knowledge of the WHO-3-step-analgesic-ladder. Again the aforementioned differentiations yield somewhat better results for GPs (49.2%), internists (51.5%), gynecologists (34.7%) and pain specialists (55.6%), however only two thirds of these physicians were able to identify the correct number of steps of the WHO-algorithm.CONCLUSIONS: These results verify an insufficient structural quality in palliative care in Lower-Saxony. In the authors' opinion effective improvements can only be achieved by implementing a parallel strategy:improvement of basic knowledge in pain management with sufficient transfer of this knowledge into practice as well as raising the rate of availability of opioid-prescription-forms,and, on the other hand, establishing local palliative-care-teams with nursing and medical expertise with 24/7 on-demand availability to optimize palliative care.

Institutions:
(Ensink FB) Arbeitsgruppe SUPPORT der Arztekammer Niedersachsen am Zentrum Anaesthesiologie, Rettungs- und Intensivmedizin, Georg-August-Universität Göttingen, Germany. fensink@gwdg.de
Database: PubMed

257. Chronic non-cancer pain in older people: current evidence for prescribing.

Author(s): Cowan DT

Source: British journal of community nursing; Aug 2002; vol. 7 (no. 8); p. 420-425

Publication Date: Aug 2002

Publication Type(s): Journal Article; Review

DOI: http://dx.doi.org/10.12968/bjcn.2002.7.8.10648

ISSN: 1462-4753

Place of Publication: England

PubMedID: 12192346

Accession Number: 12192346

Available at British journal of community nursing - from MAG Online Library

Available at British journal of community nursing - from EBSCO (CINAHL Complete)

Abstract: Chronic pain is a serious problem for many older people, yet its management in this population is often less than satisfactory. The high incidence of toxicity and side effects with some medications and irrational fears of opioid dependence are significant barriers to effective pain management. In this article, the research evidence relating to these issues is examined, and their impact on nurse prescribing considered. More studies are needed to assess the veracity of opposing arguments, and to support effective assessment and prescribing by nurses.

Institutions:
(Cowan DT) Primary Care Research Group, Florence Nightingale School of Nursing and Midwifery, King's College London, London, UK.
Database: PubMed

258. Ethical issues in pain management.

Author(s): Swenson CJ

Source: Seminars in oncology nursing; May 2002; vol. 18 (no. 2); p. 135-142

Publication Date: May 2002

Publication Type(s): Journal Article; Review

DOI: http://dx.doi.org/10.1053/sonu.2002.32511

ISSN: 0749-2081

Place of Publication: United States

PubMedID: 12051165

Accession Number: 12051165

Available at Seminars in oncology nursing - from David Adams Library Journals Collection Local Print Collection [location] : David Adams Library.

Abstract:OBJECTIVE: To review the ethical principles of autonomy, nonmaleficence, beneficence, and justice to assist in understanding nursing's ethical obligation to

patients and families in the practice of pain management.DATA SOURCES: Position papers, government guidelines, and nursing and legal literature.CONCLUSION: Cancer pain management has been an issue in nursing and medical practice for more than a quarter of a century. Today we have numerous organisations that focus exclusively on the issue of pain and yet the oncology patient continues to have inadequate pain control.IMPLICATIONS FOR NURSING PRACTICE: With the acknowledgement that we have the technical skills and the physiological knowledge to reduce pain, yet it is not being done, health care professionals have begun to explore the ethics behind pain.

Institutions:
(Swenson CJ) Regional Cancer Center, SwedishAmerican Hospital, 1400 Charles St, Rockford, IL 61104-2298, USA.
Database: PubMed

259. Pediatric nurses' knowledge and attitudes survey regarding pain.

Author(s): Manworren RC

Source: Pediatric nursing; 2000; vol. 26 (no. 6); p. 610-614

Publication Date: 2000

Publication Type(s): Journal Article

ISSN: 0097-9805

Place of Publication: United States

PubMedID: 12026363

Accession Number: 12026363

Available at Pediatric nursing - from EBSCO (CINAHL Complete)

Available at Pediatric nursing - from ProQuest (MEDLINE with Full Text) - NHS Version

Abstract:PURPOSE: The purpose of this study is to determine pediatric nurses' current attitudes and knowledge regarding pain.METHOD: The Pediatric Nurses' Knowledge and Attitudes Regarding Pain Survey was completed by a convenience sample of 274 nurses at a large children's medical center.RESULTS: Sixty-six percent of the questions were answered correctly. Nurses with their master's degree scored significantly higher (75%). Hematology/oncology nurses (76%), nurses from the intensive care unit (71%), and emergency room nurses (70%) scored significantly higher than nurses from other patient care units.CONCLUSIONS: Pain management knowledge deficiencies were identified, including assessment; pharmacologic management with opioids, nonopioids, and adjuvant medications; risks of addiction; risks of respiratory depression; nonpharmacologic pain interventions; and the treatment of procedural pain, surgical pain, and cancer pain.

Institutions:
(Manworren RC) Children's Medical Center of Dallas, Dallas, TX, USA.
Database: PubMed

260. Cancer pain management among underserved minority outpatients: perceived needs and barriers to optimal control.

Author(s): Anderson KO; Richman SP; Hurley J; Palos G; Valero V; Mendoza TR; Gning I; Cleeland CS

Source: Cancer; Apr 2002; vol. 94 (no. 8); p. 2295-2304

Publication Date: Apr 2002

Publication Type(s): Journal Article; Research Support, U.S. Gov't, Non-P.H.S.; Research Support, U.S. Gov't, P.H.S.

DOI: http://dx.doi.org/10.1002/cncr.10414

ISSN: 0008-543X

Place of Publication: United States

PubMedID: 12001130

Accession Number: 12001130

Available at Cancer - from Wiley Online Library

Available at Cancer - from Unpaywall

Abstract:BACKGROUND: Minority patients with cancer are at risk for undertreatment of cancer-related pain. Most studies of patient-related barriers to pain control have surveyed primarily non-Hispanic Caucasian patients. The purpose of the current study was to explore barriers to optimal pain management among African-American and Hispanic patients with cancer through the use of structured patient interviews. Structured interviews allowed the authors to probe for previously unidentified barriers to pain management in these populations.METHODS: Thirty-one socioeconomically disadvantaged minority patients with cancer (14 African-American patients and 17 Hispanic patients) who had cancer-related pain completed structured interviews that assessed three main content areas: information and communication regarding cancer pain, treatment of cancer pain, and the meaning of cancer pain.RESULTS: The African-American and Hispanic patients reported severe pain and many concerns about pain management. The majority of patients in both ethnic groups expressed a belief in stoicism and concerns about possible addiction to opioid medications and the development of tolerance. The patients described their physicians as the most frequent and trusted source of information about cancer pain. However, patients also reported difficulties with communication and a reluctance to complain of pain.CONCLUSIONS: The reported barriers to pain management indicate that socioeconomically disadvantaged African-American and Hispanic patients can benefit from educational interventions on cancer pain that dispel myths about opioids and teach patients to communicate assertively about their pain with their physicians and nurses.

Institutions:
(Anderson KO) Department of Symptom Research, The University of Texas M. D. Anderson Cancer Center, Houston, Texas 77030, USA.
koanderso@mdanderson.org
Database: PubMed

261. Why study pain? A qualitative analysis of medical and nursing faculty and students' knowledge of and attitudes to cancer pain management.

Author(s): Lasch K; Greenhill A; Wilkes G; Carr D; Lee M; Blanchard R

Source: Journal of palliative medicine; Feb 2002; vol. 5 (no. 1); p. 57-71

Publication Date: Feb 2002
Publication Type(s): Journal Article; Research Support, U.S. Gov't, P.H.S.
DOI: http://dx.doi.org/10.1089/10966210252785024
ISSN: 1096-6218
Place of Publication: United States
PubMedID: 11839228
Accession Number: 11839228

Abstract: Although effective means for pain management have long been available, cancer pain remains widely undertreated. Surveys of medical personnel have revealed knowledge deficits and attitudinal barriers to pain management, but have not determined why such attitudes persist and how they may be addressed in medical and nursing curricula. This paper presents findings from a qualitative study of the beliefs and attitudes toward pain and cancer pain management held by medical and nursing students and faculty who participated in the Cancer Education Module for the Management of Pain (CEMMP) project. Analysis centered on informants' prioritization and knowledge of pain and cancer pain management and on the meanings informants assigned to pain in a clinical context. Themes in prioritization included the importance of learning about pain versus cancer pain and the responsibility of primary care providers versus specialists for pain and cancer pain management. Themes in informants' knowledge of pain included knowledge deficits about medications and adjunct therapies and the presence of pain management in the curriculum, and the role of knowledgeable faculty members and mentors in the dissemination of information about pain management. Themes in the meanings informants' assigned to pain included opioidphobia, and the (inter-)subjectivity of pain. The discussion focuses in particular on tensions within the prioritization, knowledge and meanings of pain that must be resolved before students can be appropriately educated for optimal pain management.

Institutions:
(Lasch K) The Health Institute, Division of Clinical Care Research, New England Medical Center, Boston, Massachusetts 02111, USA. klasch@lifespan.org
Database: PubMed

262. The knowledge and attitudes of surgical staff towards the use of opioids in cancer pain management: can the Hospital Palliative Care Team make a difference?

Author(s): Wells M; Dryden H; Guild P; Levack P; Farrer K; Mowat P

Source: European journal of cancer care; Sep 2001; vol. 10 (no. 3); p. 201-211
Publication Date: Sep 2001
Publication Type(s): Journal Article; Research Support, Non-U.S. Gov't
DOI: http://dx.doi.org/10.1046/j.1365-2354.2001.00259.x
ISSN: 0961-5423
Place of Publication: England
PubMedID: 11829383
Accession Number: 11829383

Available at European journal of cancer care - from Wiley Online Library Medicine and Nursing Collection 2020

Available at European journal of cancer care - from David Adams Library Journals Collection Local Print Collection [location] : David Adams Library.

Abstract: The principles of cancer pain management are well established, but evidence suggests that these are not incorporated into daily practice and patients are still in pain. Deficiencies in knowledge and inappropriate attitudes towards the use of opioids may partially explain why the management of cancer pain is still such a widespread problem. This study assessed the knowledge and attitudes of 135 nursing and medical staff working in a surgical unit, before and after working with a newly established Hospital Palliative Care Team. The baseline survey highlighted the existence of a number of myths and misconceptions in relation to opioid use. Results of the follow-up survey indicated that the knowledge and attitudes of doctors and nurses had improved after working with the team, but that this probably occurred as a result of good working relationships and case discussions rather than through formal teaching. The study provided a useful method of identifying deficiencies in knowledge and attitude among staff, and helped to raise awareness of the problem of cancer pain management. Our challenge now is to work with staff to ensure that positive changes in knowledge and attitudes are translated into the everyday practice of hospital nurses and doctors caring for patients with cancer.

Institutions:
(Wells M) University of Dundee School of Nursing and Midwifery/Tayside University Hospitals NHS Trust, UK. emwells@snm.dundee.ac.uk
Database: PubMed

263. Opioid use in chronic pain management in the Philippines.

Author(s): Javier FO; Magpantay LA; Espinosa EL; Harder SM; Unite MA

Source: European journal of pain (London, England); 2001 ; p. 83-85

Publication Date: 2001

Publication Type(s): Journal Article

DOI: http://dx.doi.org/10.1053/eujp.2001.0286

ISSN: 1090-3801

Place of Publication: England

PubMedID: 11798224

Accession Number: 11798224

Available at European journal of pain (London, England) - from Wiley Online Library Medicine and Nursing Collection 2020

Abstract: The aim of this investigation was to determine current opioid use in the Philippines and the reasons why its use is very low. We surveyed 314 doctors in Metro Manila to determine their specialty, possession of narcotics license, and knowledge of opioid use beyond the terminal stage. We found that the majority of respondents possess a narcotics licence. All of them see pain patients in their practice. They agree that opioids should not be reserved for the terminally ill; 235 have prescribed opioids for non-cancer pain. A small minority believes that use of opioids for non-cancer pain can lead to addiction. Opioids that were most easily

recalled were morphine, meperidine and nalbuphine. The survey contradicts the national data for opioid use. With an INCB (International Narcotics Control Board) allocation of 87 kilograms annually, less than 15 kilograms are consumed every year. Fentanyl has a 7 gram usage versus a 100 gram INCB allocation. We conclude that actual opioid use in the Philippines is minimal. However, the correlation between survey results and actual usage indicates a strong awareness of the usefulness of opioids but hesitancy in opioid prescription.

Institutions:
(Javier FO) Pain Management Center, St. Luke's Medical Center, Metro Manila, Philippines.

Database: PubMed

264. [Factors that enable the patients to live their life till death at home by controlling cancer-pains with continuous subcutaneous injection of opioids-case reports of 3 patients with a terminal cancer].

Author(s): Yoshimura M; Takeu R

Source: Gan to kagaku ryoho. Cancer & chemotherapy; Dec 2001 ; p. 110-113

Publication Date: Dec 2001

Publication Type(s): Case Reports; English Abstract; Journal Article

ISSN: 0385-0684

Place of Publication: Japan

PubMedID: 11787275

Accession Number: 11787275

Abstract: Recently, it is gradually getting easier to change a life-style of the patients with cancer-pains from the conventional hospitalized way to the home healthcare system, because of the progress in technique of reducing pains and symptoms and because of prevalence of visiting nurse system. Home healthcare system or hospice is aimed at the improvement of the quality of life (QOL) of the patients. We had 3 patients who died at home after home healthcare service and whose cancer-pains were well controlled till their death at home by continuous subcutaneous injections of opioids (painkiller) when these patients could not take oral medications any more. Therefore, in order to determine the factors that enable the patients to live the terminal stage of their life at home until death by controlling their cancer-pain with continuous subcutaneous injections of opioid, we examined 3 patients who died of cancer at home under a good pain control. The subjects of the present study were 3 patients, who initially had oral or rectal medication of opioid for their pain control, eventually switched to subcutaneous injection of opioid and then died at home under a good pain control between April 1998 and December 2000. We collected all the information through nursing diaries, regarding painkiller care, and interaction of the patients, their family members and other people, and discussed the factors which enabled to maintain a good pain control in these patients at home by continuous subcutaneous injection of painkiller (opioid). As a result, the following 7 items were notified as the factors common in these 3 patients. 1) The patients themselves understood the diagnosis of their diseases and symptoms and could openly discuss the issues such as "how the patient and his/her family would like to live his/her life from now on" among family members, and also between family and medical

associates. 2). The patients received detailed explanation of continuous subcutaneous injection at the time of admission to the hospital and chose to receive the continuous subcutaneous injection with their own will. 3) The patients had no other painful symptoms except cancer pains or had them well controlled if they had any, and had much stronger desire to live their life at home than above all. The family member agreed with the patients and respected their choice. 4) The family members had enough nursing capability, so they could properly handle medications and medical equipment as well as they could take care of the patients. 5) Both primary care physicians and visiting nurses had enough knowledge of home healthcare service for painkiller, and were able to frequently interact with the patients and their family in order to reduce their pains. 6) Visiting nurses supported the family by 24 hr-system and assisted the family in nursing the patients at home without worry. 7) Pharmacists also participated in the home healthcare system, thus, they could smoothly provide and manage opioids without any trouble.

Institutions:
(Yoshimura M) Visiting Nurse Station Mizuho.
Database: PubMed

265. Understanding opioid tolerance in cancer pain.

Author(s): Cady J

Source: Oncology nursing forum; 2001; vol. 28 (no. 10); p. 1561

Publication Date: 2001

Publication Type(s): Journal Article; Review

ISSN: 0190-535X

Place of Publication: United States

PubMedID: 11759304

Accession Number: 11759304

Available at Oncology nursing forum - from EBSCO (CINAHL Complete)

Available at Oncology nursing forum - from David Adams Library Journals Collection Local Print Collection [location] : David Adams Library.

Abstract:PURPOSE/OBJECTIVES: To review opioid tolerance in chronic cancer pain, define the phenomenon and its scope, review physiologic mechanisms, and discuss clinical strategies to identify and manage this complex issue.DATA SOURCES: Review articles, case studies, original research, and published guidelines.DATA SYNTHESIS: Novel therapies to prevent/reverse tolerance are being investigated with a possible future role for N-methyl-d-aspartate antagonists.CONCLUSIONS: Greater nursing research is needed to identify patient risk factors for tolerance development and clinical measurement of the phenomenon. Understanding cellular mechanisms for tolerance may contribute to better management.IMPLICATIONS FOR NURSING PRACTICE: Nursing knowledge of tolerance is important to provide the basis for accurate patient assessment, education, and pain management.

Institutions:
(Cady J) Department of Anesthesiology, Virginia Mason Medical Center, Seattle, WA, USA. jormain.cady@vmmc.org

Database: PubMed

266. An educational implementation of a cancer pain algorithm for ambulatory care.

Author(s): Du Pen AR; Du Pen S; Hansberry J; Miller-Kraybill B; Millen J; Everly R; Hansen N; Syrjala K

Source: Pain management nursing : official journal of the American Society of Pain Management Nurses; Dec 2000; vol. 1 (no. 4); p. 116-128

Publication Date: Dec 2000

Publication Type(s): Clinical Trial; Journal Article; Multicenter Study; Randomized Controlled Trial; Research Support, U.S. Gov't, P.H.S.

DOI: http://dx.doi.org/10.1053/jpmn.2000.19333

ISSN: 1524-9042

Place of Publication: United States

PubMedID: 11709865

Accession Number: 11709865

Abstract: Algorithms are proposed as a means of operationalizing guidelines or standards for cancer pain management. Professional education is used as the means to translate knowledge into practice. Outcomes measurement is the gold standard for validating improvement. This study used an educational intervention to transfer knowledge on implementing a previously tested algorithm for cancer pain management into community outpatient oncology clinics and, subsequently, measuring patient outcomes. Physicians and nurses from 9 Puget Sound clinics were randomized by institution blocks to either "training" or "no training." Role model physician/nurse teams were the core faculty for a day-long seminar. Written reference materials and documentation tools were provided to the trained physician/nurse teams. A total of 105 patients of trained and untrained providers were accrued and assessed over 4 months. Patients of trained providers had a significant reduction in usual pain over the 4 months of data collection compared with patients of untrained providers ($t = 2.0$; $p = .05$). Improvements were modest in the prescription of opioid analgesics and dramatic in the prescription of co-analgesics for neuropathic pain. There was a clear deterioration in the impact of the training over time. The most significant effect occurred within the first 140 days after the intervention and was followed by a gradual return to baseline practice. In conclusion, algorithmic interventions can be successfully transferred into community practice, but further work must be performed to develop methods for securing retention of knowledge and maintaining improved outcomes.

Institutions:
(Du Pen AR) Department of Pain Research, Swedish Medical Center, First Hill, Seattle, WA, USA. anna.dupen@painconsult.com

Database: PubMed

267. [Improvement of palliative outpatient treatment of terminally ill cancer patients - SUPPORT as example - The ethically preferable alternative to euthanasia].

Author(s): Ensink FB; Bautz MT; Hanekop GG

Source: Anasthesiologie, Intensivmedizin, Notfallmedizin, Schmerztherapie : AINS; Sep 2001; vol. 36 (no. 9); p. 530-537

Publication Date: Sep 2001

Publication Type(s): English Abstract; Journal Article

DOI: http://dx.doi.org/10.1055/s-2001-17256

ISSN: 0939-2661

Place of Publication: Germany

PubMedID: 11686126

Accession Number: 11686126

Abstract:INTRODUCTION: Industrial countries are experiencing substantial increases in cancer prevalence. While advanced cancer therapies resulted in prolonged survival most neoplasms still are incurable. Especially advanced stages of cancer are often accompanied by severe pain and other disabling symptoms. Sufficient pain and symptom control is needed to maintain a decent quality of life for cancer patients. However, expert palliative care for patients suffering from cancer pain is still insufficient. These deficits have encouraged pro-euthanasia pressure groups demanding legitimation of physician-assisted-suicide in Germany. Acting under the guise of promoting patient's autonomy these groups are gaining additional momentum from similar legislation passed in the Netherlands.METHODS: Hospice movement and specialists in palliative medicine reject euthanasia as unethical and instead push for the global development of palliative care services. To address these issues the project SUPPORT was established in the Southern part of Lower-Saxony in 1996 with approval by the local ethics committee and sponsored by the German Ministry of Health. A palliative-care-team (PCT) of nurses and physicians with expert knowledge in palliative medicine supports patients after discharge from hospital by providing state-of-the-art palliative care at home. The PCT is available as a 24/7 standby service and can be called on demand by general practitioners, members of outpatient nursing services as well as by patients and their relatives. By cooperating with the PCT these professional and lay caregivers improve their knowledge and skills regarding pain and symptom control for terminally ill patients.RESULTS: During almost 4 years of practical work more than 50 % of the patients enrolled in the project died at home compared to about 20 % under regular conditions. These data point out quite impressively that due to the PCT-interventions recurrent hospitalisations in a majority of cancer pain patients can be avoided when expert knowledge and help is available at home for patients, their relatives and caregivers whenever needed.CONCLUSION: When sufficiently supported at home by palliative experts the number of patients dying at home is reasonably higher than the rate observed under regular conditions. This would also comply with the wishes of most patients who prefer to die in the privacy of their own home. The project data suggest that the concept of SUPPORT should be capable to improve the current state of palliative medicine in other areas of Germany as well.

Institutions:
(Ensink FB) Arbeitsgruppe SUPPORT der Arztekammer Niedersachsen und des Zentrums Anaesthesiologie, Rettungs- und intensivmedizin. fensink@gwdg.de
Database: PubMed

268. Establishing a cancer pain clinic in a developing country: effect of a collaborative link project with a UK cancer pain center.

Author(s): Williams JE; Chandler A; Ranwala R; DeSilva BS; Amarasinghe I

Source: Journal of pain and symptom management; Oct 2001; vol. 22 (no. 4); p. 872-878

Publication Date: Oct 2001

Publication Type(s): Journal Article

DOI: http://dx.doi.org/10.1016/s0885-3924(01)00342-6

ISSN: 0885-3924

Place of Publication: United States

PubMedID: 11576804

Accession Number: 11576804

Available at Journal of pain and symptom management - from David Adams Library Journals Collection Local Print Collection [location] : David Adams Library.

Available at Journal of pain and symptom management - from Unpaywall

Abstract: This paper describes a project for the establishment of a cancer pain clinic in a developing country. The project was conducted according to guidelines from the World Health Organization and utilized a link with an existing cancer pain clinic in the UK. The principal methods used for establishing the new pain clinic included: an assessment of barriers to effective cancer pain control, teaching programs for nurses and trainee doctors, educational links with a UK cancer pain clinic, and analgesic guidelines and introduction of a pain assessment tool. As a result of these interventions, a new cancer pain clinic was founded. The methods used serve as one possible model for establishing cancer pain treatment facilities in developing countries.

Institutions:
(Williams JE) Pain Management and Anesthesia, Royal Marsden NHS Trust, London, United Kingdom.
Database: PubMed

269. Educational interventions to improve cancer pain control: a systematic review.

Author(s): Allard P; Maunsell E; Labbé J; Dorval M

Source: Journal of palliative medicine; 2001; vol. 4 (no. 2); p. 191-203

Publication Date: 2001

Publication Type(s): Journal Article; Research Support, Non-U.S. Gov't; Review; Systematic Review

DOI: http://dx.doi.org/10.1089/109662101750290227

ISSN: 1096-6218

Place of Publication: United States

PubMedID: 11441627

Accession Number: 11441627

Abstract:CONTEXT: Inadequate pain control is a dismaying reality in cancer patients.OBJECTIVES: To review studies on cancer pain control interventions, and describe their findings with respect to participants' attitudes and knowledge, pain management, and pain levels.DATA SOURCES: Computer searches were made in MEDLINE from January 1962, in PsychLIT from January 1974, and in CINAHL from January 1982 to August 1999, using a search strategy based on a combination of key words.STUDY SELECTION: Computerized listings from these sources contained 383, 26, and 85 articles, respectively. After exclusion of duplicates, abstracts, editorials, letters, and irrelevant articles, we retained for review 33 articles, of which 25 (76%) were interventions targeting health professionals, and 8 (24%) interventions targeting patients and family caregivers.DATA EXTRACTION: Study reports were reviewed using the following structured framework: Intervention Setting, Study Methods, Process Assessment, and Pain Outcome Assessment (Attitudes and Knowledge, Pain Management, and Pain Relief/Quality of Life).DATA SYNTHESIS AND CONCLUSION: Educational interventions can successfully improve cancer pain knowledge and attitudes of health care professionals, but without having much impact on patients' pain levels. The most promising avenue for improving cancer pain control in ambulatory settings may be brief, nursing interventions targeting patients in combination with a daily pain diary. This review suggests that further progress may occur through incorporating a systematic and valid method of documenting daily fluctuation in pain levels, and ensuring that documented uncontrolled pain is followed rapidly by clinical reassessment and dose adjustment.

Institutions:
(Allard P) Epidemiology Research Group, Department of Social and Preventive Medicine, Laval University, Quebec, QC, Canada. pallard@scohs.on.ca
Database: PubMed

270. Attitudes and knowledge about cancer pain in Flanders. The educational effect of workshops regarding pain and symptom control.

Author(s): Bauwens S; Distelmans W; Storme G; Kaufman L

Source: Palliative medicine; May 2001; vol. 15 (no. 3); p. 181-189

Publication Date: May 2001

Publication Type(s): Journal Article

DOI: http://dx.doi.org/10.1191/026921601678576167

ISSN: 0269-2163

Place of Publication: England

PubMedID: 11407189

Accession Number: 11407189

Available at Palliative medicine - from ProQuest (MEDLINE with Full Text) - NHS Version

Available at Palliative medicine - from ProQuest (Health Research Premium) - NHS Version

Abstract: Despite international agreements and recommendations regarding cancer pain therapy, the effectiveness of pain treatment is still a major problem even in Western countries. Part of the problem is that physicians and nurses often lack

knowledge of methods for the assessment and treatment of cancer pain and may have many rigid beliefs and attitudes. This study investigated the misconceptions of physicians and nurses that play a role in the undertreatment of pain in Flanders (Belgium). We approached 197 health care workers who participated in the pain and symptom control education sessions organized by the Federation Palliative Care Flanders, and asked them to complete a questionnaire both before and after the sessions. The impact of the education sessions on their knowledge and beliefs regarding the management of cancer pain was substantial. Methods of reaching the target groups that do not feel the need for further education are discussed.

Institutions:
(Bauwens S) Oncological Centre, Academic Hospital Free University of Brussels, Laarbeeklaan 101, 1090 Brussels, Belgium.
Database: PubMed

271. Is hands-on experience more effective than didactic workshops in postgraduate cancer pain education?

Author(s): Lasch KE; Wilkes G; Lee J; Blanchard R

Source: Journal of cancer education : the official journal of the American Association for Cancer Education; 2000; vol. 15 (no. 4); p. 218-222

Publication Date: 2000

Publication Type(s): Clinical Trial; Journal Article; Randomized Controlled Trial; Research Support, Non-U.S. Gov't; Research Support, U.S. Gov't, P.H.S.

DOI: http://dx.doi.org/10.1080/08858190009528701

ISSN: 0885-8195

Place of Publication: England

PubMedID: 11199239

Accession Number: 11199239

Abstract:BACKGROUND: This study examined the nurse outcomes of a cancer pain education program for nurses of patients from 11 different ethnic groups.METHODS: Four hundred ninety six home, hospital, and hospice nurses participated in a one-day workshop or two half-day workshops on cancer pain assessment and management. Of these, 116 were randomized to participate in a bedside-precepted visit with an oncology nurse specialist with pain specialization and a focus group to discuss attitudinal issues. Eighty-six nurses served as controls. Pre-, post- and one-year follow-up tests were administered.RESULTS: Attitudes, knowledge, and application skills significantly improved for workshop-only and enriched-model nurses relative to controls.CONCLUSION: For postgraduate nurses, daylong cancer pain education workshops were, in the group studied, as effective as hands-on experience in improving cancer pain knowledge and changing attitudes. Both the workshop-only and the enriched-model nurses relative to controls had significantly improved knowledge and changed attitudes towards optimal pain management.

Institutions:
(Lasch KE) Health Institute, New England Medical Center, Department of Medicine, Tufts University School of Medicine, Boston, MA 02111, USA.
Database: PubMed

272. Feasibility of quantitative pain assessment in outpatient oncology practice.

Author(s): Rhodes DJ; Koshy RC; Waterfield WC; Wu AW; Grossman SA

Source: Journal of clinical oncology : official journal of the American Society of Clinical Oncology; Jan 2001; vol. 19 (no. 2); p. 501-508

Publication Date: Jan 2001

Publication Type(s): Journal Article; Research Support, Non-U.S. Gov't

DOI: http://dx.doi.org/10.1200/JCO.2001.19.2.501

ISSN: 0732-183X

Place of Publication: United States

PubMedID: 11208844

Accession Number: 11208844

Abstract:PURPOSE: Although physicians view failure to assess pain systematically as the most important barrier to outpatient cancer pain management, little is known about pain assessment in this setting. We sought to determine whether pain is routinely assessed and whether routine quantitative pain assessment is feasible in a busy outpatient oncology practice.PATIENTS AND METHODS: We conducted a pre- and postintervention chart review of 520 randomly selected medical and radiation oncology patient visits at a community hospital-based private outpatient practice. The intervention consisted of training health assistants (HAs) to measure and document patient pain scores by using a visual analog scale. The main outcome measures included HA documentation of patient pain scores, quantitative and qualitative mention of pain in the physician note, and analgesic treatment before and after the intervention.RESULTS: After the intervention, HA documentation of pain scores increased from 1% to 75.6% ($P < .0001$). Physician documentation increased from 0% to 4.8% for quantitative documentation ($P < .01$), and from 60.0% to 68.3% for qualitative documentation (not significant). Of all the patients, 23.1% reported significant pain. Subgroups with greater pain included patients actively receiving radiation treatments and patients with lung cancer. Of patients with significant pain, 28.2% had no mention of pain in the physician note and 47.9% had no documented analgesic treatment.CONCLUSION: Quantitative pain assessment was virtually absent before our intervention but easily implemented and sustained in a busy outpatient oncology practice. Pain score collection identified a high prevalence of pain, patient subgroups at risk for pain, and a significant proportion of patients with pain that was neither evaluated nor treated by their oncologists.

Institutions:
(Rhodes DJ) Johns Hopkins Hospital and St Agnes Hospital, Baltimore, MD, USA.
Database: PubMed

273. Barriers in cancer pain management.

Author(s): Fazeny B; Muhm M; Hauser I; Wenzel C; Mares P; Berzlanovich A; Hagmeister H; Marosi C

Source: Wiener klinische Wochenschrift; Nov 2000; vol. 112 (no. 22); p. 978-981

Publication Date: Nov 2000

Publication Type(s): Journal Article; Review

ISSN: 0043-5325

Place of Publication: Austria

PubMedID: 11142136

Accession Number: 11142136

Abstract: Inadequate pain management of cancer patients remains a striking problem despite impressive scientific progress in the knowledge of the pathophysiology, pathogenesis and therapy of pain. Our paper focuses on three topics: 1. physician-related barriers, 2. patient-related barriers, and 3. society- and tradition-related barriers as well as government regulations. It is imperative to overcome these barriers, especially since legal regulations for pain management were embodied into statutory regulations in Austria two years ago.

Institutions:
(Fazeny B) Department of Internal Medicine I/Oncology, University of Vienna, Austria. barbara.fazeny@akh-wien.ac.at
Database: PubMed

274. Hospice and hospital oncology unit nurses: a comparative survey of knowledge and attitudes about cancer pain.

Author(s): Hollen CJ; Hollen CW; Stolte K

Source: Oncology nursing forum; 2000; vol. 27 (no. 10); p. 1593-1599

Publication Date: 2000

Publication Type(s): Comparative Study; Journal Article

ISSN: 0190-535X

Place of Publication: United States

PubMedID: 11103378

Accession Number: 11103378

Available at Oncology nursing forum - from EBSCO (CINAHL Complete)

Available at Oncology nursing forum - from David Adams Library Journals Collection Local Print Collection [location] : David Adams Library.

Abstract:PURPOSE/OBJECTIVES: To identify knowledge strengths and weaknesses and misperceptions about cancer pain management between two groups of registered nurses in different settings.DESIGN: Descriptive, comparative survey.SETTING: 11 community-based hospices and 7 inpatient hospital oncology units within an urban county.SAMPLE: A convenience sample of 30 hospice and 34 hospital oncology unit nurses. Sample criteria included registered nurses who had worked for at least the preceding six months exclusively in either a hospice or hospital oncology unit.METHODS: The North Carolina Cancer Pain Initiative survey and a demographic survey were distributed to the work mailboxes of nurses in the participating facilities who met the inclusion criteria.MAIN RESEARCH VARIABLES: Hospice and hospital oncology unit nurses' knowledge and attitudes about basic pharmacologic cancer pain management.FINDINGS: Hospice nurses scored significantly higher than hospital oncology unit nurses regarding overall pain

management knowledge, opioids, scheduling, and liberalness. Hospice nurses also reported more pain education and a higher frequency of pain guideline review requirements than hospital oncology unit nurses.CONCLUSIONS: The most prevalent knowledge deficits concerned opioids. Practice setting and pain education may influence knowledge, as well as attitudes, about pain.IMPLICATIONS FOR NURSING PRACTICE: Further research is needed regarding nurses' pain management behavior and outcomes of pain management education in various settings.

Institutions:
(Hollen CJ) College of Nursing, University of Oklahoma, Oklahoma City, USA. chollen@aol.com

Database: PubMed

275. Physicians' knowledge and attitudes toward the use of analgesics for cancer pain management: a survey of two medical centers in Taiwan.

Author(s): Ger LP; Ho ST; Wang JJ

Source: Journal of pain and symptom management; Nov 2000; vol. 20 (no. 5); p. 335-344

Publication Date: Nov 2000

Publication Type(s): Journal Article; Research Support, Non-U.S. Gov't

DOI: http://dx.doi.org/10.1016/s0885-3924(00)00207-4

ISSN: 0885-3924

Place of Publication: United States

PubMedID: 11068155

Accession Number: 11068155

Available at Journal of pain and symptom management - from David Adams Library Journals Collection Local Print Collection [location] : David Adams Library.

Available at Journal of pain and symptom management - from Unpaywall

Abstract: The purposes of this study were to examine the attitudes of physicians regarding the optimal use of analgesics for cancer pain management (CPM), to evaluate their knowledge and attitudes toward opioid prescribing, and to comprehend their perceptions of the barriers to optimal CPM. A survey was conducted on 356 physicians with cancer patient care responsibilities practicing in two medical centers in Taiwan. A total of 204 (57%) physicians responded, including internists (28%), surgeons (27%), oncologists (11%), anesthesiologists (10%), and other specialties (24%). The majority of physicians displayed significantly inadequate knowledge and negative attitudes toward the optimal use of analgesics and opioid prescribing. Multivariate analyses showed that the following six categories of physicians would be inclined to have inadequate knowledge of opioid prescribing: 1) those with perception of good medical school training in CPM, 2) those with perception of poor residency or fellowship training in CPM, 3) those with a medical specialty in surgery, medicine, or oncology (vs. anesthesiology), 4) those with limited clinical experience in cancer patient care (number of patients less than 30), 5) those with a limited aim of pain relief, and 6) those with an underestimation of analgesic effect. Additionally, physicians with inadequate knowledge of opioid prescribing and

with hesitation to intervene earlier with maximal dose of analgesia would be inclined to have reluctant attitudes toward opioid prescribing. The most important barriers to optimal CPM identified by physicians themselves were physician-related problems, such as inadequate guidance from a pain specialist, inadequate knowledge of CPM, and inadequate pain assessment. The results of this study suggest that active analgesic education programs are urgently needed in Taiwan.

Institutions:
(Ger LP) Department of Medical Education and Research, Kaohsiung Veterans General Hospital, Kaohsiung, Taiwan.
Database: PubMed

276. [Palliative care of the terminal head and neck cancer patient].

Author(s): Kocierz S; Namysłowski G; Nowińska E; Scierski W

Source: Otolaryngologia polska = The Polish otolaryngology; 2000 ; p. 296-300

Publication Date: 2000

Publication Type(s): English Abstract; Journal Article

ISSN: 0030-6657

Place of Publication: Poland

PubMedID: 10974910

Accession Number: 10974910

Abstract: The main problems of treatment of head and neck cancer patient are envisaged in that article. The palliative care principles together with treatment of chronic cancer pain should be not only well-known for family and hospices doctors, who care for patients in terminal stage very frequently, but for head and neck surgeons as well. Insufficient knowledge of cancer pain treatment results in permanent patient's suffering due to pain persistence. The latest theories and viewpoints on treatment of chronic cancer pain with analgetic ladder and adjuvant drugs in particular are described. Patient with head and neck cancer in the terminal stage are more interested in high quality of life assurance than in putting therapy with all possible side-effects.

Institutions:
(Kocierz S) II Katedra i Oddział Kliniczny Laryngologii, SI. AM w Zabrzu.
Database: PubMed

277. Patients' and nurses' assessment of cancer pain.

Author(s): Hovi SL; Lauri S

Source: European journal of cancer care; Dec 1999; vol. 8 (no. 4); p. 213-219

Publication Date: Dec 1999

Publication Type(s): Comparative Study; Journal Article

DOI: http://dx.doi.org/10.1046/j.1365-2354.1999.00171.x

ISSN: 0961-5423

Place of Publication: England

PubMedID: 10889618

Accession Number: 10889618

Available at European journal of cancer care - from Wiley Online Library Medicine and Nursing Collection 2020

Available at European journal of cancer care - from David Adams Library Journals Collection Local Print Collection [location] : David Adams Library.

Abstract: The purpose of this study was to examine hospitalised cancer patients' and nurses' assessment of patients' cancer pain and to compare them. The data were collected from 51 patient-nurse pairs in two hospitals from oncological and medical clinics. Each nurse and patient took part in the study no more than once. The data were collected with a structured interview and the questionnaire. The intensity of pain was measured with a visual analogue scale (VAS) and the Finnish version of the McGill Pain Questionnaire (FPQ). The results showed that the differences between patients' and nurses' assessments were statistically significant for most intensive pain and for acceptable pain. In both cases nurses' assessments of the intensity of pain were lower than patients' assessments. The nurses identified 40 words in the verbal FPQ that the patients used in describing their experiences of pain. The words used most often by patients were agonizing, tender, wave-like and radiant. The word that the nurses used most often was that of intense. Nurses' knowledge about pain medication in general and morphine in particular was clearly associated with the differences observed in estimates of the intensity of pain. Nurses with poor knowledge underestimated the patients' most intensive experiences of pain. The difference was statistically significant.

Institutions:
(Hovi SL) Department of Nursing, University of Turku, Finland.
Database: PubMed

278. Minority cancer patients and their providers: pain management attitudes and practice.

Author(s): Anderson KO; Mendoza TR; Valero V; Richman SP; Russell C; Hurley J; DeLeon C; Washington P; Palos G; Payne R; Cleeland CS

Source: Cancer; Apr 2000; vol. 88 (no. 8); p. 1929-1938

Publication Date: Apr 2000

Publication Type(s): Journal Article; Research Support, Non-U.S. Gov't; Research Support, U.S. Gov't, Non-P.H.S.; Research Support, U.S. Gov't, P.H.S.

ISSN: 0008-543X

Place of Publication: United States

PubMedID: 10760771

Accession Number: 10760771

Available at Cancer - from Wiley Online Library

Available at Cancer - from Unpaywall

Abstract:BACKGROUND: The goals of the current studies were: 1) to determine the pain treatment needs of socioeconomically disadvantaged African-American and Hispanic patients with recurrent or metastatic cancer and 2) to assess the attitudes of health care professionals who treat them.METHODS: In the first study 108 African-American and Hispanic patients with metastatic or recurrent cancer and pain

completed a survey about their pain intensity, pain interference, and attitudes toward analgesic medications. Physicians also rated their patients' pain and the adequacy of the patients' current analgesic prescriptions was assessed. In the second study 55 physicians and nurses who treat these patients completed a questionnaire regarding cancer pain and its management in their practice settings.RESULTS: Approximately 28% of the Hispanic and 31% of the African-American patients received analgesics of insufficient strength to manage their pain. Although the majority of patients received appropriate analgesics, 65% reported severe pain. Physicians underestimated pain severity for 64% of the Hispanic and 74% of the African-American patients. Physicians were more likely to underestimate the pain severity of female patients than male patients. Inadequate pain assessment, patient reluctance to report pain, and lack of staff time were perceived as barriers to pain management.CONCLUSIONS: Although the data suggest recent improvements in analgesic prescribing practices for African-American and Hispanic cancer patients, the majority of patients reported high levels of pain and limited pain relief from analgesic medications. Inadequate pain assessment remains a major barrier to optimal cancer pain treatment.

Institutions:
(Anderson KO) Pain Research Group, The University of Texas M. D. Anderson Cancer Center, Houston, TX 77030, USA.
Database: PubMed

279. Persisting misconceptions of Belgian physicians and nurses about cancer pain treatment.

Author(s): Devulder J

Source: Acta clinica Belgica; Dec 1999; vol. 54 (no. 6); p. 346-350

Publication Date: Dec 1999

Publication Type(s): Journal Article

DOI: http://dx.doi.org/10.1080/17843286.1999.11754258

ISSN: 1784-3286

Place of Publication: England

PubMedID: 10686707

Accession Number: 10686707

Abstract: In Belgium palliative hospices, palliative support teams in hospitals and palliative home care are well-developed. The author gave a lecture about pain treatment in palliative care and inquired after the knowledge and attitudes of 28 nurses and 45 physicians. A questionnaire containing questions about morphine and cancer pain treatment was completed by the attendees before and after the session. In the initial questionnaire the care-providers' attitudes towards palliative care and symptom control were included also. The Wilcoxon test revealed a significant difference in knowledge between the physicians and nurses before the session ($p = 0.007$). Afterwards knowledge had improved in both groups ($p = 0.007$) but a difference still remained ($p = 0.007$). This study reveals that continued education is mandatory. An oral presentation seems not ideal; interactive training with practical exercises might be more appropriate.

Institutions:

(Devulder J) Universitair Ziekenhuis Gent, België. Jacques.devulder@rug.ac.be
Database: PubMed

280. Influencing nurses' knowledge, attitudes, and practice in cancer pain management.

Author(s): Howell D; Butler L; Vincent L; Watt-Watson J; Stearns N

Source: Cancer nursing; Feb 2000; vol. 23 (no. 1); p. 55-63

Publication Date: Feb 2000

Publication Type(s): Comparative Study; Journal Article; Research Support, Non-U.S. Gov't

DOI: http://dx.doi.org/10.1097/00002820-200002000-00009

ISSN: 0162-220X

Place of Publication: United States

PubMedID: 10673808

Accession Number: 10673808

Available at Cancer nursing - from David Adams Library Journals Collection Local Print Collection [location] : David Adams Library.

Abstract: The purpose of this study was to explore the effects of an education intervention on nurses' knowledge, attitudes, and practice in pain assessment and management over 3 months. The education intervention program was designed to change knowledge and influence the attitudes of registered nurses through a values clarification process using a conceptual framework based on a theory of reeducation. Participants in this descriptive, exploratory study were 53 nurses from six oncology units. Data were collected on their knowledge, attitudes, documentation practices, and analgesic choices in defined patient situations. The intervention was effective in changing the knowledge, attitudes, and behaviors of nurses in the study, but the effect was not maintained over time. Study findings suggest that further educational and organizational support is needed for effective practice in pain assessment and management. Further research should explore education programs that will maintain new knowledge over time. In addition, assessment of the effect that new knowledge has on the achievement of improved pain relief for patients should be explored in the future.

Institutions:
(Howell D) Queen Elizabeth II Health Sciences Center, Halifax, Nova Scotia, Canada.
Database: PubMed

281. Concerns and misconceptions about pain among Hong Kong Chinese patients with cancer.

Author(s): Wills BS; Wootton YS

Source: Cancer nursing; Dec 1999; vol. 22 (no. 6); p. 408-413

Publication Date: Dec 1999

Publication Type(s): Journal Article

DOI: http://dx.doi.org/10.1097/00002820-199912000-00002
ISSN: 0162-220X
Place of Publication: United States
PubMedID: 10603687
Accession Number: 10603687

Available at Cancer nursing - from David Adams Library Journals Collection Local Print Collection [location] : David Adams Library.

Abstract: It is estimated that approximately 50% of patients with cancer experience pain, and this percentage increases to 80% in patients with terminal cancer. Misconceptions and concerns of patients with cancer regarding the use of opioid analgesics have been identified as one of the major barriers to achieving optimal pain control. Misconceptions and concerns regarding addiction and tolerance to opioid analgesics and patients' desire to be "good" have been reported in the United States. The aim of this survey was to determine if similar misconceptions and concerns exist in Hong Kong Chinese patients with cancer. The results indicate that Hong Kong Chinese patients have the same concerns regarding the use of opioid analgesics. The respondents' fatalistic beliefs are a major hindrance to optimizing pain control, with 79% indicating that pain is an inevitable aspect of hospitalization because they believe that cancer pain cannot be relieved by medications. Fear of addiction was a major concern for 52% of the respondents, and about the same number of respondents believed that opioid analgesics should be administered only as a last resort. Regarding a desire to be "good," more patients reported that they would prefer to disturb nurses rather than physicians. It is desirable that culturally specific education programs be provided to dispel patient misconceptions and concerns regarding the use of opioid analgesics.

Institutions:
(Wills BS) Chinese University of Hong Kong.
Database: PubMed

282. Testing a multimedia module in cancer pain management.

Author(s): Thompson AR; Savidge MA; Fulper-Smith M; Strode SW

Source: Journal of cancer education : the official journal of the American Association for Cancer Education; 1999; vol. 14 (no. 3); p. 161-163
Publication Date: 1999
Publication Type(s): Journal Article; Research Support, Non-U.S. Gov't
DOI: http://dx.doi.org/10.1080/08858199909528608
ISSN: 0885-8195
Place of Publication: England
PubMedID: 10512333
Accession Number: 10512333

Abstract:BACKGROUND: The technologies for providing better pain management for cancer patients are widely available, but barriers still exist that prevent their optimal use. One of the most important barriers is a knowledge deficit among primary care physicians in adequate pain assessment, in opioid pharmacology, and

in the importance of an interdisciplinary approach to the management of pain.METHOD: The authors prepared a CD-ROM multimedia cancer pain management module to overcome the physician knowledge barrier.RESULTS: Tests of the module with primary care physician and primary care resident volunteers indicate that the cancer pain management multimedia module meets its educational objectives.

Institutions:
(Thompson AR) University of Arkansas for Medical Sciences in Little Rock, Department of Geriatrics, 72205, USA.

Database: PubMed

283. [Attitudes towards terminal care among the general population and medical practitioners in Japan].

Author(s): Miyashita M; Hashimoto S; Kawa M; Kojima M

Source: [Nihon koshu eisei zasshi] Japanese journal of public health; May 1999; vol. 46 (no. 5); p. 391-401

Publication Date: May 1999

Publication Type(s): Clinical Trial; English Abstract; Journal Article; Randomized Controlled Trial; Research Support, Non-U.S. Gov't

ISSN: 0546-1766

Place of Publication: Japan

PubMedID: 10483132

Accession Number: 10483132

Abstract:OBJECTIVES: Assessment of attitudes held by the general population and medical practitioners in Japan regarding medical interventions in cases of painful terminal illness or a prolonged vegetative state.METHOD: A mail survey was conducted in 1998. The subjects were 5,000 persons randomly sampled members of the general population age 20 years or more, and 3,104 doctors and 6,059 nurses in hospitals, clinics, palliative units, and visiting nursing service stations randomly sampled. The response rates were 48% among general population, 51% among doctors, 56% among nurses.RESULTS: 1. 68-76% of the general population and medical practitioners expressed disapproval of life-extending medical treatment of terminal patients suffering pain. The application of euthanasia in certain cases was acceptable to 13% of the general population but only 1% of the medical practitioners. 2. Respondents in almost groups favored a home care setting for terminal patient in pain, and regarded relocation to a palliative unit as acceptable if necessary. 3. 46% of doctors and 22% of nurses indicated knowledge of the WHO method for cancer pain relief, and 45% of doctors and 25% of nurses showed that they were able to explain appropriate opioid administration. 4. 74-79% of general population and medical practitioners opposed life-extending medical intervention for patients in a vegetable state. 26% of the general population favored termination of all means of life support, while about 10% of the medical practitioners held this view. 5. Most medical practitioners felt that some medical treatments, such as bedsore care, should be continued in lieu of life support, but there were differences in opinion between practitioners at various types of medical facilities regarding the necessity of such specific measures as, for example, blood pressure monitoring by automatic

sphygmomanometer among the medical facilities.CONCLUSION: Both the general population and the medical practitioners in Japan tended to oppose life-extending medical treatment for painful terminal cases and patients in a prolonged vegetable state. There are some differences in opinion between the general population and practitioners at various types of medical facilities regarding the extent of desirable medical care in such circumstances.

Institutions:
(Miyashita M) School of Health Sciences and Nursing, Faculty of Medicine, University of Tokyo.

Database: PubMed

284. Evolution of the French public's knowledge and attitudes regarding postoperative pain, cancer pain, and their treatments: two national surveys over a six-year period.

Author(s): Larue F; Fontaine A; Brasseur L

Source: Anesthesia and analgesia; Sep 1999; vol. 89 (no. 3); p. 659-664

Publication Date: Sep 1999

Publication Type(s): Journal Article; Research Support, Non-U.S. Gov't

DOI: http://dx.doi.org/10.1097/00000539-199909000-00023

ISSN: 0003-2999

Place of Publication: United States

PubMedID: 10475300

Accession Number: 10475300

Abstract:UNLABELLED: Pain management has become a notable feature of public health policy and mass media communication in France over the past few years. To assess the evolution of the knowledge and attitudes of the French population with respect to pain management and morphine use, telephone surveys using similar questionnaires were conducted in 1990 (n = 1001) and 1996 (n = 1006). The proportion of respondents who would take pain management adequacy into consideration when selecting a surgical facility increased from 52% to 81% ($P < 0.001$), as did the proportion who associated morphine with pain treatment (from 44% to 80%; $P < 0.001$) or who would not be afraid of becoming addicted to morphine after it had been prescribed for pain relief (from 26% to 69%; $P < 0.001$). However, the proportion of respondents who agreed that morphine can be prescribed to patients with pain increased only slightly. In 1996, 58% of the respondents believed that their knowledge had improved over the past 5 yr and associated this improvement first with television, followed by written press articles and by interaction with physicians. Increased awareness of pain management possibilities among the public may generate increased demand on health professionals to provide adequate and precise information addressing each patient's needs.IMPLICATIONS: The results of two representative surveys conducted over a 6-yr interval show significant improvements of knowledge and attitudes regarding pain and its management in the French general population. However, these results point to the need for additional specific information that should be provided through patient-physician interactions.

Institutions:

(Larue F) Département d'Anesthésie, Hôpital Antoine Béclère, Clamart, France. larue@ext.jussieu.fr
Database: PubMed

285. Patient-related barriers to cancer pain management in a palliative care setting in Hong Kong.

Author(s): Chung TK; French P; Chan S

Source: Cancer nursing; Jun 1999; vol. 22 (no. 3); p. 196-203

Publication Date: Jun 1999

Publication Type(s): Journal Article

DOI: http://dx.doi.org/10.1097/00002820-199906000-00002

ISSN: 0162-220X

Place of Publication: United States

PubMedID: 10376380

Accession Number: 10376380

Available at Cancer nursing - from David Adams Library Journals Collection Local Print Collection [location] : David Adams Library.

Abstract: This article reviews a study of pain management and its barriers in Hong Kong. Using an interview technique, several measures were used to understand the level of concern in patients about pain, the patients' hesitancy in reporting pain, use of analgesics, and adequacy of medication for pain. A total of nine barriers were identified, which include "addiction," "tolerance," "side effects," "physician distraction," "good patient," "fear of injection," "time interval," "fatalism," and "disease progression." Thirty-nine interviews were carried out. The interviewees were all cancer patients with pain in a palliative setting in Hong Kong. When the findings in Taiwan and the United States were compared, it was found that the cancer patients in Hong Kong had a higher level of concern toward the patient-related barriers. It was also found that the level of concern was generally higher in the group with hesitancy in reporting pain and using analgesics. Last of all, this project also identified the educational needs of patients and health care workers in Hong Kong.

Institutions:
(Chung TK) Shatin Hospital, Hong Kong.
Database: PubMed

286. The treatment of chronic cancer pain in a cancer hospital in The Netherlands.

Author(s): de Wit R; van Dam F; Vielvoye-Kerkmeer A; Mattern C; Abu-Saad HH

Source: Journal of pain and symptom management; May 1999; vol. 17 (no. 5); p. 333-350

Publication Date: May 1999

Publication Type(s): Clinical Trial; Journal Article; Randomized Controlled Trial; Research Support, Non-U.S. Gov't

DOI: http://dx.doi.org/10.1016/s0885-3924(98)00150-x

ISSN: 0885-3924

Place of Publication: United States

PubMedID: 10355212

Accession Number: 10355212

Available at Journal of pain and symptom management - from David Adams Library Journals Collection Local Print Collection [location] : David Adams Library.

Available at Journal of pain and symptom management - from Unpaywall

Abstract: In a prospective study of 313 Dutch cancer patients with chronic pain, the practice of pain treatment was evaluated by means of Donabedian's structure-process-outcome framework. The practice of pain treatment was assessed by: (1) structural resources, describing the setting in which pain treatment is provided; (2) process components, which describe the clinical practice; and (3) outcome measures, which refer to patients' pain intensity, patient satisfaction, or composite pain management index scores. Results showed that 31.4-59.8% of the cancer pain patients received less than optimal pain treatment. Although pain education and refresher courses for health care providers are scarce, structural resources were not the major cause of the suboptimal level of pain treatment. Rather, the major cause was the process components. Only 36.4% of the patients received strong opioids; 23.1% received analgesics "as needed." Patients' pain knowledge was far from optimal (54.8 on a 0-100 scale), and written pain information was given to only 15.8% of the patients. After discharge, only 36.8% of the district nurses were informed about patients' pain. These results emphasize that continuing efforts to improve the practice of pain treatment are needed.

Institutions:
(de Wit R) Division of Psychosocial Research and Epidemiology, The Netherlands Cancer Institute/Antoni van Leeuwenhoek Hospital, Amsterdam, The Netherlands.
Database: PubMed

287. [The pharmaceutical care and pain caused by cancer].

Author(s): Hernandez L

Source: Puerto Rico health sciences journal; Mar 1999; vol. 18 (no. 1); p. 47-51

Publication Date: Mar 1999

Publication Type(s): Comparative Study; English Abstract; Journal Article

ISSN: 0738-0658

Place of Publication: Puerto Rico

PubMedID: 10343986

Accession Number: 10343986

Abstract: Pharmaceutical care is the philosophy of practice of the University of Puerto Rico, School of Pharmacy. Pharmacy students enrolled in the Oncology Clerkship providedpharmaceuticalcare to 35 patients with advanced cancer. The objectives were to provide pharmacy students the opportunity to practice pharmaceutical care and to implement published guidelines for the management of cancer pain. Activities included the assessment of pain intensity and of patients' educational needs, identification of drug related problems, intervention with patients

and physicians to solve problems, and documentation of processes and outcomes measures. Thirty three patients had pain due to their disease with an average "worst pain" intensity of 5.5 on a 0-10 scale. Drug related problems were: subtherapeutic dosage, 24.2%; lack of treatment, 18.2%; adverse drug reactions, 12%; improper drug selection, 9.1%; and not taking the prescribed drug, 3%. Outcomes of pharmaceutical services were: reduction of pain, increased knowledge about therapy, improved compliance, improved physician prescribing, and change in cost of medication. Students evaluated the clerkship favorably. The study provides data that supports a randomized trial to further study the impact of pharmaceutical care on the management of cancer pain. Outcomes evaluation should include clinical, humanistic and economic measures.

Institutions:
(Hernandez L) Escuela de Farmacia, Universidad de Puerto Rico.
Database: PubMed

288. Improving cancer pain management using a performance improvement framework.

Author(s): Grant M; Rivera LM; Alisangco J; Francisco L

Source: Journal of nursing care quality; Apr 1999; vol. 13 (no. 4); p. 60-72

Publication Date: Apr 1999

Publication Type(s): Journal Article; Research Support, U.S. Gov't, P.H.S.

DOI: http://dx.doi.org/10.1097/00001786-199904000-00007

ISSN: 1057-3631

Place of Publication: United States

PubMedID: 10330791

Accession Number: 10330791

Available at Journal of nursing care quality - from EBSCO (CINAHL Complete)

Abstract: Pain is an important issue in quality of care and is increasingly cited as an outcome used to evaluate effectiveness of nursing care. Research indicates that nurses are not well prepared to care for patients with pain. Thus many patients are inadequately assessed and treated and consequently receive less than optimal pain management. The article describes the development, implementation, and evaluation of a pain education program designed to provide clinical nurses with the knowledge necessary to use appropriate pain management techniques. Program content stresses the use of a performance improvement framework for changing clinical practice in individual clinical settings.

Institutions:
(Grant M) Department of Nursing Research and Education, City of Hope National Medical Center, Duarte, CA, USA.
Database: PubMed

289. Learning effects of a workshop in palliative cancer care for general practitioners.

Author(s): Schuit KW; Bender W; Meijler WJ; Otter R; Meyboom-Dejong B; Sleijfer DT

Source: Journal of cancer education : the official journal of the American Association for Cancer Education; 1999; vol. 14 (no. 1); p. 18-22

Publication Date: 1999

Publication Type(s): Clinical Trial; Controlled Clinical Trial; Journal Article

DOI: http://dx.doi.org/10.1080/08858199909528568

ISSN: 0885-8195

Place of Publication: England

PubMedID: 10328319

Accession Number: 10328319

Abstract:BACKGROUND: Cancer patients may unnecessarily suffer from pain and other symptoms due to insufficient knowledge on the part of their doctors. In The Netherlands, the general practitioner is considered to be the key provider of palliative care for the cancer patient. Therefore, the authors developed and conducted workshops to teach symptom control to general practitioners. These workshops contained learning objectives from which they selected 18 items to form a questionnaire with five-point response scales. The goal of this study was to investigate changes in the knowledge and attitude scores of the participating general practitioners.METHODS: The participants were asked to complete the questionnaire at the start of the workshop and also four months later.RESULTS: Responses were obtained from 120 general practitioners for the pre-workshop and 96 for the post-workshop questionnaires. The majority of the scores increased toward the desired effect, and some items' scores improved by almost two points.CONCLUSION: The results suggest improvements in general practitioners' knowledge and attitude scores with regard to cancer pain and symptom management. Future studies should try to link these improvements with quality-of-life parameters of terminal cancer patients and their families.

Institutions:
(Schuit KW) Comprehensive Cancer Center North Netherlands.
Database: PubMed

290. Cancer pain: knowledge and attitudes of physicians in Israel.

Author(s): Sapir R; Catane R; Strauss-Liviatan N; Cherny NI

Source: Journal of pain and symptom management; Apr 1999; vol. 17 (no. 4); p. 266-276

Publication Date: Apr 1999

Publication Type(s): Journal Article

DOI: http://dx.doi.org/10.1016/s0885-3924(98)00156-0

ISSN: 0885-3924

Place of Publication: United States

PubMedID: 10203879

Accession Number: 10203879

Available at Journal of pain and symptom management - from David Adams Library Journals Collection Local Print Collection [location] : David Adams Library.

Available at Journal of pain and symptom management - from Unpaywall

Abstract: The effectiveness of cancer pain therapy is influenced by the attitudes and knowledge of the treating physicians. As part of a quality improvement project in the management of cancer pain, a survey of 236 medical practitioners was conducted. One hundred seventy-six respondents (74.5%) completed the survey. Fifty-two percent treated patients with cancer pain several times a week or more. Whereas 57.7% of physicians stated that 76-100% of patients could achieve a satisfactory outcome from analgesic therapy, only 17.2% of respondents reported that > 75% actually achieve a satisfactory outcome in their own experience. Unsatisfactory outcome was ascribed to inadequate pain relief (59.7%), or excessive central nervous system (CNS) side effects (43.3%). According to the responding physicians, the major barriers to effective relief include inadequate assessment of the pain and pain relief (65.3%), inadequate knowledge of pain therapy (57.9%), and physician reluctance to prescribe opioids (49.1%). Questions evaluating physician knowledge identified widely prevalent knowledge deficits in pain physiology, risk of addiction, use of adjuvant analgesics, opioid dosing, and treatment of side effects. Specialists in oncology tended to evaluate their knowledge more highly than others ($P < 0.05$). Despite this, there was no significant knowledge difference between oncologists and noncancer specialists. The data highlight some of the barriers to the successful management of cancer pain in Israel, the prevalence of knowledge deficits, and the common disparity between clinicians' self-assessment of clinical competence and their ability to respond correctly to questions on the management of cancer pain.

Institutions:
(Sapir R) Department of Pharmacy, Shaare Zedek Medical Center, Jerusalem, Israel.
Database: PubMed

291. Cancer and chronic pain.

Author(s): Hassed C

Source: Australian family physician; Jan 1999; vol. 28 (no. 1); p. 17

Publication Date: Jan 1999

Publication Type(s): Journal Article; Review

ISSN: 0300-8495

Place of Publication: Australia

PubMedID: 9988910

Accession Number: 9988910

Abstract:BACKGROUND: The effective management of cancer pain is one of the greatest challenges for GPs.OBJECTIVE: Pain management can be best achieved with a knowledge of the fundamentals of cancer pain and its assessment. A systematic and holistic approach to its treatment needs to be individualised for each patient, taking into account the relevant physical, psychological and spiritual factors.DISCUSSION: GPs are part of a team including oncologists, palliative care specialists, nursing services, paramedical staff, counsellors and clergy and so should know about and make use of the ever-improving support services which are available to ensure that insoluble pain problems are a rare occurrence.

Institutions:
(Hassed C) Monash University Department of Community Medicine.
Database: PubMed

292. Barriers to cancer pain relief: fear of tolerance and addiction.

Author(s): Paice JA; Toy C; Shott S

Source: Journal of pain and symptom management; Jul 1998; vol. 16 (no. 1); p. 1-9

Publication Date: Jul 1998

Publication Type(s): Journal Article; Research Support, Non-U.S. Gov't

DOI: http://dx.doi.org/10.1016/s0885-3924(98)00025-6

ISSN: 0885-3924

Place of Publication: United States

PubMedID: 9707652

Accession Number: 9707652

Available at Journal of pain and symptom management - from David Adams Library Journals Collection Local Print Collection [location] : David Adams Library.

Available at Journal of pain and symptom management - from Unpaywall

Abstract: The purposes of this study were to (a) test the feasibility of the Cancer Total Quality Pain Management (TQPM) Patient Assessment Tool in a population of oncology inpatient and outpatients; and (b) identify factors associated with poor pain relief. The Cancer TQPM Tool was adapted from the American Pain Society's Quality Assurance Standards on Acute Pain and Cancer Pain and was tested in a convenience sample of 200 patients. The majority of patients reported that the TQPM Tool was easy to understand and to use, providing evidence for the feasibility of the tool. Factors associated with higher pain intensity included the inpatient setting, the presence of metastatic disease, hesitancy in bothering the nurse, and concerns regarding tolerance and addiction. Although there was a strong relationship between concern about addiction and concern about tolerance, fear of tolerance appeared to have a greater effect on pain intensity scores than did fear of addiction. The findings from this study suggest that the Cancer TQPM Patient Assessment Tool can be used effectively in both inpatients and outpatients to determine outcomes and the quality of cancer pain management, as well identify factors associated with poor pain control. Clinical implications include more effective education of patients and caregivers, including equivalent emphasis on tolerance and addiction.

Institutions:
(Paice JA) Department of Neurosurgery, Rush Neuroscience Institute, Rush Medical Center, Chicago, IL 60612, USA.
Database: PubMed

293. Knowledge and attitudes of health-care providers toward cancer pain management: a comparison of physicians, nurses, and pharmacists in the state of New Hampshire.

Author(s): Furstenberg CT; Ahles TA; Whedon MB; Pierce KL; Dolan M; Roberts L; Silberfarb PM

Source: Journal of pain and symptom management; Jun 1998; vol. 15 (no. 6); p. 335-349

Publication Date: Jun 1998

Publication Type(s): Comparative Study; Journal Article; Research Support, Non-U.S. Gov't; Research Support, U.S. Gov't, P.H.S.

DOI: http://dx.doi.org/10.1016/s0885-3924(98)00023-2

ISSN: 0885-3924

Place of Publication: United States

PubMedID: 9670634

Accession Number: 9670634

Available at Journal of pain and symptom management - from David Adams Library Journals Collection Local Print Collection [location] : David Adams Library.

Available at Journal of pain and symptom management - from Unpaywall

Abstract: The knowledge and attitudes toward cancer pain management of physicians, nurses, and pharmacists in the state of New Hampshire were examined through the use of a statewide survey. Many of the providers who completed the survey, and thus indicated that they treated patients with cancer pain on a regular basis, were not pain or oncology specialists. Most of these providers were quite well informed about the fundamentals of cancer pain management. Approximately 90% of providers in all three groups were not concerned about addiction among cancer patients. Yet, there was a small percentage of providers who responded in less than optimal ways to items dealing with opioid pharmacology, pain assessment, and the importance of pain relief. Comparison of responses among provider groups indicated that nurses were the most knowledgeable and pharmacists the least knowledgeable about pain assessment. Physicians were the most knowledgeable regarding opioid pharmacology but seemed the least committed to providing optimal pain relief. Further analysis identified a small group of physicians that included a disproportionately high percentage of family practitioners and surgeons who consistently responded in less than optimal ways to items dealing with the importance of pain relief. The results of this study indicate a continuing need for broad-based educational programs in cancer pain management and for new initiatives focused on practitioners who see relatively few cancer patients and may have difficulty accessing traditional educational programs.

Institutions:
(Furstenberg CT) Center for Psycho-Oncology Research, Dartmouth Hitchcock Medical Center, Lebanon, NH 03756, USA.
Database: PubMed

294. Cancer pain survey: patient-centered issues in control.

Author(s): Thomason TE; McCune JS; Bernard SA; Winer EP; Tremont S; Lindley CM

Source: Journal of pain and symptom management; May 1998; vol. 15 (no. 5); p. 275-284

Publication Date: May 1998

Publication Type(s): Journal Article; Research Support, U.S. Gov't, P.H.S.

DOI: http://dx.doi.org/10.1016/s0885-3924(98)00016-5
ISSN: 0885-3924
Place of Publication: United States
PubMedID: 9654832
Accession Number: 9654832

Available at Journal of pain and symptom management - from David Adams Library Journals Collection Local Print Collection [location] : David Adams Library.

Available at Journal of pain and symptom management - from Unpaywall

Abstract: It is widely believed that patients' reluctance to report pain and adhere to treatment recommendations are significant barriers to cancer pain control. However, few investigators have examined barriers to cancer pain management from the cancer patient's perspective. Ambulatory patients with cancer who had experienced cancer-related pain in the previous month or were currently taking analgesics for cancer pain control were asked to participate in this study. Information regarding (a) pain assessment, (b) pain medication use, (c) concerns and barriers to compliance, (d) communication patterns regarding pain and pain control, and (e) demographics were collected during a 10-min structured interview. Approximately 20% of patients with a current cancer diagnosis who were approached reported that they had experienced pain or taken analgesic drugs during the preceding month. Eighty-eight percent of these patients ranked their pain as five or greater (scale, 0-10), and 81% reported impaired function due to pain. Major barriers to effective treatment included forgetfulness, the belief that pain should be tolerated, concerns about side effects, and fear and disdain of dependence, addiction, and tolerance. One-third of patients felt that their pain could not be better controlled than it currently was. Patients reported frequent communication regarding pain and pain control with physicians (52%), nurses (41%), and pharmacists (17%). The low pain prevalence, coupled with high pain intensity and associated dysfunction, appears to be a reflection of patient's unwillingness to report pain of mild to moderate intensity. In addition to previously recognized factors, stoicism and fatalism represent significant barriers to cancer pain control.

Institutions:
(Thomason TE) University of North Carolina Hospitals, Chapel Hill, Durham, USA.
Database: PubMed

295. Regulatory barriers to pain management.

Author(s): Joranson DE; Gilson AM

Source: Seminars in oncology nursing; May 1998; vol. 14 (no. 2); p. 158-163
Publication Date: May 1998
Publication Type(s): Journal Article; Review
DOI: http://dx.doi.org/10.1016/s0749-2081(98)80022-3
ISSN: 0749-2081
Place of Publication: United States
PubMedID: 9580940
Accession Number: 9580940

Available at Seminars in oncology nursing - from David Adams Library Journals Collection Local Print Collection [location] : David Adams Library.

Abstract:OBJECTIVES: To provide an overview of relevant federal and state policies, as well as recommendations for identifying and addressing barriers to the treatment of cancer and non-cancer pain.DATA SOURCES: Review of federal and state statutes and medical board guidelines.CONCLUSIONS: There has been an increase in pain-related policies since the mid 1980s, with recent years showing a significant amount of policy development and adoption. However, a variety of laws and policies contain provisions that have the potential to discourage the use of opioid analgesics for the relief of pain.IMPLICATIONS FOR NURSING PRACTICE: The evaluation and treatment of patients with cancer and non-cancer pain can be enhanced by a knowledge of the specific restrictions of controlled substances statutes and practice guidelines. In this way, there will be less chance for nurses to practice outside established legal parameters.

Institutions:
(Joranson DE) Pain and Policy Studies Group, University of Wisconsin Comprehensive Cancer Center, Madison 53705, USA.
Database: PubMed

296. An examination of nursing attitudes and pain management practices.

Author(s): Dalton JA; Carlson J; Mann JD; Blau W; Bernard S; Youngblood R

Source: Cancer practice; 1998; vol. 6 (no. 2); p. 115-124

Publication Date: 1998

Publication Type(s): Clinical Trial; Controlled Clinical Trial; Journal Article; Research Support, U.S. Gov't, P.H.S.

DOI: http://dx.doi.org/10.1046/j.1523-5394.1998.1998006115.x

ISSN: 1065-4704

Place of Publication: United States

PubMedID: 9573911

Accession Number: 9573911

Available at Cancer practice - from EBSCO (MEDLINE Complete)

Available at Cancer practice - from EBSCO (CINAHL Complete)

Abstract:PURPOSE: The purpose of this evaluation is to examine the relationship among nurses' pain management attitudes and pain management practices and to begin to explore the theoretical underpinnings that may influence this relationship.DESCRIPTION OF STUDY: A convenience sample of 29 female registered nurses working in hospice or home health settings participated in an educational program 1 day per week for 6 weeks. All participants were asked to complete the Cancer Pain Knowledge Inventory and Survey of Expectations and Pain Assessment Questionnaire 5 weeks before, immediately before, immediately after, 6 months after, and 12 months after the program. Seventeen participants completed all questionnaires at the 6-month follow-up; 16 participants completed all questionnaires at the 1-year follow-up. Personal beliefs about pain were evaluated in relation to the dimensions and treatment of pain. Intentions and expectations to perform specific activities were evaluated in relation to in-depth assessments,

equianalgesic conversions, demonstration of new ideas, and communication.RESULTS: Nurses' attitudes, beliefs, intentions, and expectations about pain and pain management influenced nurses' patient care and educational activities. Nurses who believed that patients should be pain free and nurses who focused on both the dimensions and treatment of pain implemented more pain management activities. In general, nurses who had high intentions and expectations performed more pain management activities.CLINICAL IMPLICATIONS: Although nurses reported change in attitude, and high expectancy for change, feelings of increased credibility, and increased motivation as advocates for new approaches to practice, nurses sometimes found it difficult to implement new practices because of constraints in time and collaborative efforts. To implement new knowledge and achieve individualized goals for change, nurses must be allowed adequate time to analyze the relationships between their beliefs about pain and the ways that they solve patients' pain problems. In addition, more support for multidisciplinary collaboration is needed.

Institutions:
(Dalton JA) Department of Adult and Geriatric Health, School of Nursing, University of North Carolina at Chapel Hill 27599, USA.
Database: PubMed

297. [Indications and limits of nerve block techniques].

Author(s): Donner B; Schnell P; Zenz M

Source: Zeitschrift fur arztliche Fortbildung und Qualitatssicherung; Jan 1998; vol. 92 (no. 1); p. 29-33

Publication Date: Jan 1998

Publication Type(s): English Abstract; Journal Article; Review

ISSN: 1431-7621

Place of Publication: Germany

PubMedID: 9553210

Accession Number: 9553210

Abstract: Repetitive nerve blocks as a monotherapeutic treatment are loosing importance in the therapy of chronic pain. Such invasive methods for pain reduction are just one strategy in the interdisciplinary and multimodal planning of pain therapy. They are mostly used in special indications, e.g. reflex sympathetic dystrophy neurolysis in S3-S5 localized cancer pain. Premises for an invasive pain therapy are the patient's knowledge and agreement concerning this method. Furthermore, it is necessary for the physician to know the typical complications of the invasive treatment and to be able to manage them. It is recommended to document the pain course.

Institutions:
(Donner B) Klinik für Anaesthesiologie, Intensiv- und Schmerztherapie, Universitätsklinik Bochum.
Database: PubMed

298. A pain education program for chronic cancer pain patients: follow-up results from a randomized controlled trial.

Author(s): de Wit R; van Dam F; Zandbelt L; van Buuren A; van der Heijden K; Leenhouts G; Loonstra S

Source: Pain; Oct 1997; vol. 73 (no. 1); p. 55-69

Publication Date: Oct 1997

Publication Type(s): Clinical Trial; Journal Article; Multicenter Study; Randomized Controlled Trial; Research Support, Non-U.S. Gov't

DOI: http://dx.doi.org/10.1016/s0304-3959(97)00070-5

ISSN: 0304-3959

Place of Publication: United States

PubMedID: 9414057

Accession Number: 9414057

Abstract: The effectiveness of a Pain Education Program in cancer patients with chronic pain offered by nurses was investigated in a randomized controlled clinical trial. A multi-method approach was used in which verbal instruction, written material, an audio cassette tape, and the use of a pain diary were combined to inform and instruct patients about pain and pain management. The Pain Education Program was tailored to the needs of the individual patient and consisted of three elements: (1) educating patients about the basic principles regarding pain and pain management; (2) instructing patients how to report their pain in a pain diary; and (3) instructing patients how to communicate about pain and how to contact health care providers. Following pretesting in 313 patients, patients who needed district nursing and who did not need district nursing at home were randomly assigned to a control or intervention group. Intervention group patients received the Pain Education Program in the hospital, and 3 and 7 days postdischarge by telephone; this was done by nurses who were specially trained as pain counselors. Follow-up assessments were at 2, 4 and 8 weeks postdischarge. Results of the pretest showed that many patients lacked knowledge about pain and pain management. The majority of pain topics had to be discussed. The Pain Education Program proved to be feasible: 75.0% of the patients had read the entire pain brochure, 55.7% had listened to the audio cassette, and 85.6% of pain scores were completed in the pain diary. Results showed a significant increase in pain knowledge in patients who received the Pain Education Program and a significant decrease in pain intensity. However, pain relief was mainly found in the intervention group patients without district nursing. It can be concluded that the tailored Pain Education Program is effective for cancer patients in chronic pain. The use of the Pain Education Program by nurses should be seriously considered on oncology units.

Institutions:
(de Wit R) Division of Psychosocial Research and Epidemiology, The Netherlands Cancer Institute/Antoni van Leeuwenhoek Hospital, Amsterdam. rdewit@nki.nl
Database: PubMed

299. Patients' knowledge of and attitudes toward the management of cancer pain.

Author(s): Riddell A; Fitch MI

Source: Oncology nursing forum; 1997; vol. 24 (no. 10); p. 1775-1784

Publication Date: 1997

Publication Type(s): Journal Article

ISSN: 0190-535X

Place of Publication: United States

PubMedID: 9399275

Accession Number: 9399275

Available at Oncology nursing forum - from David Adams Library Journals Collection Local Print Collection [location] : David Adams Library.

Abstract:PURPOSE/OBJECTIVES: To examine patients' knowledge of and attitudes toward the management of cancer pain and to identify, from the patients' perspectives, factors contributing to effective and ineffective pain relief.DESIGN: Descriptive, correlational.SETTING: Ambulatory care oncology facility in Canada.SAMPLE: Convenience sample of 42 patients receiving oral pain medication for chronic cancer-related pain.METHODS: Participants completed a modified version of the Patient Pain Questionnaire and a demographic questionnaire and responded to two open-ended questions.MAIN RESEARCH VARIABLES: Patients' knowledge of and attitudes toward cancer pain management and their perceptions of factors contributing to effective and ineffective pain relief.FINDINGS: Many patients locked knowledge of the principles involved in effective cancer pain management and had unrealistic concerns about taking pain medications. Significant negative relationships were found between pain intensity ratings and factors such as patients' knowledge of pain management, their level of satisfaction with pain relief, and their perception of the goal of pain management. Patients identified a number of impediments to effective pain relief, including concerns about addiction and various side effects to pain medications.CONCLUSIONS: Many patients have inadequate knowledge about the management of cancers pain and have unrealistic concerns about taking pain medications, both of which have been identified in the literature as barriers to effective cancer pain management.IMPLICATIONS FOR NURSING PRACTICE: A need exists for patient education that addresses patients' misconceptions and concerns about using pain medications and the principles involved in effective cancer pain management.

Institutions:
(Riddell A) Princess Margaret Hospital, Toronto, Ontario, Canada.
Database: PubMed

300. Attitudes of Italian general practitioners in the treatment of cancer pain. The Committee of the Associazione Italiana di Oncologia Medica (AIOM).

Author(s): Minotti V; Betti M

Source: Tumori; 1997; vol. 83 (no. 4); p. 729-731

Publication Date: 1997

Publication Type(s): Journal Article

ISSN: 0300-8916

Place of Publication: United States

PubMedID: 9349310

Accession Number: 9349310

Abstract: The attitude of Italian general practitioners in prescribing practices for patients with cancer pain was assessed by means of a questionnaire. The results indicated that among most of the doctors who completed the questionnaire the basic principles of pain treatment in cancer patients are largely understood. Oral morphine emerged as the most commonly used opioid (60%) and controlled-release morphine as the preferred preparation. Non-steroidal anti-inflammatory drugs were the most commonly used minor analgesics. Fear of side effects and restrictive prescribing regulations emerged as the most important barrier against adequate pain management. The survey emphasised the need for continued efforts in implementing specific educational programming for improvement in cancer pain management.

Institutions:
(Minotti V) Oncologia Medica, Policlinico Monteluce, Perugia, Italy.

Database: PubMed

Strategy 1081122

#	Database	Search term	Results
1	CINAHL	("cancer pain").ti,ab	4851
2	CINAHL	"CANCER PAIN"/	5814
3	CINAHL	(1 OR 2)	8497
4	CINAHL	("health professional*" OR "health care professional*" OR "healthcare professional*" OR nurs* OR doctor* OR "allied health" OR physician).ti,ab	792977
5	CINAHL	exp "HEALTH PERSONNEL"/	599126
6	CINAHL	(4 OR 5)	1167496
7	CINAHL	(knowledge OR attitud* OR barrier*).ti,ab	372439
8	CINAHL	(6 AND 7)	127775
9	CINAHL	exp "ATTITUDE OF HEALTH PERSONNEL"/	108317
10	CINAHL	(8 OR 9)	211460
11	CINAHL	(3 AND 10)	690
12	CINAHL	exp "ATTITUDE OF HEALTH PERSONNEL"/ [DT 2010-2020]	65342
13	CINAHL	11 [DT 2010-2020]	304
14	CINAHL	11 [DT 2010-2020] [Languages eng]	286
15	PubMed	("cancer pain").ti,ab	0
16	PubMed	"CANCER PAIN"/	0
17	PubMed	("cancer pain").ti,ab	8273

18	PubMed	"CANCER PAIN"/	0
19	PubMed	(17 OR 18)	8273
20	PubMed	("health professional*" OR "health care professional*" OR "healthcare professional*" OR nurs* OR doctor* OR "allied health" OR physician).ti,ab	794153
21	PubMed	exp "HEALTH PERSONNEL"/	0
22	PubMed	("health professional*" OR "health care professional*" OR "healthcare professional*" OR nurs* OR doctor* OR "allied health" OR physician).ti,ab	794153
23	PubMed	exp "HEALTH PERSONNEL"/	0
24	PubMed	(22 OR 23)	794153
25	PubMed	(knowledge OR attitud* OR barrier*).ti,ab	1206771
26	PubMed	("health professional*" OR "health care professional*" OR "healthcare professional*" OR nurs* OR doctor* OR "allied health" OR physician).ti,ab	794153
27	PubMed	exp "HEALTH PERSONNEL"/	0
28	PubMed	(26 OR 27)	794153
29	PubMed	(knowledge OR attitud* OR barrier*).ti,ab	0
30	PubMed	(28 AND 29)	109975
31	PubMed	exp "ATTITUDE OF HEALTH PERSONNEL"/	0
32	PubMed	("health professional*" OR "health care professional*" OR "healthcare professional*" OR nurs* OR doctor* OR "allied	794153

		health" OR physician).ti,ab	
33	PubMed	exp "HEALTH PERSONNEL"/	0
34	PubMed	(32 OR 33)	794153
35	PubMed	(knowledge OR attitud* OR barrier*).ti,ab	0
36	PubMed	(34 AND 35)	109975
37	PubMed	exp "ATTITUDE OF HEALTH PERSONNEL"/	0
38	PubMed	(36 OR 37)	109975
39	PubMed	("cancer pain").ti,ab	8273
40	PubMed	"CANCER PAIN"/	0
41	PubMed	(39 OR 40)	8273
42	PubMed	("health professional*" OR "health care professional*" OR "healthcare professional*" OR nurs* OR doctor* OR "allied health" OR physician).ti,ab	794153
43	PubMed	exp "HEALTH PERSONNEL"/	0
44	PubMed	(42 OR 43)	0
45	PubMed	(knowledge OR attitud* OR barrier*).ti,ab	1206771
46	PubMed	(44 AND 45)	0
47	PubMed	exp "ATTITUDE OF HEALTH PERSONNEL"/	0
48	PubMed	(46 OR 47)	0
49	PubMed	(41 AND 48)	352
50	PubMed	exp "ATTITUDE OF HEALTH PERSONNEL"/	0

51	PubMed	("cancer pain").ti,ab	8273
52	PubMed	"CANCER PAIN"/	0
53	PubMed	(51 OR 52)	8273
54	PubMed	("health professional*" OR "health care professional*" OR "healthcare professional*" OR nurs* OR doctor* OR "allied health" OR physician).ti,ab	0
55	PubMed	exp "HEALTH PERSONNEL"/	0
56	PubMed	(54 OR 55)	794153
57	PubMed	(knowledge OR attitud* OR barrier*).ti,ab	0
58	PubMed	(56 AND 57)	109975
59	PubMed	exp "ATTITUDE OF HEALTH PERSONNEL"/	0
60	PubMed	(58 OR 59)	0
61	PubMed	(53 AND 60)	0
62	PubMed	61	0
63	PubMed	("cancer pain").ti,ab	8273
64	PubMed	"CANCER PAIN"/	0
65	PubMed	(63 OR 64)	8273
66	PubMed	("health professional*" OR "health care professional*" OR "healthcare professional*" OR nurs* OR doctor* OR "allied health" OR physician).ti,ab	794153
67	PubMed	exp "HEALTH PERSONNEL"/	0
68	PubMed	(66 OR 67)	0

69	PubMed	(knowledge OR attitud* OR barrier*).ti,ab	1206771
70	PubMed	(68 AND 69)	109975
71	PubMed	exp "ATTITUDE OF HEALTH PERSONNEL"/	0
72	PubMed	(70 OR 71)	0
73	PubMed	(65 AND 72)	0
74	PubMed	73	352
75	PubMed	74 [DT 2010-2020]	0
