# Supplementary material for: Cancer pain knowledge and attitudes of healthcare professionals: A systematic review of surveys and their measurement properties
Source: Br J Pain. 2026 Apr 13:20494637261442745. Online ahead of print. doi: 10.1177/20494637261442745 (PMC13076462; doi:10.1177/20494637261442745)
Supplement: Supplemental material - Cancer pain knowledge and attitudes of healthcare professionals: A systematic review of surveys and their measurement properties [file sj-pdf-2-bjp-10.1177_20494637261442745.pdf]

Supplementary Information 2: Inclusion / Exclusion criteria

Inclusion	Exclusion
Studies that aim to measure the knowledge and/or attitudes of healthcare professional regarding cancer-related pain.<br><br>Studies with registered healthcare professionals caring for adults over the age of 18 years old with a cancer diagnosis.<br><br>Quantitative study design.	Studies that measure the knowledge of non-cancer-related pain.<br><br>Studies with registered healthcare professionals caring for adults under the age of 18 years old with or without a cancer diagnosis.<br><br>Non-registered ore pre-licensure healthcare workers caring for people with or without a cancer diagnosis.<br><br>Studies examining professionals practice, reported practice or attitude towards opioids in practice<br><br>Qualitative and mixed methods design studies.<br><br>Published in alternative languages with no English translation available
