# Supplementary material for: Cancer pain knowledge and attitudes of healthcare professionals: A systematic review of surveys and their measurement properties
Source: Br J Pain. 2026 Apr 13:20494637261442745. Online ahead of print. doi: 10.1177/20494637261442745 (PMC13076462; doi:10.1177/20494637261442745)
Supplement: Supplemental material - Cancer pain knowledge and attitudes of healthcare professionals: A systematic review of surveys and their measurement properties [file sj-pdf-3-bjp-10.1177_20494637261442745.pdf]

Supplementary Information 4 KASRP Survey Modifications

**Study**<br>1. Author<br>2. Date<br>3. Location	**Language**	**No. Of Questions**	**Score required to demonstrate "Good" knowledge level**	**Modifications**	**Additional psychometric testing performed?**
**Original Version: Ferrell (2014) Knowledge and Attitudes Survey Regarding Pain (www.cityofhope.org/NRE/resources), revised 2014.**					
Ferrell (2014)	English	39	Not stated	N/A	
1. Gustafsson & Borglin [38]<br>2. 2013<br>3. Sweden	Back translated into Swedish	38 items	Not stated	9 & 18 removed	No
1. Shahriary et al [17]<br>2. 2015<br>3. Iran	English*	39	80%	No Modifications stated	No
1. Beck et al [44]<br>2. 2016<br>3. USA	English	40	80%	No Modifications stated	No
1. Kasasbeh et al [39]<br>2. 2016<br>3. Ireland	English	40	Not stated	No Modifications stated	No
1. Tufail et al [18]<br>2. 2017<br>3. Pakistan	English*	36	70%	No Modifications stated	No
1. Utne et al [40]<br>2. 2018<br>3. Norway	English*	41	75%	Reports using Norwegian version of the KASRP however the reference used is for Ferrell (2014). No details given regarding additional questions or translation.	No
1. Al-Atiyyat et al [19]<br>2. 2019<br>3. UAE	English*	39	80%	No Modifications stated	No
1. Alnajar et al [20]<br>2. 2019<br>3. Jordan	English*	39	50 – 75% Fair Knowledge<br>75% Good Knowledge**	Stated a modified version was used but did not outline the modifications	No
1. Cowperthwaite et al [45]<br>2. 2019<br>3. USA	English	41	Not stated	Stated that 4 questions were removed bringing the total down to 37 questions.	No
1. Darawad et al [21]<br>2. 2019<br>3. Jordan	English*	39	50 – 75% Fair Knowledge<br>75% Good Knowledge**	No Modifications stated	No
1. Admass et al [48]<br>2. 2020<br>3. Ethiopia	English (acknowledged this was a second language for participants)	41	80%	No Modifications stated	No
1. El-Aqoul et al [23]<br>2. 2020<br>3. Jordan	English*	39	50 – 75% Fair Knowledge<br>75% Good Knowledge**	Stated 39 items used	No
1. Yassin et al [24]<br>2. 2020<br>3. Qatar	English*	39	80%	No Modifications stated	No
1. Yu et al [31]<br>2. 2020<br>3. China	Translated into Chinese	41	80%	Chinese Version however has used the reference for Ferrell (2014).<br>Item 16, 32a replaced to reflect local practice	No

1. Hadjisavva et al [16]<br>2. 2021<br>3. Cyprus	Greek translation	37	Not stated	No details given	No
1. Li et al [33]<br>2. 2021<br>3. China	English*	41	80%	No Modifications stated	No
1. Omer & Nematala [25]<br>2. 2022<br>3. Iraq	English*	34	Not stated	Stated that modifications where made however no details given.	Yes – no details given regarding results.
1. Zaabi et al [55]<br>2. 2023<br>3. Oman	English*	36	<50% Poor<br>50 – 70% Fair<br>>70% Good	No Modifications stated	No

*Assumption was made that the tool was used in its original English as no mention of translation stated.
** Standard was taken from Al-Khawaldeh et al (2013) [54]
