# Supplementary material for: Cancer pain knowledge and attitudes of healthcare professionals: A systematic review of surveys and their measurement properties
Source: Br J Pain. 2026 Apr 13:20494637261442745. Online ahead of print. doi: 10.1177/20494637261442745 (PMC13076462; doi:10.1177/20494637261442745)
Supplement: Supplemental material - Cancer pain knowledge and attitudes of healthcare professionals: A systematic review of surveys and their measurement properties [file sj-pdf-4-bjp-10.1177_20494637261442745.pdf]

Supplementary Information 5 Feasibility characteristics

Tool / Author	No of Questions	Type of Questions	Target Population	Time to:<br>1. Complete<br>2. Score	1. Literacy level<br>2. Language	Ease of Administration	1. Cost of Instrument<br>2. Availability of Instrument
Knowledge and Attitudes Survey Regarding Pain [8]	39	True/False<br>Multiple Choice<br>Vignette	Healthcare professionals	1. Not stated<br>2. Not stated	1. Not stated<br>2. English	Self-directed completion	1. Free<br>2. Open Access online
Nurses' knowledge about the management of cancer pain – WHO [49]*	24	Likert-type items	Healthcare professionals, professional groups not stated.	1. Not stated<br>2. Not stated	1. Not stated<br>2. Spanish	Self-directed completion	1. Unclear<br>2. Unable to locate
Jho (2014) [30]	14 Knowledge Questions	Yes / No	Healthcare Professionals	1. Not Stated<br>2. Not stated	1. Not Stated<br>2. English, Arabic	Self-directed completion	1. Free<br>2. Available in Toba (2019) publication.
Breuer et al (2015) [43]	Survey 1 – 4<br>Survey 2 – 4	Vignette<br>Multiple Choice	Oncologists	1. 20mins<br>2. Not stated	1. Not stated<br>2. English	Self-directed completion	1. Free<br>2. Available in publication.
Cheng & Yu (2011)* [28]	45	5 Subjective items<br>30 single choice<br>10 multiple choice	Medical Students	1. Not Stated<br>2. Not stated	1. Not Stated<br>2. Not Stated	Self-directed completion	1. Unclear<br>2. Unable to locate
Garcia-Mata et al (2018)* [41]	77 (full version)<br>25 (abridged)	Closed questions	Physicians	1. Not stated<br>2. Not stated	1. Not stated<br>2. Not stated	Self-directed completion	1. Unclear<br>2. Unable to locate
Hashemi (2015)* [16]	54	Unclear	Physicians	1. Not stated<br>2. Not stated	1. Not stated<br>2. Not stated	Self-directed completion	1. Unclear<br>2. Unable to locate
Weissman & Dahl (1990) [50] used in Kaki (2011)	18	Closed and Open ended.	Medical Students	1. Not stated<br>2. Not stated	1. Not stated<br>2. Not stated	Self-directed completion	1. Free<br>2. Available in publication Weissman & Dahl (1990)

Tool / Author	No of Questions	Type of Questions	Target Population	Time to:<br>1. Complete<br>2. Score	1. Literacy level<br>2. Language	Ease of Administration	1. Cost of Instrument<br>2. Availability of Instrument
Kim (2014) [29]	20	Yes / No and Multiple choice	Physicians	1. Not stated<br>2. Not stated	1. Not stated<br>2. Not stated	Self-directed completion	1. Free<br>2. Available in publication
Kim (2011)* [27]	29	Two/three-degree scales, Likert scales and yes/no questions	Physicians	1. Not stated<br>2. Not stated	1. Not stated<br>2. Not stated	Self-directed completion	1. Unclear<br>2. Unable to locate
Kwok (2021)* [32]	24	Multiple choice, unknown other types	Nurses	1. 20mins<br>2. Not stated	1. Not stated<br>2. Not stated	Self-directed completion	1. Unclear<br>2. Unable to locate
Piano (2013) [37]	16	Vignette with multiple choice options	Physicians	1. Not stated<br>2. Not stated	1. Not stated<br>2. French, English	Self-directed completion	1. Free<br>2. Available within publication
Su (2021) [34]	40	Multiple Choice	Healthcare Professionals	1. Not stated<br>2. Not stated	1. Not Stated<br>2. English	Self-directed completion	1. Free<br>2. Available within publication
Xie (2022) [35]	30	Multiple Choice<br>Single Choice	Physician, Nurse & Pharmacist	1. Not stated<br>2. Not stated	1. Not stated<br>2. Not stated	Self-directed completion	1. Unclear<br>2. Unable to locate
Yu (2022) [36]	51	Likert scales<br>Multiple choice<br>True / False	Physician, Nurse & Pharmacist	1. Not stated<br>2. Not stated	1. Not stated<br>2. English	Self-directed completion	1. Unclear<br>2. Available within publication
Arahata et al (2018) [56] used in Shimizu et al (2024) [57]	10*	Multiple choice options of “correct”, “incorrect” or “don’t know”.	Nurses	1. Not stated<br>2. Not stated	1. Not stated<br>2. Not stated	Self-directed completion	1. Unclear<br>2. Available within publication

*Please note that a revised version of the quiz was used in Shimizu [57] and only 10 questions used, in the full version of the tool developed by Arahata [56] there are 90 questions.
